# Platelet Proteome Links Metabolism to Reactivity in Essential Thrombocythemia

**DOI:** 10.1016/j.mcpro.2026.101617

**Published:** 2026-06-29

**Authors:** Xiomara Guerrero-Carreño, Sanne Smits, Alfonso Esteban Lasso, Martina Samiotaki, Virtu Calabuig-Navarro, Francisco José Iborra, Frida Rantanen, Alberto Álvarez-Larrán, Anna Angona Figueras, Beatriz Bellosillo, Adolfo J. Sáez Marín, Valentín García-Gutiérrez, Dick H.W. Dekkers, Jeroen A.A. Demmers, Francisca Ferrer-Marín, Juan Carlos Hernández-Boluda, Antonios Matsakas, Celina Benavente Cuesta, Peter Vandenberghe, Petros Papadopoulos

**Affiliations:** 1 Department of Hematology, Cellular differentiation and gene expression, Instituto de Investigación Sanitaria San Carlos (IdISSC), Hospital Clínico San Carlos, Madrid, Spain; 2 Department of Hematology, Hospital Clínico San Carlos, Madrid, Spain; 3 Center for Human Genetics, KU Leuven, Leuven, Belgium; 4 University Rey Juan Carlos, Madrid, Spain; 5 Institute for Bioinnovation, BSRC “Al. Fleming”, Vari, Greece; 6 Biological Noise and Cell Plasticity, IBV (CSIC), Valencia, Spain; 7 Medical Systems Biology, University of Helsinki, Helsinki, Finland; 8 Department of Hematology, Hospital del Mar, Barcelona, Spain; 9 Department of Hematology, Hospital Clínic, Barcelona, Spain; 10 Institut Català d'Oncologia, Hospital Josep Trueta, Girona, Spain; 11 Instituto Ramón y Cajal de Investigación Sanitaria, Universidad de Alcala, Madrid, Spain; 12 Proteomics Center, Erasmus University Medical Center, Rotterdam, The Netherlands; 13 Department of Hematology, Hospital General Universitario Morales Meseguer, CIBERER, IMIB, UCAM, Murcia, Spain; 14 Department of Hematology, Hospital Clínico Universitario, INCLIVA, Valencia, Spain; 15 Department of Life Sciences, Manchester Metropolitan University, Manchester, UK; 16 Department of Hematology, University Hospitals Leuven, Leuven, Belgium

**Keywords:** essential thrombocythemia, platelet proteome, platelet activation, calreticulin, JAK2 V617F

## Abstract

Essential thrombocythemia (ET) is a myeloproliferative neoplasm in which JAK2, CALR, and MPL mutations are associated with distinct clinical phenotypes. Here, we analyzed the platelet proteome of ET patients by MS, combined with functional assays, to investigate platelet activation. Compared with healthy controls, ET platelets showed altered abundance of mitochondrial and metabolic proteins, including reduced levels of tricarboxylic acid cycle enzymes and a relative enrichment of glycolytic signatures. Acetylsalicylic acid decreased the abundance of metabolic proteins in *CALR* Type1 platelets, whereas *JAK2* V617F platelets showed only minor, heterogeneous increases restricted to a subset of proteins and samples. In functional assays, *JAK2* V617F platelets showed aggregation responses similar to or lower than those of healthy controls, whereas untreated *CALR* Type2 platelets showed higher CD62P expression, indicating a more activated phenotype than the other ET groups. These findings indicate that ET platelets display mutation-associated proteomic and functional differences and suggest that an altered metabolic state may contribute to platelet reactivity.

## Highlights

• Platelet activation related proteins are more abundant in healthy controls than ET.
• TCA cycle enzymes are downregulated in ET platelets and glycolysis is favored.
• *JAK2* V617F and *CALR* Type1 platelets differ in metabolic signatures upon ASA.
• Surface CD36 is higher in untreated *CALR* Type1 among healthy and ET platelets.
• Surface CD62P is higher in untreated *CALR* Type2 among ET platelets.

## Graphical Abstract

**Figure:**
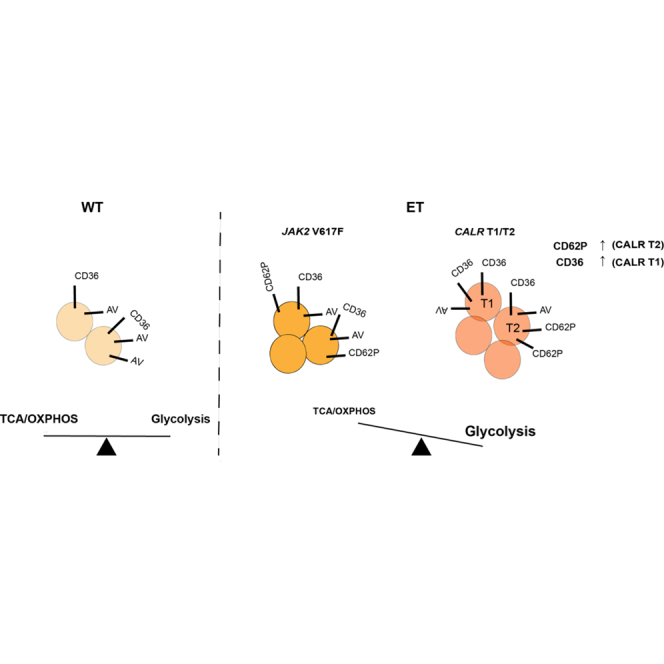
TCA/OXPHOS is downregulated in ET platelets thus favoring other metabolic pathways as indicated by related protein abun

## In Brief

ET platelets presented lower stoichiometries for certain mitochondrial proteins involved in the TCA/OXPHOS cycle as compared to healthy controls. Still, glycolysis status seems to be favored in ET platelets. Specifically, upon ASA-treatment the abundance of metabolic proteins was decreased in *CALR* Type1 when compared to *JAK2* V617F platelets and overall remained at lower levels than in healthy controls. Importantly, mitochondrial mass remained relatively the same between healthy controls and ET platelets. MS and functional data together suggest that different levels of reactivity exist between *JAK2* V617F and *CALR*-mutated platelets, and they are linked to their metabolic signatures.

Patients suffering from essential thrombocythemia (ET), a clonal hematopoietic disease (1), are at variable risk of hemorrhagic or thrombotic complications. Their treatment is based on risk assessment and ranges from observation alone, to anti-platelet agents alone (*i.e.,* acetylsalicylic acid (ASA)), to a combination with cytoreductive agents such as hydroxycarbamide (HU) or anagrelide (ANG) (2).

*JAK2* V617F positive ET has a higher risk of thrombotic events than ET with calreticulin (CALR) mutations (3), which instead carries a higher risk of evolution to myelofibrosis (4). At the same time, the bleeding tendency of ET patients might be higher than previously thought, and could be increased by antiplatelet agents in both high risk (5) and low-risk patients (6).

Platelets are key players in the tissue homeostasis, and an elevated platelet count is the cardinal finding in ET. Still, our understanding of platelet activation in ET remains incomplete, especially its modulation by driver mutations and current treatment regimens. Human platelets retain their protein synthesis capacity and have a diverse transcriptome (7). They also retain a functional spliceosome that can process pre-mRNAs to mature transcripts so that the platelet transcriptome can change dynamically without new transcription. Additionally, platelet translation is controlled by the mTOR pathway (8) that is a main regulator of cell cycle progression and cell growth, linking the activation of platelets with the demand for energy production and metabolism. Interestingly, previous reports have linked metabolism to thrombosis (9, 10).

In this study, we performed MS and flow cytometry-based functional assays on platelets from ET patients with different driver mutations. ET platelets showed proteomic differences from healthy controls in pathways related to platelet activation and aggregation. In addition, *JAK2* V617F and *CALR*-mutated platelets differed in the abundance of several metabolic and mitochondrial proteins, particularly in the context of ASA treatment, suggesting mutation-associated metabolic remodeling. Overall, our results support a link between metabolism and platelet activation in ET and point to mitochondria as an important area for future investigation.

## Experimental Procedures

### Human Samples

Peripheral blood was collected from patients and healthy donors after informed consent according to the guidelines of the Declaration of Helsinki and the Ethical Committee of the Hospital Clínico San Carlos (Madrid, C.P. - C.I. 16/257-E_BS) and the University Hospital Leuven (study number S53745). The diagnosis of ET was made according to the WHO 2016 criteria (4, 11). A total of 76 ET patients and 36 healthy controls were included. Most of the patients included in this study were treated with low dose (80–100 mg) ASA once-daily: therefore, low dose ASA was also given for 3 days to healthy controls prior to blood sampling. Samples were processed within the same day after venipuncture and kept at RT in rotation at all times. Pellets of platelets were snap frozen in liquid nitrogen immediately after platelet rich plasma separation and automated hematogram and stored at −80 °C for MS.

### MS

LC-MS/MS on platelets was performed and analyzed as described (12, 13). Two independent MS rounds have been performed.

### Experimental Design

3 technical replicates from 42 ET and 11 healthy control (CTRL) biological samples in total were processed in 2 rounds of MS. More details can be found in the legends of the MS data sets that follow. 35 ET with *JAK2* V617F, *CALR* Type1, *CALR* Type2, *MPL* W515K/L, triple-negative (TN) that is without any of these driver mutations, and 8 healthy controls were processed in Round#1 MS (Supplementary Table_DDA). 10 ET samples were untreated and 33 samples were treated with ASA, HU, ANG or a combination of HU and ASA or ANG. ASA is the most common treatment among patients (n = 12) and therefore analysis is focused on ASA for treated samples of *JAK2* V617F and *CALR*-mutated patients.

In Round #2, 20 biological samples from 17 unique individuals were analyzed in three technical replicates by data-independent acquisition MS (Supplementary Table_DIA). These included 14 ET samples carrying *JAK2* V617F, *CALR* Type1, or *CALR* Type2 mutations, as well as paired samples from three healthy controls collected before and after 3 days of ASA administration. Nine of the 14 ET samples were obtained from ASA-treated patients.

#### Round #1

Platelet samples were thawed on ice and 10 volumes of lysis buffer 100 mM triethylammonium bicarbonate (TEAB, Sigma), 1% SDS (Genomic Solutions) was added. Immediately, samples were boiled at 95 °C and 1000 rpm for 5 min. After cooling to RT samples were sonicated in a Bioruptor (Diagenode) for 10 min at 30 s on/off cycles. An aliquot was removed for BCA protein determination (Pierce kit, Thermo Fisher Scientific). 500 μg of protein (or less if unavailable) was supplemented with lysis buffer to a final volume of 270 μl. Proteins were reduced in 5 mM dithiothreitol (Sigma) for 1 h at 50 °C and 1000 rpm followed by alkylation in 10 mM 2-chloro-acetamide (Fluka) for 45 min at RT and 1000 rpm. Proteins were precipitated after addition of 1/20 volume of 1M NaCl (Fluka) with four volumes of acetone (BioSolve) (14). Pellets were dissolved in 150 μl 100 mM TEAB, 0.5% sodium deoxycholate (Sigma-Aldrich) by sonication in a Bioruptor for 10 min at 30 s on/off cycles. Proteins were digested with 1:100 Lys-C (Wako) at 37 °C and 1200 rpm for 1 h followed by addition of 1:40 Trypsin (TPCK-treated, Thermo Fisher Scientific) and incubation at 37 °C and 1200 rpm overnight. Trifluoroacetic acid (Thermo Fisher Scientific) was added to a final concentration of 0.5% and sodium deoxycholate was pelleted by centrifugation at 13.000 rpm for 10 min. Supernatants were transferred to a clean tube and 20 μg proteins were desalted by C18 STAGE tip purification (modified from (15)). Final 50% acetonitrile (BioSolve) eluates were dried in a SpeedVac (Thermo Fisher Scientific) and dissolved in 100 μl 2% acetonitrile, 0.5% formic acid (BioSolve). 800 ng proteins was analyzed on an Orbitrap Fusion Lumos Tribrid Mass Spectrometer and an EASY-nLC 1200 System (both Thermo Fisher Scientific) basically as described before (12). DDA raw files were analyzed using MaxQuant version 1.6.17.0. The database search was performed against a human proteome FASTA released in February 2021 containing 97,795 protein entries, together with an additional custom CALR human mutant FASTA. Trypsin digestion permitting up to 2 missed cleavages. Precursor-ion mass tolerance settings 20 ppm for the first search and 4.5 ppm for the main search were used as standard MaxQuant settings. Fragment-ion mass tolerances were 20 ppm for FTMS, and 0.5 Da for ITMS fragment scans. Acceptance criteria were 1% peptide/PSM-level FDR and 1% protein-level FDR. LFQ and iBAQ data were calculated.

#### Round #2

Samples were lysed with 4% SDS, 0.1 M DTT, 0.1 M Tris pH 7.4, heated for 3 min 99 °C and sonicated in water-bath for 15 min and followed by centrifugation for 15 min at 13,000 rpm.

##### Bottom-up proteomic sample preparation using the Sp3 mediated protein digestion protocol

The samples were processed according to the sensitive Sp3 protocol (16). The cysteine residues were reduced in 100 mM DTT and alkylated in 200 mM iodoacetamide (Acros Organics). 20 μg of beads (1:1 mixture of hydrophilic and hydrophobic SeraMag carboxylate-modified beads, GE Life Sciences) were added to each sample in 50% ethanol. Protein clean-up was performed on a magnetic rack. The beads were washed two times with 80% ethanol and once with 100% acetonitrile (Fisher Chemical). The captured on beads proteins were digested overnight at 37 °C under vigorous shaking (1200 rpm, Eppendorf Thermomixer) with 0.5 μg Trypsin/LysC (MS grade, Promega) prepared in 25 mM ammonium bicarbonate. Next day, the supernatants were collected and the peptides were purified using a modified Sp3 clean up protocol and finally solubilized in the mobile phase A (0.1% Formic acid in water), sonicated and the peptide concentration was determined through absorbance at 280 nm measurement using a nanodrop instrument.

### LC-MS/MS Analysis

Samples were run on a LC-MS/MS setup consisting of a Dionex Ultimate 3000 nano RSLC online with a Thermo Q Exactive HF-X Orbitrap mass spectrometer. Peptidic samples were directly injected and separated on a 25 cm-long analytical C18 column (PepSep, 1.9 μm3 beads, 75 μm ID) using an 1-h long run, starting with a gradient of 7% Buffer B (0.1% Formic acid in 80% Acetonitrile) to 35% for 40 min and followed by an increase to 45% in 5 min and a second increase to 99% in 0.5 min and then kept constant for equilibration for 14.5 min. A full MS was acquired in profile mode using a Q Exactive HF-X Hybrid Quadrupole-Orbitrap mass spectrometer, operating in the scan range of 375 to 1400 m/z using 120K resolving power with an AGC of 3 × 10ˆ^6^ and max IT of 60 ms followed by data independent analysis using 8 Th windows (39 loop counts) with 15K resolving power with an AGC of 3 x 10ˆ^5^ and max IT of 22 ms and a normalized collision energy of 26.

### Data Analysis

Orbitrap raw data was analyzed in DIA-NN 1.8 (Data-Independent Acquisition by Neural Networks) (17) through searching against the reviewed Human Uniprot database (including 50518 proteins, retrieved 4/21) in the library free mode of the software, allowing up to two tryptic missed cleavages. A spectral library was created from the DIA runs and used to reanalyze them. DIA-NN default settings have been used with oxidation of methionine residues and acetylation of the protein N-termini set as variable modifications and carbamidomethylation of cysteine residues as fixed modification. N-terminal methionine excision was also enabled. The match between runs feature was used for all analyses and the output (precursor) was filtered at 0.01 FDR and finally the protein inference was performed on the level of genes using only proteotypic peptides. The generated results were processed statistically and visualized in the Perseus software (18) (1.6.15.0) (https://maxquant.net/perseus/).

The raw files and processed proteomics data of the two rounds of MS have been deposited to the ProteomeXchange Consortium via the PRIDE (19) partner repository.

### Western Blotting

Platelets were lysed in RIPA buffer (Sigma-Aldrich) supplemented with protease inhibitors (complete, Roche) and run on gradient 4 to 20% mini-Protean TGX Precast gels (Biorad). Proteins were transferred to PVDF membranes. After blocking with 3% milk/0,05%TBS for 1h at RT, the membranes were incubated with a primary antibody in 1%milk/0,05%TBST for 1,5 h at RT or overnight at 4 °C, followed by a 1h incubation at RT with a secondary antibody (HRP or dye Light 600/680 conjugated). Membranes were developed with ECL (Biorad) after three washes of 10 min each at RT with 0,05%TBST or directly scanned for fluorescence with Imager Azure 600 (Azure Biosystems). The antibody list is available in the supplementary data.

### Platelet Phenotyping and Functional Analysis

Blood was collected in EDTA tubes (BD bio) as described (20). Complete blood counts were measured on Beckman Coulter hemato-counters. Platelet rich plasma separation and flow cytometry based platelet aggregation and activation assay were performed as previously described (20). In addition, TRAP-6 (Thrombin receptor activator, BACHEM) was used at 100 μM final concentration. For the staining of platelet surface markers, 10^6^ platelets were incubated with antibodies in PBS/1% BSA at RT for 15 min except for CD62P, which was prepared in Hepes (+CaCl_2_ 2 mM final) and fixed in 1% formaldehyde prior to acquisition. The list of antibodies used is available in the supplementary section.

Flow cytometry was performed on LSR Fortessa-HTS or FACSCanto-II (BD Biosciences) cytometers, and data were analyzed using FlowJo v10.8 Software (BD Life Sciences; https://www.flowjo.com).

### Statistical Analysis

Visualization of the flow cytometry data and statistics were performed with the R-package or GraphPad (two-way ANOVA).

## Results

### Sample Size of the Study

**Fig. 1:**
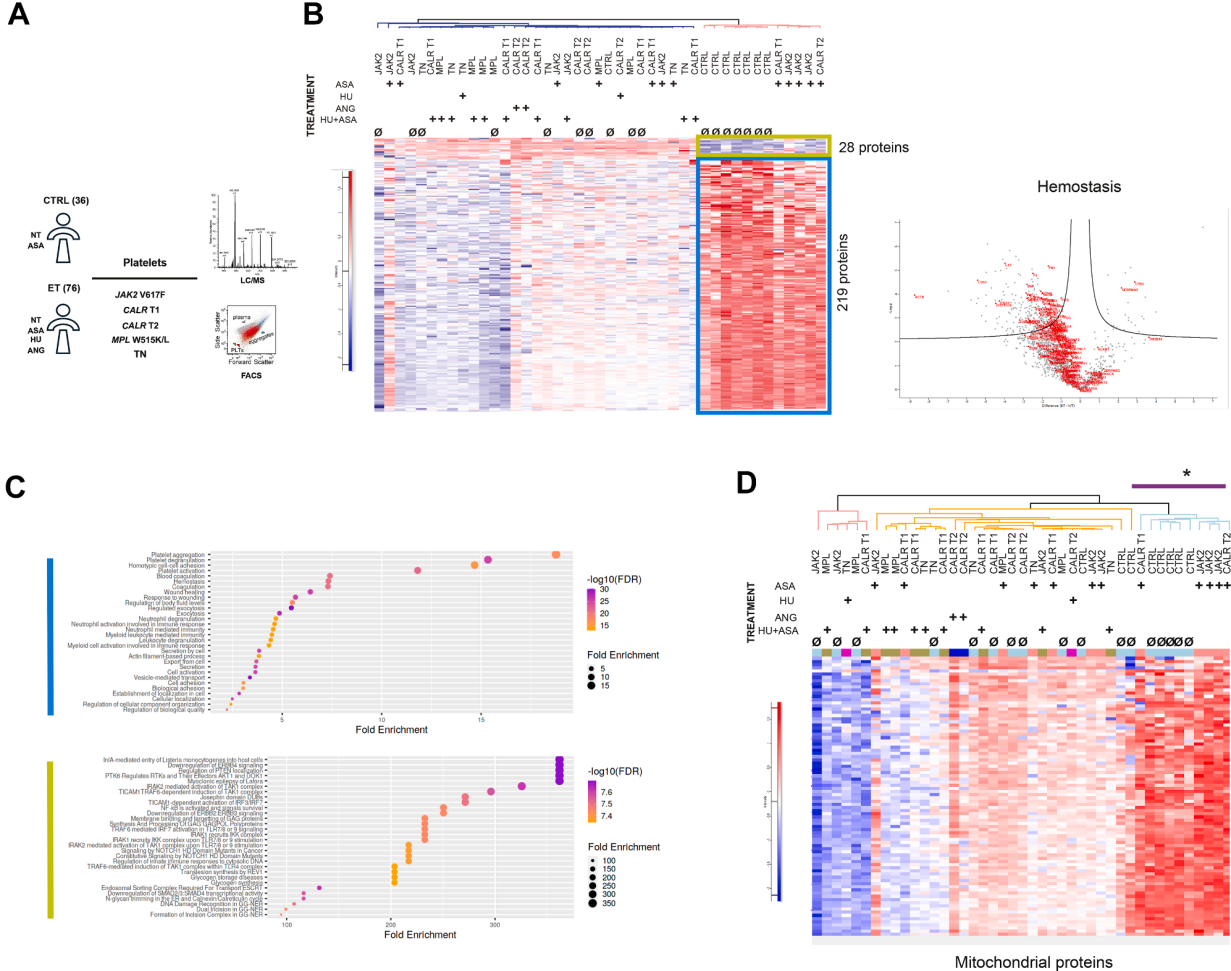
**Round#1 MS.A.** Overview of the study **B*.*** Unsupervised clustering of ET and normal CTRL platelet samples after LC-MS/MS and volcano plot generated by filtering the proteome for hemostasis related proteins only (FDR 0.05) **C***.* Pathway enrichment analysis of proteins with relative increased abundance (*blue box*) or relative decreased abundance (*light green box*) in CTRL samples shown in the heatmap (**B**) **D***.* Unsupervised clustering of the same samples as in (*A*) filtered for mitochondrial proteins only. Analysis has been performed with Perseus software. CTRL, control; ET, essential thrombocythemia.

76 ET patients were recruited, 20 with the *JAK2* V617F mutation, 8 with an *MPL* W515K/L mutation, 17 with a *CALR* Type1 mutation, 9 with a *CALR* Type2 mutation, and 22 TN ET. As controls, 36 healthy donors were recruited (Table 1& Fig. 1*A*). Hematological parameters are shown in Supplementary Fig. S1.

**Table 1:** Clinical and hematological parameters of Essential Thrombocythemia patients per mutational group and normal controls enrolled in the study

	*Nb (Male/Female)*	PLTs	Mean platelet volume (MPV)	Treatment *(Nb)*					
		Mean (range, 10ˆ3/ul)	Mean (range, fl)	ASA	HU	ASA + HU	ANG	HU +ANG	NT
**ET**									
JAK2 V617F	*20*	628,5 (195–1135)	8,9 (7,2–11,5)	*15*		*2*			*3*
CALR Type1	*17*	666,8 (355–1198)	9,2 (7,8–10,6)	*7*	*2*	*6*			*2*
CALR Type2	*9*	631,4 (252–1168)	8,97 (6,5–11,2)	*1*	*2*		*3*		*3*
Triple-negative (TN)	*22 (5*^a^*)*	557,32 (145–1201)	10,42 (7,2–12,70)	*5 (2*^a^*)*	*2 (1*^a^*)*	*3*	*4 (1*^a^*)*	*1*	*7 (1*^a^*)*
MPL W515K/L	*8*	687,33 (306–1183)	8,06 (7,1–10,1)	*4*		*3*			*1*
Total	*76 (27/49)*			*32*	*6*	*14*	*7*	*1*	*16*
**Control**		231,82 (154–429)	9,16 (7,2–12,1)	*12*					*24*
Total	*36 (18/18)*								

ASA, acetylsalicylic acid; HU, hydroxycarbamide; ANG, anagrelide; NT, no treatment.

Italic indicates number of patients or healthy controls.

a Number of *MPL* S204F/P patients included in the TN group.

### Round #1 Mass Spectrometry on ET Platelets and Healthy Controls

LC-MS/MS was performed on platelets from patients with ET and healthy donors, in order to get insights into their function and activation status. 43 samples were processed: *JAK2* V617F: n = 9, *CALR* Type1: n = 8; *CALR* Type2: n = 6, MPL W515K/L: n = 6, TN: n = 6, and controls: n = 8 (Fig. 1*A*). 60 out of the 76 ET and 12 out of the 36 controls were treated with ASA, HU, ANG or a combination of them (Table 1& Supplementary Fig. S1). The rest were untreated ET samples or ASA-free controls respectively and are named as such throughout the text.

In all MS experiments we have not been able to detect any peptides corresponding specifically to the mutant CALR using trypsin as digestion enzyme (Supplementary Fig. S2). We assume that mutant CALR is at very low levels in platelets, as the mutant CALR (del52bp or Ins5bp) is known to be very unstable in over-expressing conditions and it is rapidly degraded (21).

Unsupervised hierarchical clustering of the MS data segregated the protein signatures of ET platelets from that of healthy donors (Fig. 1*B*). Healthy controls clustered together (right side of the heatmap) next to ASA-treated *JAK2* V617F samples. 219 proteins were significantly less abundant in ET platelets (FDR <0.05): with functions in hemostasis, platelet activation, aggregation and degranulation *(e.g.,* CD63, GP1BA, GP1BB, von Willebrand factor (VWF), CLEC1B) shown in the volcano plot (Fig. 1*B*), in cellular stress response (*e.g.,* HSPA1A/B, COX3, BNIP2, PACS-2), in signaling by Rho GTPases and metabolic processes (*e.g.,* ENO2, IDH2, IDH3A, COX2/3) based on gene ontology term/KEGG and reactome gene sets (Fig. 1*C*). 28 proteins (light green box) were more abundant in ET, and involved in MAP kinase activation, TGF receptor signaling, and down-regulation of EGFR/ERBB signaling (RPS27A, UBA52, UBB, UBC) (Fig. 1*C*). Comparison of platelets from untreated ET and ASA-free controls only identified a set of proteins related to platelet activation and aggregation (100 proteins) with lower abundance in ET indicating that the proteome differences between healthy controls and ET samples as seen in the mixed samples (Fig. 1*B*) were independent of treatment (Supplementary Fig. S3).

Among the 219 proteins with lower abundance in ET (Fig. 1*B*, heatmap) many are involved in mitochondrial metabolism. A similar clustering pattern emerges when analysis is restricted to mitochondrial proteins only (Fig. 1*D*). Specifically, healthy controls show higher abundance for approximately 88 proteins including aconitase 2 (ACO2), citrate synthase (CS), PKM, IDH1, IDH2 as compared to the rest of ET samples, with the exception of 3 *JAK2* V617F and 2 CALR-mutated ASA-treated ET samples (Fig. 1*D*, asterisk).

In summary, the platelet proteome in ET is distinct from healthy controls with enrichment of proteins related to kinase activation indicative of an underlying inflammatory signature described previously in MPN (22, 23). Many of the protein signatures of ASA-treated *JAK2* V617F ET were similar to healthy controls and related to platelet activation and mitochondrial function.

### Analysis of ASA-Treated ET Patients

ASA was the most common treatment type among the analyzed samples, specifically in *JAK2* V617F (Table 1). ASA blocks the cyclooxygenase 1 and thromboxane synthesis (24). Therefore, we analyzed the proteome of the ASA-treated ET and healthy CTRL samples only.

Proteome comparison of the ASA-treated ET versus untreated ET and ASA-free CTRL samples unveiled a number of proteins significantly differentiating the two conditions in *JAK2* V617F and *CALR*-mutated ET (Supplementary Fig. S4, *A* and *B*). In *JAK2* V617F samples, more proteins were significantly increased in the ASA-treated condition. This comparison included 2 non-treated (NT) and 6 ASA-treated *JAK2* V617F platelet samples, and reflected the higher average abundance of 763 of a total 783 detected proteins in the ASA-treated group. However, the magnitude of enrichment varied across samples, as two distinct groups of ASA-treated *JAK2* V617F samples were observed in the heatmap of Supplementary Fig. S4. Among them there are proteins involved in signaling (*i.e.,* PPAR, MAPK and JAK-STAT) and mitochondrial metabolism (PDK1, ACO2, IDH2, CS, succinate dehydrogenase A, CYC1, FH, DECR1, COX5B, GSR etc). Also, proteins related to platelet activation, aggregation and degranulation were enriched in the ASA-treated samples (CD36, CD9, GP1BA, GP1BB, PF4, GP5, GP6, GP9, VWF, SELP, MMRN1) as compared to the NT condition (Supplementary Fig. S4*A*).

Similarly, 4 *CALR-*mutated ASA-treated samples clustered separately from the 3 NT *CALR-*mutated samples (Supplementary Fig. S4, *A* and *B*). However, the 2 out of 3 *CALR* Type1 ASA-treated platelets showed a lower abundance for 52 proteins, the majority of which are metabolic enzymes or mitochondrial proteins (*e.g.,* TARDBP, COX5B, COX6A1, AGK), and proteins directly related to platelet activation did not significantly change in contrast to what we observed for *JAK2* V617F ET. However, given the low numbers of *CALR*-mutated samples including 2 different CALR mutations it is difficult to draw conclusions separately regarding *CALR* Type2 platelets.

Taken together, these data suggest that ASA treatment has an impact on mitochondrial metabolism of ET platelets. Still, there was heterogeneity between samples both for *JAK2* V617F platelets and *CALR*-mutated platelets suggesting that the link between metabolic activity and platelet activation might be dependent on the allele burden of the driver mutation, specifically for *JAK2* V617F.

### Round#2 MS on ASA-Treated ET and Healthy Controls Platelets Only

**Fig. 2:**
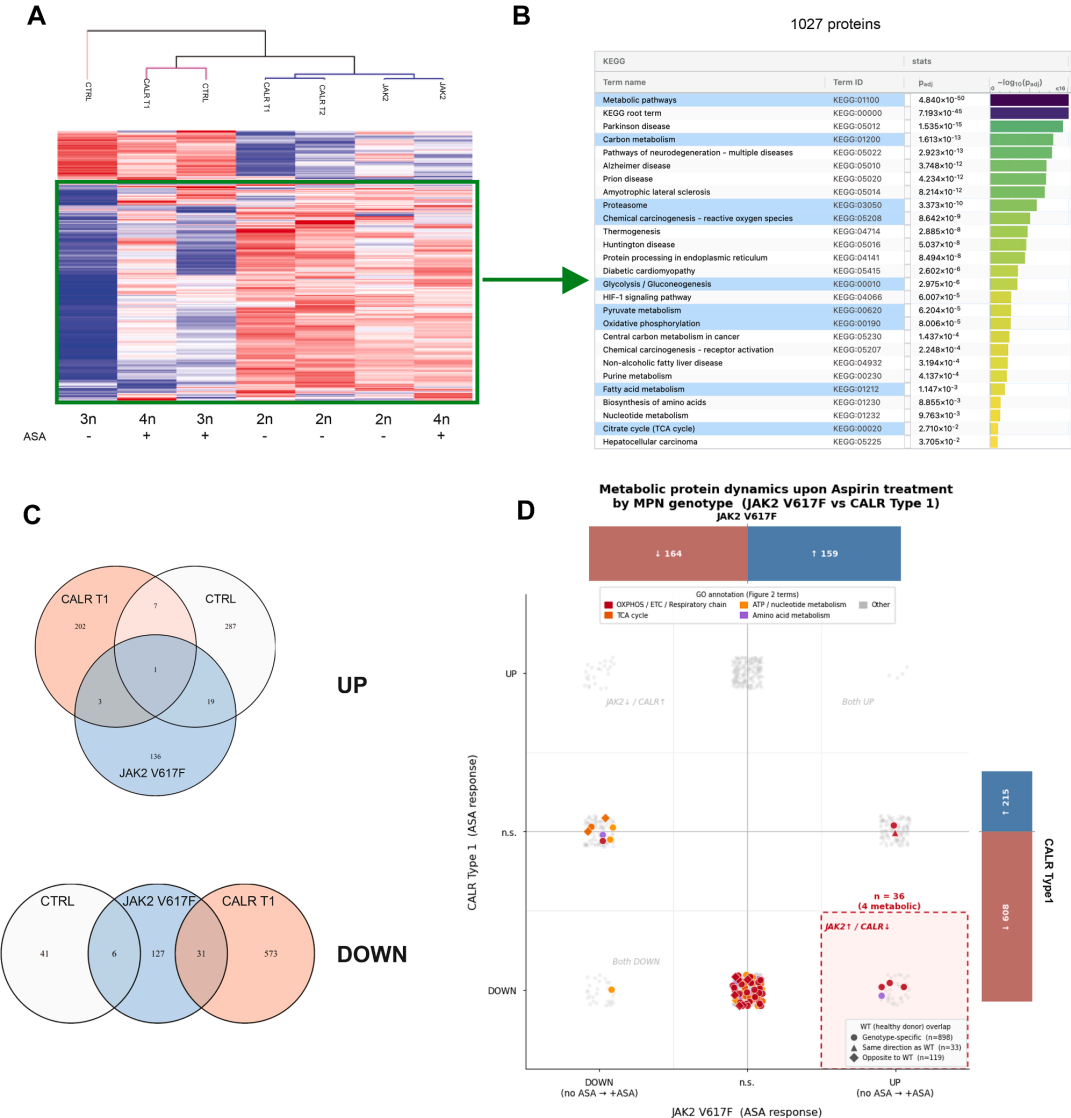
**Round#2 MS.A***.* Unsupervised clustering of ET and CTRL platelet samples by MS (±ASA). Mean abundance values (triplicates per sample) have been generated for all proteins per mutational group (*JAK2* V617F, *CALR* Type1 and *CALR* Type2) and CTRL. Number of patient/donor samples (n) per group is shown **B**. Gene ontology pathway (g:profiler) analysis on metabolic pathways from the 1027 enriched proteins (*green box in A*) **C*****.*** Venn diagrams shows the number of upregulated (*up*) or downregulated (*down*) proteins upon ASA treatment for each ET group and healthy controls (*CTRL*) **D***.* Each protein was assigned a binary directional score (+1 *up*, −1 *down*) per genotype and plotted on a two-dimensional scatter to visualize concordant and divergent ASA responses between *JAK2* V617F (x-axis) and *CALR* Type 1 (y-axis). Proteins were annotated to metabolic pathways based on gene ontology terms identified as significantly enriched in Figure 2**B**, covering oxidative phosphorylation and electron transport chain , TCA cycle, ATP and nucleotide metabolism, and amino acid metabolism. The relationship to WT ASA response was encoded by marker shape: genotype-specific changes not observed in healthy donors (*circle*), changes shared with WT in the same direction (*triangle*), and changes opposite to the WT response (*diamond*). ASA: acetylsalicylic acid; CTRL, control; ET, essential thrombocythemia; TCA, tricarboxylic acid cycle.

Given the impact of the results of Round#1 MS upon ASA treatment we set out to perform another MS round (LC-MS/MS) focusing specifically on the effect of ASA on mitochondrial metabolism in *JAK2* V617F and *CALR* Type1 platelets. ASA-treated *CALR* Type2 patients were rare, thus not included in this new MS experiment. Importantly, new ET patients were recruited with *JAK2* V617F and *CALR* Type1 mutations. Also, three healthy controls were sampled before and after 3 days of low-dose ASA administration once-daily, which is sufficient time to cause COX inhibition in platelets and confer irreversible inhibition in thromboxane synthesis in the circulation (25). A total of 20 samples were processed for LC-MS/MS consisting of healthy controls, *JAK2* V617F, *CALR* Type1 and *CALR* Type2 patients (Fig. 2*A*).

As shown in Figure 2*A* the ET samples clustered separately except from the *CALR* Type1 ASA-treated samples, which were found in between the ASA-free and ASA-treated healthy controls even though with higher abundance scores for the majority of the 1027 proteins (green box). This group of stoichiometrically enriched proteins, which were mostly shared by the ET platelets (shown in the green box of the heatmap at Fig. 2*A*) were strongly related to metabolism, glycolysis, tricarboxylic acid cycle (TCA) and fatty acid metabolism (Fig. 2*B*) apart from platelet activation, degranulation, aggregation and hemostasis that we also observed in Round#1 MS. Comparing ASA treatment versus untreated or ASA-free conditions we found no significant overlap in proteins with significant abundance change between ET mutational groups (*JAK2* V617F, *CALR* Type1 and *CALR* Type2) and healthy controls with only 7 common proteins (1 UP- and 6 DOWN-regulated) as it is shown in venn diagrams (Fig. 2*C*). Gene ontology analysis of the 202 proteins uniquely enriched (Venn-UP) in the *CALR* Type1 ASA-treated samples highlighted immune related processes while the 136 proteins in *JAK2* V617F were rather related to actin polymerization, mitochondrial ATP synthesis and oxidative phosphorylation (Supplementary Fig. S5*A*). Interestingly, none of these pathways were found to be related to the 314 enriched proteins deregulated in ASA-treated healthy controls. Regarding the proteins with reduced stoichiometry in the ASA condition (Venn-DOWN), 573 were found to be unique in *CALR* Type1 platelets and they were related to mitochondrial and nucleotide metabolism while 127 unique proteins of the *JAK2* V617F platelets were mainly related to immune responses (Fig. 2*C* and Supplementary Fig. S5*B*). Overall, the analysis reveals that ASA broadly suppresses mitochondrial and metabolic proteins in *CALR* Type1 cells, the majority of which are not significantly changed in *JAK2* V617F (bottom-center protein cluster, Fig. 2*D*). A subset of these proteins (n = 36, highlighted quadrant) shows simultaneous upregulation in *JAK2* V617F alongside downregulation in *CALR* Type1, indicating divergent and potentially opposing metabolic responses to ASA treatment between the two ET genotypes. Most of these changes are genotype-specific and not observed in healthy donors, suggesting that the metabolic response to ASA is shaped by the underlying mutation rather than representing a general drug effect.

In summary, these results emphasize the different protein dynamics between *JAK2* V617F and *CALR* Type1 ET regarding the stoichiometries of mitochondrial proteins and the related metabolic pathways upon ASA treatment.

### Western Blotting on Platelet Lysates

**Fig. 3:**
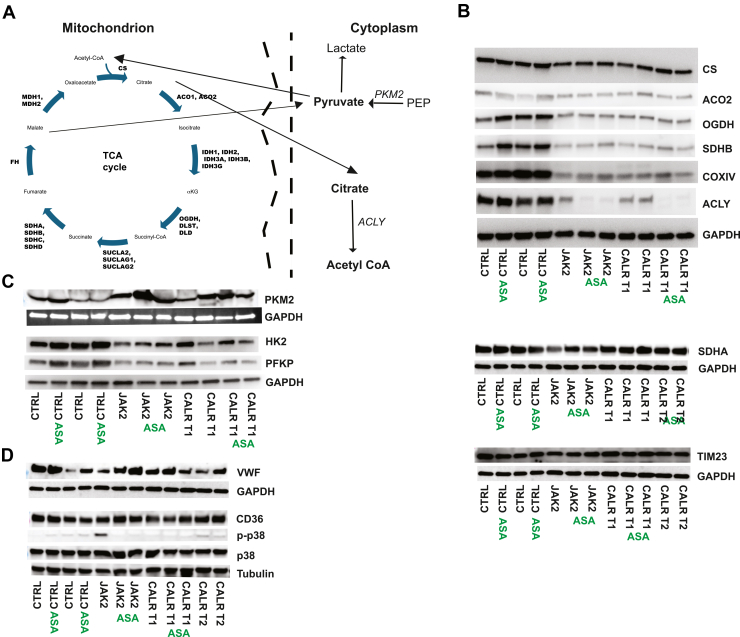
**Western blot validation of metabolic and platelet activation.A.** TCA cycle scheme **B**. Western blot analysis on platelet lysates for the main TCA enzymes including aconitase, citrate synthase, oxoglutarate dehydrogenase complex, succinate dehydrogenase *A* and *B*, cytochrome c oxidase subunit IV of the electron transport chain, the cytosolic ATP-citrate lyase and TIM23 of the inner mitochondrial membrane **C**. the pyruvate kinase PKM2 (marker for aerobic glycolysis), Hexokinase 2 (HK2) and Phosphofructokinase, Platelet type (PFKP) are key enzymes in glycolysis **D***.* other proteins related to platelet reactivity (von Willebrand factor, CD36, p38 and phospho-p38) in ET patients and CTRL (±ASA). GAPDH or tubulin has been used as loading controls. CTRL, control; ET, essential thrombocythemia; TCA, tricarboxylic acid cycle.

The differences in the abundance of mitochondrial proteins upon ASA treatment between ET platelets, accompanied by sample heterogeneity, prompted us to investigate the metabolic pathways linked to energy production and specifically glycolysis and TCA/oxidative phosphorylation (26) (Fig. 3*A*). Protein levels for several enzymes involved in the respective pathways were assessed by western blotting in ASA-treated and untreated or ASA-free conditions.

Several TCA enzymes and the c oxidase subunit IV (electron transport chain) were tested and in general their levels were considerably lower in ET platelets as compared to normal controls (Fig. 3*B*). Some exceptions were found to this trend for example CS and ACO2 or succinate dehydrogenase A that was lower only in *JAK2* V617F platelets. In particular, CS and ACO2 did not show significant changes probably due to their position at the entry point of the TCA cycle they may be less susceptible to fluctuation, as continued influx of cytoplasmic substrates require stable levels of expression. Still, they might adjust to lower activity. By contrast, enzymes downstream in the TCA cycle may be more markedly affected by substrate limitation, leading to stronger alterations in their abundance and/or activity. Importantly, levels of the inner mitochondrial membrane TIM23 were found to be comparable in all ET and healthy CTRL samples independently of treatment with ASA suggesting that mitochondrial mass does not change significantly and therefore the stoichiometry changes observed in TCA enzymes were real.

Pyruvate kinase (PKM2) catalyzes the final rate-limiting step of glycolysis by converting phosphoenolpyruvate to pyruvate while phosphofructokinase (PFKP) and hexokinase 2 (HK2) are prominent regulatory enzymes of glycolysis. We found higher PKM2 levels in ET platelets that increased upon ASA specifically in *JAK2* V617F while PFKP and HK2 levels were somewhat reduced or unchanged in general (Fig. 3*C*). PFKP and HK2 increased markedly upon ASA in healthy controls and slightly increased in *JAK2* V617F while CALR Type1 levels were heterogeneous. Interestingly, the ATP-citrate lyase (ACLY), a cytosolic enzyme that converts ‘mitochondria-derived’ citrate into acetyl CoA, was reduced drastically in ET (Fig. 3*B*) better consistent with a more glycolytic state as described previously in tumor cells (27). Of note, ACLY was downregulated upon ASA opposite to healthy CTRL platelets suggesting that cytosolic citrate derived from mitochondria is either limited or not used in ET (28).

We also checked the levels of other proteins unrelated to mitochondria including VWF, p38 MAPK and CD36, all involved in platelet activation (29, 30) (Fig. 3*D*). Levels of the endogenous VWF consistently increased upon ASA in ET and healthy CTRL samples meaning that protein reserves (α-granules) are contained and therefore not translocated to the plasma membrane as it happens upon platelet activation (31). p38 MAPK levels remained stable, although phosphorylation of p38 MAPK was higher in ASA-free *JAK2* V617F platelets and undetectable in ASA-treated *JAK2* V617F. Cytoplasmic CD36 was higher in *CALR* Type2 among CALR mutated samples and increased in the ASA-treated *JAK2* V617F platelets unlike *CALR* Type1 ASA-treated samples.

Taken together, these results suggest that ET platelets undergo an overall metabolic shift toward a more glycolytic state. ASA treatment increased TCA enzyme levels, with a clear effect in healthy controls but only a modest increase in JAK2 V617F platelets, in which levels remained markedly lower than those observed in controls.

### Platelet Activation

**Fig. 4:**
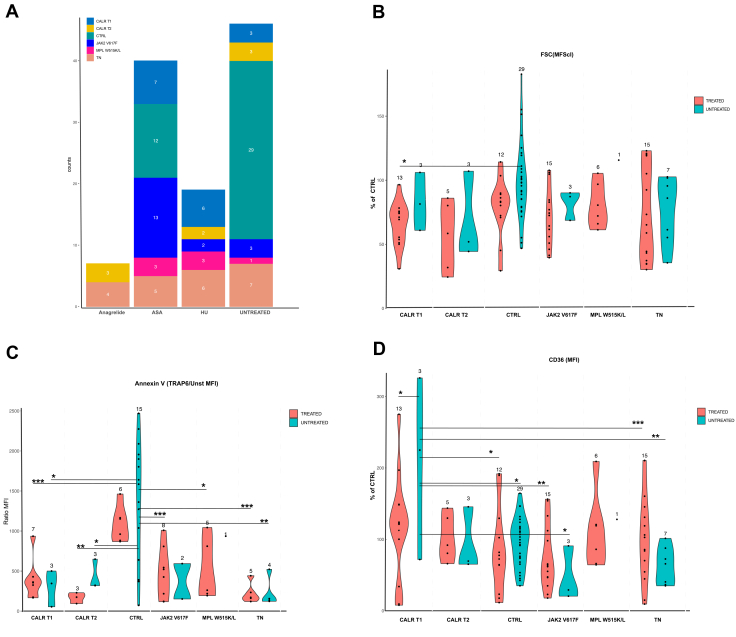
**Surface-marker and activation profiling of ET and control platelets by flow cytometry.A.** Number of samples per mutational group and treatment of ET and CTRL included in the analysis of surface marker relative expression by flow cytometry **B***.* Mean forward scatter intensity obtained by flow cytometry analysis. Untreated CTRL mean forward scatter has been set to 100 and values of all samples have been represented as a percentage of the CTRL mean MFI **C***.* Ratio of MFI of Annexin-V upon TRAP6 stimulation and unstimulated condition. **D***.* CD36 levels with CTRL mean MFI set to 100 as in *(B)*. Treated samples (*red* violin graphs) include all different treatment regimens alone or in combination (ASA, HU, ANG) except from CTRL and *JAK2* V617F in which all treated samples are only ASA-treated. Nontreated samples (including ASA-free) are shown in *light blue violin plots*. Numbers of samples per group are indicated on top of every *violin plot*. Differences are shown when statistical significance was reached in equal or more than 3 samples per group analyzed (∗p-val ≤0.05, ∗∗p-val ≤0.01, ∗∗∗p-val ≤0.001), (CTRL: control; ASA: acetylsalicylic acid; HU: hydroxycarbamide; ANG: anagrelide; ET, essential thrombocythemia.

As the proteome analysis uncovered metabolic signatures that differ between *JAK2* V617F and *CALR*-mutated platelets we set out to characterize their activation status in order to see whether there is any correlation with their proteome. To this end, we measured the expression of surface markers related to platelet activation including CD36 (29, 32) without any stimulus and CD62P, CD63 upon agonist stimulation (*i.e.,* TRAP6, phorbol 12-myristate 13-acetate [PMA], collagen) in healthy controls and ET (Fig. 4).

We measured forward scatter as a relative indicator of platelet size and mean forward scatter intensity to accurately assess the changes in expression levels of activation markers per surface area in the plasma membrane. No major differences were found among samples in terms of size either between mutational groups or treatment regimens (Fig 4*B* and Supplementary Fig. S6*A*). Mean platelet volume did not change significantly either (Supplementary Fig. S1). Additionally, Annexin-V binding was assessed as a marker of phosphatidylserine exposure on the platelet membrane, which is associated with procoagulant platelet responses and can occur upon platelet activation (33). Interestingly, we observed lower levels (ratioMFI) of Annexin-V in ET platelets as compared to healthy controls upon TRAP6 stimulation and specifically in the untreated condition (Fig. 4*C*). A similar trend was observed upon PMA or collagen stimulation but it was not statistically significant (Supplementary Fig. S7, *A* and *B*). Overall, this result suggests that ET platelets might be preactivated or hyporesponsive as compared to the healthy controls and therefore they seem to respond less upon agonist stimulation (lower ratio: agonist_MFI/Unstimulated_MFI).

**Fig. 5:**
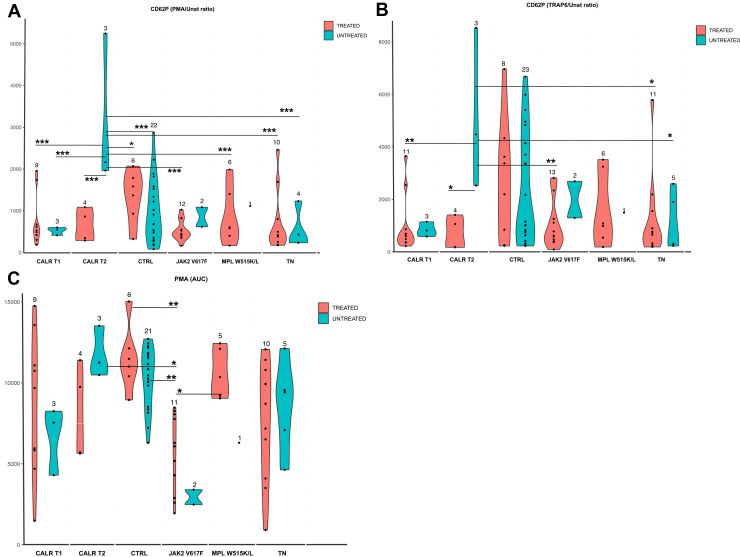
**Flow cytometry analysis of CD62P surface expression and aggregation responses to different agonists in ET platelets.A.** CD62P MFI ratio of relative expression levels upon PMA stimulation and unstimulated condition and **B.** upon TRAP6 stimulation, CD62P MFI (agonist_MFI/unstimulated_MFI) **C.** Aggregation levels (area under the curve, AUC) of ET and CTRL platelets upon PMA stimulation. Treated samples (*red violin plots*) include all different treatment regimens alone or in combination (ASA, HU, ANG) except from CTRL and *JAK2* V617F groups in which all treated samples are only ASA-treated. Nontreated samples (including ASA-free) are shown in *light blue violin plots*. Numbers of samples per group are indicated on top of every *violin plot*. Differences are shown when statistical significance was reached in equal or more than 3 samples per group analyzed (∗p-val ≤0.05, ∗∗p-val ≤0.01, ∗∗∗p-val ≤0.001), CTRL: control; ASA: acetylsalicylic acid; HU: hydroxycarbamide; ANG: anagrelide.

CD36 levels were higher in untreated *CALR* Type1 than normal controls, *JAK2* V617F, TN and treated *CALR* Type1 platelets (Fig. 4*D*). Instead, CD62P expression levels (MFI ratio) were higher in *CALR* Type2 untreated platelets upon PMA and TRAP6 stimulation and these differences were more significant in PMA (Fig. 5, *A* and *B*). Also, CD62P levels decreased significantly to levels similar to other ET samples in the treated condition, meaning that these measurements are not an artifact. With respect to CD63 levels no significant changes were observed among samples (Supplementary Fig. S7, D-F).

We also measured expression of several abundant platelet surface markers (CD49B, CD31, CD61, CD42A, CD42B, CD9, GPVI) not directly related to platelet activation to check for bias on CD36 and CD62P found on the 3 untreated *CALR* Type1 and *CALR* Type2 samples respectively. No significant differences were observed for these markers except for CD49B that was higher in controls in the ASA-treatment condition (Supplementary Fig. S6).

Taken together, these data indicate that ET platelets display surface marker expression profiles largely similar to those of healthy controls, with the notable exceptions of CD36 and CD62P, which were increased in *CALR* Type1 and *CALR* Type2 platelets, respectively. In contrast, *JAK2* V617F platelets showed expression profiles for these markers that were comparable to those of healthy controls. These findings suggest that CALR-mutated and *JAK2* V617F platelets may differ in their basal activation state and therefore in their responsiveness to stress signals.

### Platelet Aggregation

We next sought to characterize the aggregation capacity of ET platelets by flow cytometry at a single receptor level (20). The assay is suitable for the detection and quantification even of small scale aggregates (microaggregates) and allows a dynamic analysis of the aggregation capacity of platelets during time. Six receptors were analyzed (α2β1, αIIbβ3, GPVI, CLEC2, VWFR, and PAR1) on a time course stimulation (15s to 10 min) using specific agonists for each receptor (collagen -α2β1 and GPVI-, PMA -αIIbβ3-, convulxin -GPVI-, Aggretin A -CLEC2-, ristocetin -VWFR-, and TRAP6-PAR1) as described before.

The area under the curve was calculated (% aggregation) on a time course agonist stimulation for each sample. Basal aggregation (% of aggregates at Time 0) and spontaneous (no agonist stimulation) aggregation (% of aggregates at Time10 min) were also calculated but no significant differences were observed (Supplementary Fig. S8, *A* and *B*). Regarding aggregation upon agonist stimulation, ET platelets did not present significant differences in aggregation (area under the curve) upon stimulation with agonists as compared to healthy controls with the exception of TRAP6 and PMA conditions. Interestingly, *JAK2* V617F platelets showed lower aggregation levels than controls (ASA condition) on PMA and TRAP6 stimulation (Fig. 5*C* and Supplementary Fig. S8*C*) and untreated samples followed this trend despite the low numbers (2 ET patients). No differences were found for the other agonists tested (Supplementary Fig. S8, D-G).

Taken together, these data suggest that ET platelets respond similarly to healthy CTRL platelets in their capacity to aggregate in response to different agonists acting through distinct receptors and downstream signaling pathways. Unexpectedly, the aggregation capacity of *JAK2* V617F platelets was comparable to or lower than that of healthy controls, suggesting that they may differ functionally from other ET platelets.

## Discussion

Functional studies on ET platelets have been reported previously (34, 35, 36, 37, 38, 39), many of them focusing on the *JAK2* V617F mutation. Although the number of studies addressing the effects of different treatment strategies, including cytoreductive and antiplatelet agents, is increasing, important conceptual gaps remain regarding treatment efficacy and treatment-associated metabolic changes, particularly in relation to thrombosis and hemorrhage (36, 40, 41, 42, 43, 44, 45). The platelet proteome in ET has not been widely explored despite the fact that such analyses can provide important insight into platelet biology in MPN (41, 46, 47)).

In this study, 42 ET and 11 CTRL platelet samples from treated (ASA, HU, ANG) and untreated conditions were subjected to LC-MS/MS in two separate MS rounds. The inclusion of the untreated ET samples as well as ASA-treated healthy controls highlighted the importance of treatment status when interpreting both proteomic and flow cytometry-based functional data. This was particularly evident in the proteome analysis and Western blot validation of ASA-treated ET patients and healthy controls. Our data suggest that *JAK2* V617F and *CALR*-mutated platelets show different proteomic profiles in the presence of ASA, especially with respect to mitochondrial and metabolic protein stoichiometries, which remained stable or slightly increased in *JAK2* V617F but were mostly reduced in *CALR* Type1 platelets (Fig. 2 and Supplementary Fig. S4).

We also examined TIM23, a stable structural mitochondrial protein and member of the presequence translocase of the inner membrane TIM23 complex (48). No significant changes were observed between CTRL and ET platelets with or without ASA. This contrasts with a recent report showing increased TOM20, a component of the mitochondrial outer membrane TOM complex, in ET platelets (41). Previous work has also suggested that MPN, and especially ET platelets may contain increased numbers of mitochondria (42). However, in ET this likely applies only to a fraction of circulating platelets because of the clonal nature of the disease. In our dataset, the stable abundance of TIM23 does not support major differences in mitochondria mass between ET and healthy CTRL platelets.

We have further assessed the expression of TCA cycle and major glycolytic enzymes. The reduced abundance of several TCA cycle enzymes, together with higher PKM2 and markedly low ACLY levels in ET platelets, is consistent with metabolic remodeling and a shift away from mitochondrial oxidative metabolism (9). At the same time, the glycolytic pattern was not uniform across all enzymes, suggesting that the metabolic changes in ET platelets are selective rather than representing a simple global increase in glycolysis.

In addition, CD36, a fatty acid transporter (29, 49) was found higher on the surface of untreated *CALR* Type1 platelets (Fig. 4*D*). Cytoplasmic CD36 levels showed an inverse pattern relative to surface expression among CALR-mutated (Type1 and Type2) platelets, suggesting altered membrane localization or trafficking. Because surface CD36 has been linked to platelet activation and lipid metabolism in other contexts (49), these findings raise the possibility that CD36-associated lipid handling contributes to platelet phenotype in *CALR*-mutated ET. However, this remains speculative and should be addressed in future functional studies. Within the broader context of chronic inflammation known to characterize MPN, similar observations have been reported for the correction of dysregulated inflammation through modulation of fatty acid homeostasis in IL-10 deficiency (50). Together, these findings support the idea that metabolic pathways may influence platelet reactivity and could represent targets for further investigation.

Finally, *JAK2* V617F platelet activation and aggregation responses were generally within the range of other ET groups (51) but in some agonist-stimulated conditions they were significantly lower than those of healthy controls. This was also reflected in their proteomic profile, which differed from both CALR-mutated platelets and healthy CTRL platelets under ASA treatment (Fig. 2). In addition, we show that *CALR* Type1 and *CALR* Type2 platelets are not functionally identical, as levels of CD62P were significantly higher in untreated *CALR* Type2 patients (Fig. 5, *A* and *B*). Together, these results suggest that *JAK2* V617F and CALR mutations are associated with distinct platelet phenotypes, reflected in both proteomic profiles and functional responses, with metabolic differences representing one prominent feature of this divergence.

A limitation of our study is the low number of samples within each treatment subgroup, together with the heterogeneity of treatment regimens (ASA, HU, ANG, or combinations) and the particularly small number of untreated ET patients. In addition, we do not provide direct metabolic functional measurements, partly because bulk platelet analyses in ET are complicated by disease clonality and the relatively low mutational burden in circulating platelets (52). Nevertheless, our data show that ET platelets display mutation-associated proteomic and functional differences, with particularly strong changes in mitochondrial and TCA-related proteins. These patterns are consistent with altered metabolic state and differential platelet responsiveness across JAK2 and CALR subtypes, but direct metabolic function remains to be tested. Further studies addressing mitochondrial activity and metabolic dependencies at the functional level will be necessary to determine whether these pathways can be exploited therapeutically to modulate platelet reactivity in MPN.

## Data Availability

The mass spectrometry data produced in this study are available in the following databases:

MS Experiment #1: Identifier PXD052171 (https://www.ebi.ac.uk/pride/login)

Username: reviewer_pxd052171@ebi.ac.uk

Password: hcM6RHOg

MS Experiment #2: Identifier PXD050550 (https://www.ebi.ac.uk/pride/login)

Username: reviewer_pxd050550@ebi.ac.uk

Password: rkWkUT9R

## Supplemental data

This article contains supplemental data.

## Conflict of Interest

The authors declare no competing interests.

## Supplemental data

Supplementary Table DIA

Supplementary Table DDA
