# Supplementary material for: Platelet Proteome Links Metabolism to Reactivity in Essential Thrombocythemia
Source: Mol Cell Proteomics. 2026 Jun 29;25(8):101617. doi: 10.1016/j.mcpro.2026.101617 (PMC13450191; doi:10.1016/j.mcpro.2026.101617)
Supplement: Supplementary file 1 [file mmc4.pdf]

Supplemental Figure. Visualization of target peptides using the integrated DIA-NN viewer. Extracted-ion chromatogram (XIC) for the single proteotypic peptide identifying the indicated protein (gene name above each panel). Panel headers give the peptide sequence, precursor charge, and posterior error probability (PEP) (e.g. FLPSYQAVEYMR, 2+, PEP = 4.4×10^{-3}); the amino-acid ladder marks b-/y-ion coverage (colored dots). Co-elution of precursor (MS1, **[color]**) and fragment ions (MS2, multiple colors); the inset legend lists each transition (b/y ion, charge state) and its maximum intensity. X-axis, retention time (min); y-axis, intensity; vertical dashed lines indicate the automated peak boundaries assigned by DIA-NN.

AGA

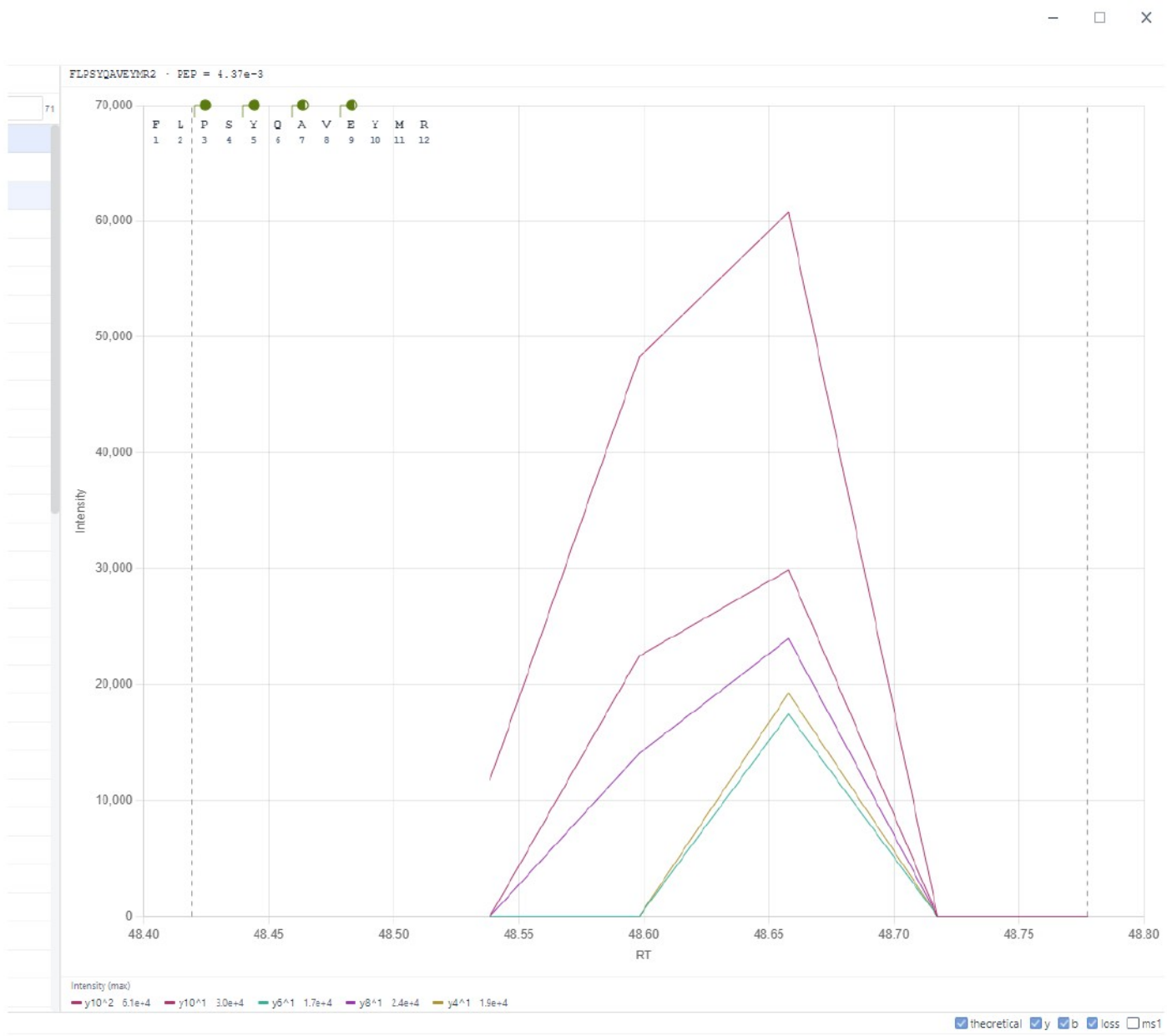

ANXA1

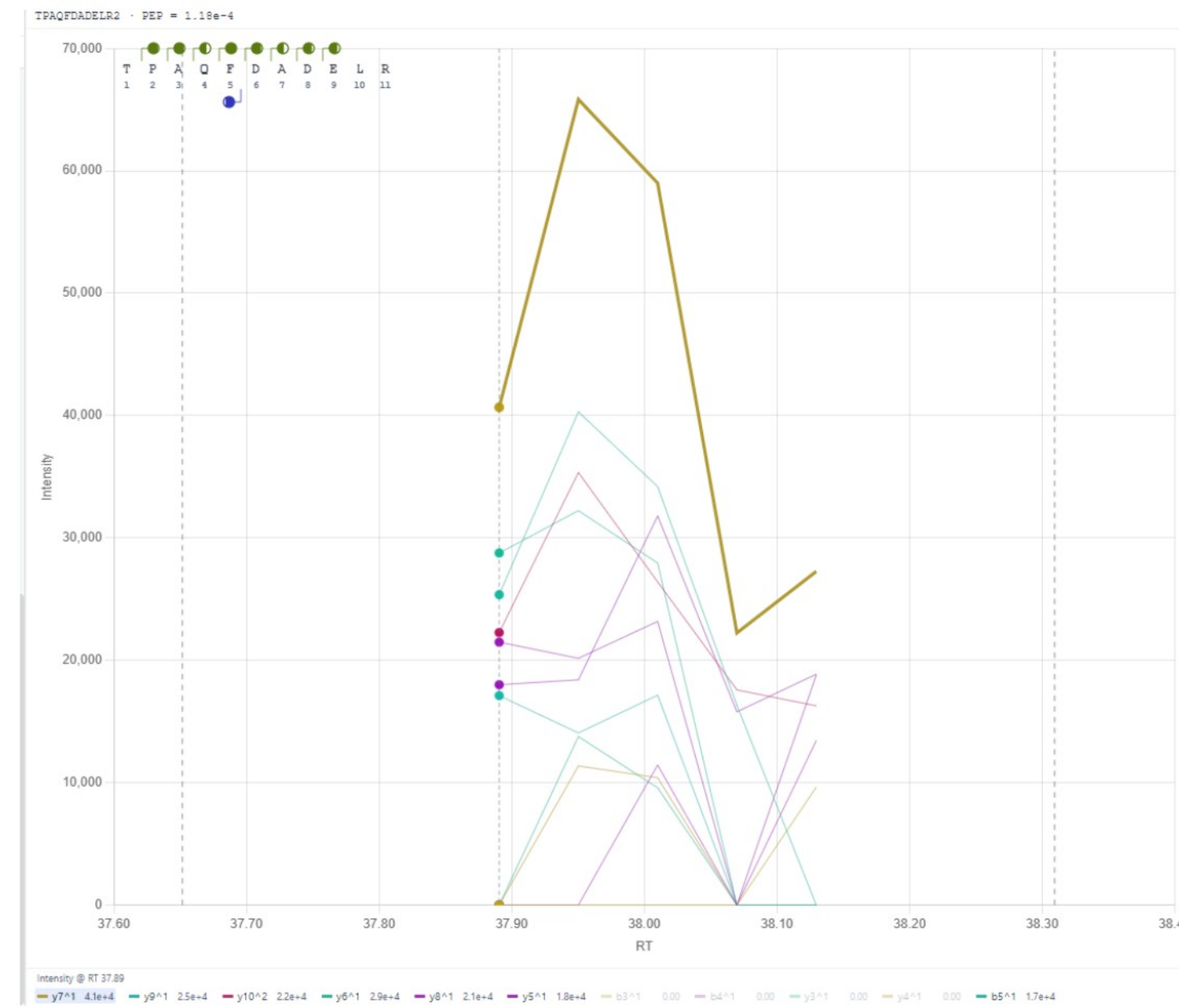

AP1S1

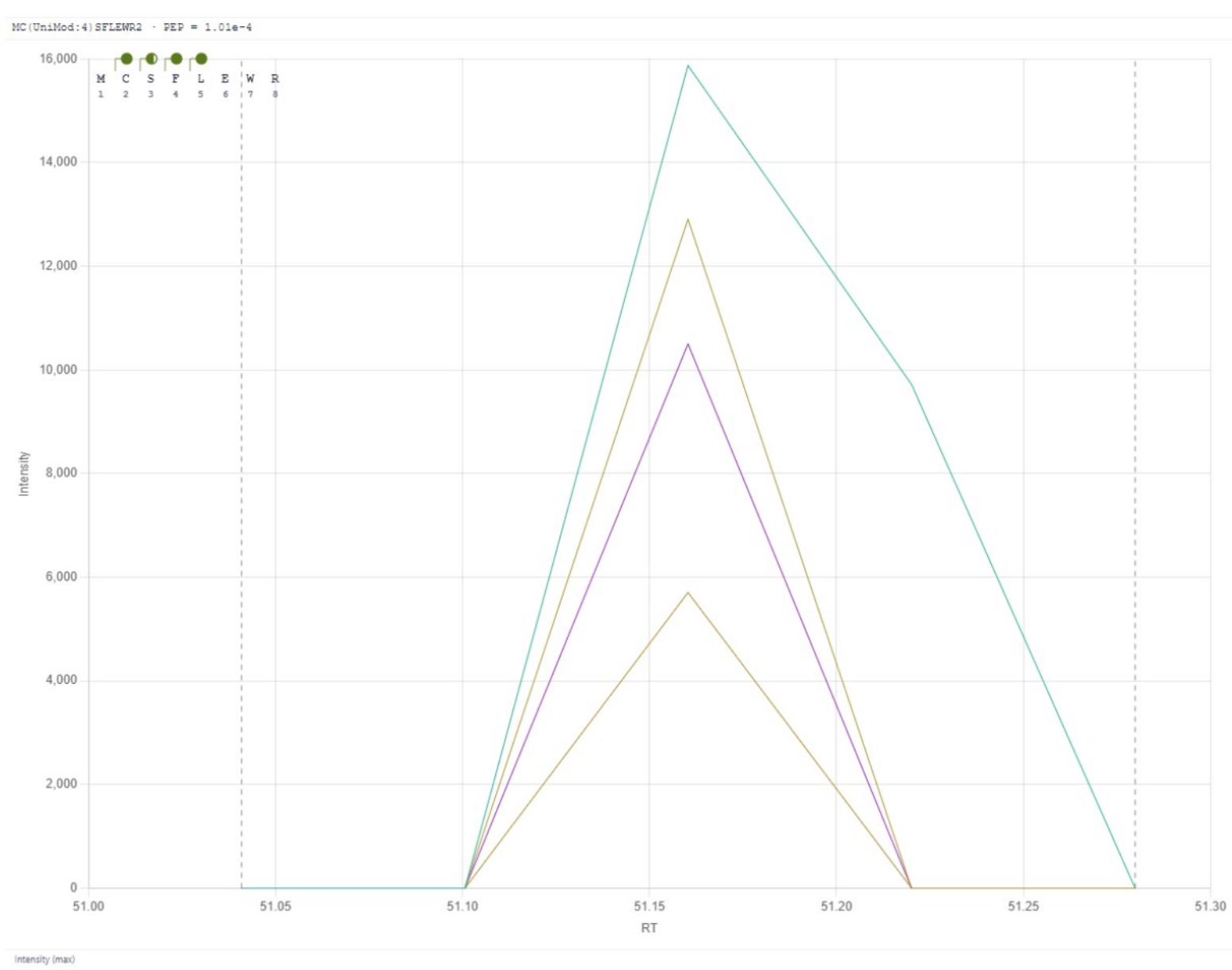

APAF1

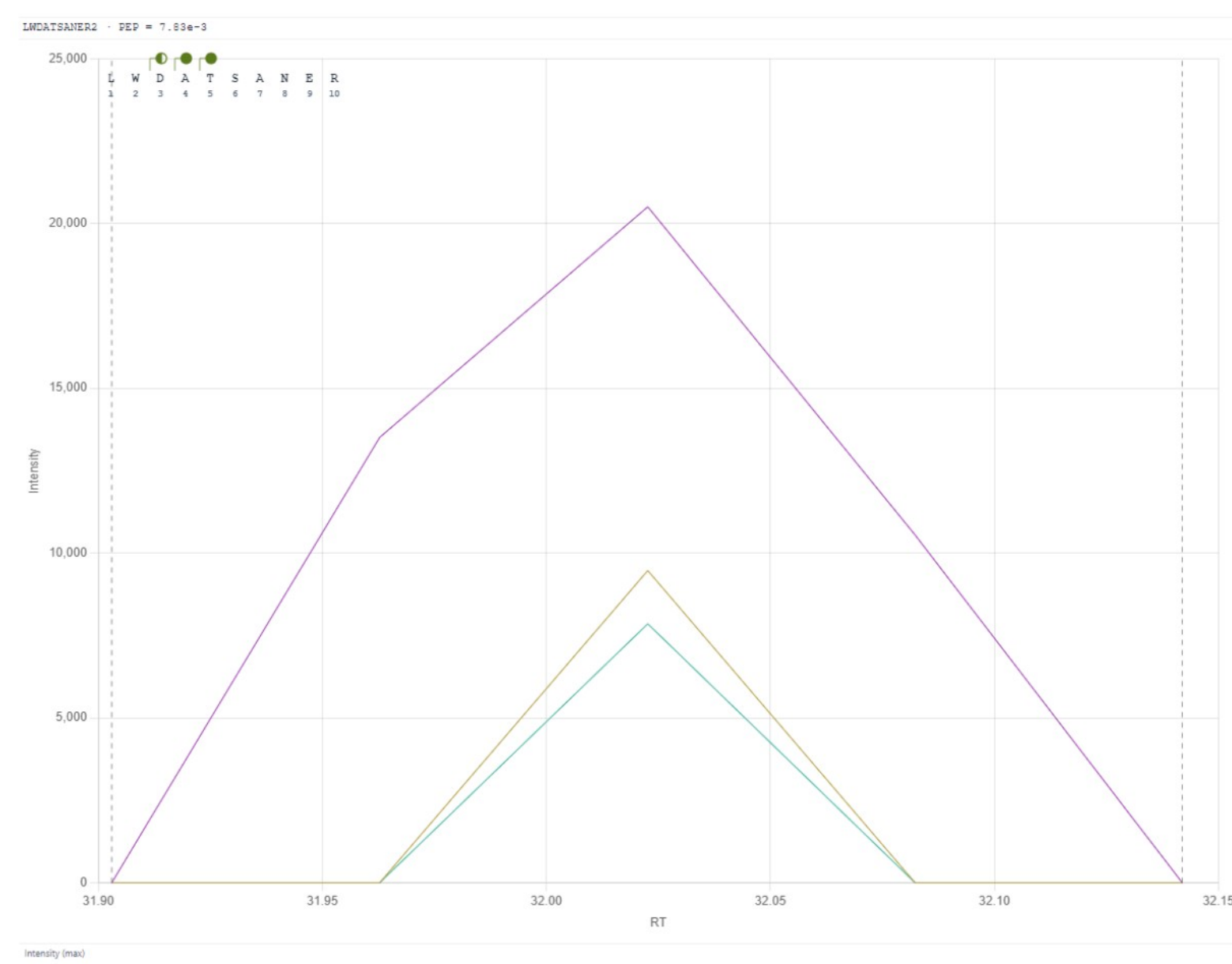

APP

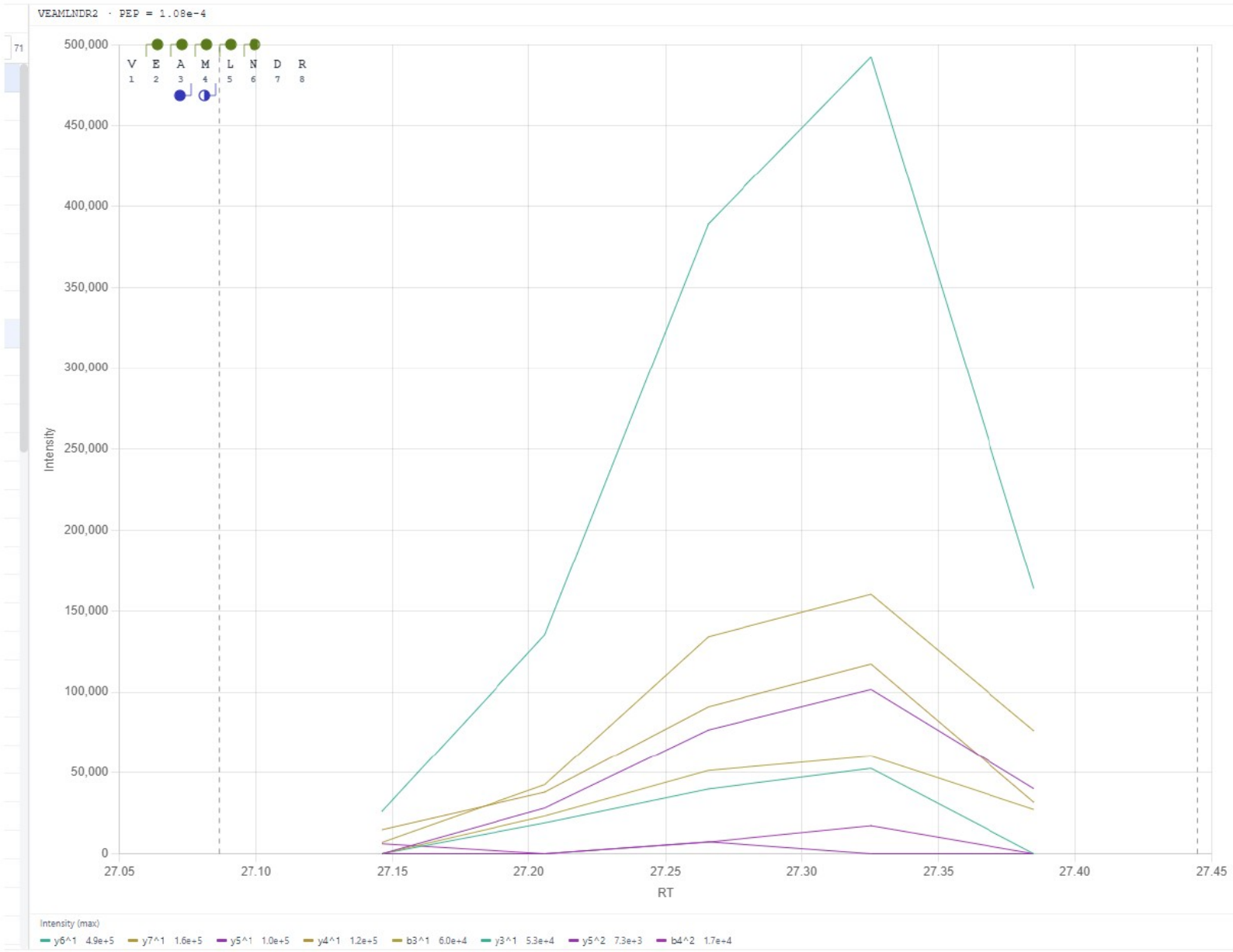

APOO

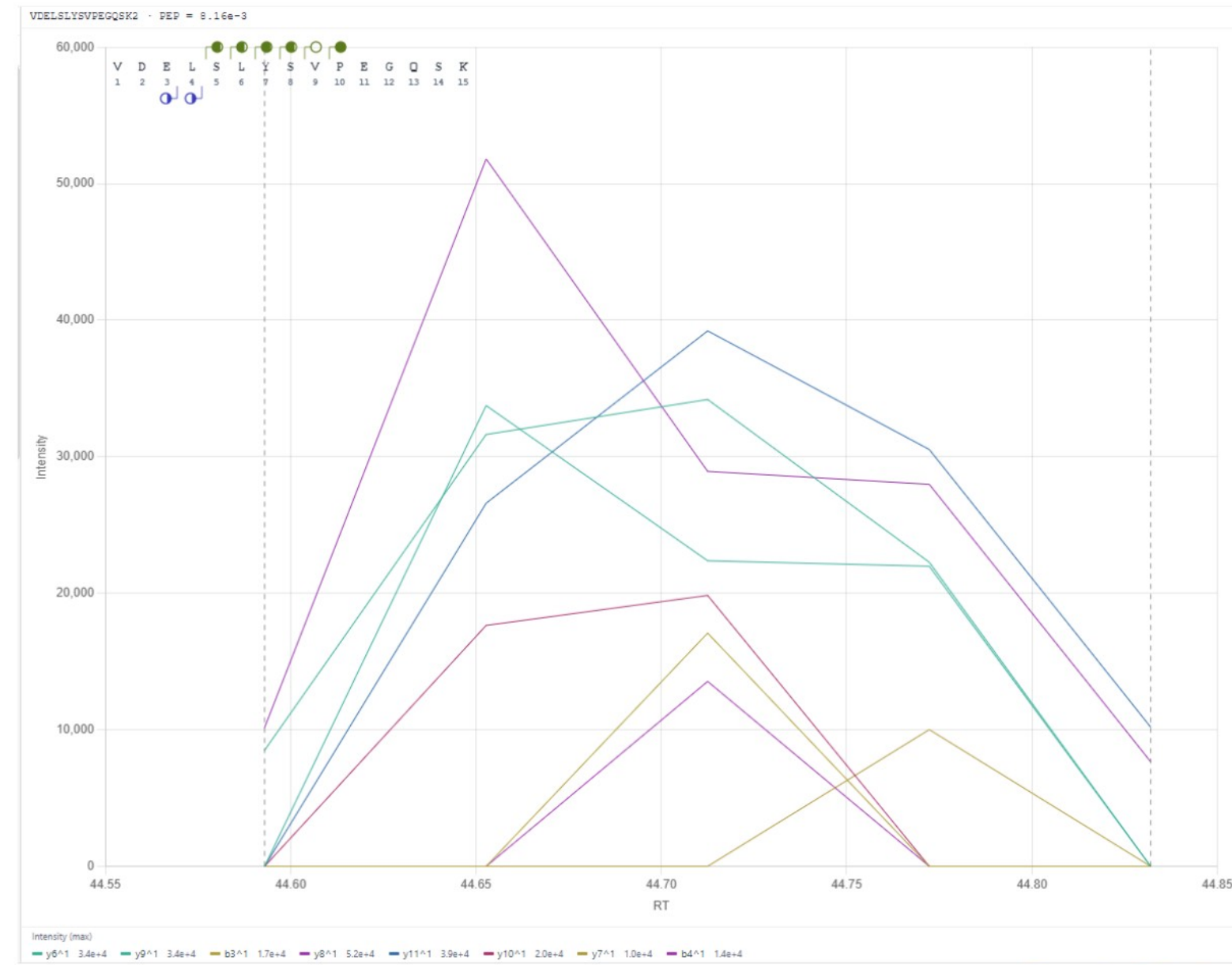

APPL1

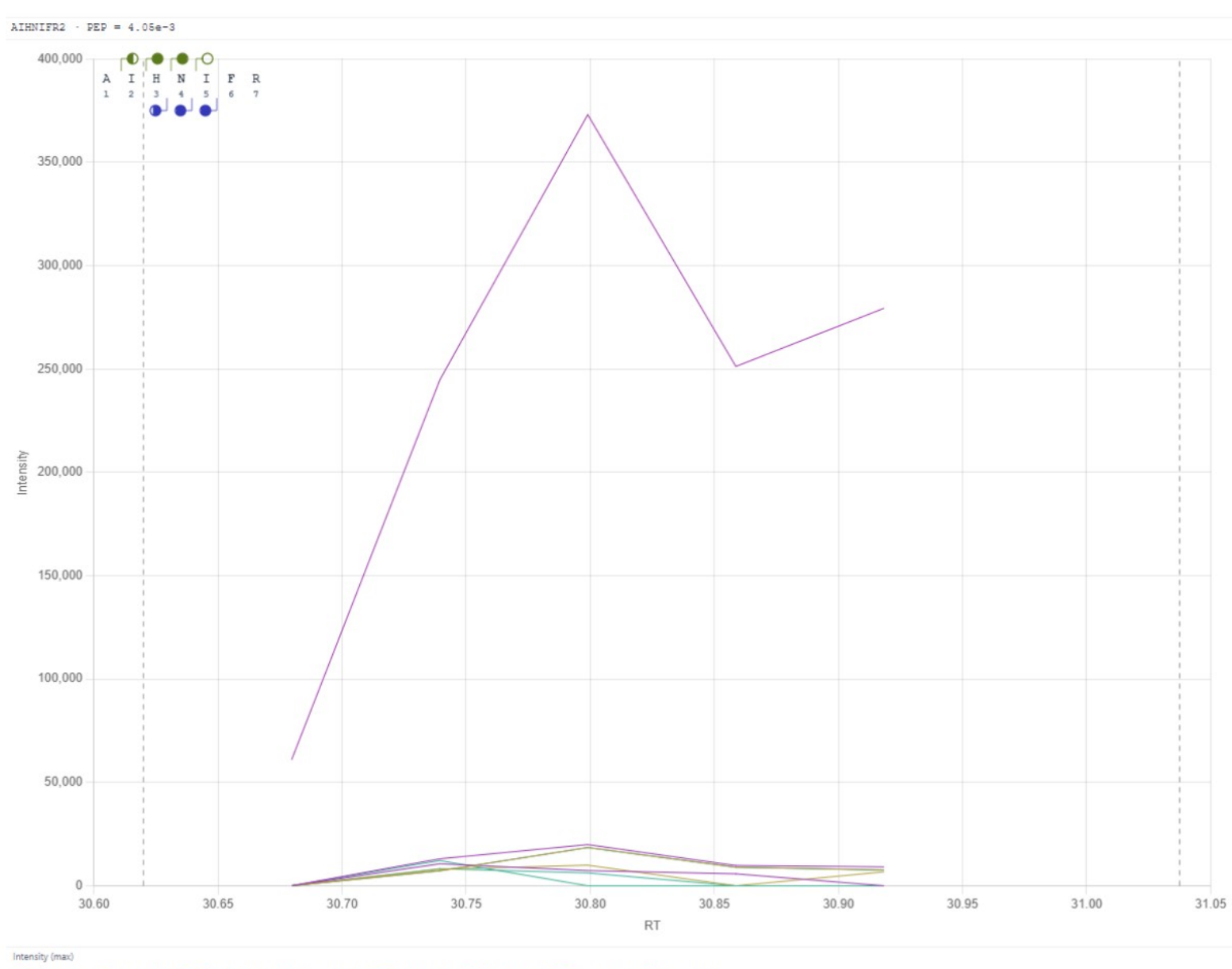

ARPC1B

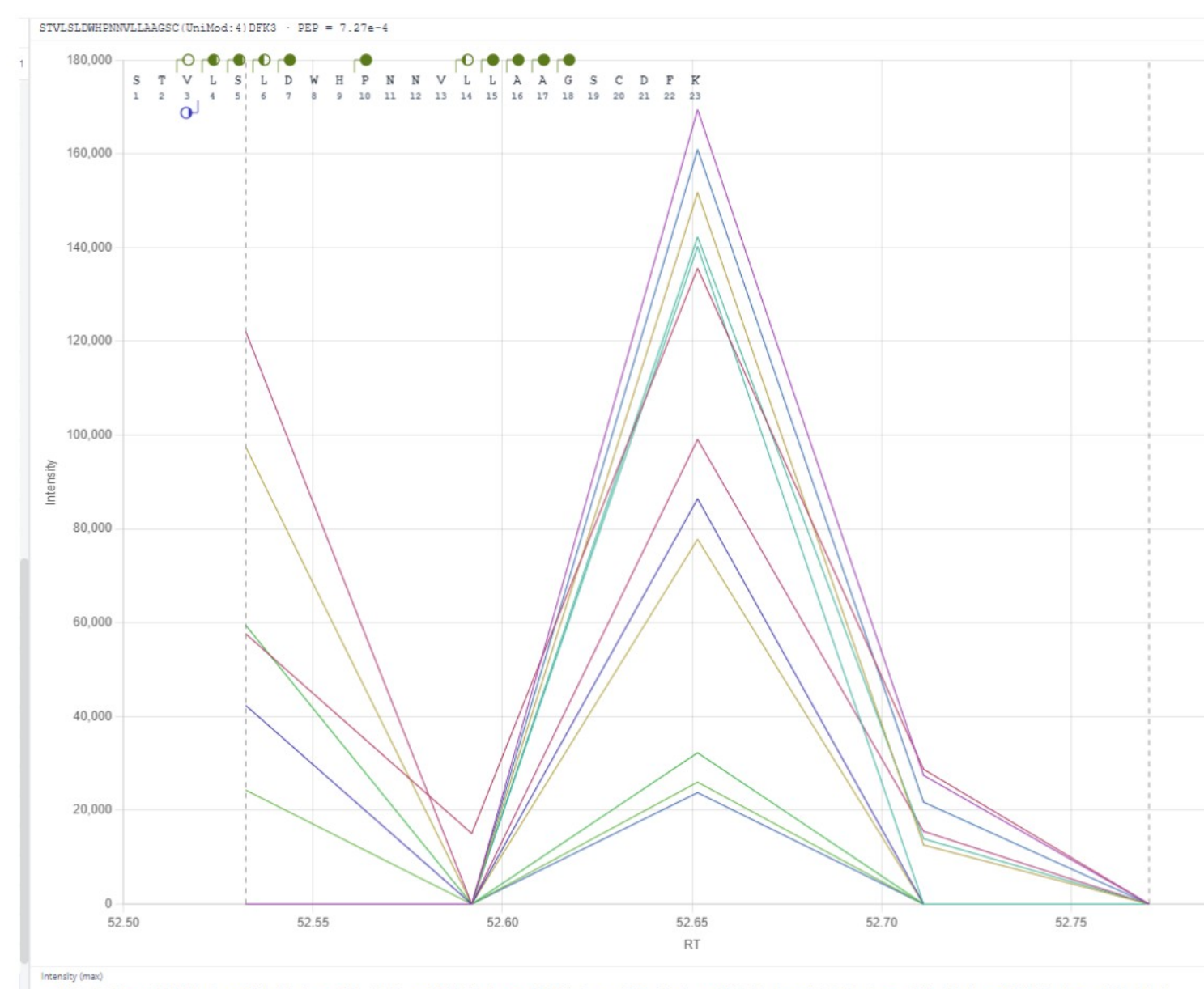

ARPC5

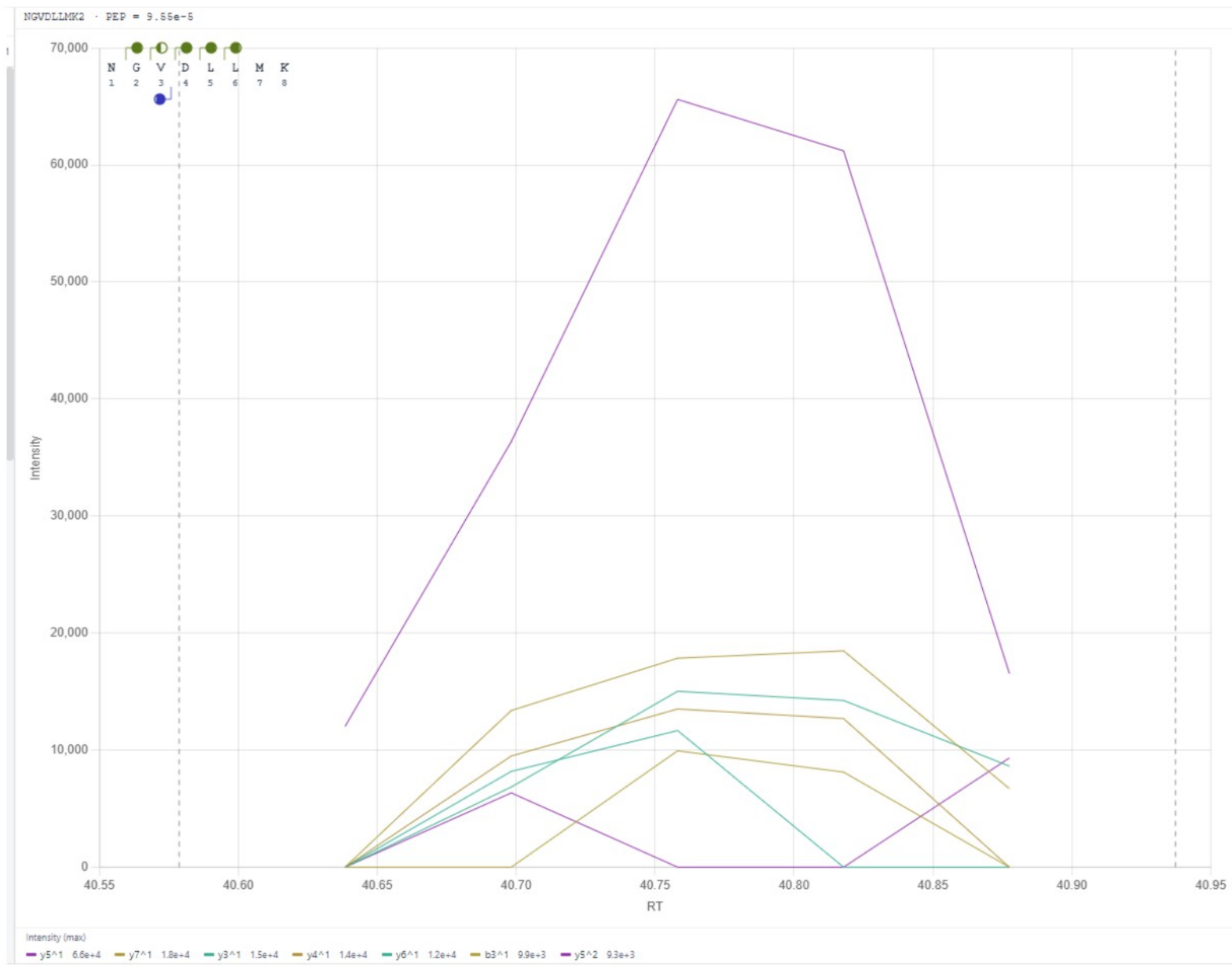

ATP6AP2

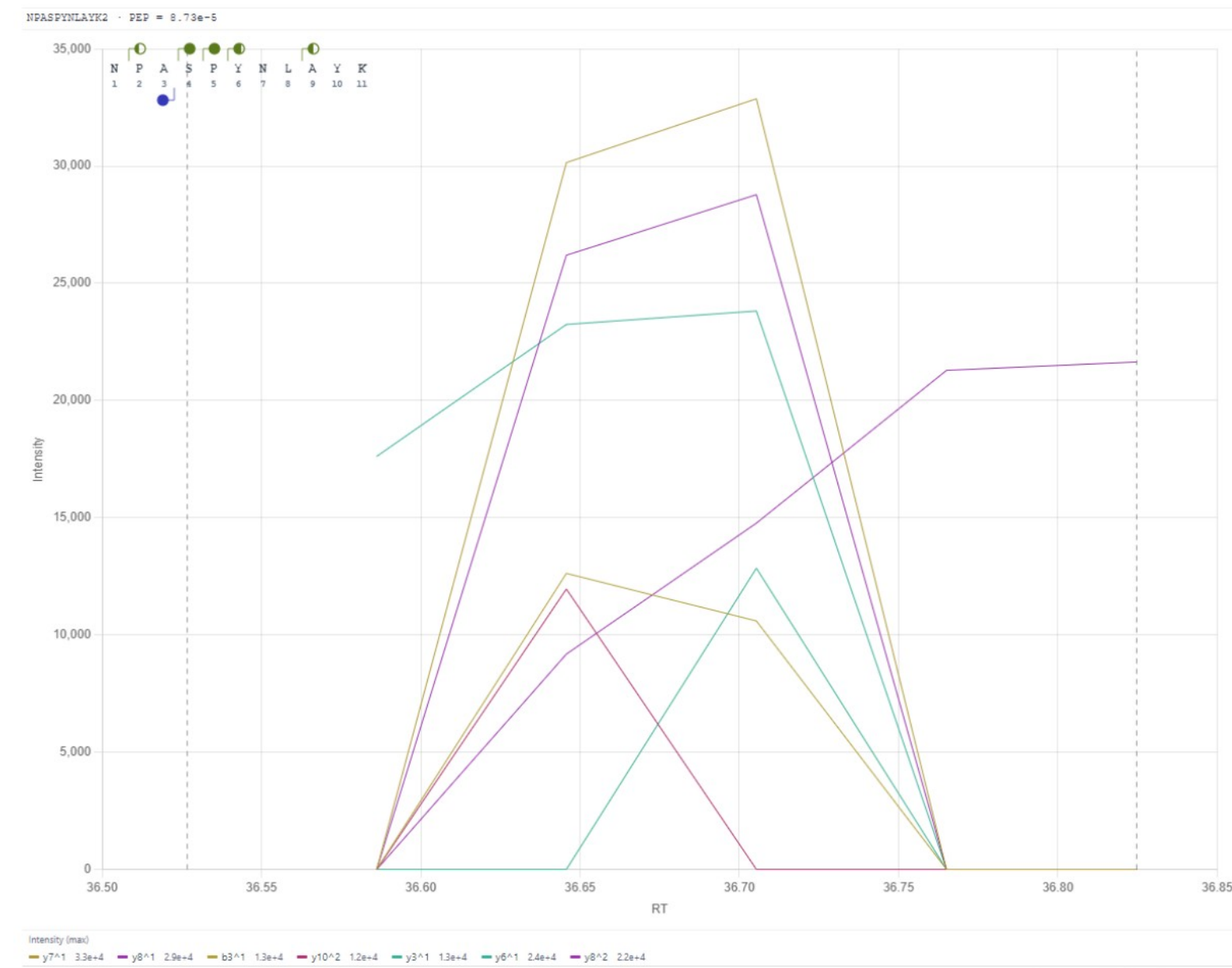

ATP6V0A1

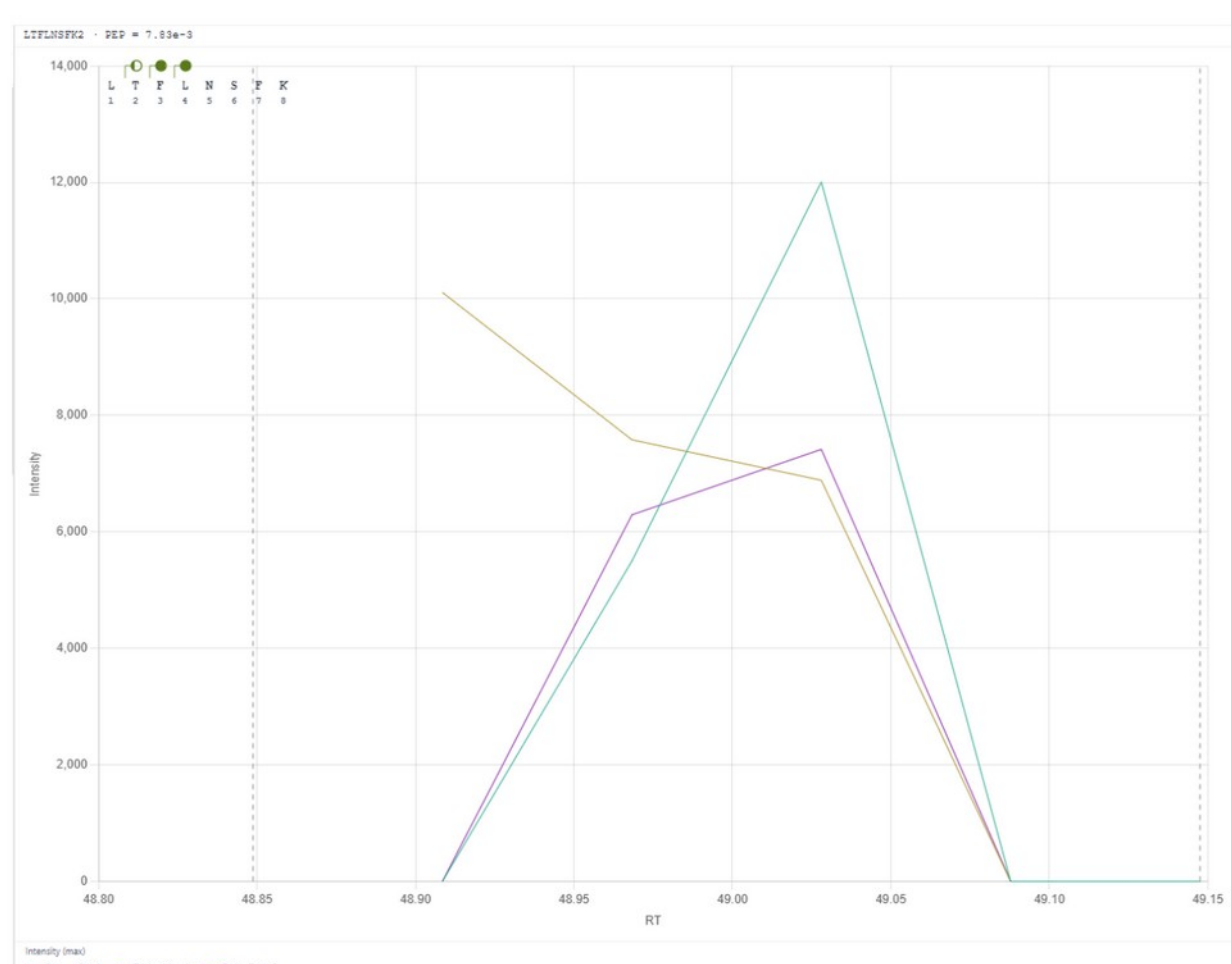

atpF1

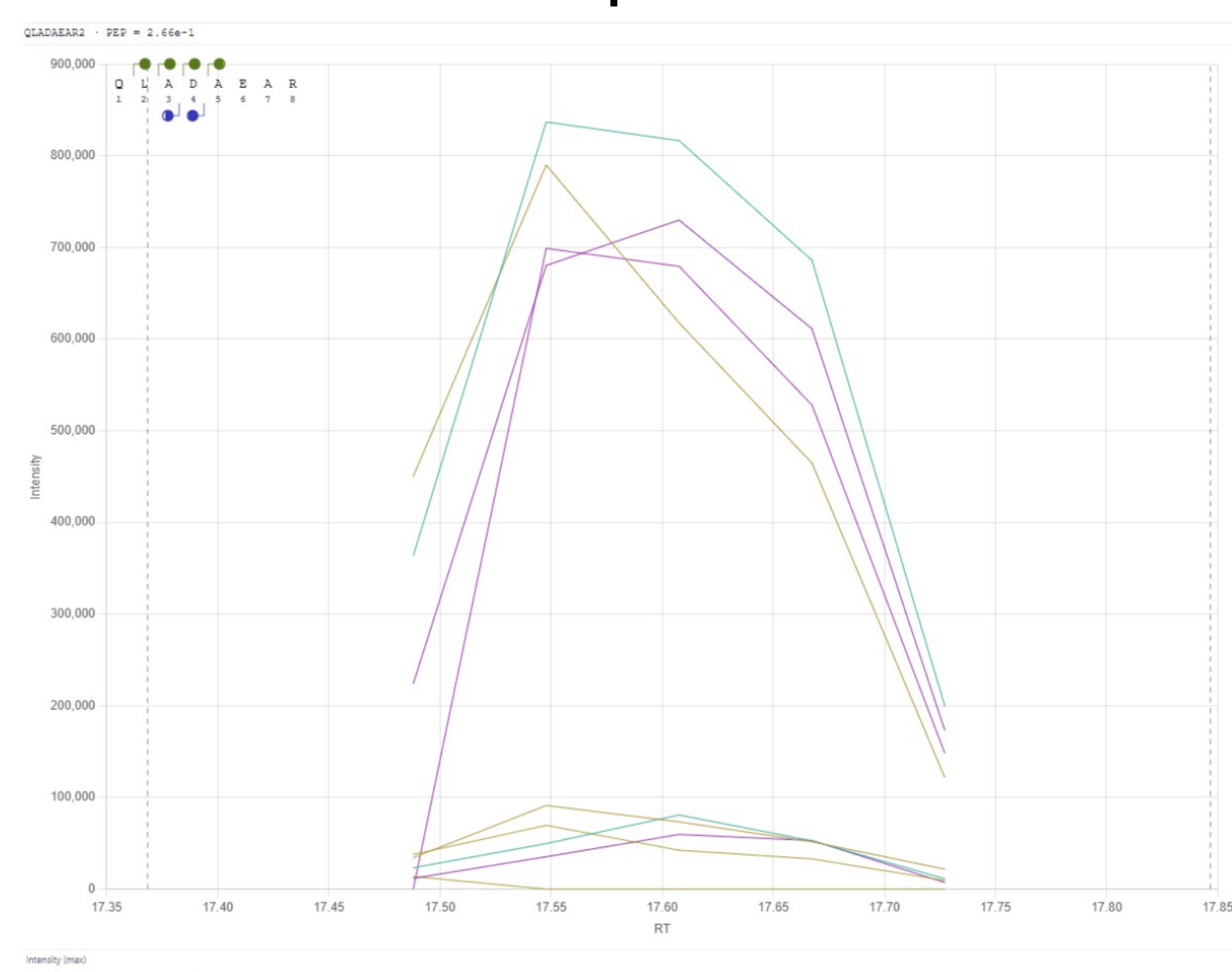

BAIAP3

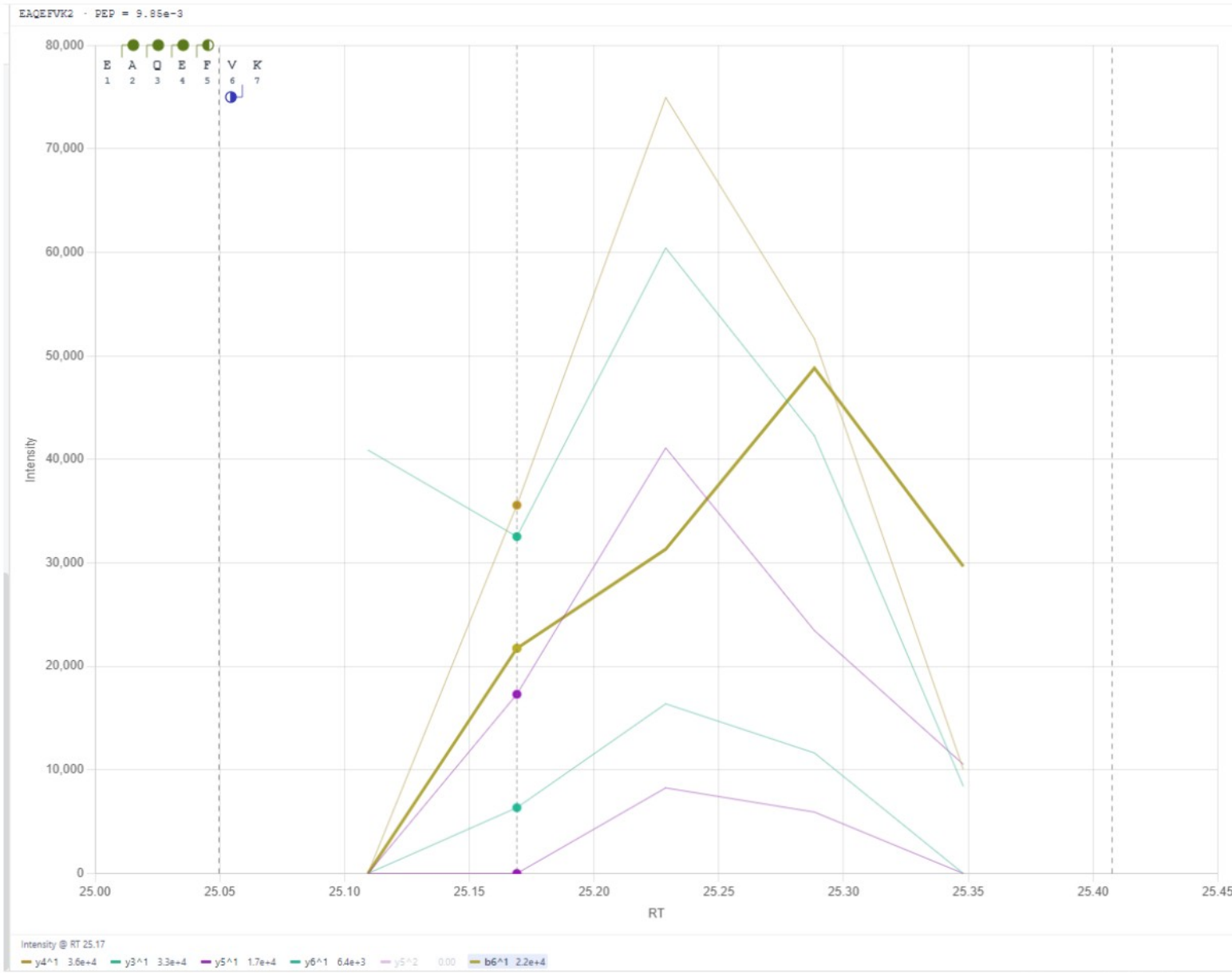

BCAT1

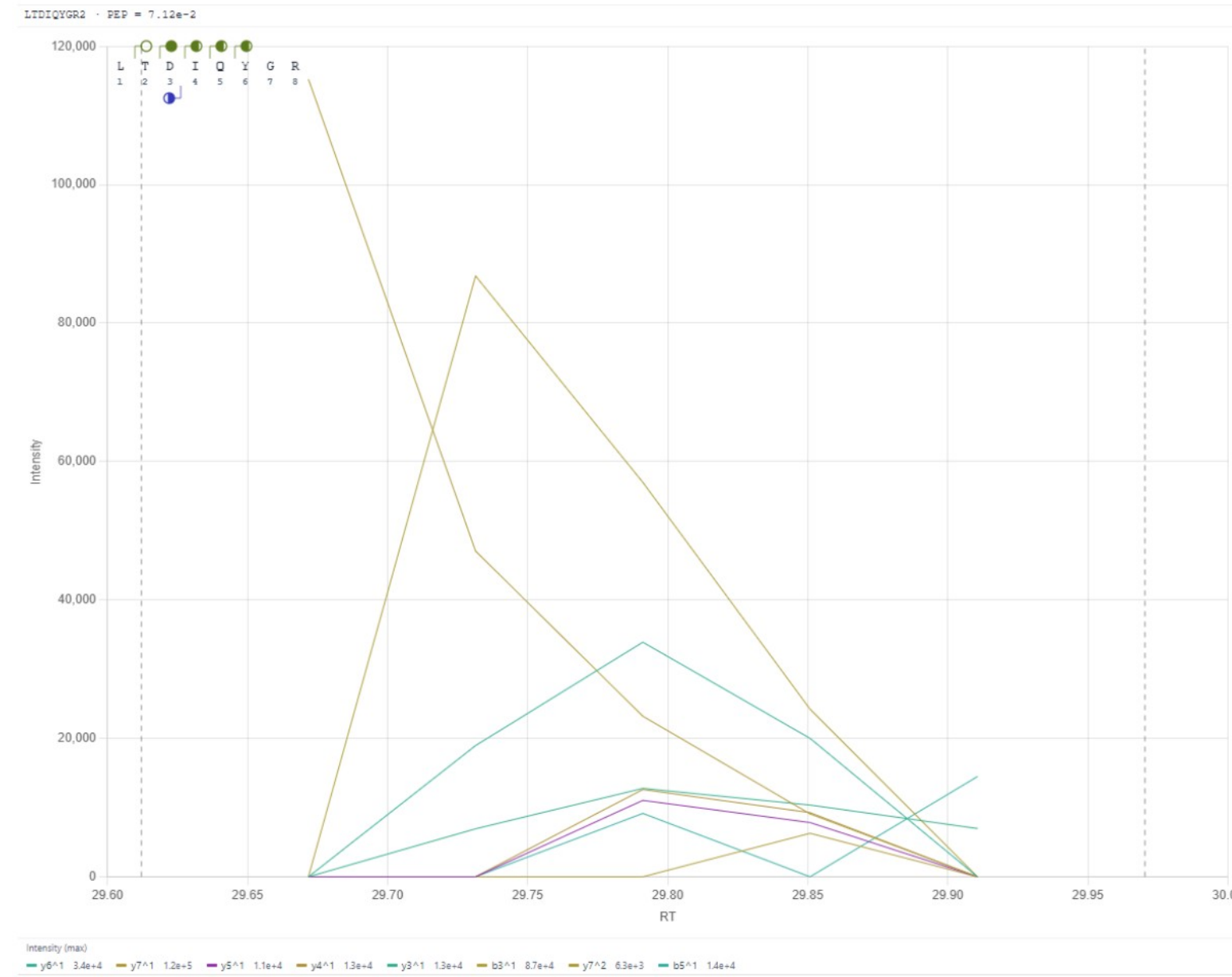

BET1L

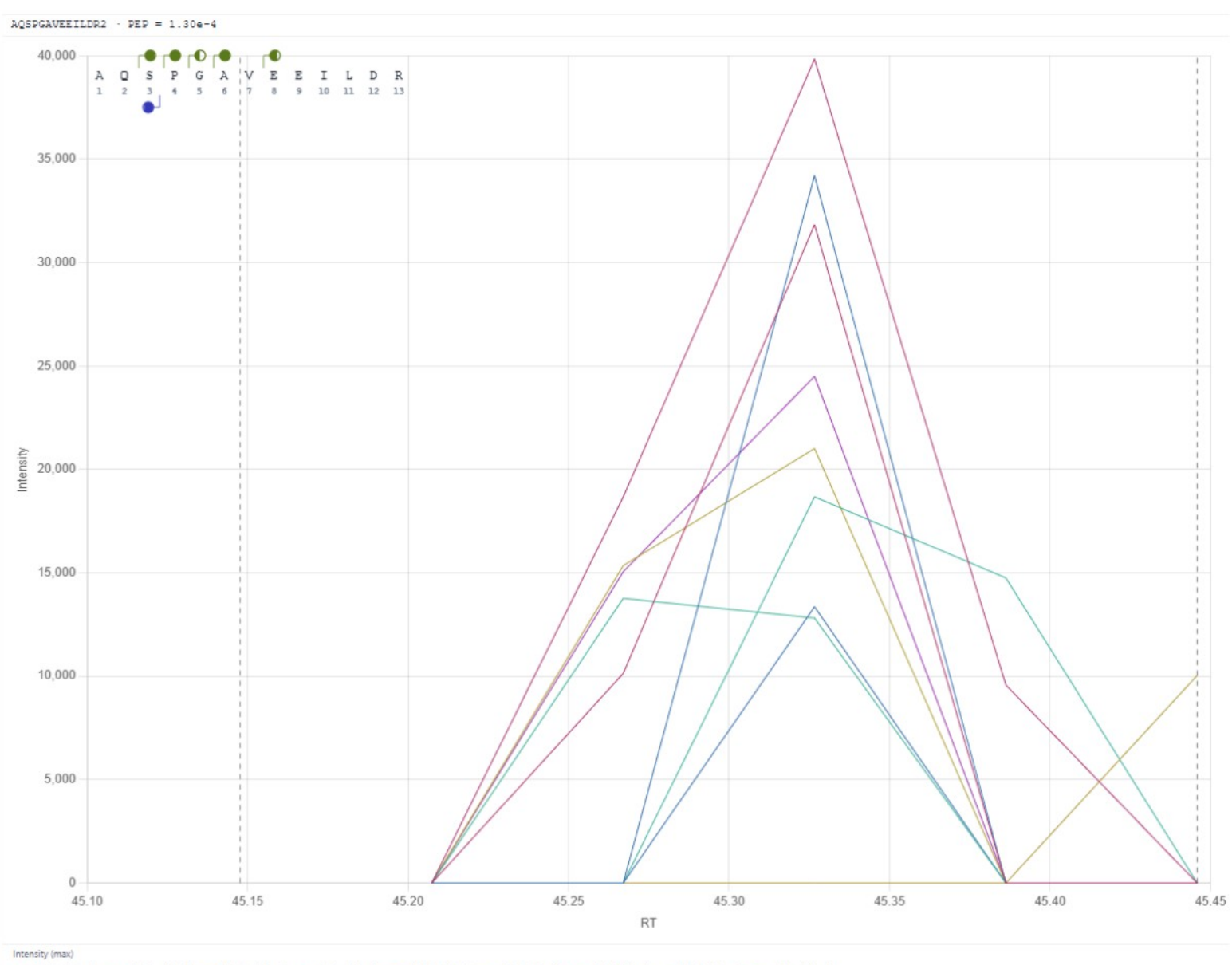

BMP2K *

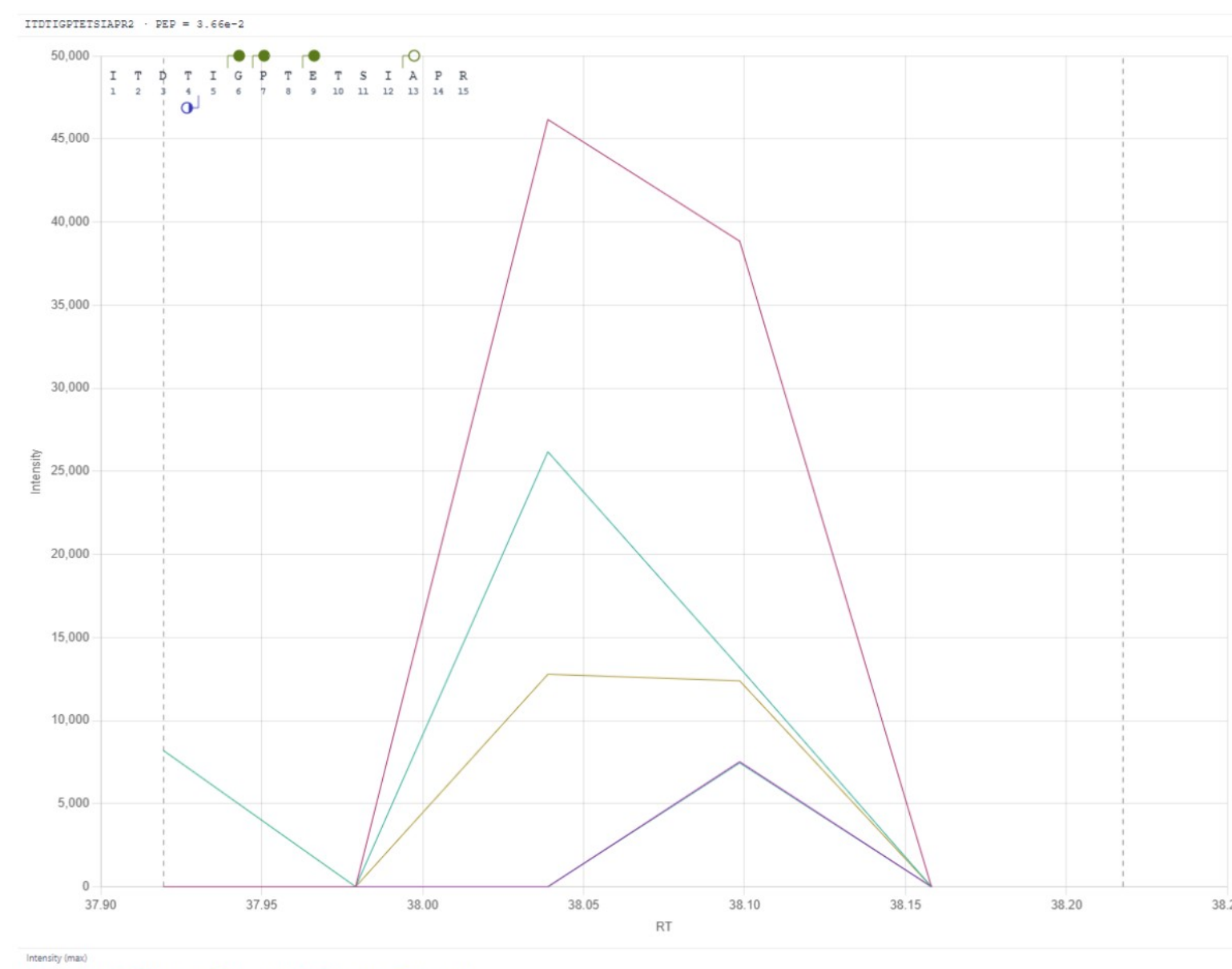

BZW1

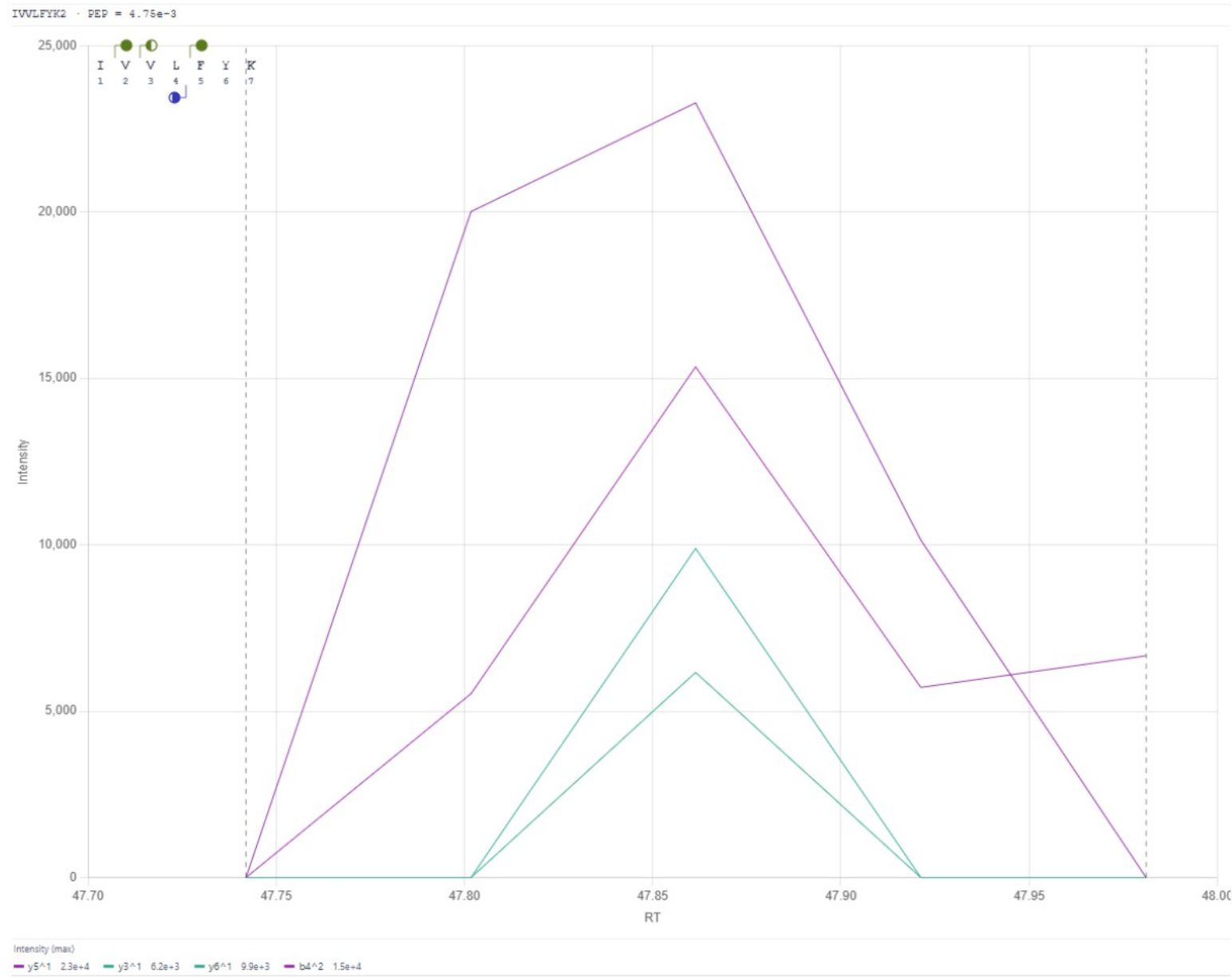

C1GALT1

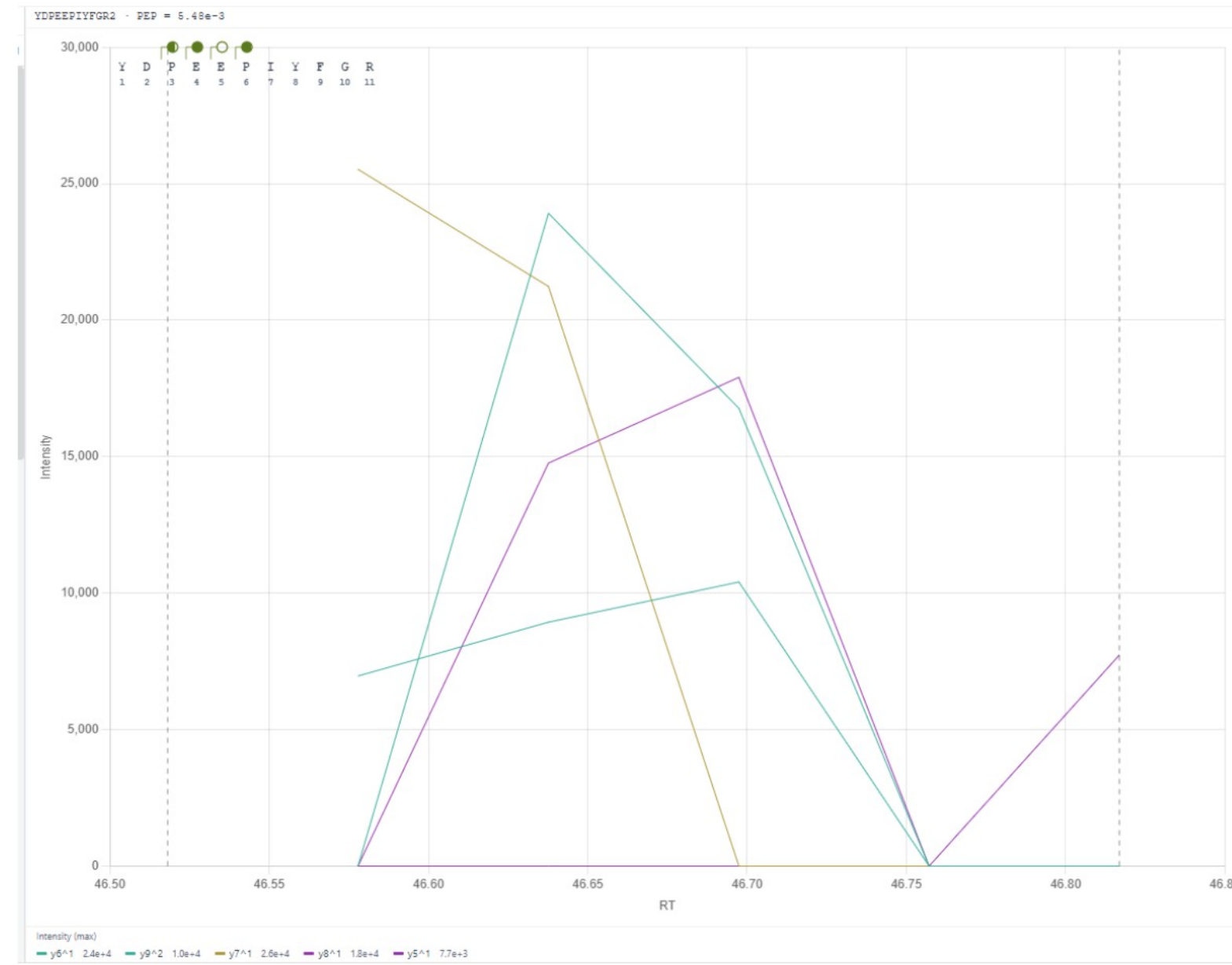

C1R

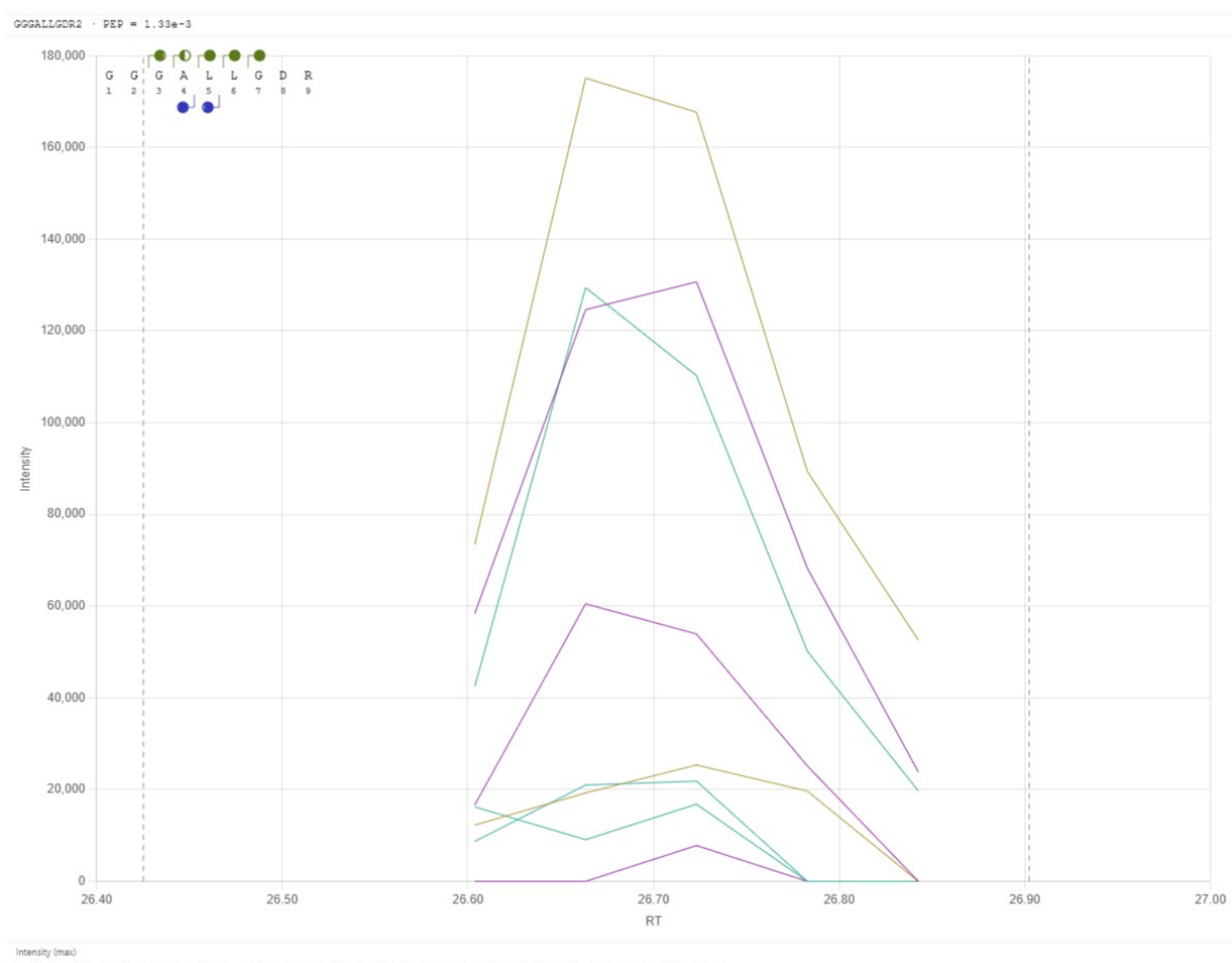

C1S

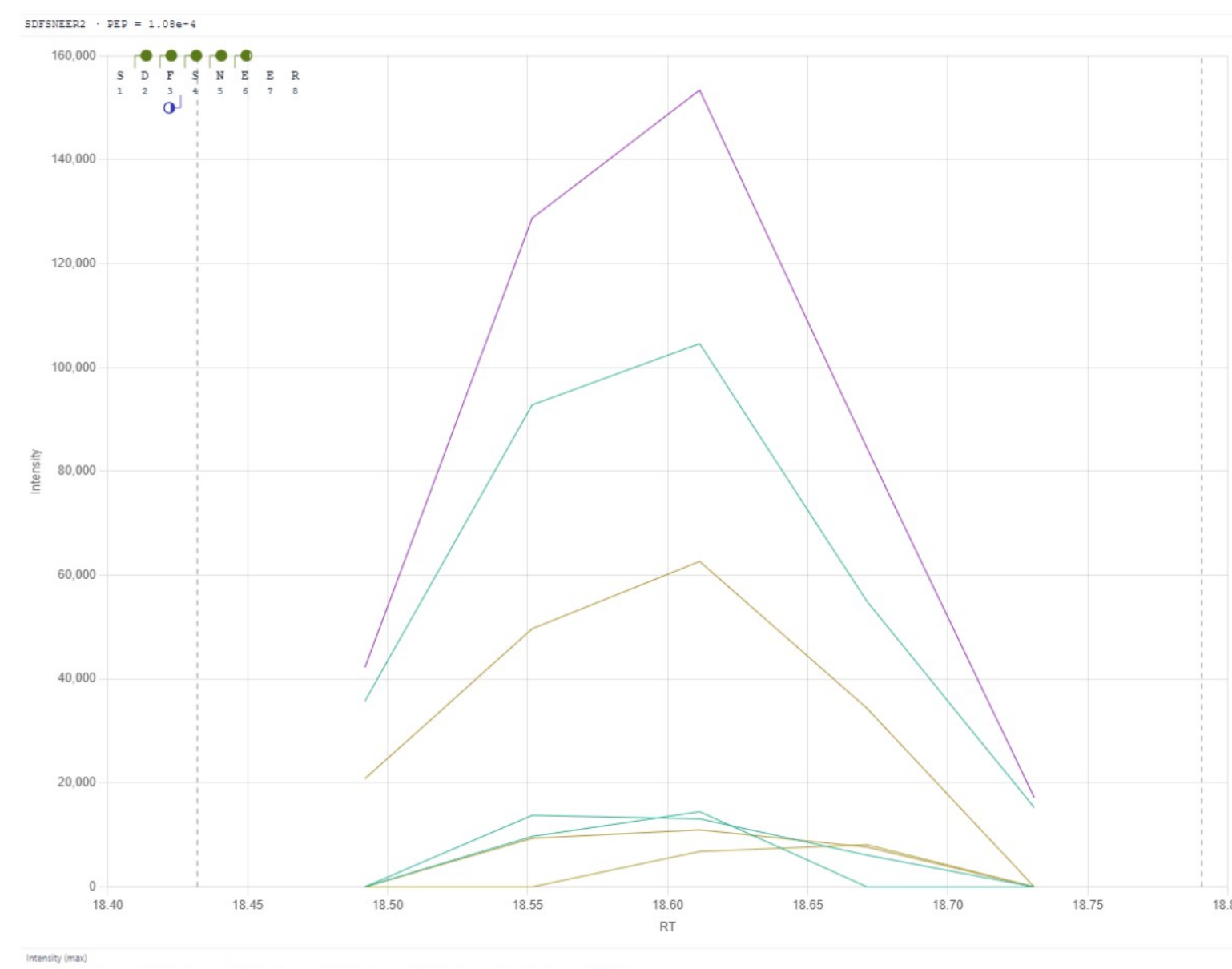

C4BPB

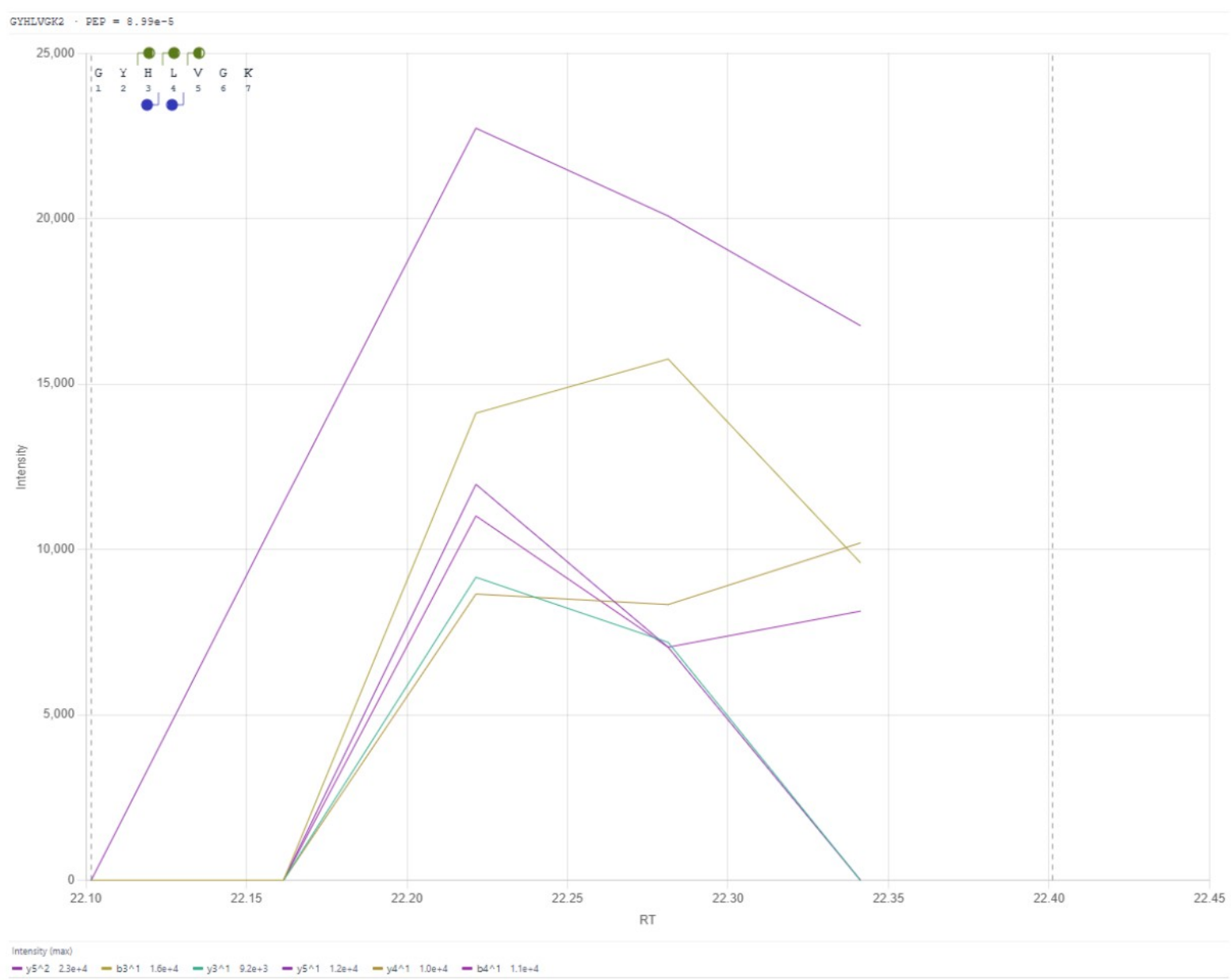

CANX

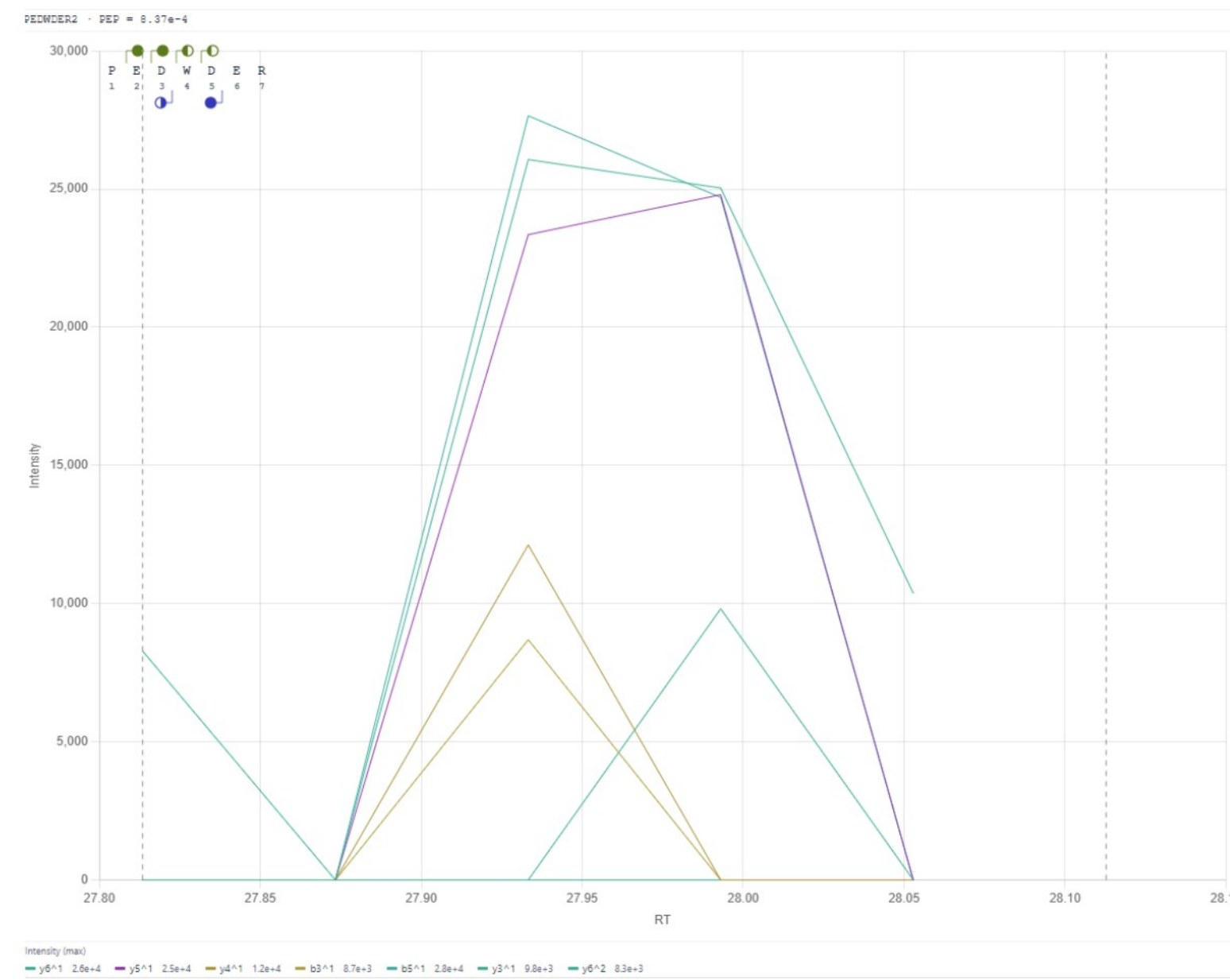

CARD9

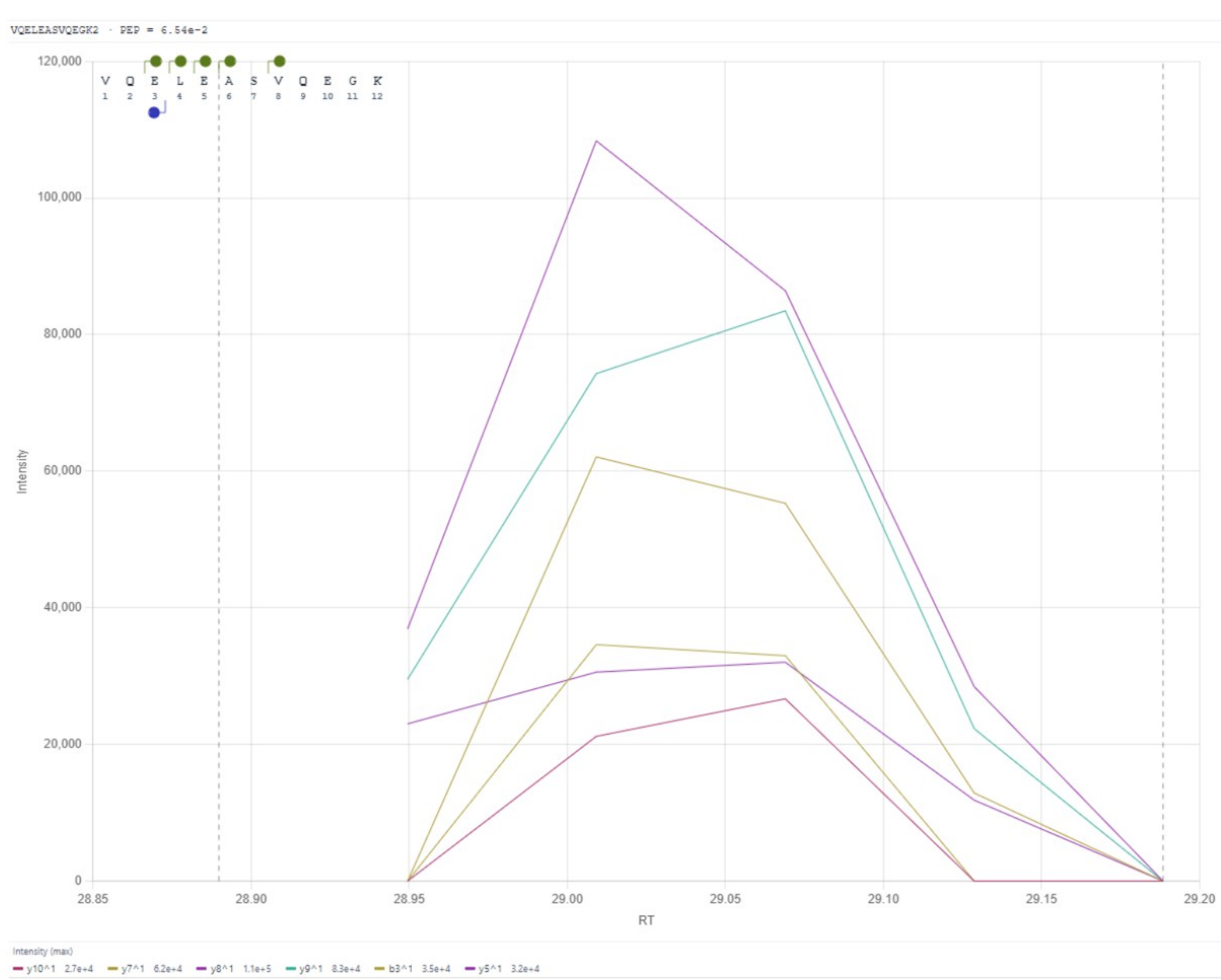

CARHSP1

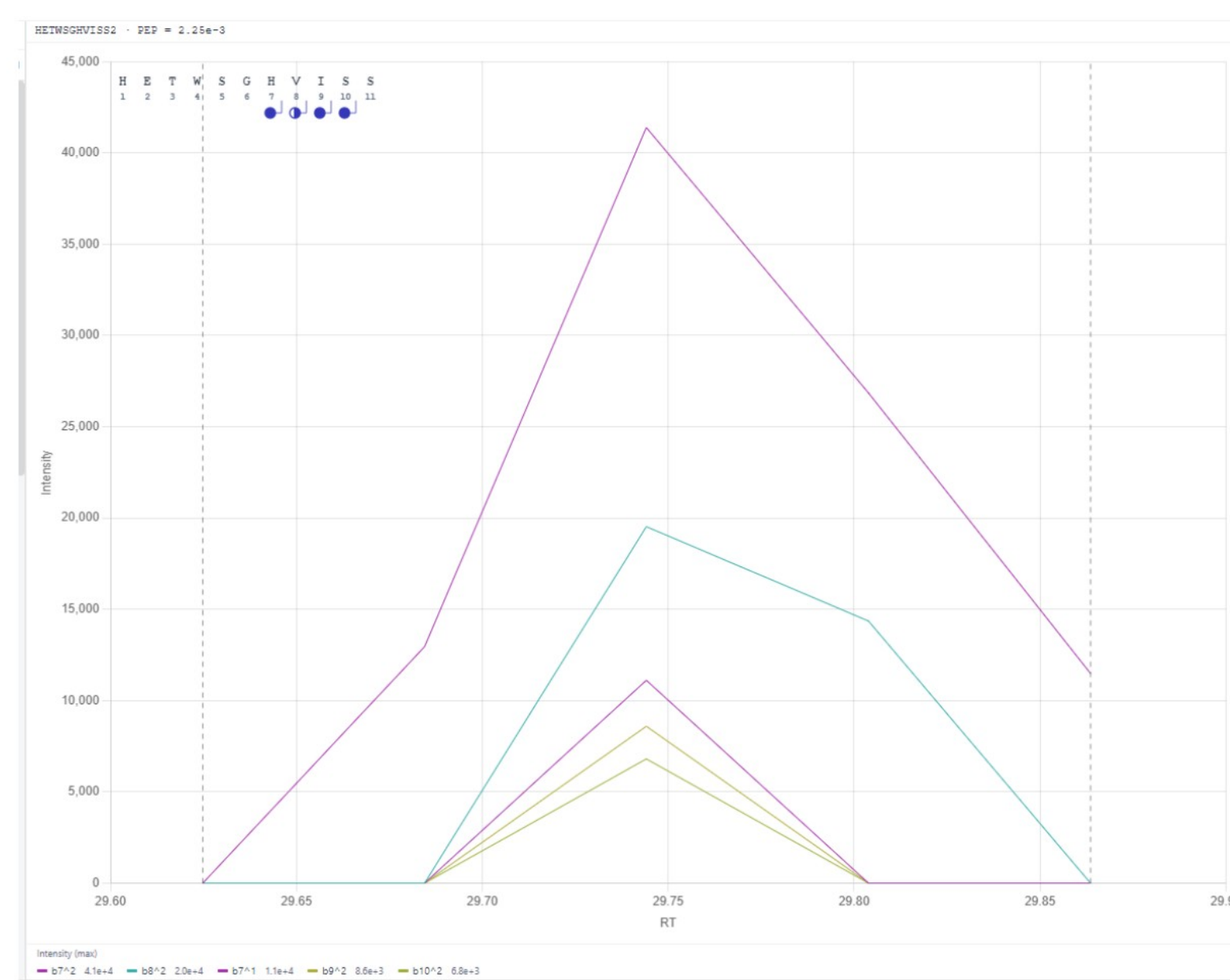

CCDC86

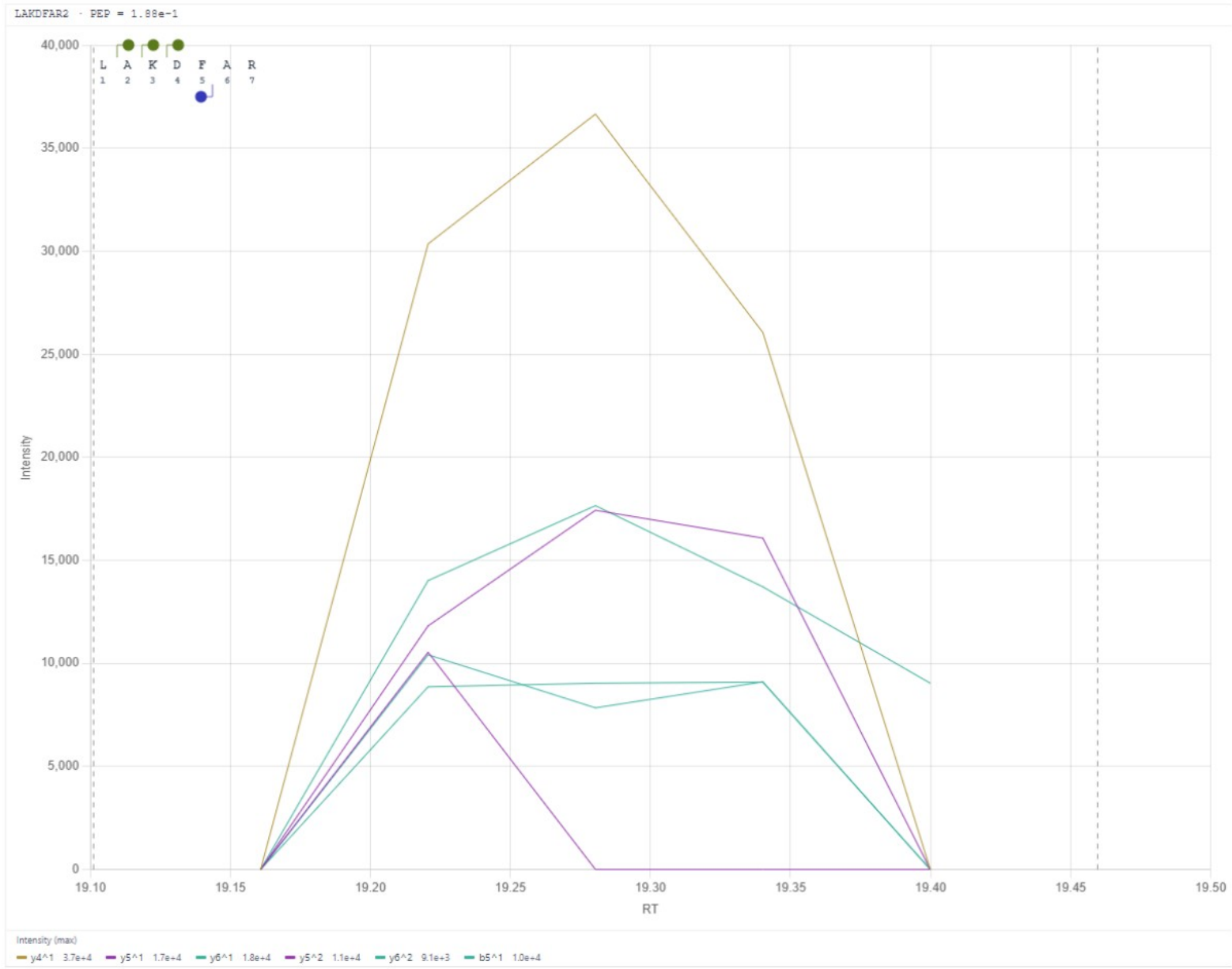

CCT6B

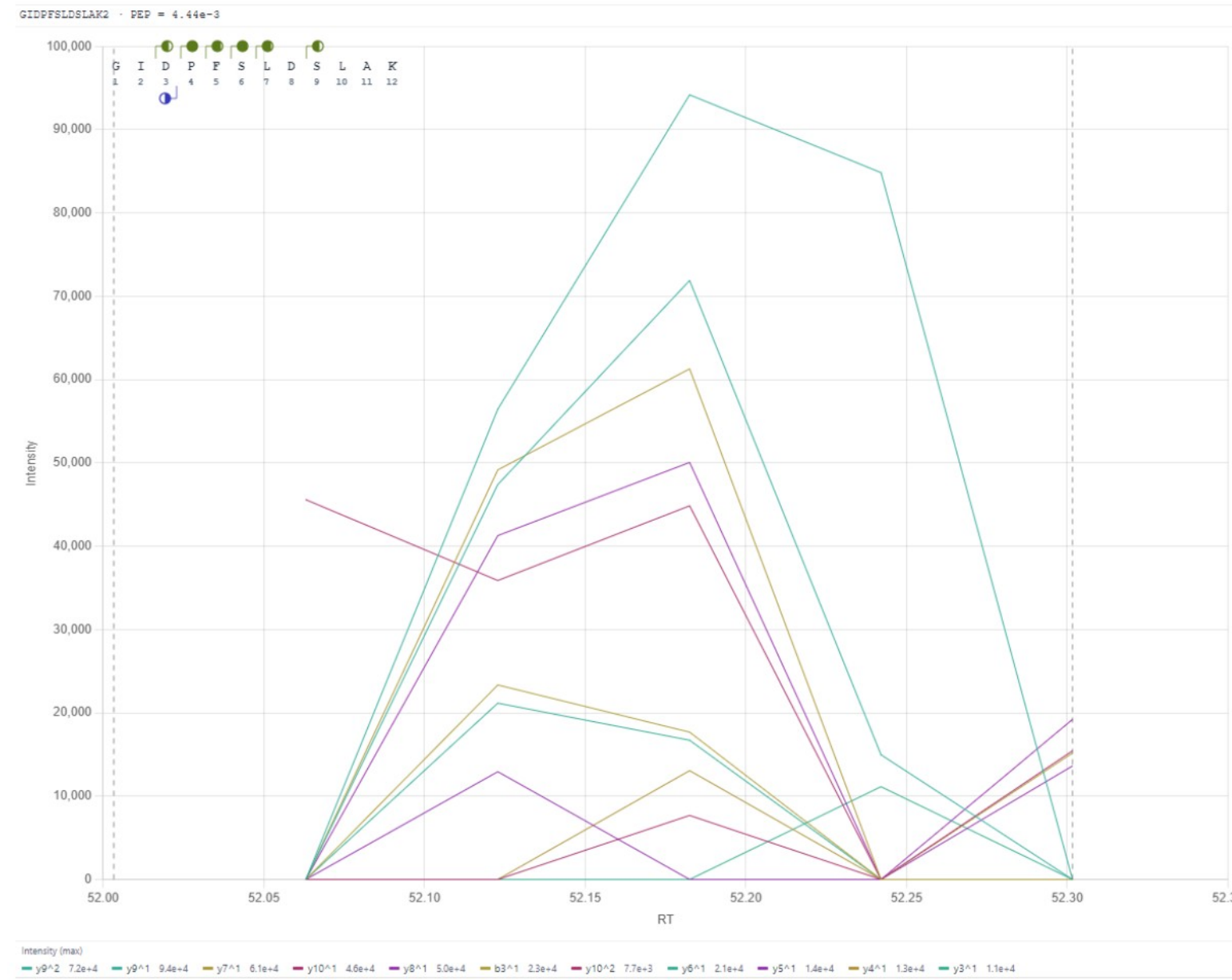

CD44

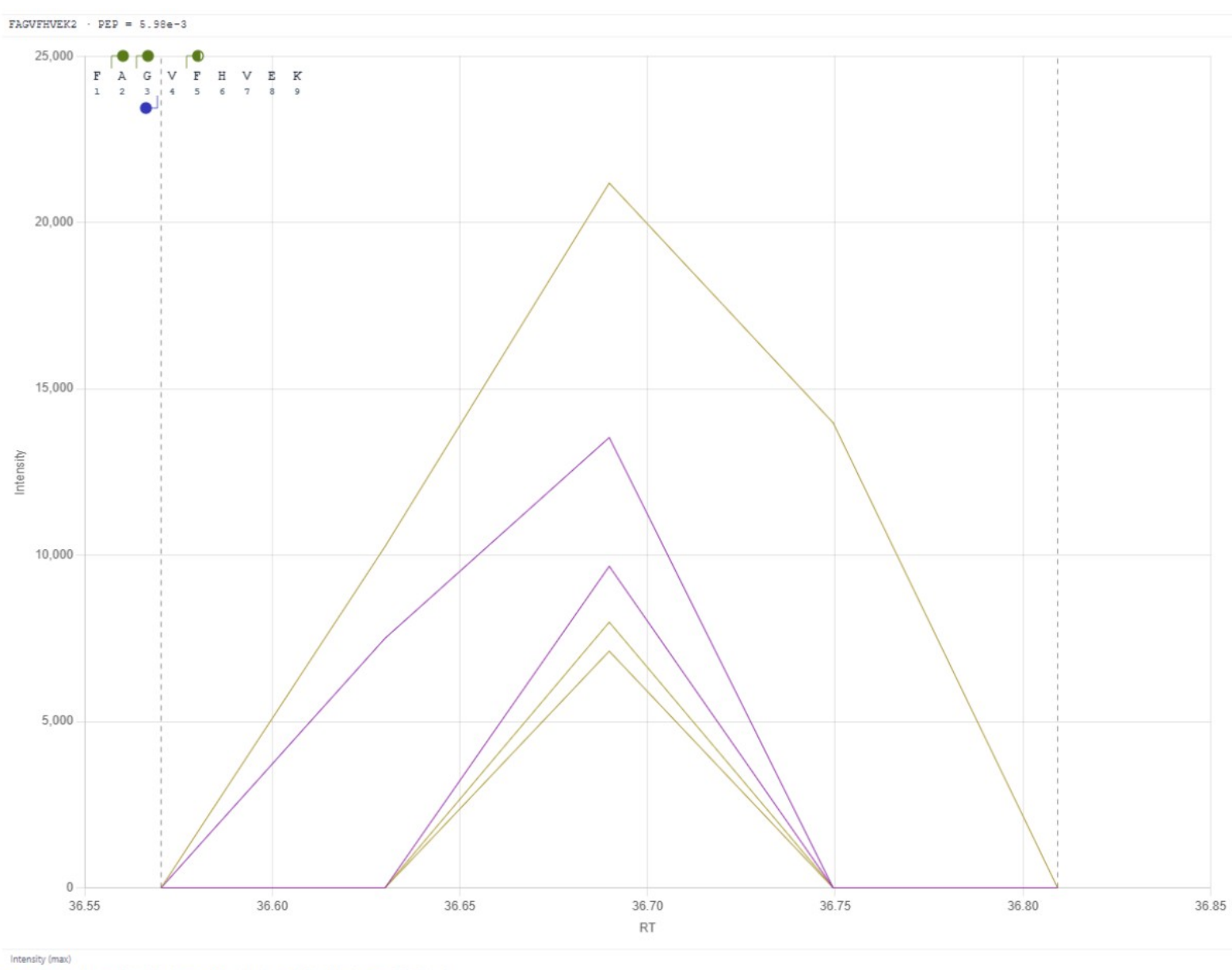

CDC42

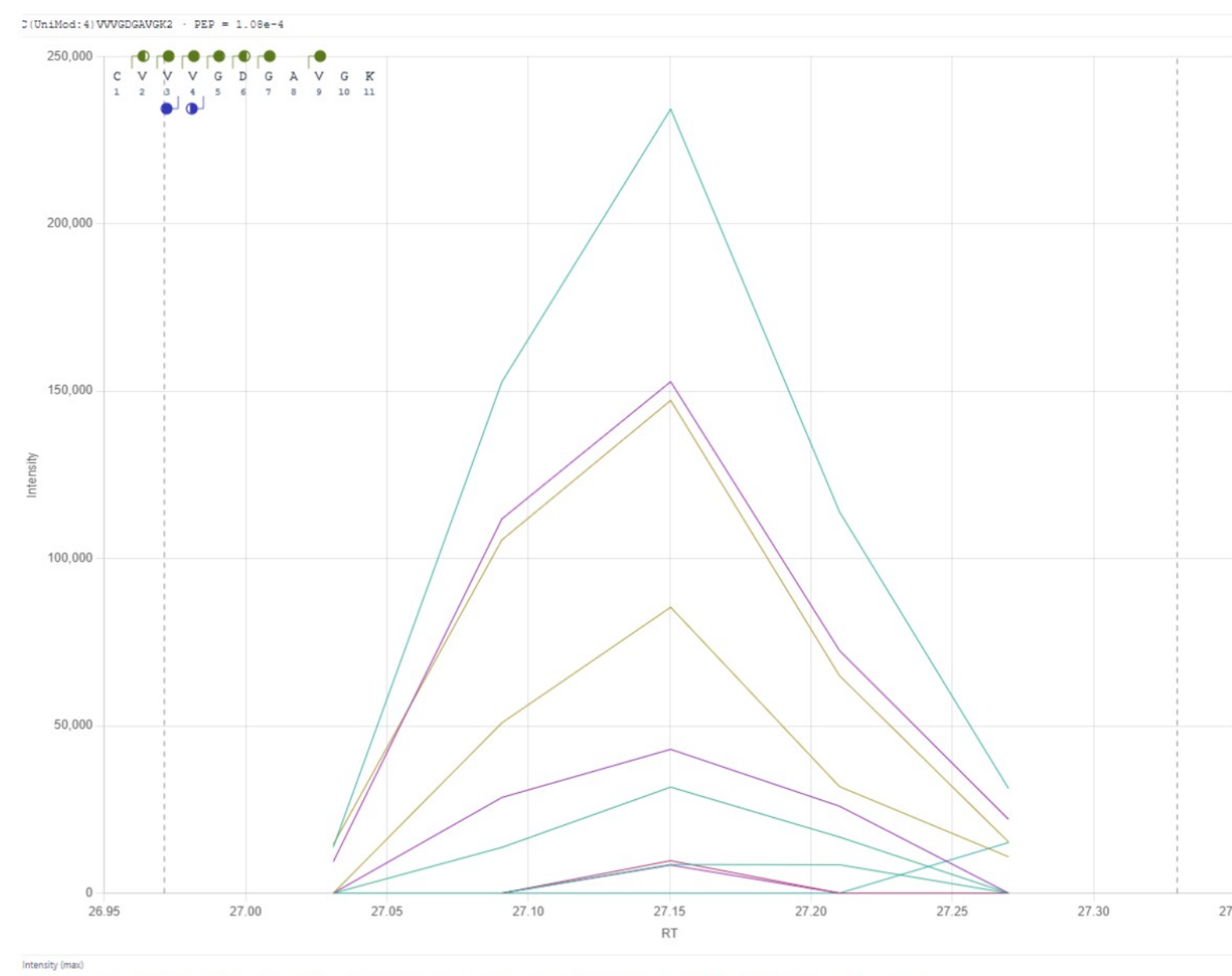

CDC42SE2

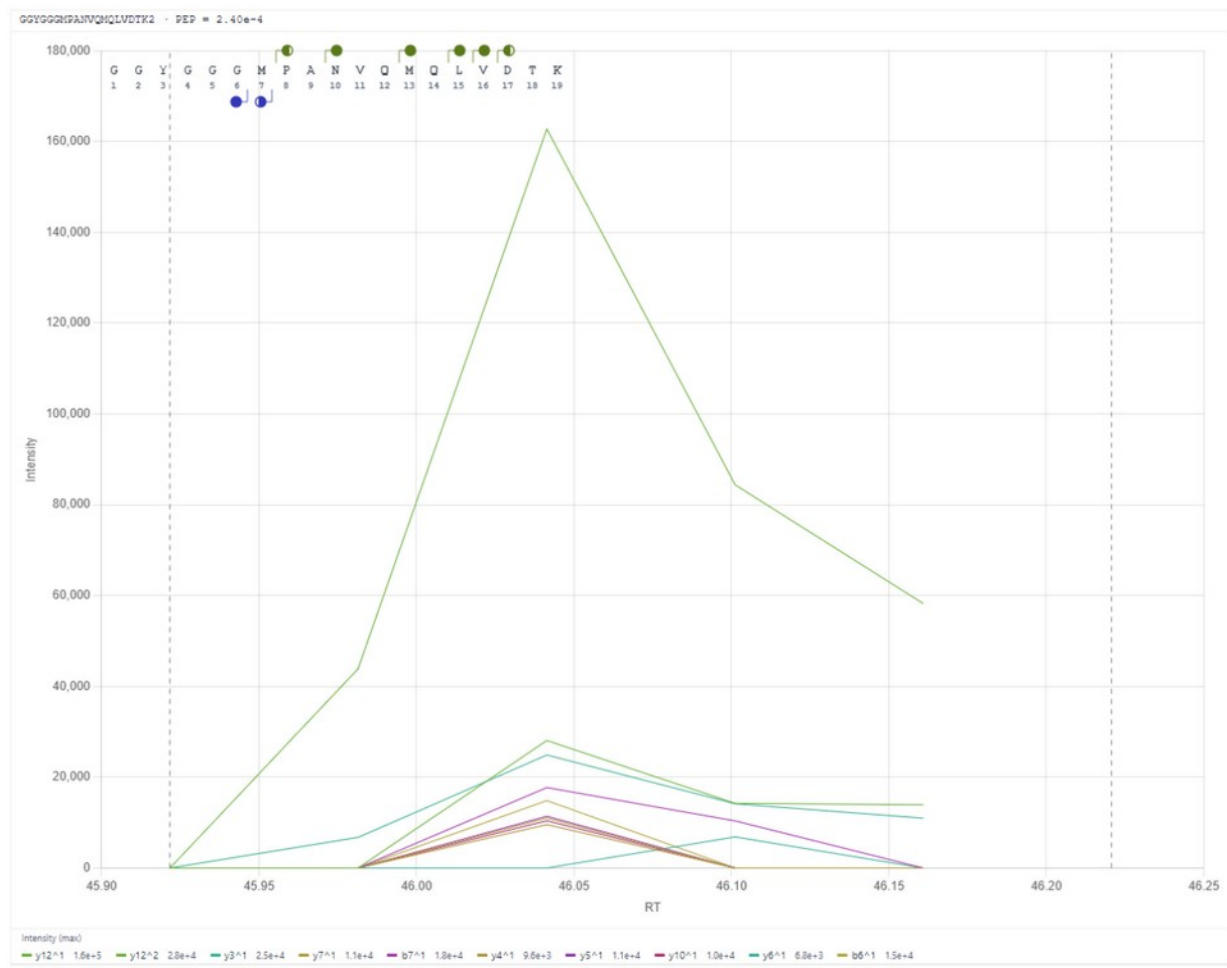

CERT1

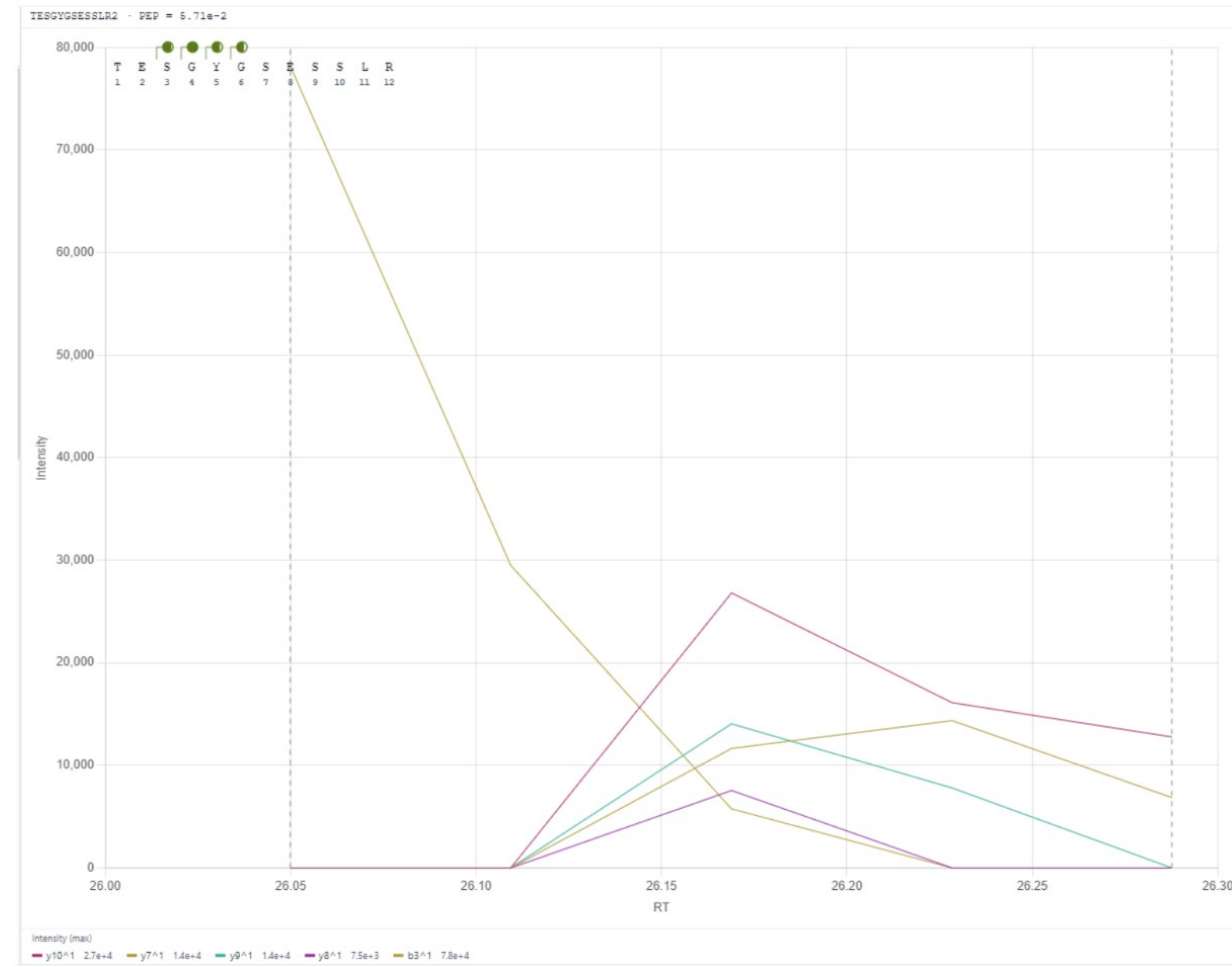

CES1

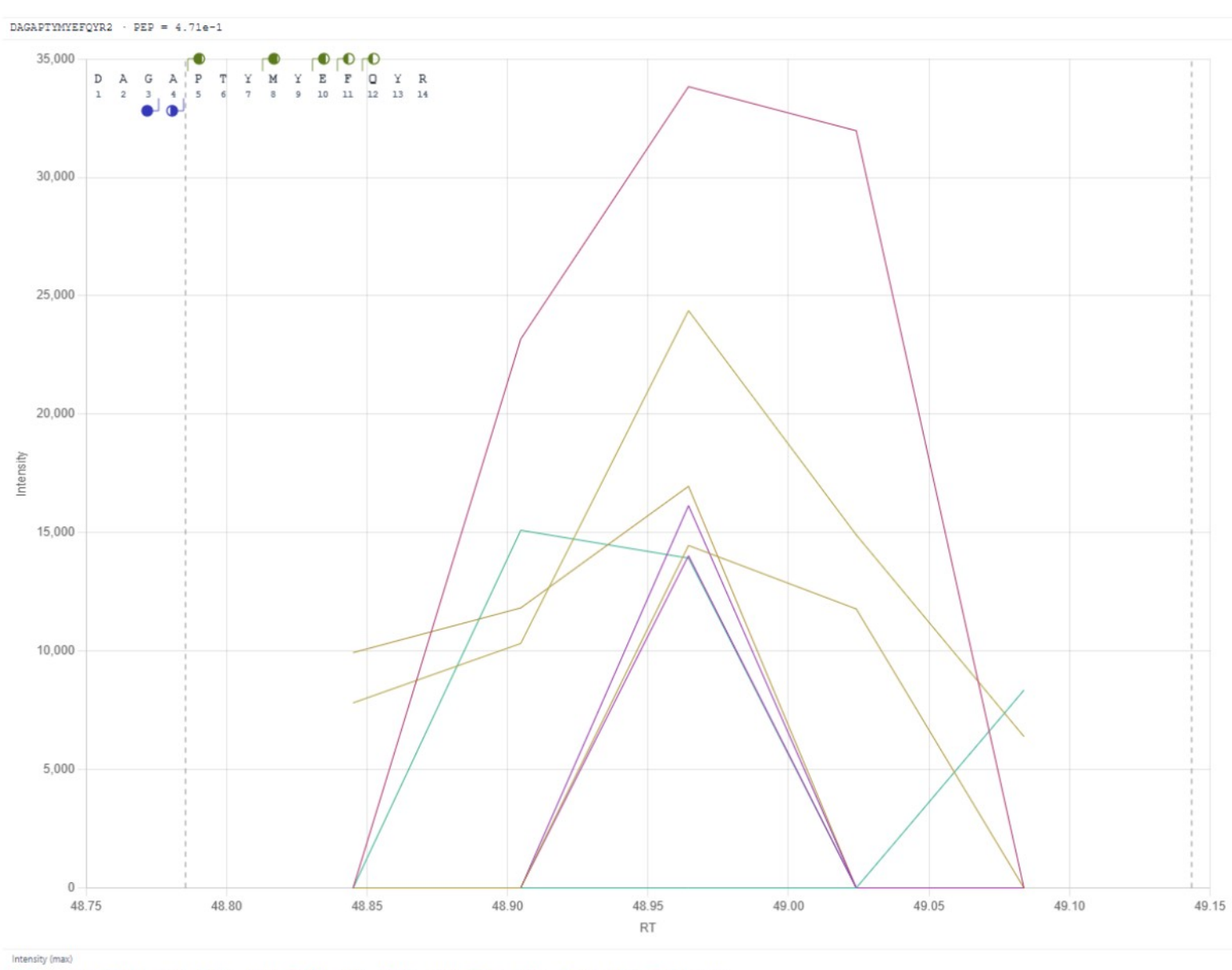

CFH

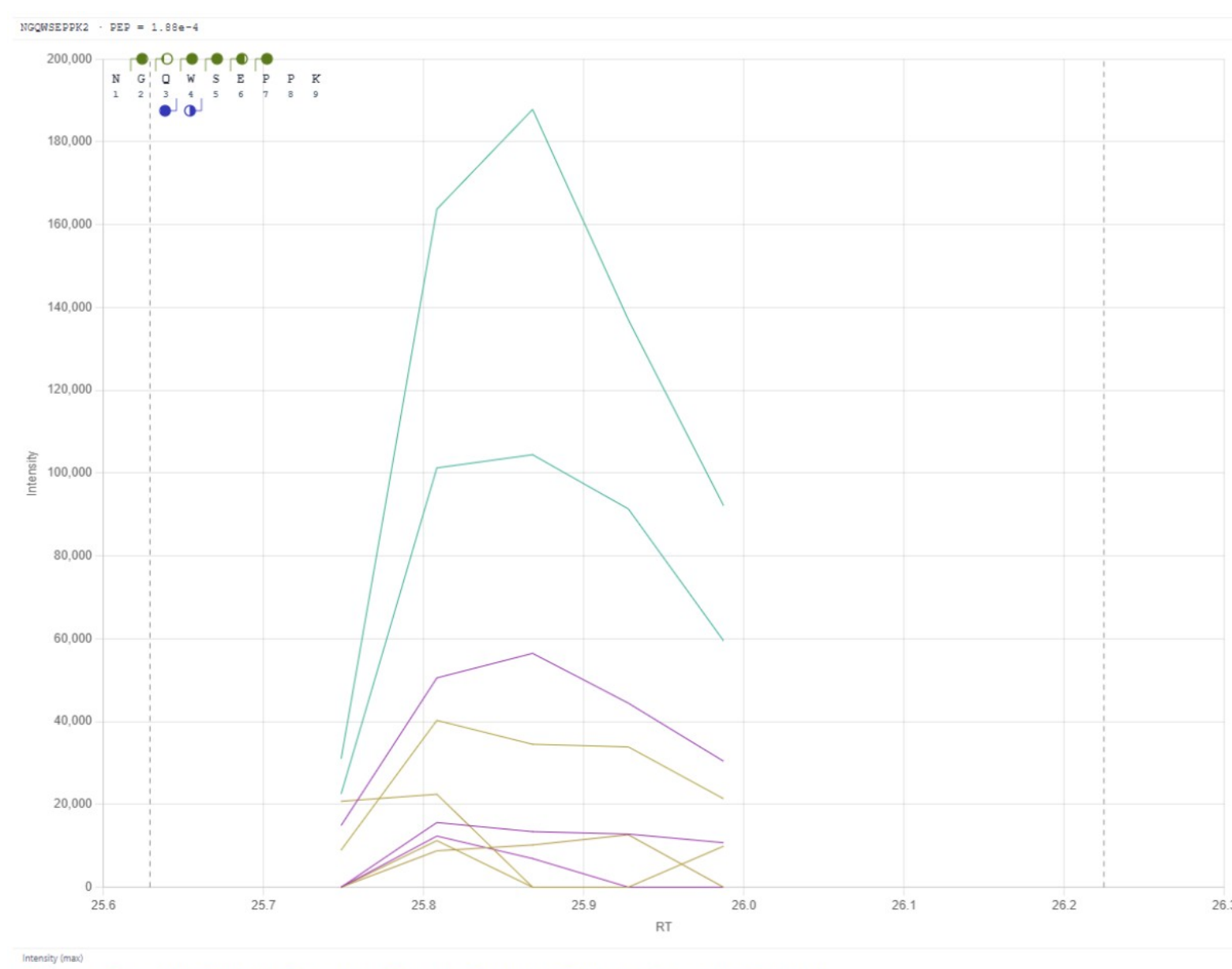

CFL1

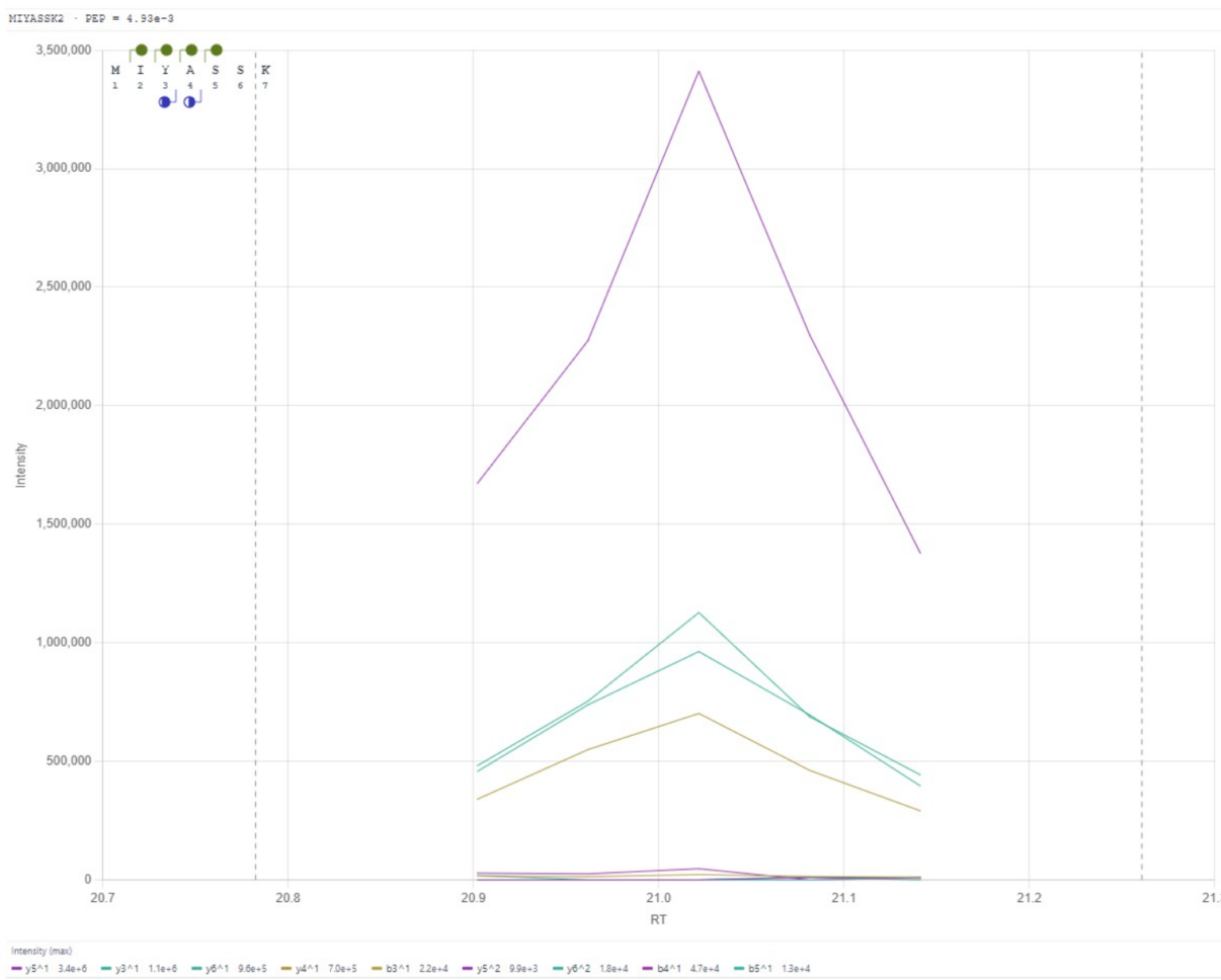

CHMP5

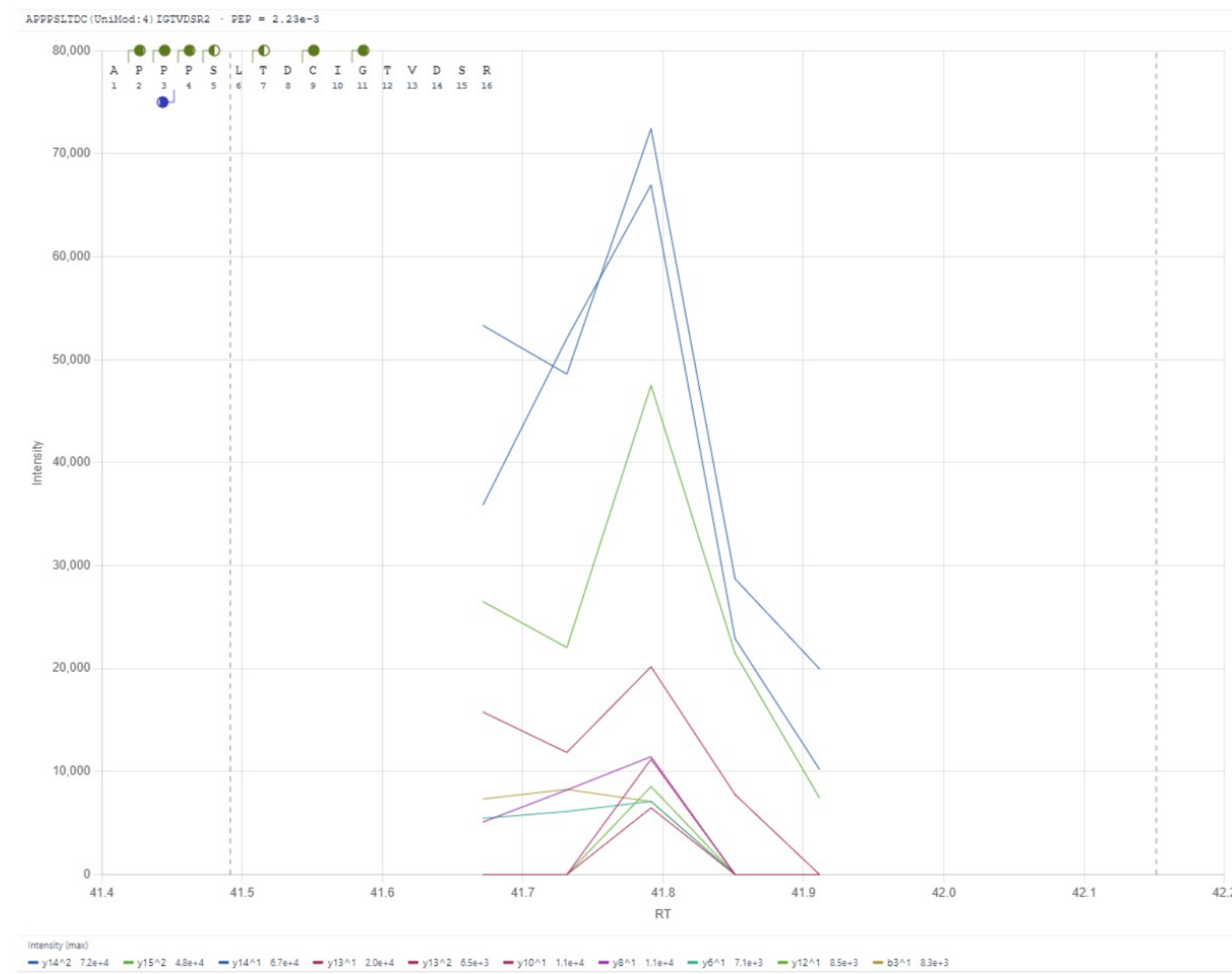

CLDN3

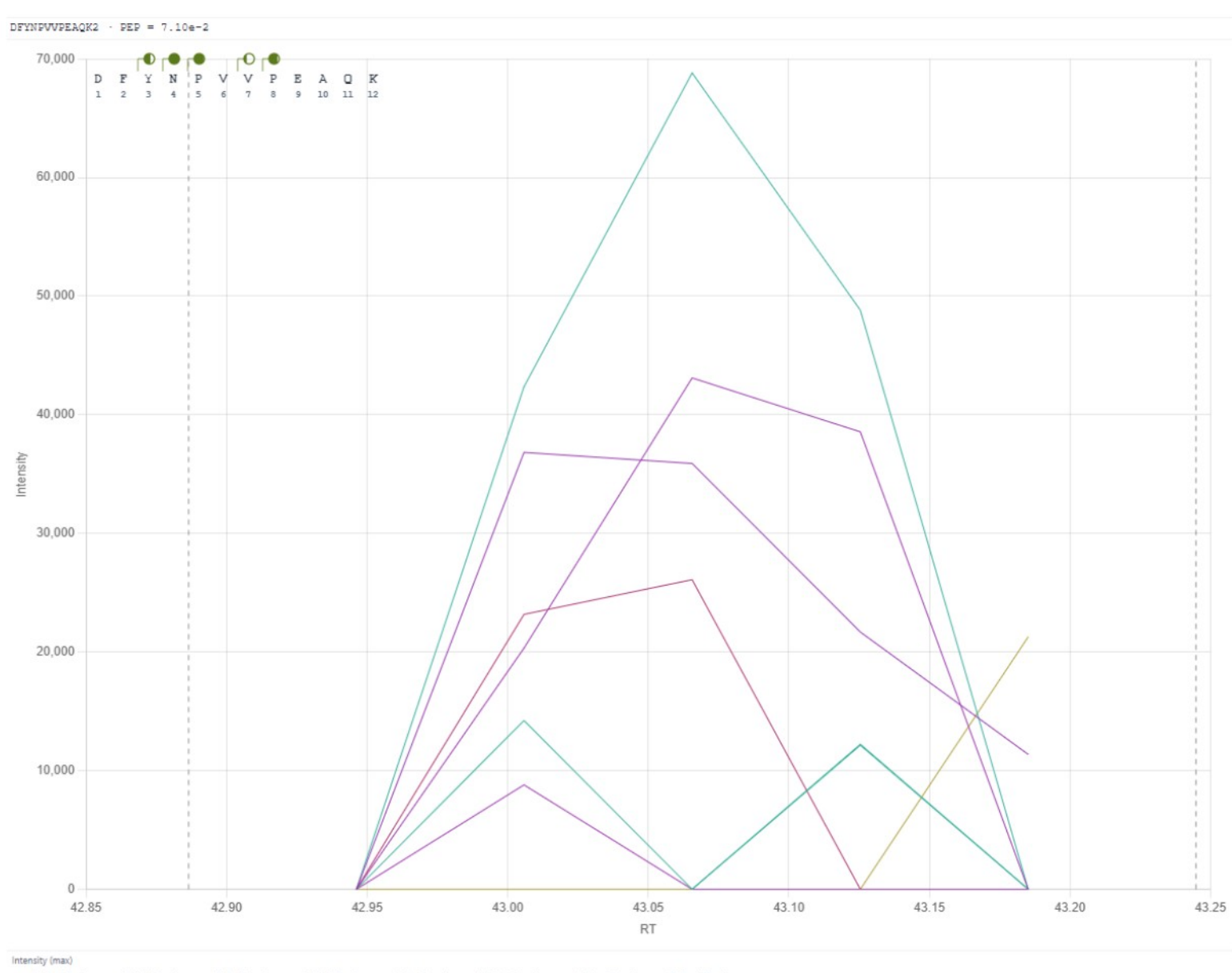

CNIH4

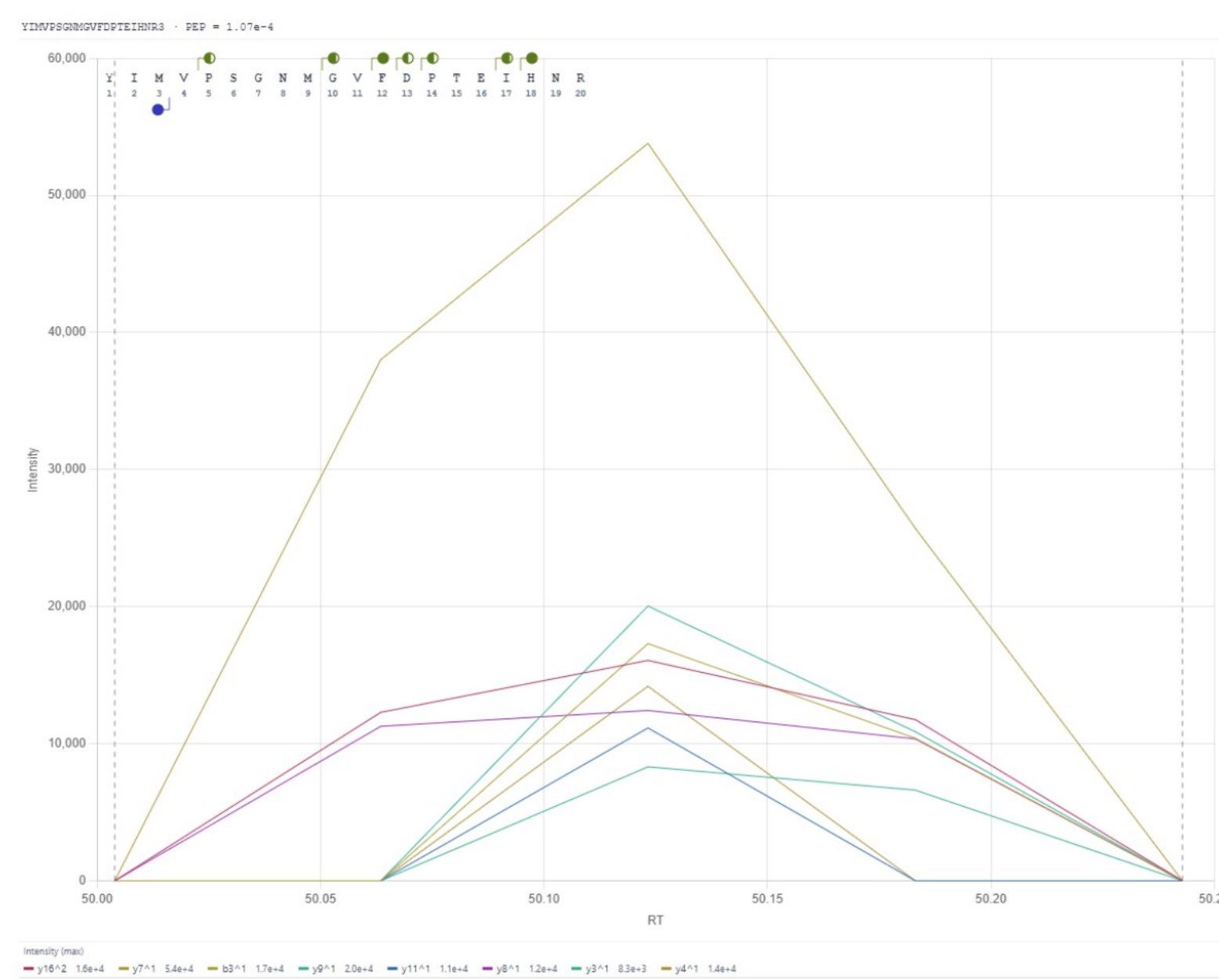

COG6

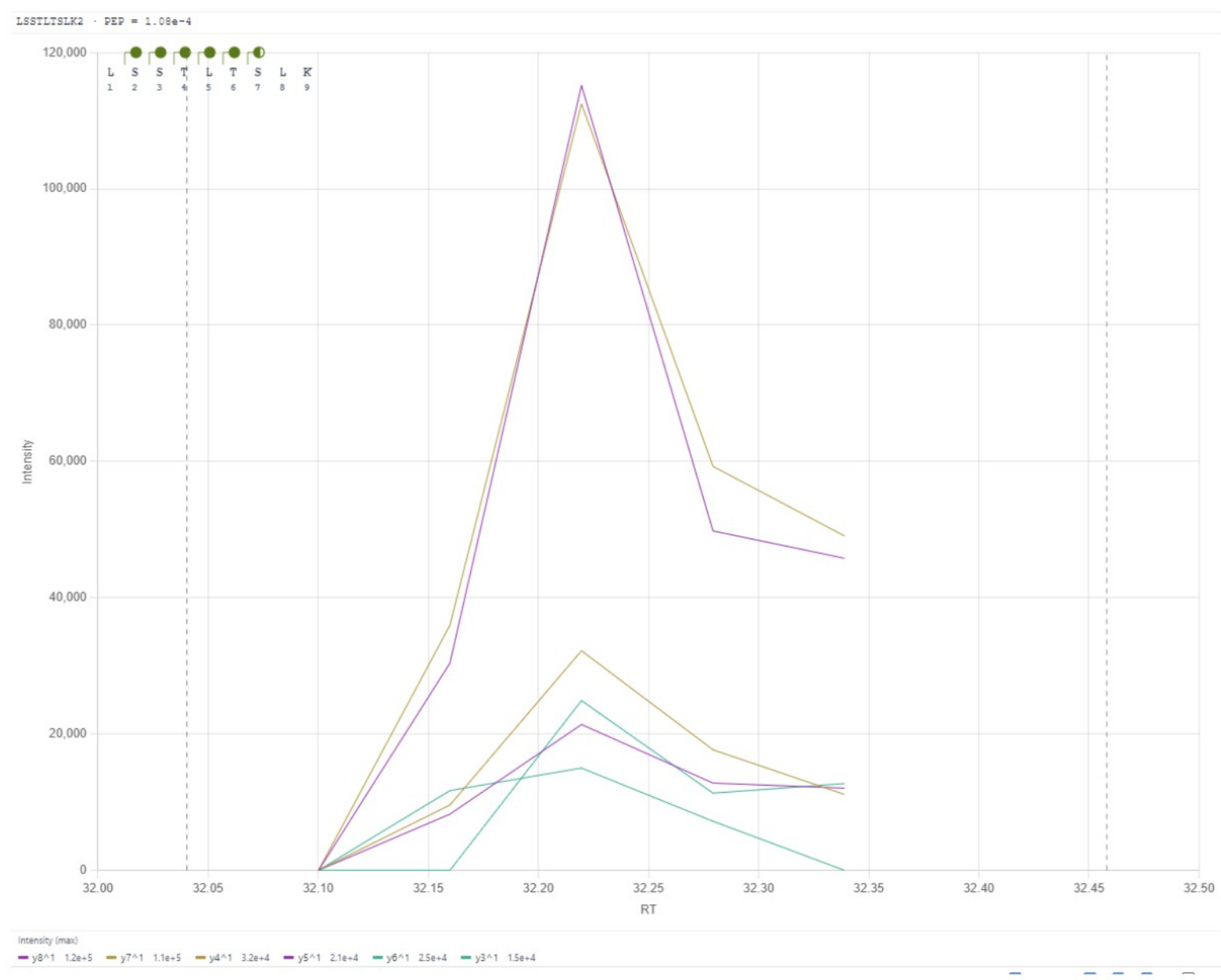

CORO1A

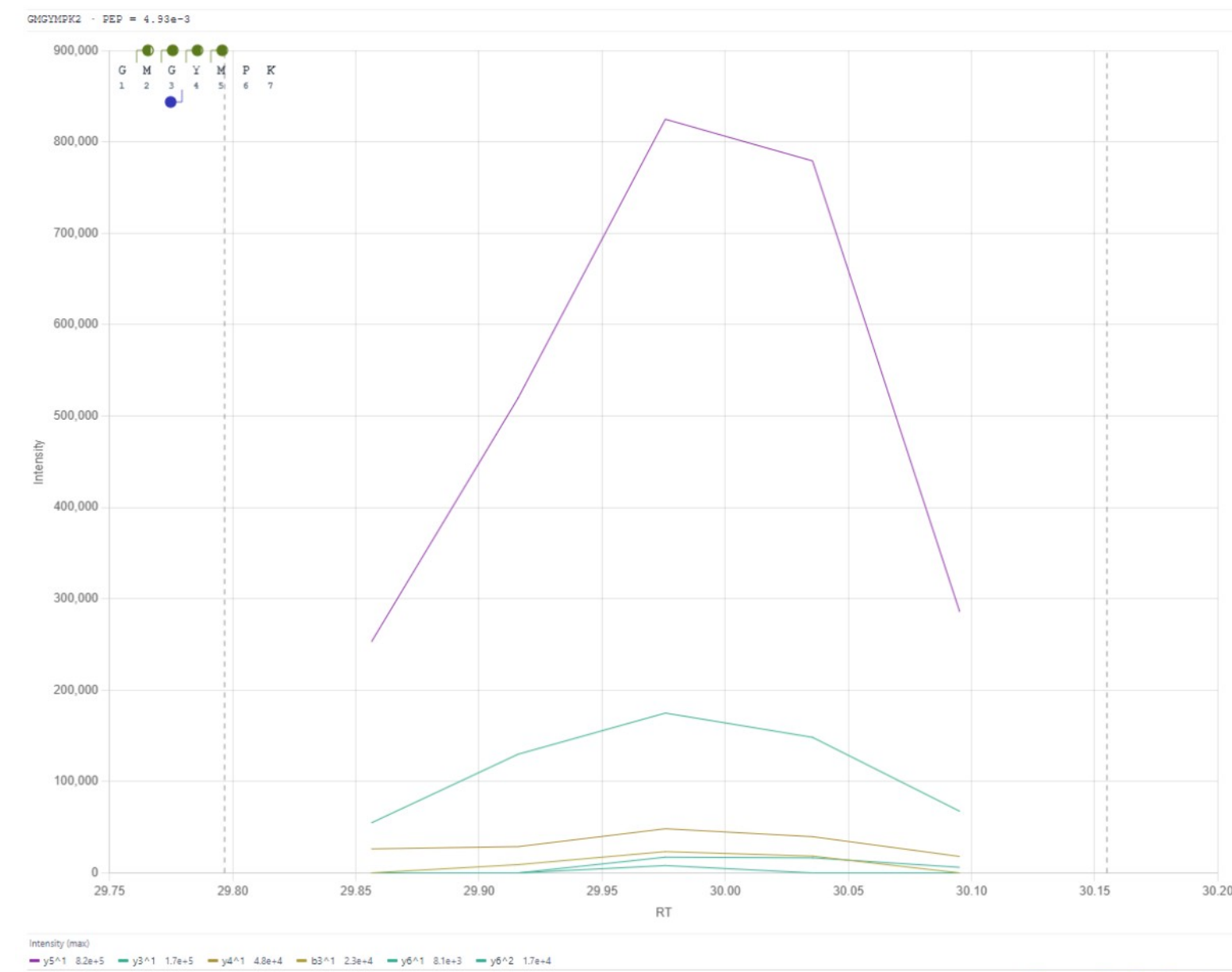

CPNE2

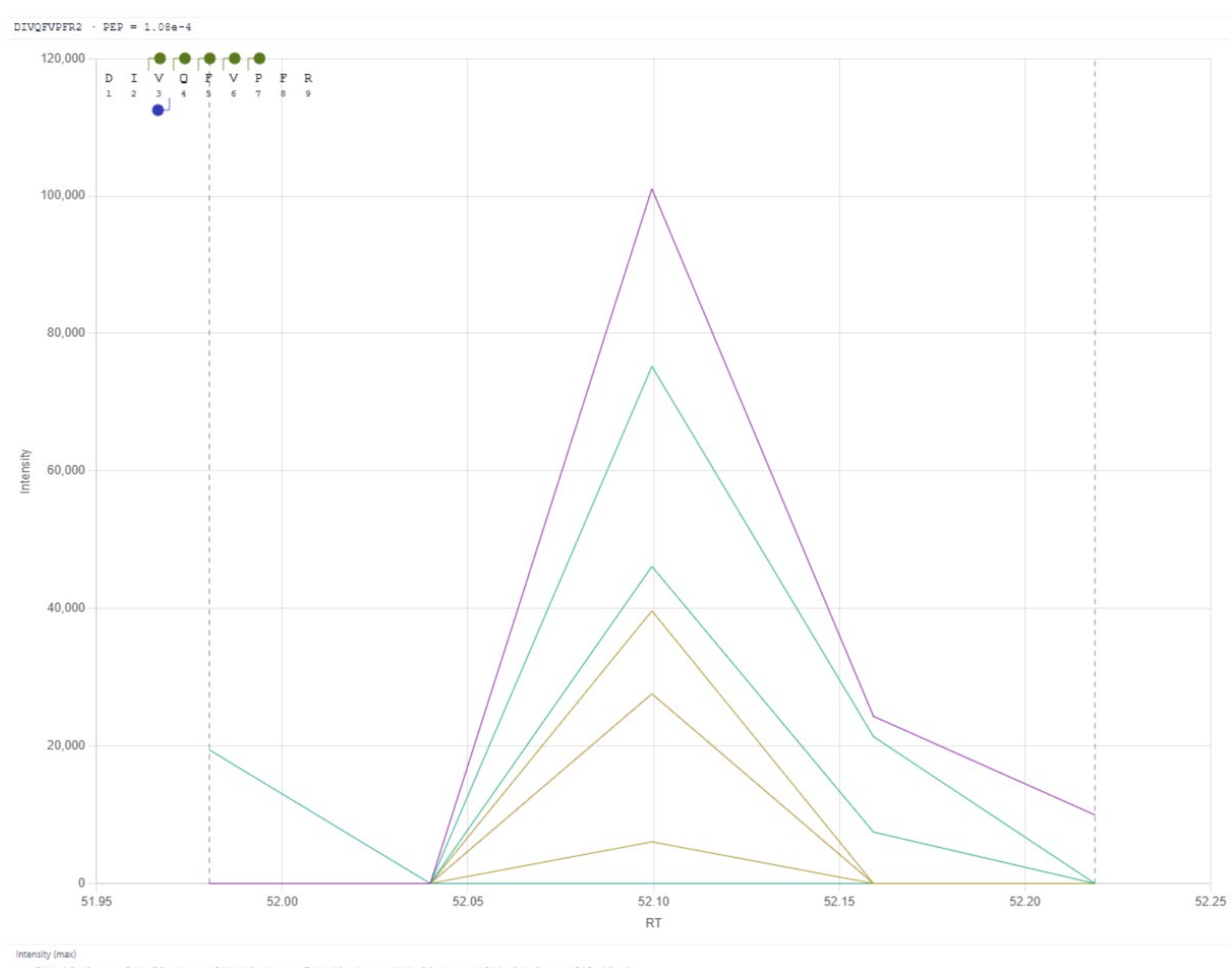

CXCL3

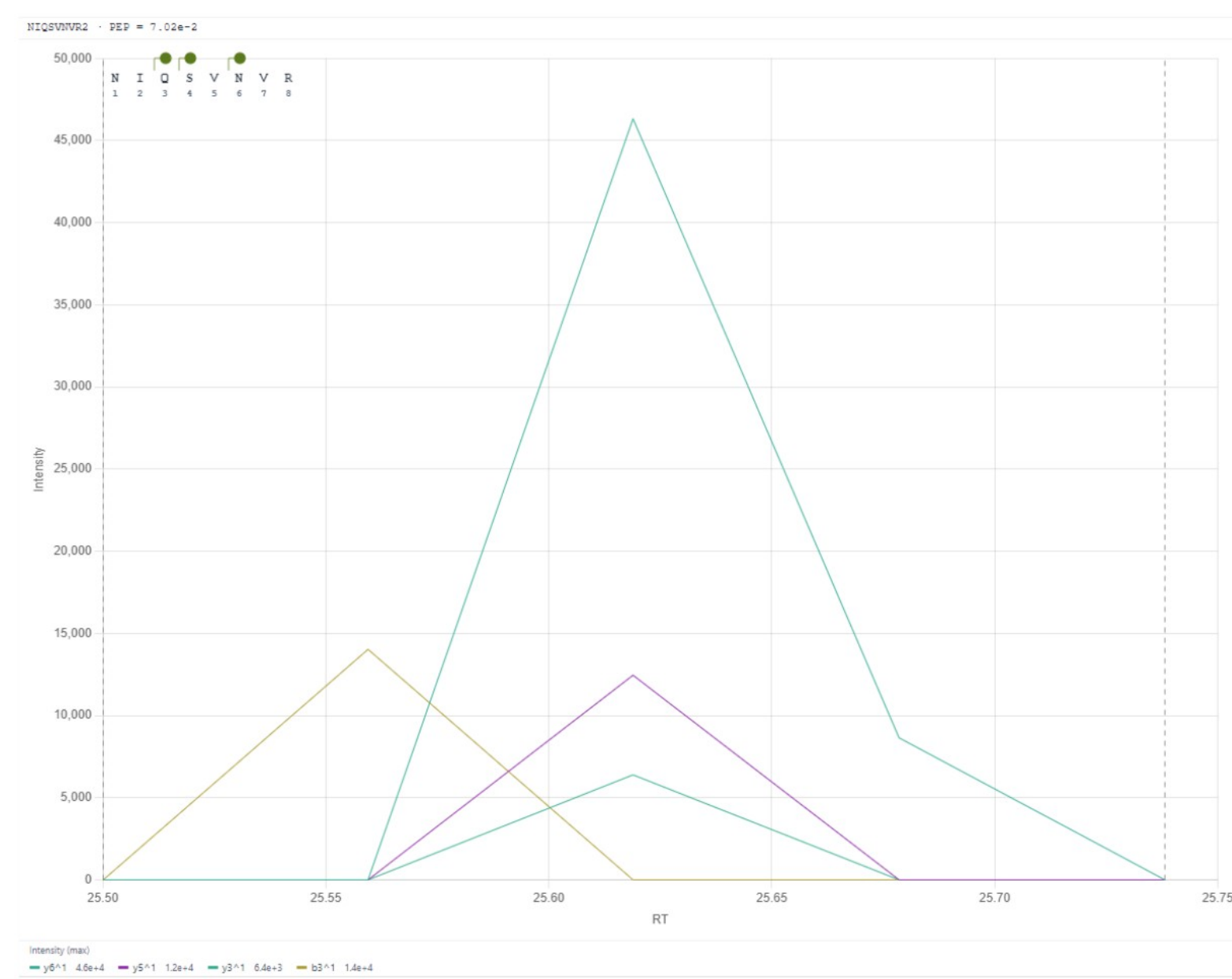

CYB5A

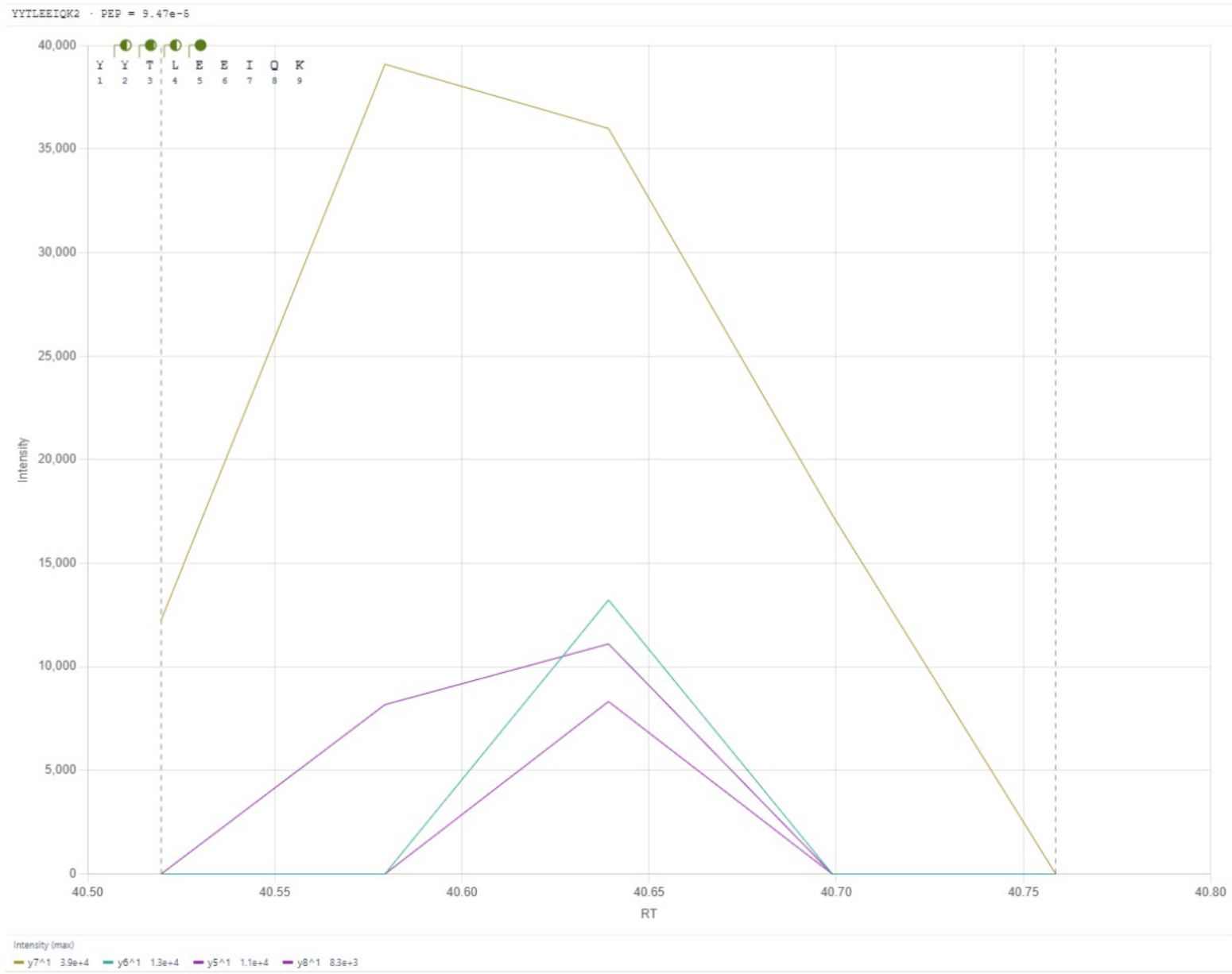

DAGLB

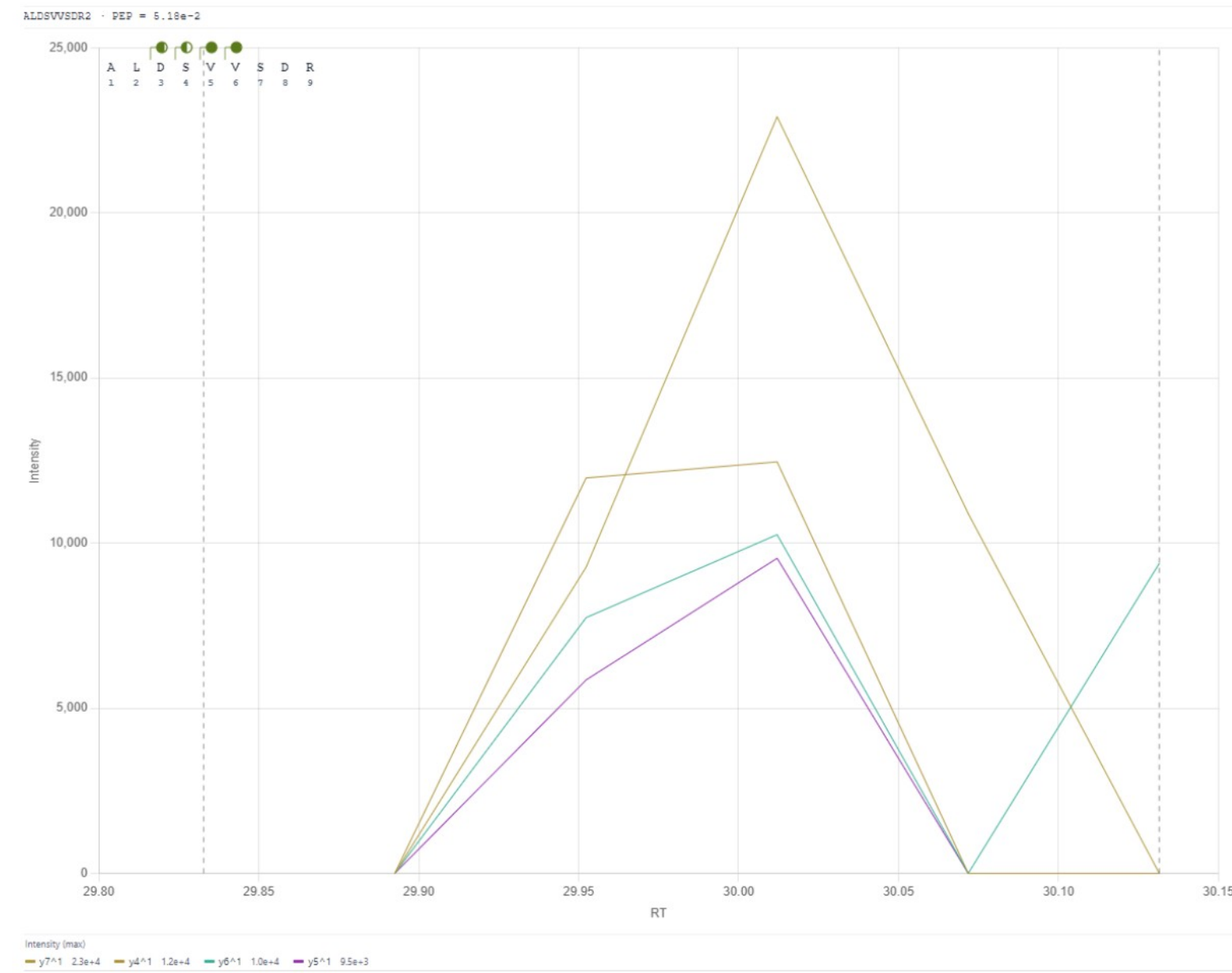

DCTN5

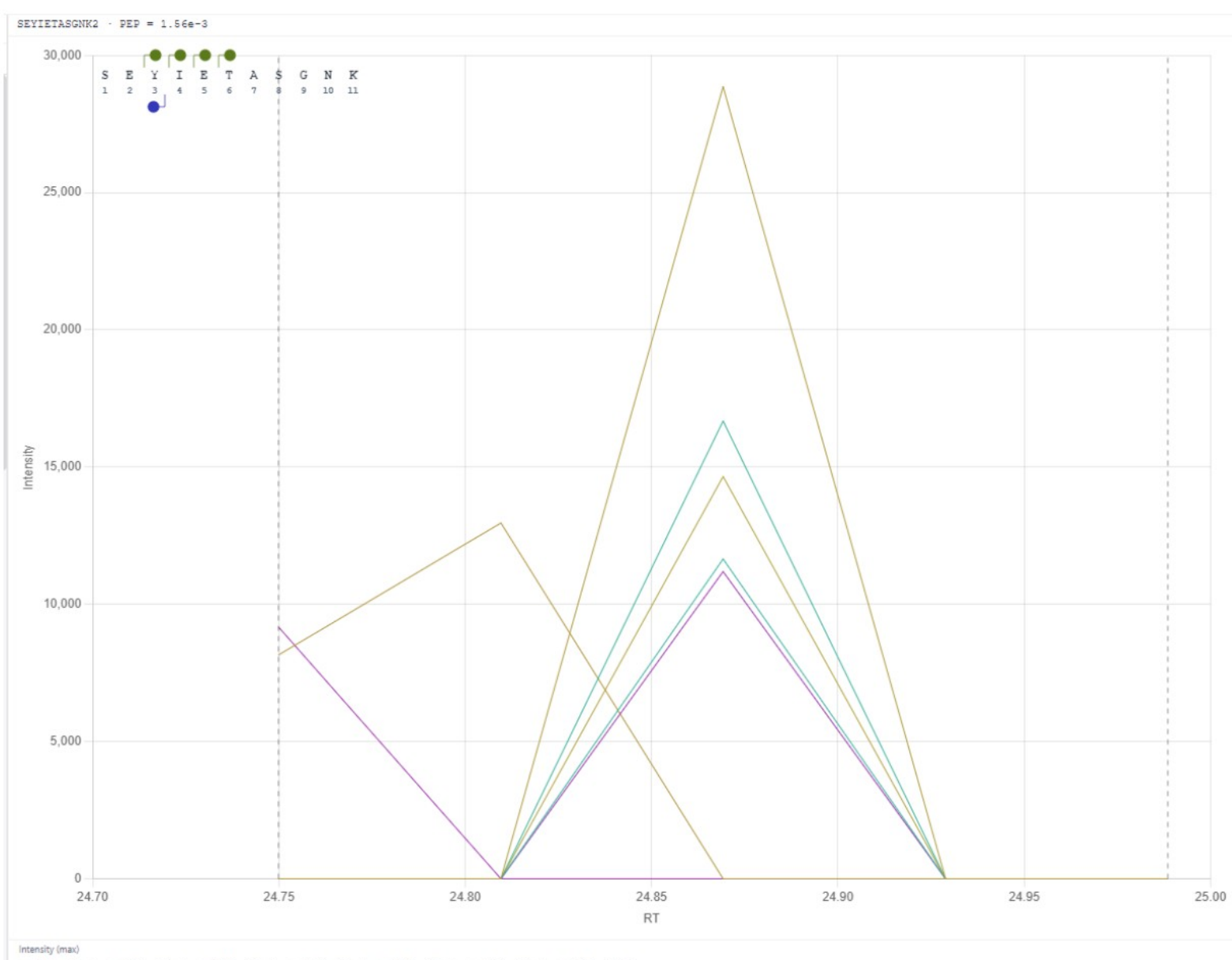

DENND4A

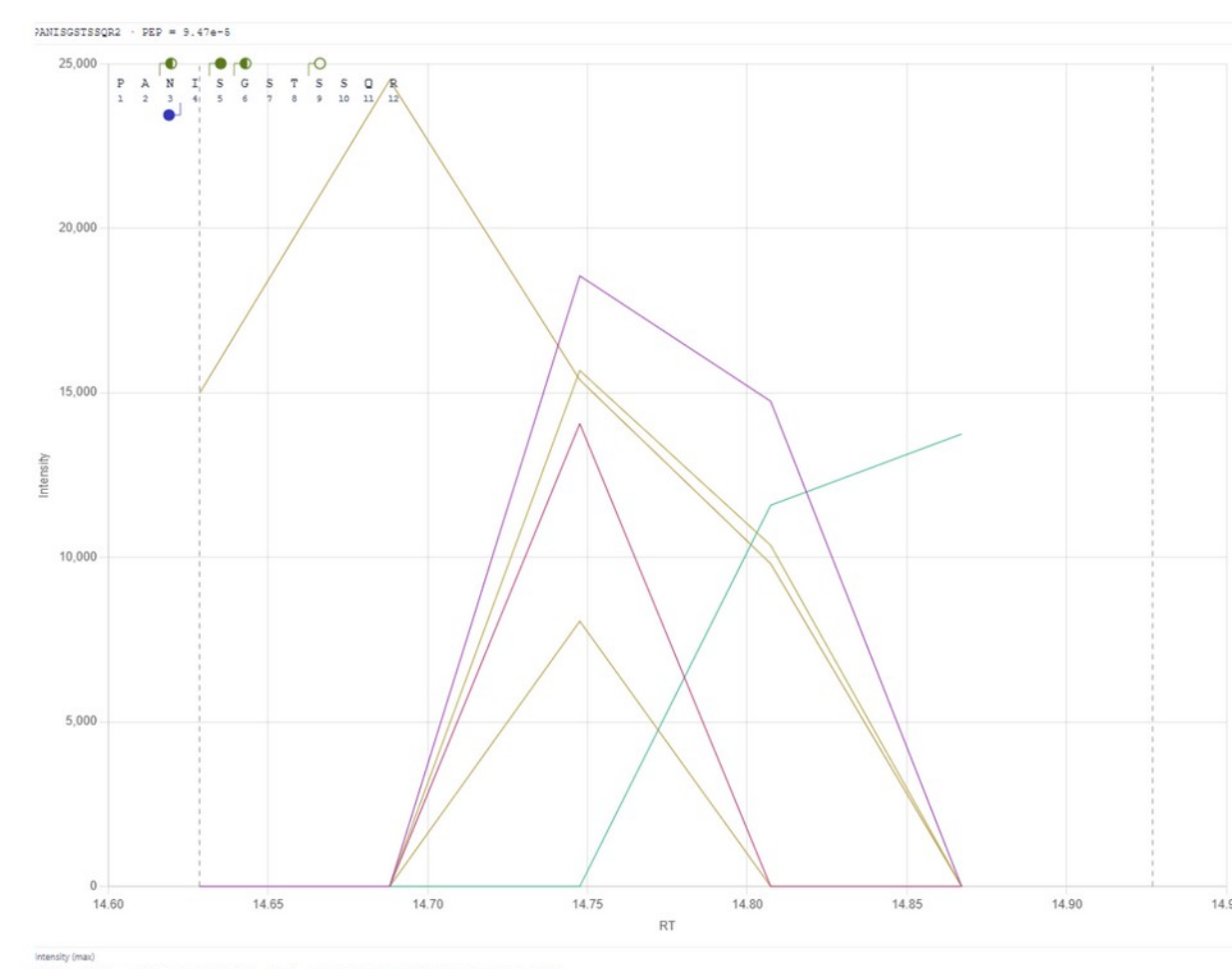

DIPK2A

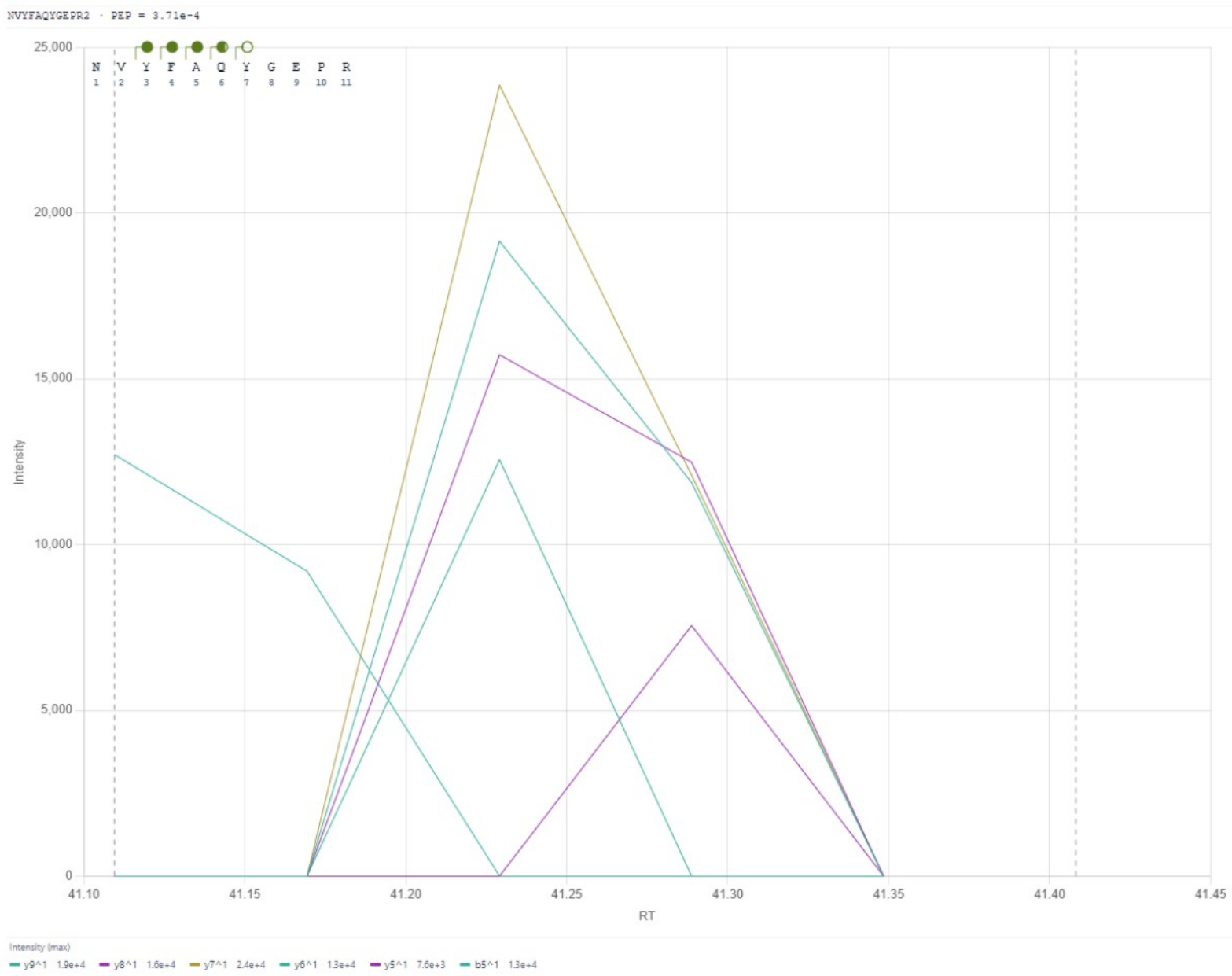

DNM2

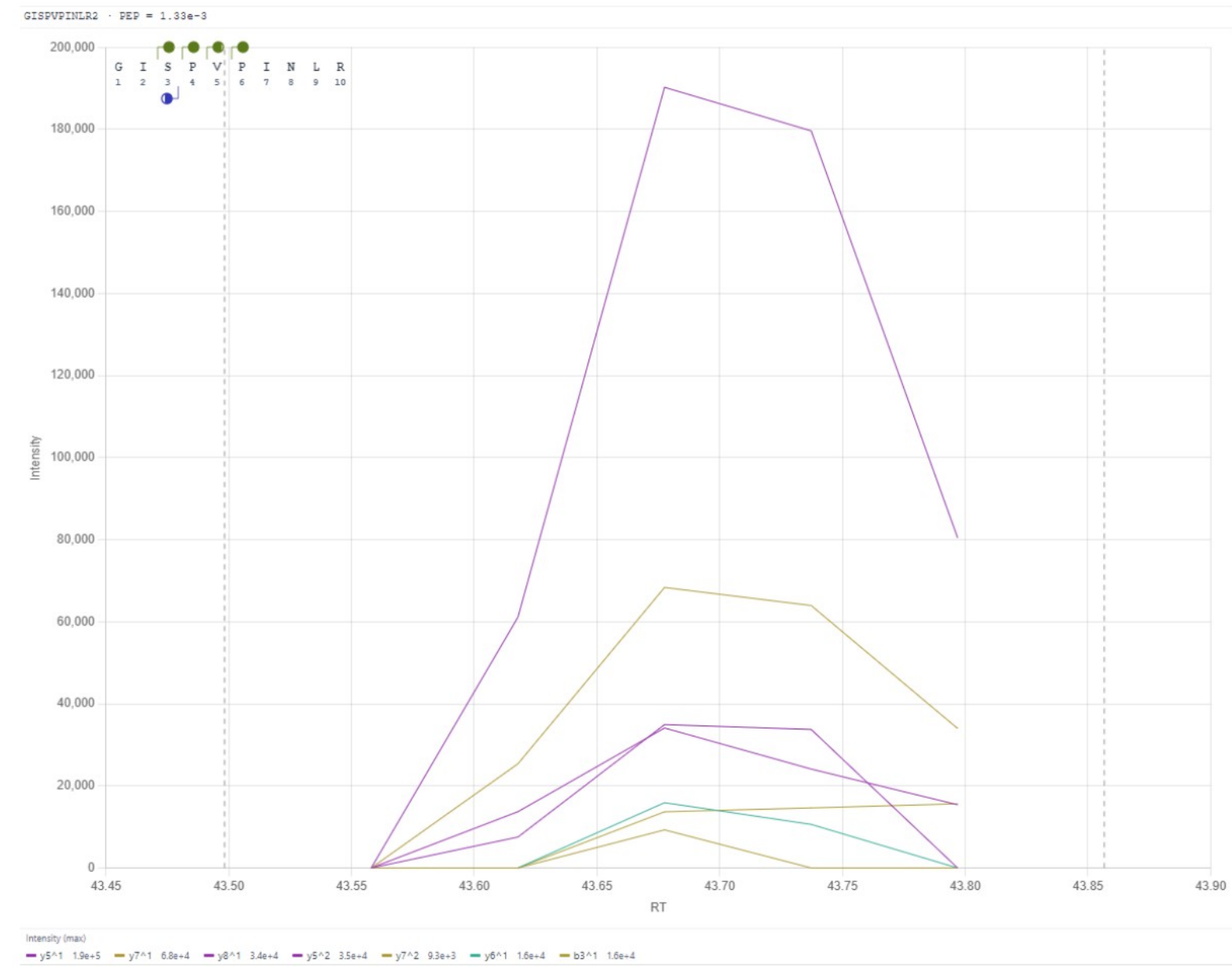

DNPH1

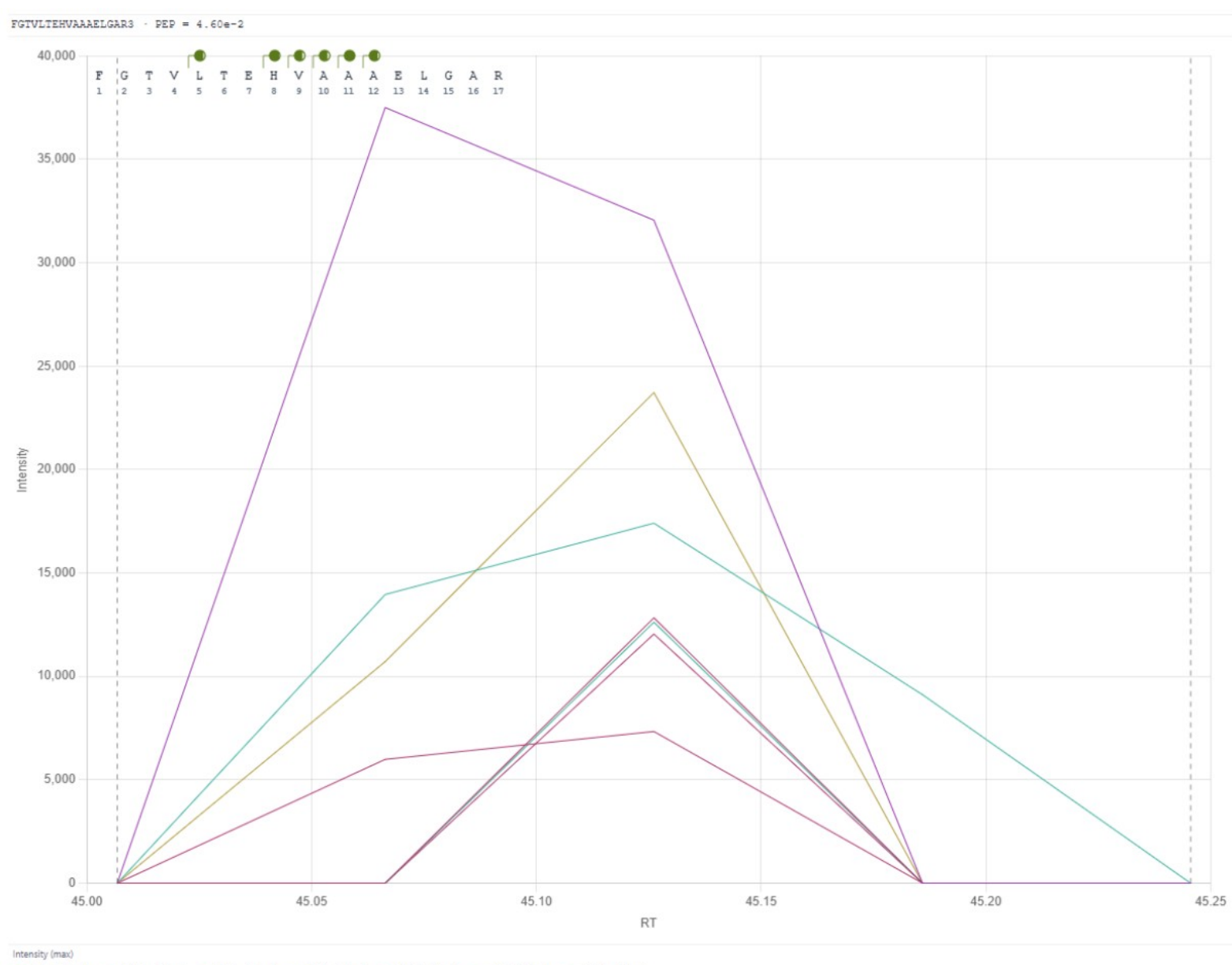

DOCK3

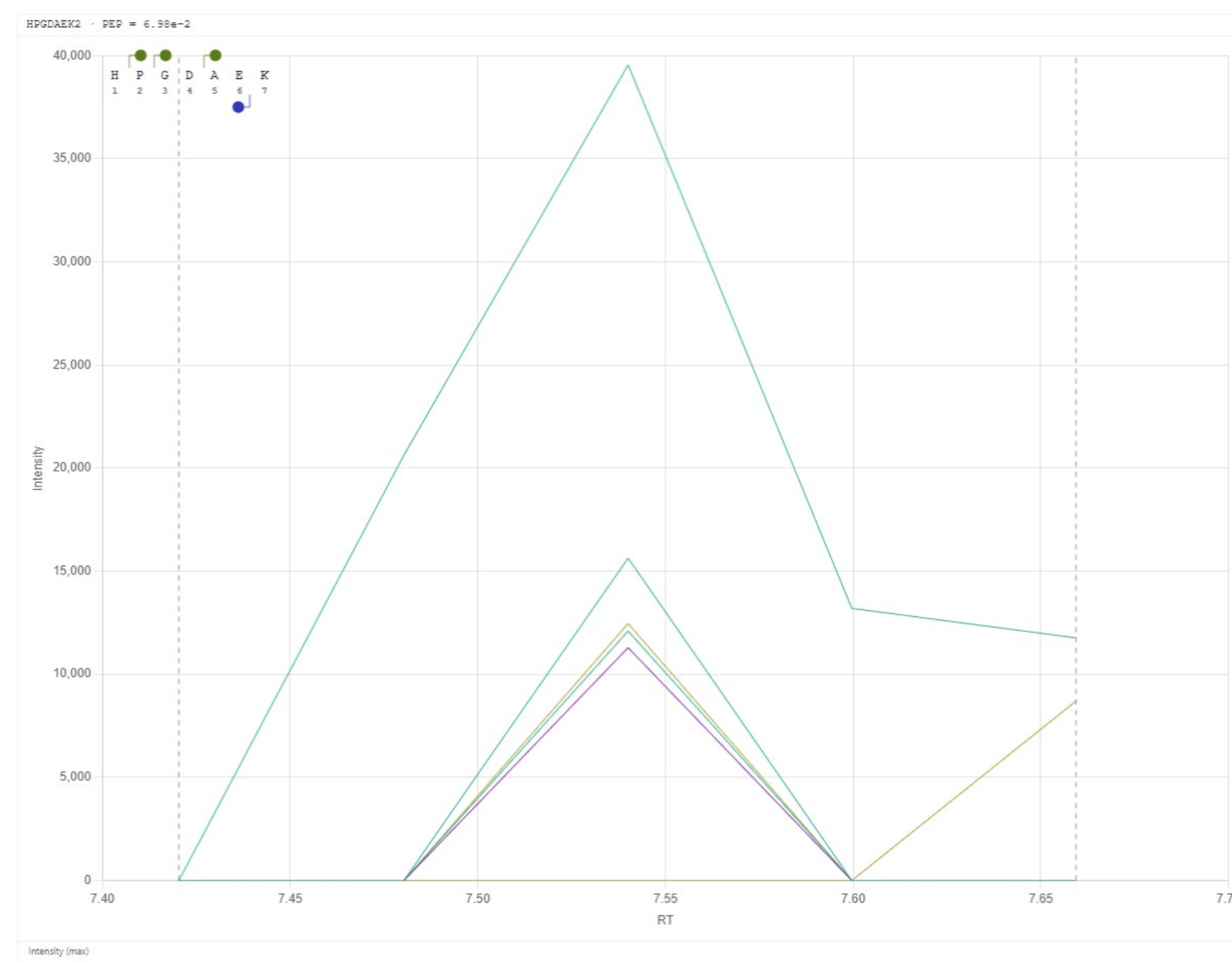

DOCK8

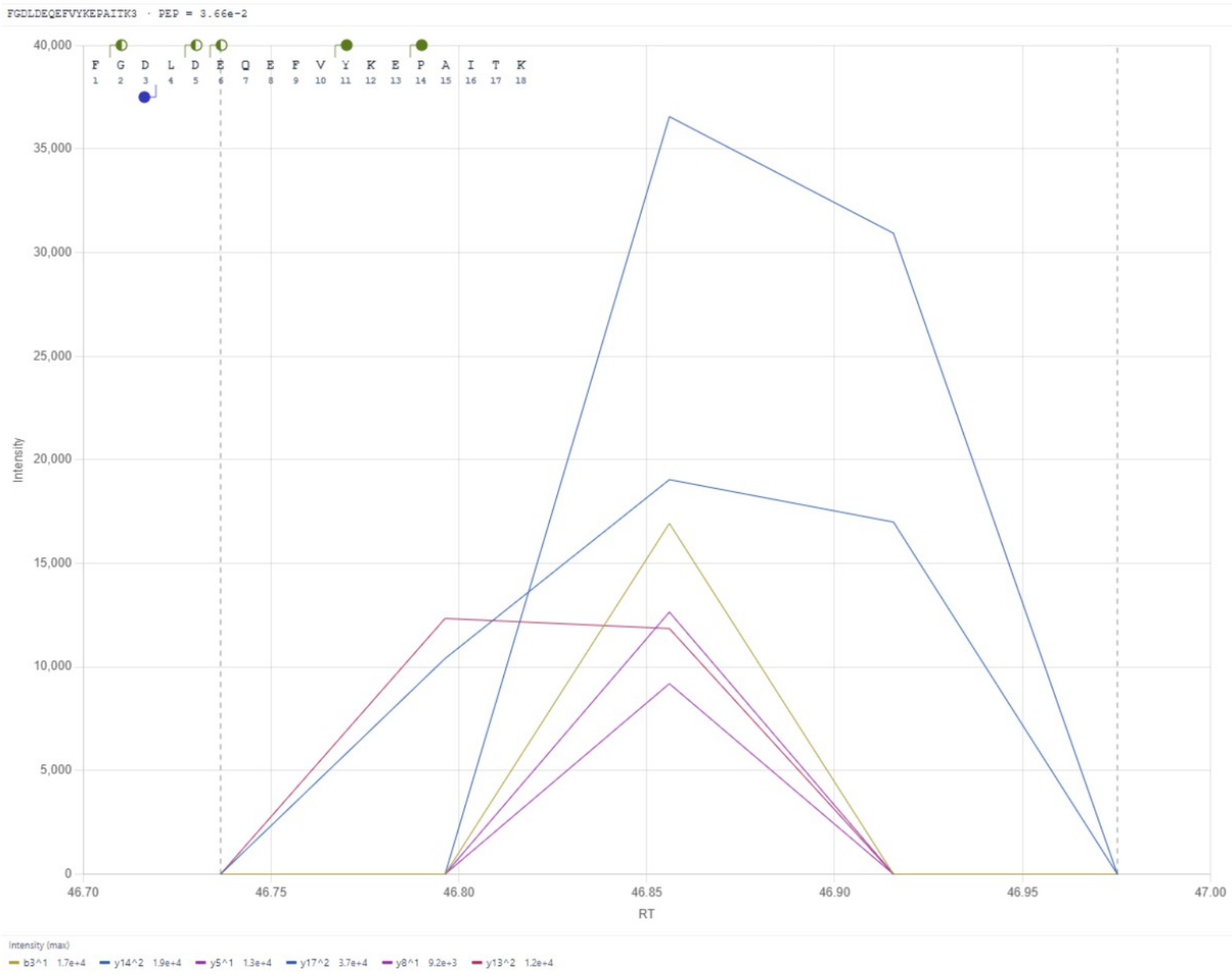

EEF1B2

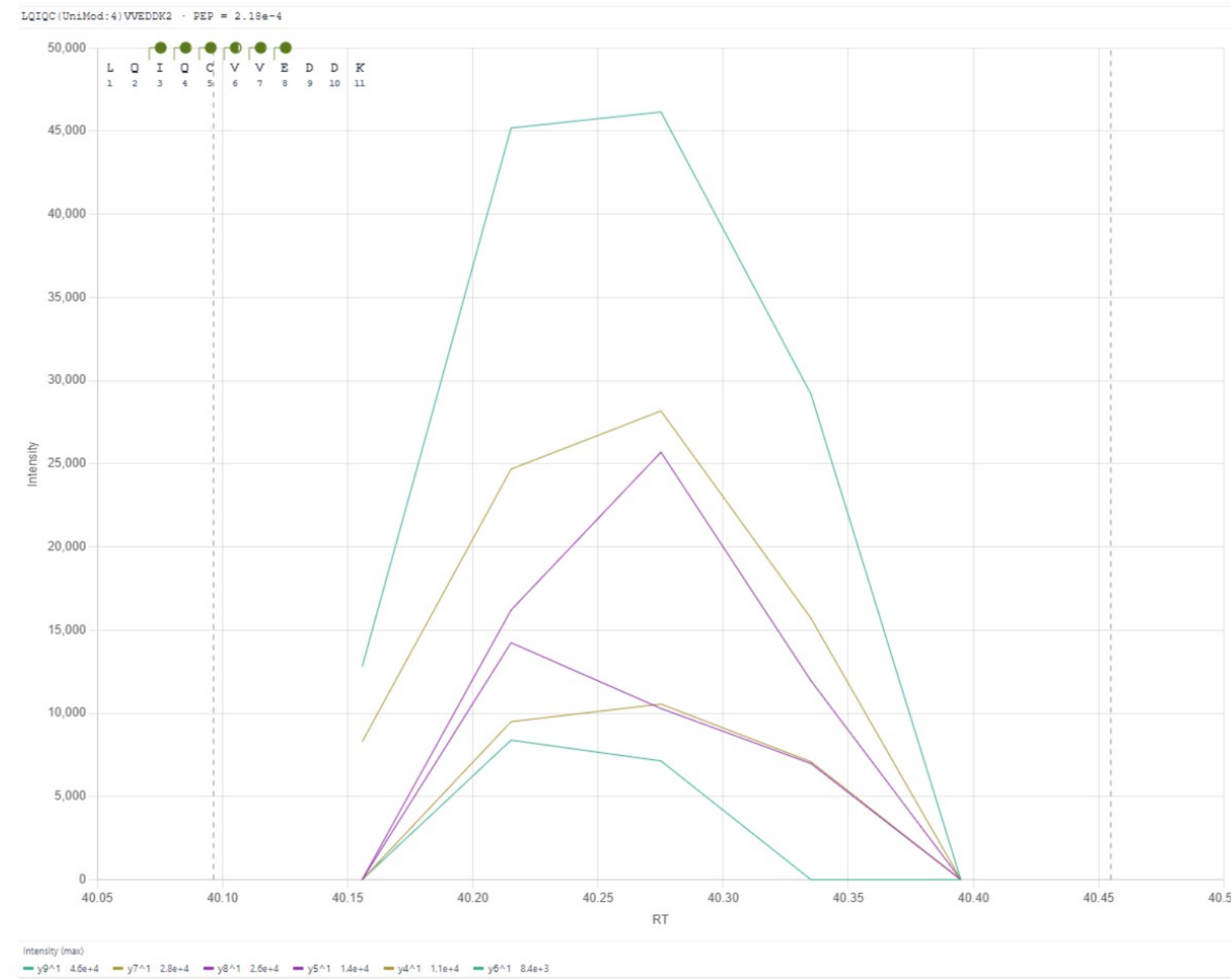

ELOVL7

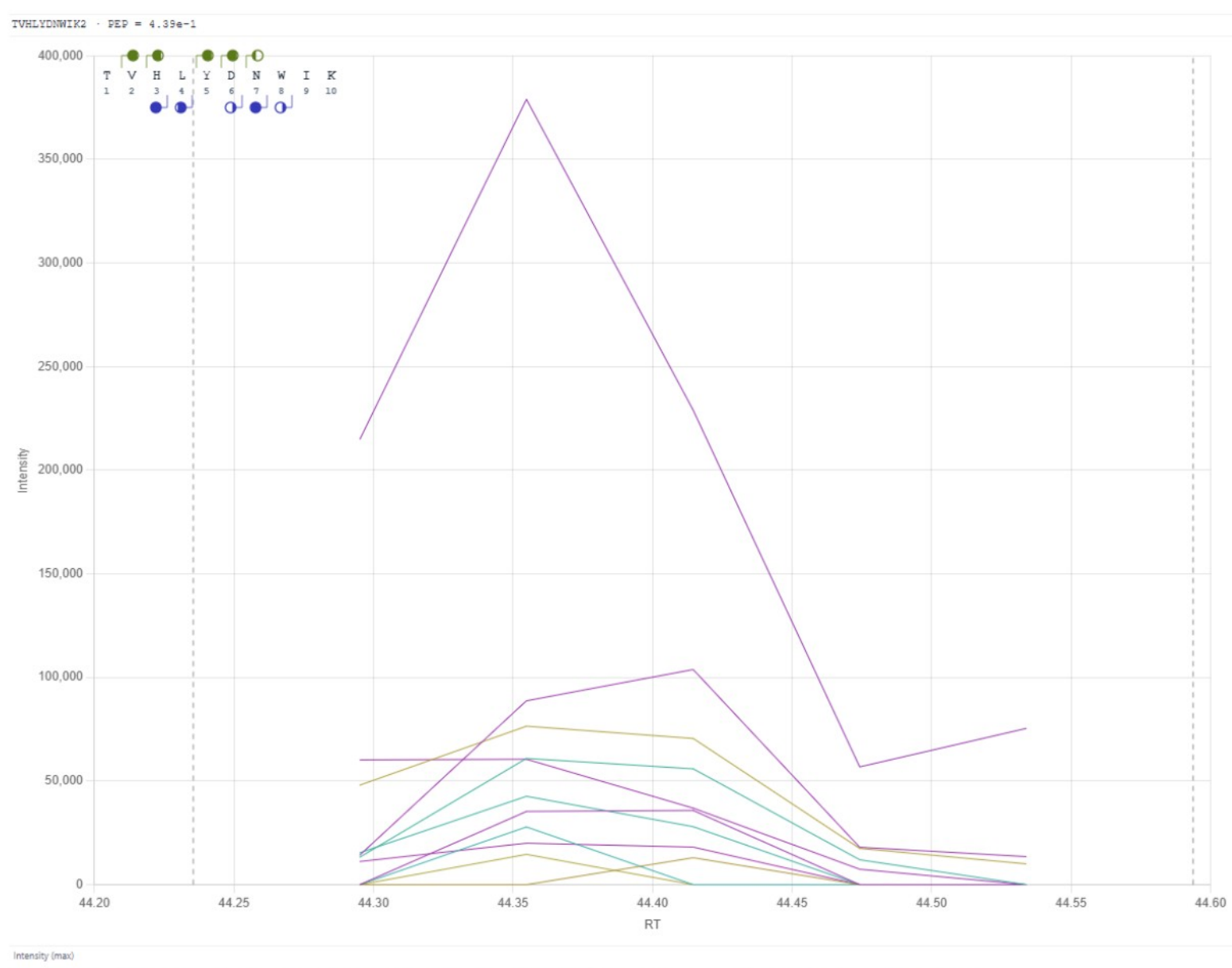

EMC3

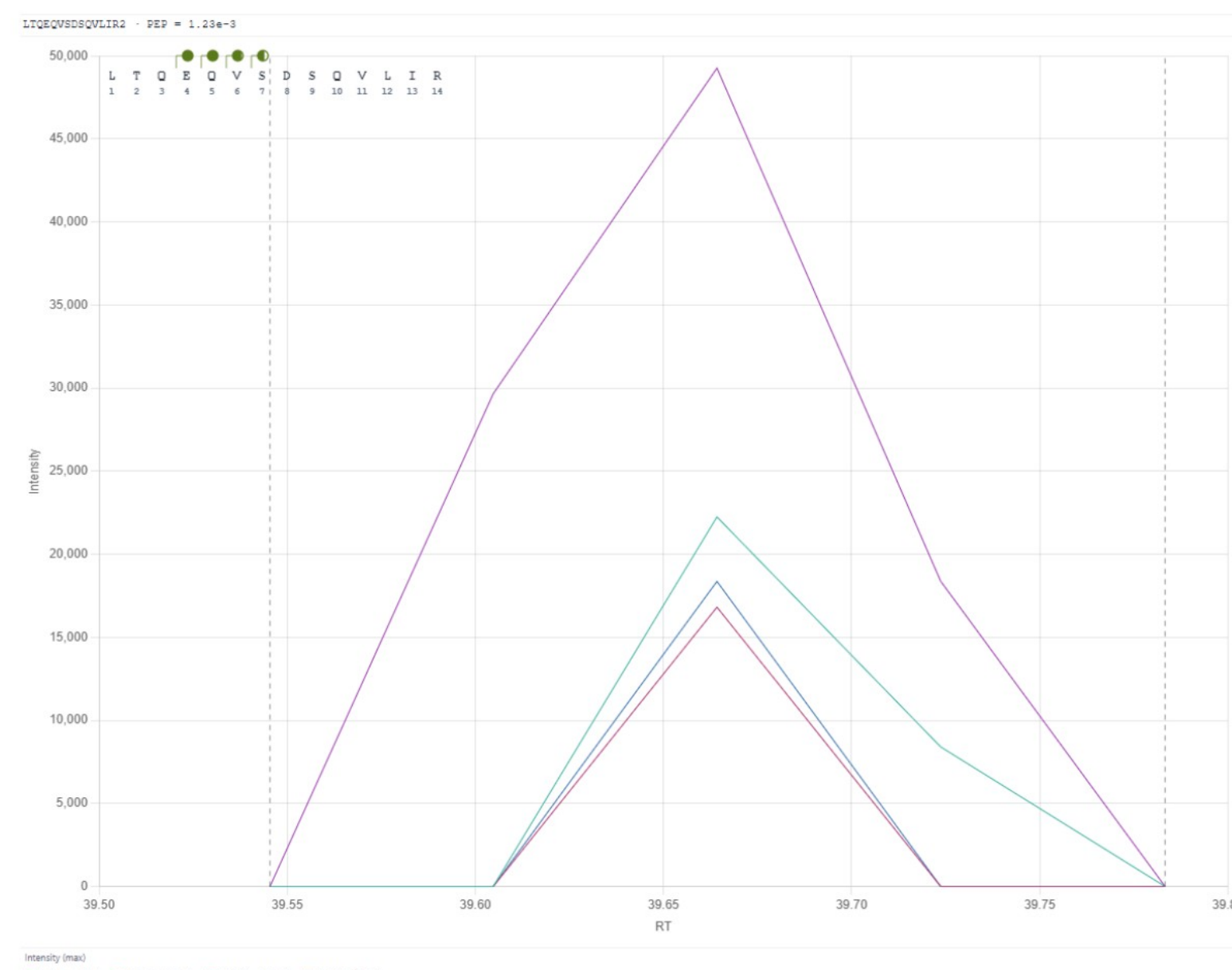

EPB41L3

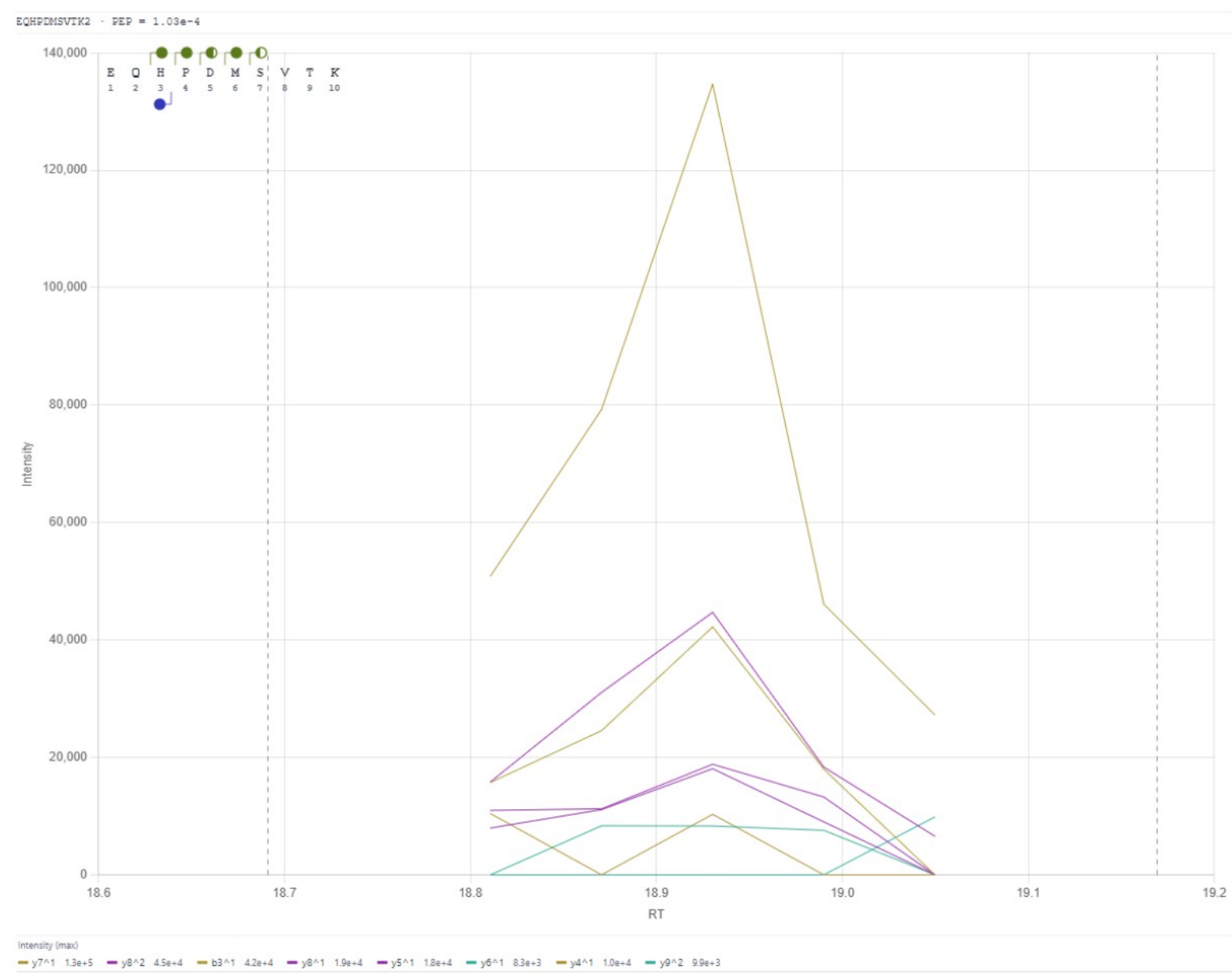

EPB42

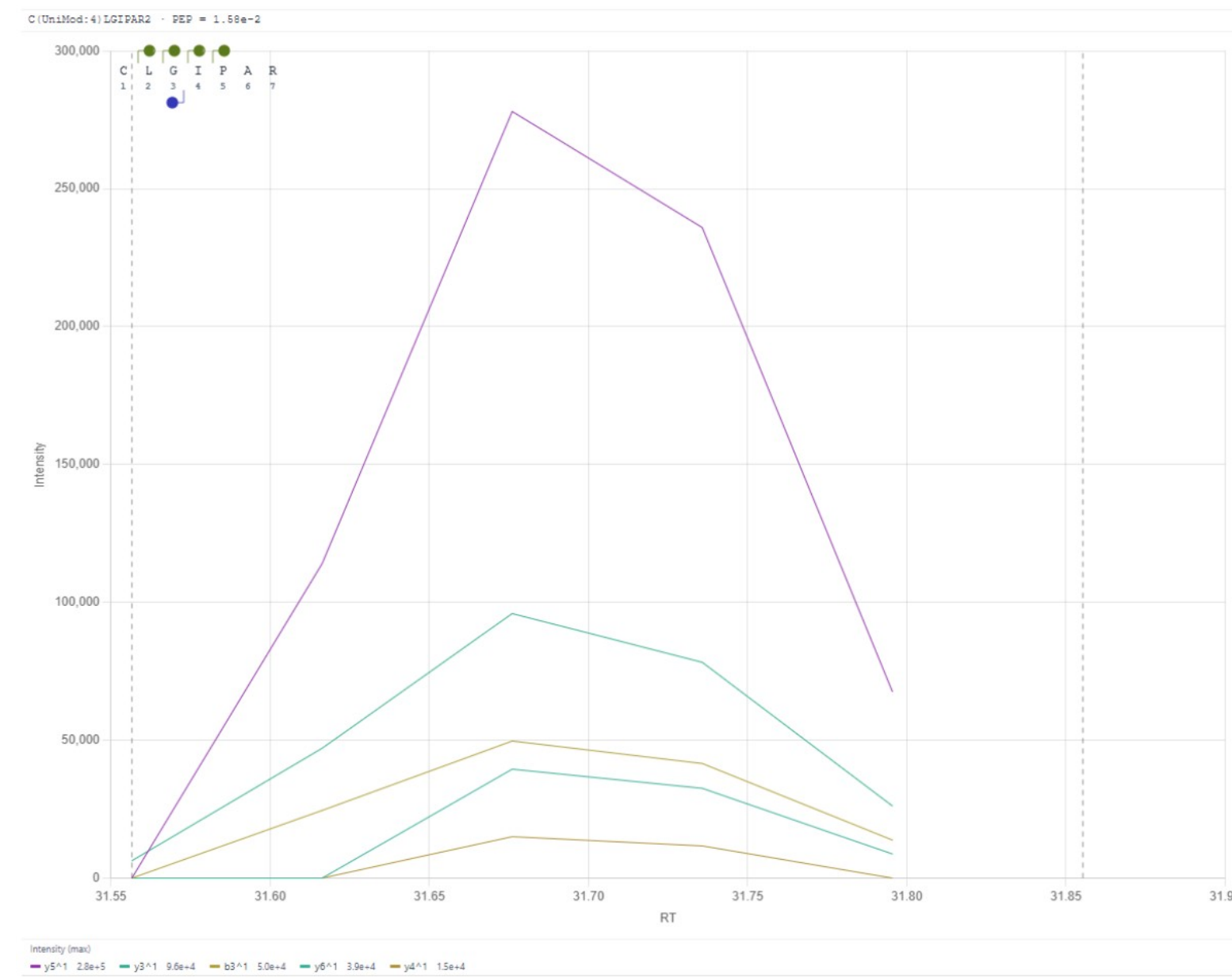

ERFL

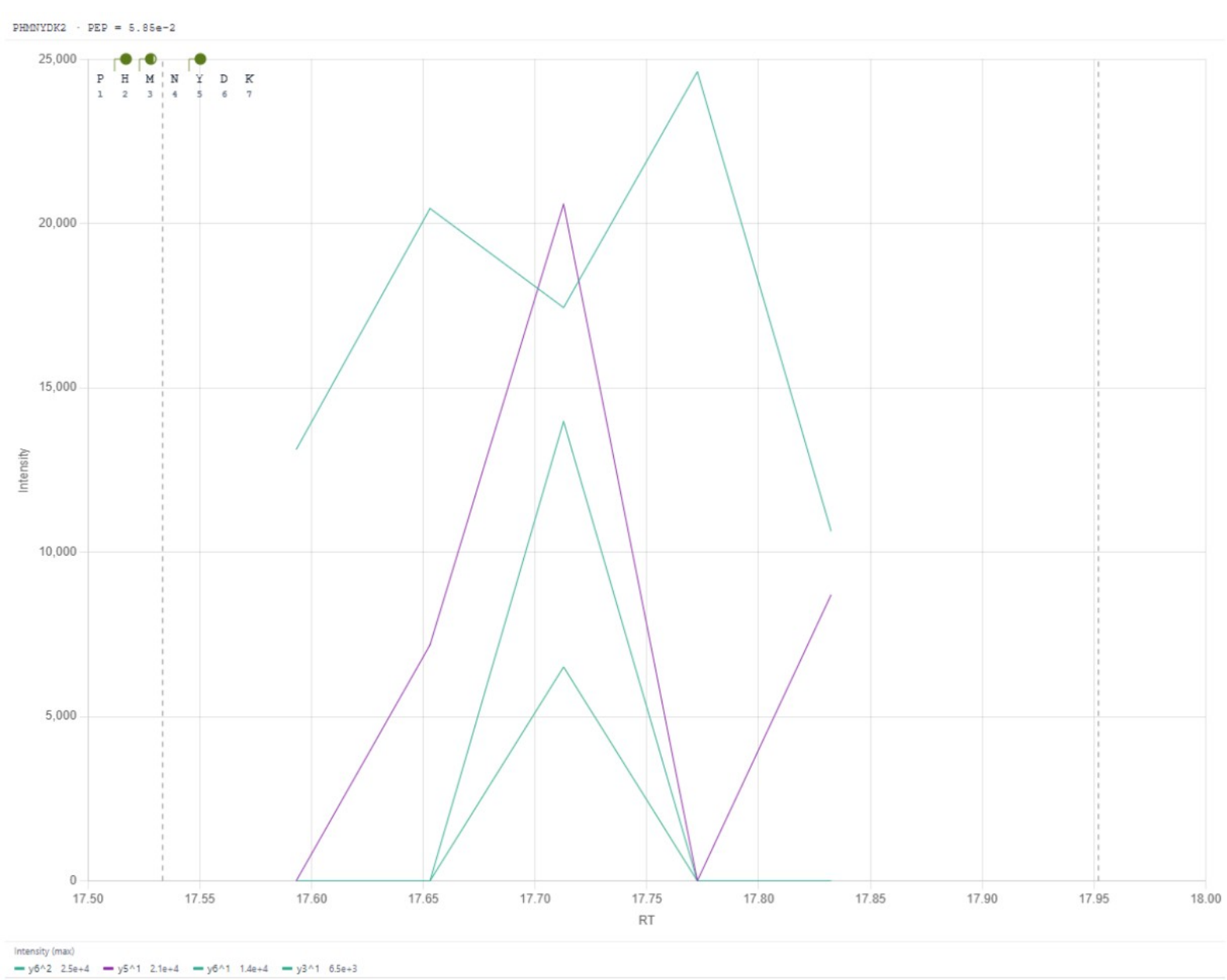

F2R

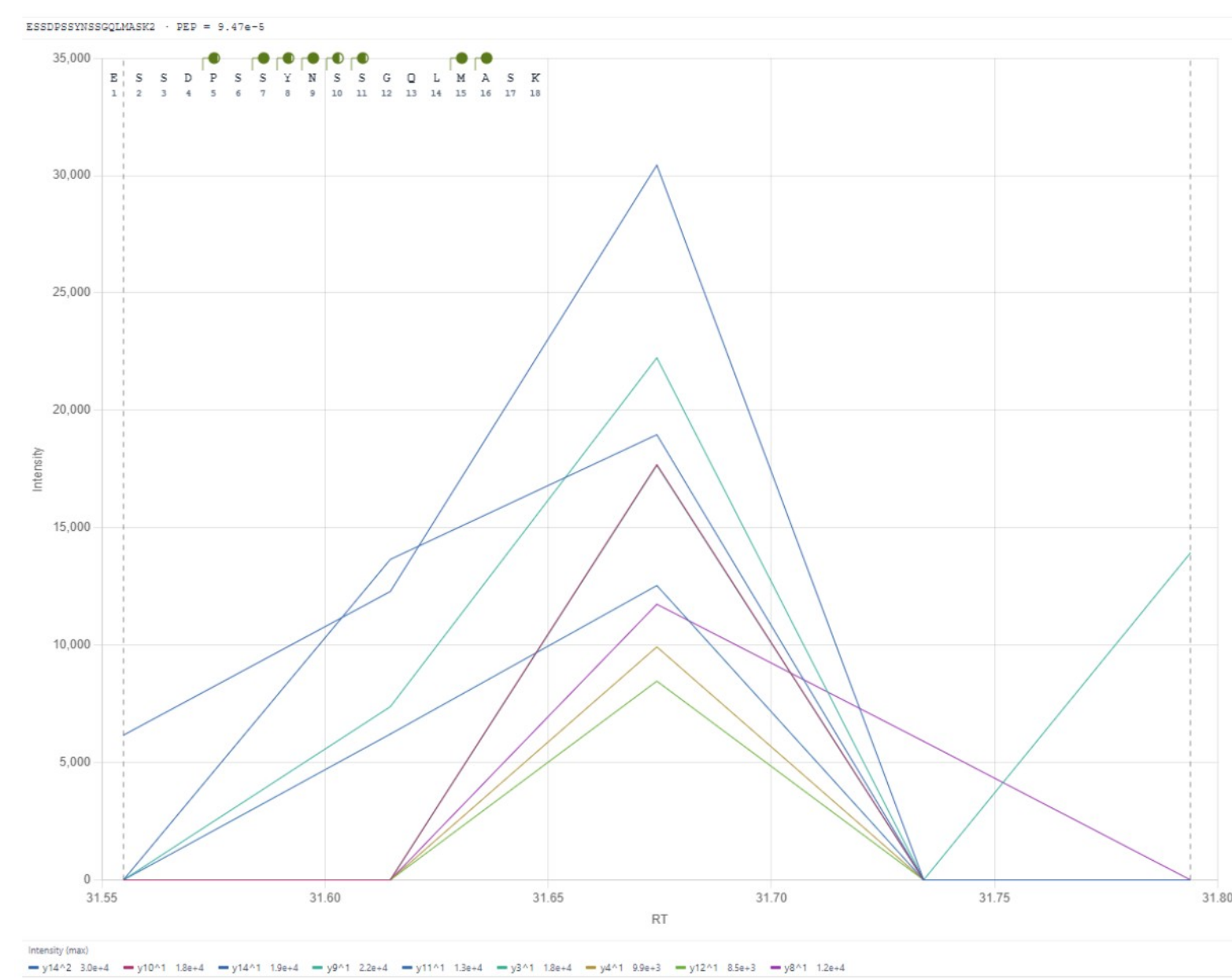

F2RL3

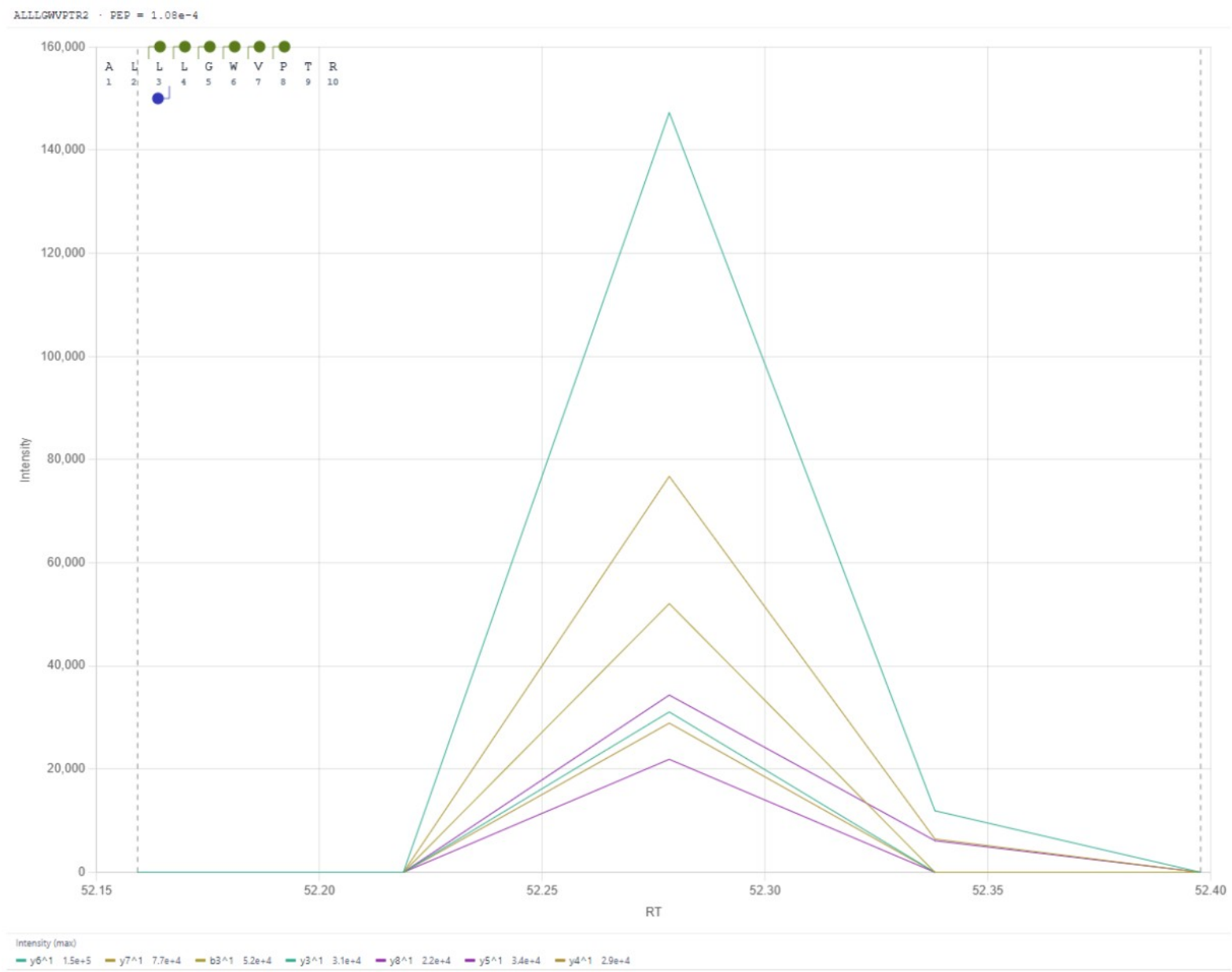

FES

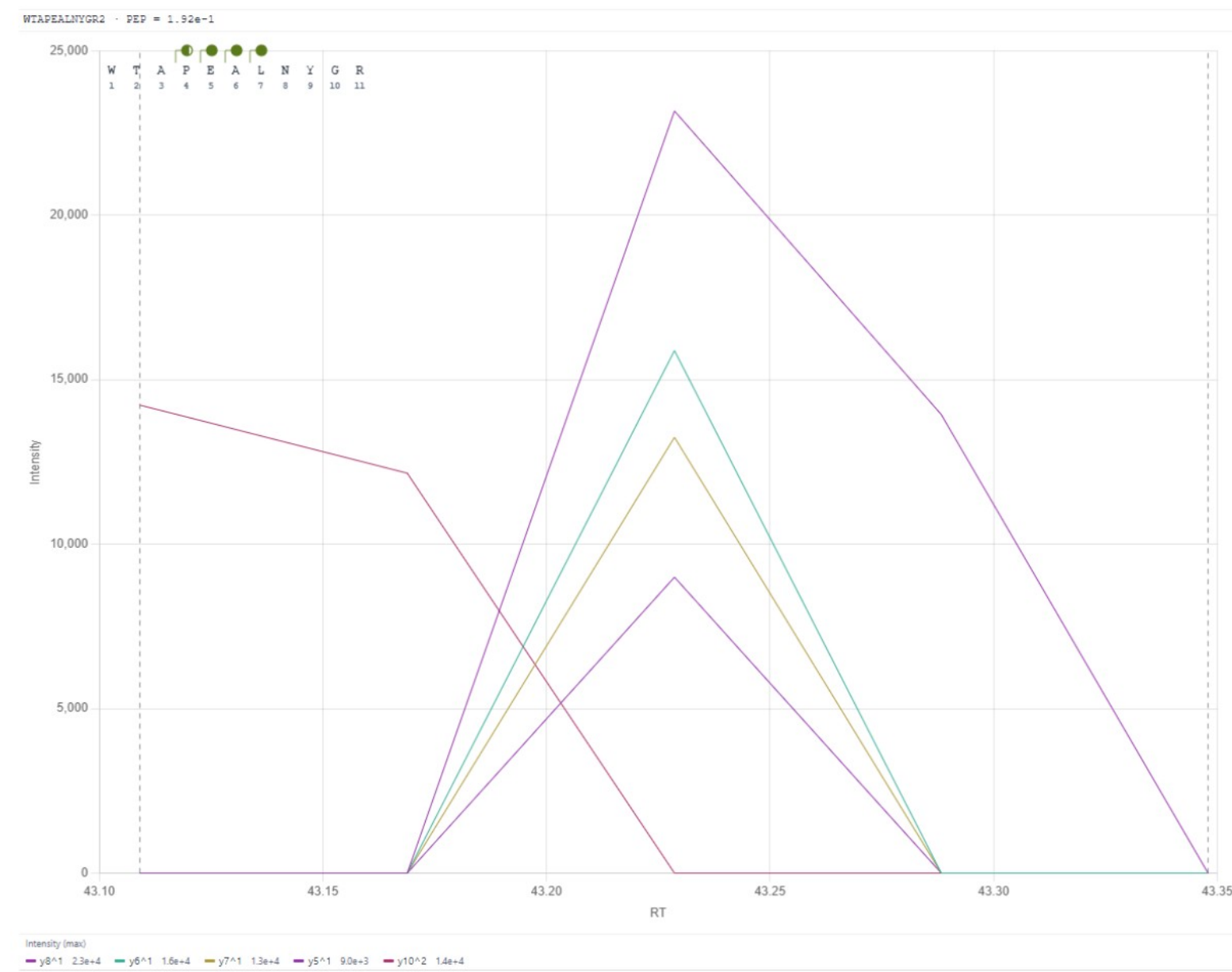

FRMD8

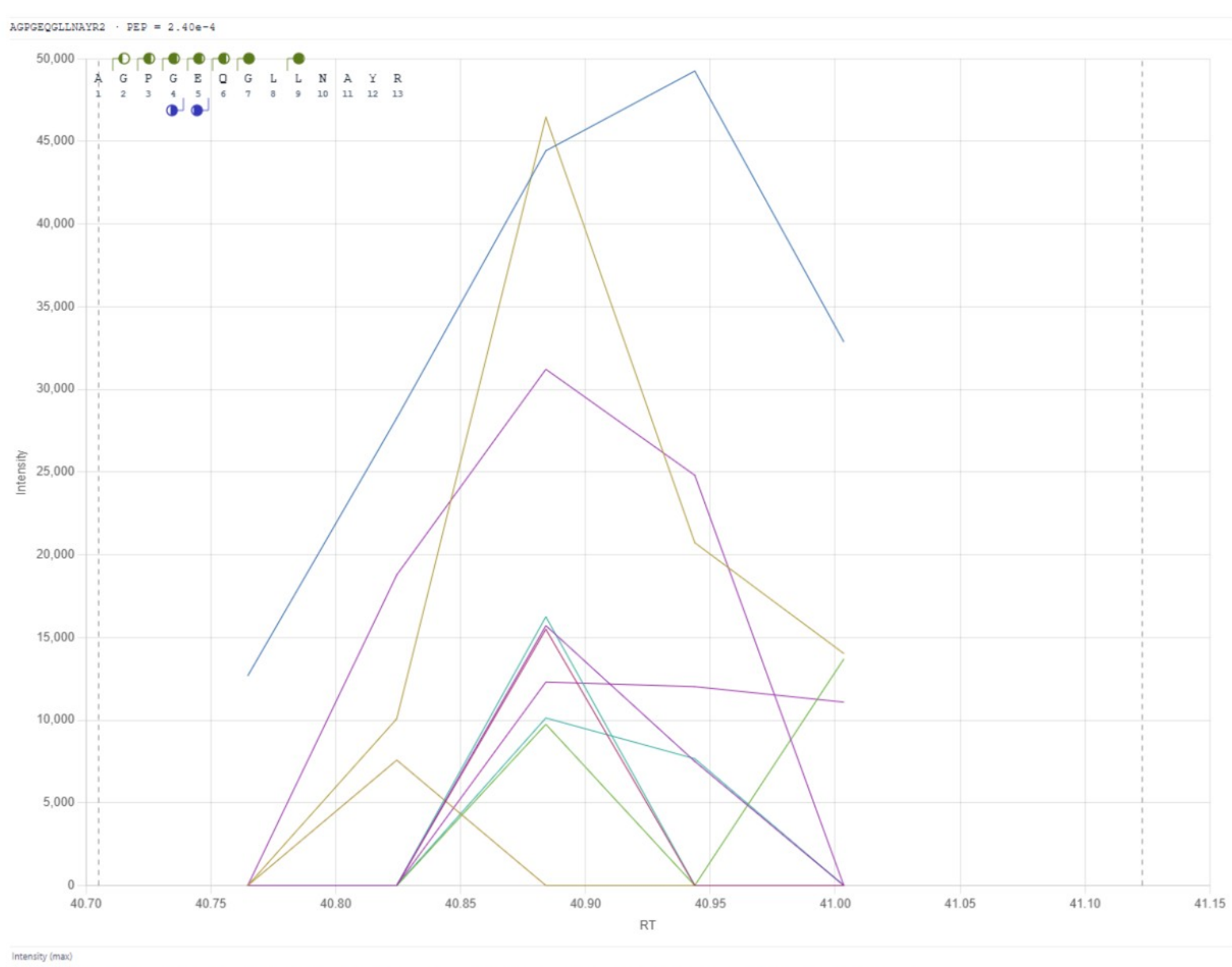

FUCA1

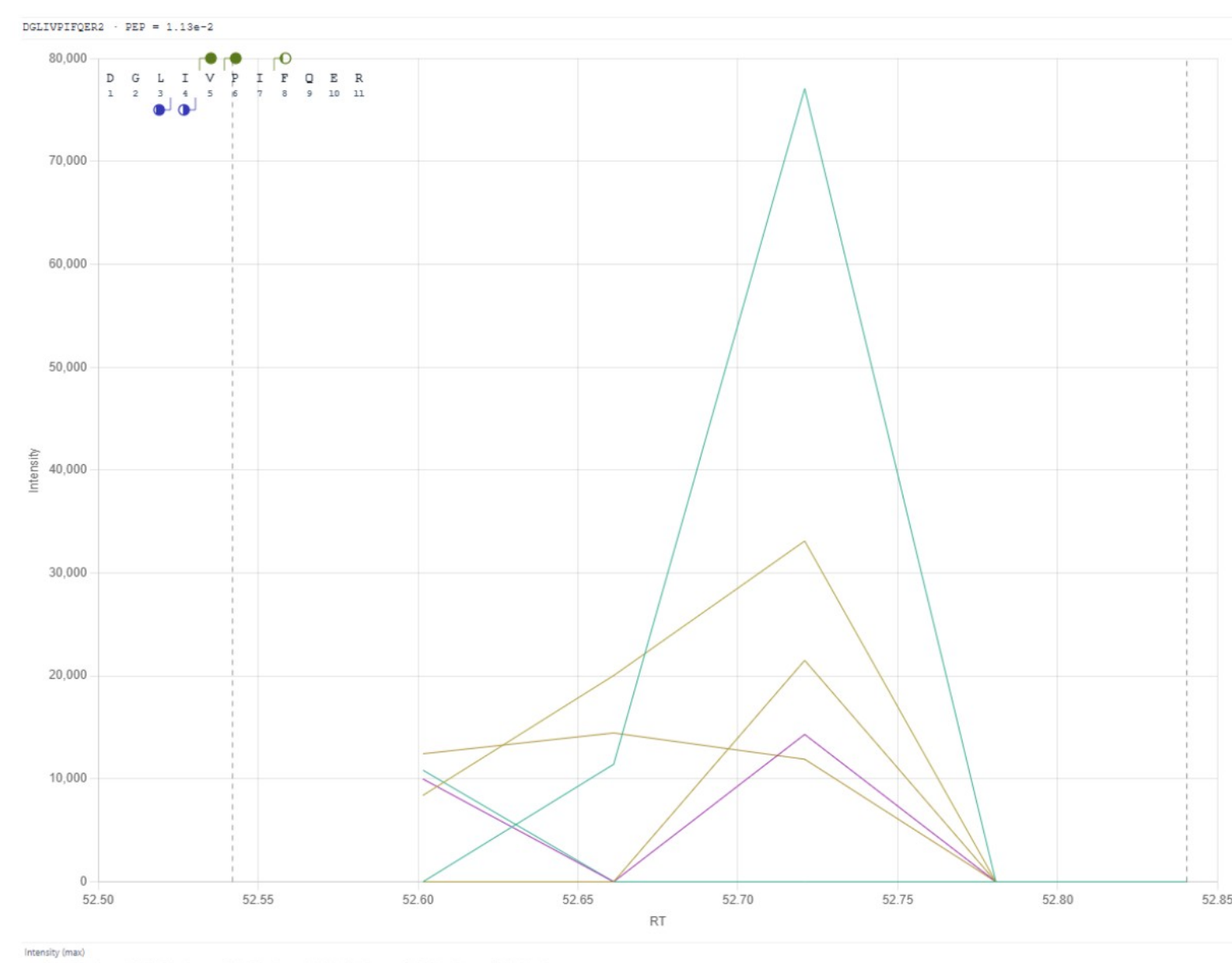

GGA1

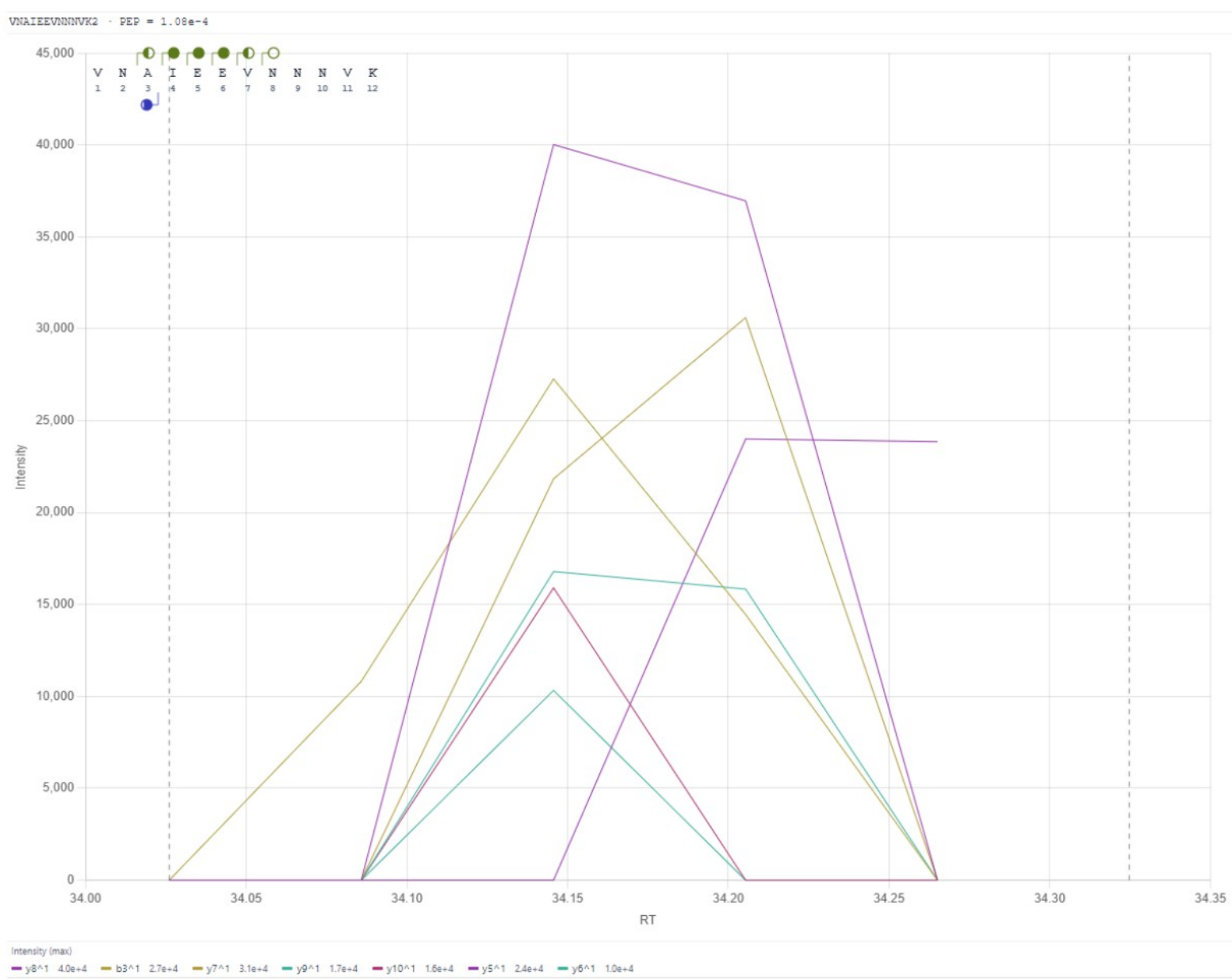

GGA3

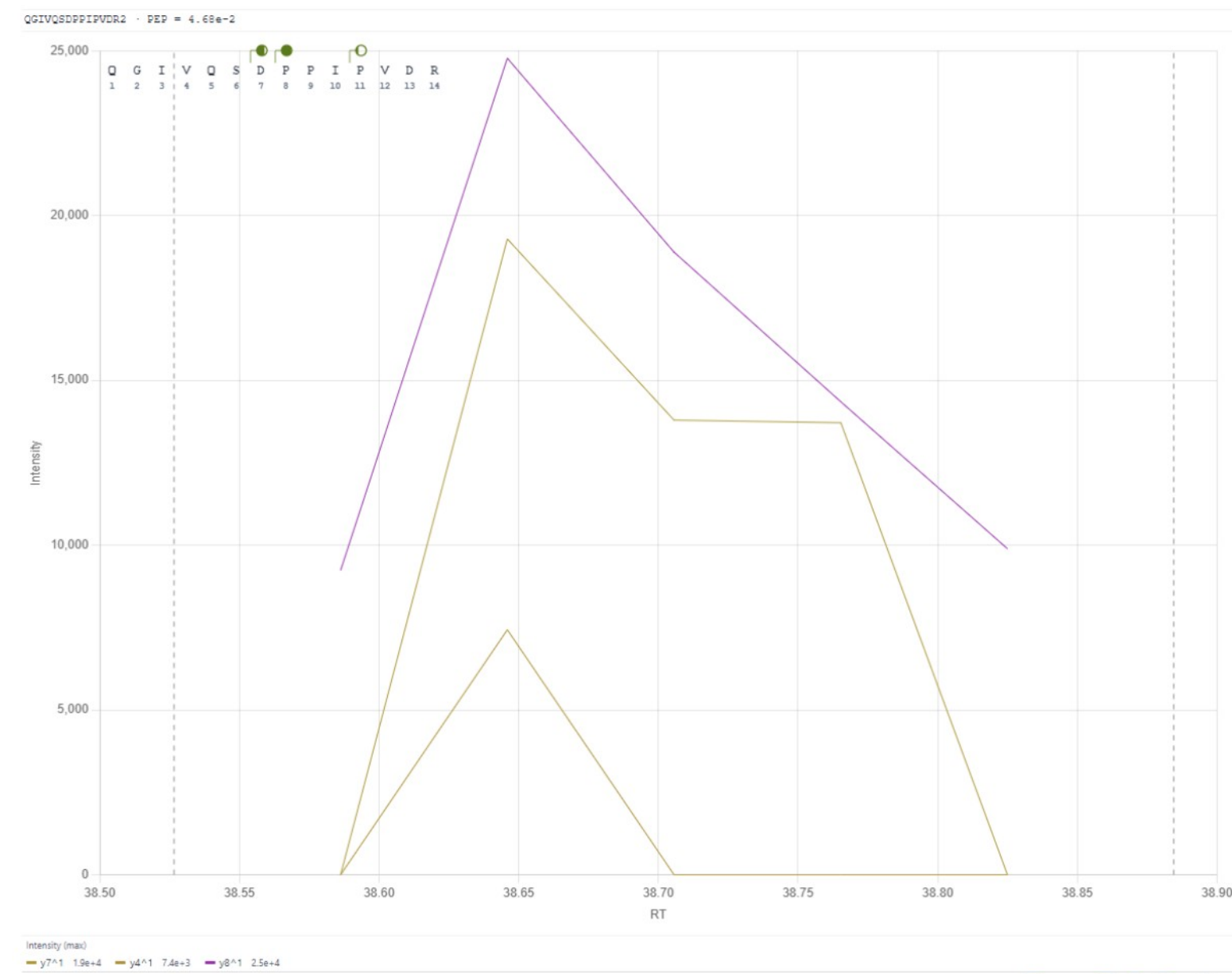

GIPC3

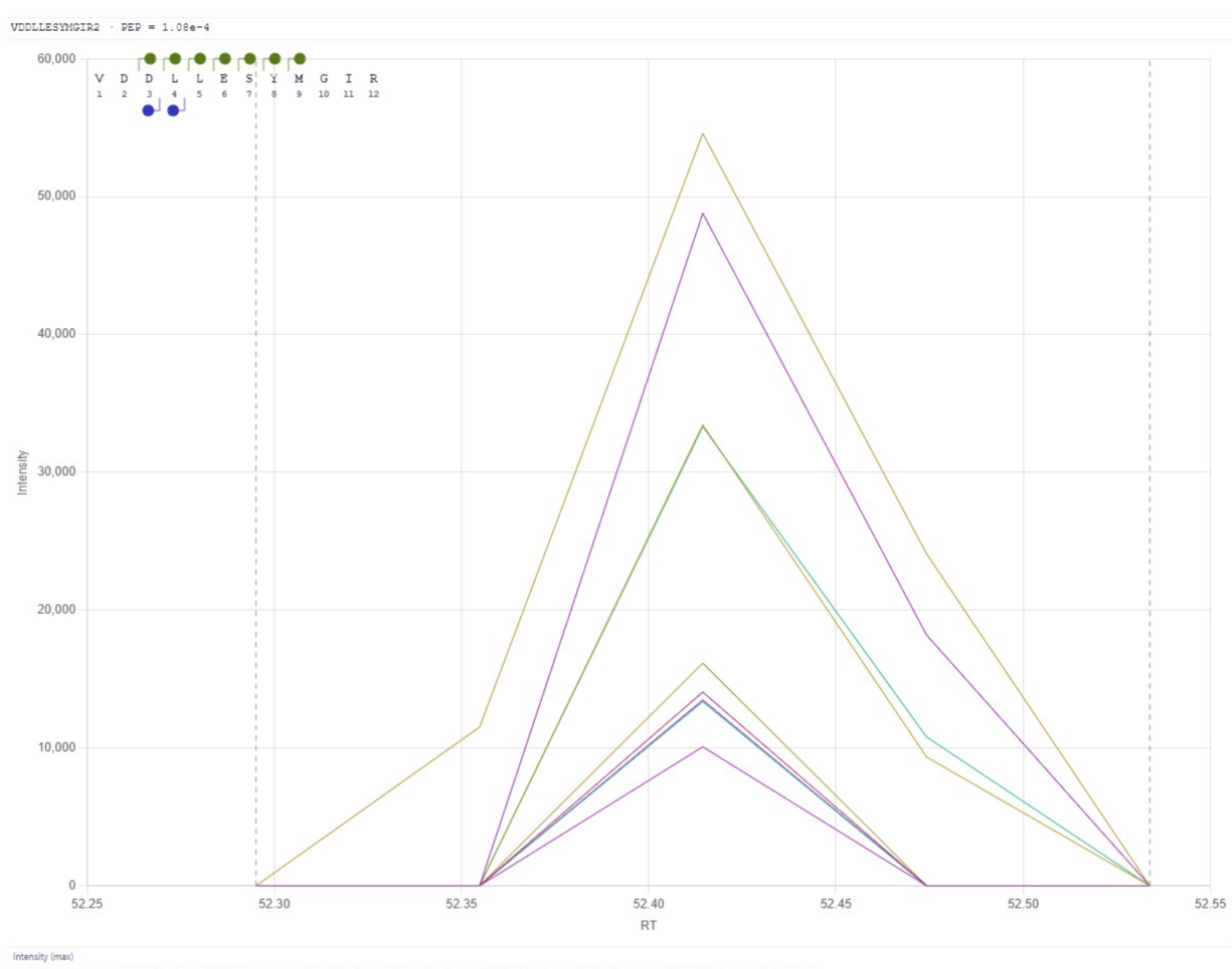

GIT1

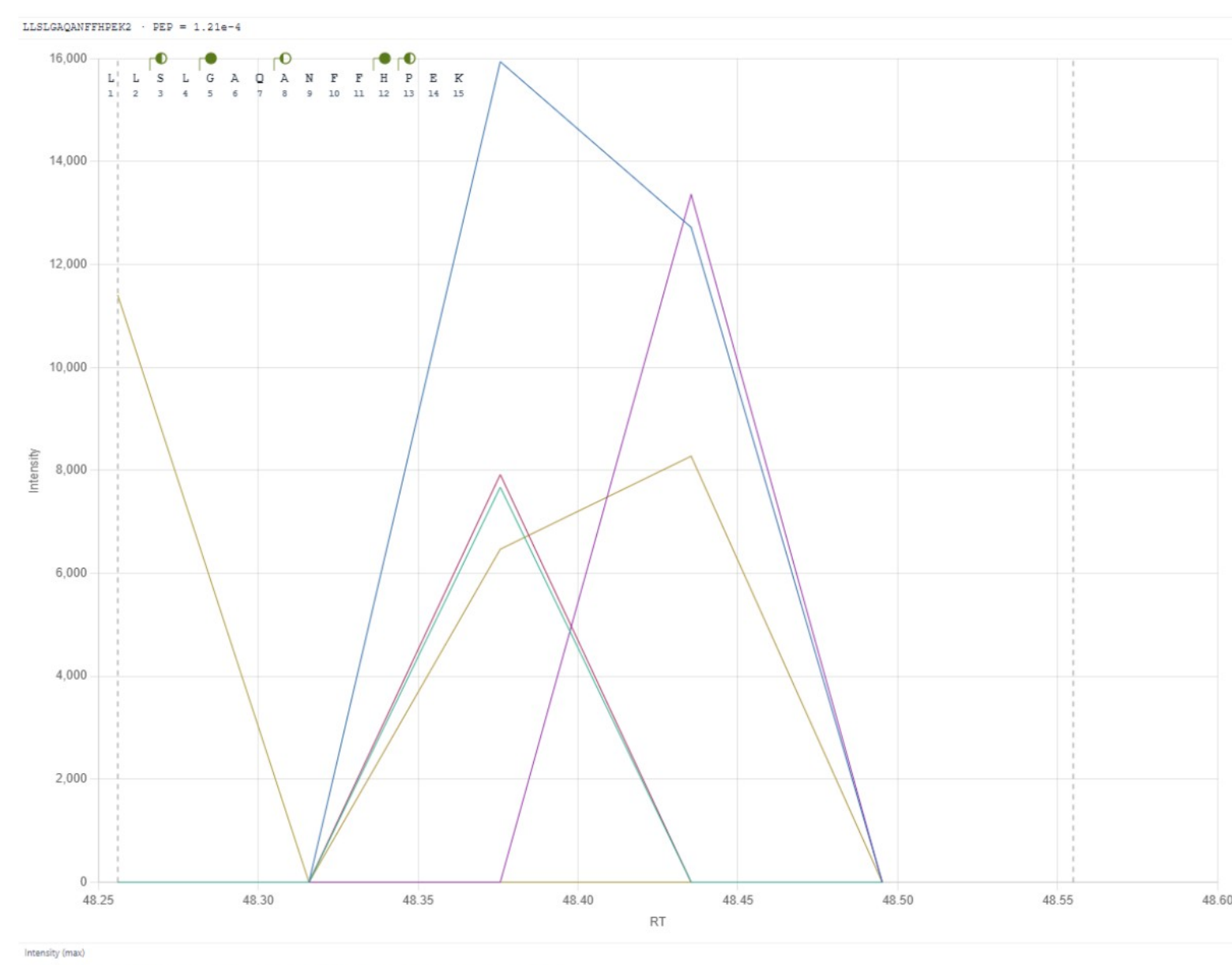

GMFG

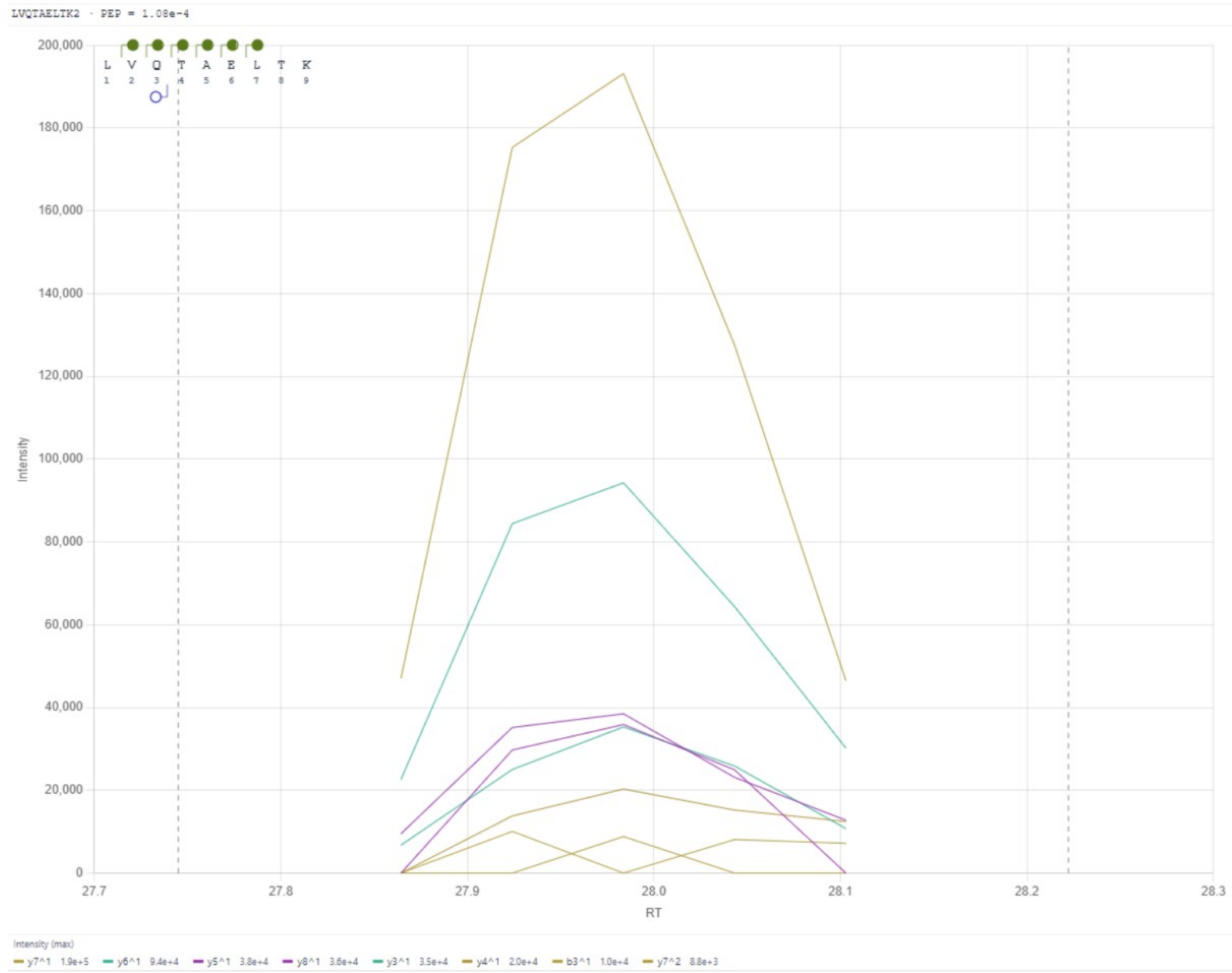

GMIP

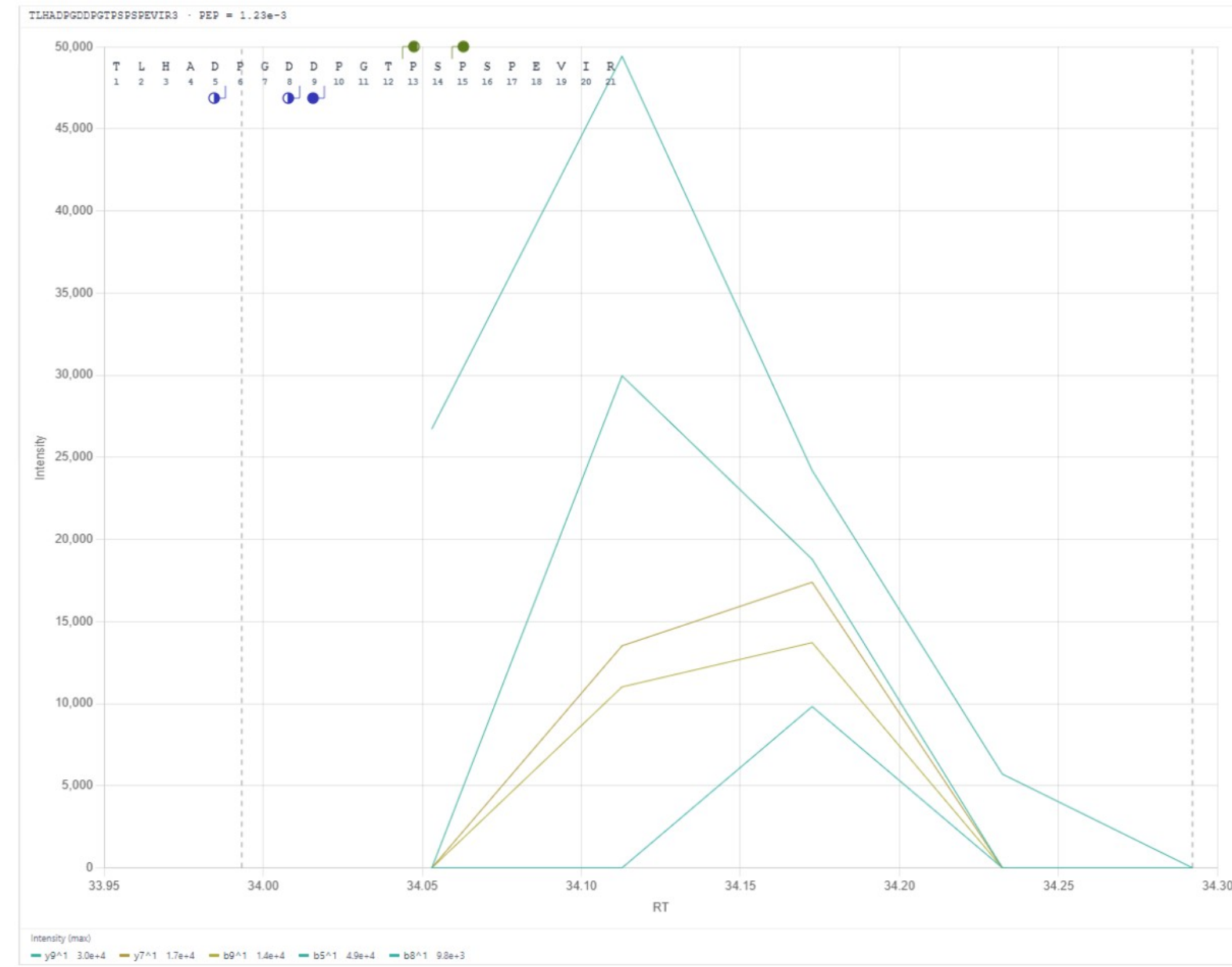

GMPR2

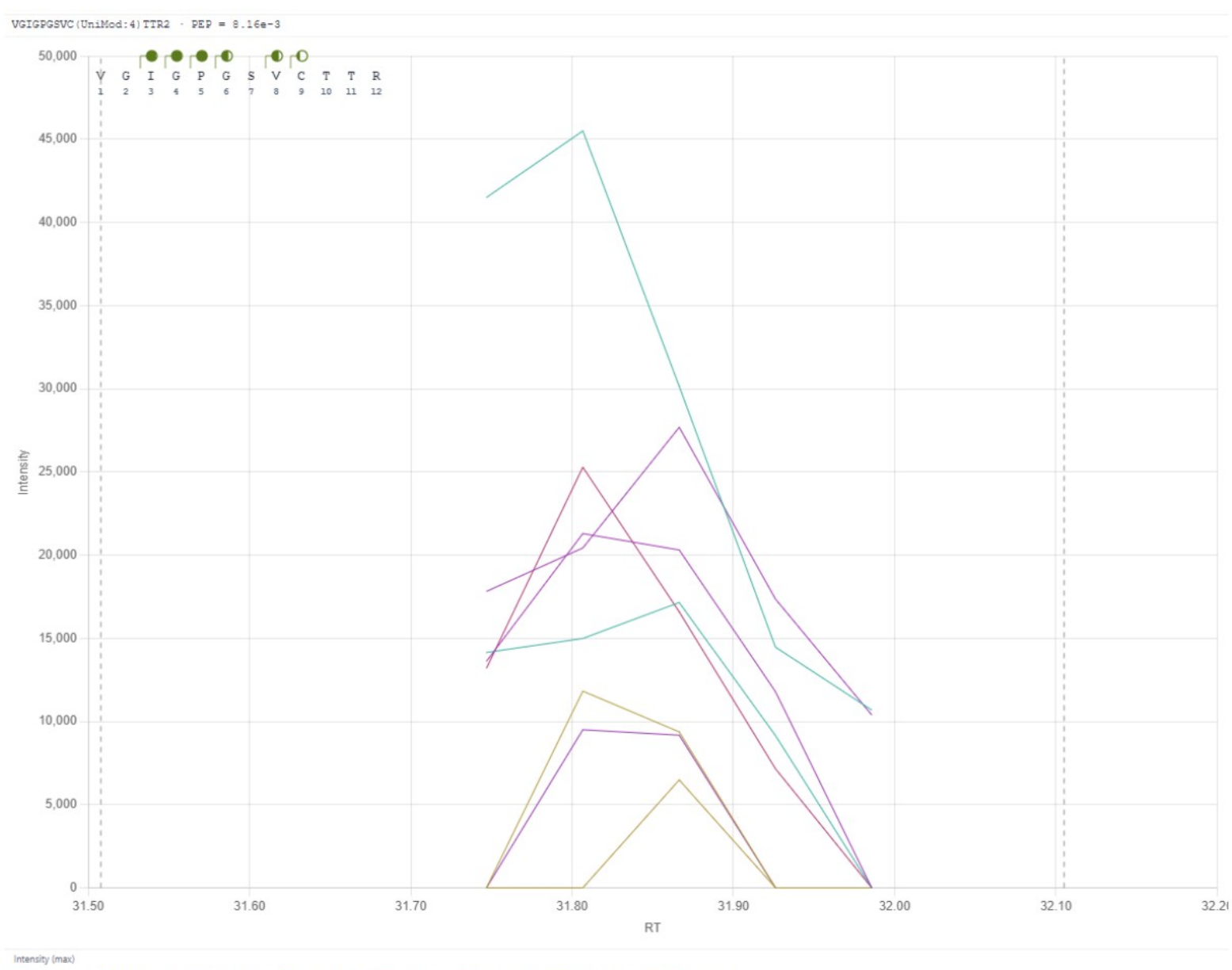

GNAQ

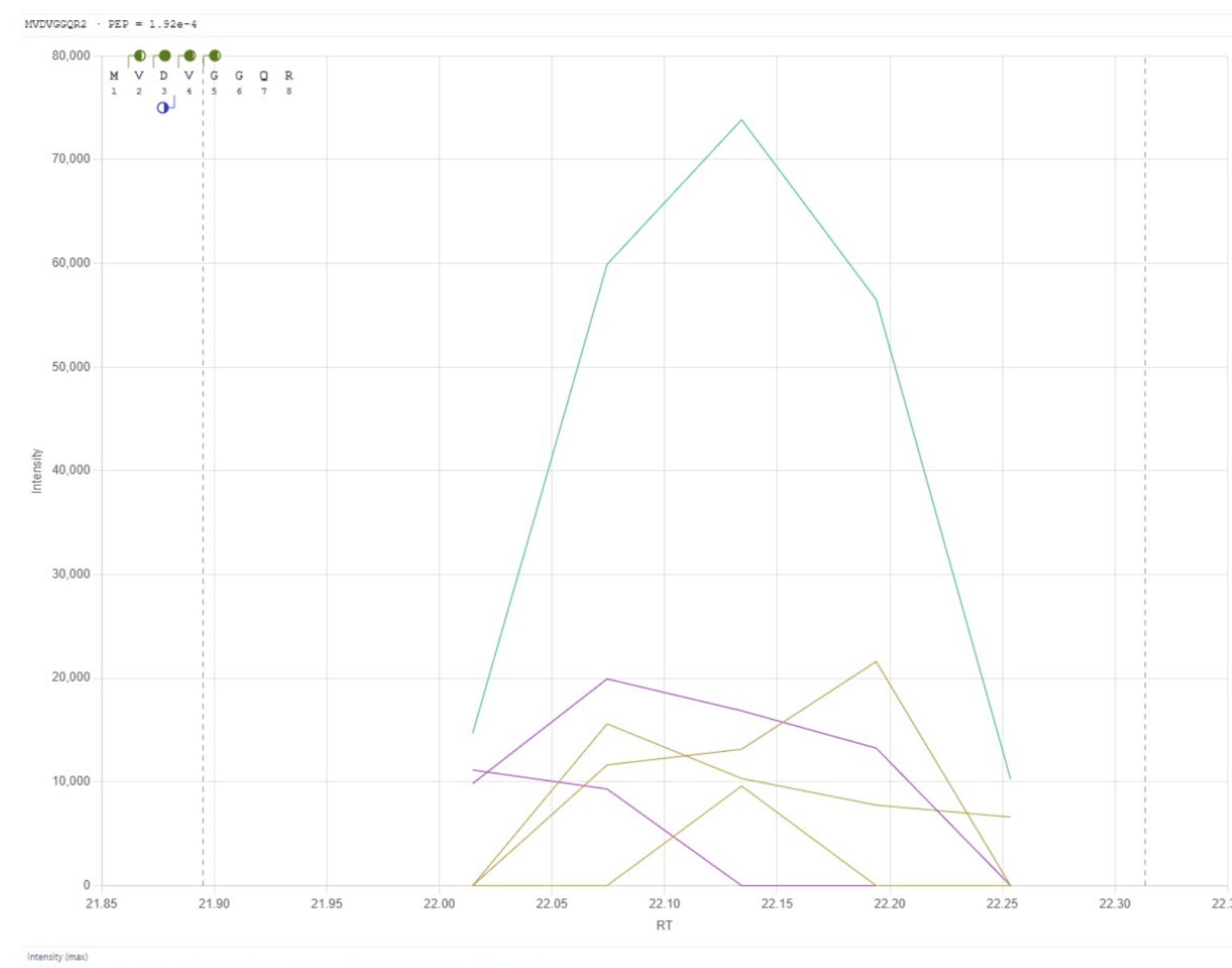

GNPNAT1

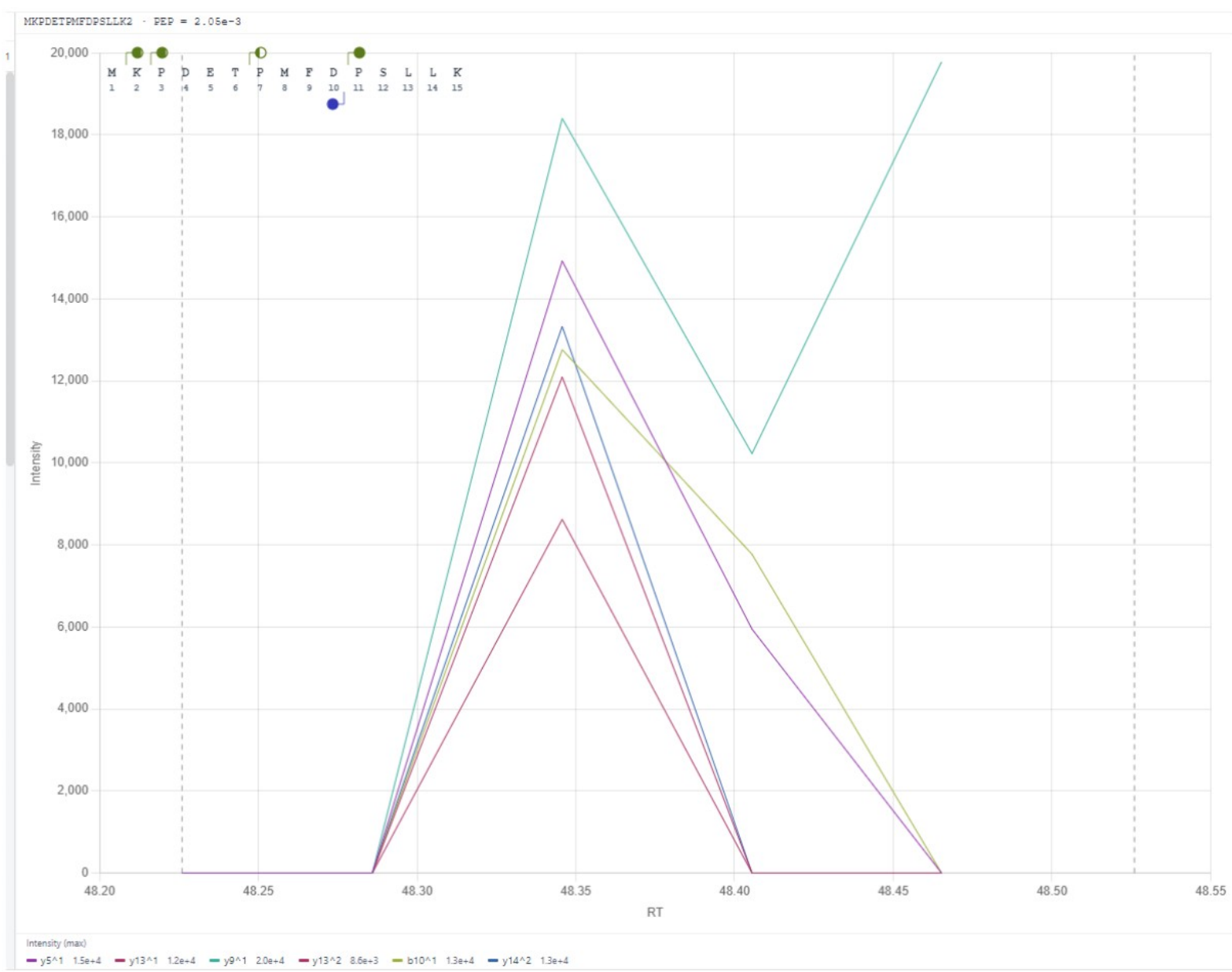

GOLT1B

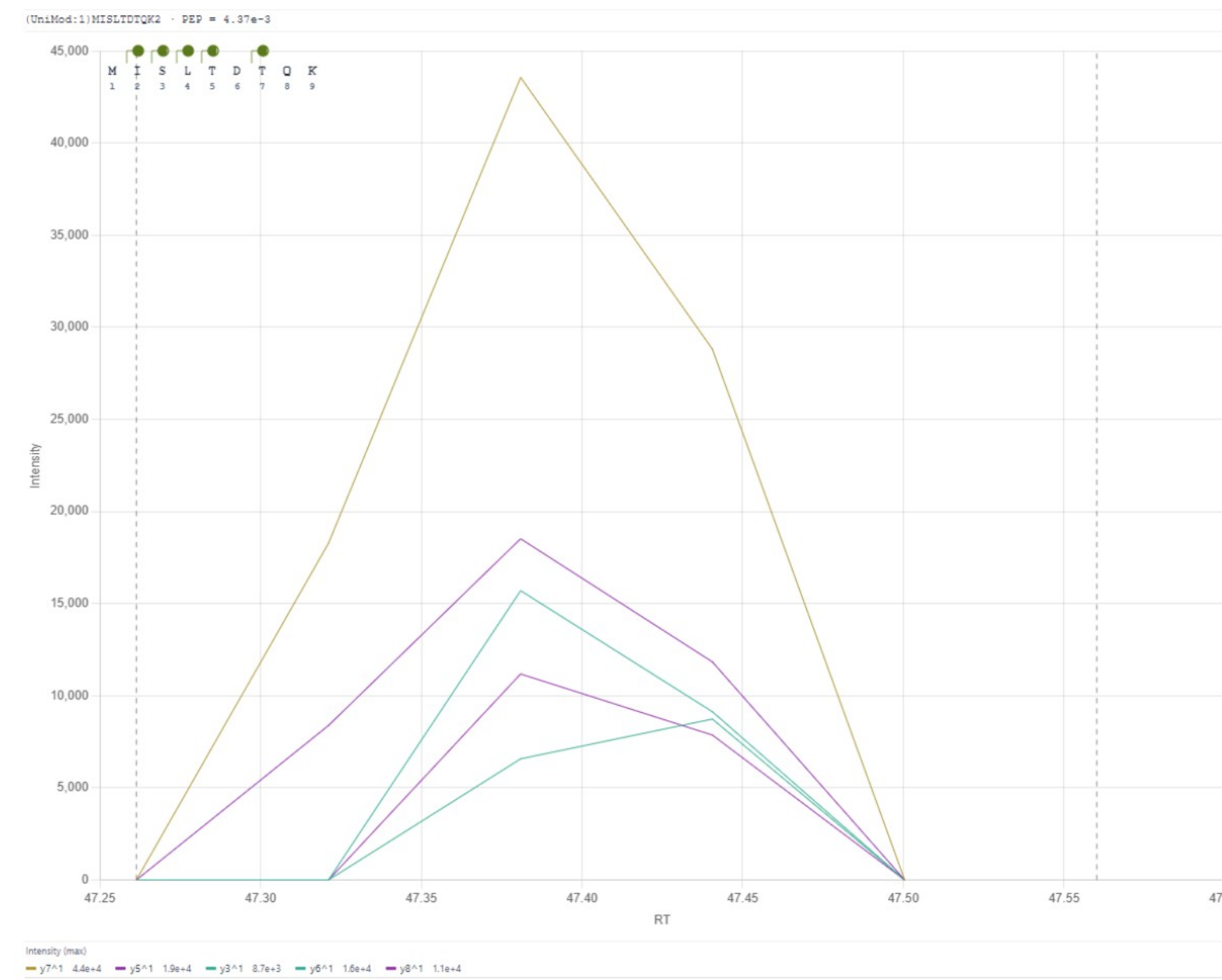

GPI

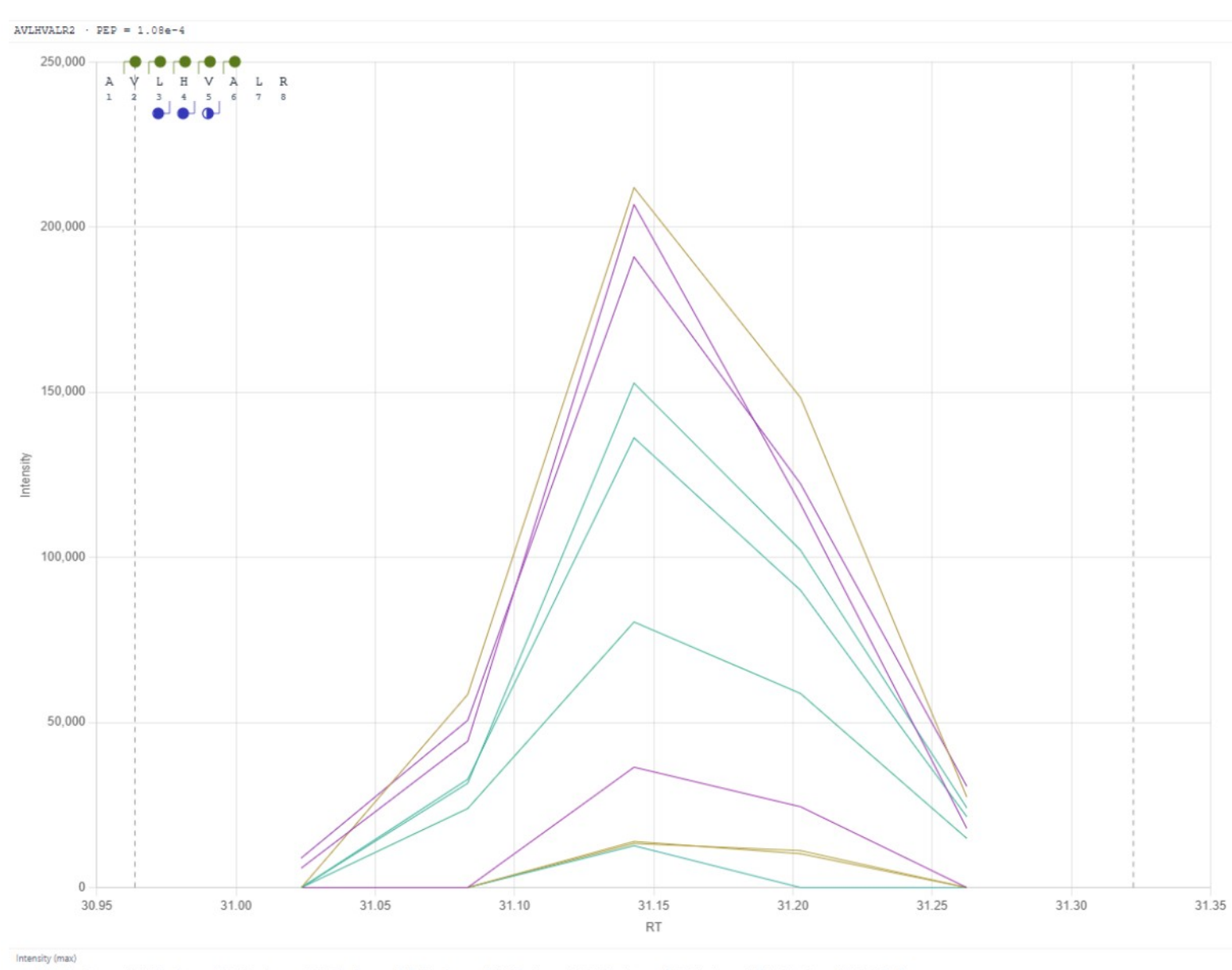

GSK3A

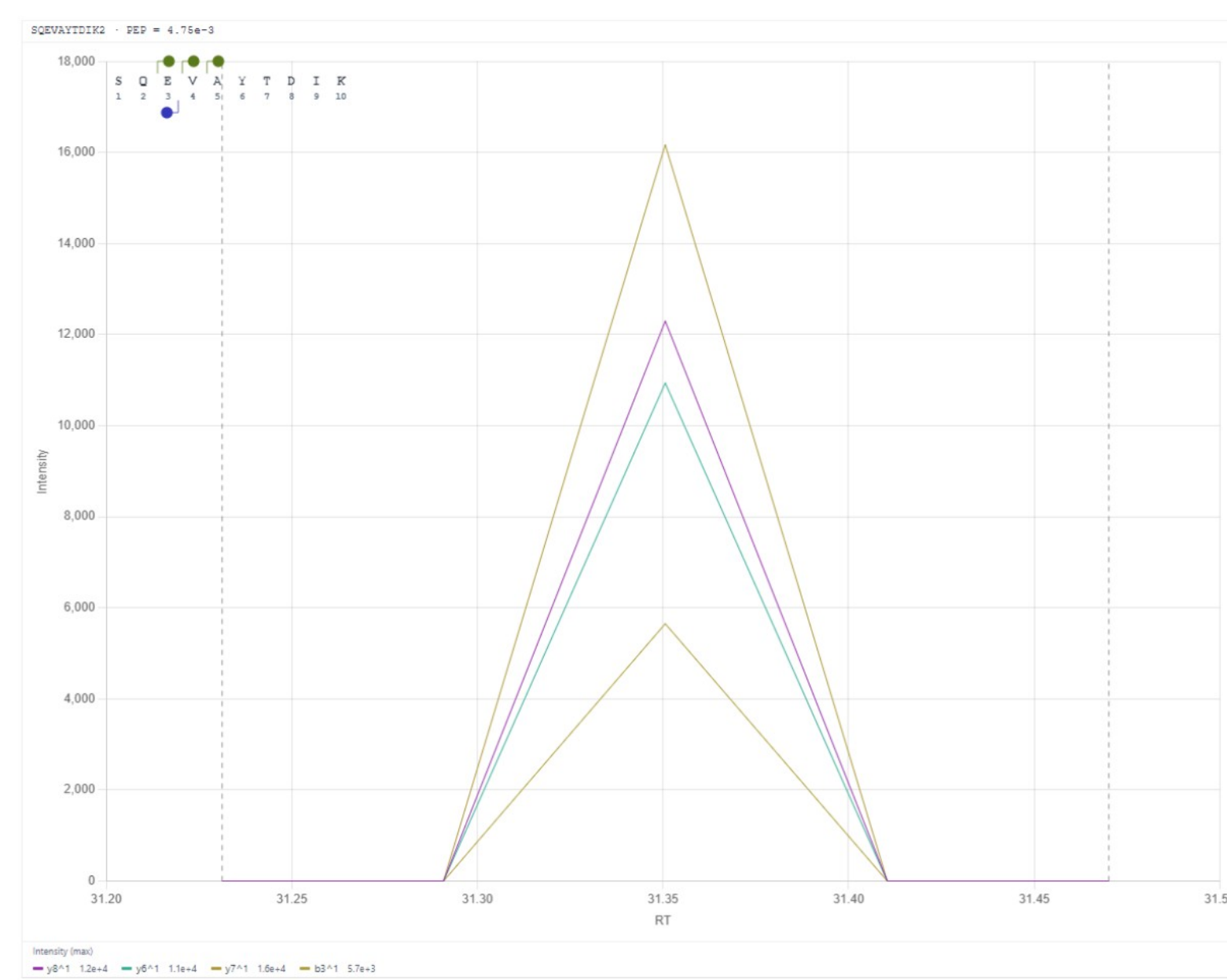

GSTM5

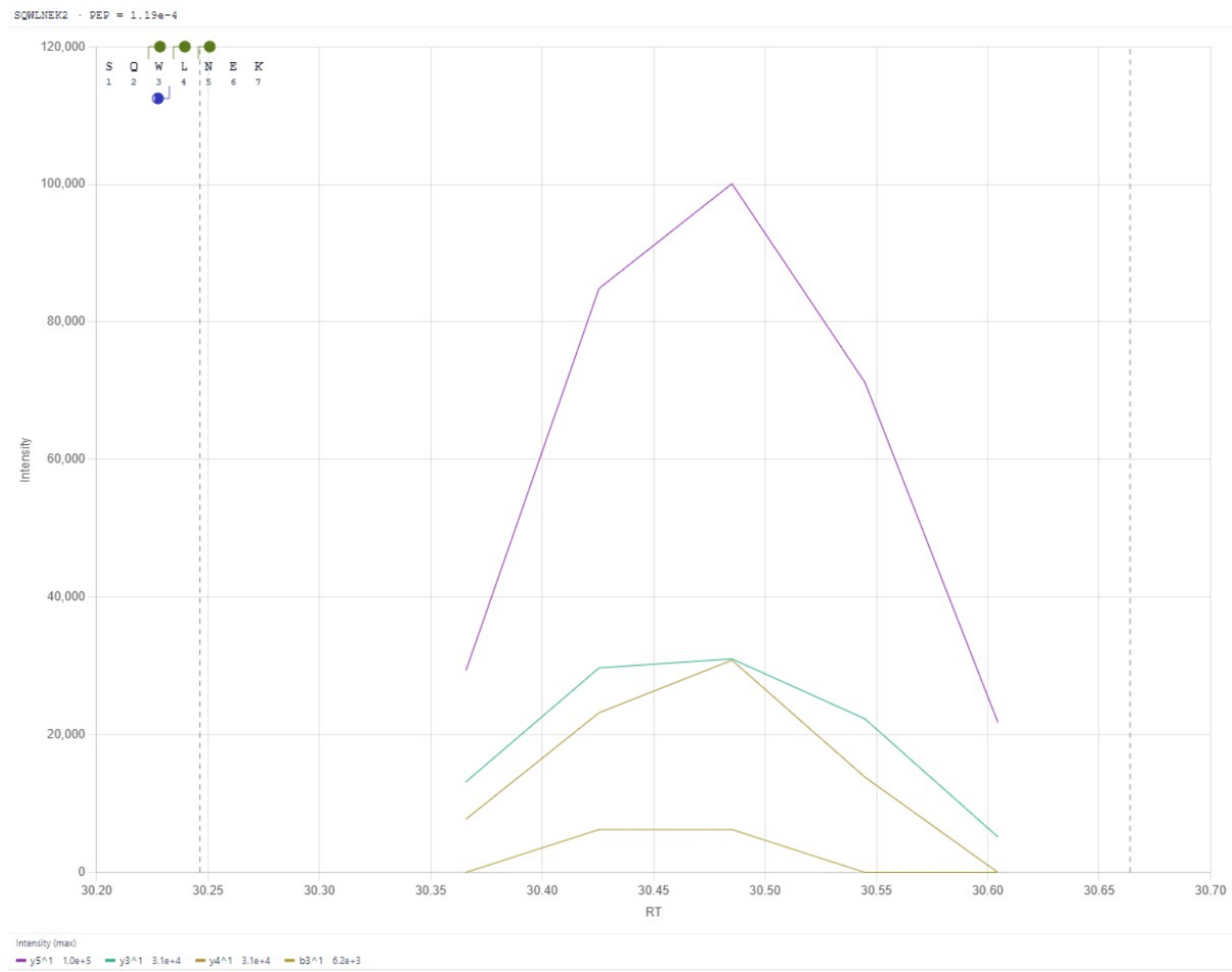

GUK1

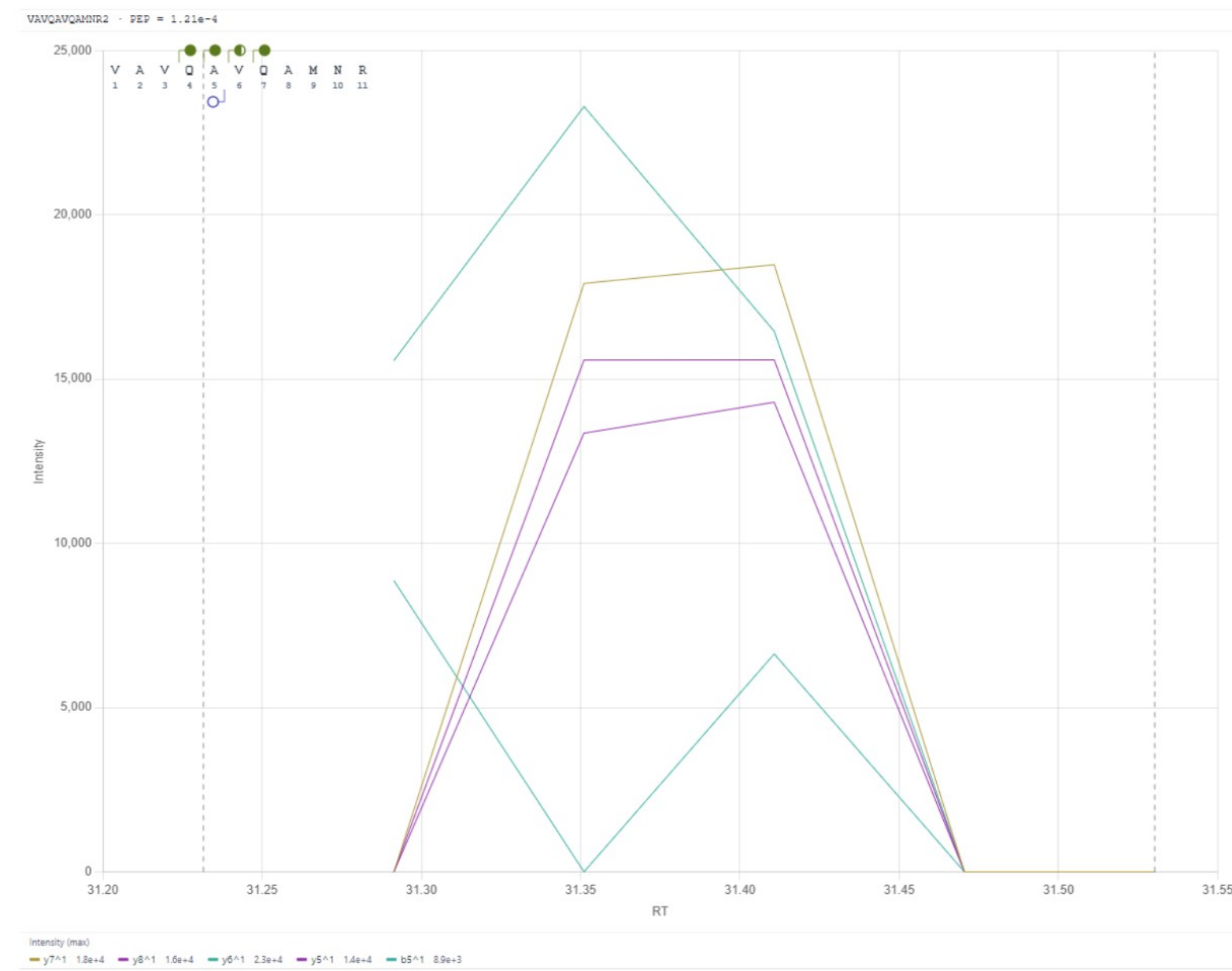

HAS1

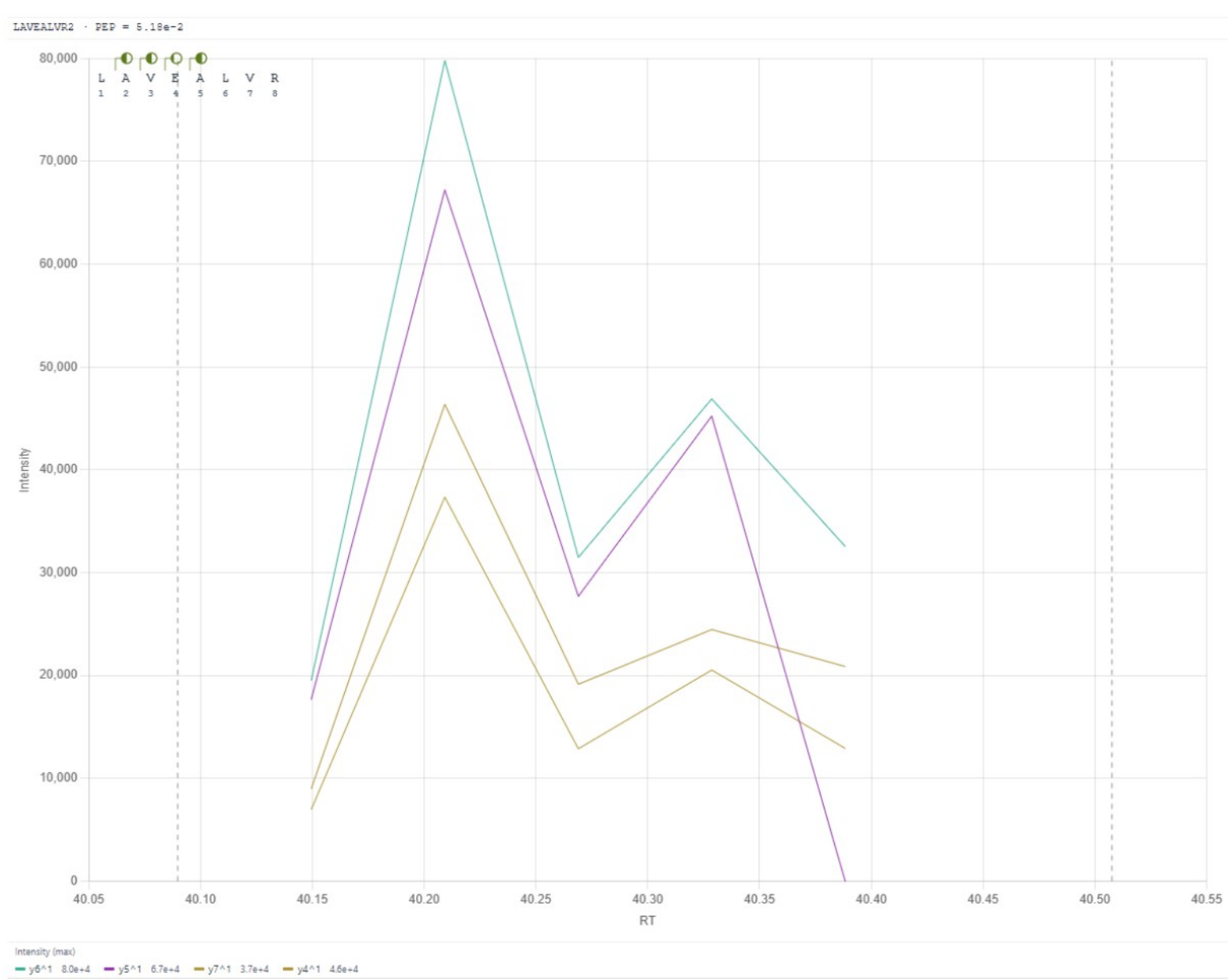

HBA

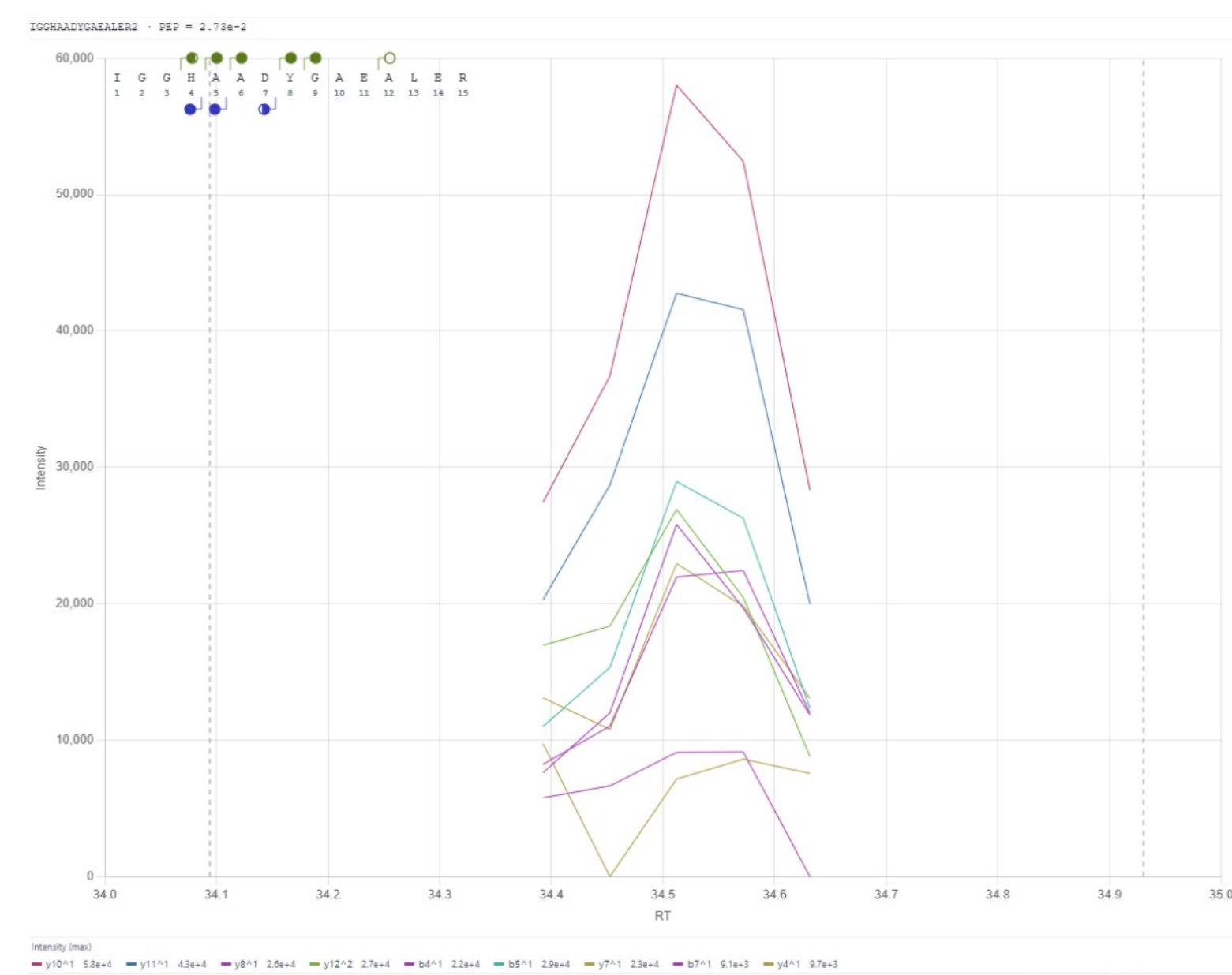

HBG2

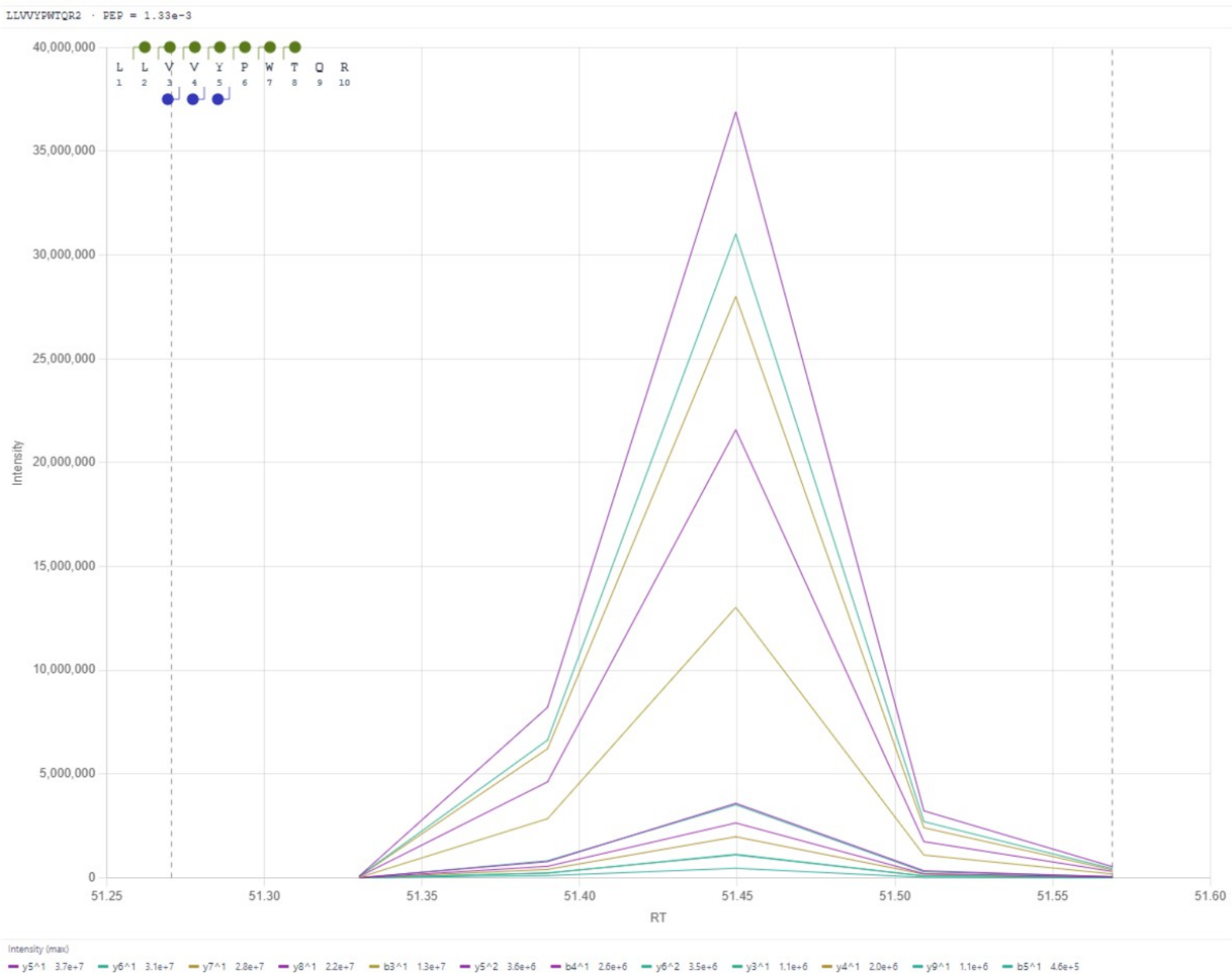

HLA-A

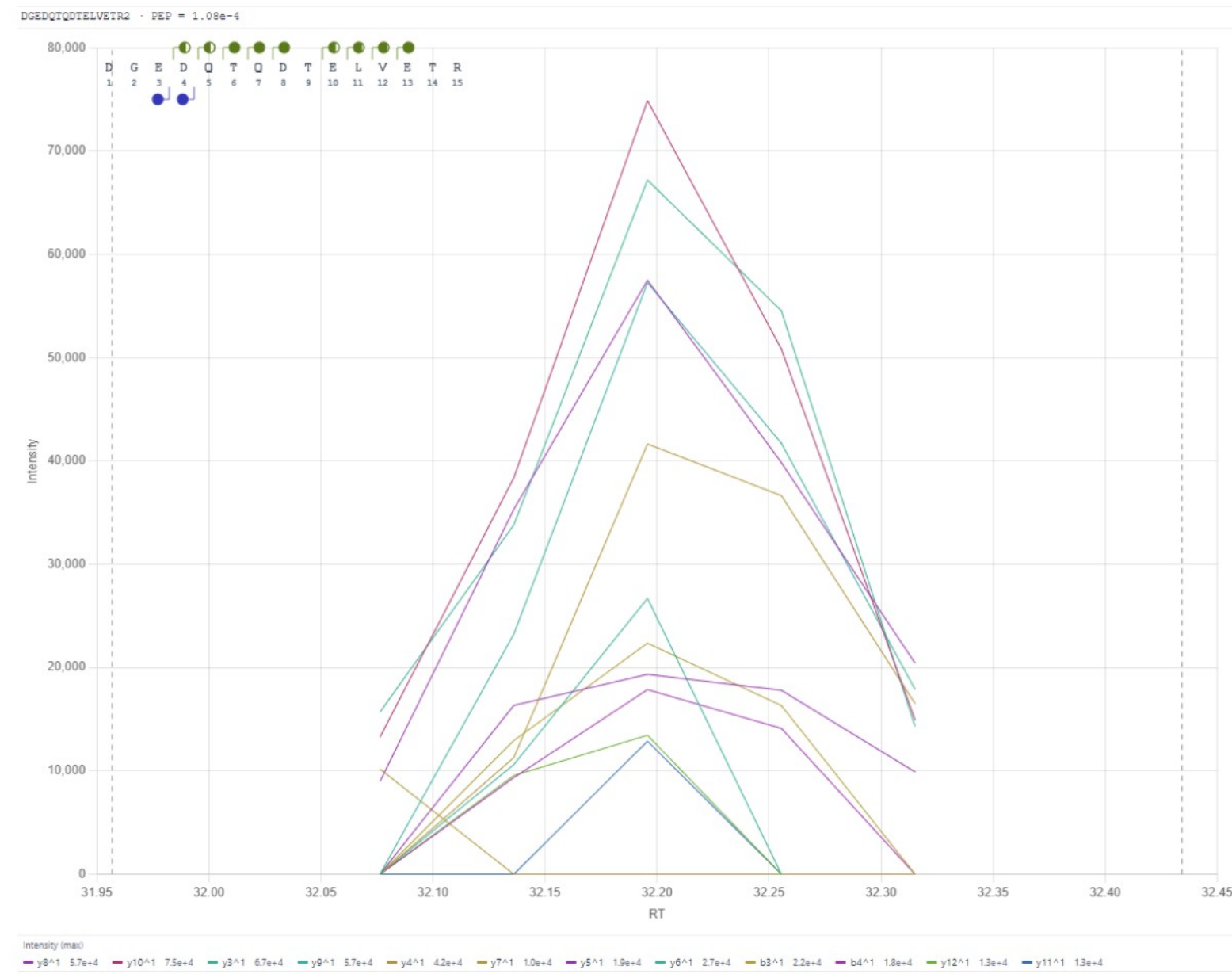

HMOX2

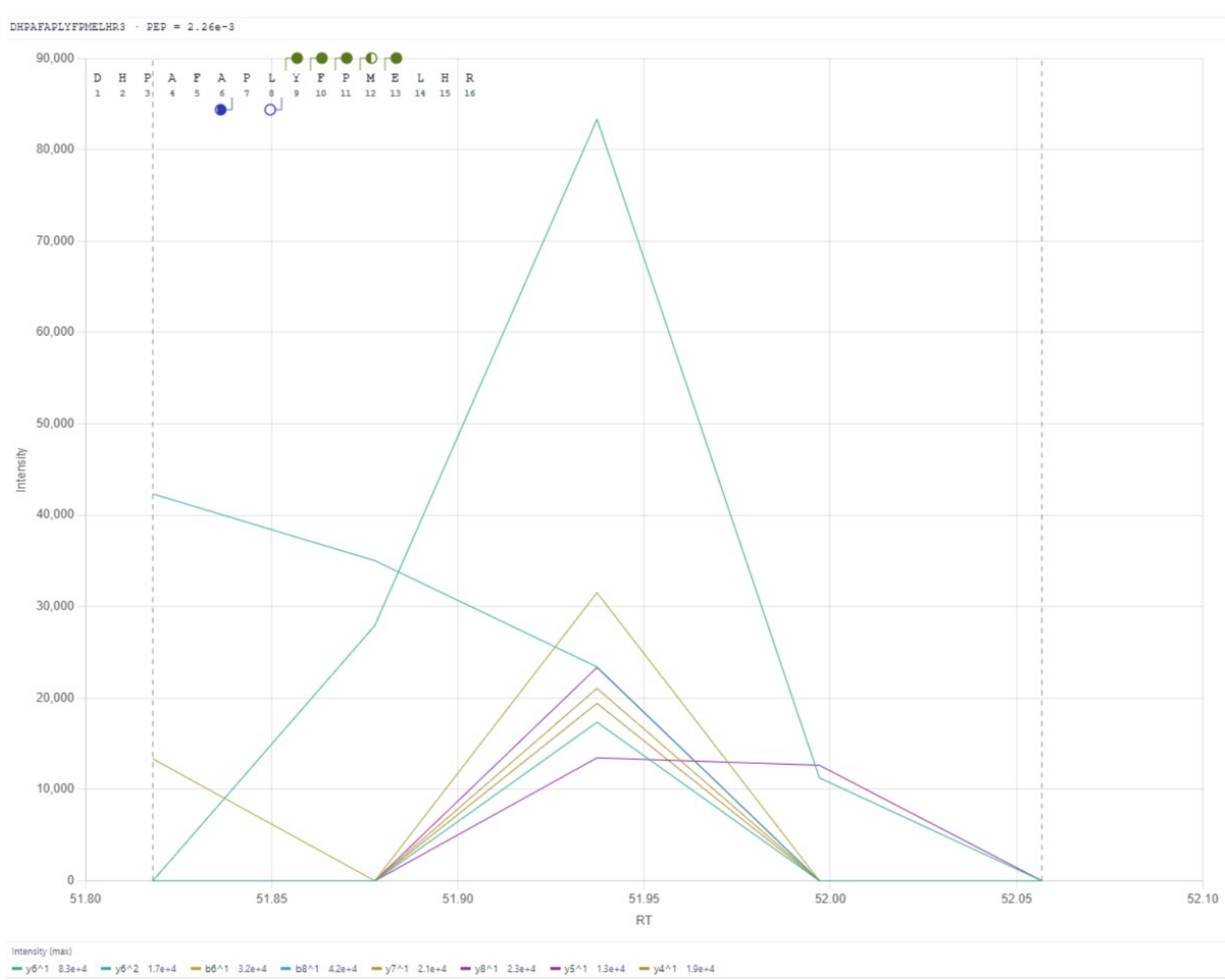

HNRNPH1

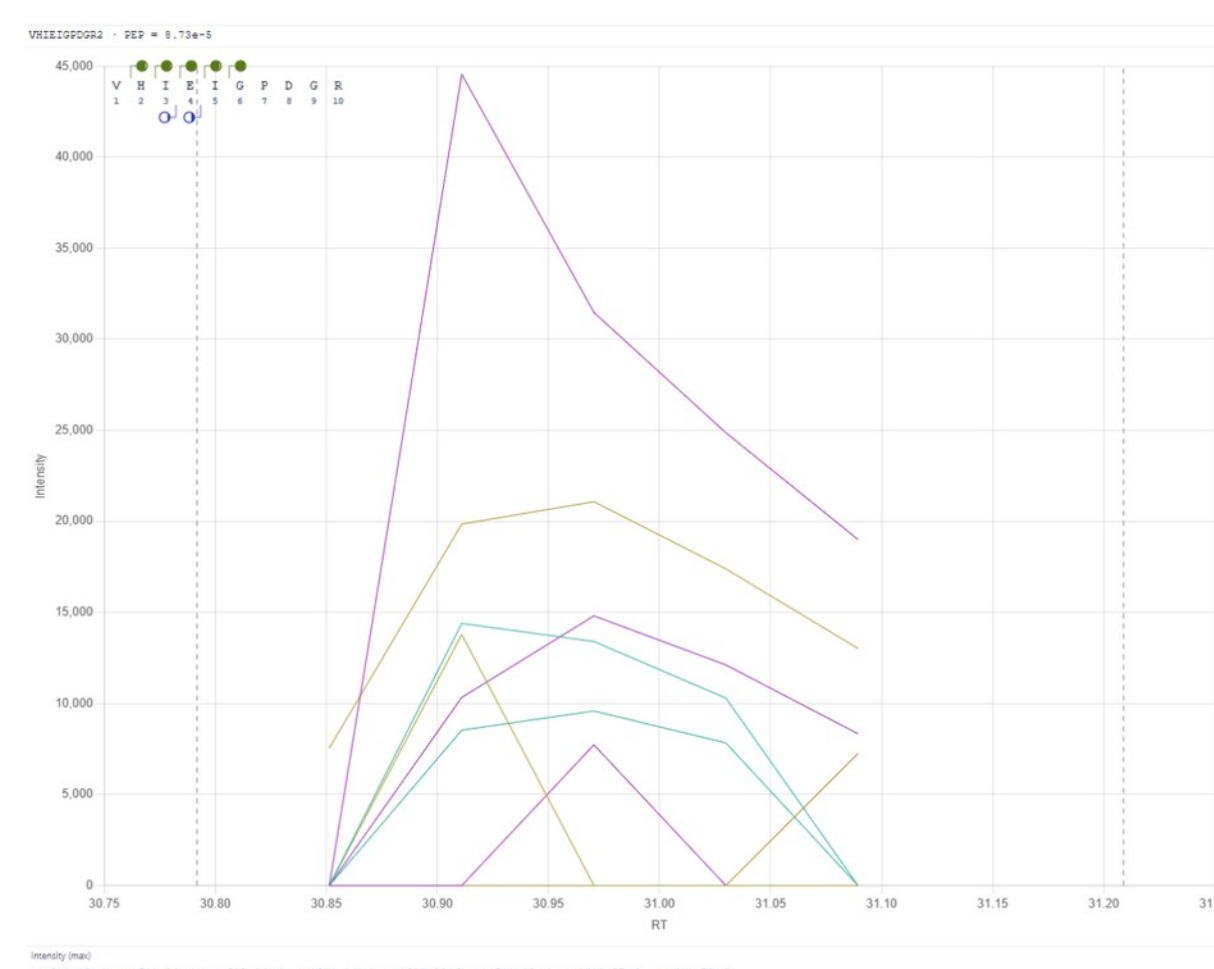

HSP90B1

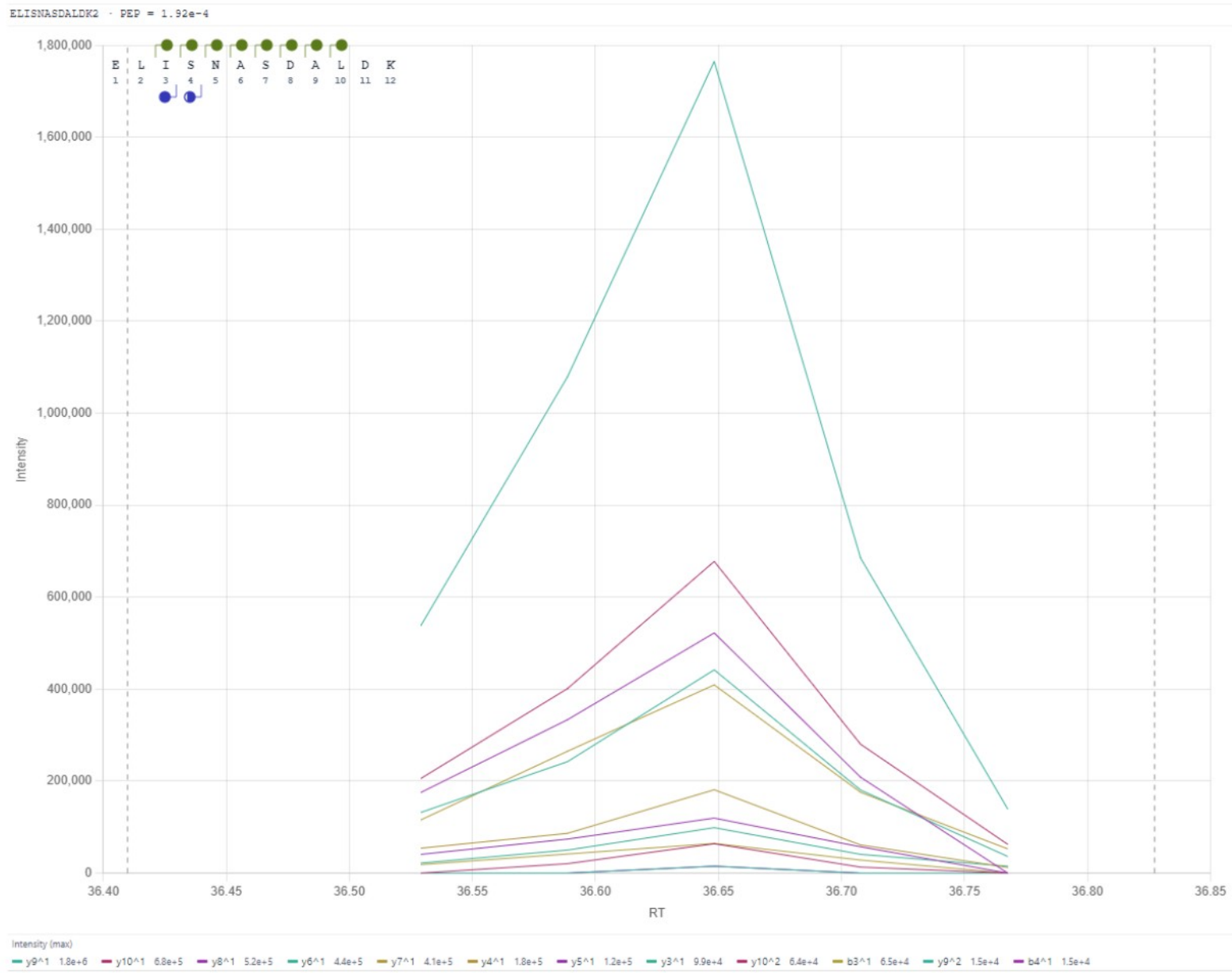

HSPA5

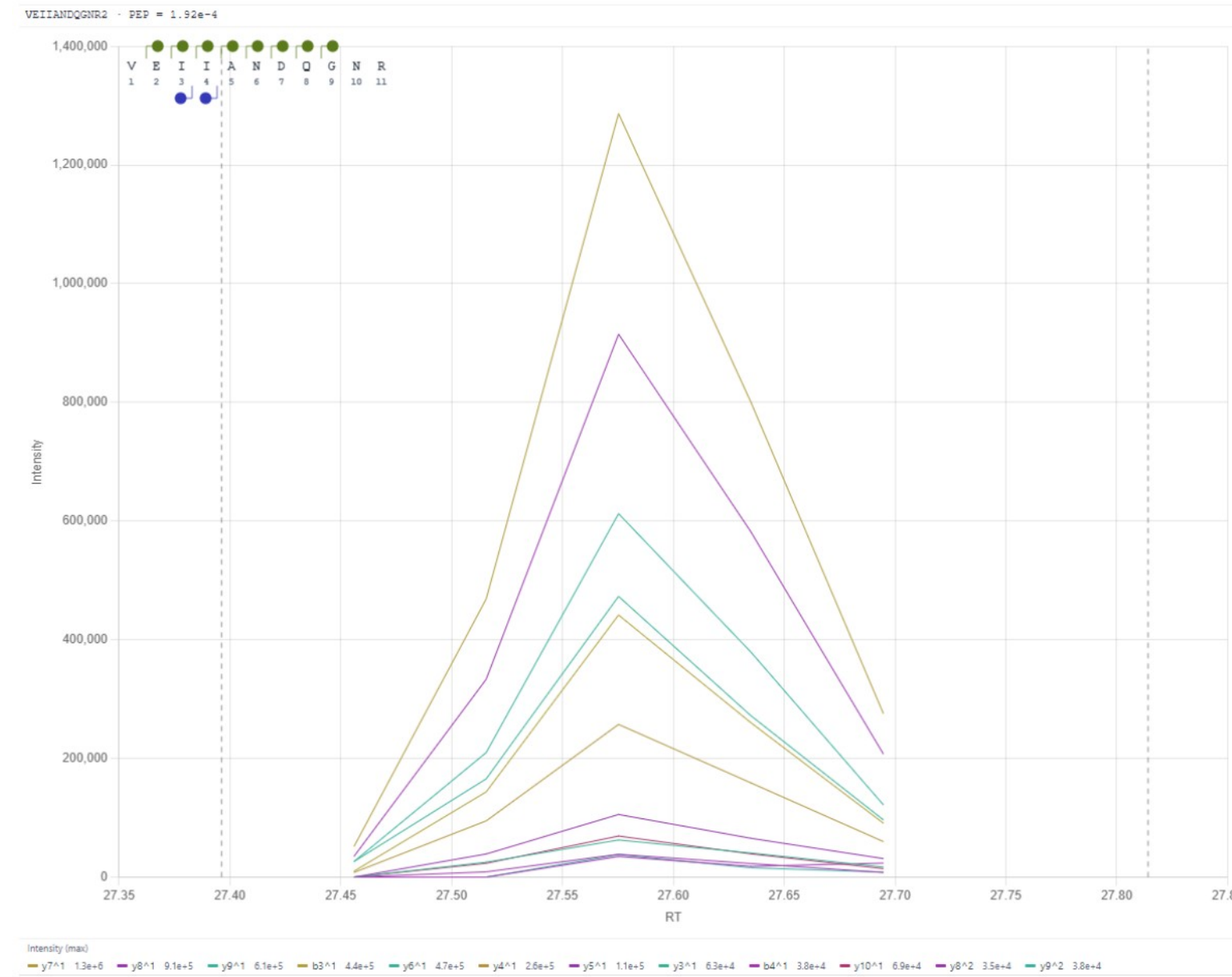

HYDIN

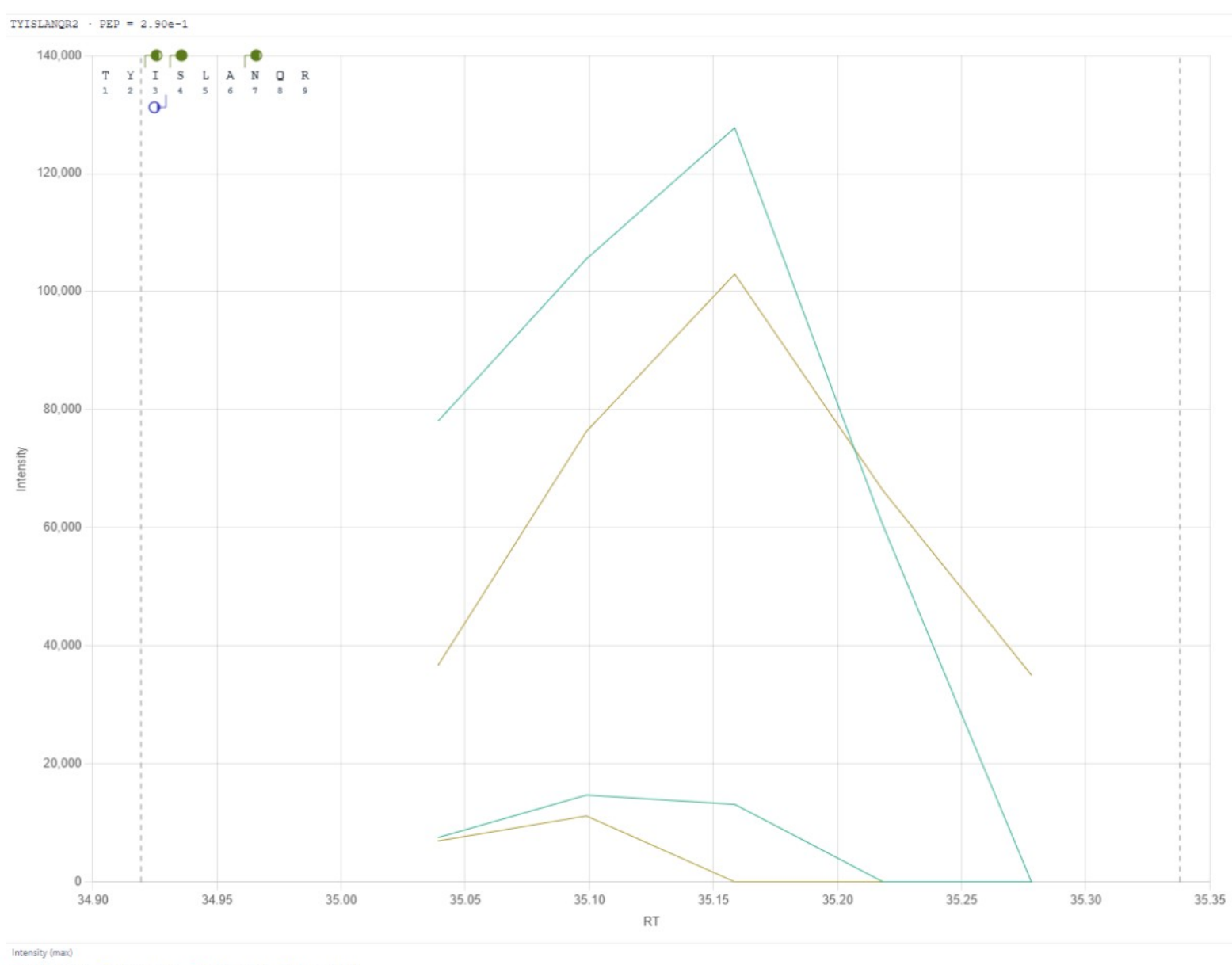

HYPK

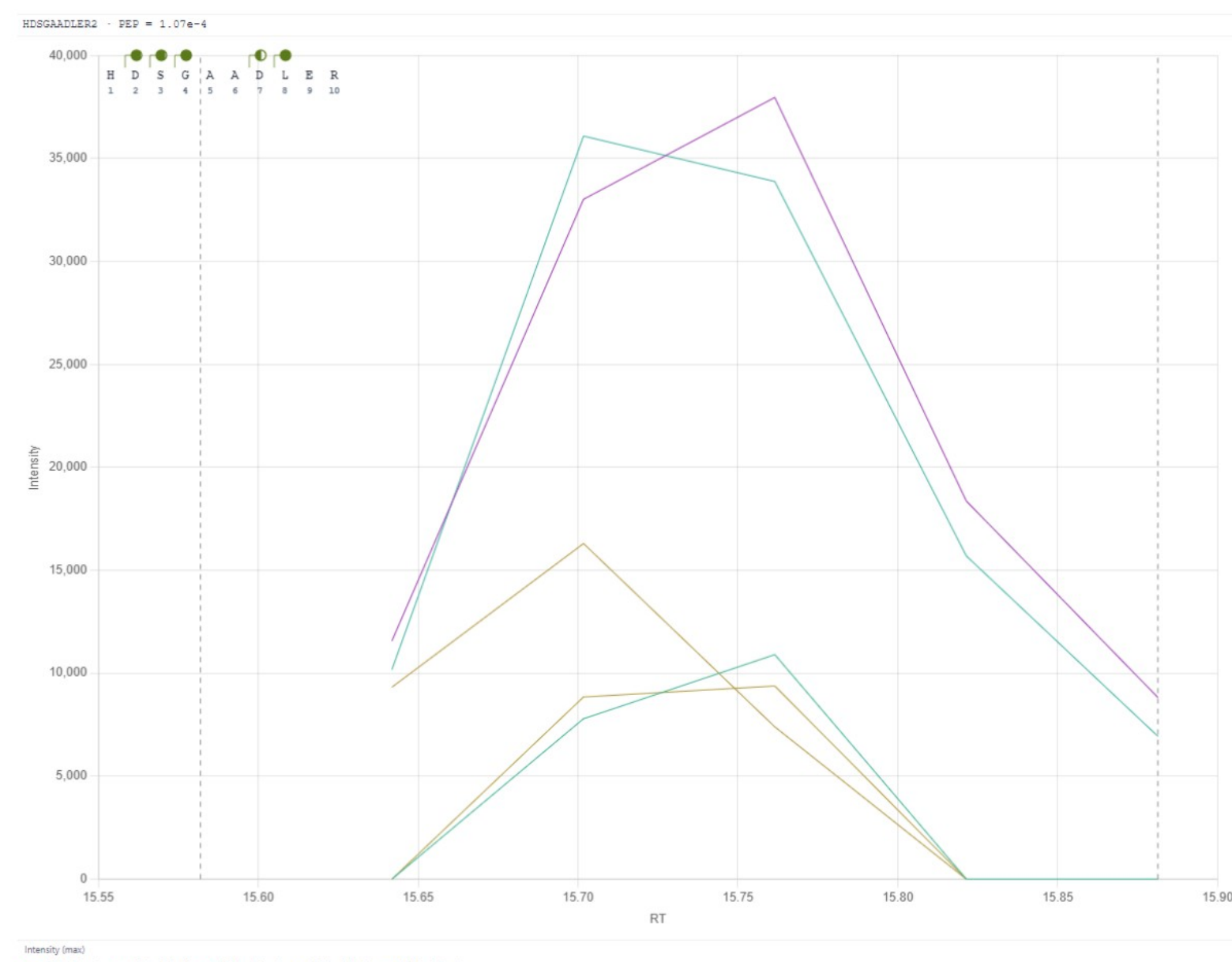

IDH2

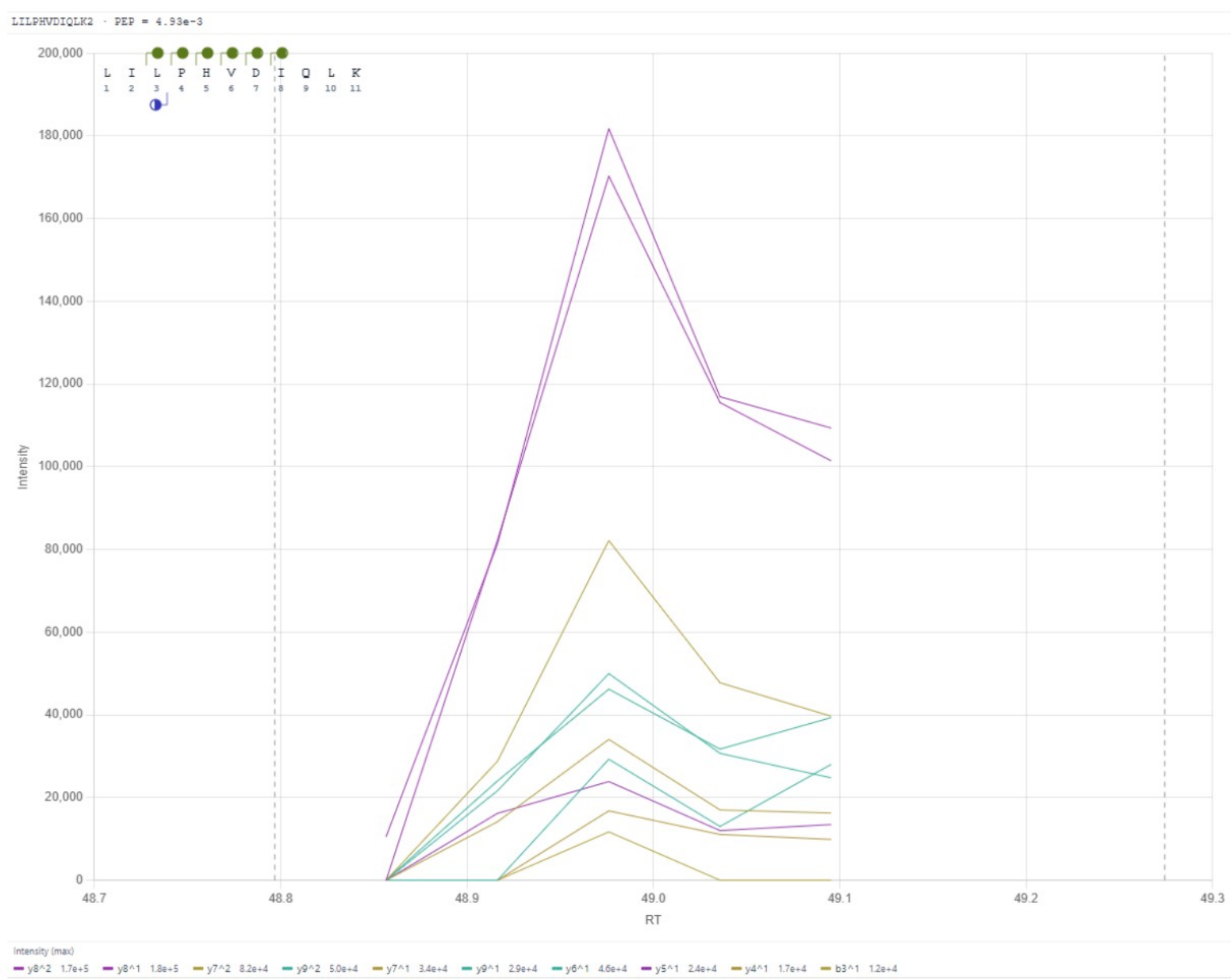

IKBKB

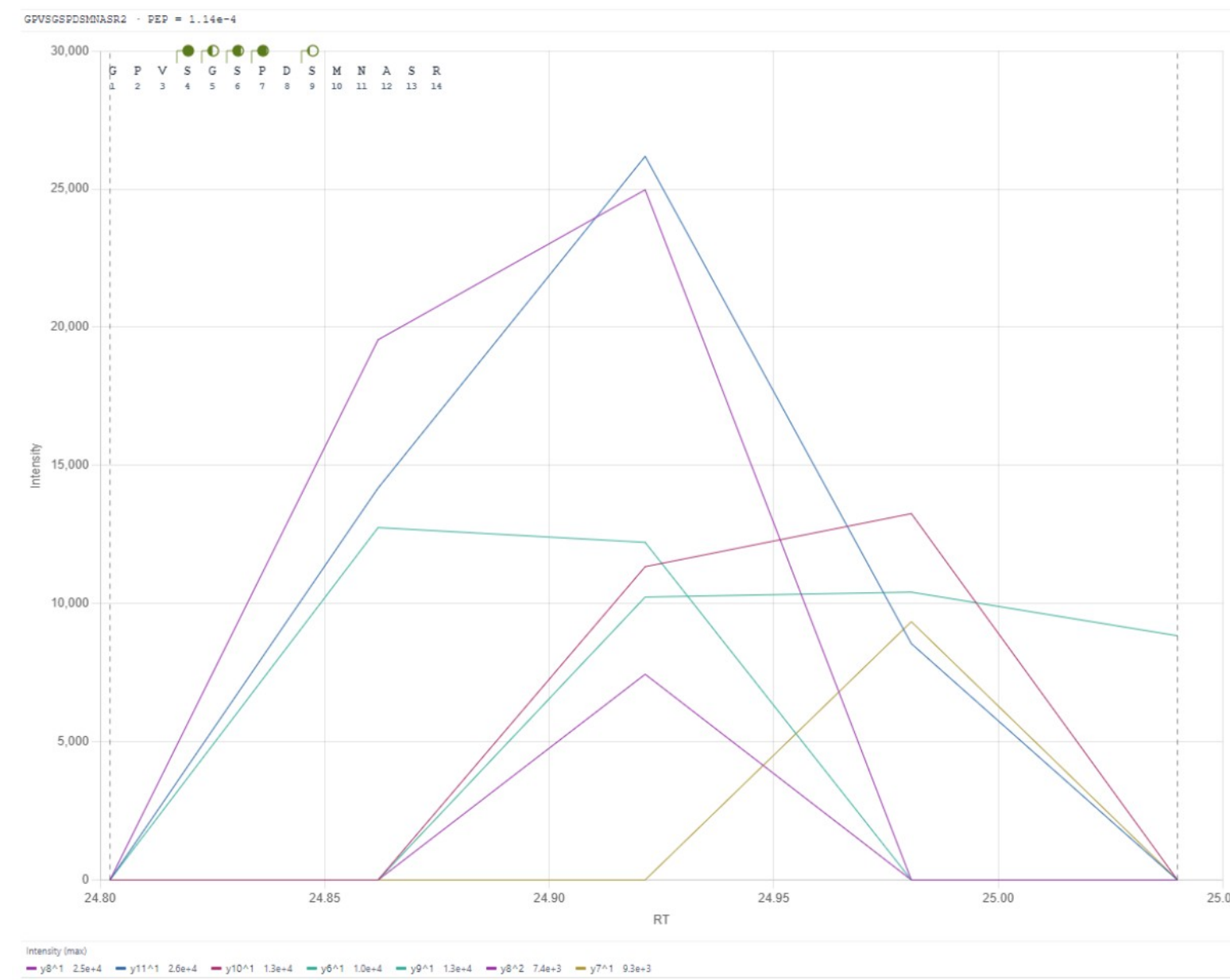

IRGQ

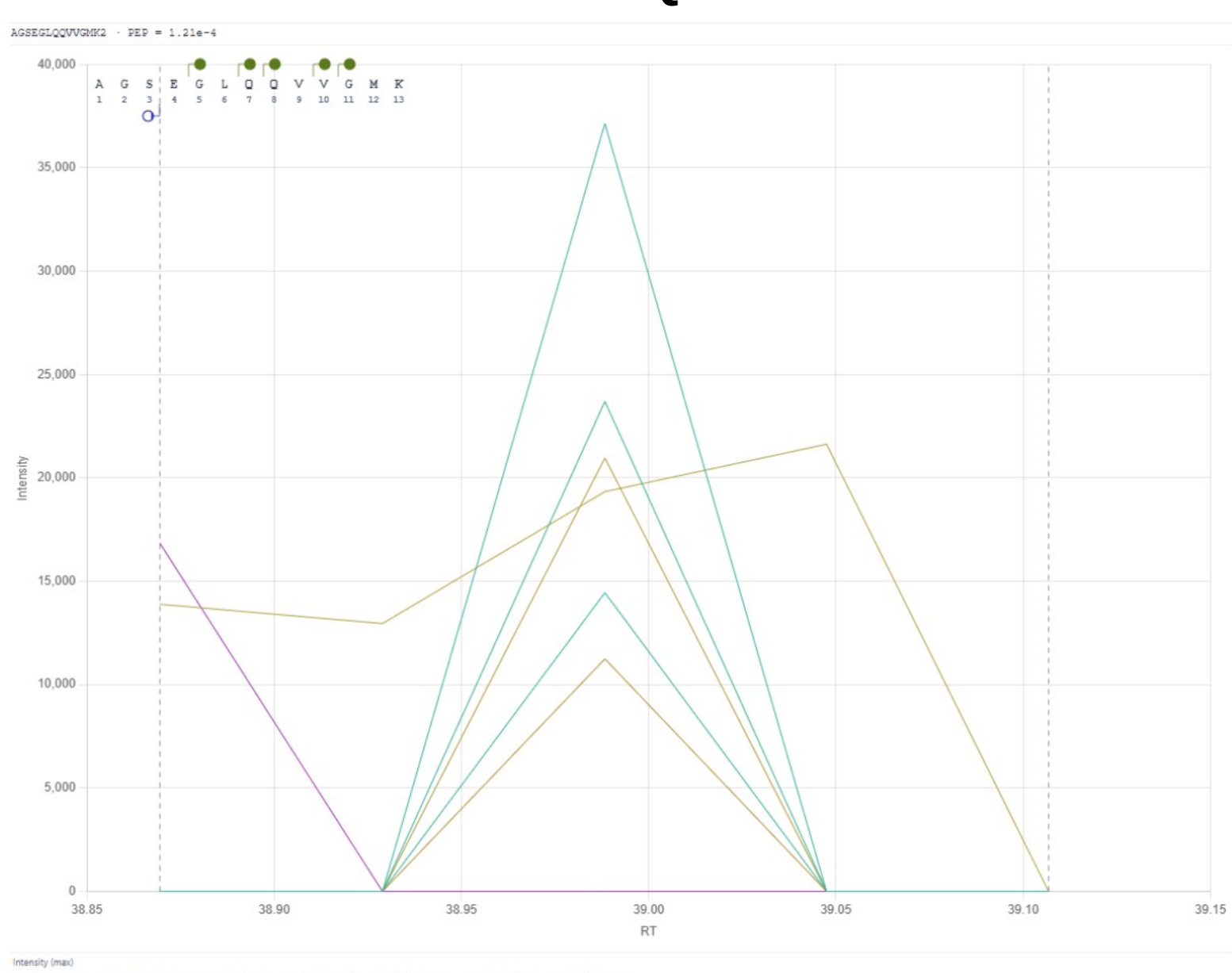

ITGB6

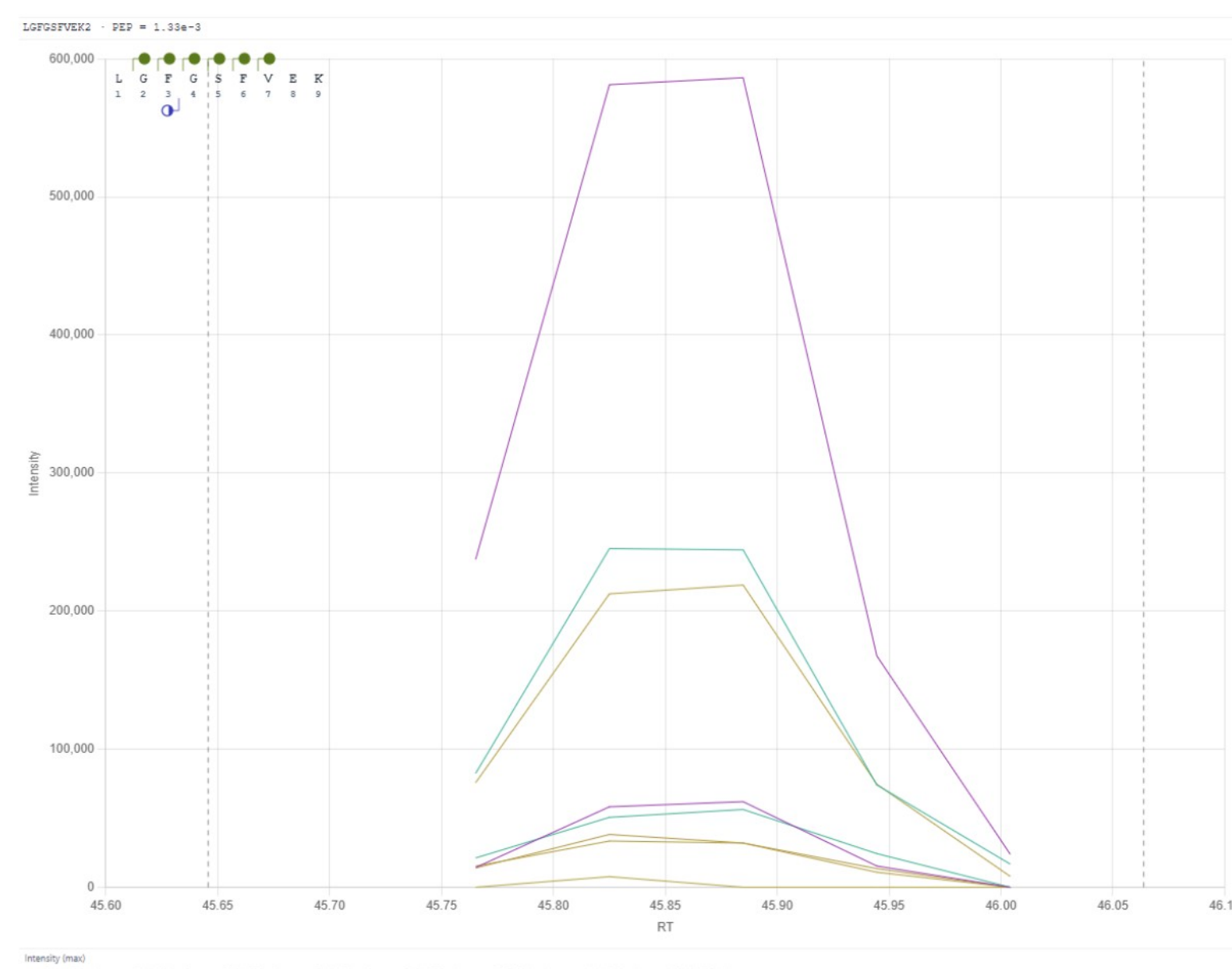

ITIH2

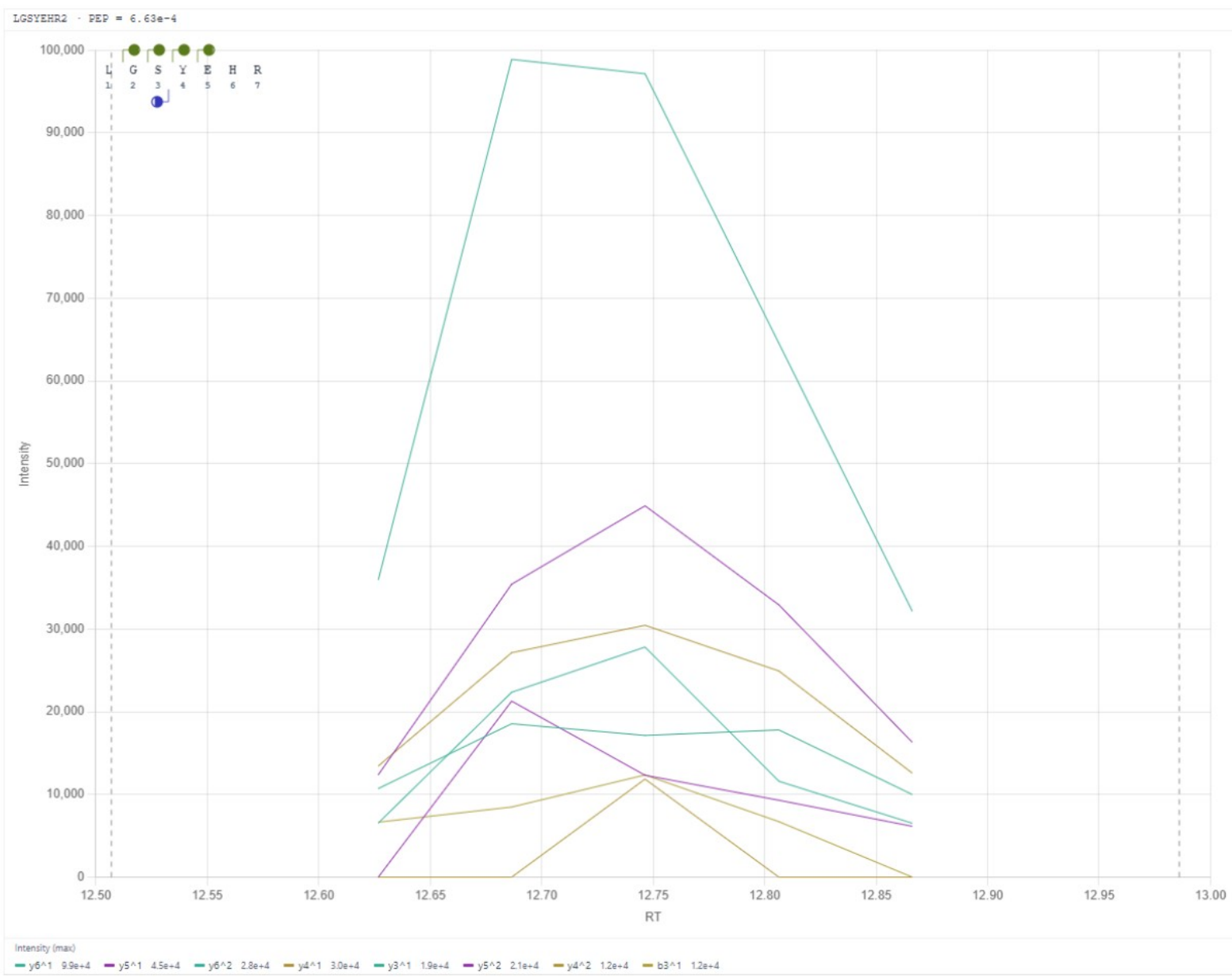

ITPR2

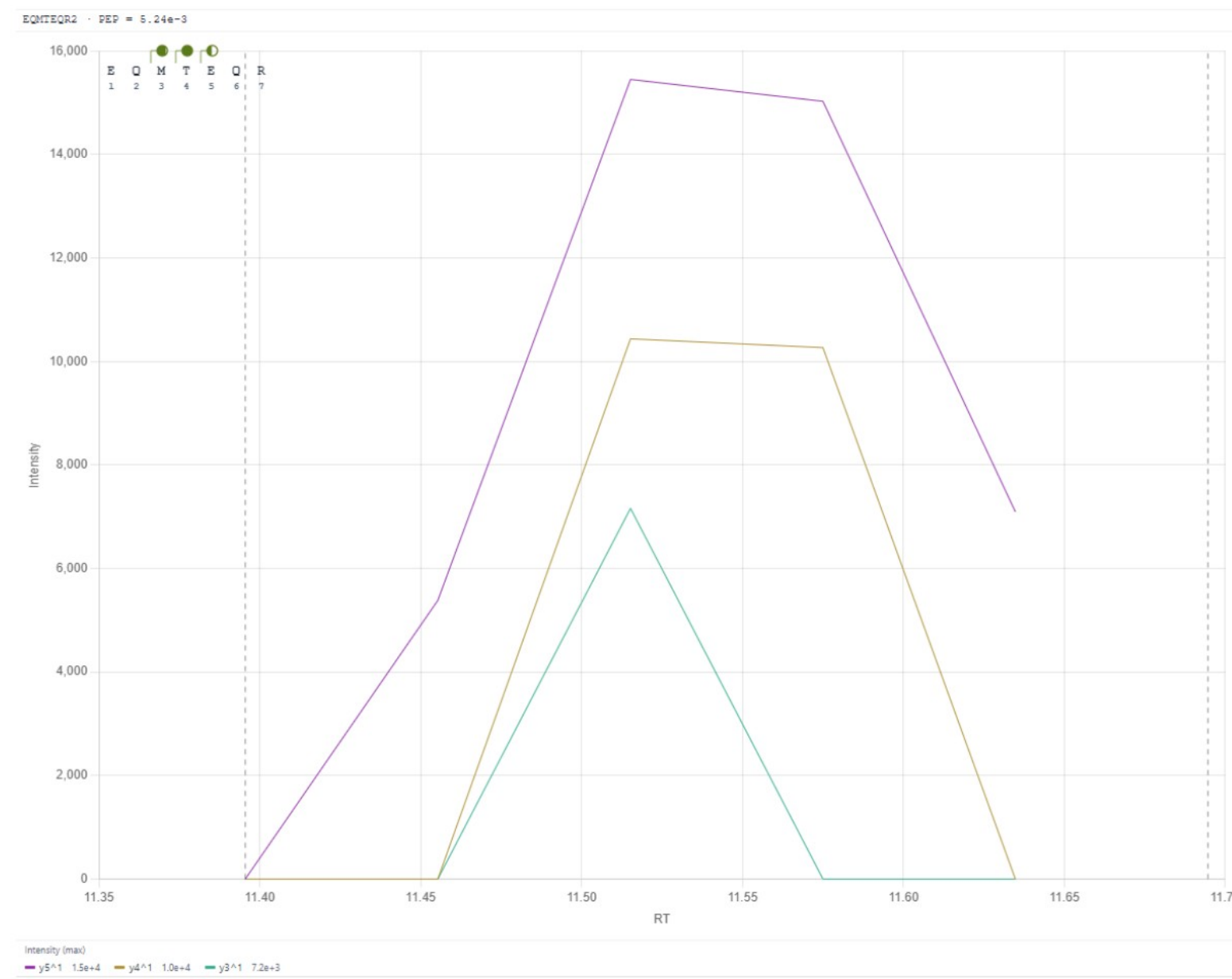

ITPRID2

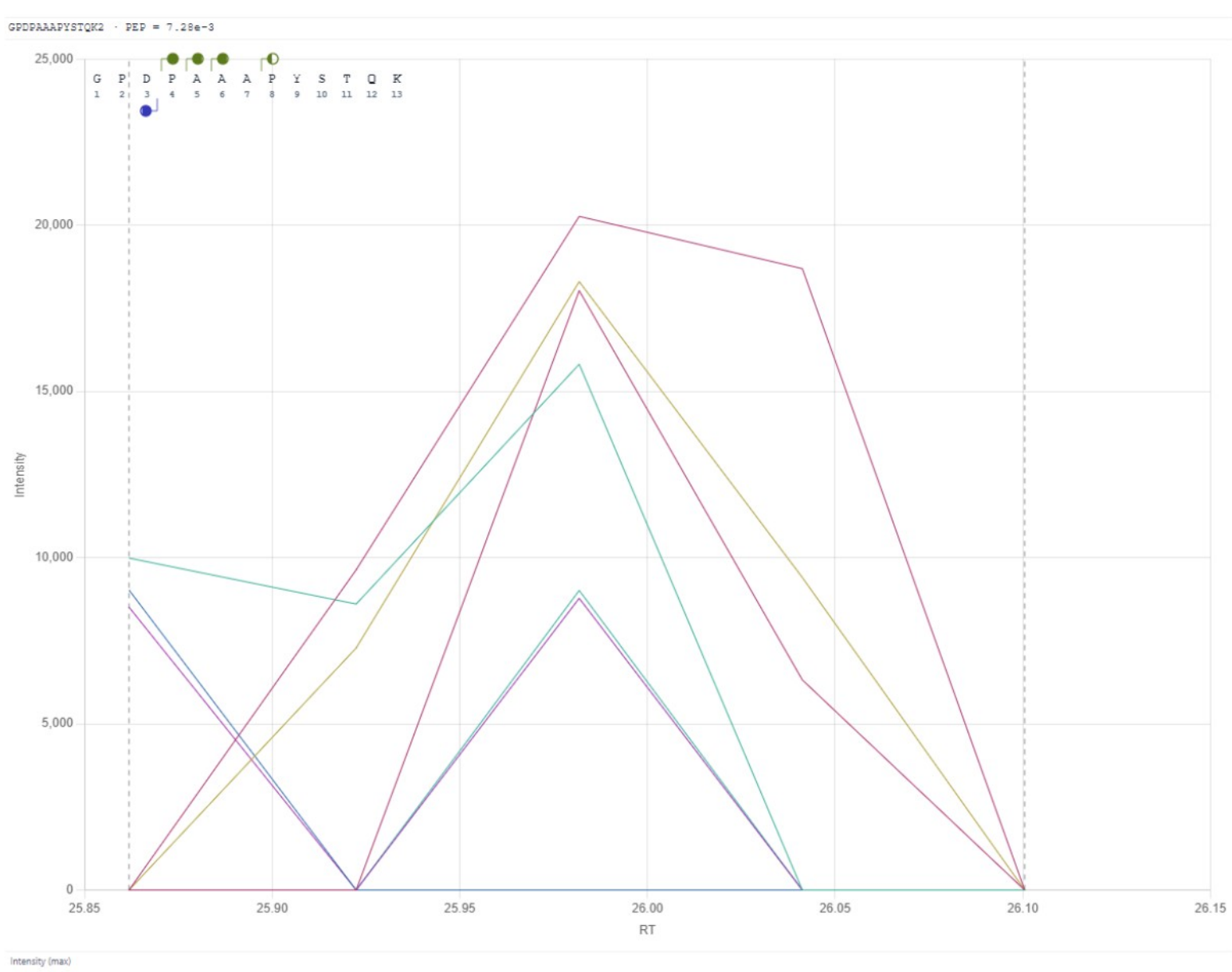

JAK3

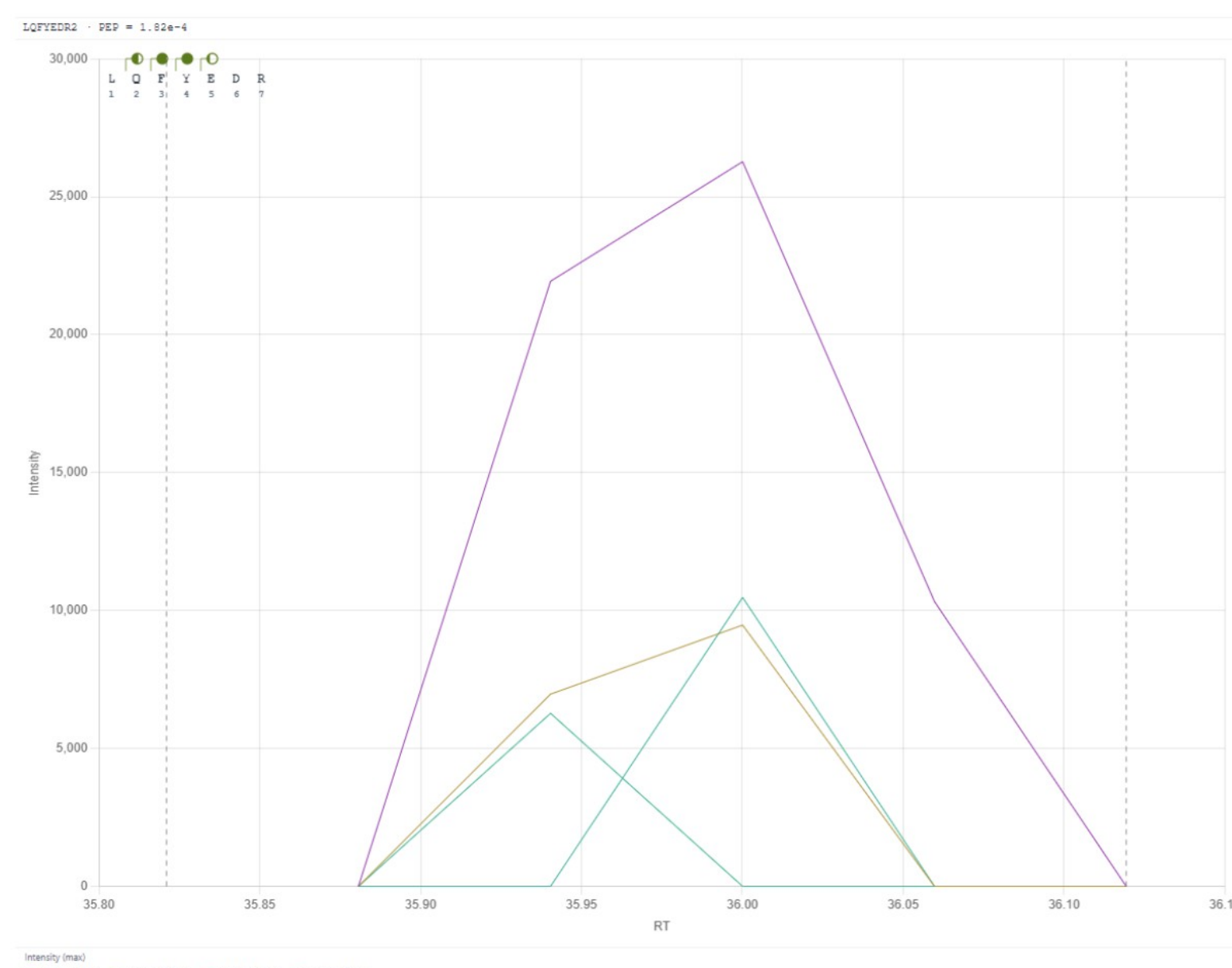

KATNAL2

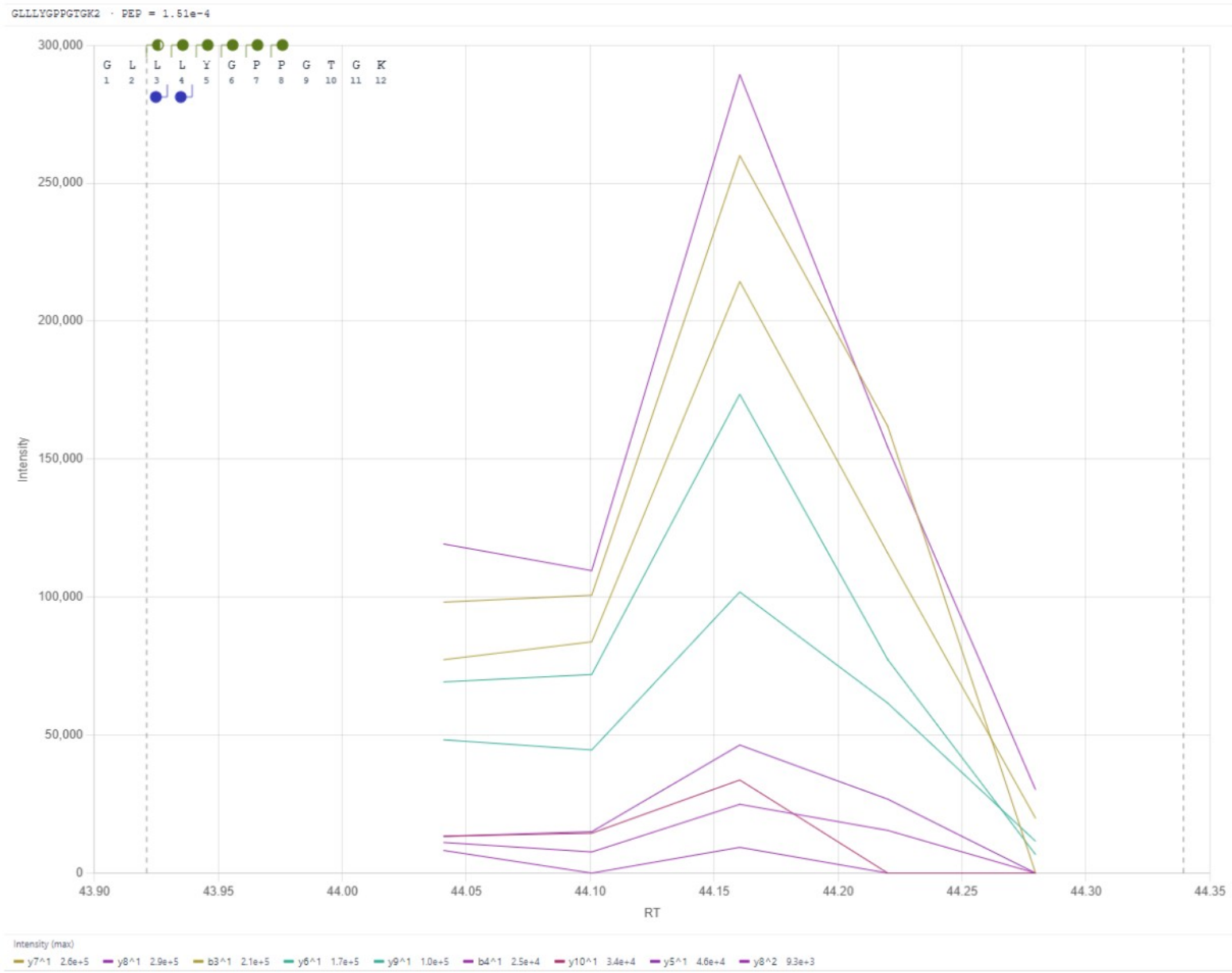

KIAA1958

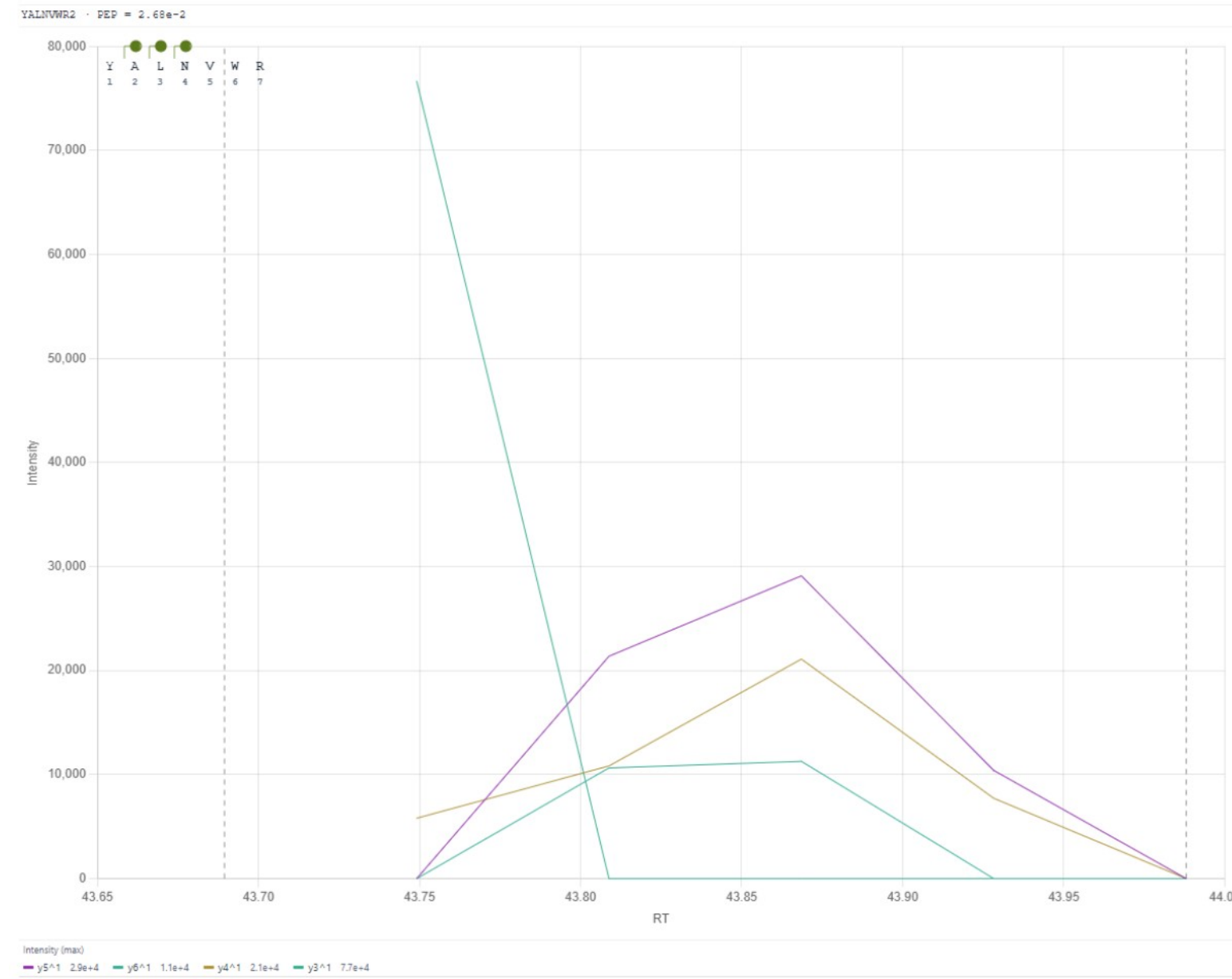

KIF20B

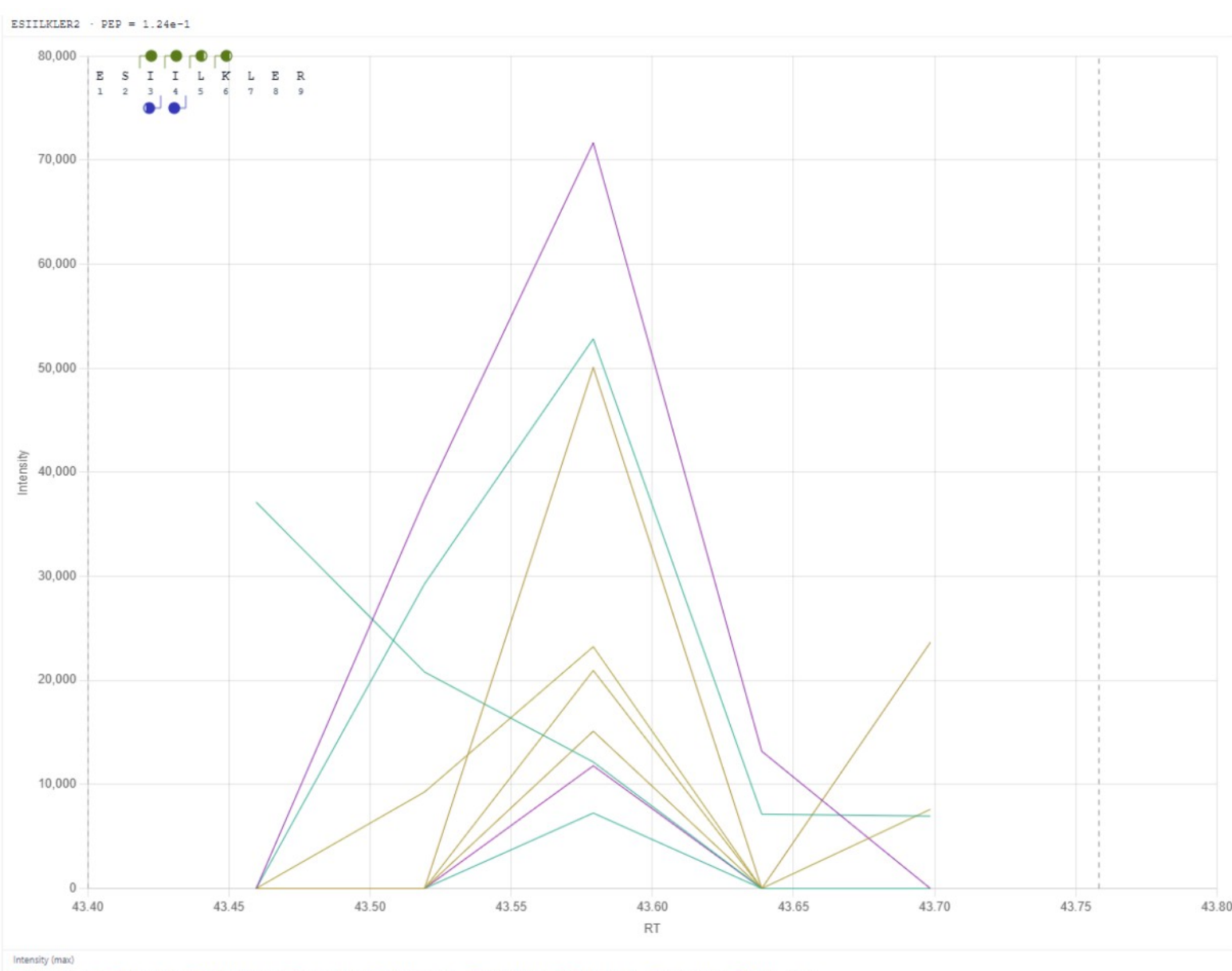

L2

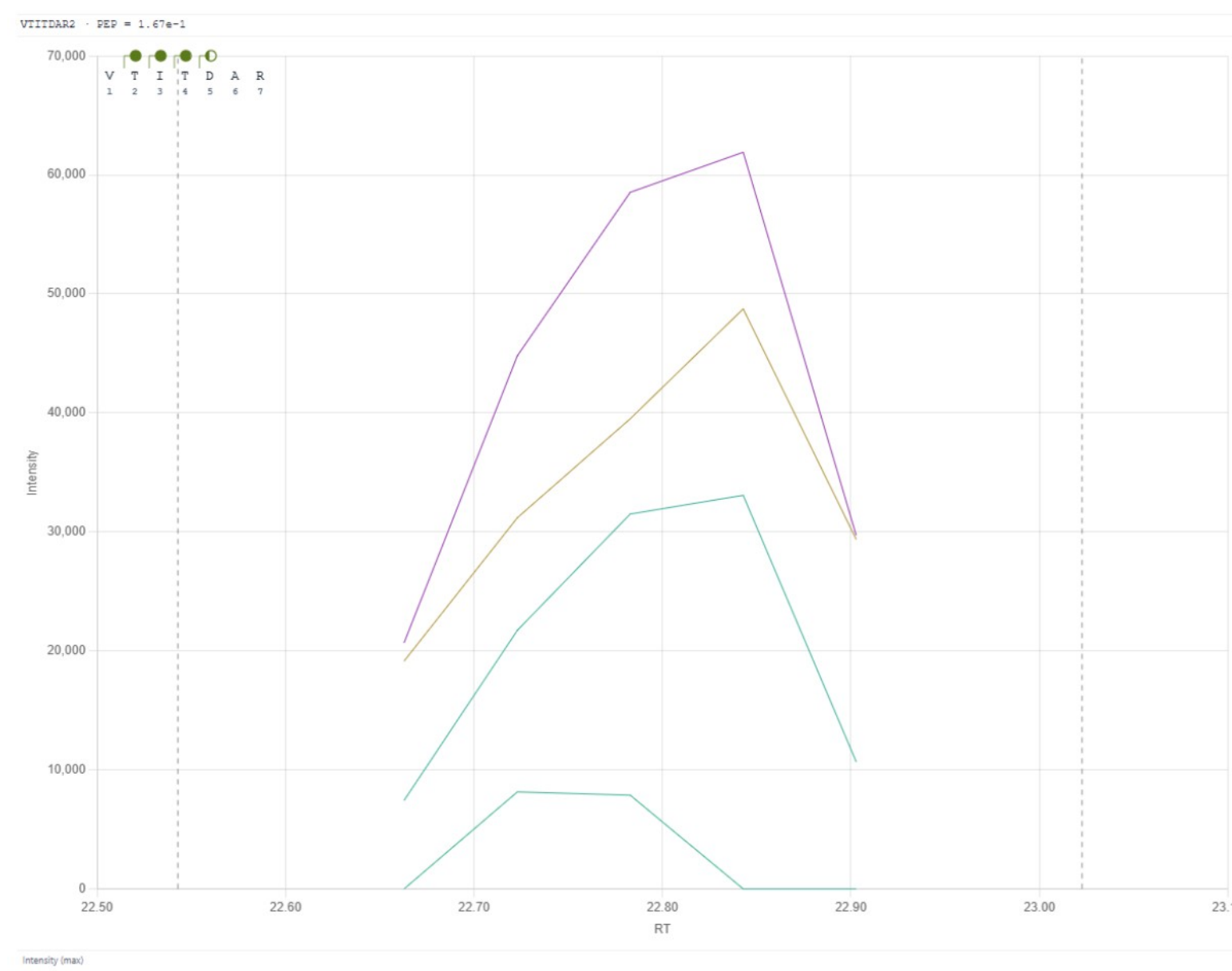

LAMP2

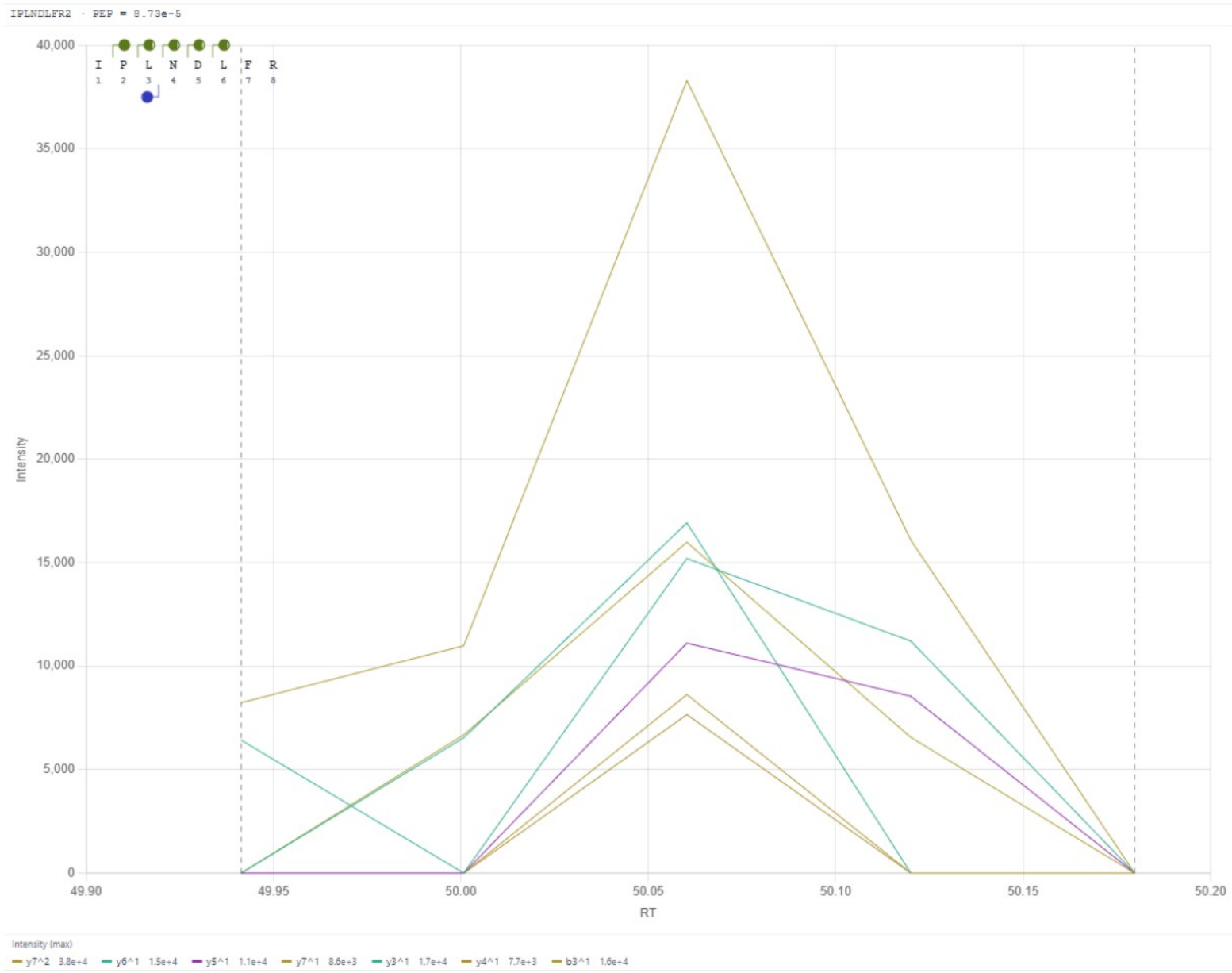

LAMTOR3

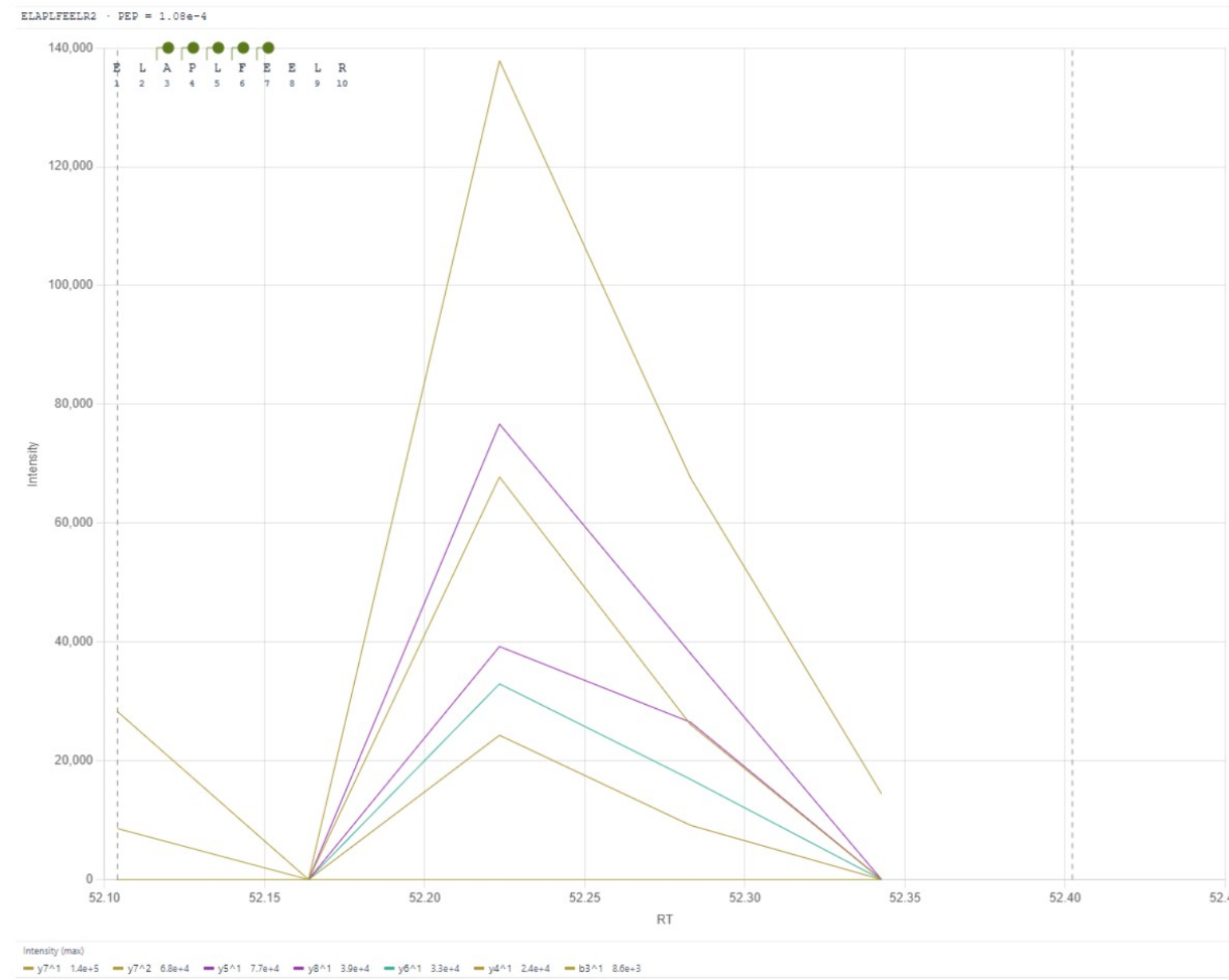

LEPROT

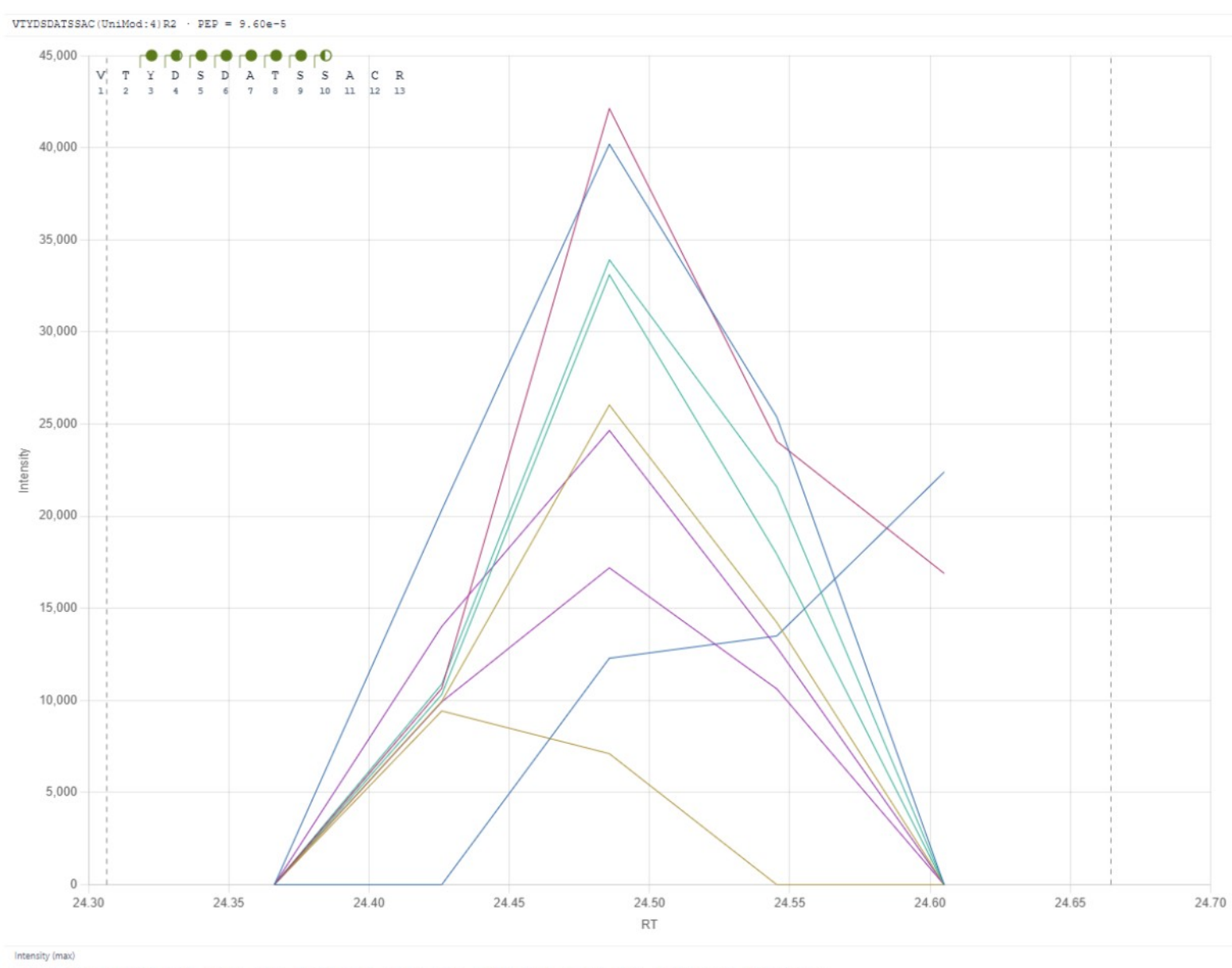

LIMA1

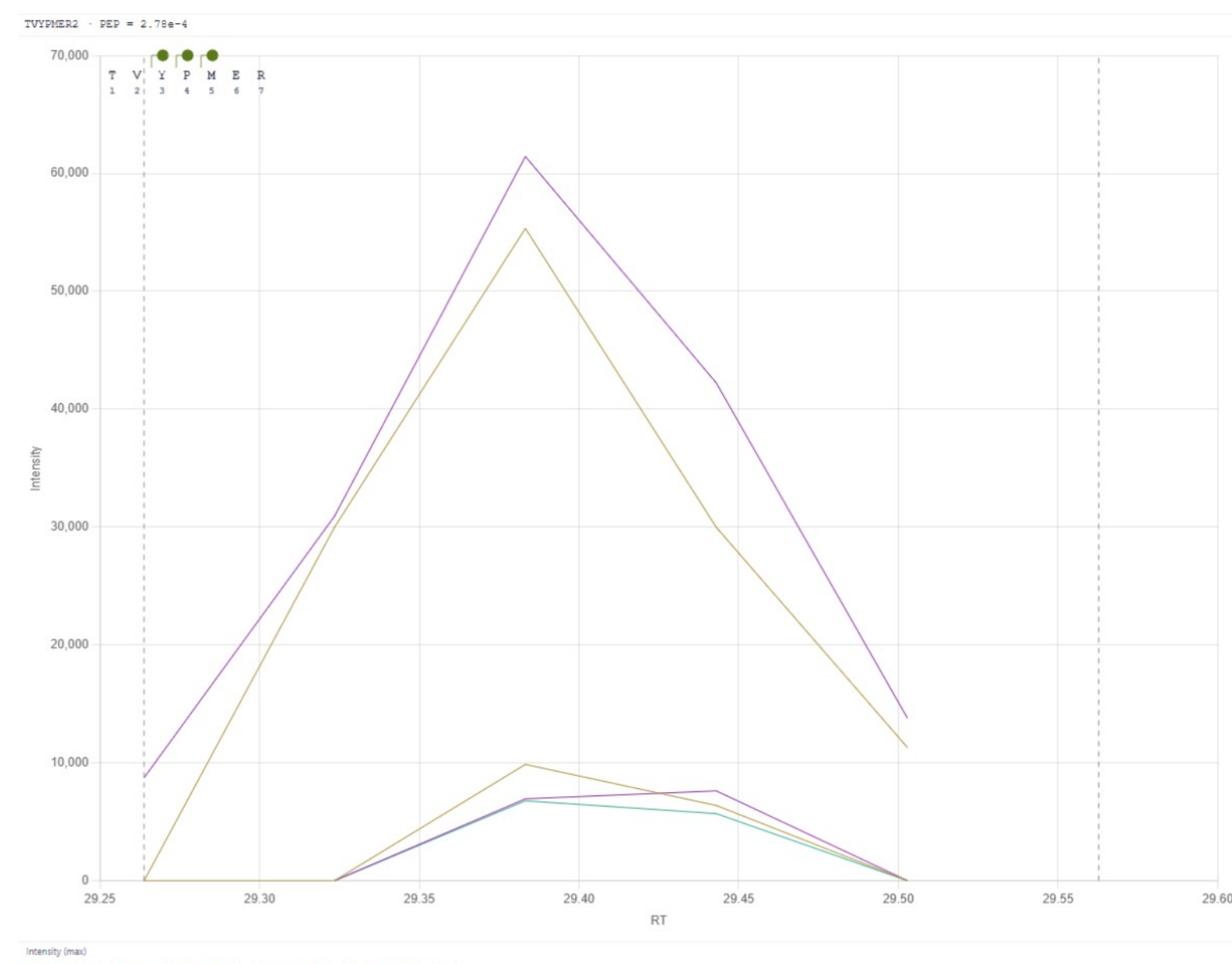

Lrrc23

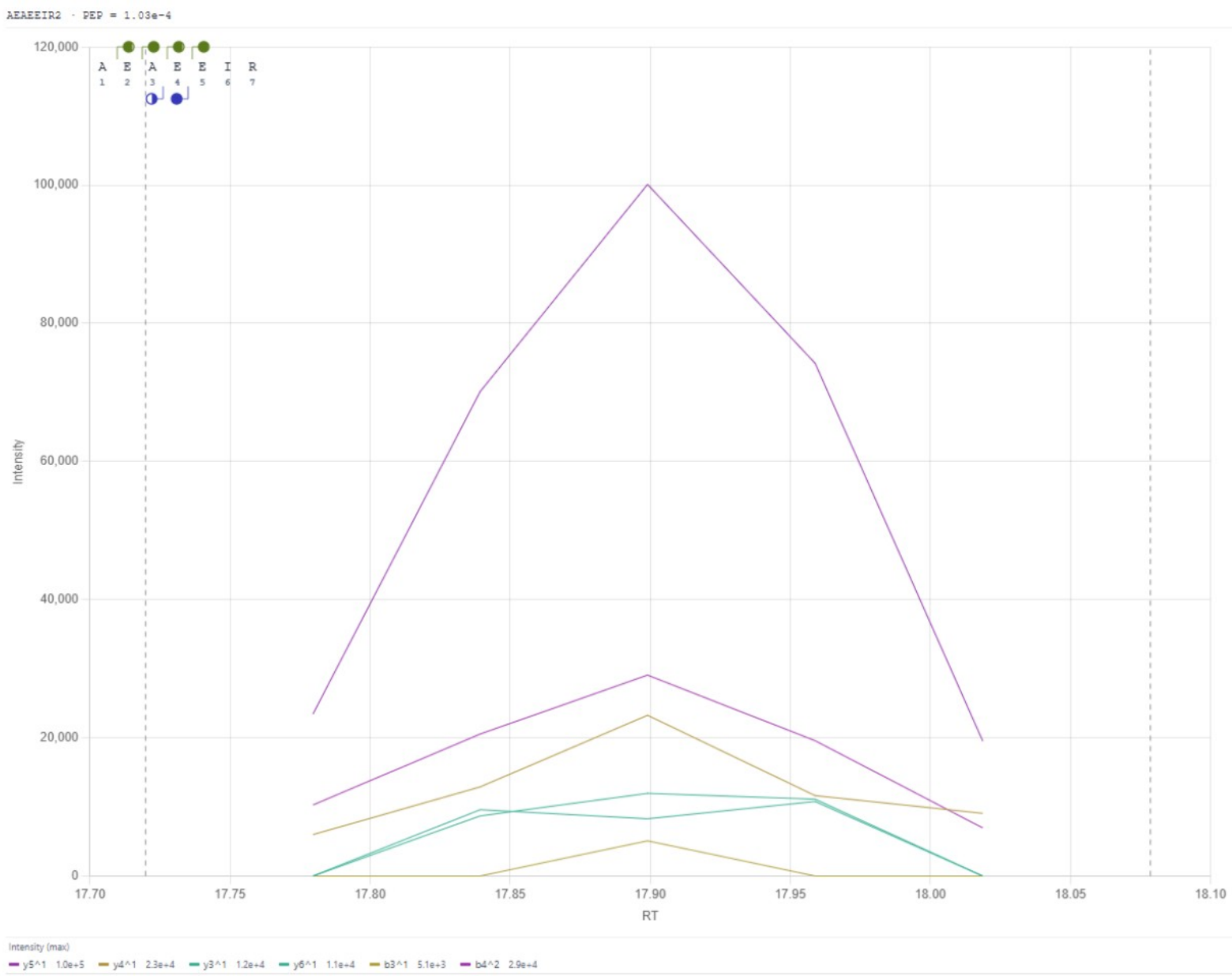

LRRC32

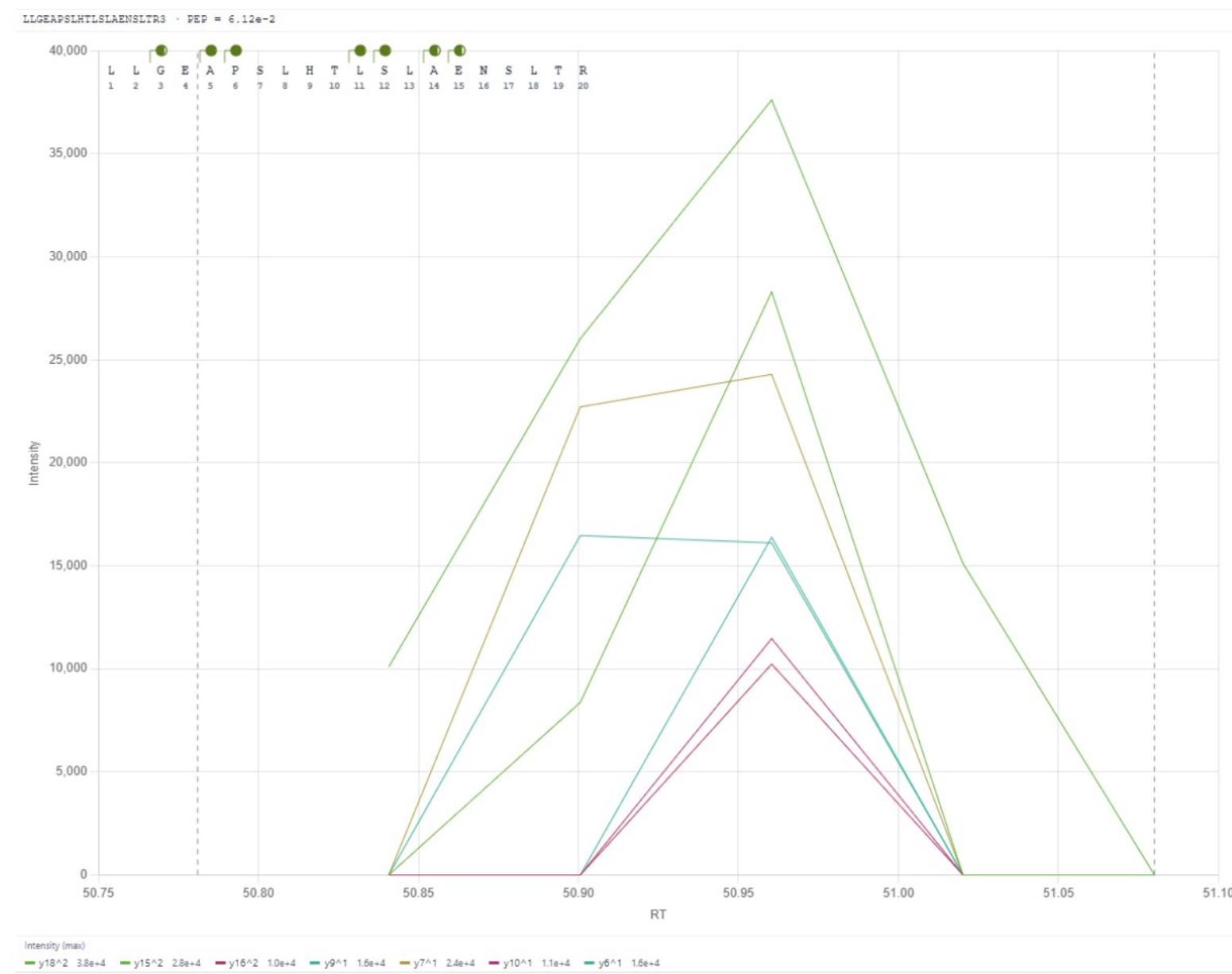

LRRFIP1

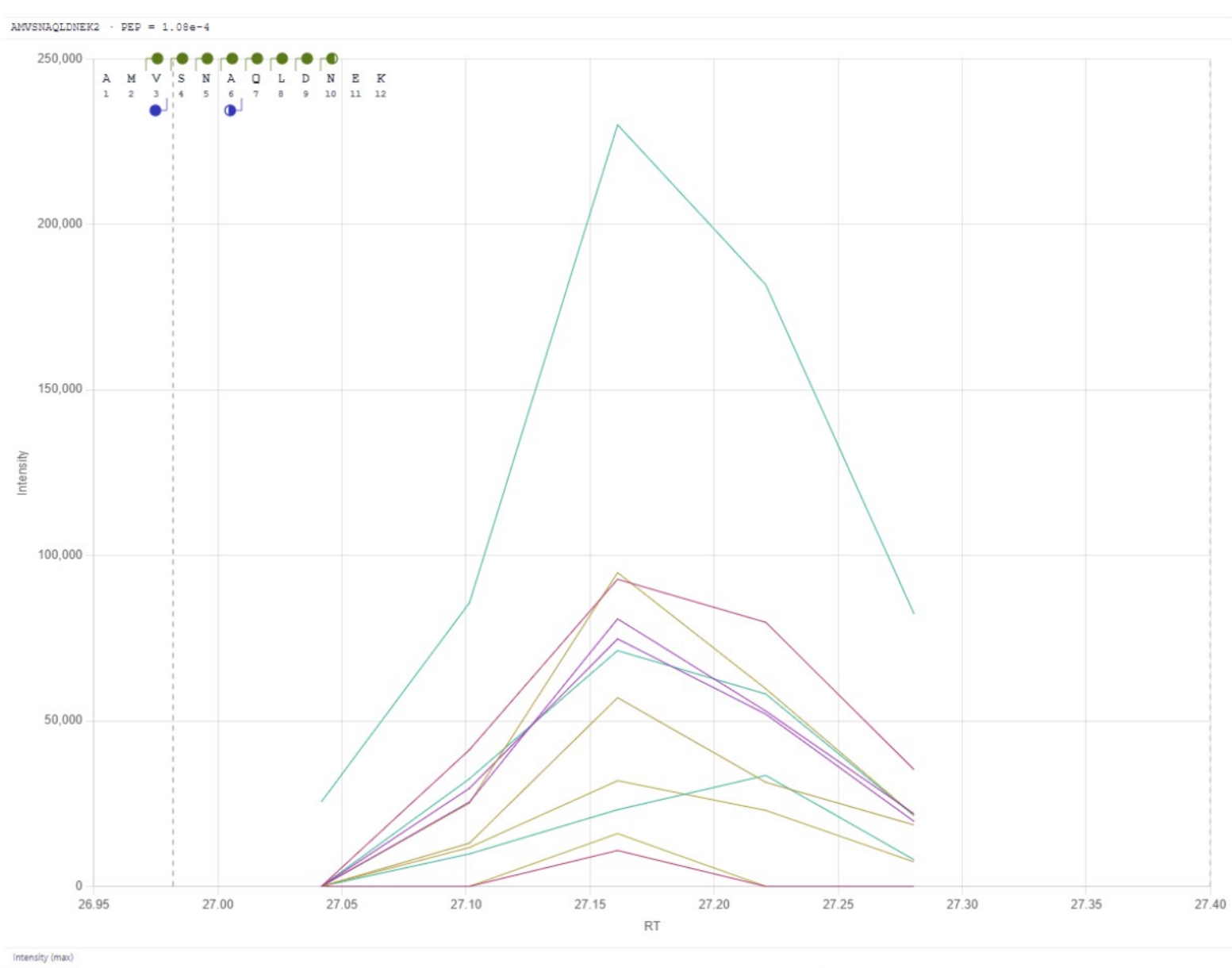

LSM3

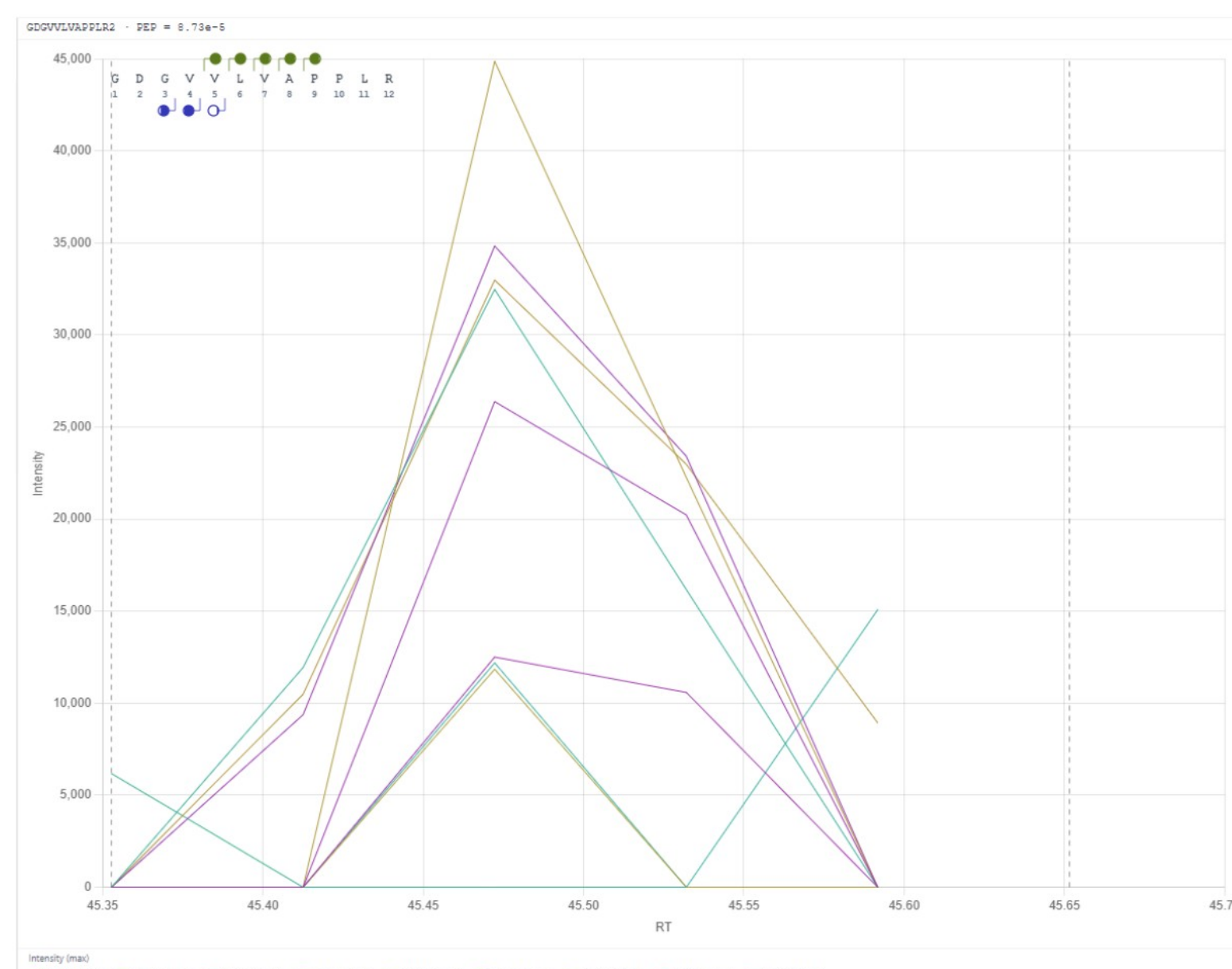

TF

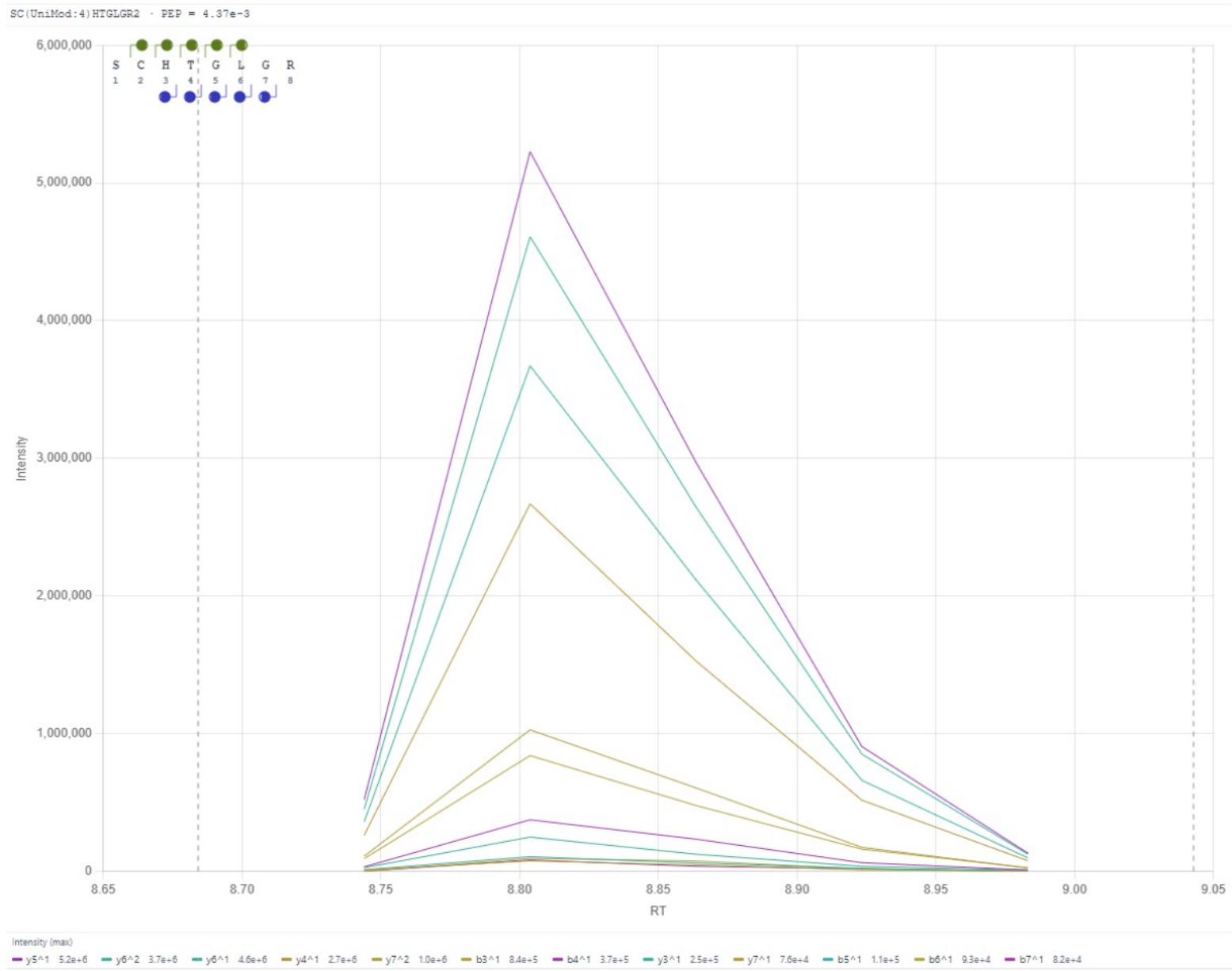

MAN2A1

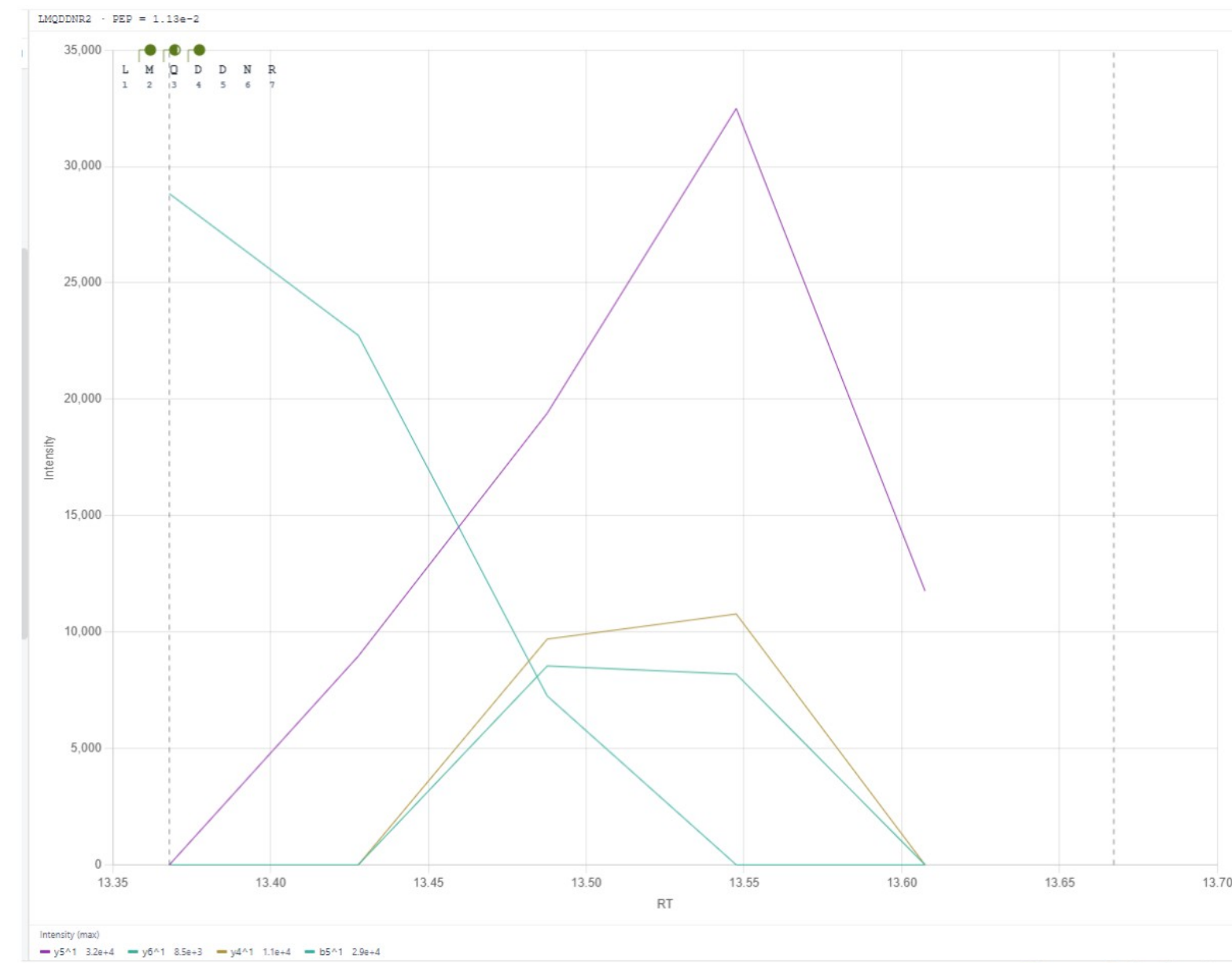

MAP1A

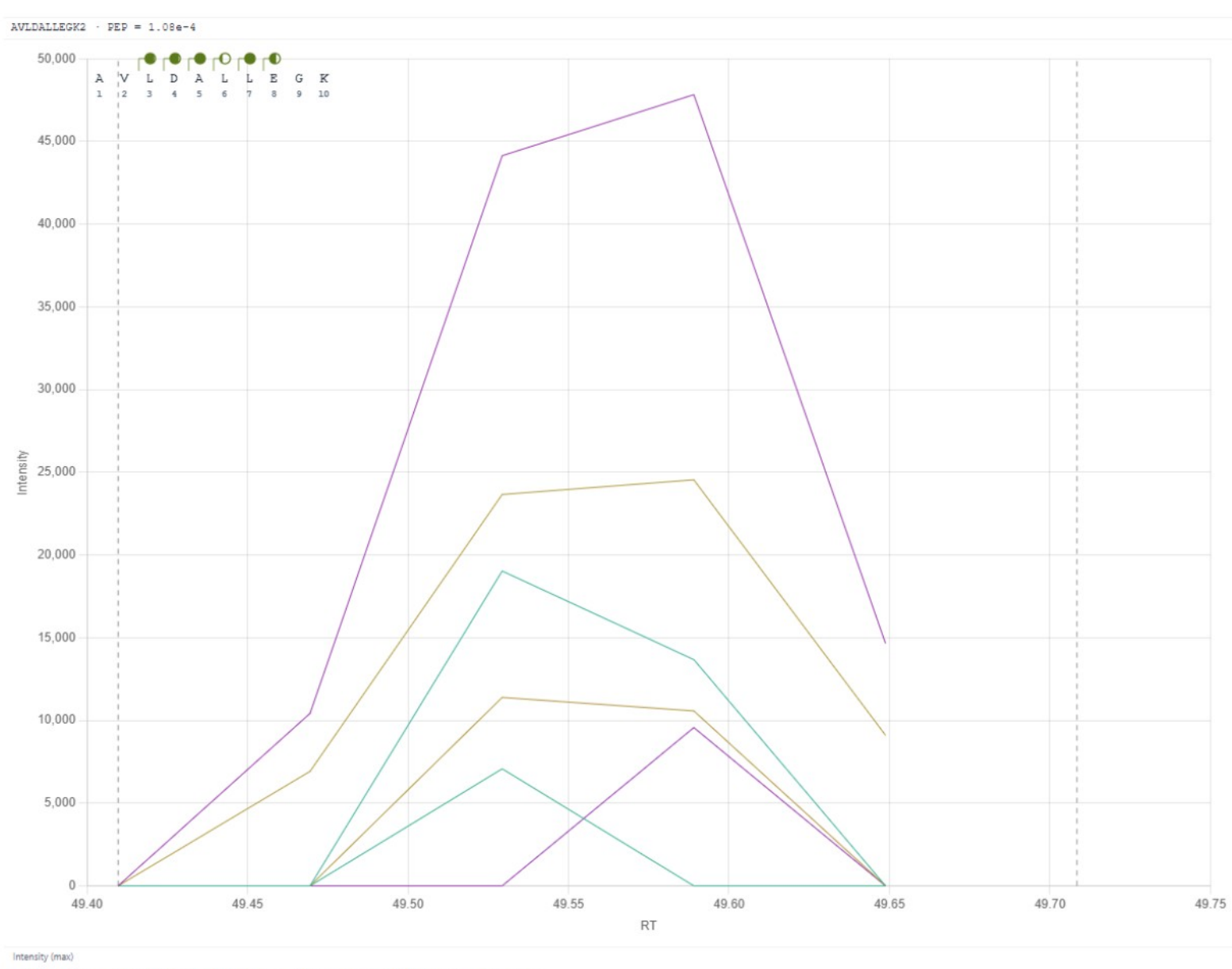

MAPRE1

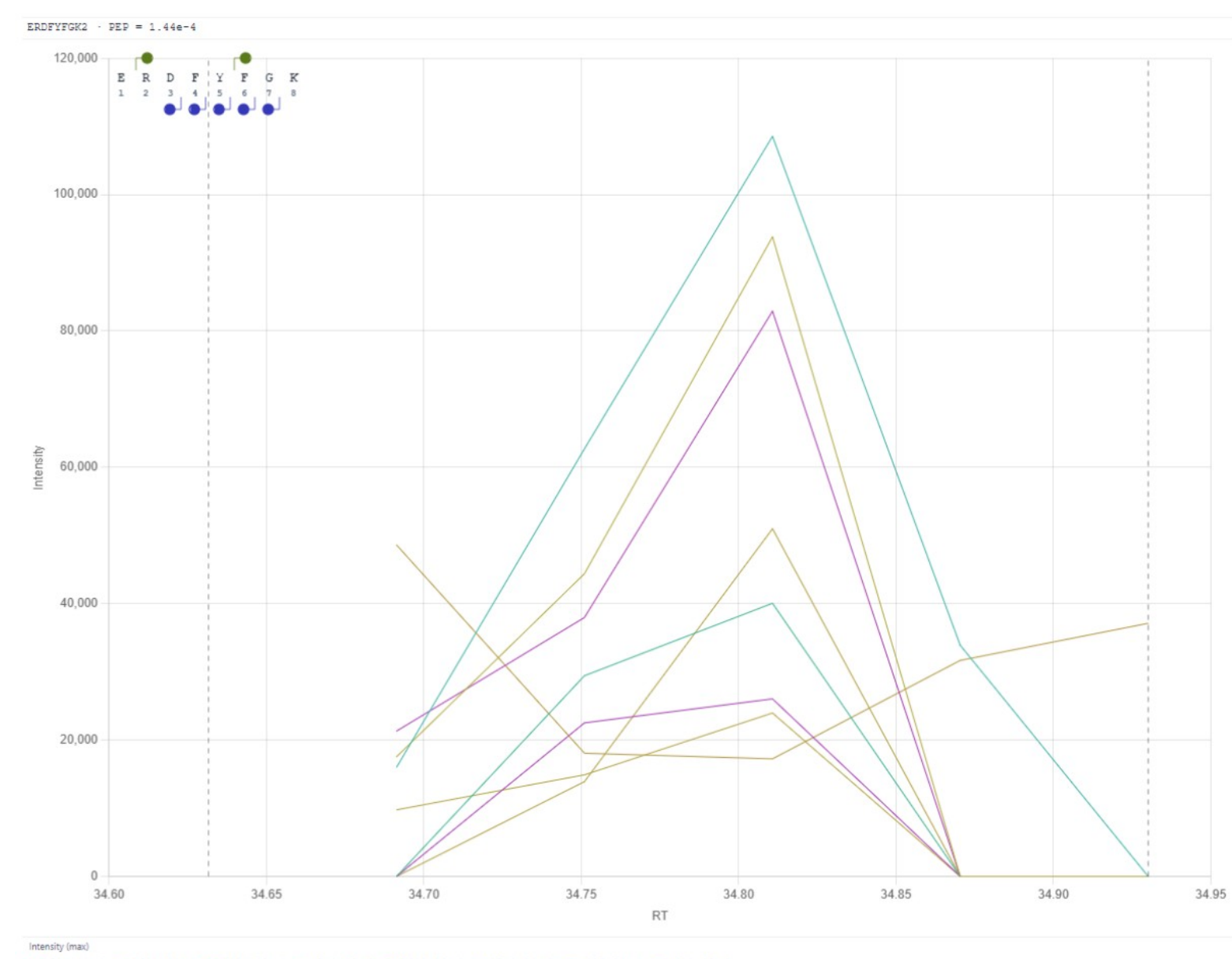

MARK2

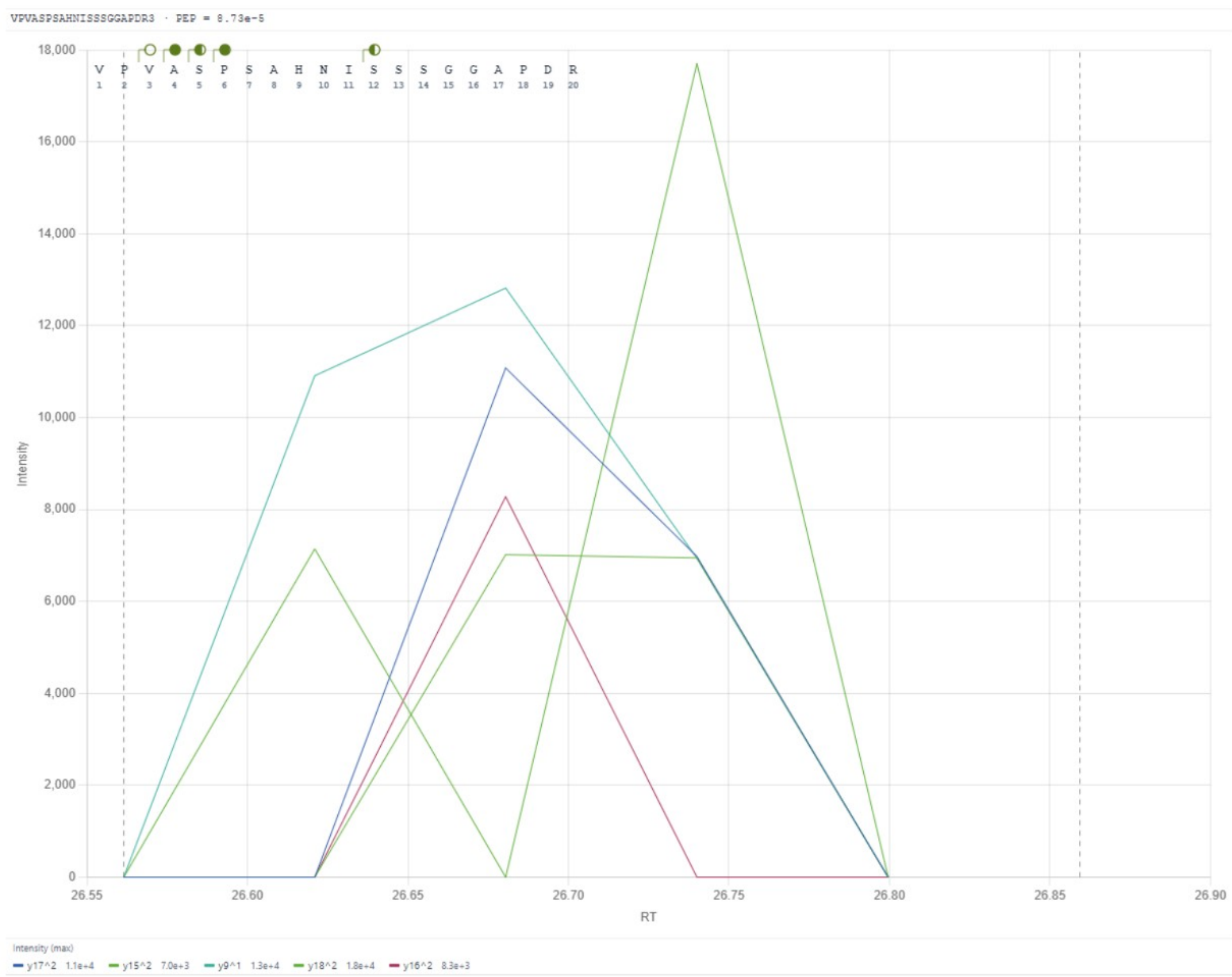

MCFD2

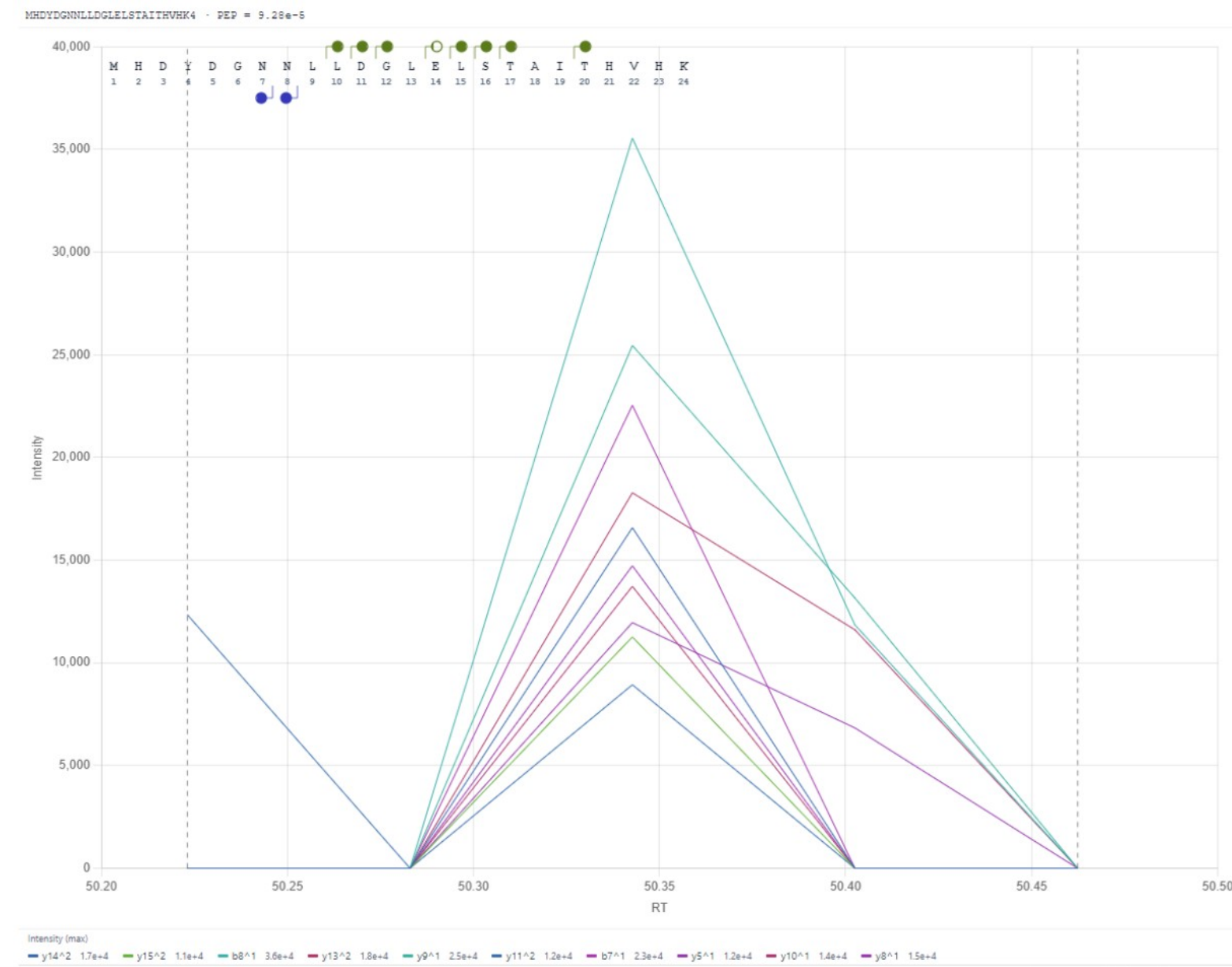

MDV1

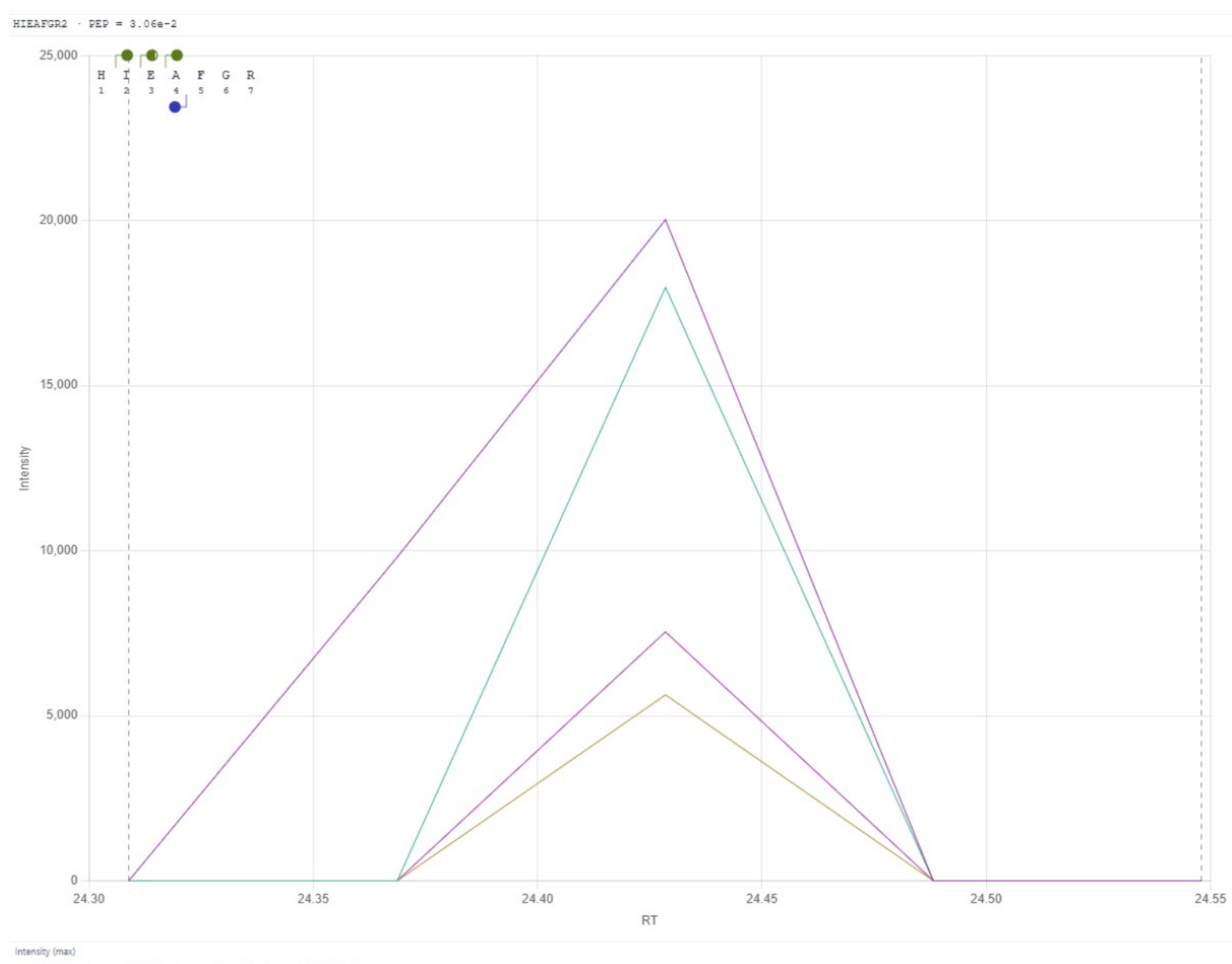

MFSD6

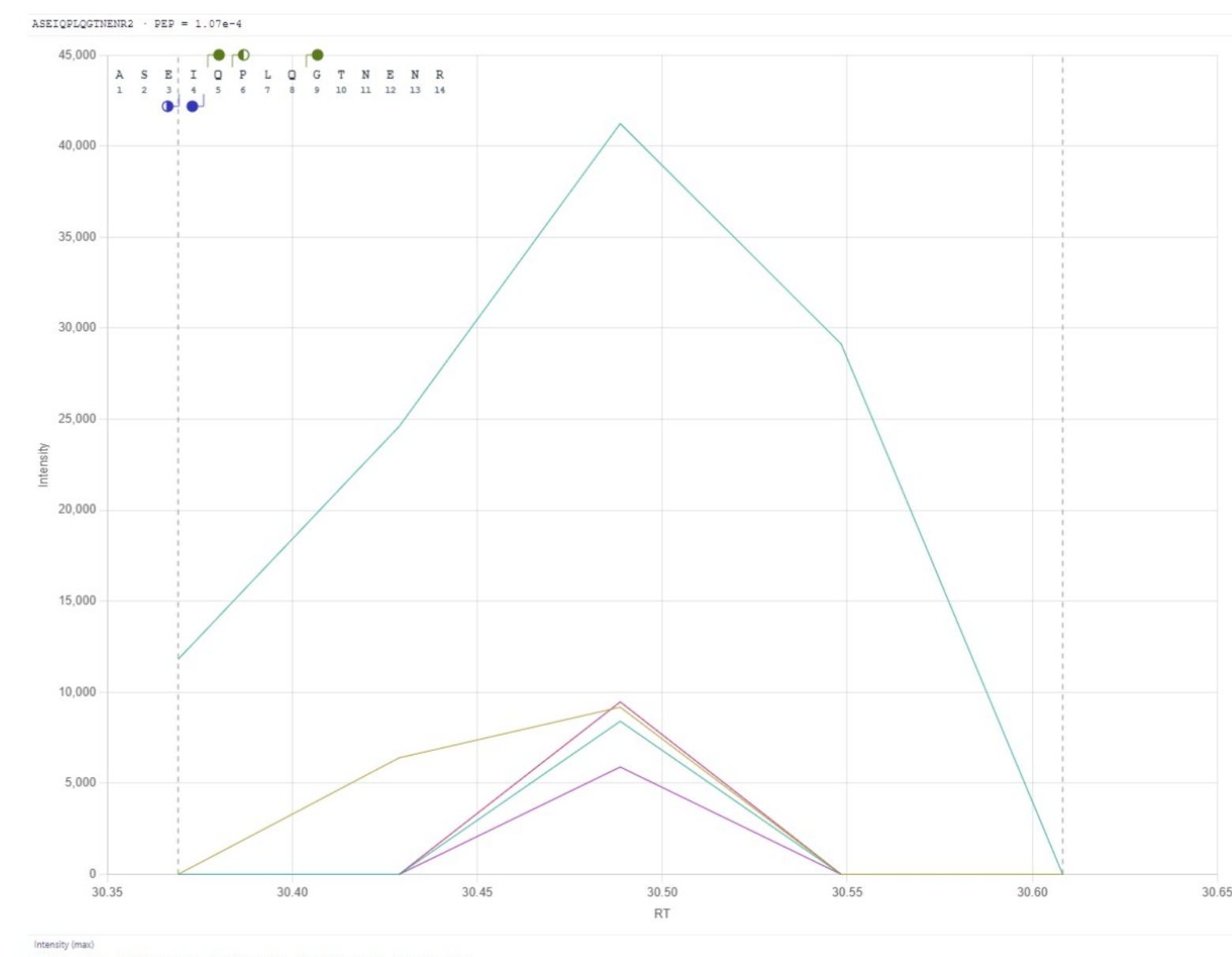

MGAT2

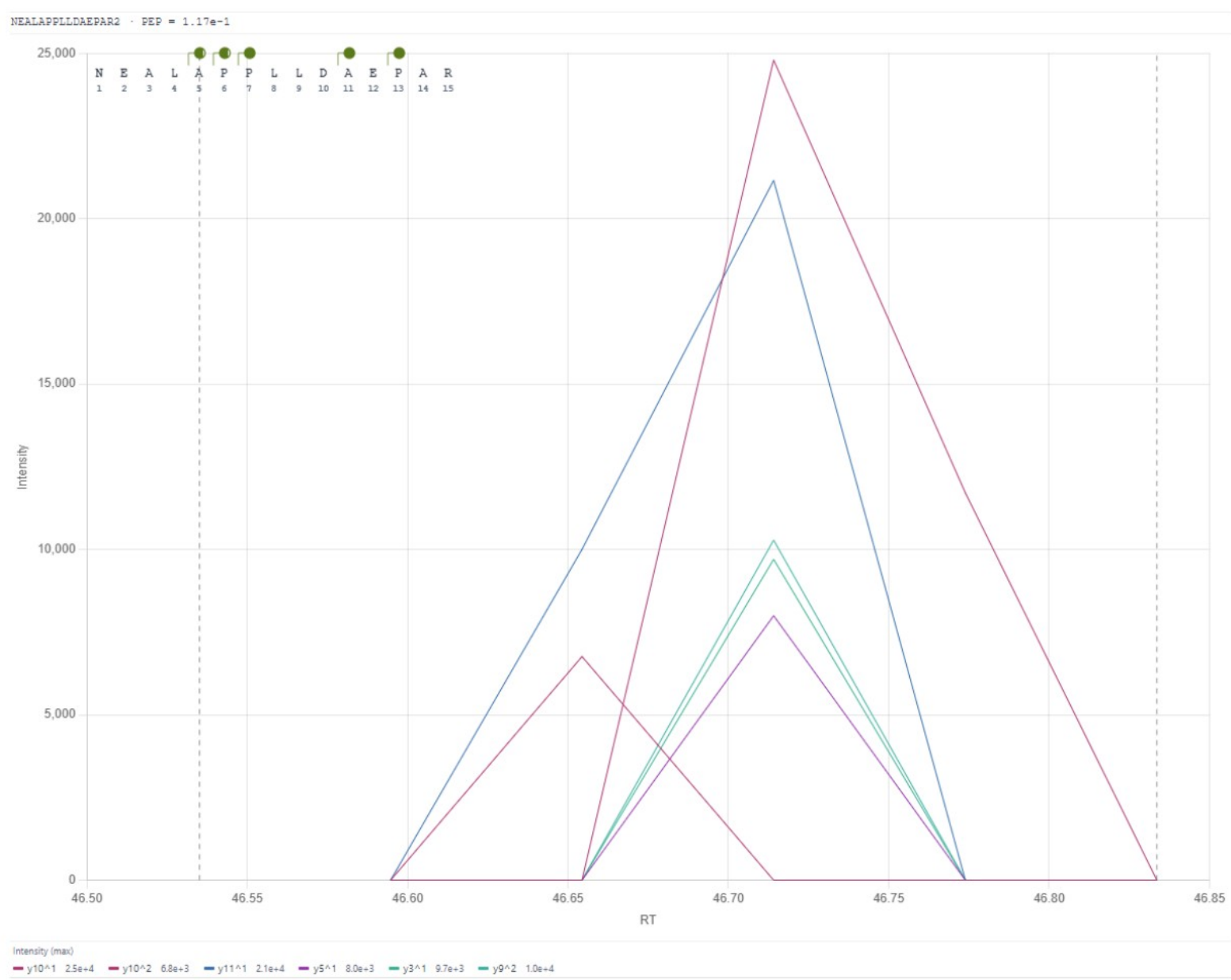

MGST2

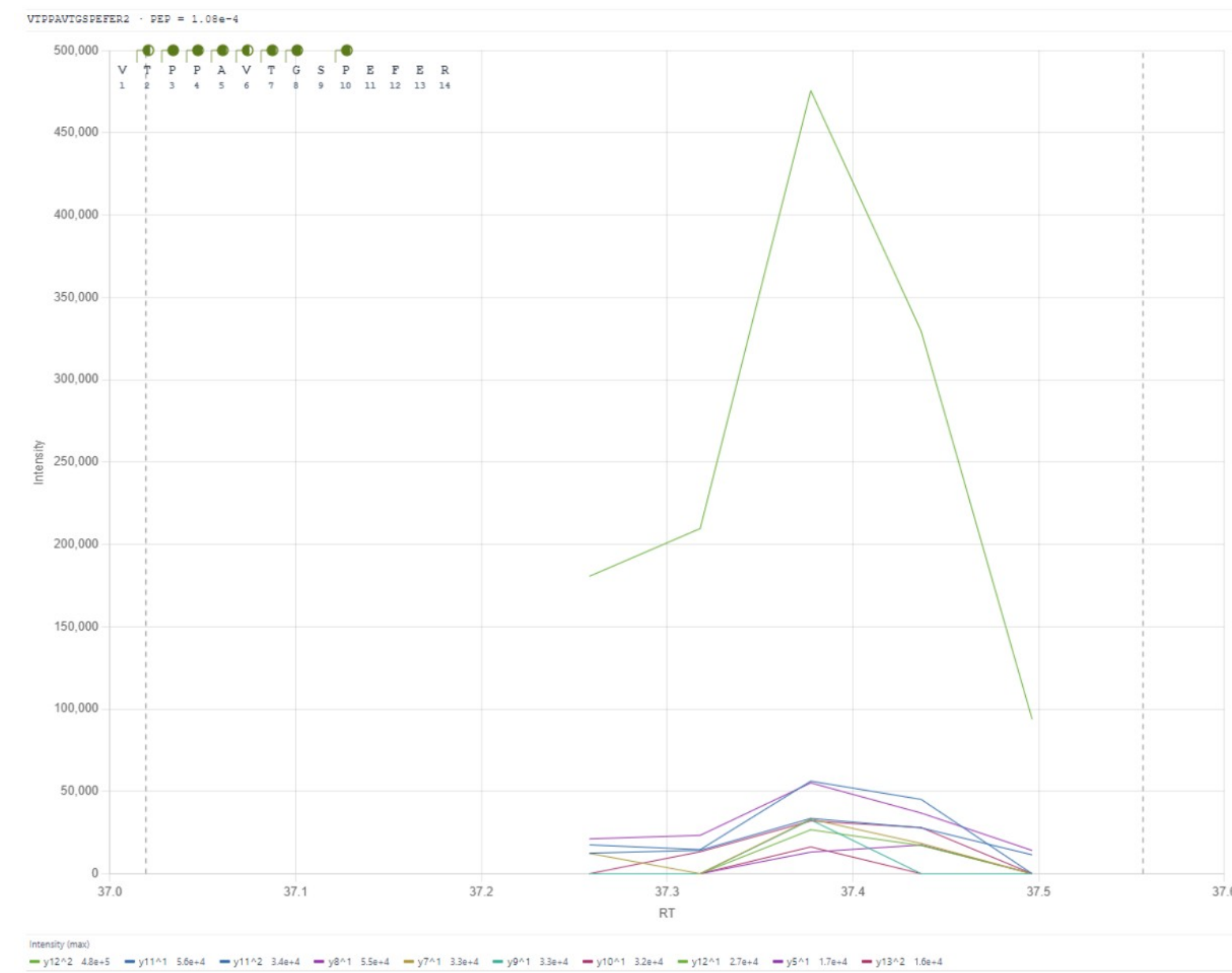

MIEN1

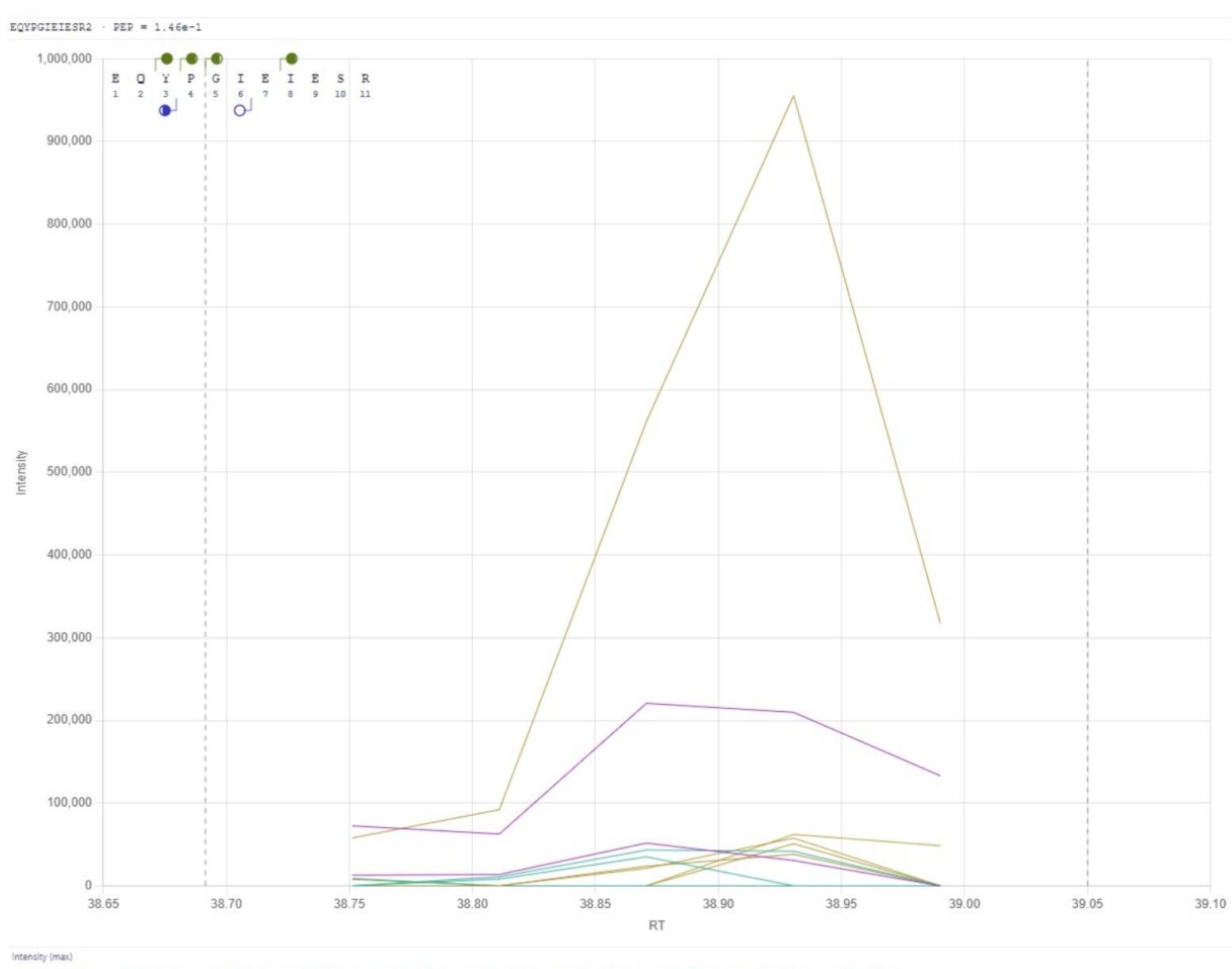

MTM1

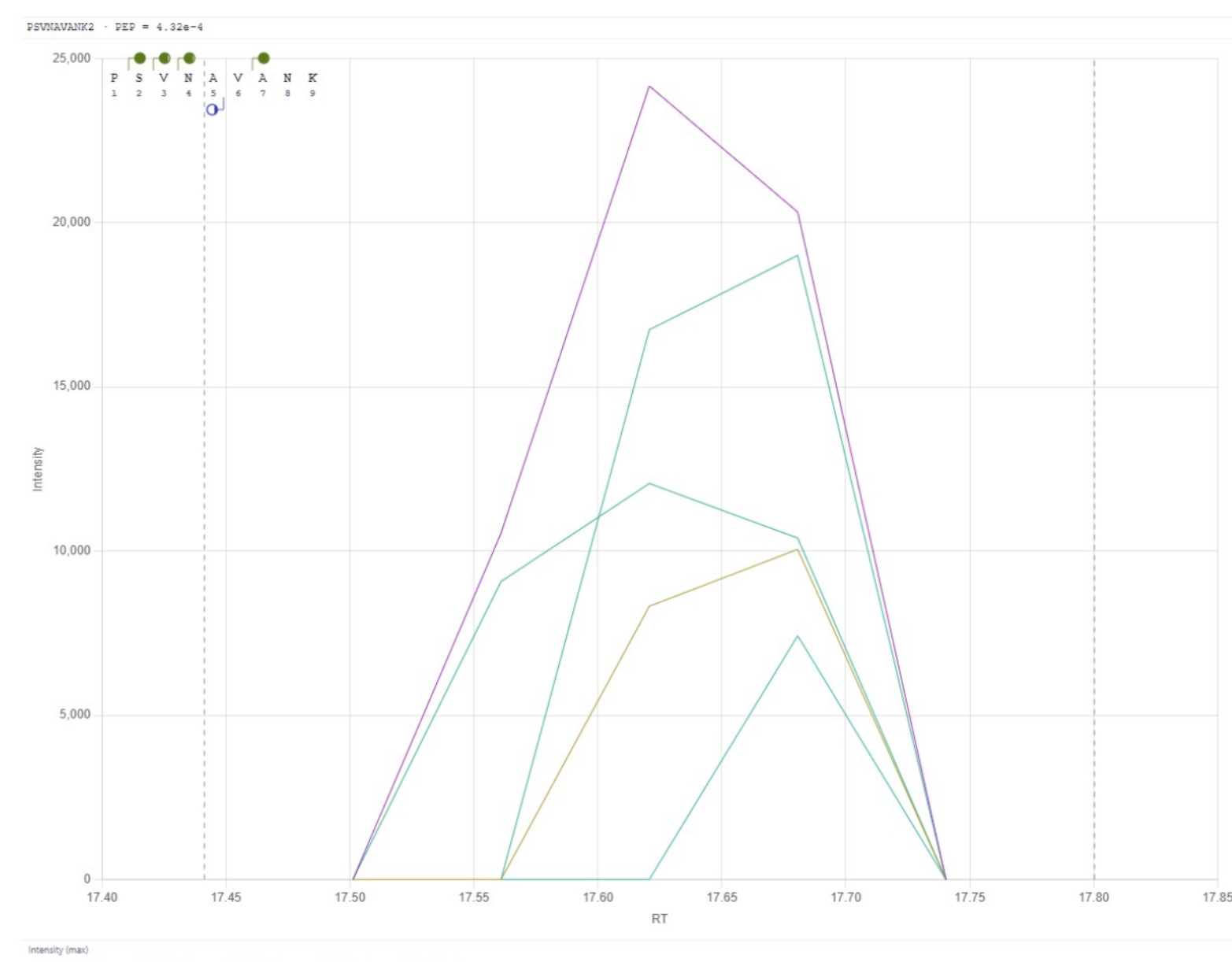

MTURN

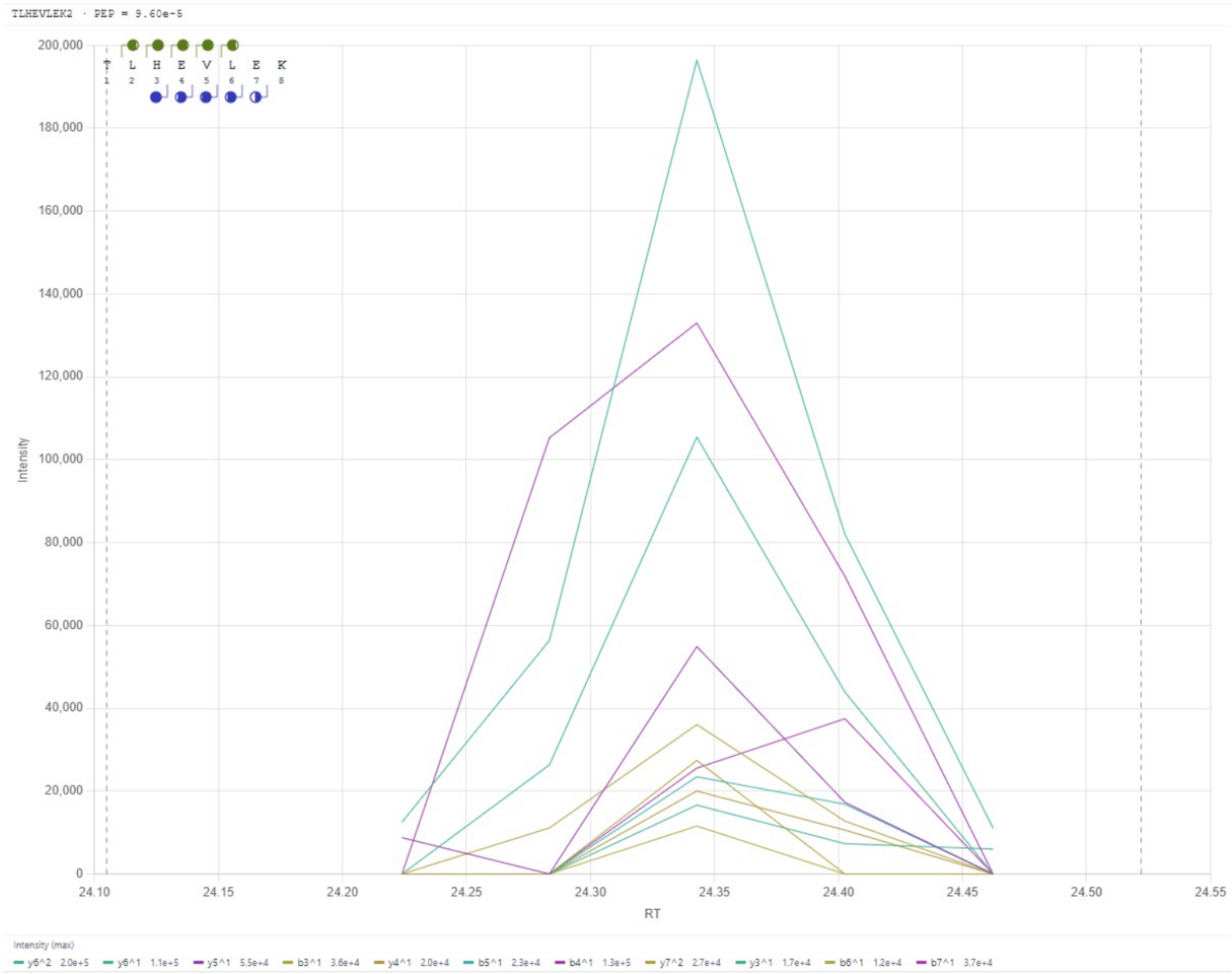

MYG1

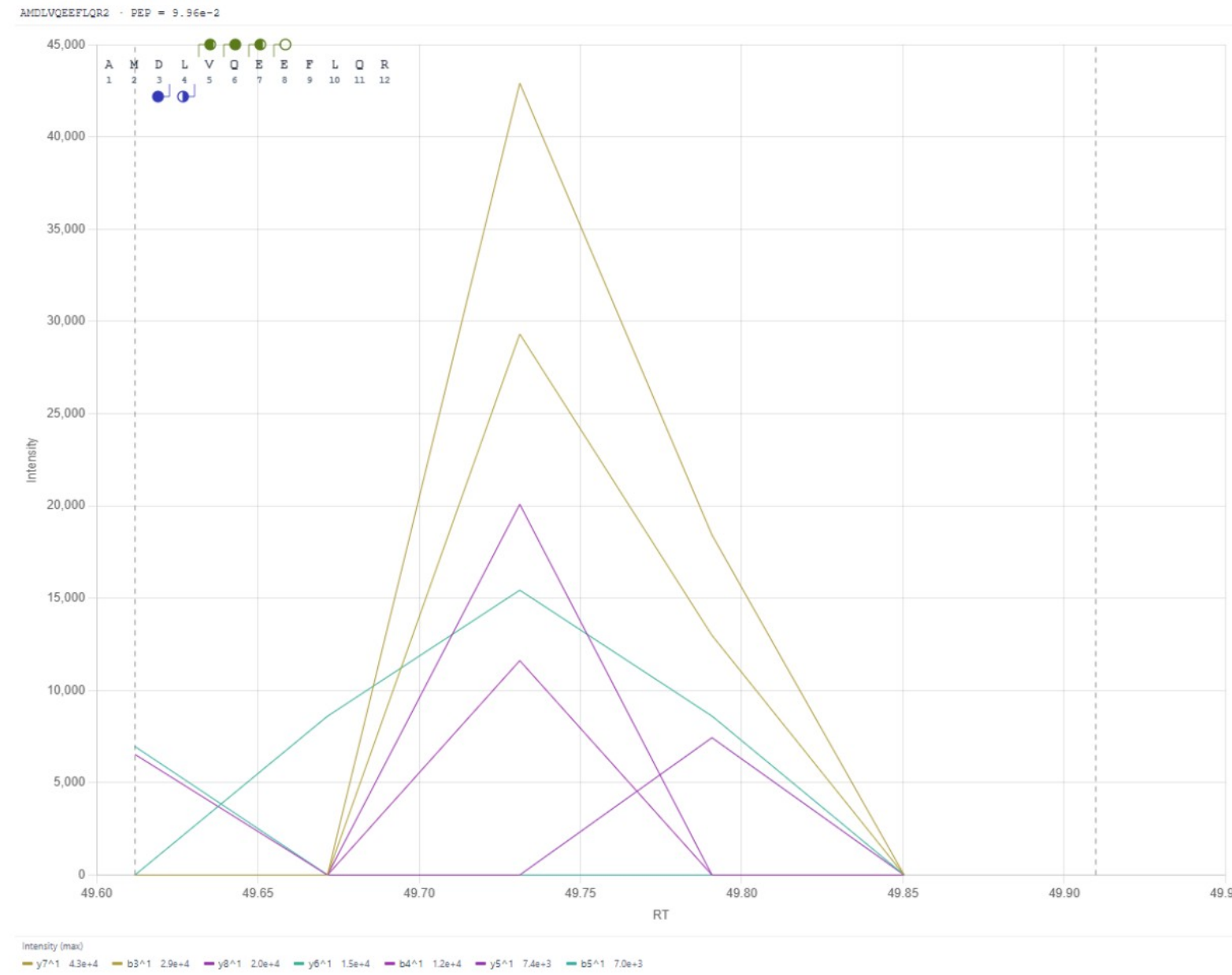

MYH9

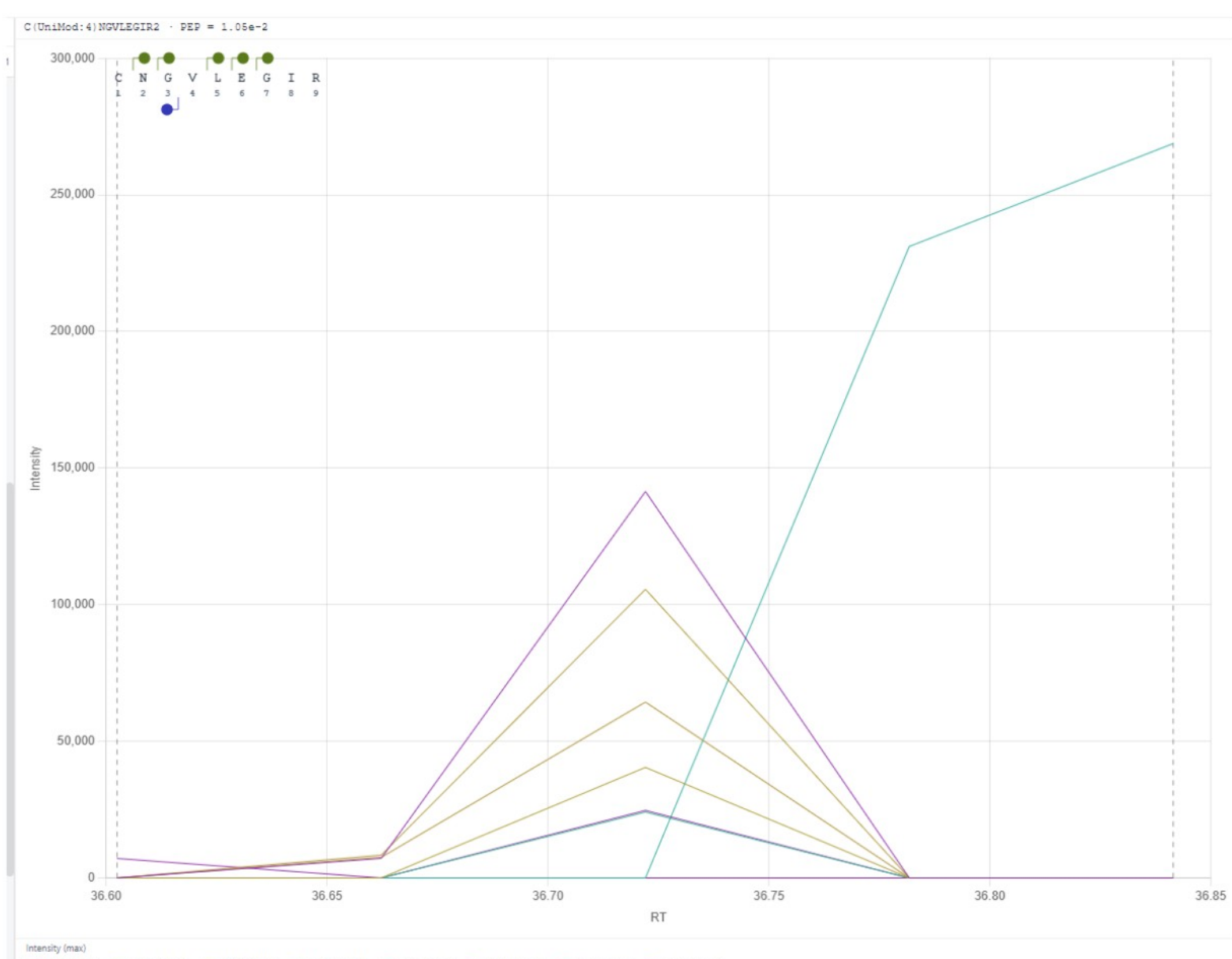

N4BP3

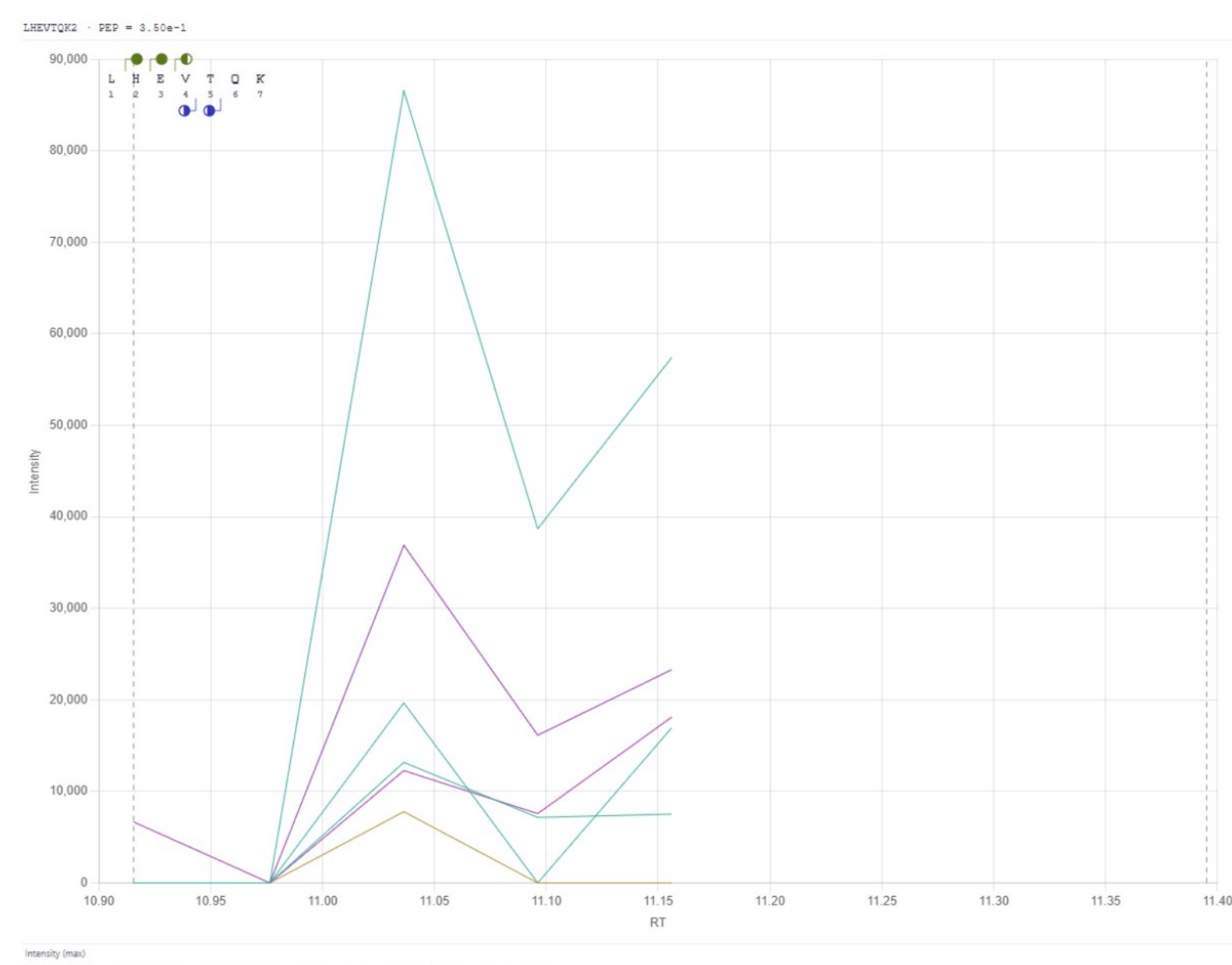

NBEAL2

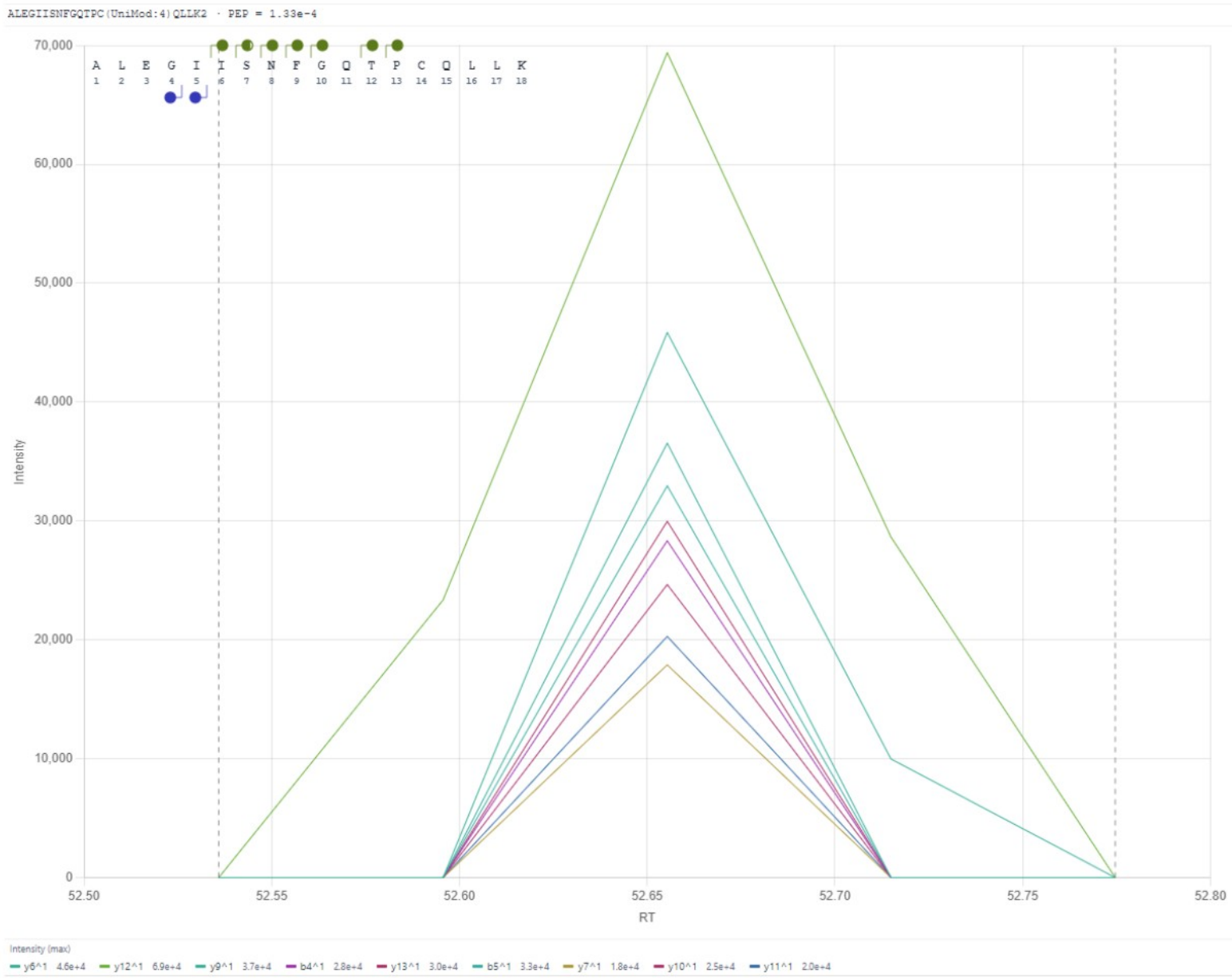

NCK2

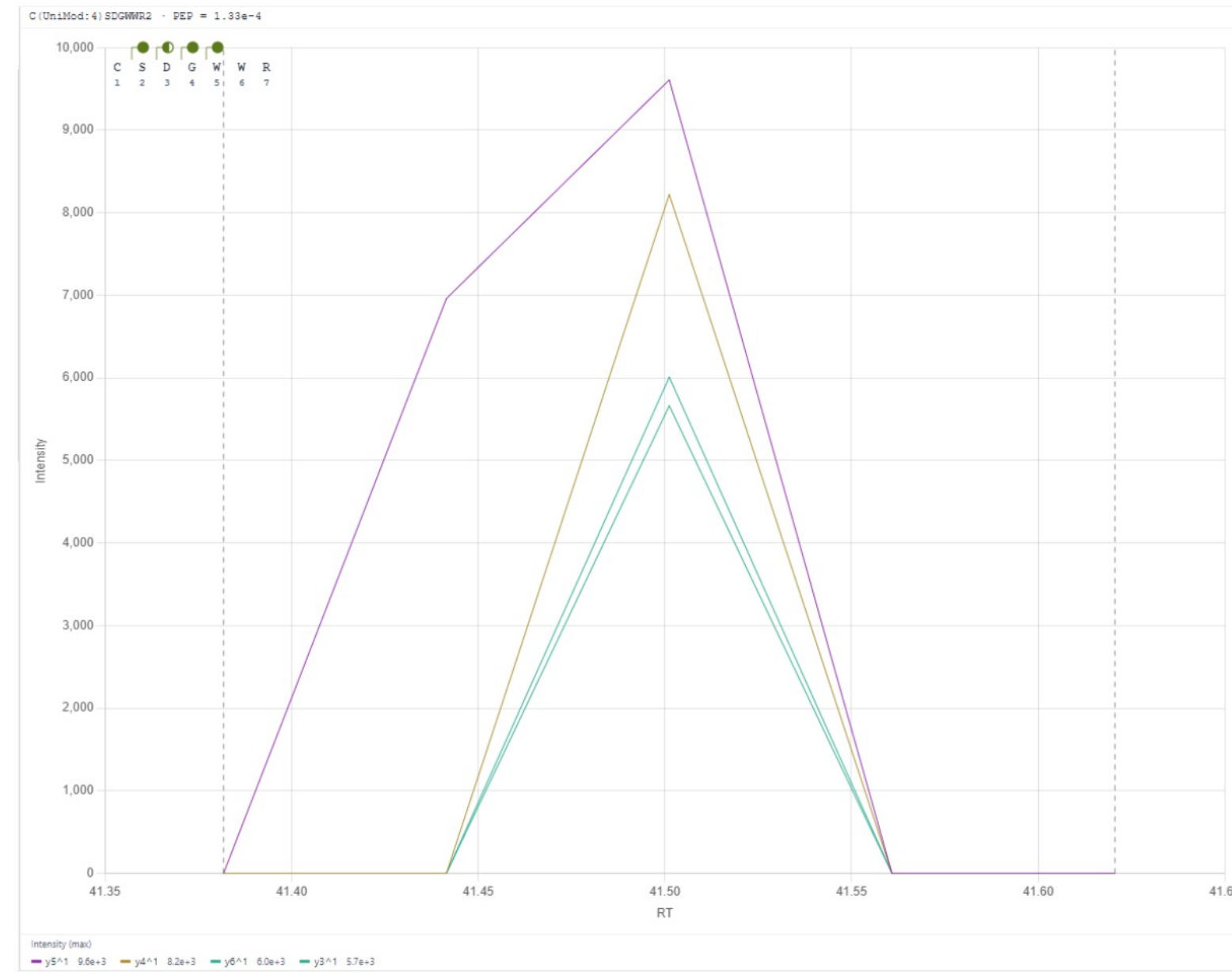

NCKAP1

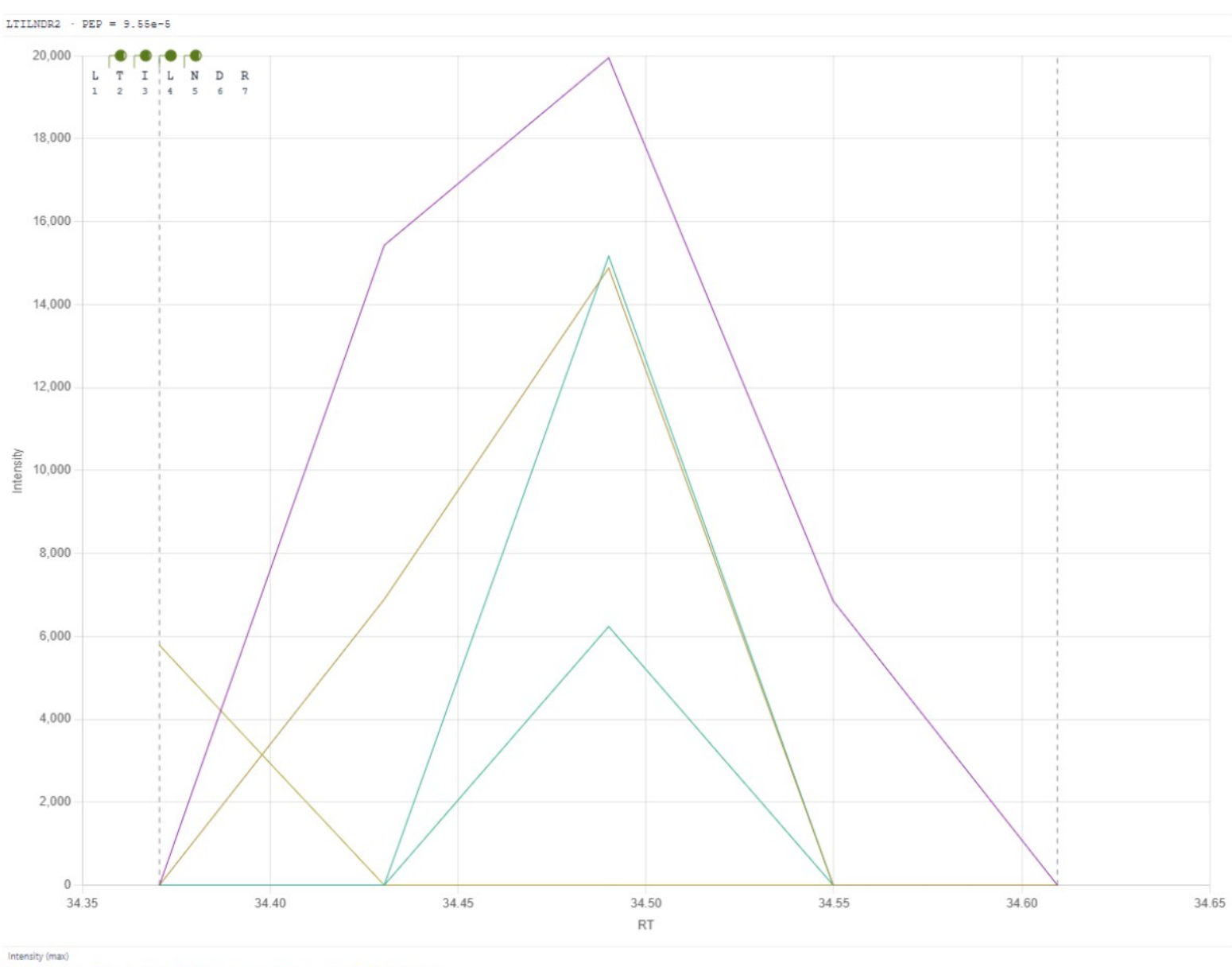

NDUFA6

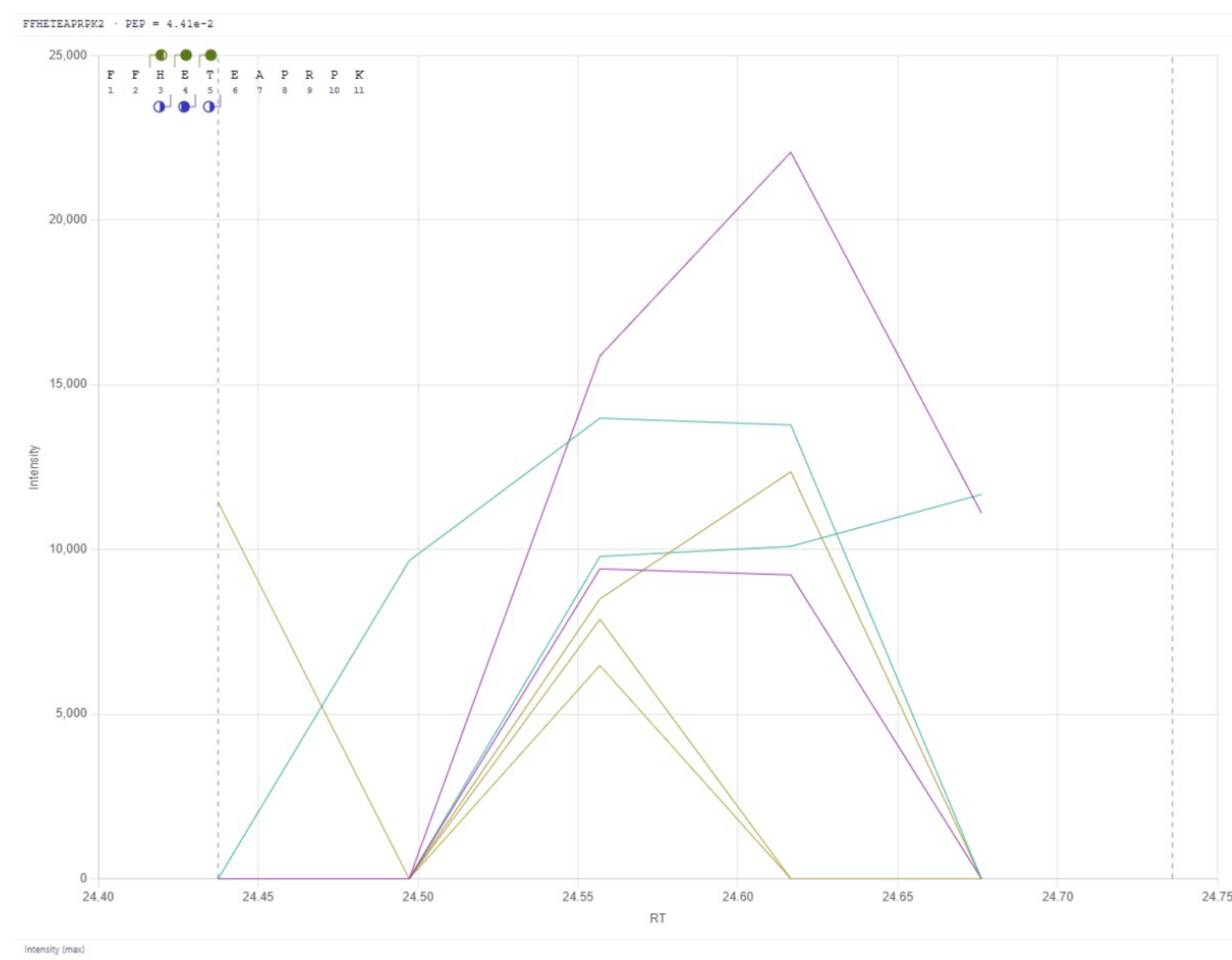

NDUFB11

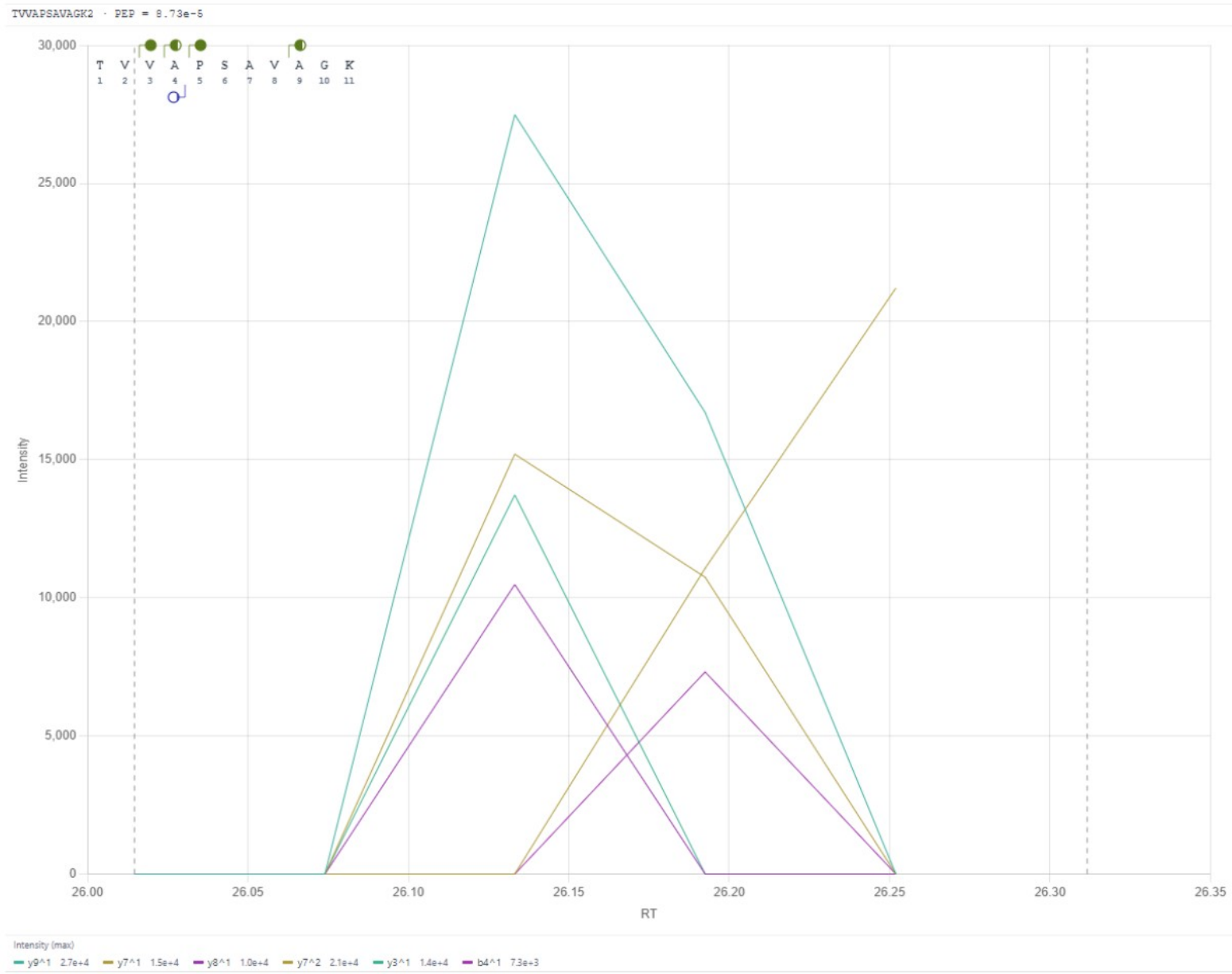

NDUFS5

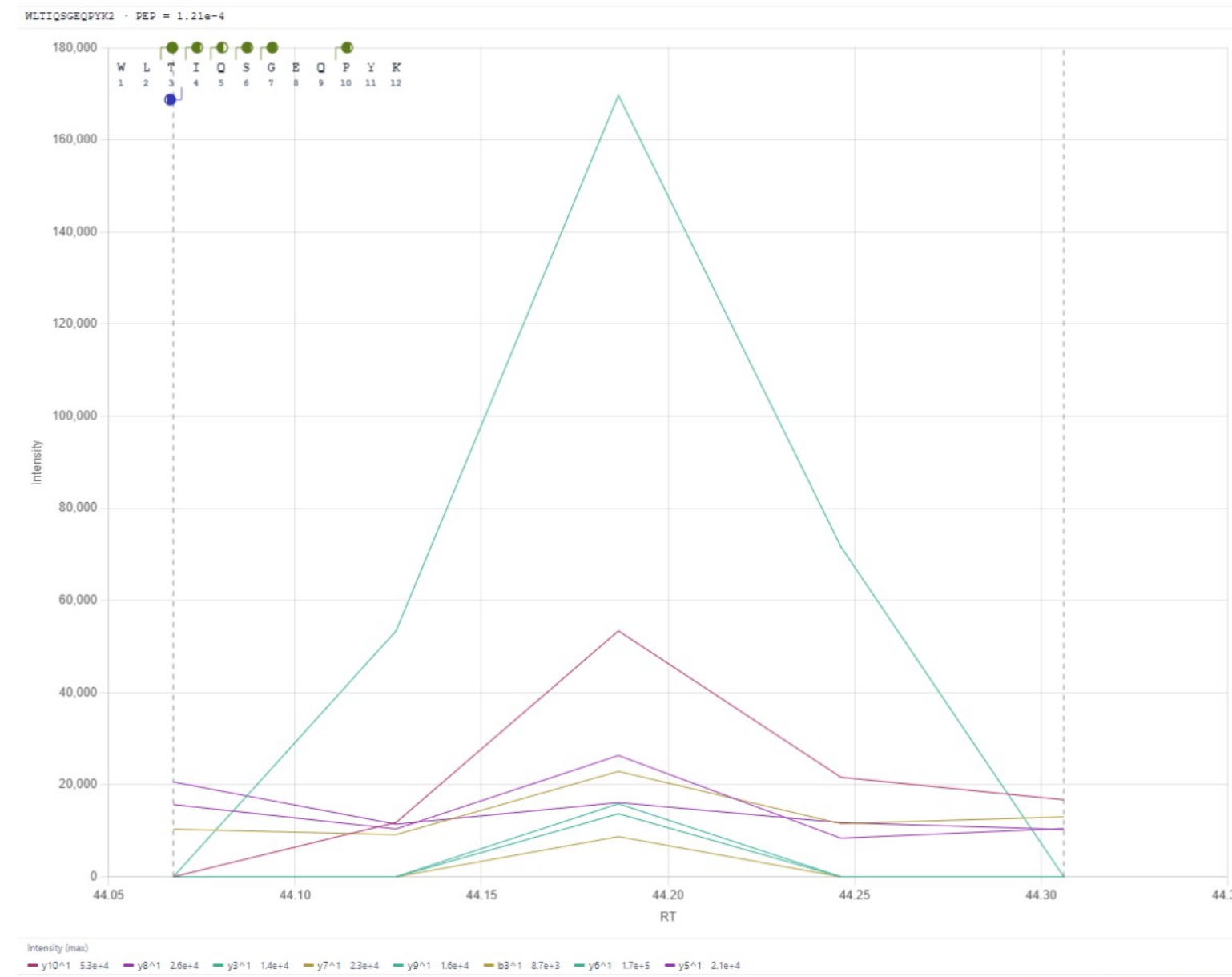

NECTIN3

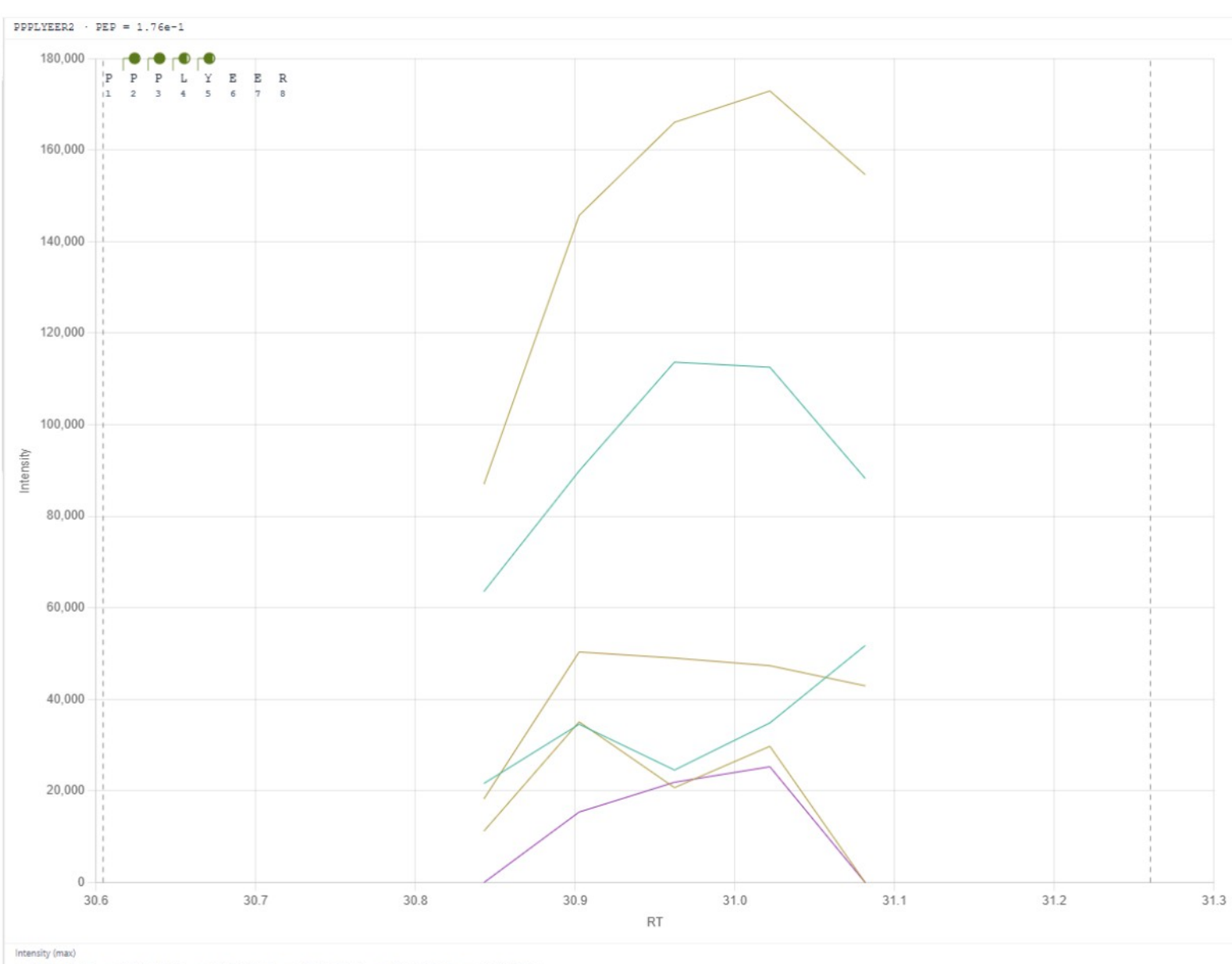

NID1

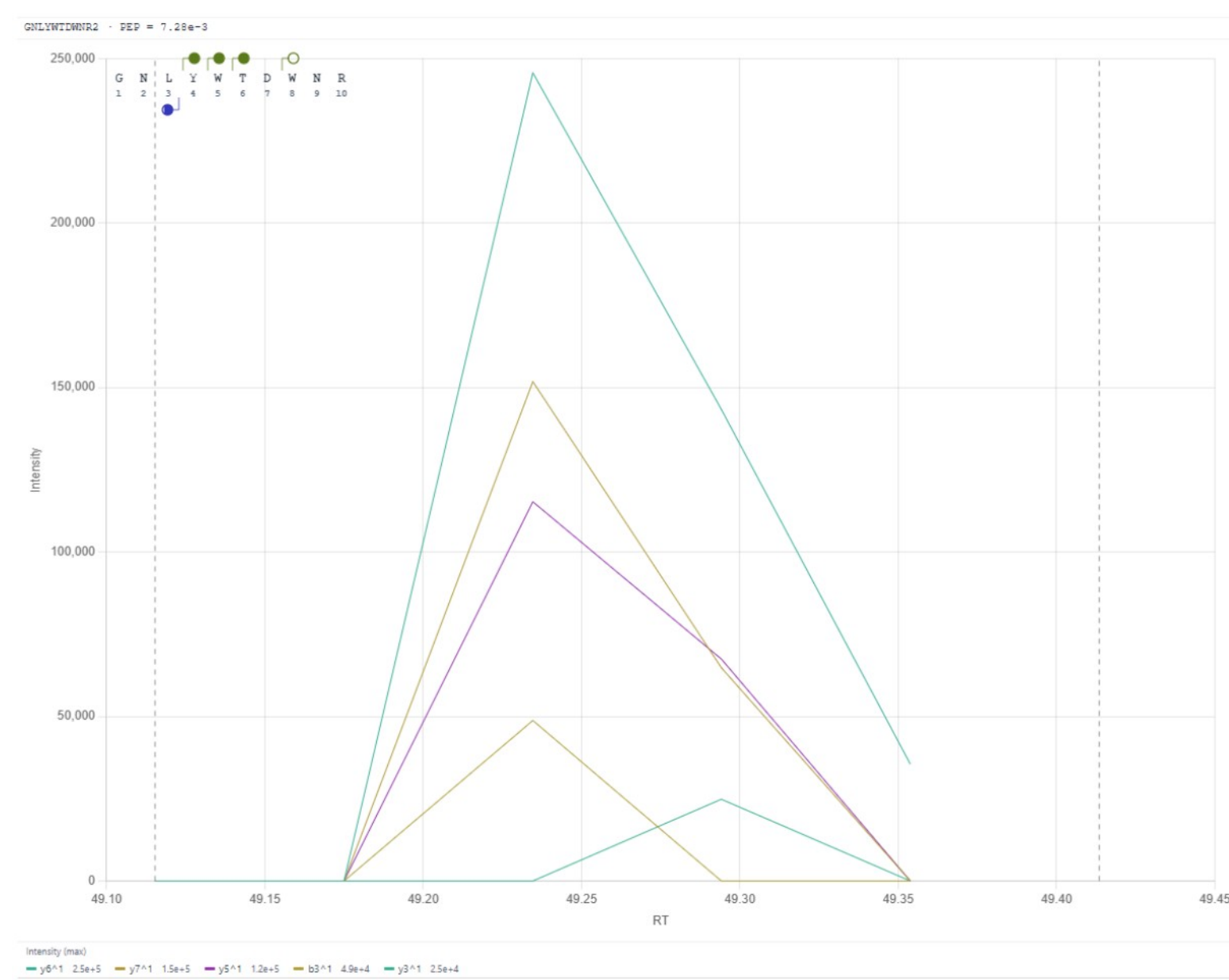

NME2P1

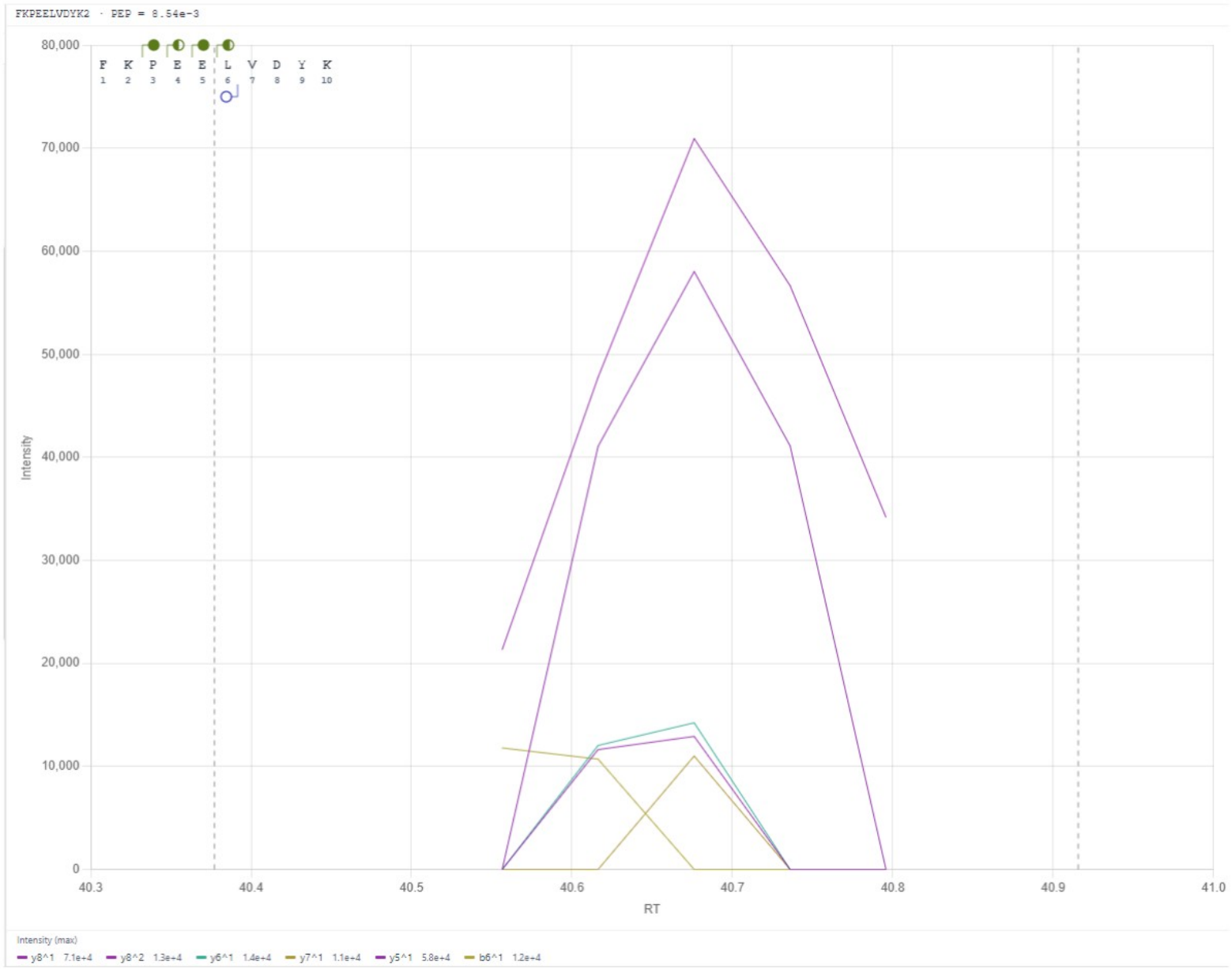

NME4

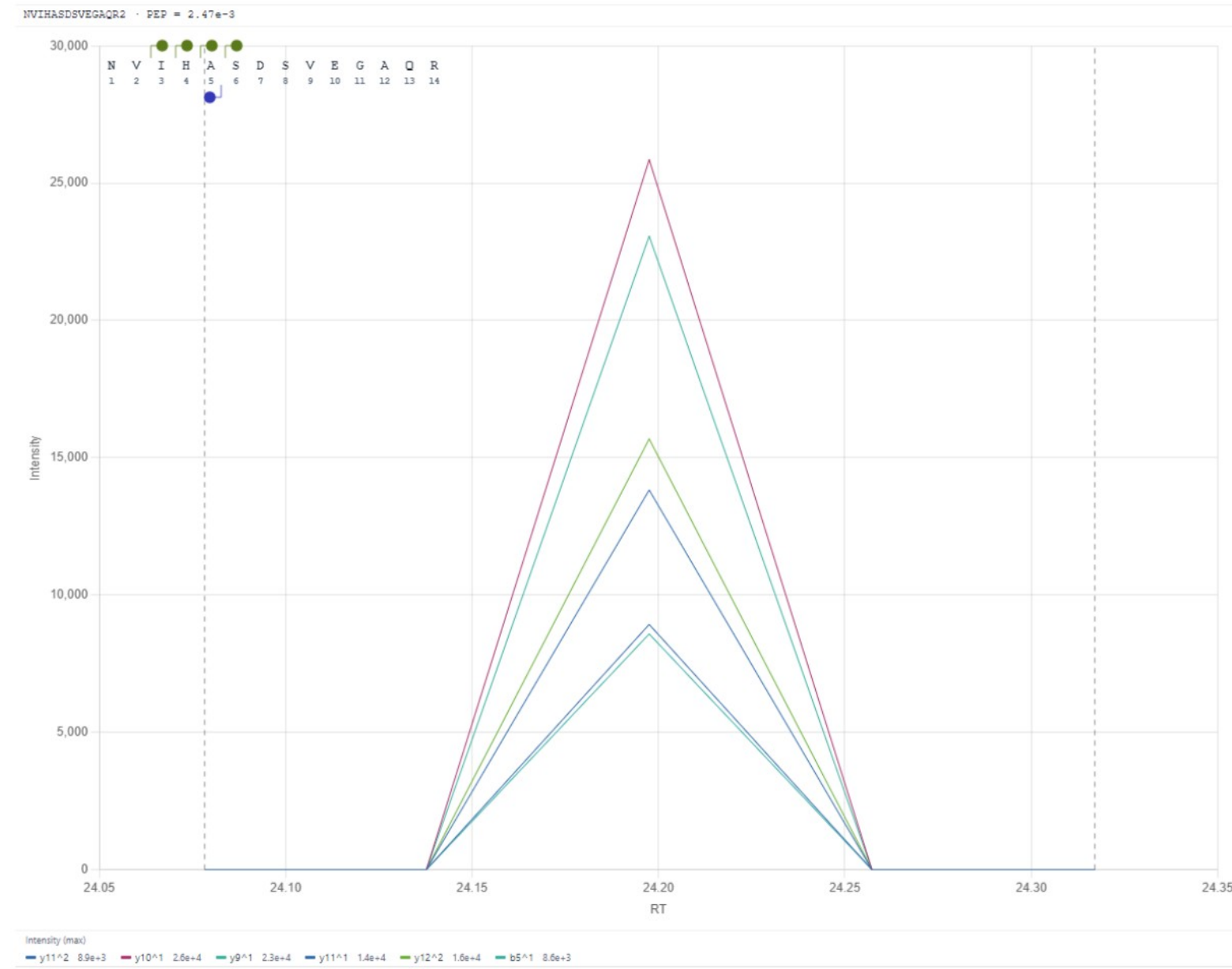

NOM1

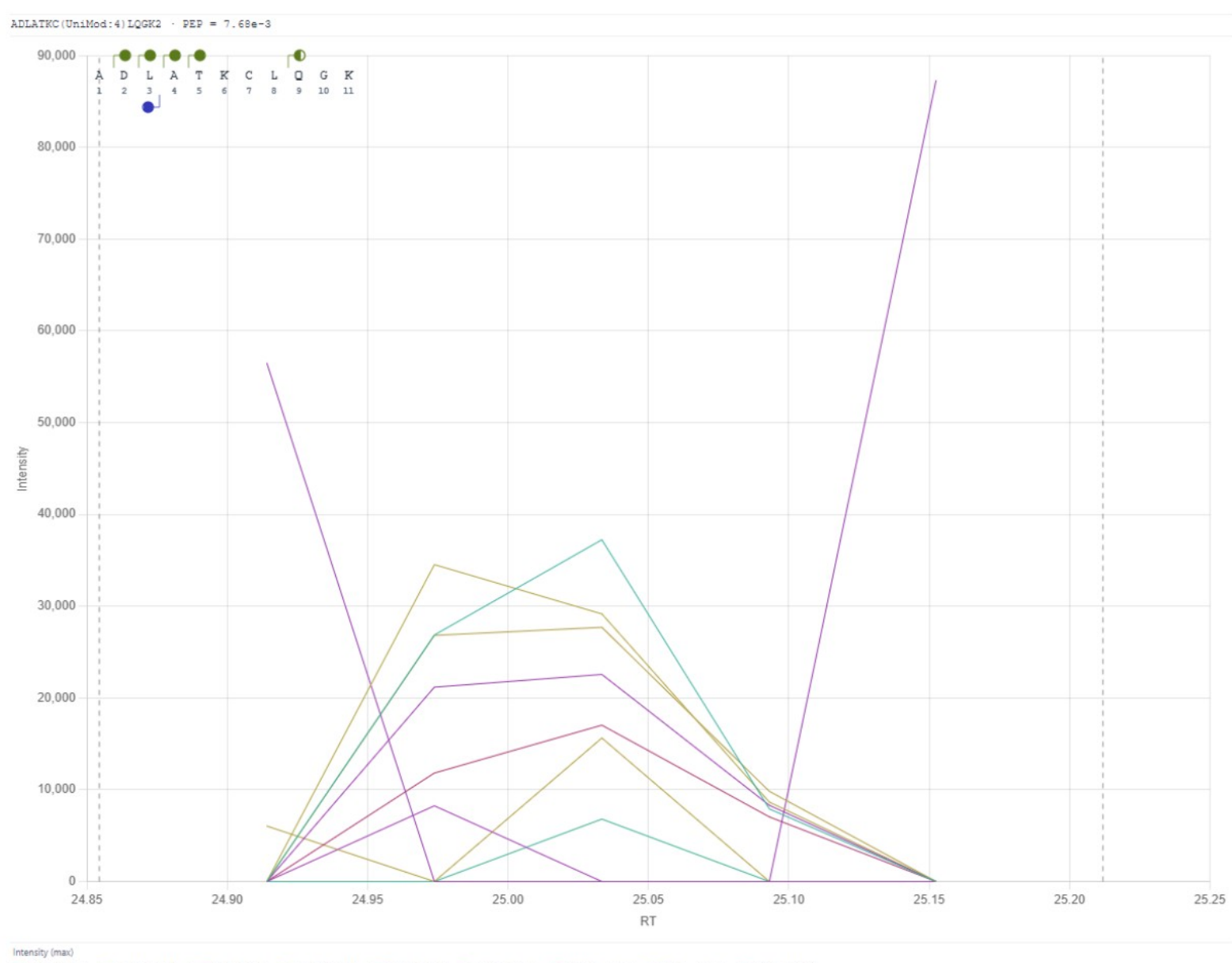

NTPCR

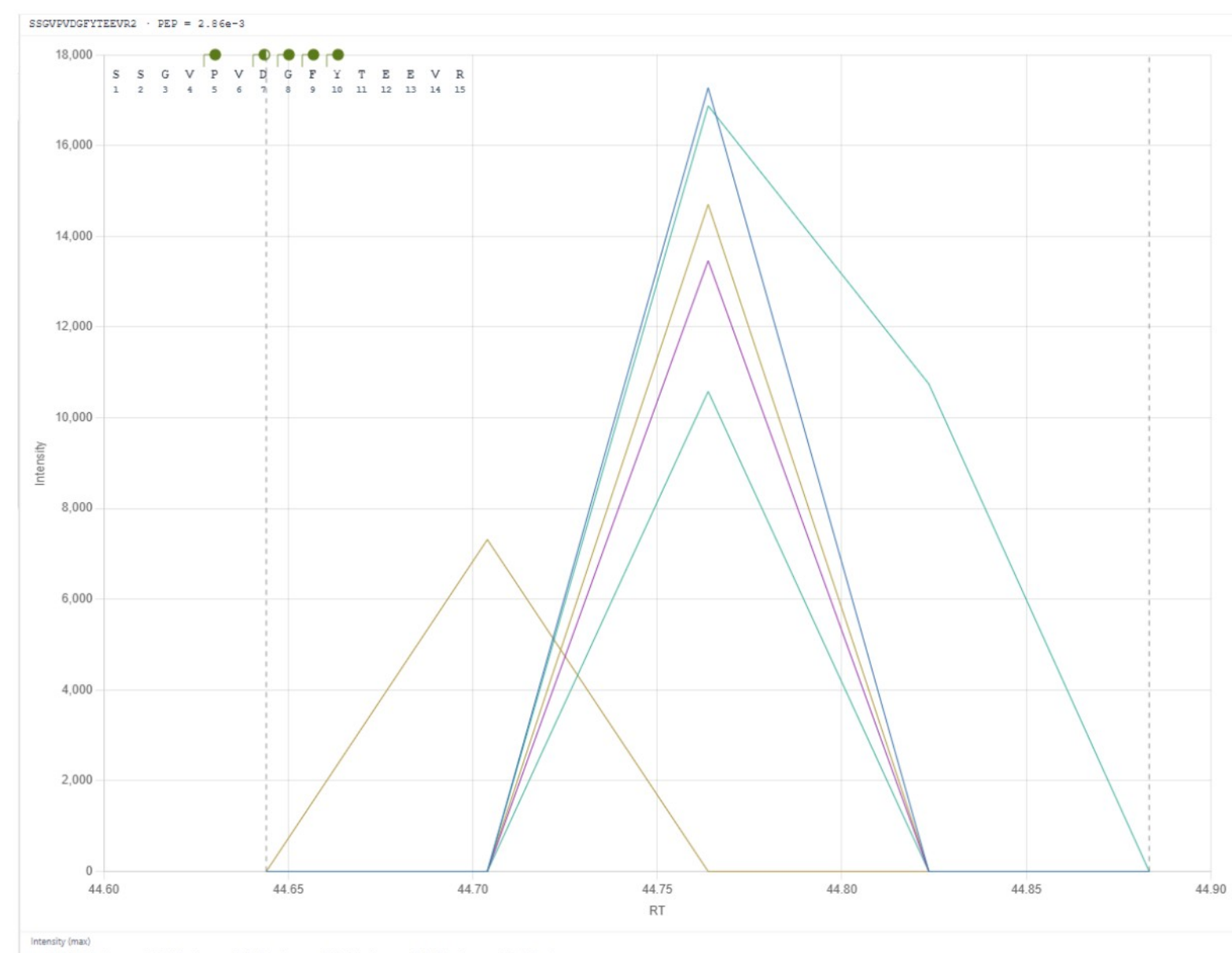

OSBPL7

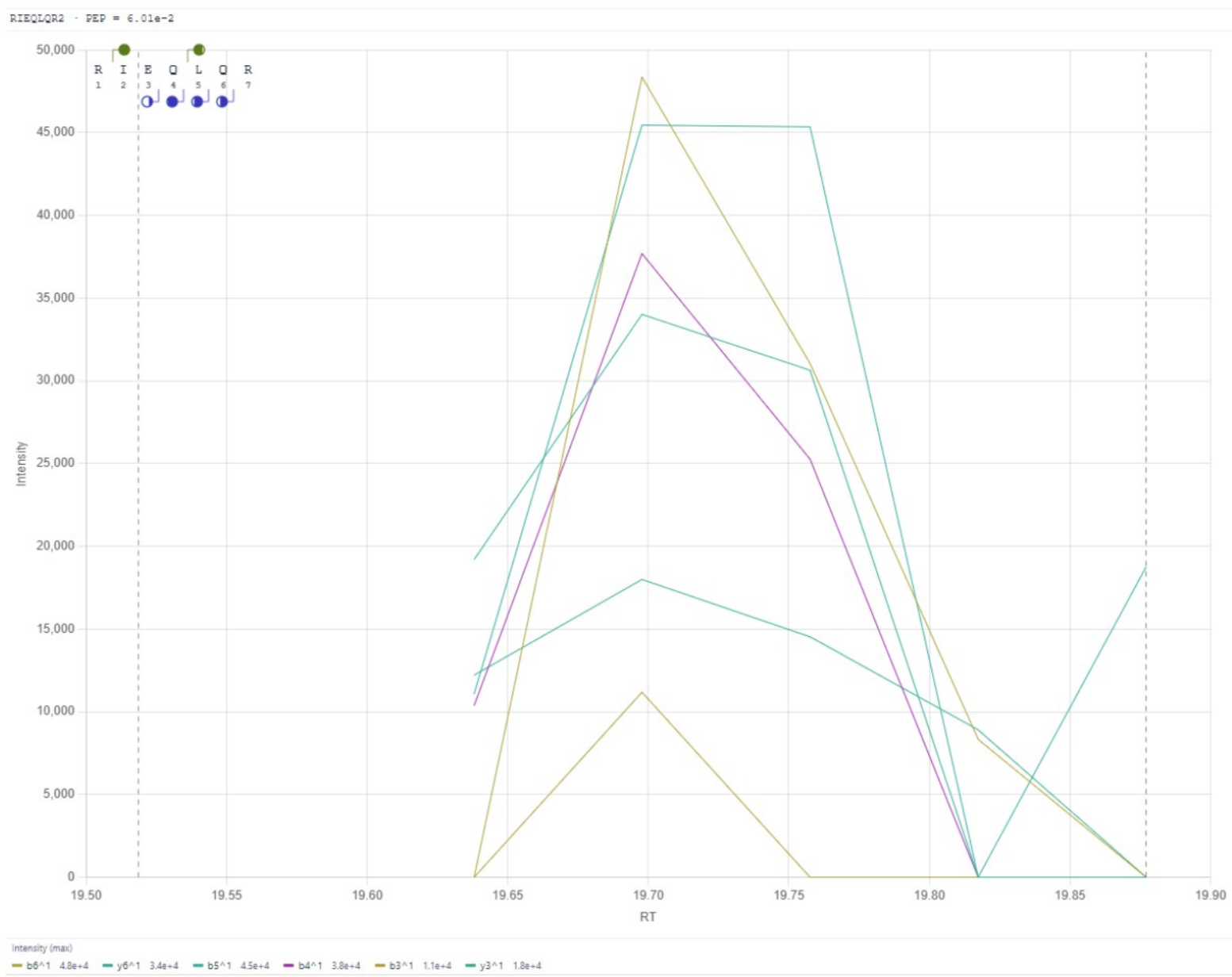

OSTC

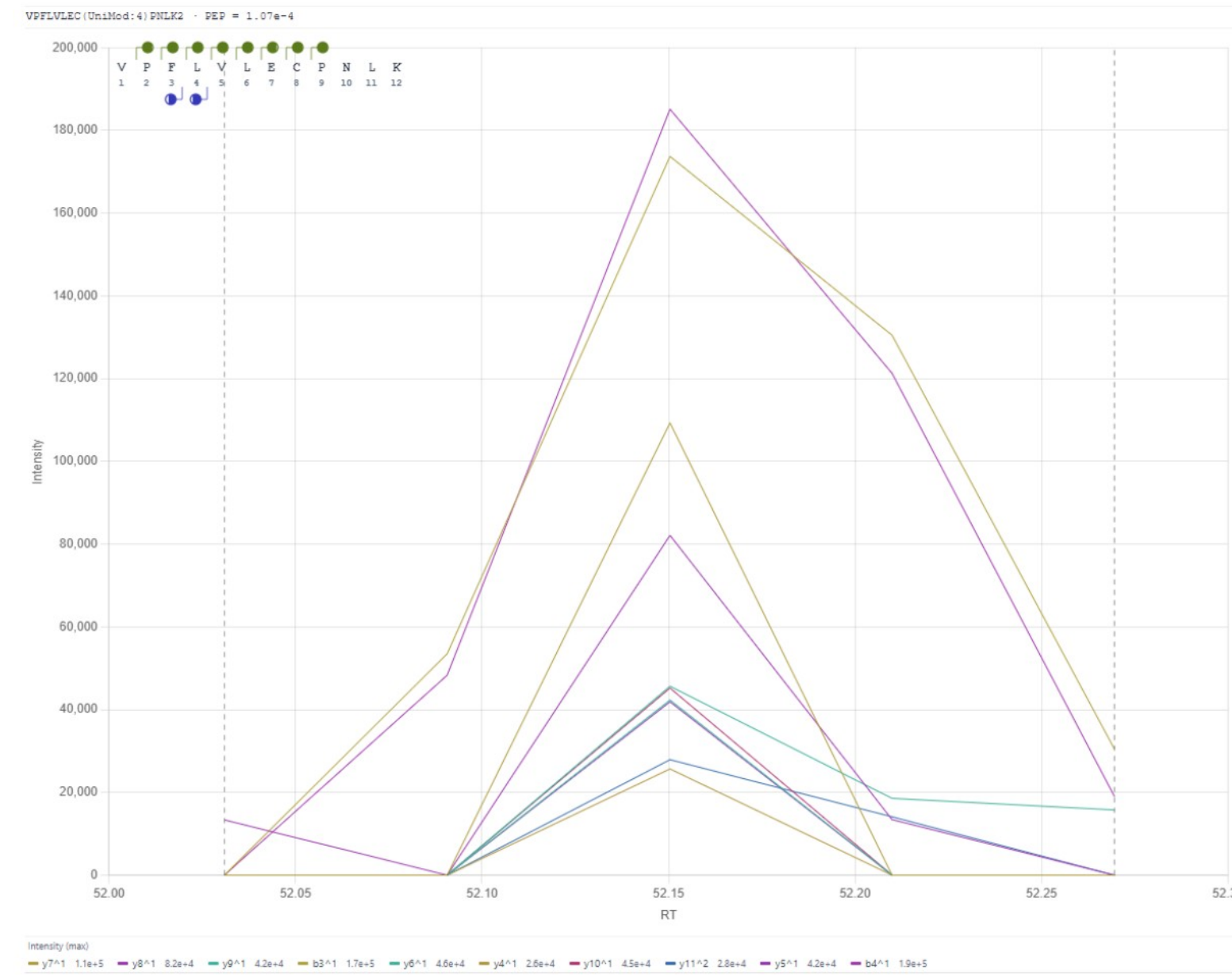

PACSIN2

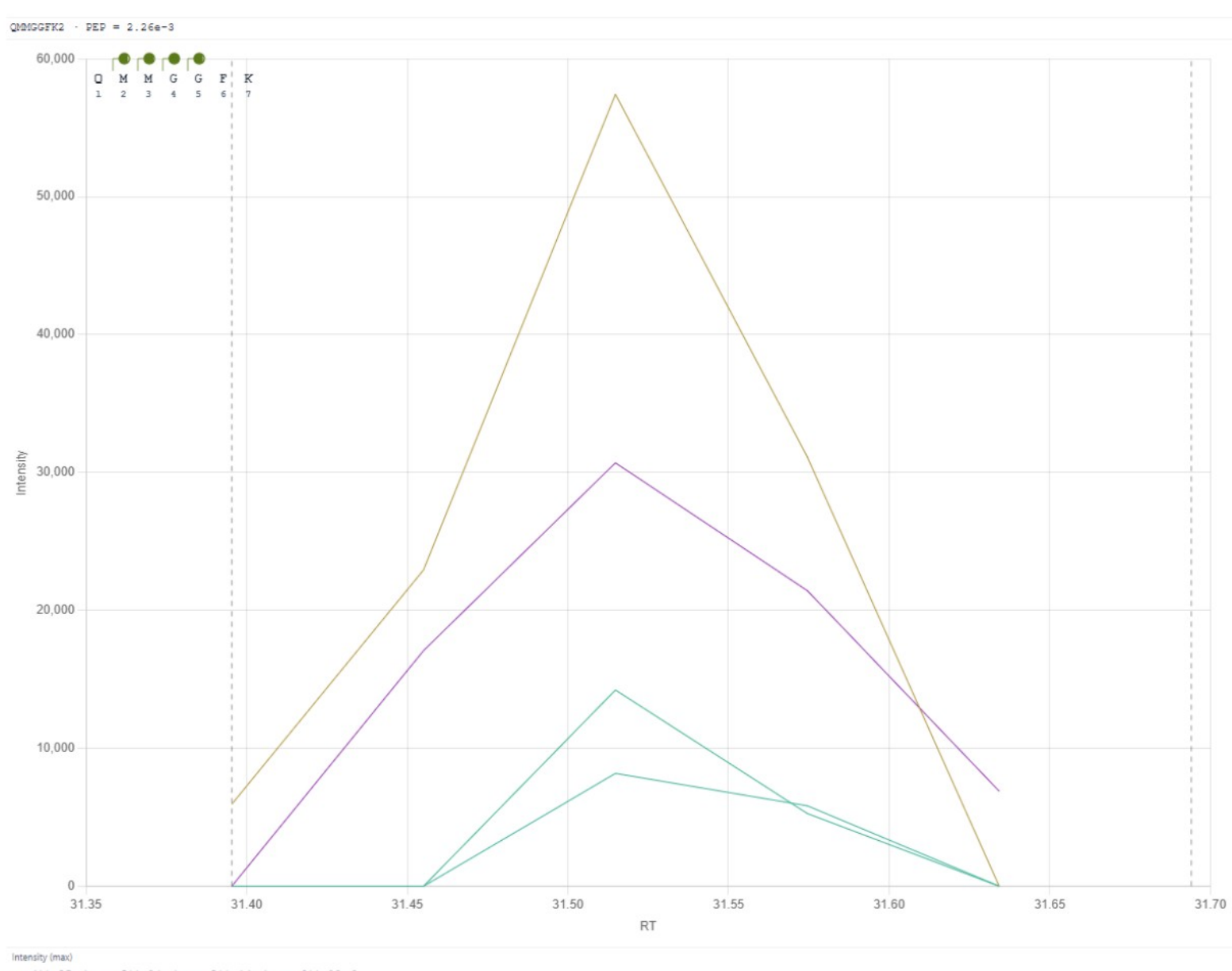

PAFAH1B3

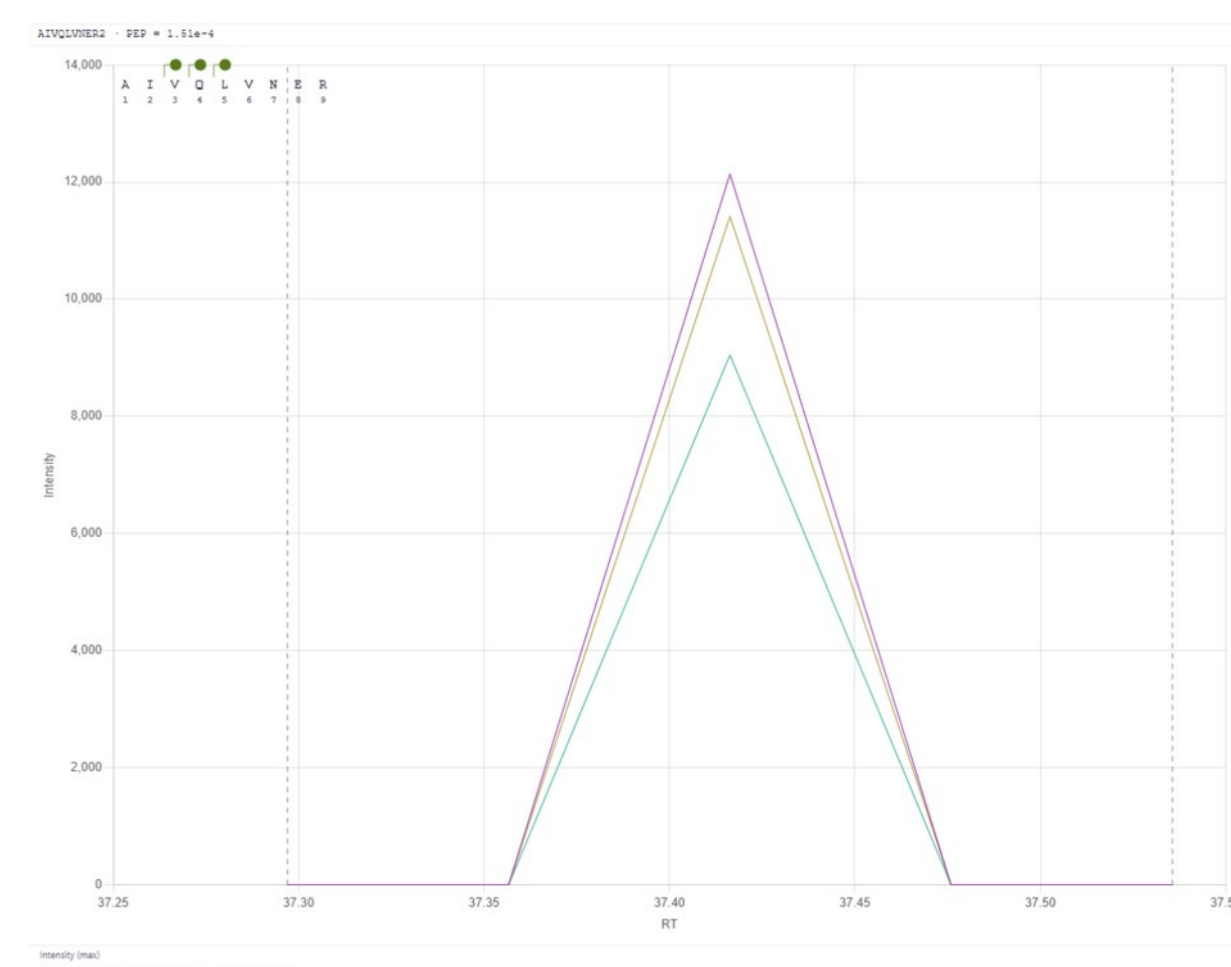

PDK1

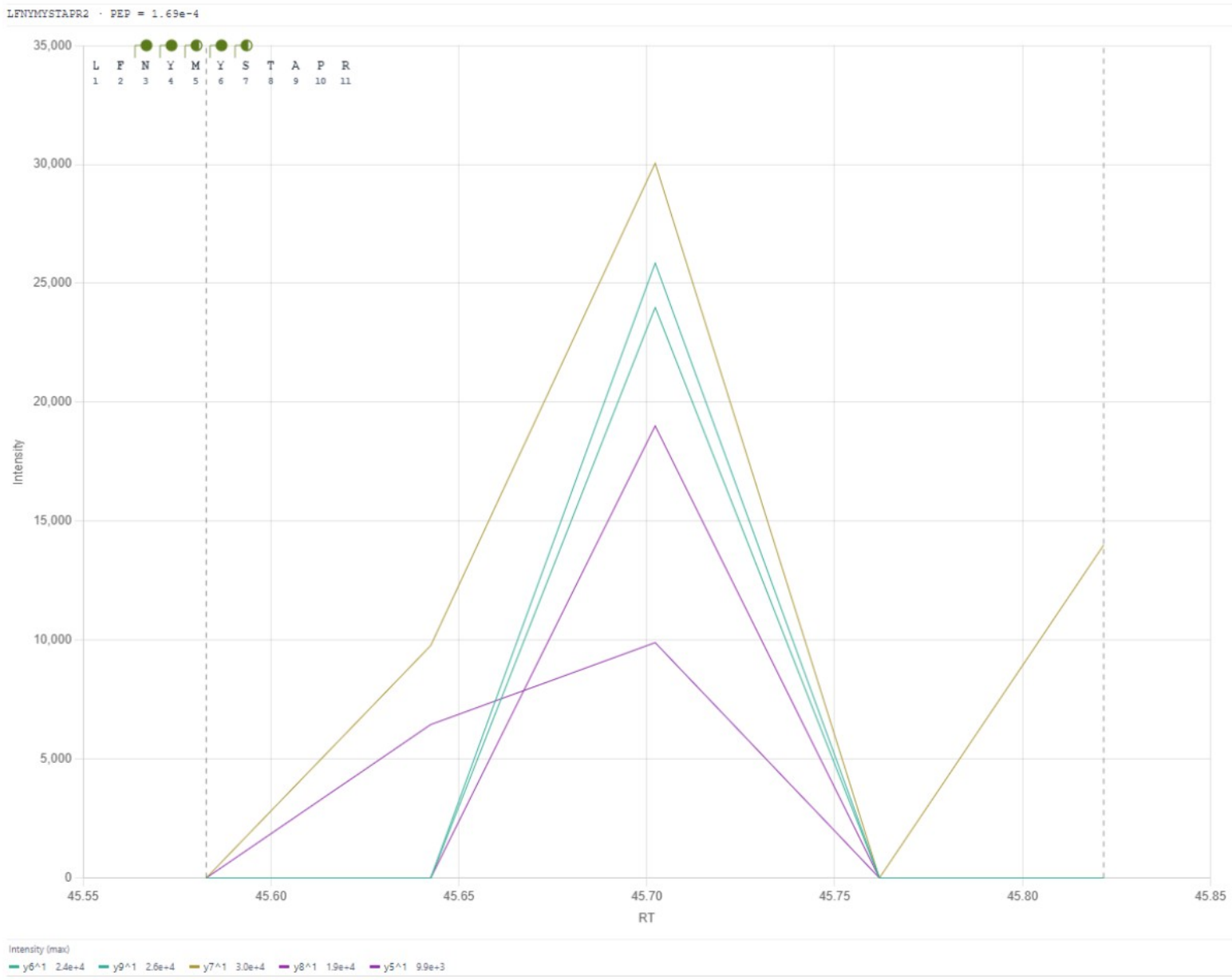

PFKL

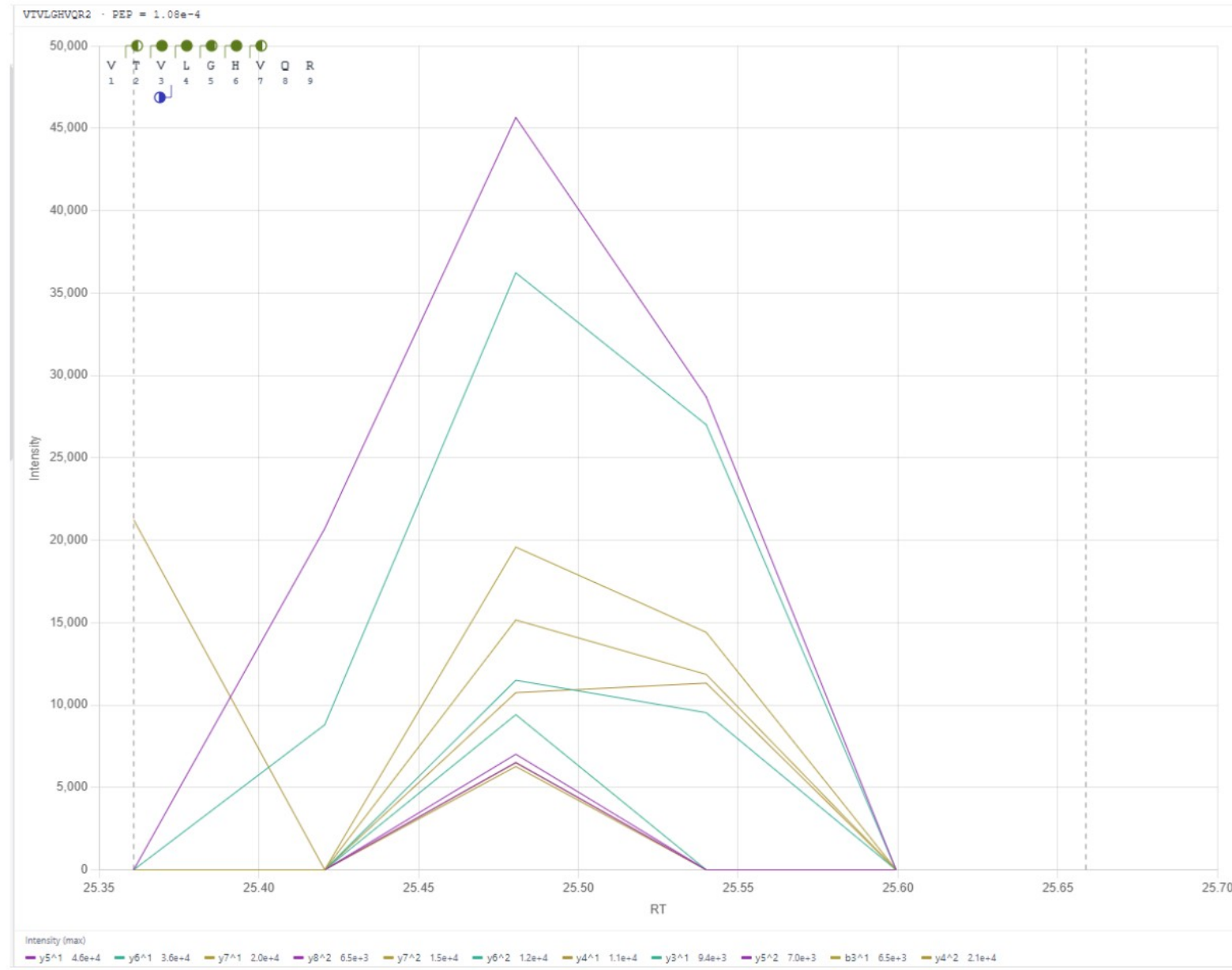

PFN3

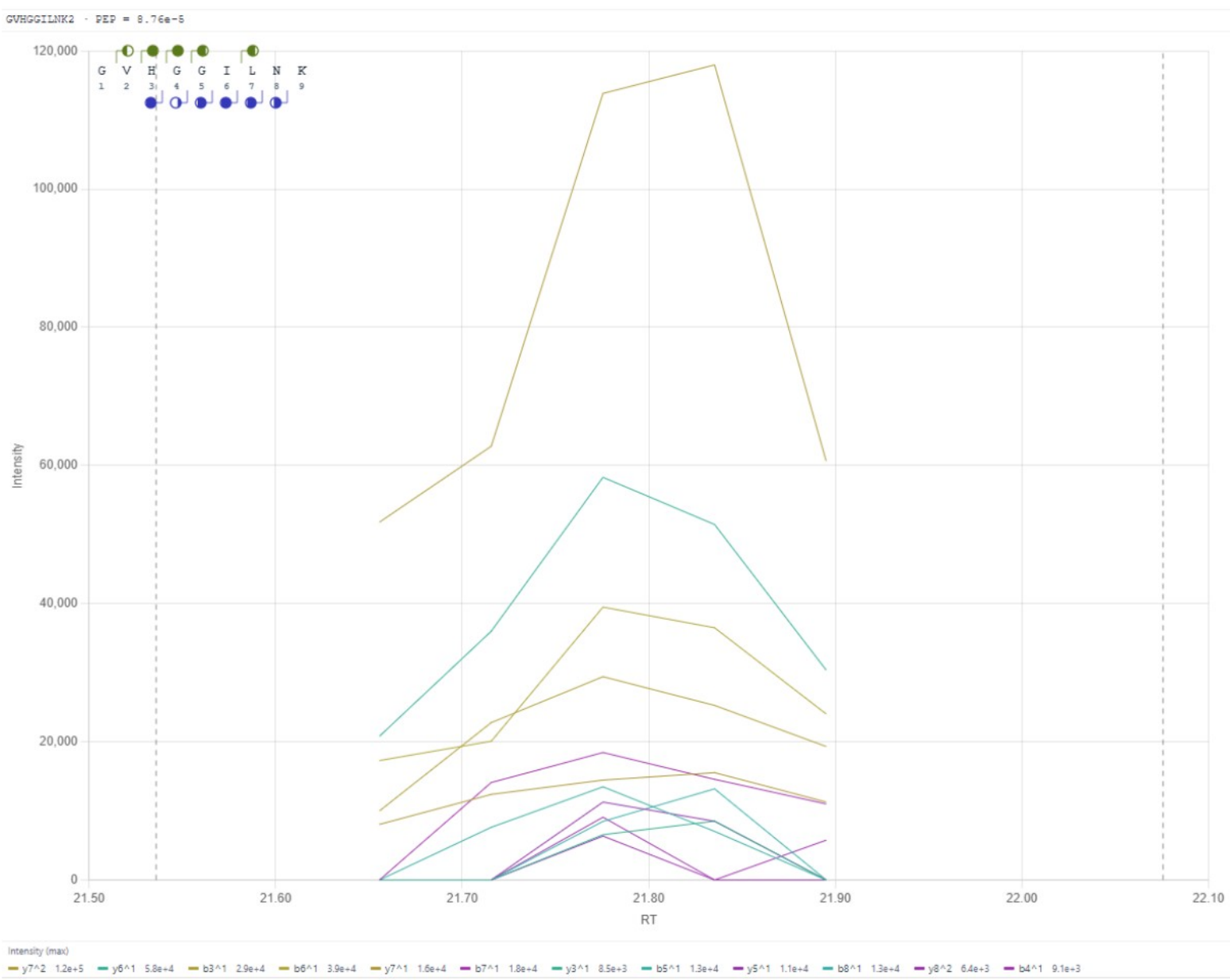

pgrmc1

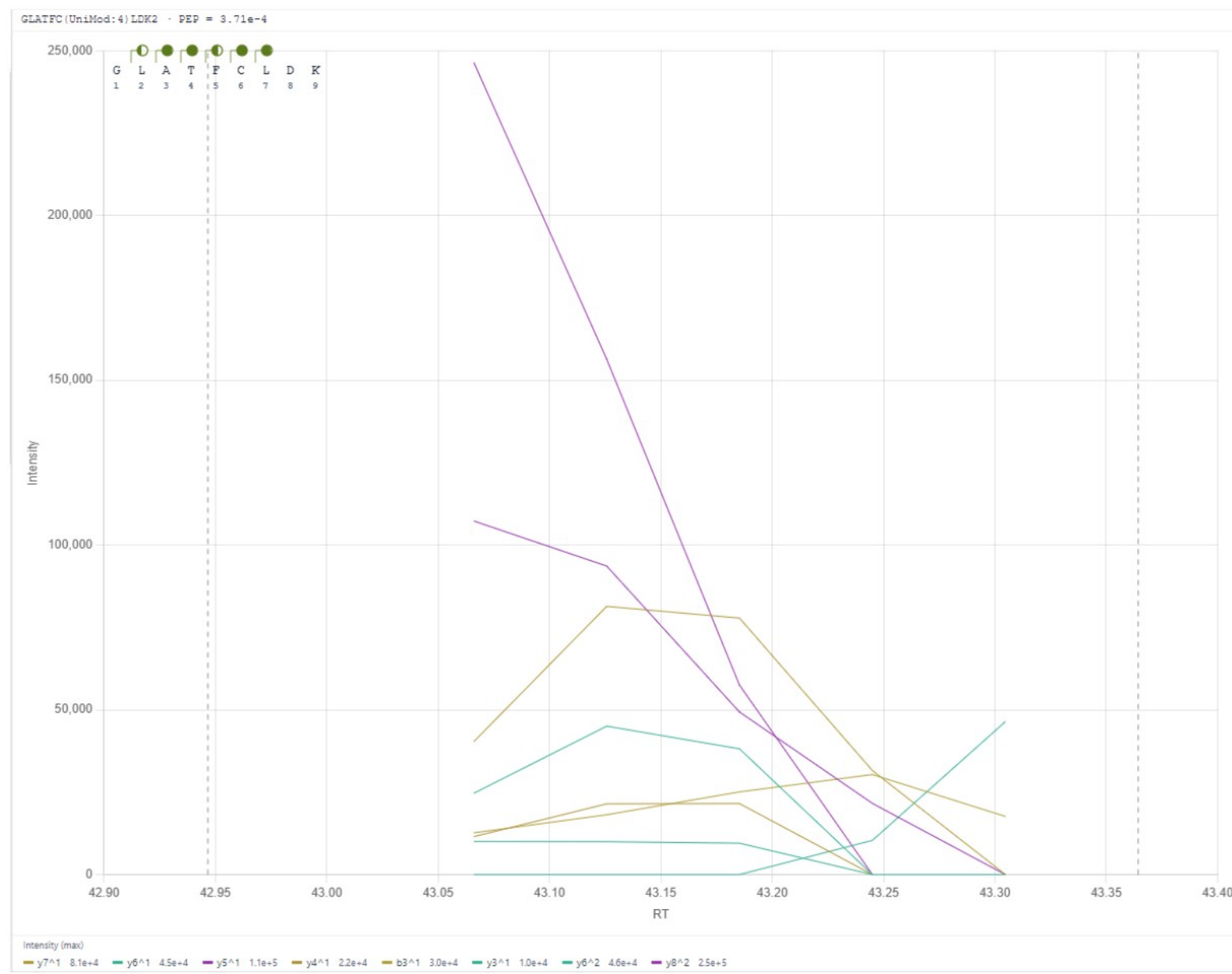

PIK3R1

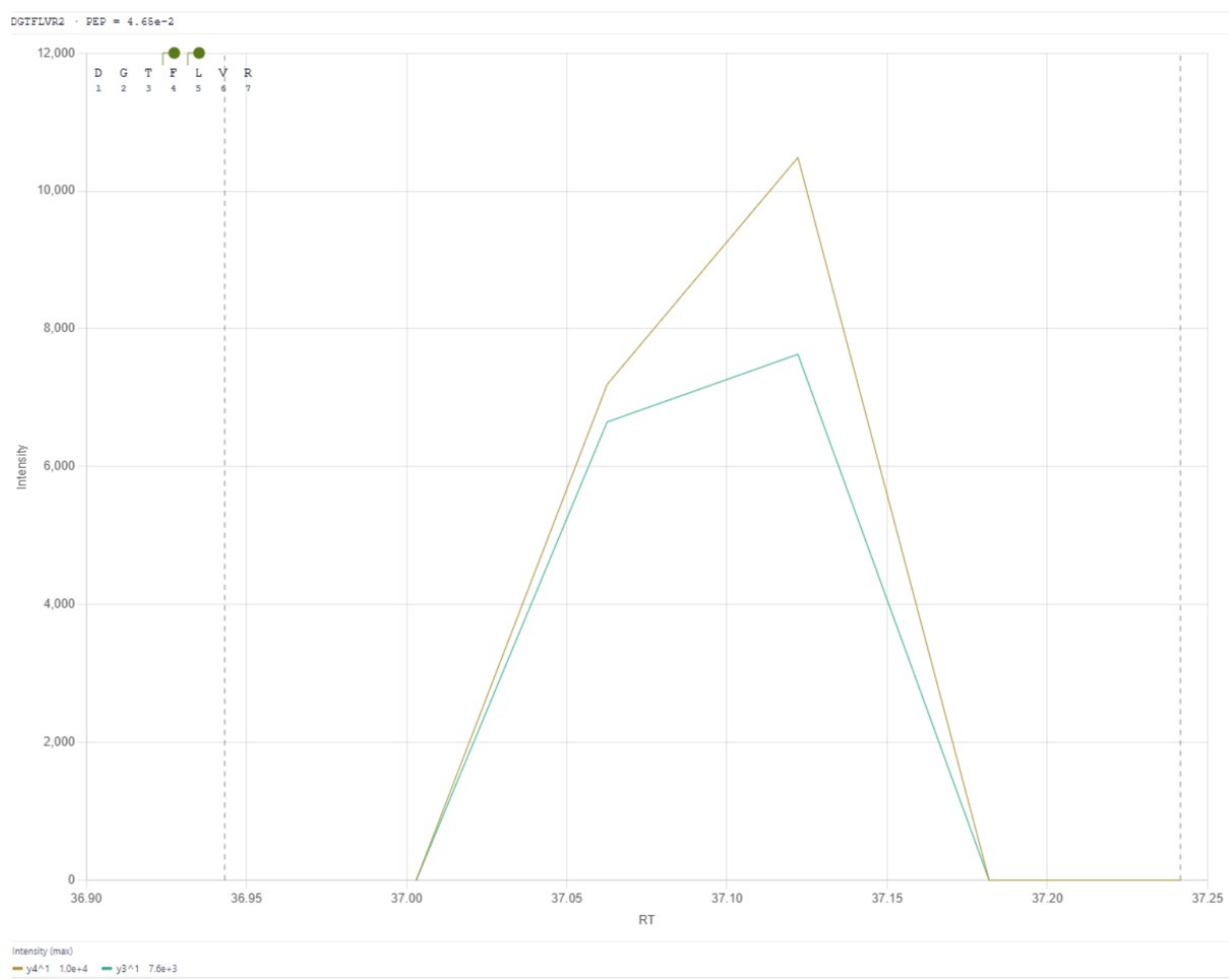

PITPNB

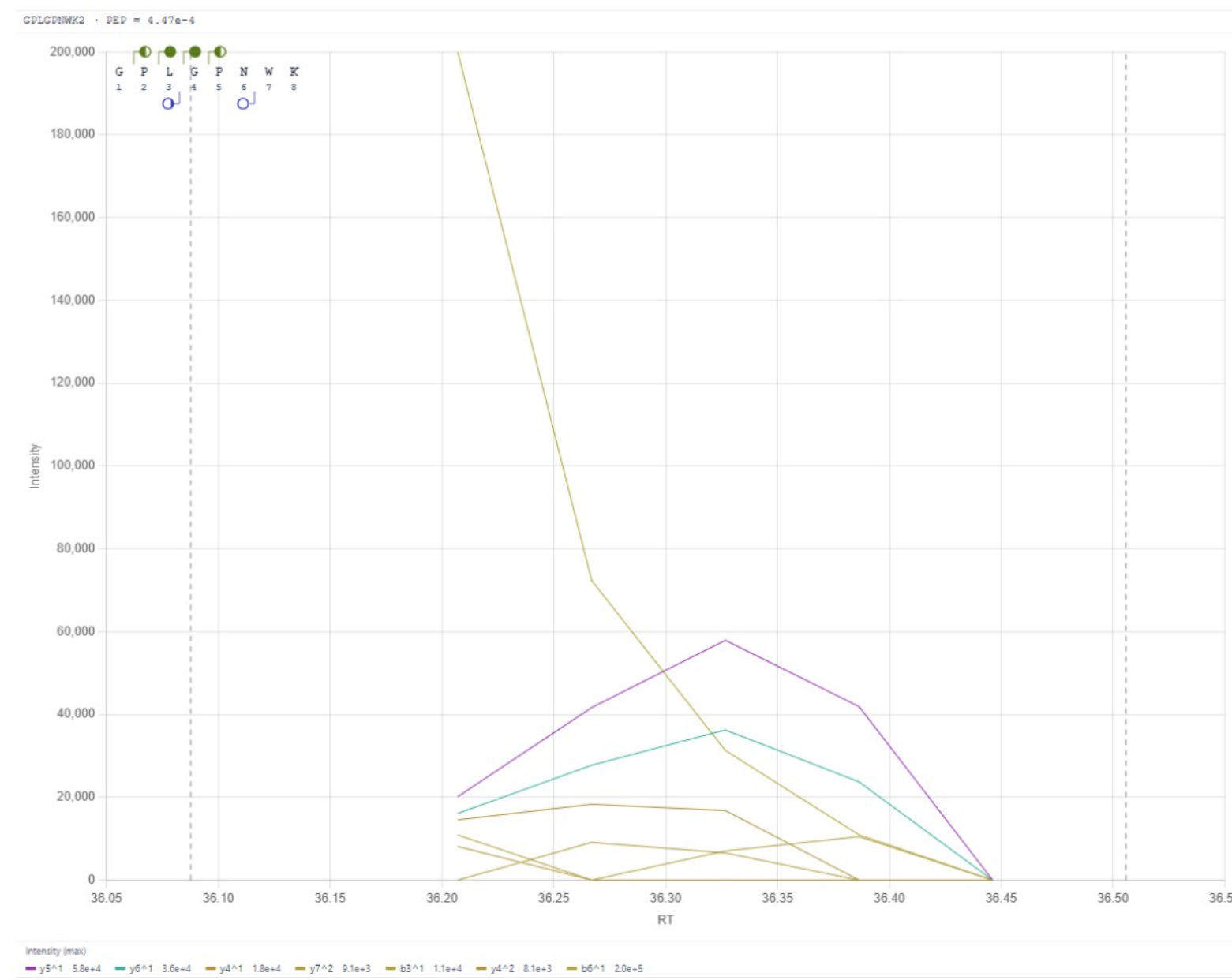

PKM

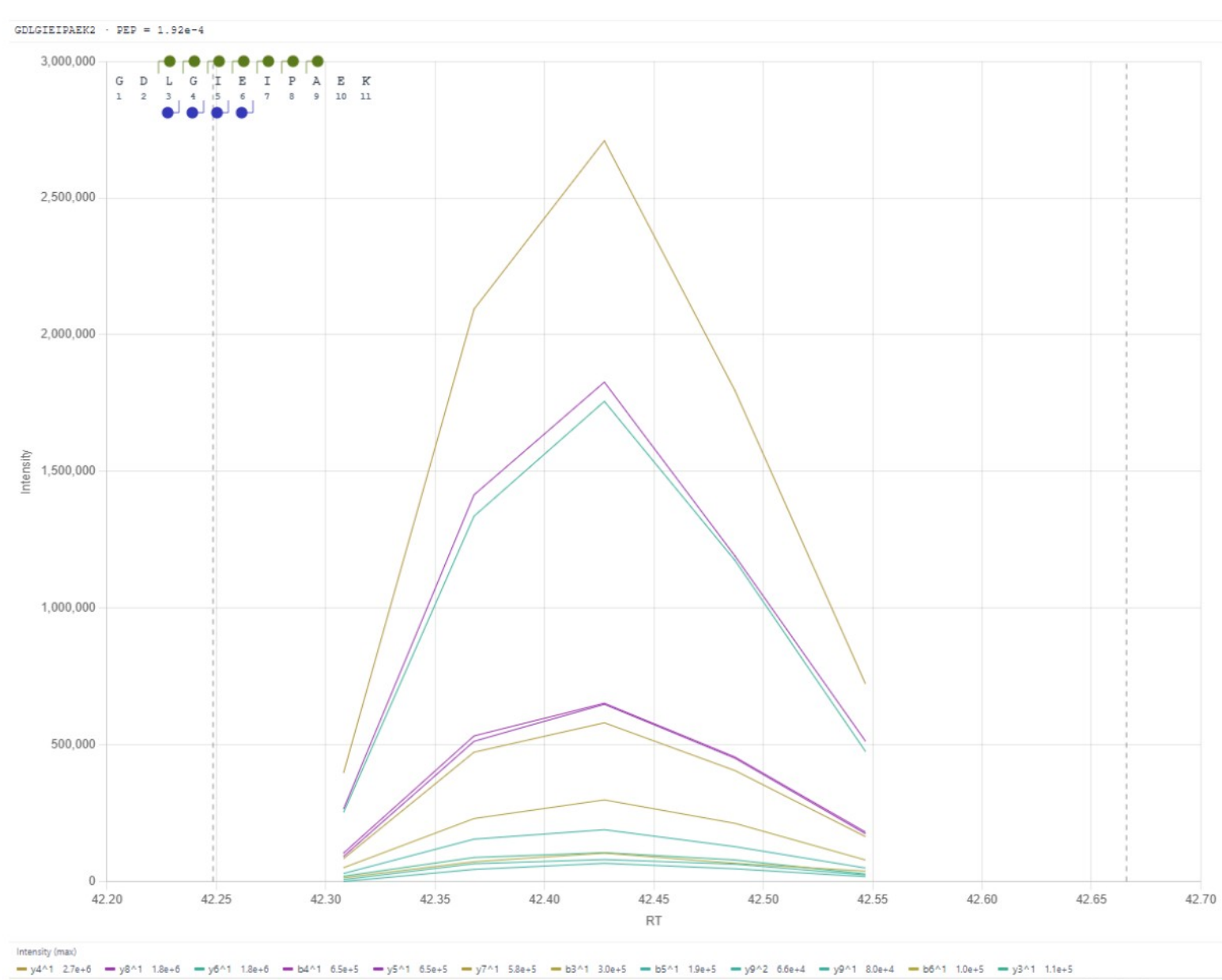

PLS3

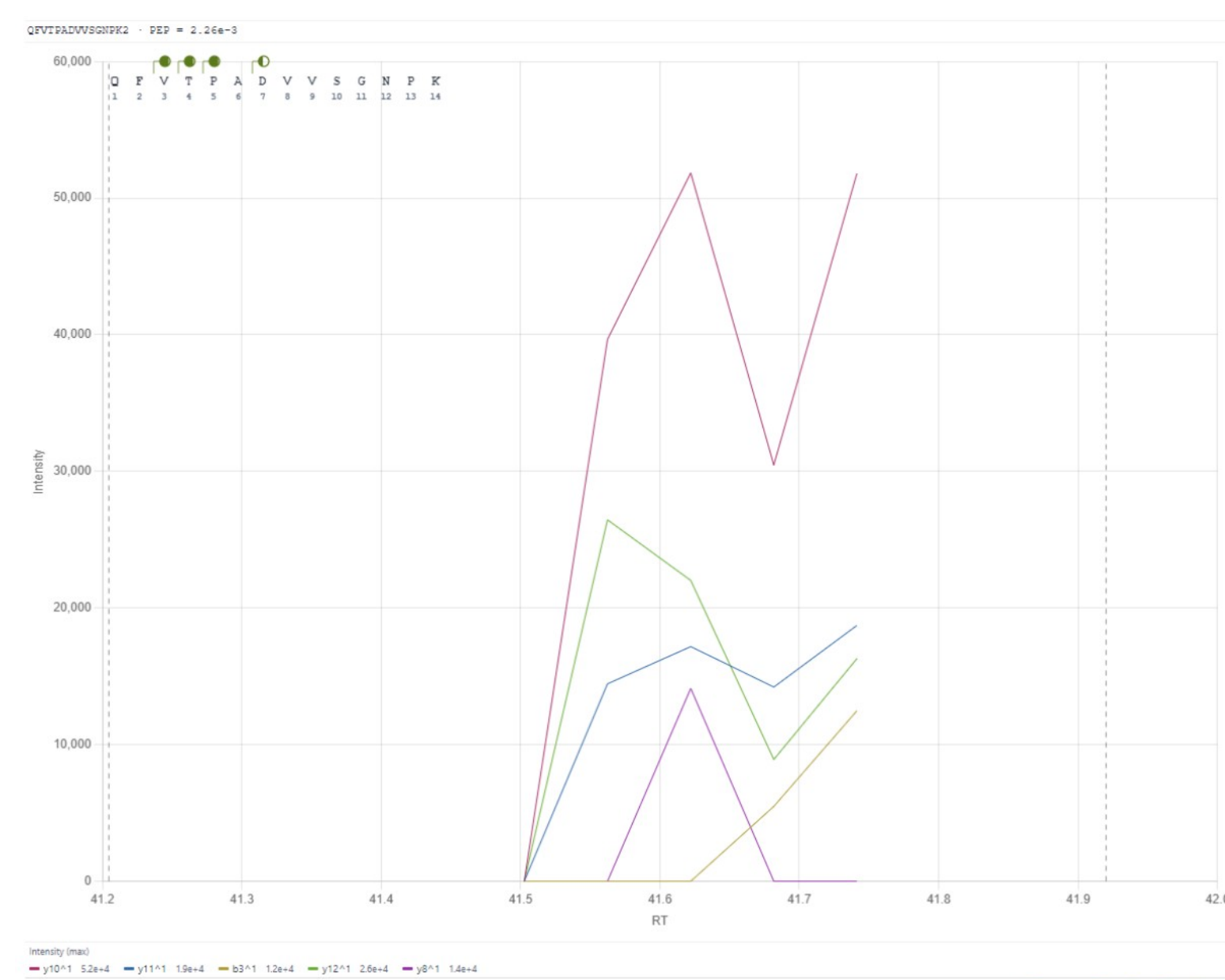

PLSCR1

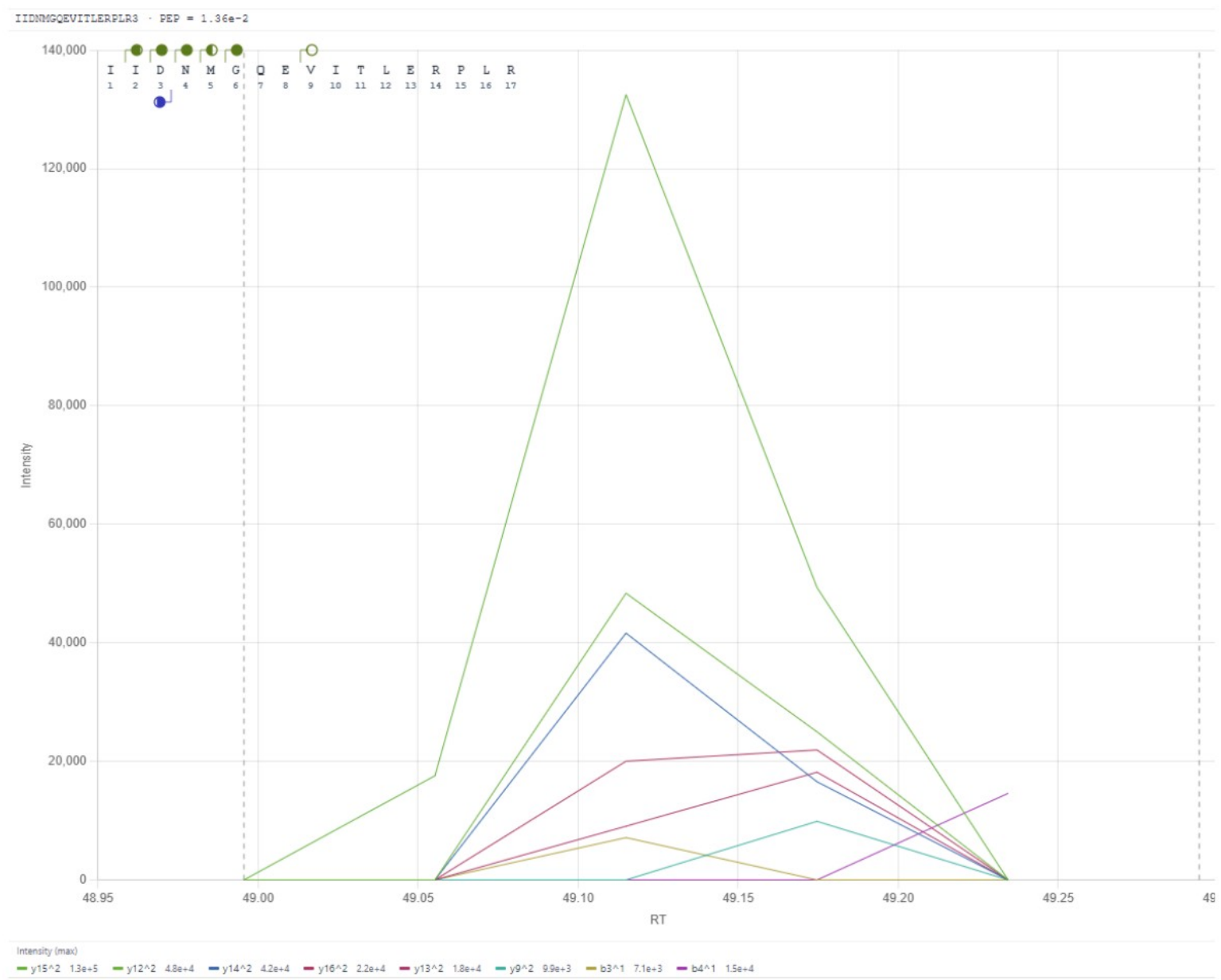

PMPCA

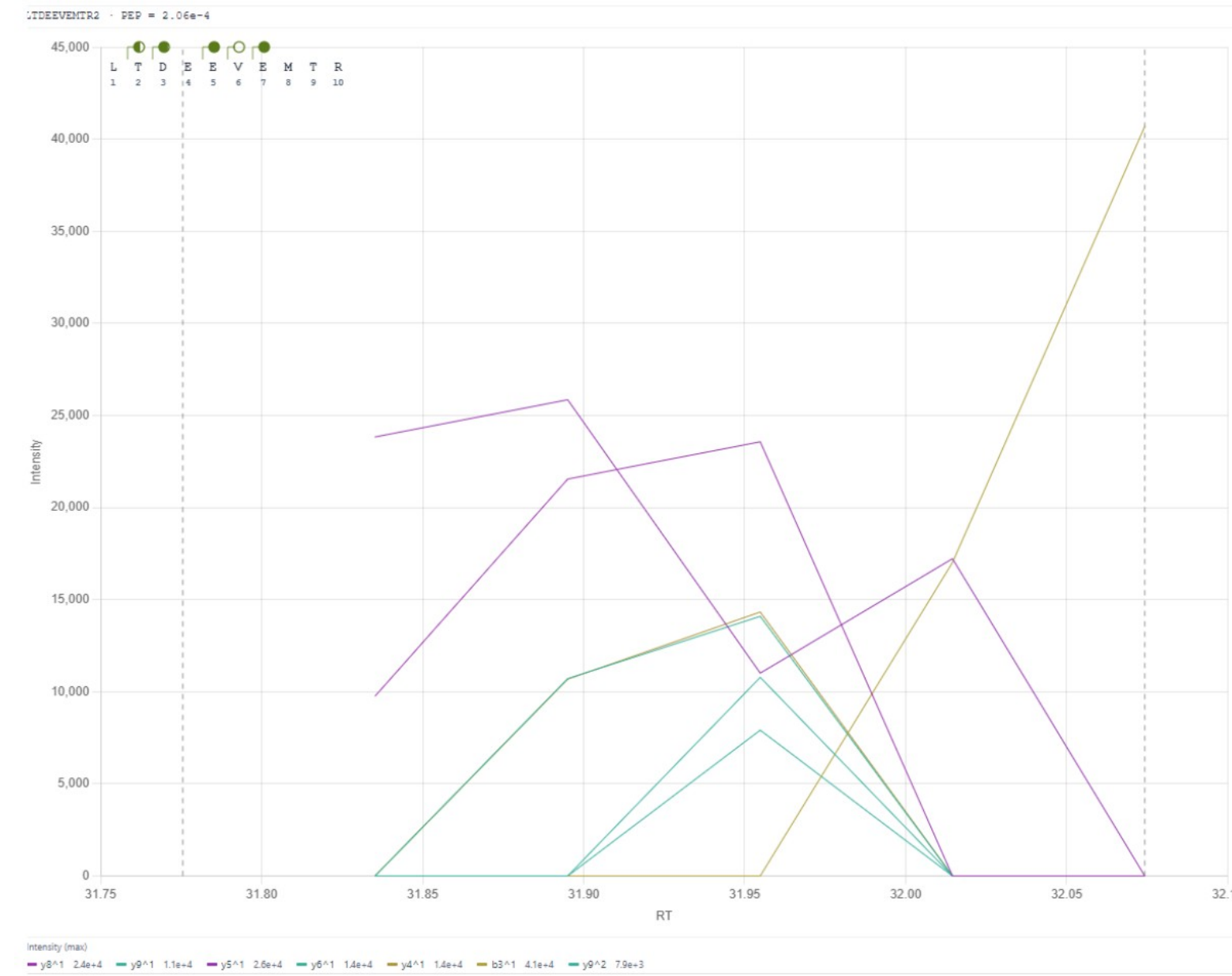

PPIF

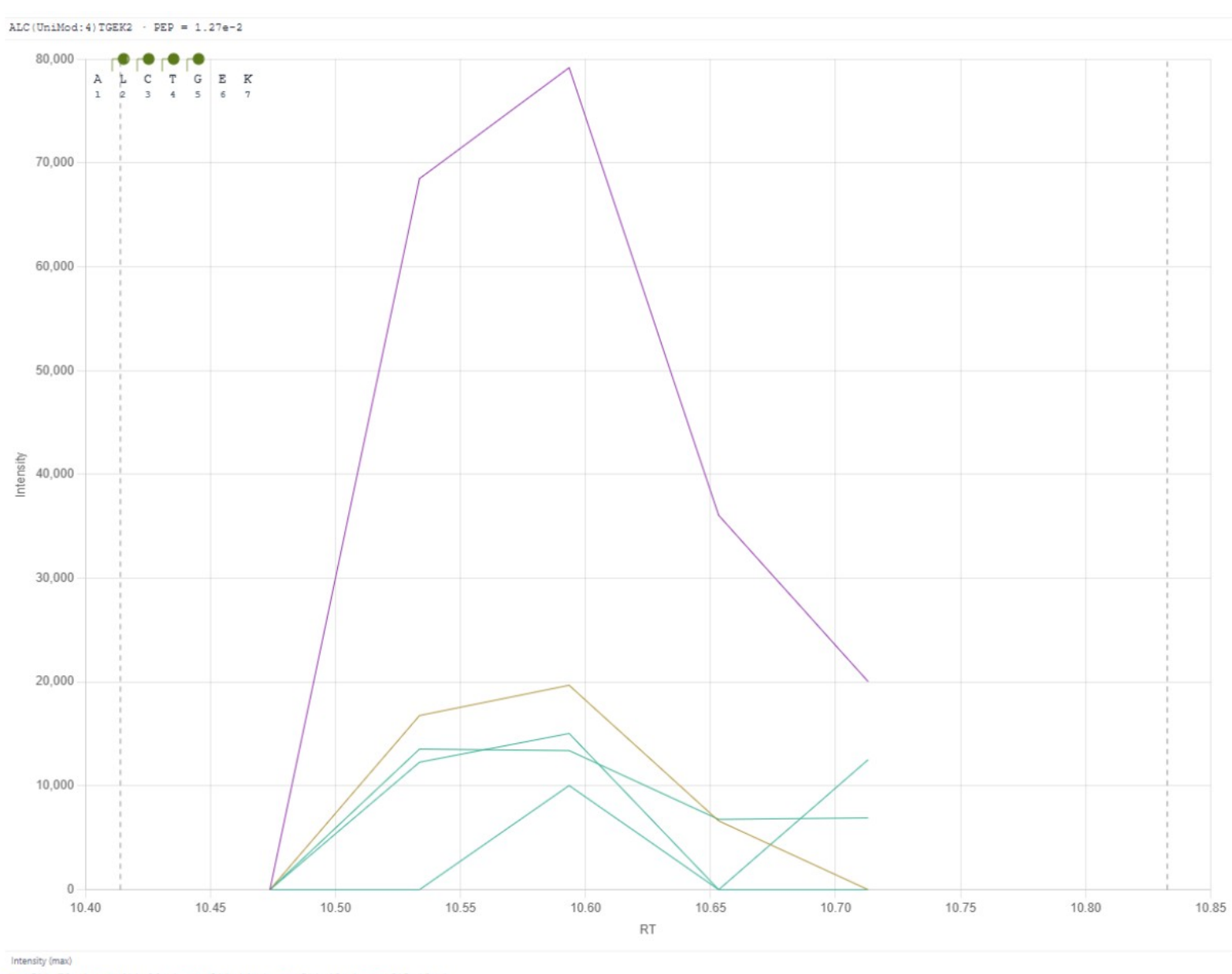

PPIH

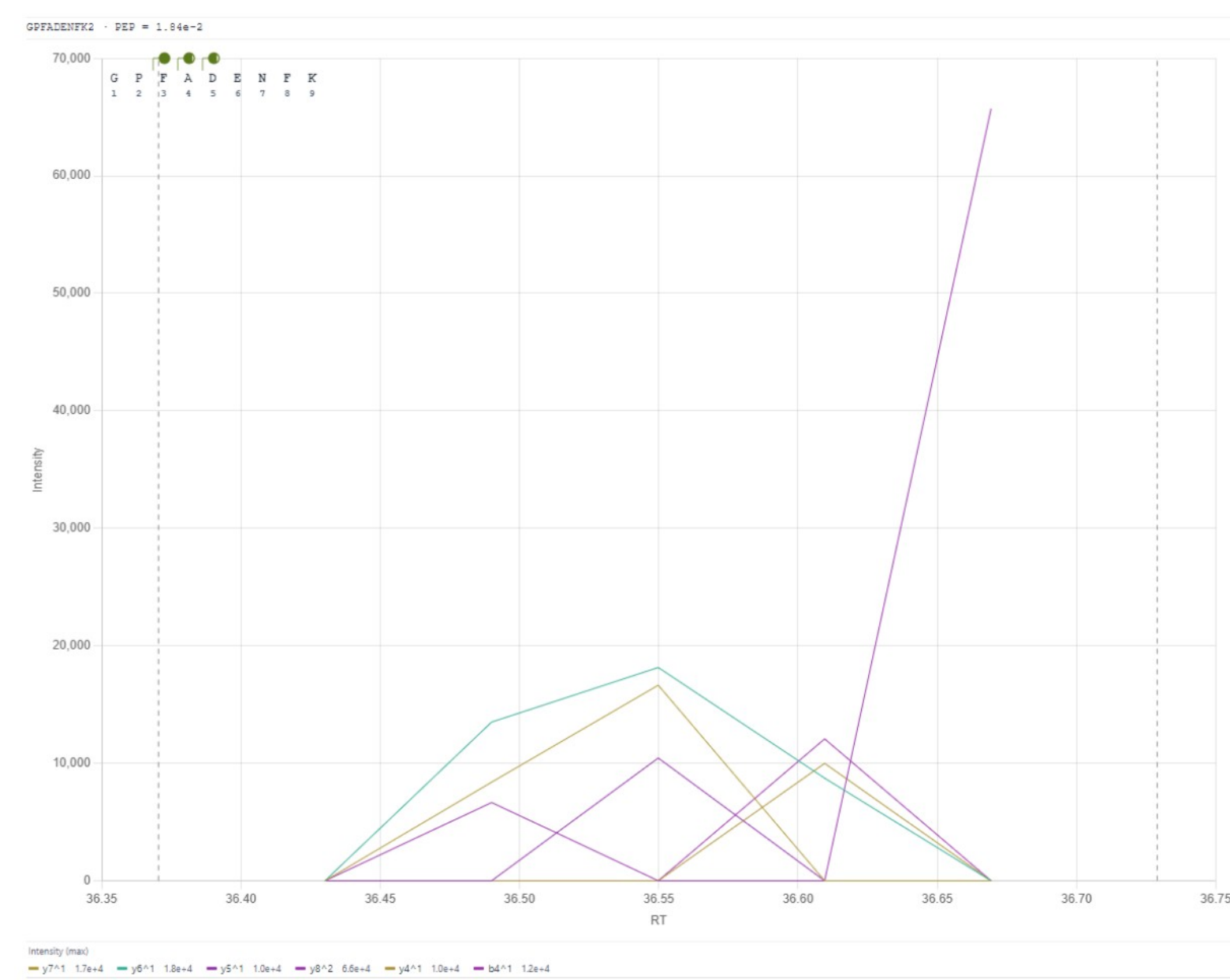

PPP1R12A

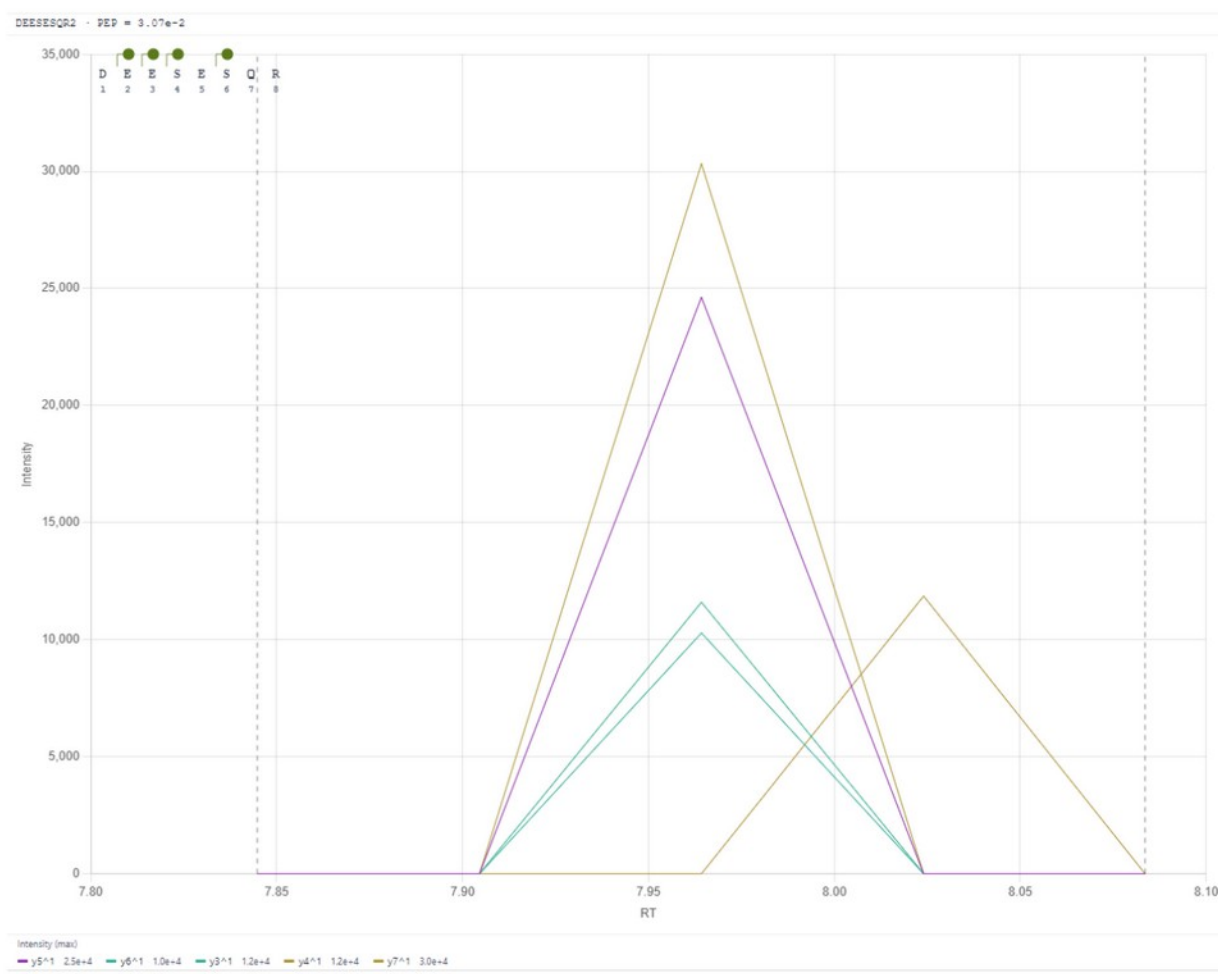

PPP2R5D

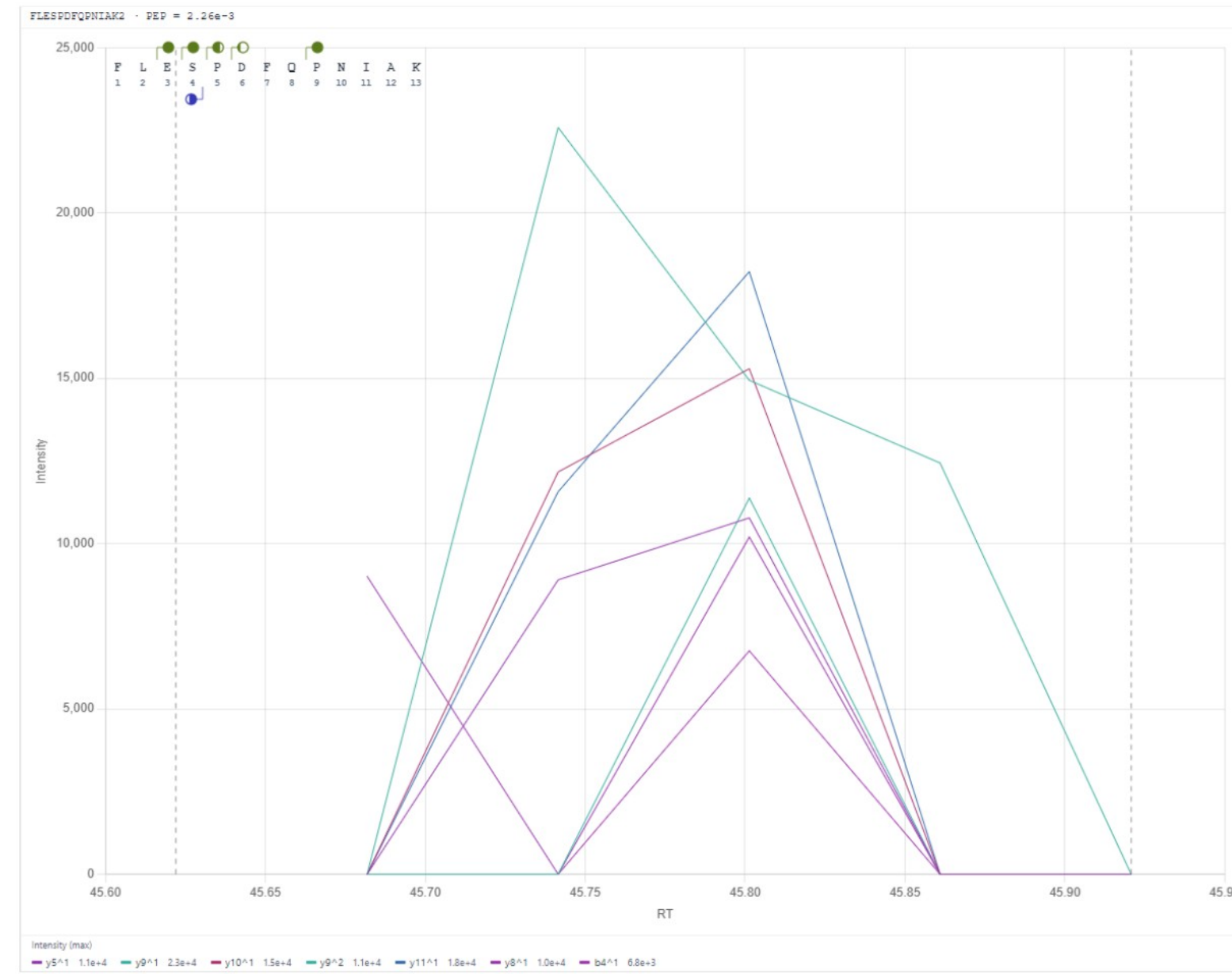

PPP2R5E

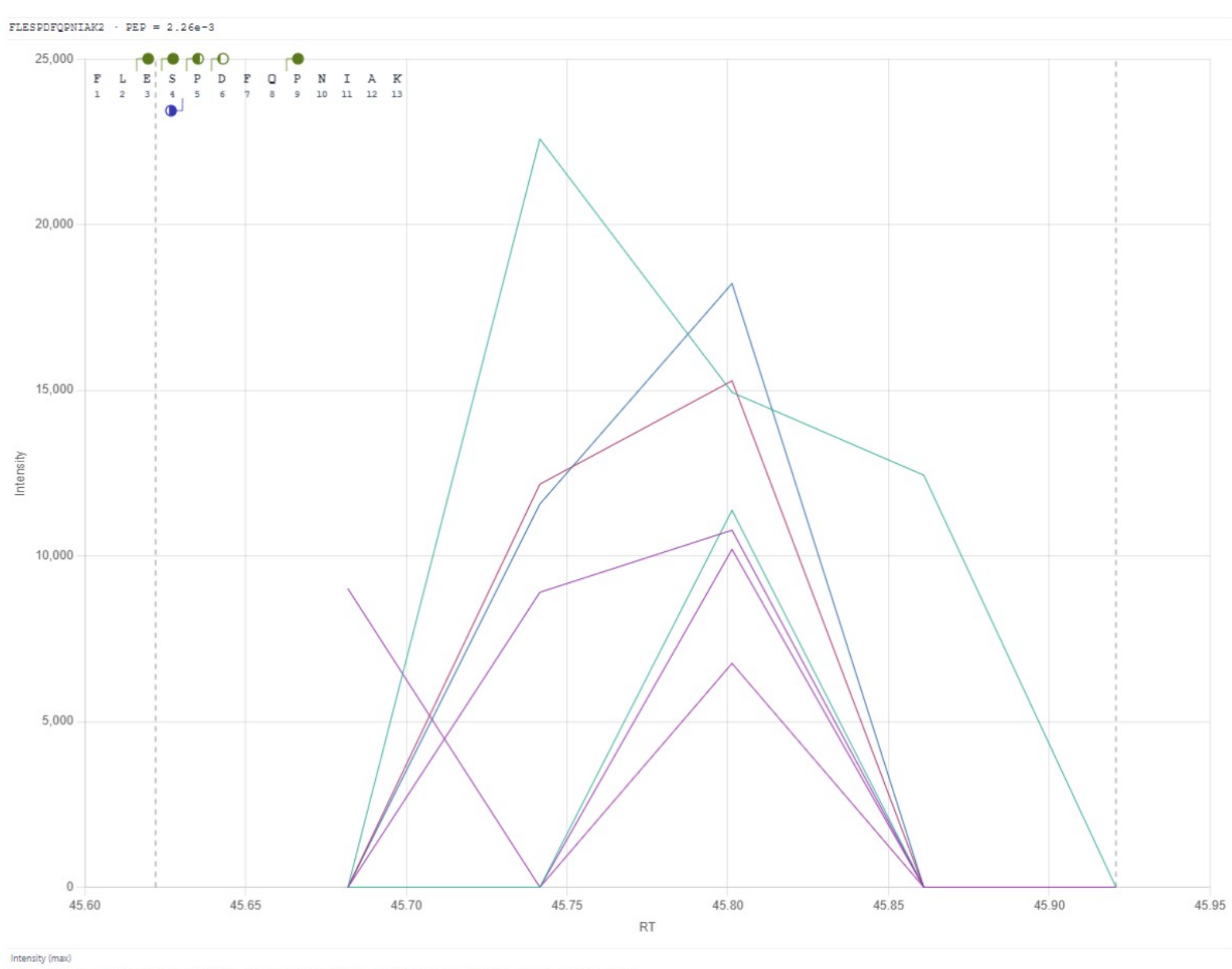

PPT1

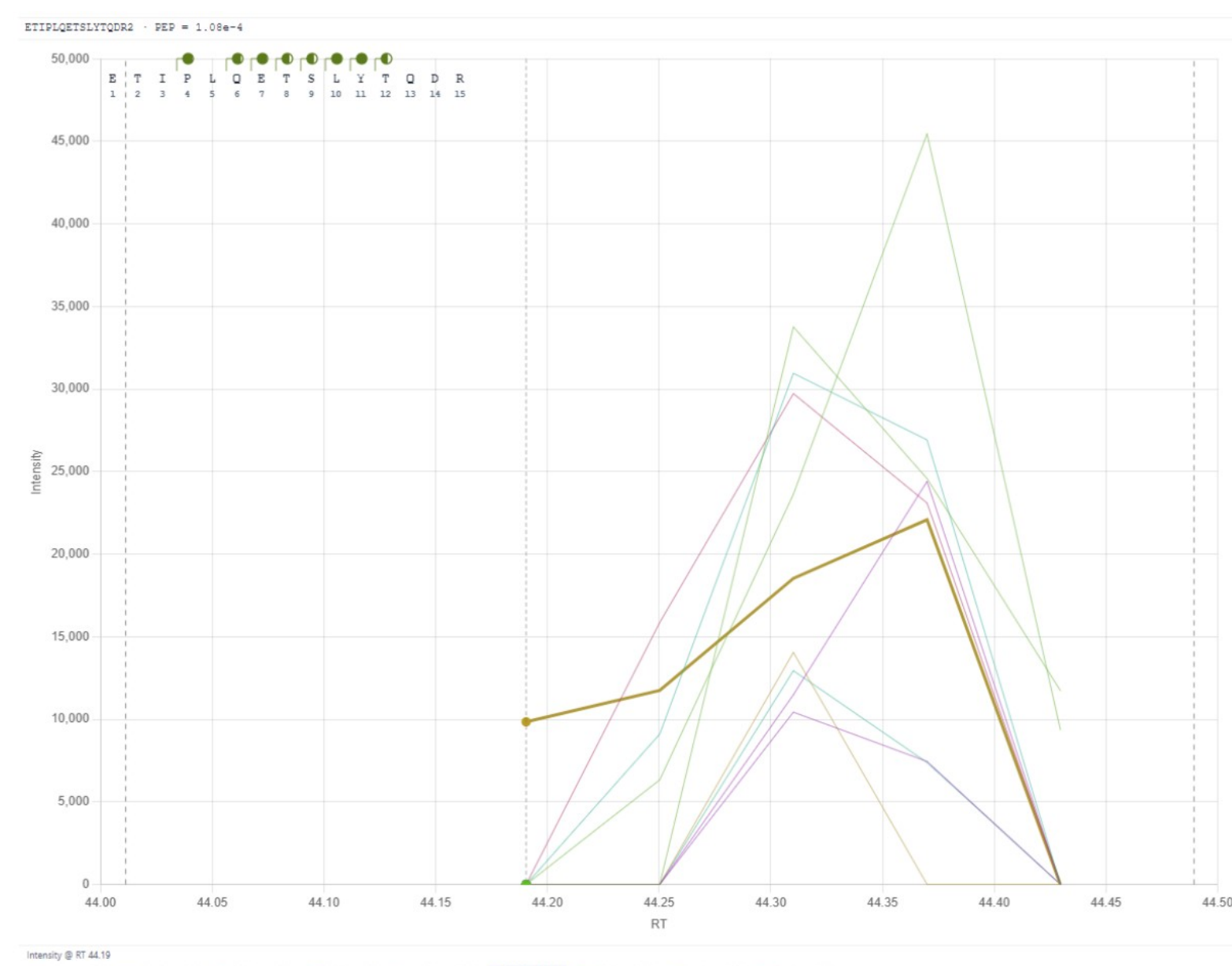

PRDX1

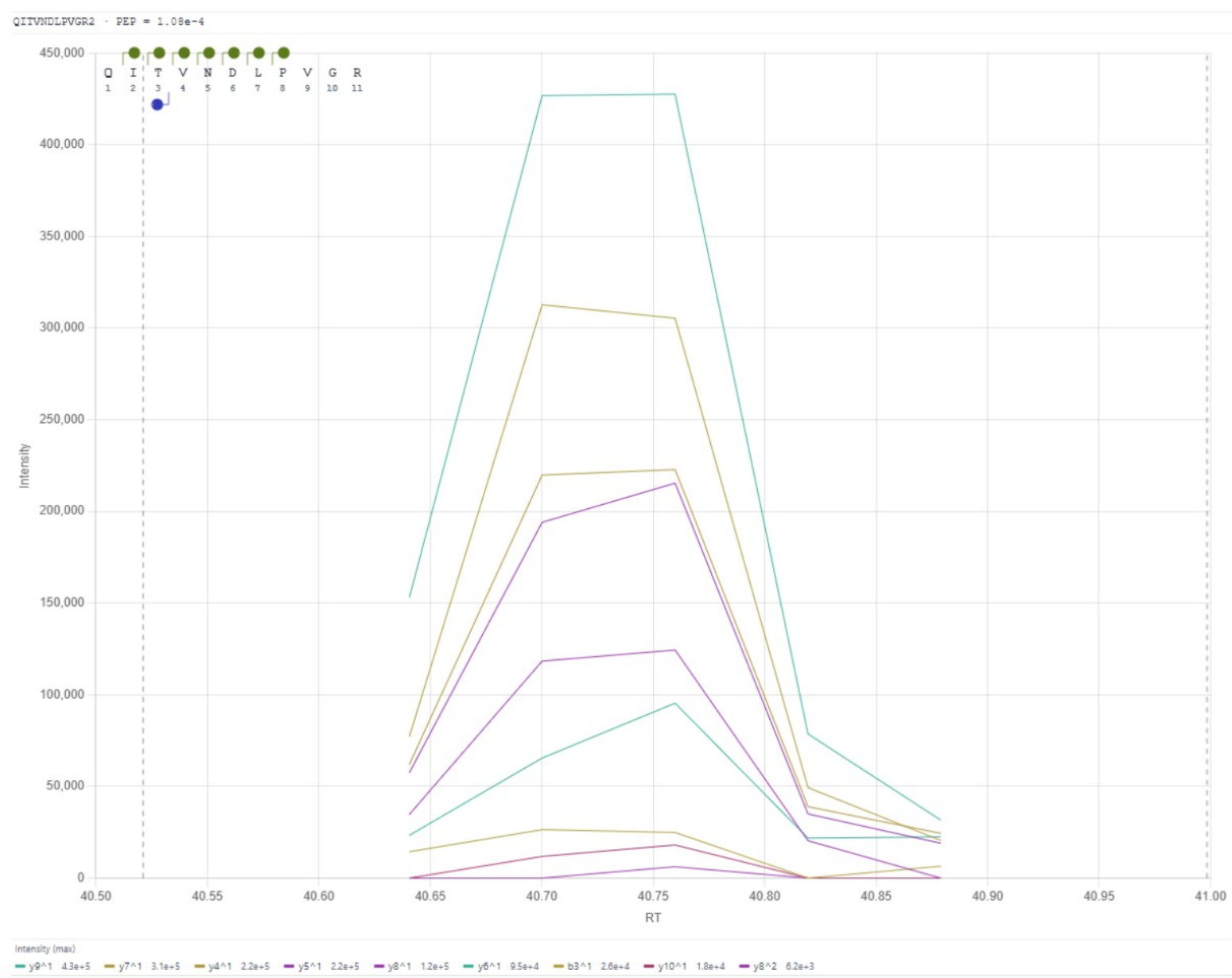

PREB

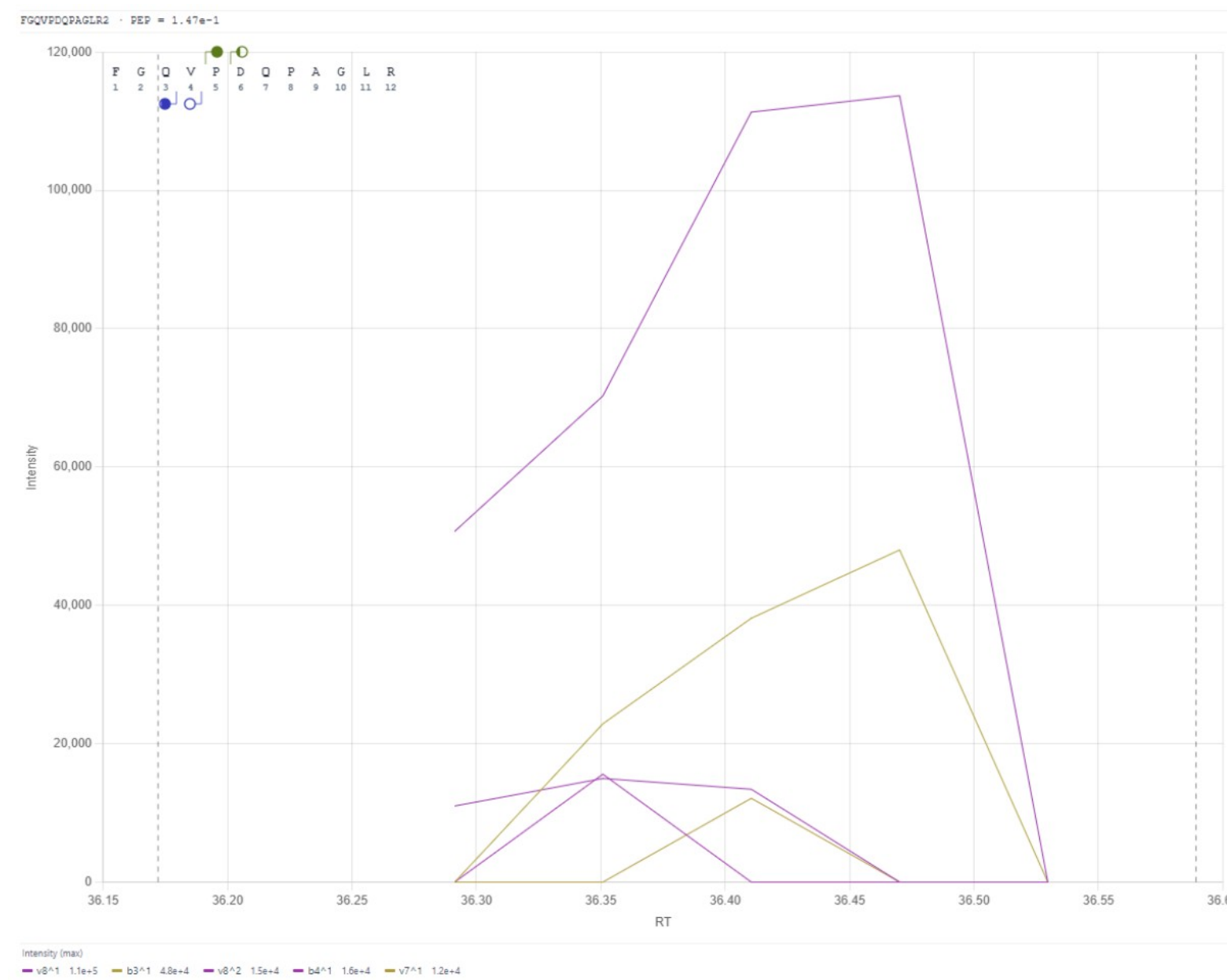

PRH1

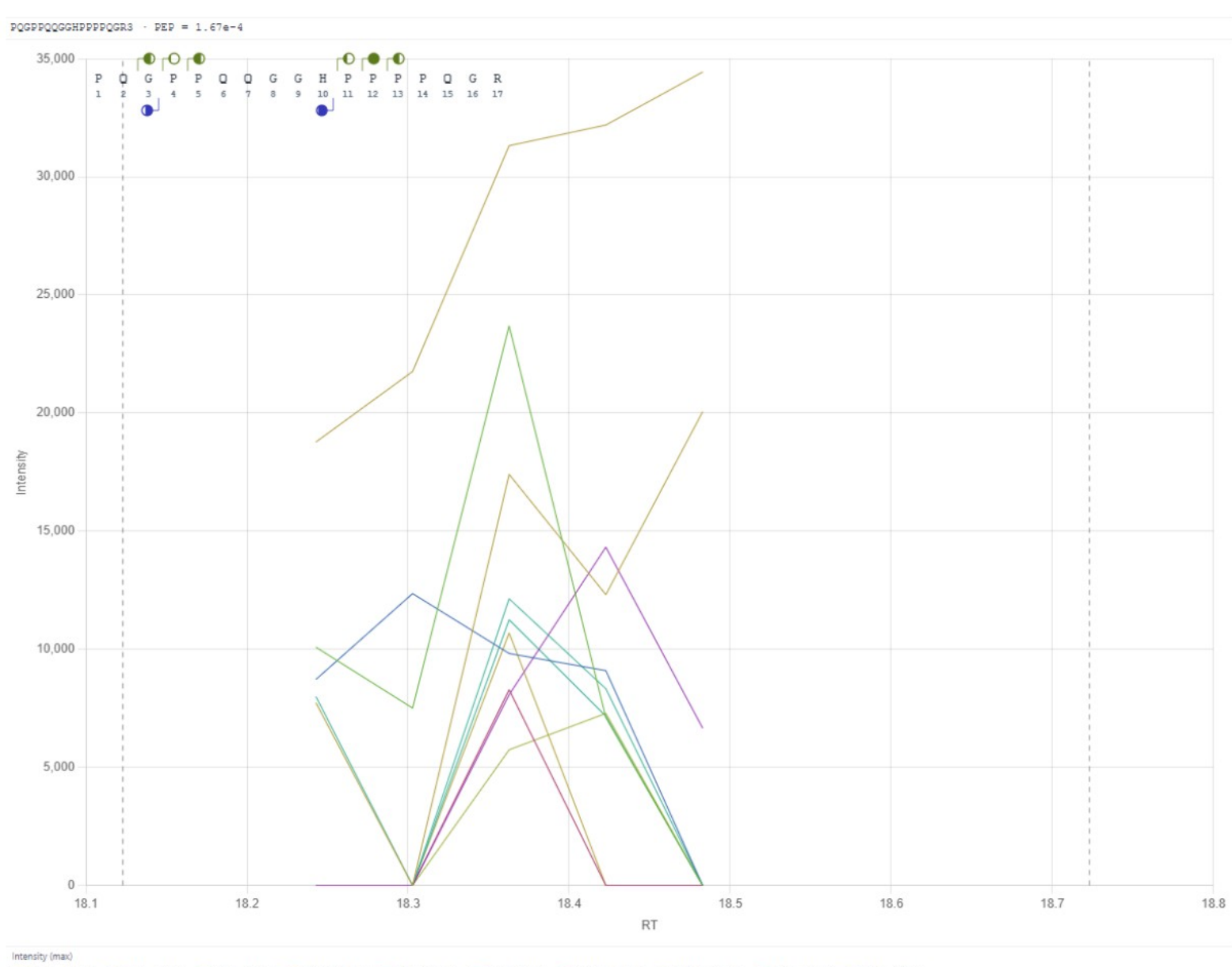

PRKCD

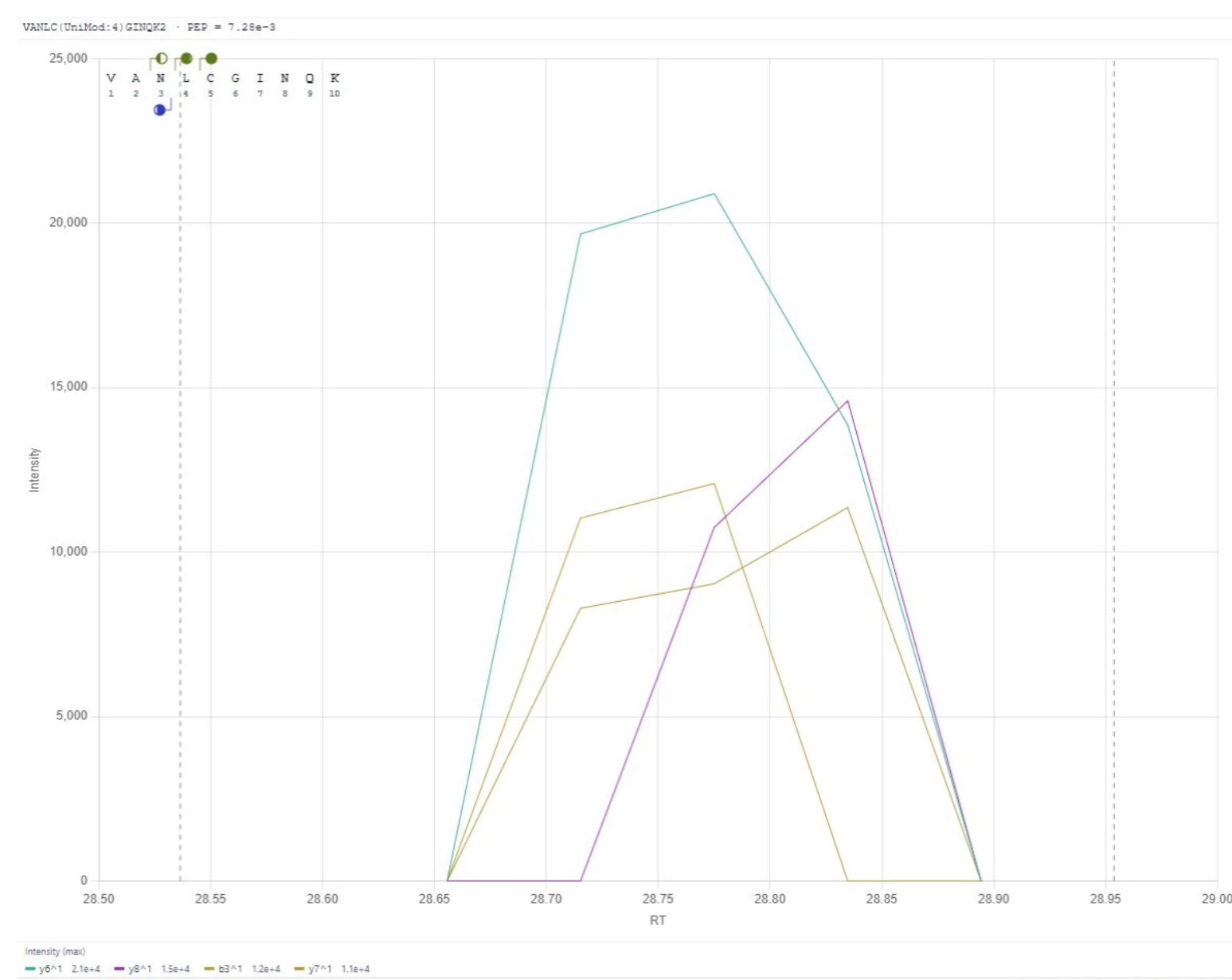

PRXL2B

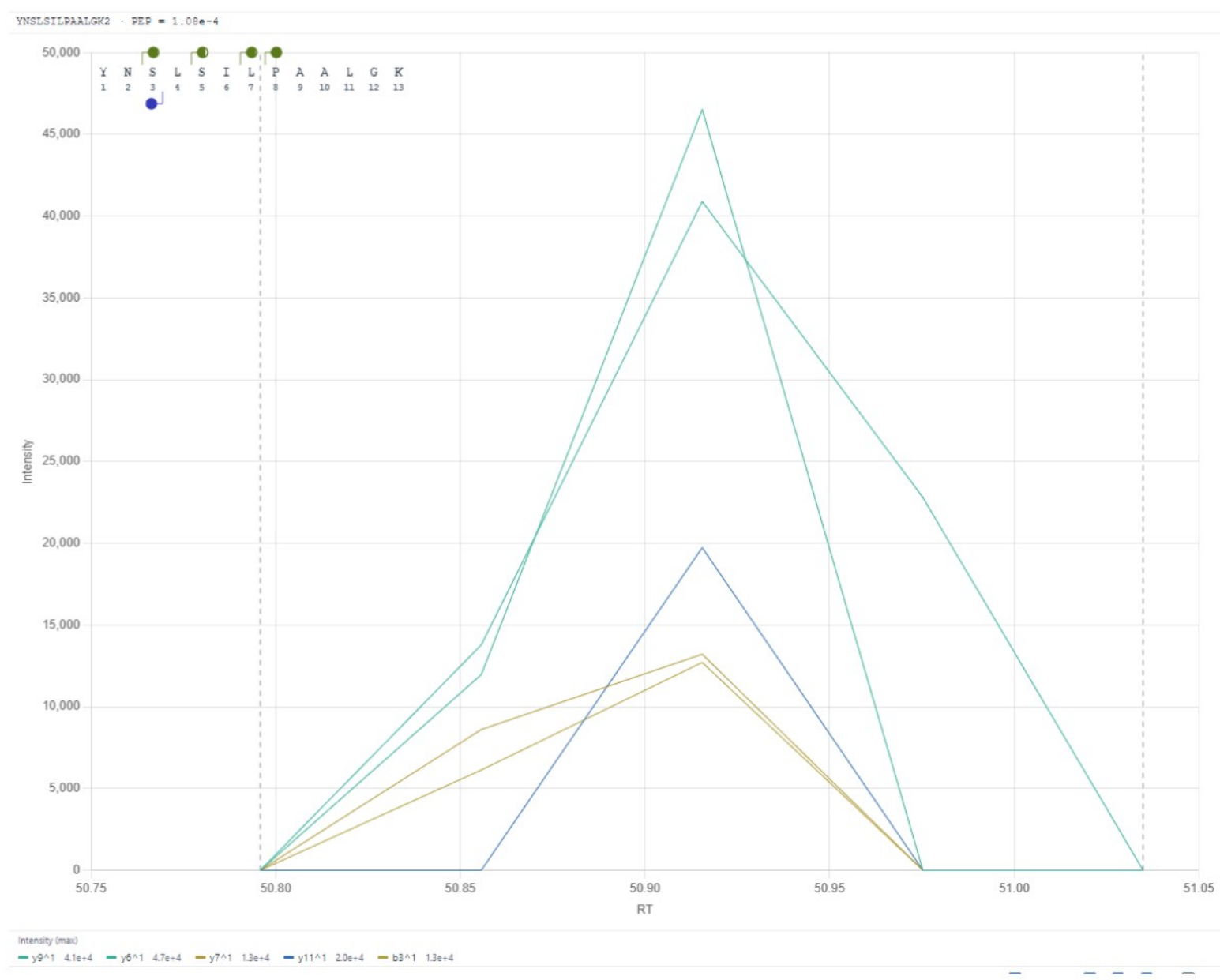

PTK2

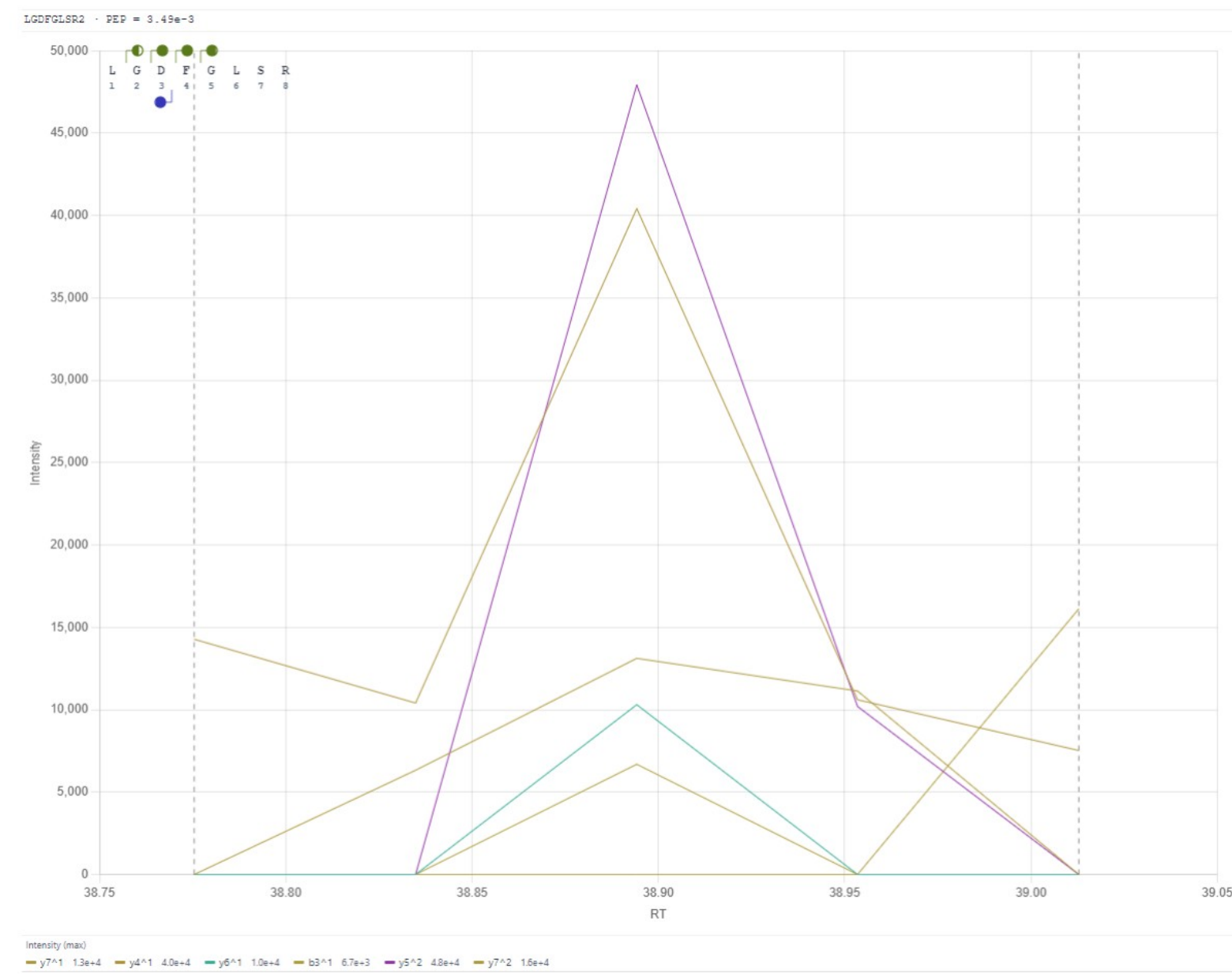

RAB8A

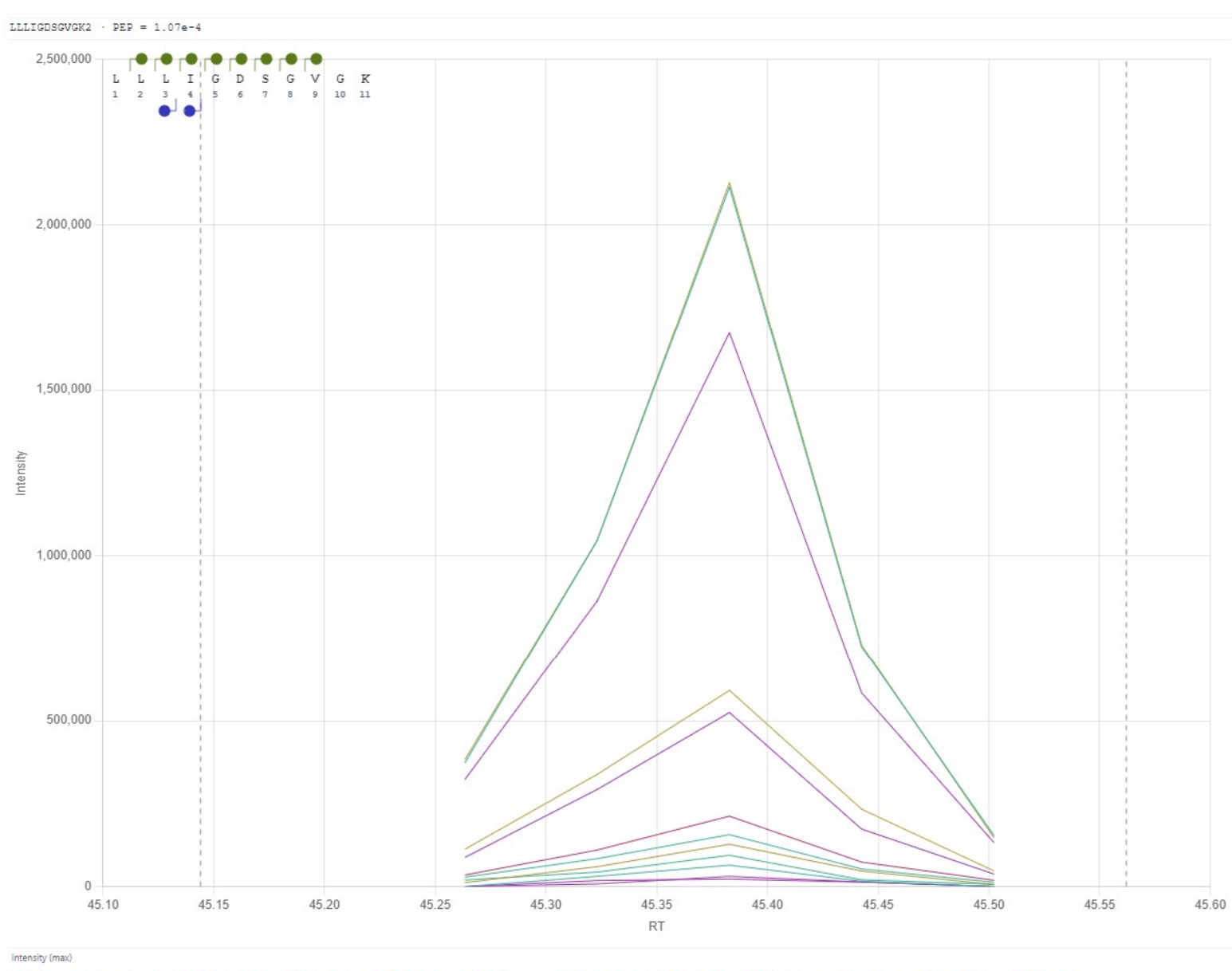

RAB5A

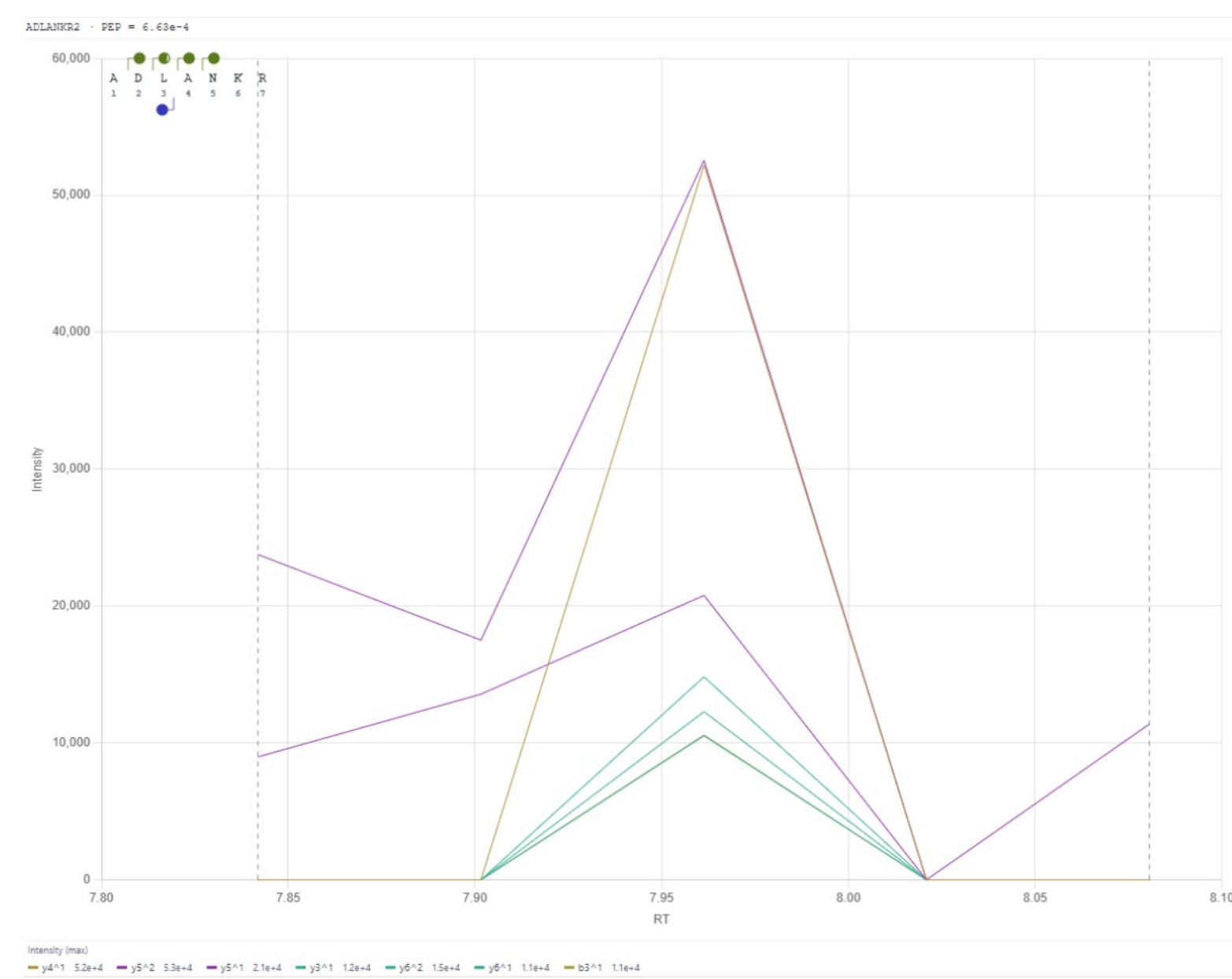

RABGEF1

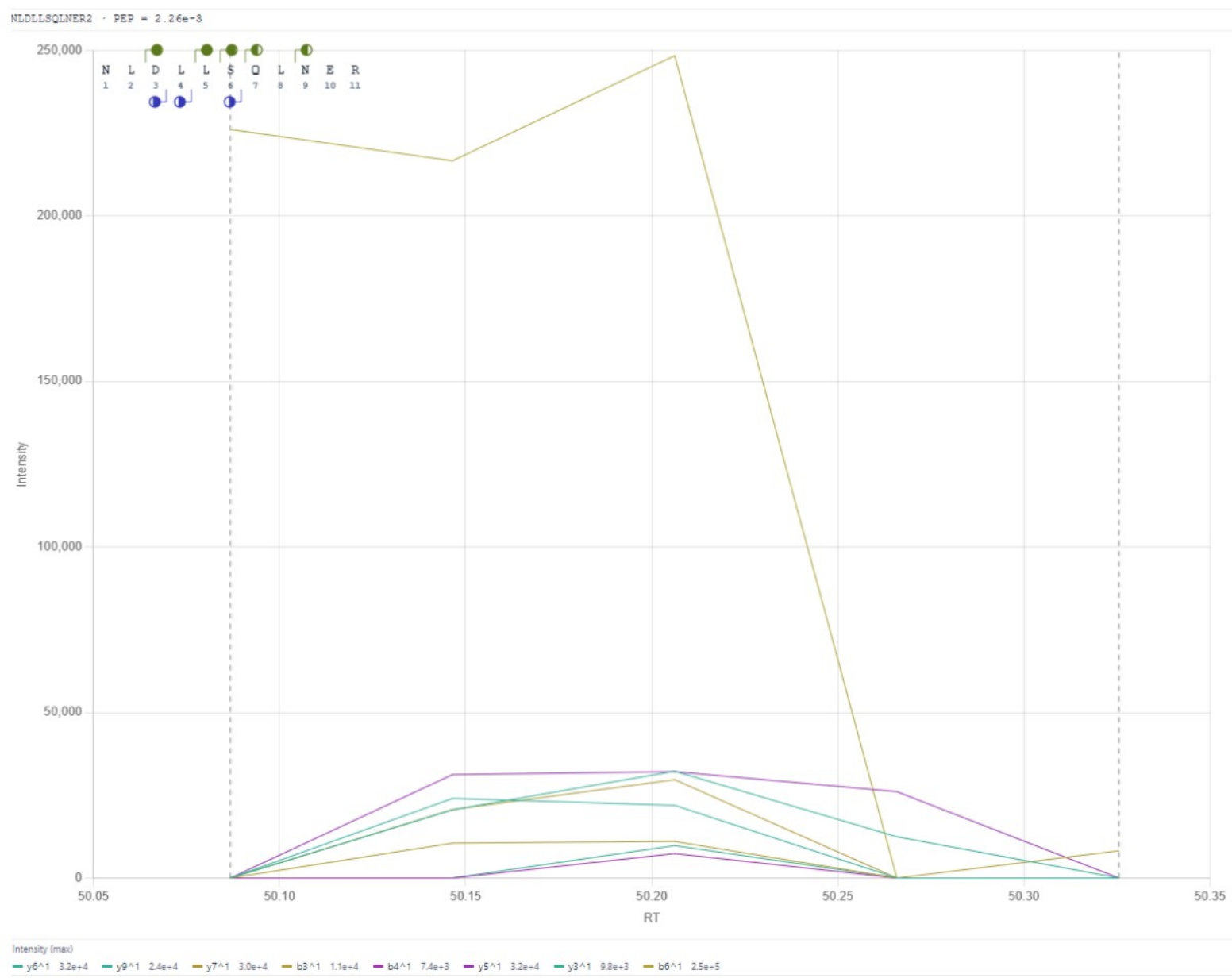

RBBP9

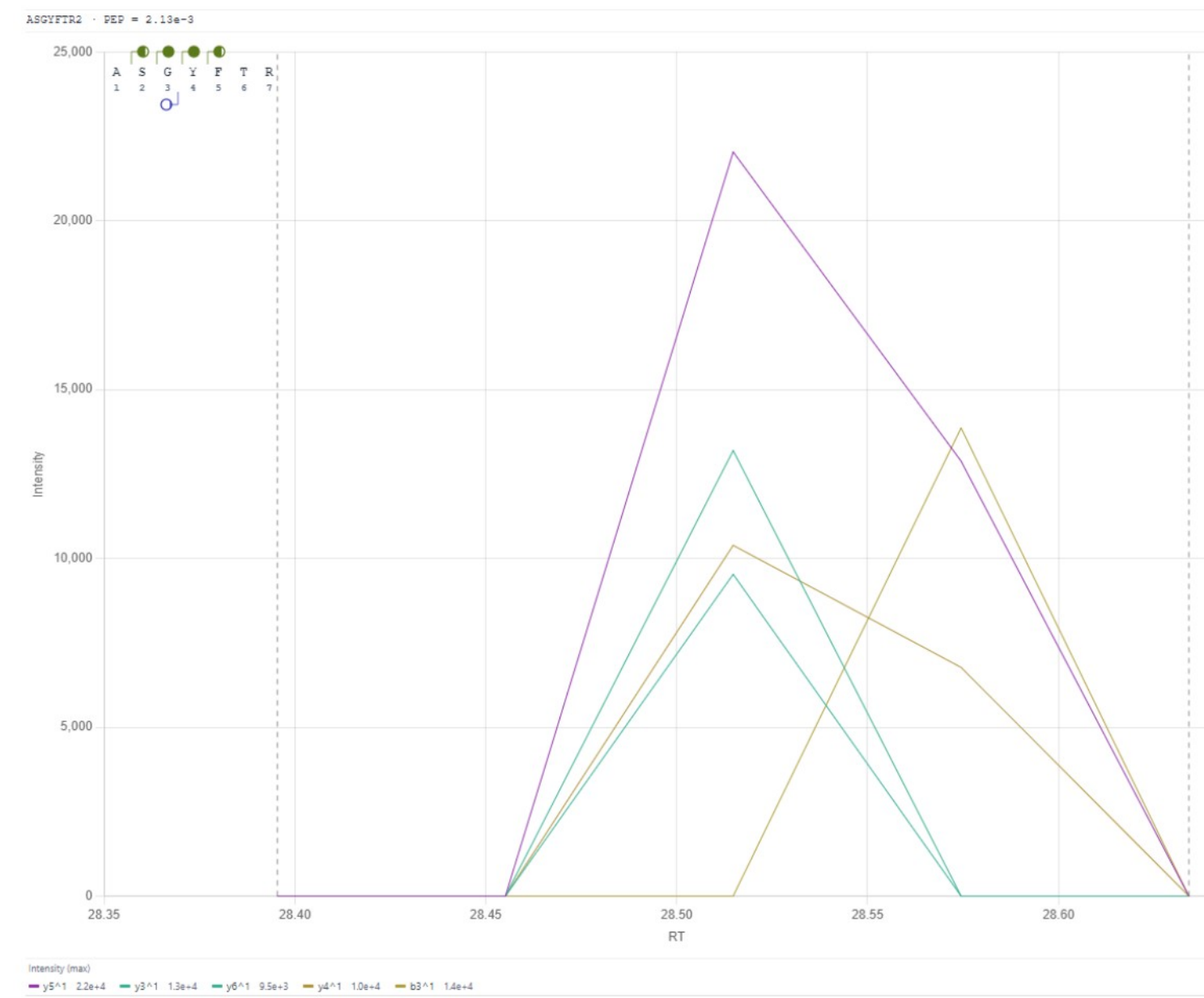

RGS5

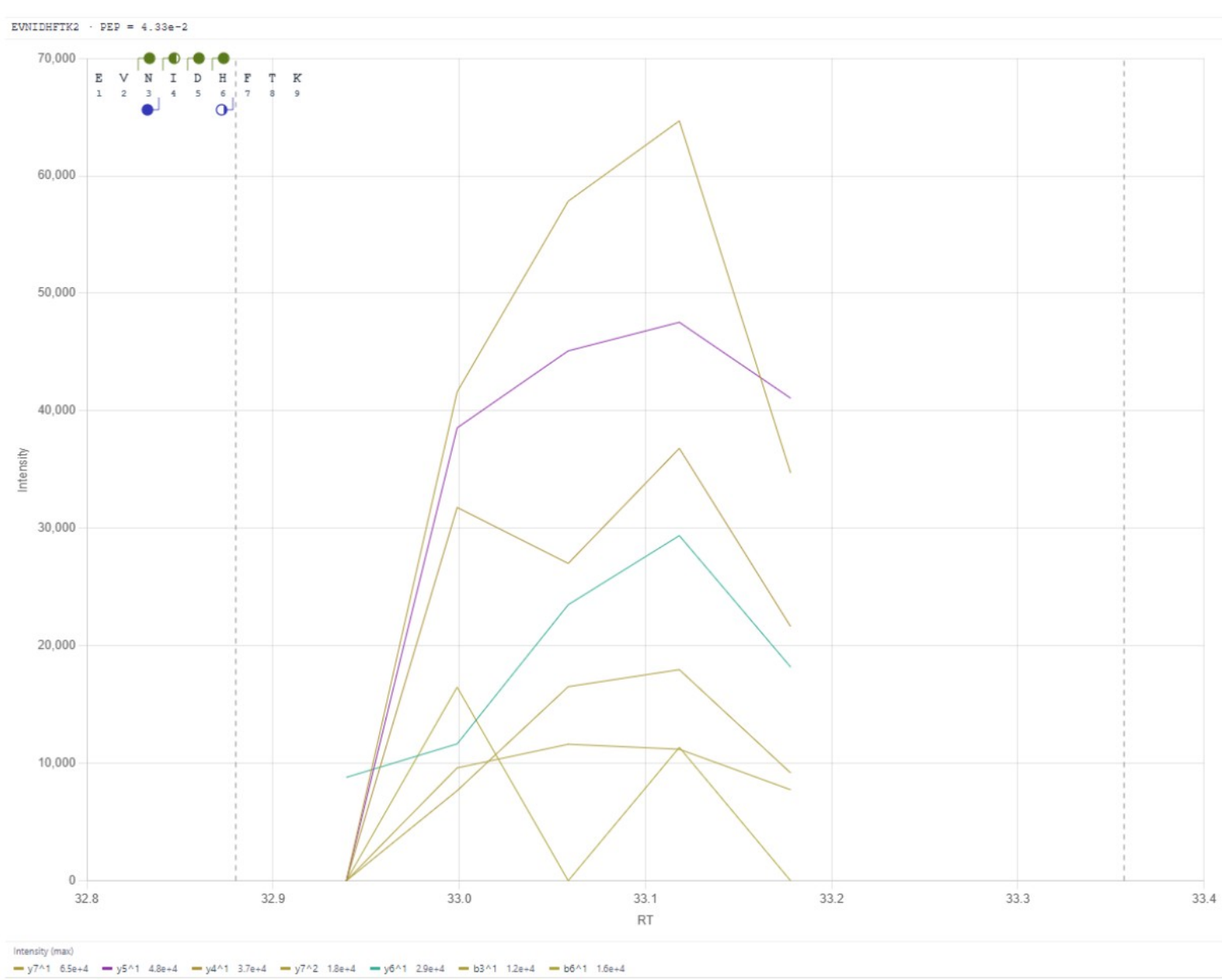

RH1

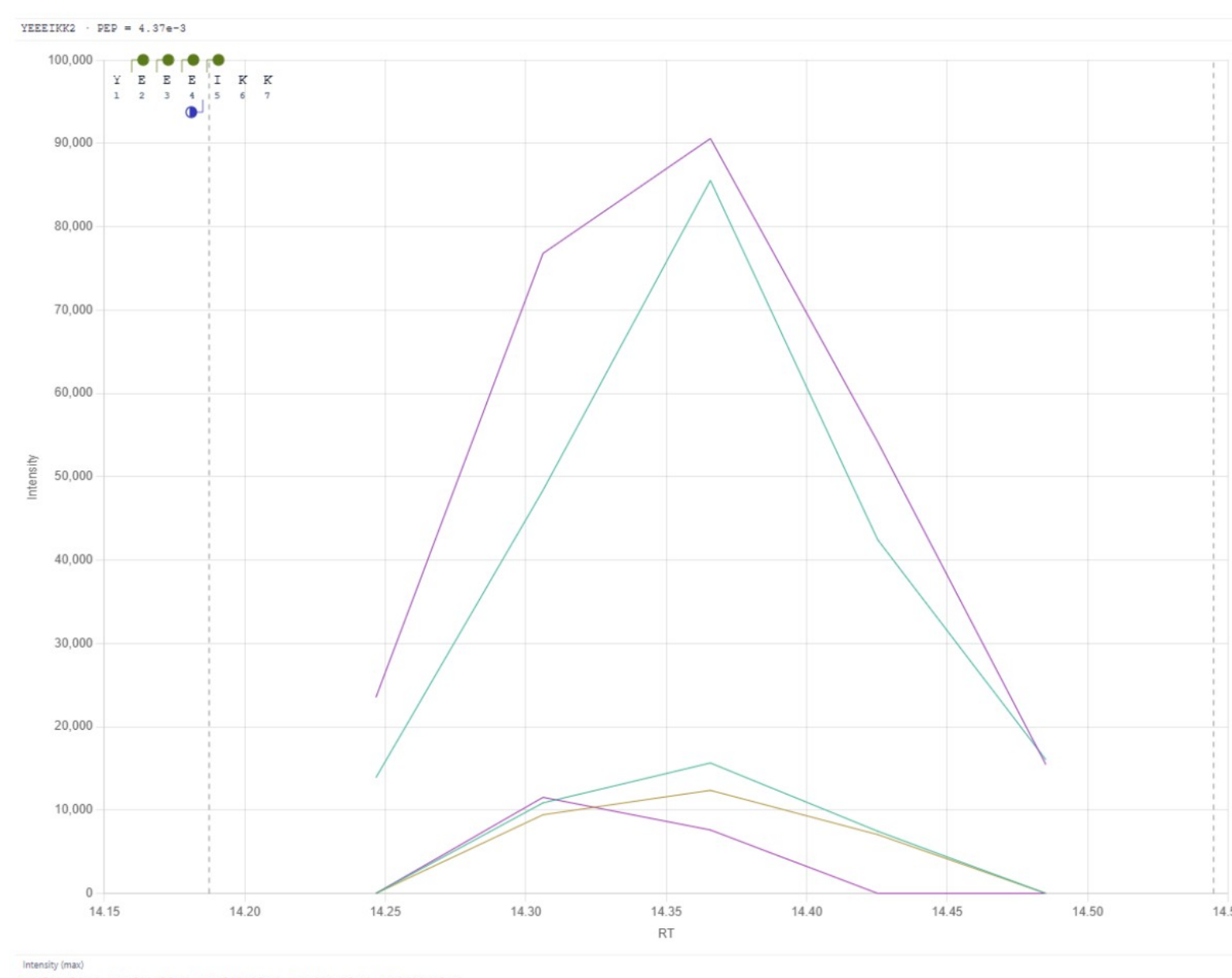

RHOT2

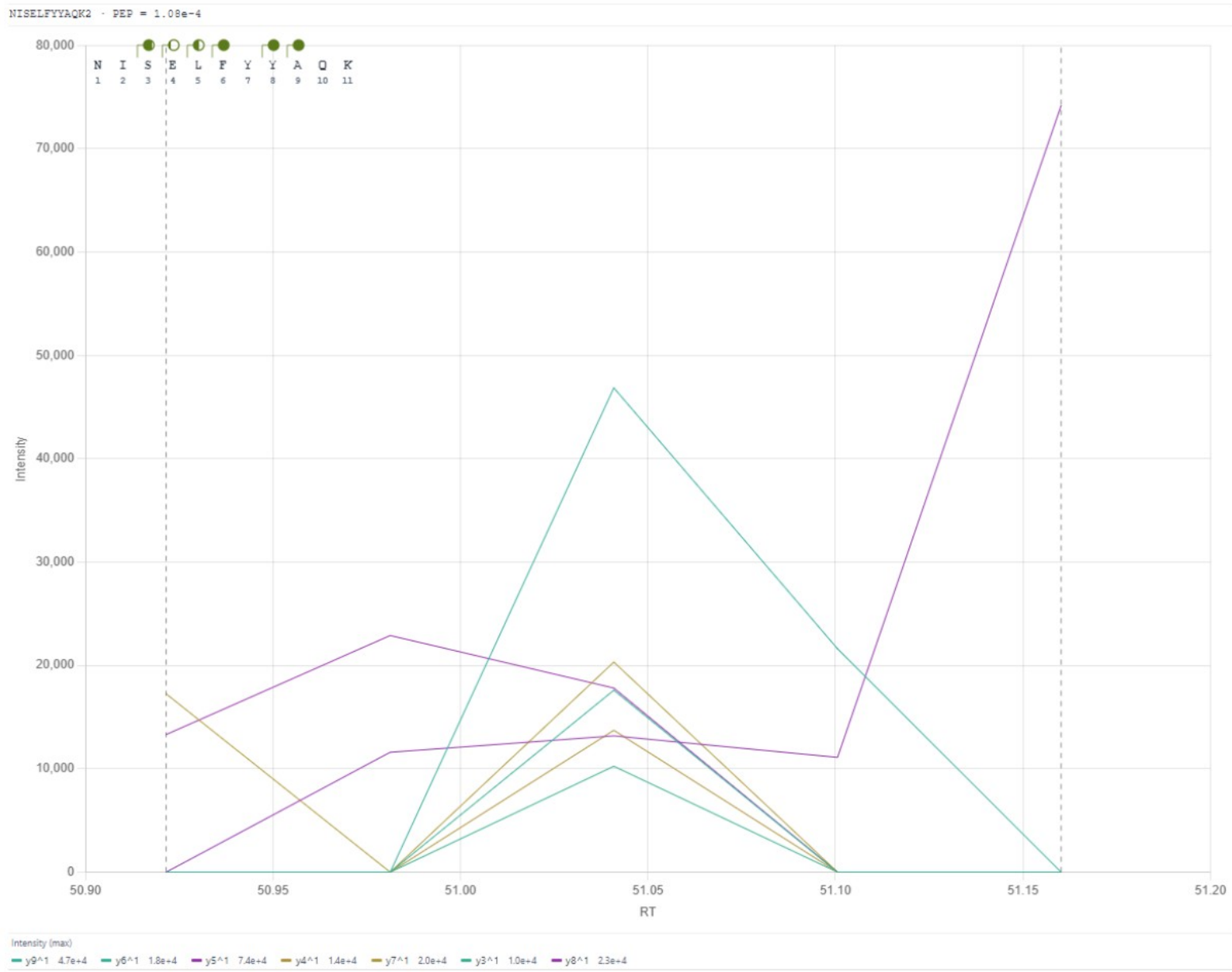

RNF31

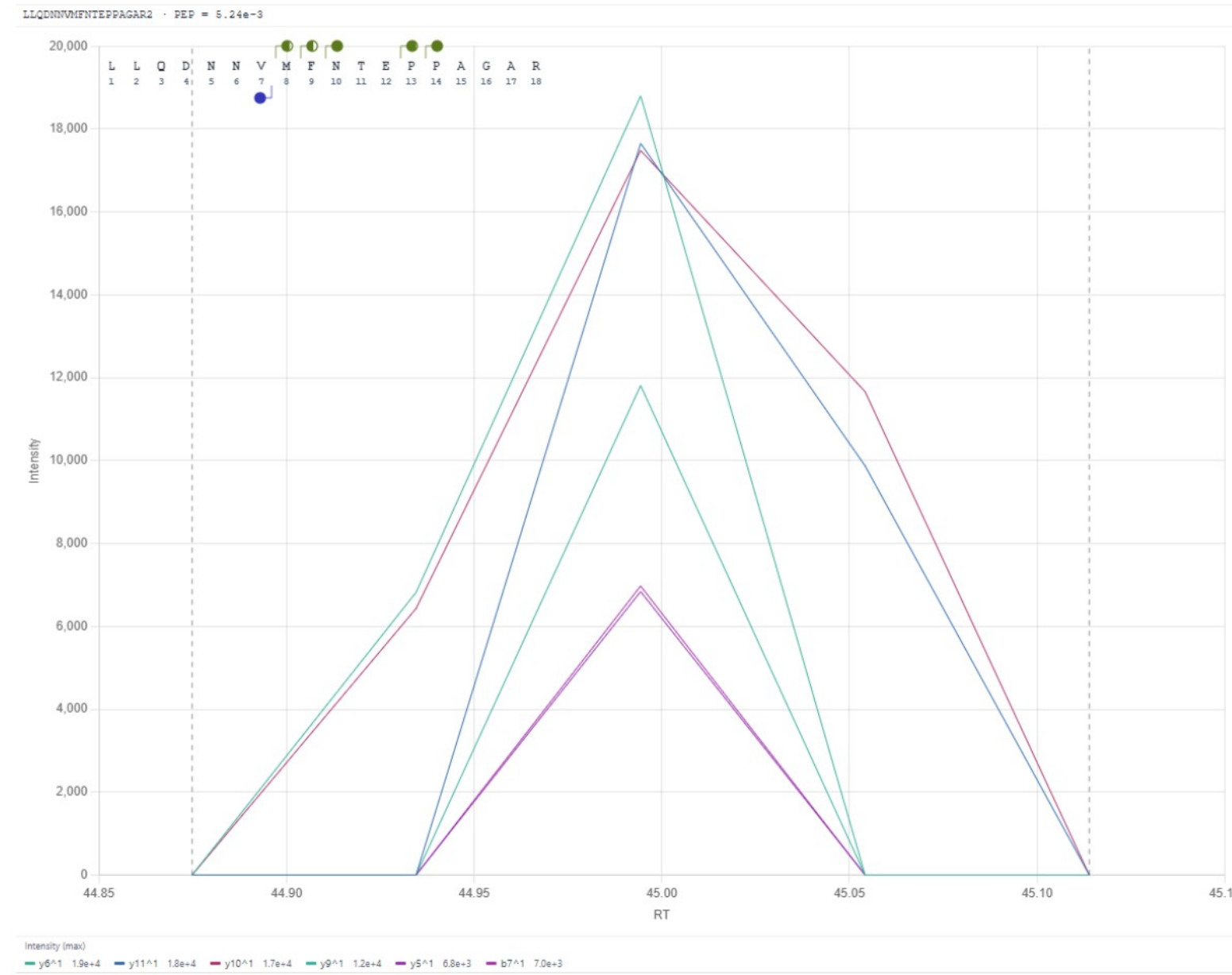

rny

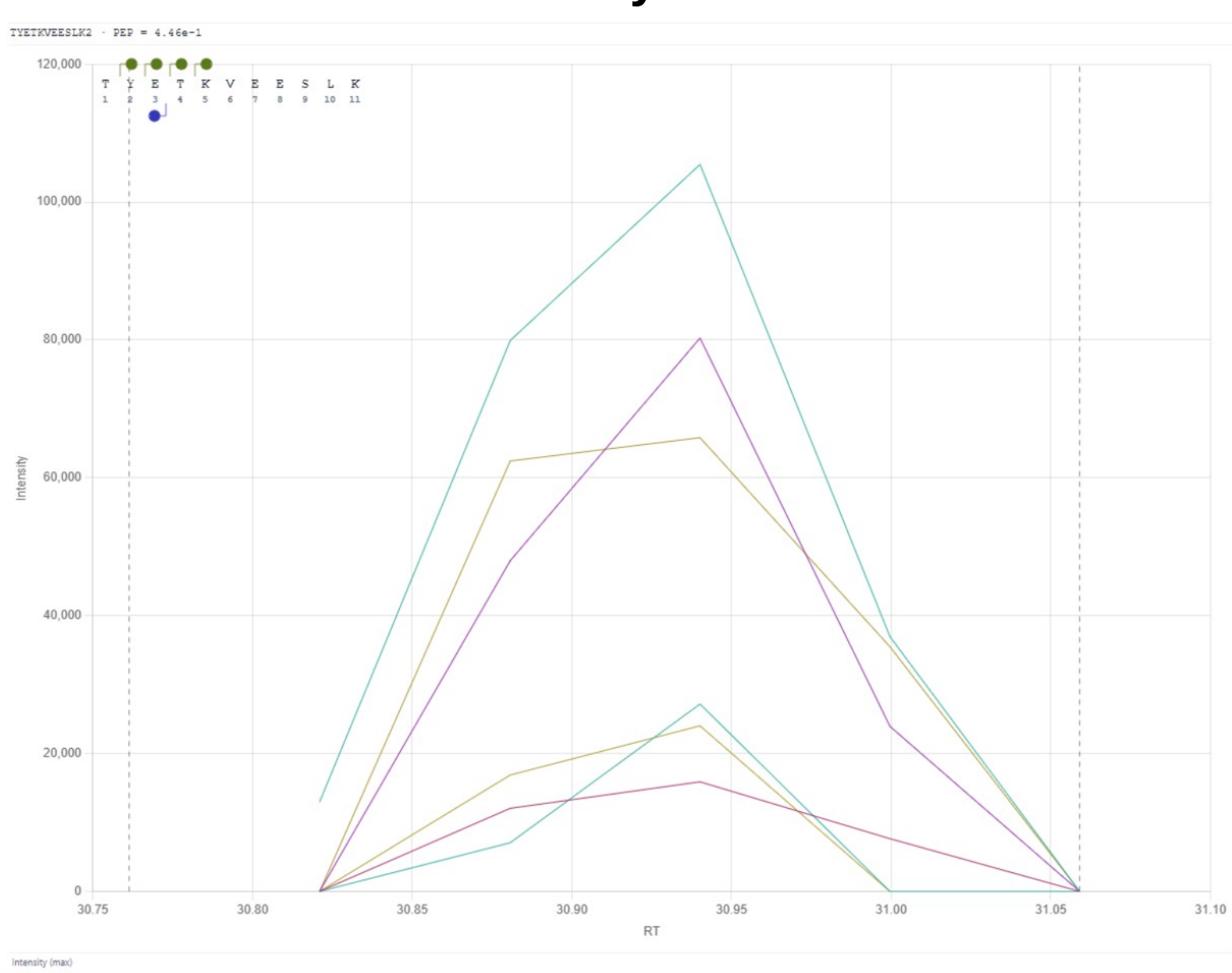

RPLP2

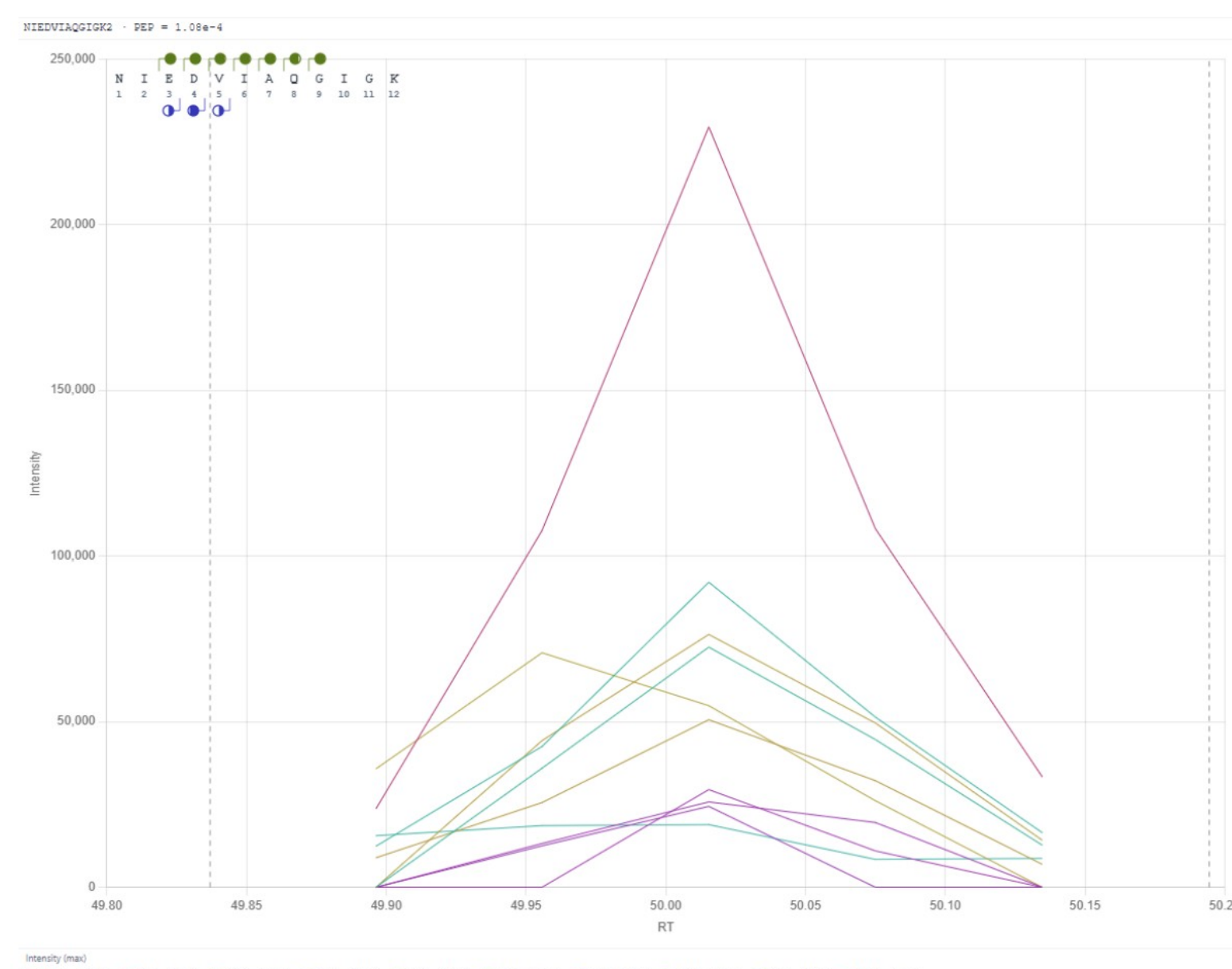

RPS28

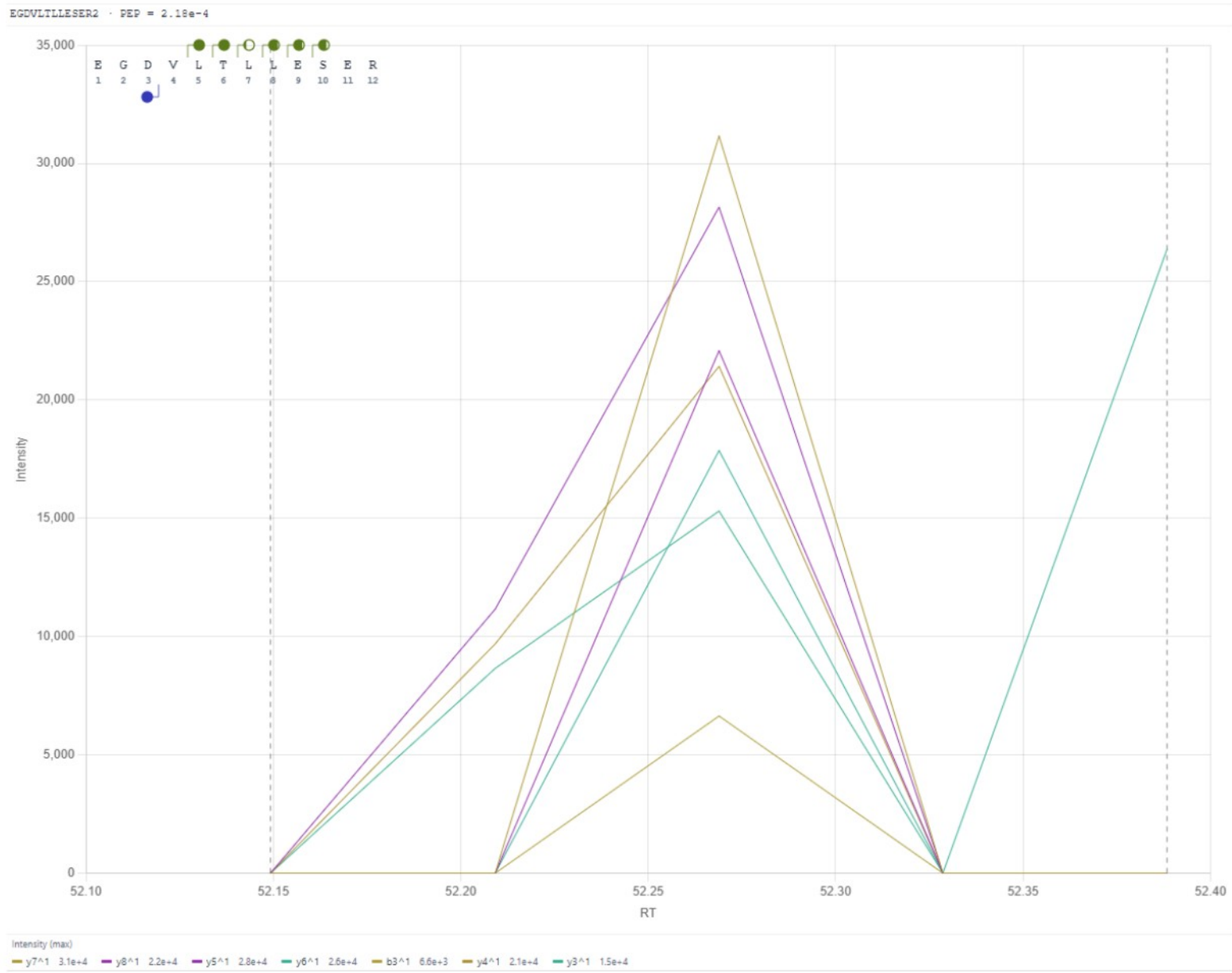

RRAS

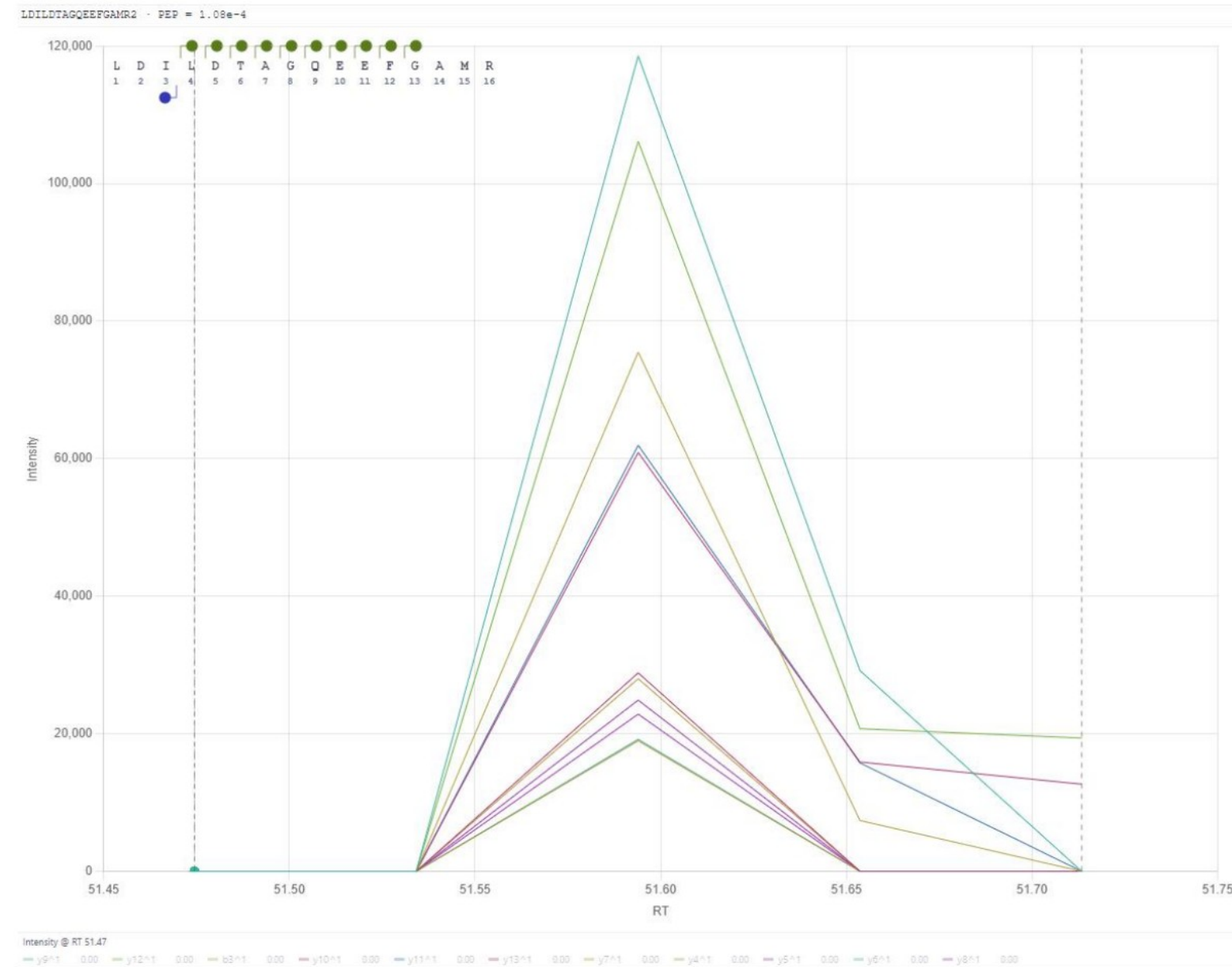

SCAMP1

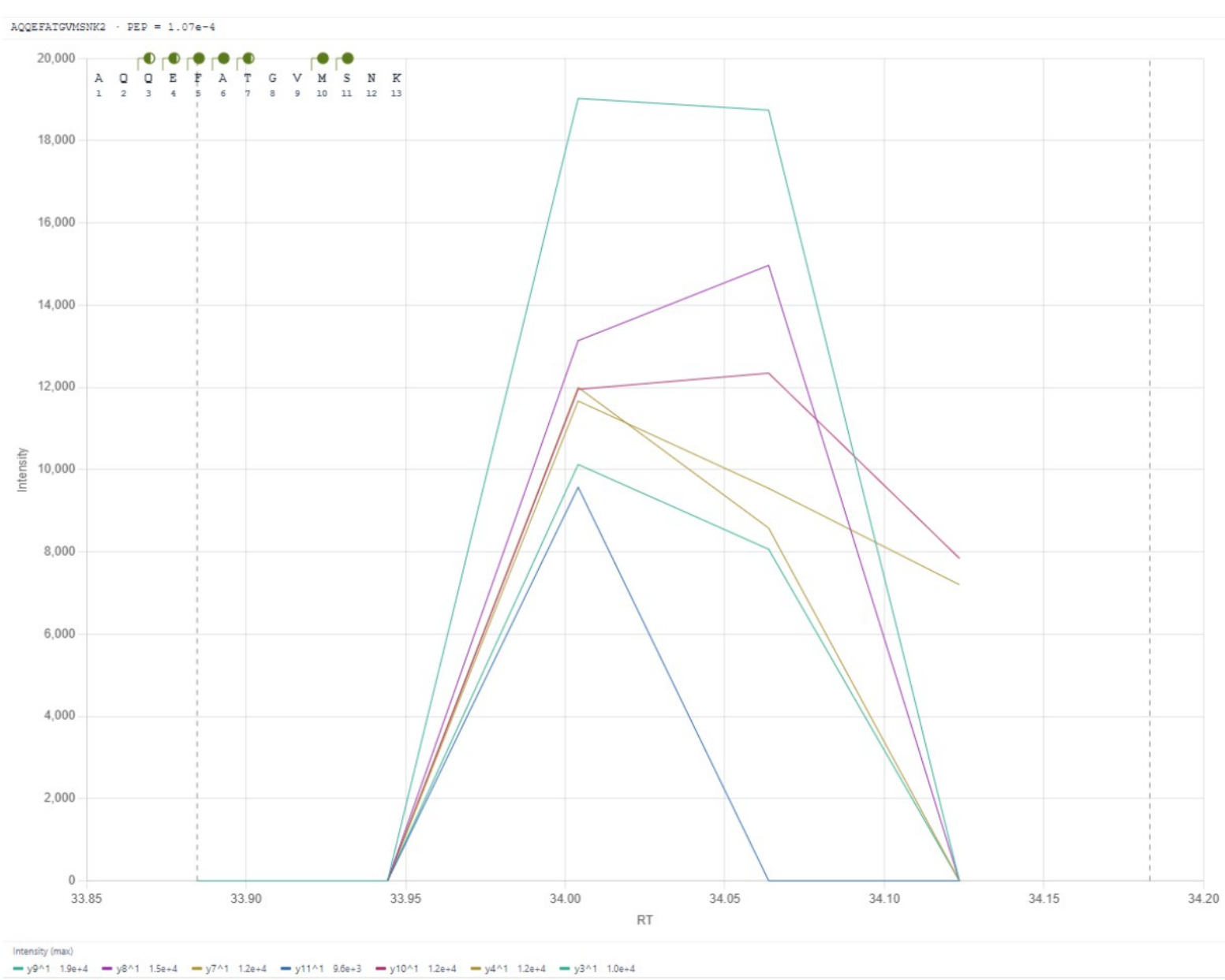

SCAMP4

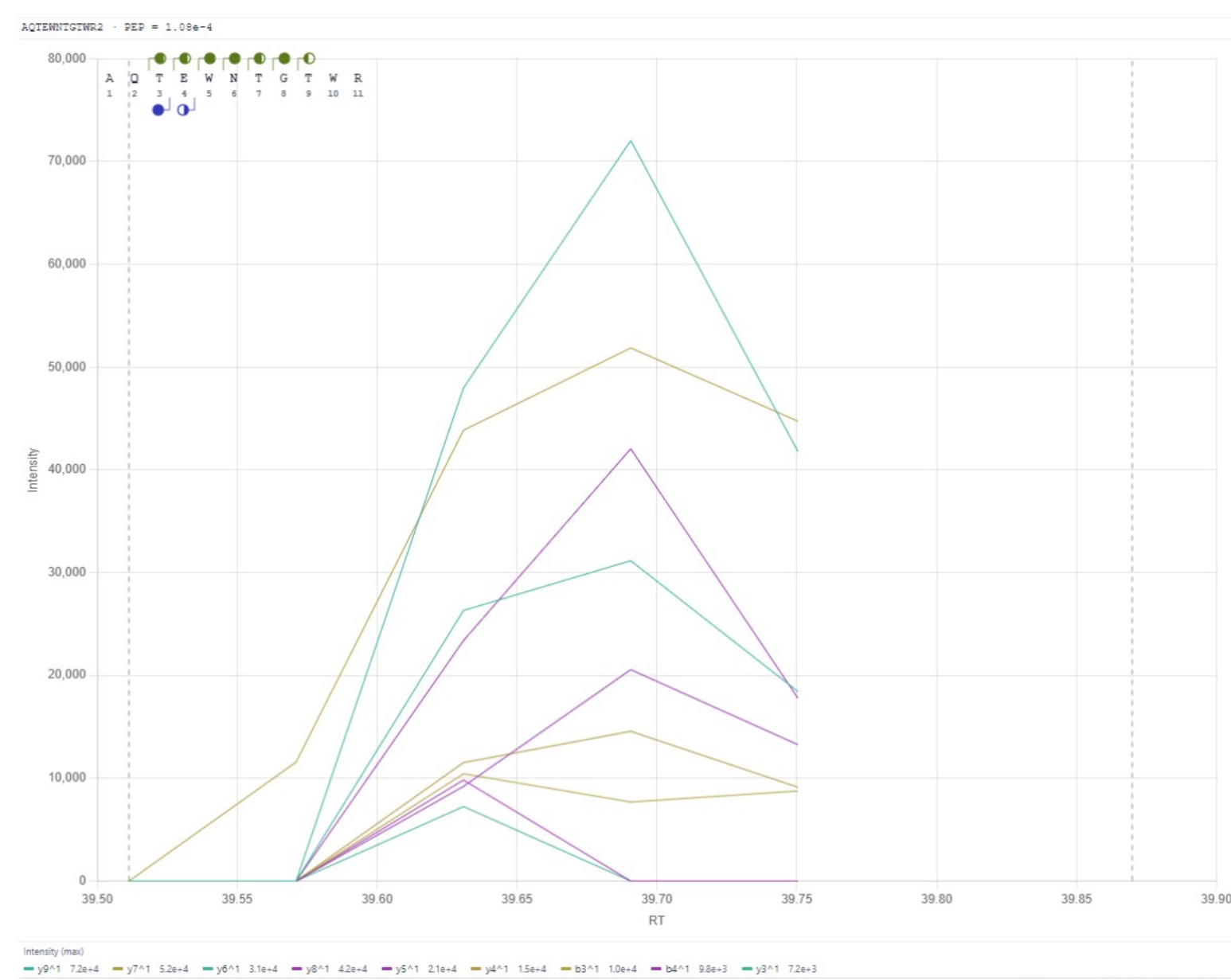

SDCBP

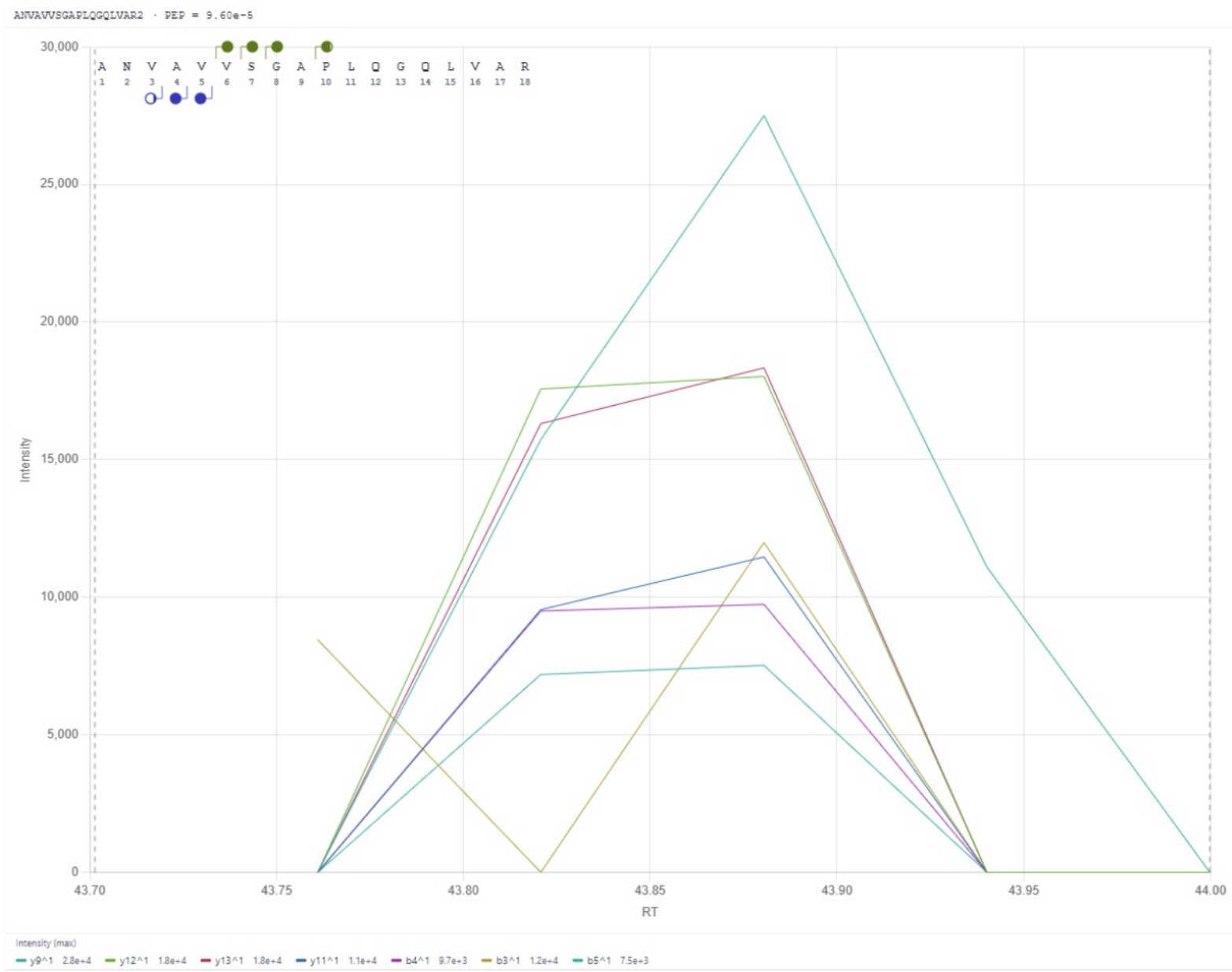

SDF2

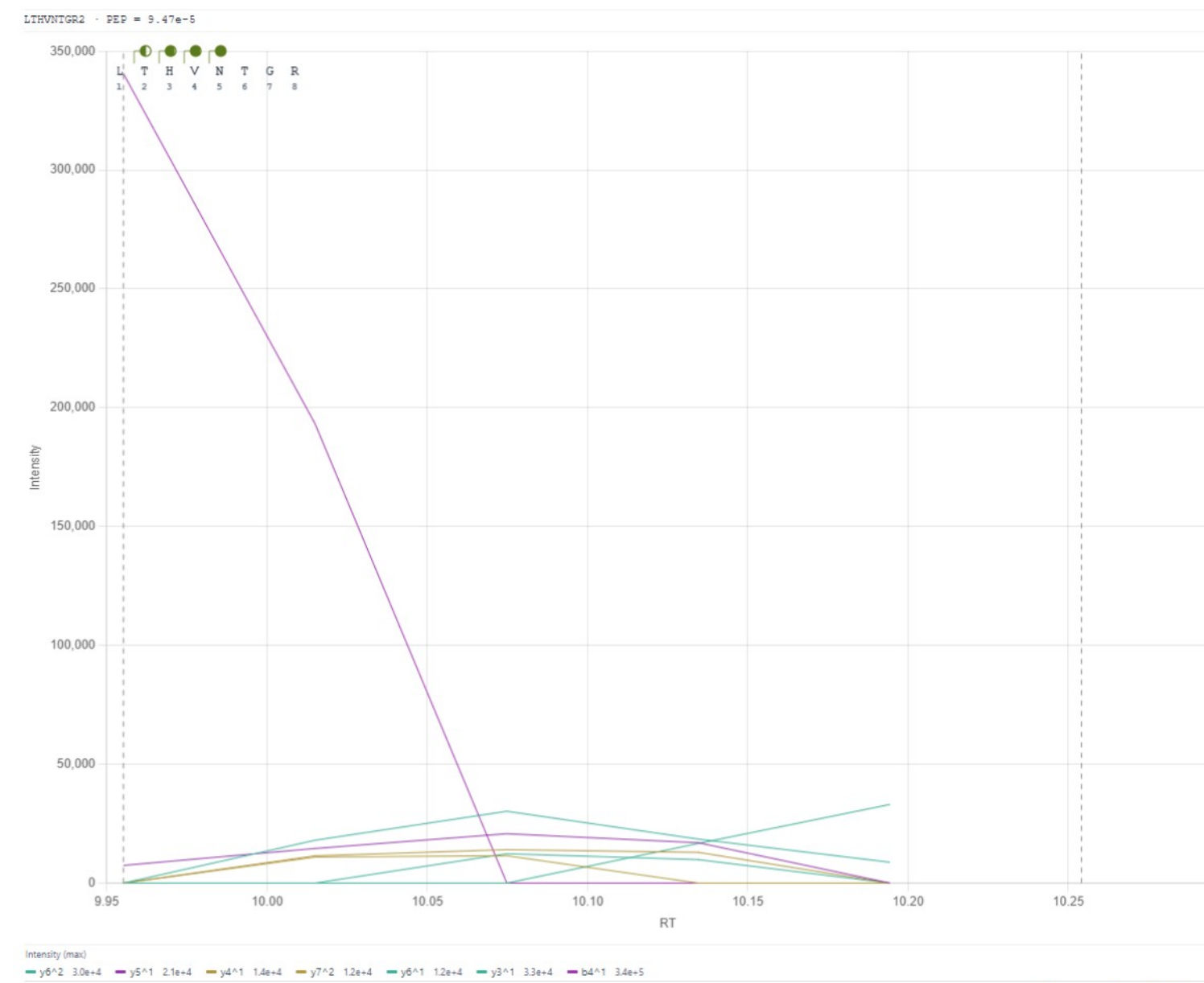

SDHC

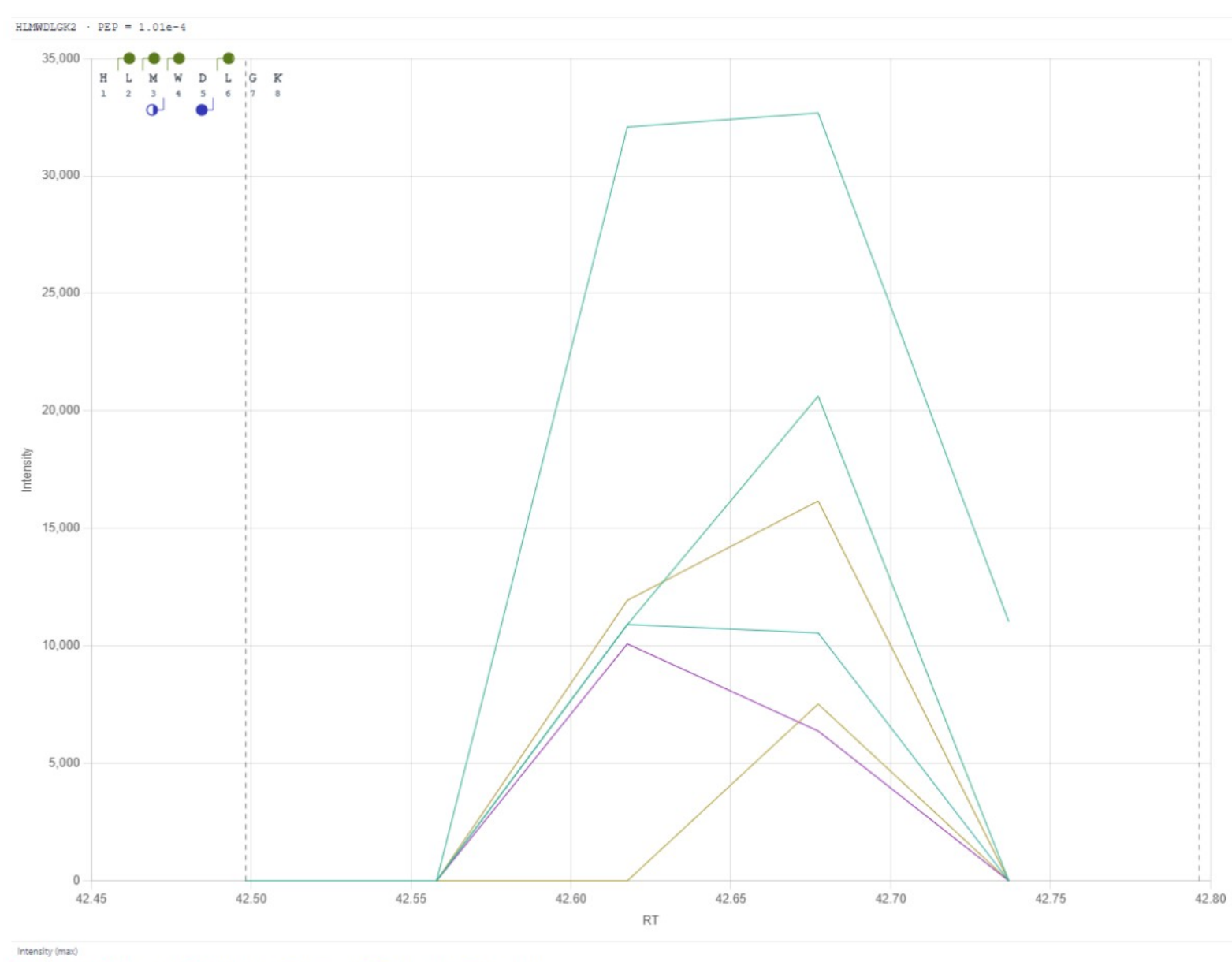

SEC23A

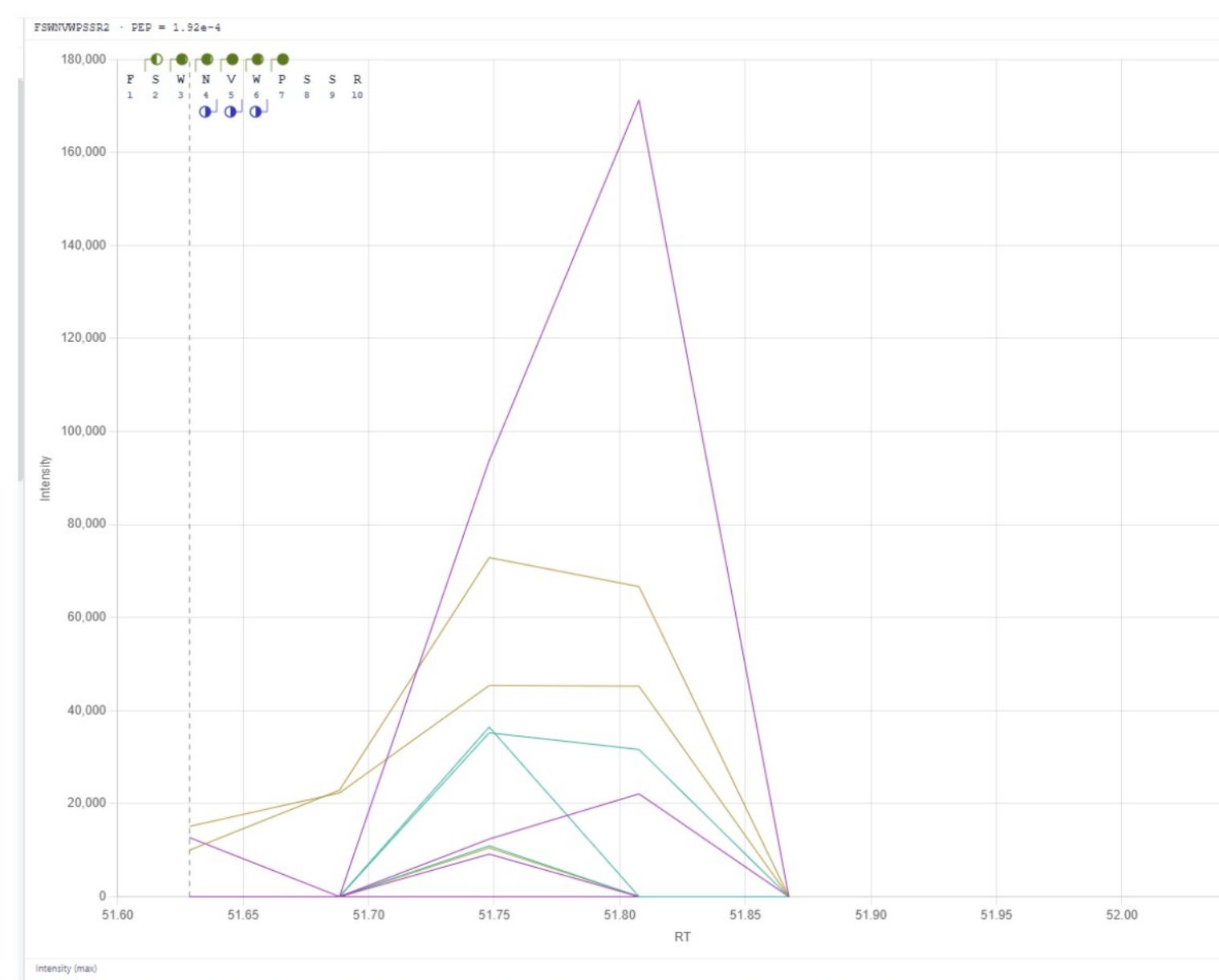

SEC24B

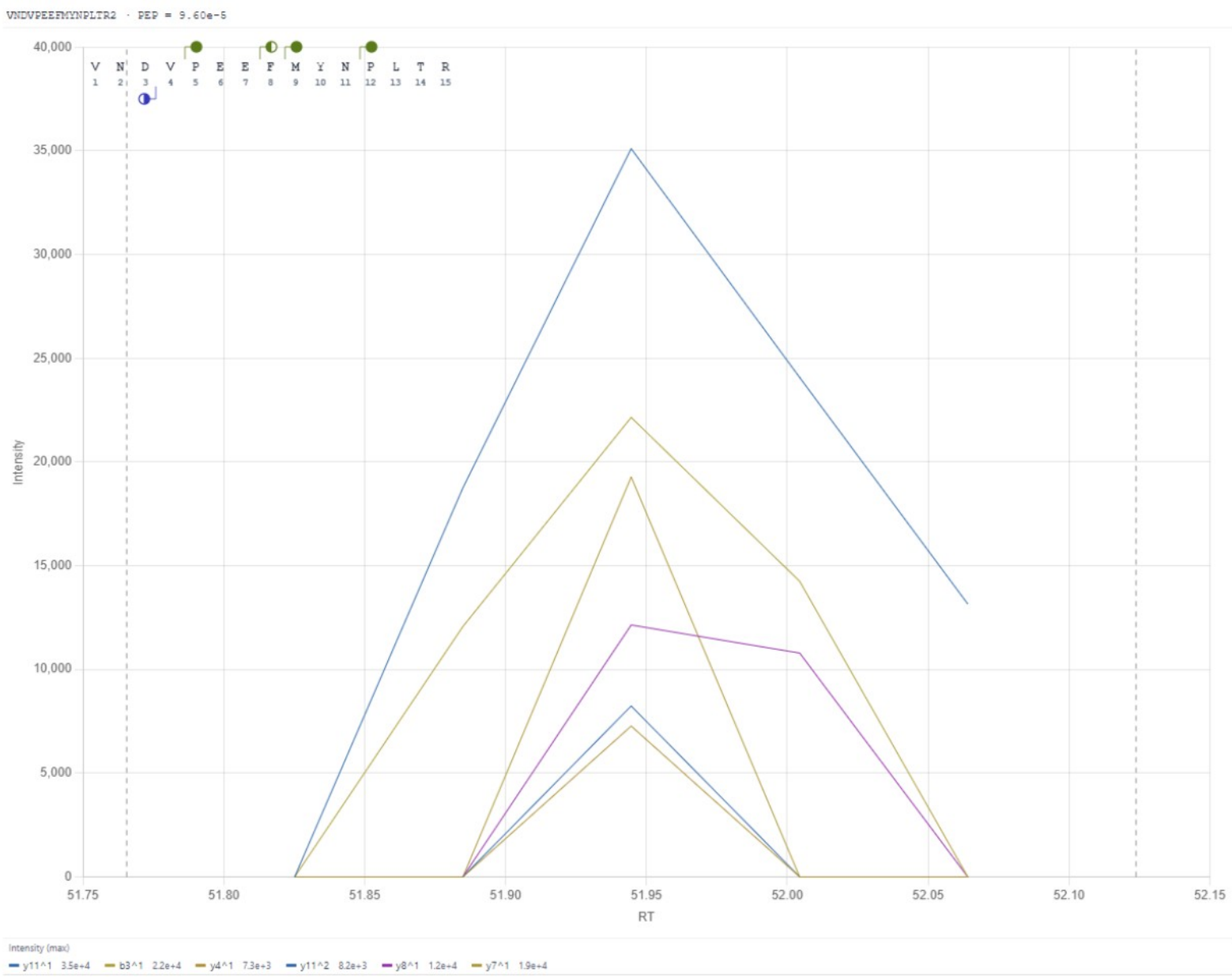

SEC24D

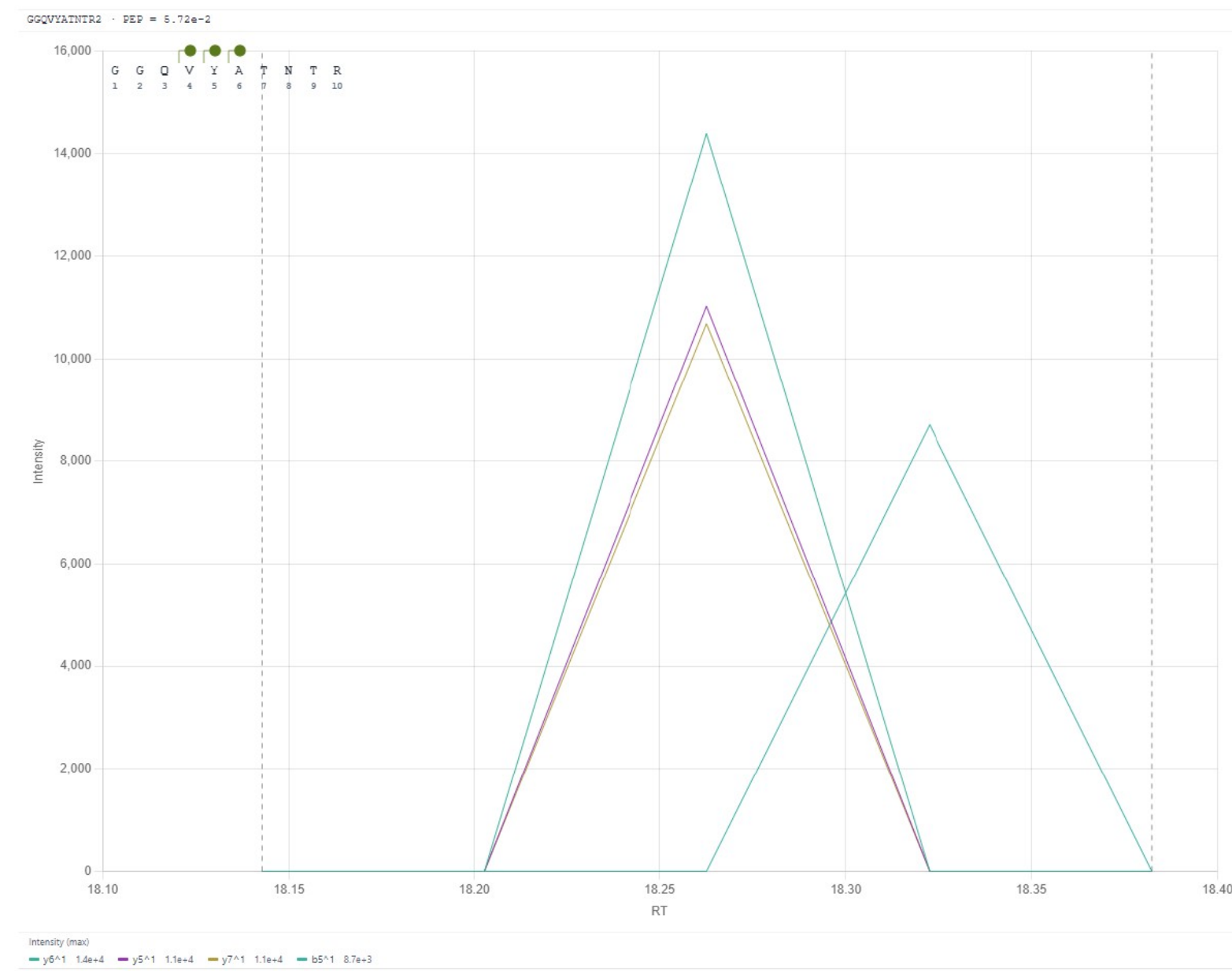

SEC65

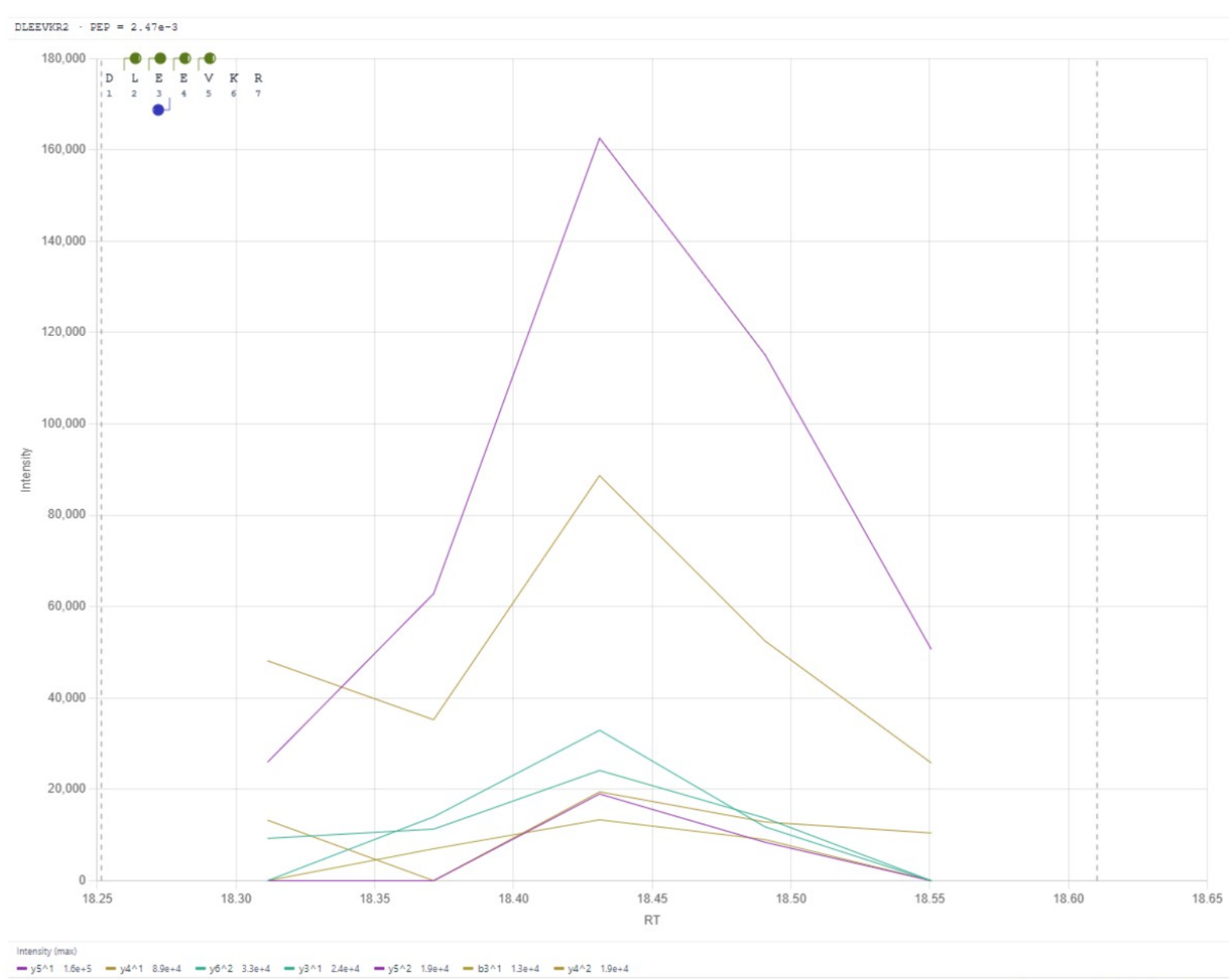

SEPTIN7

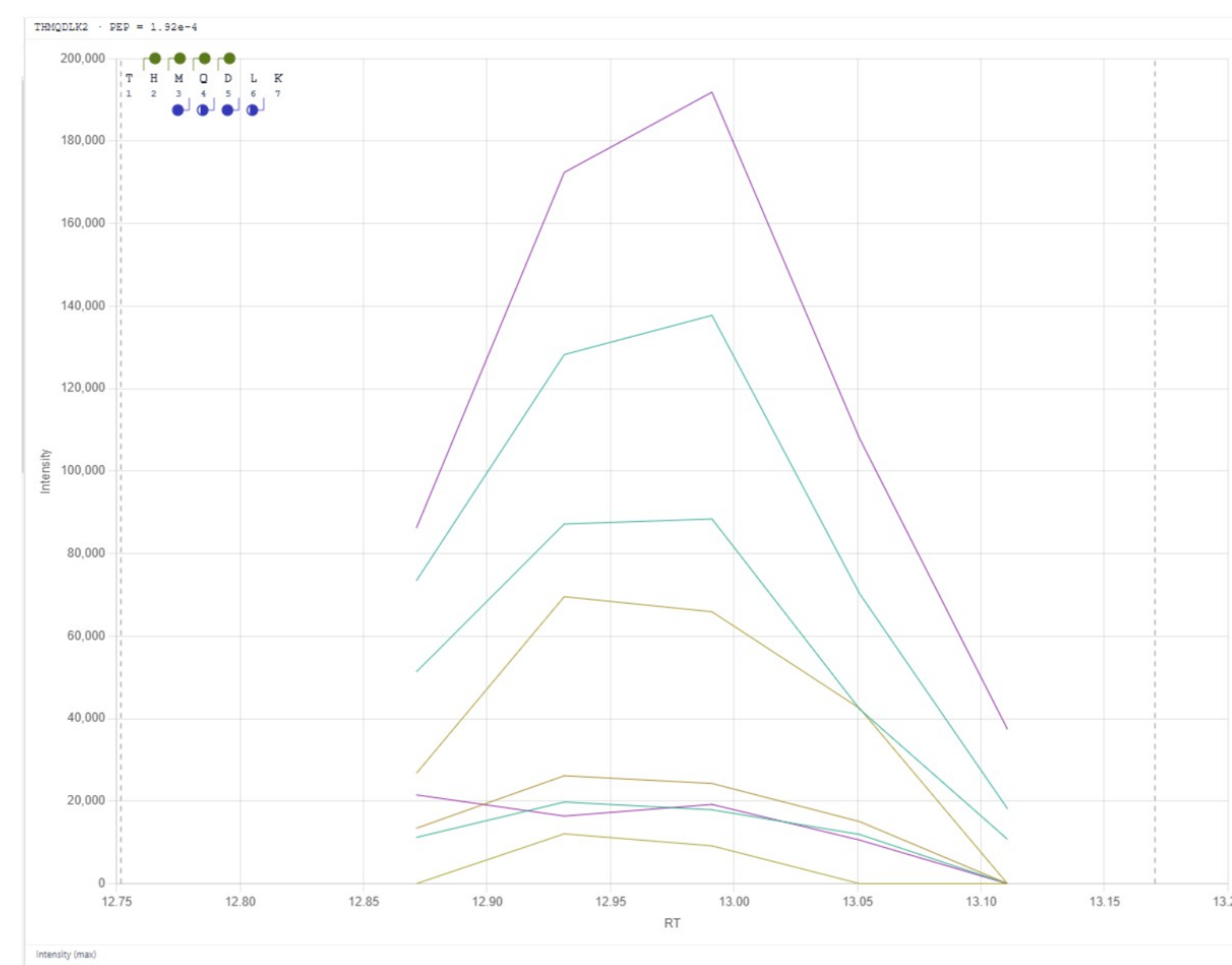

SERPINB1

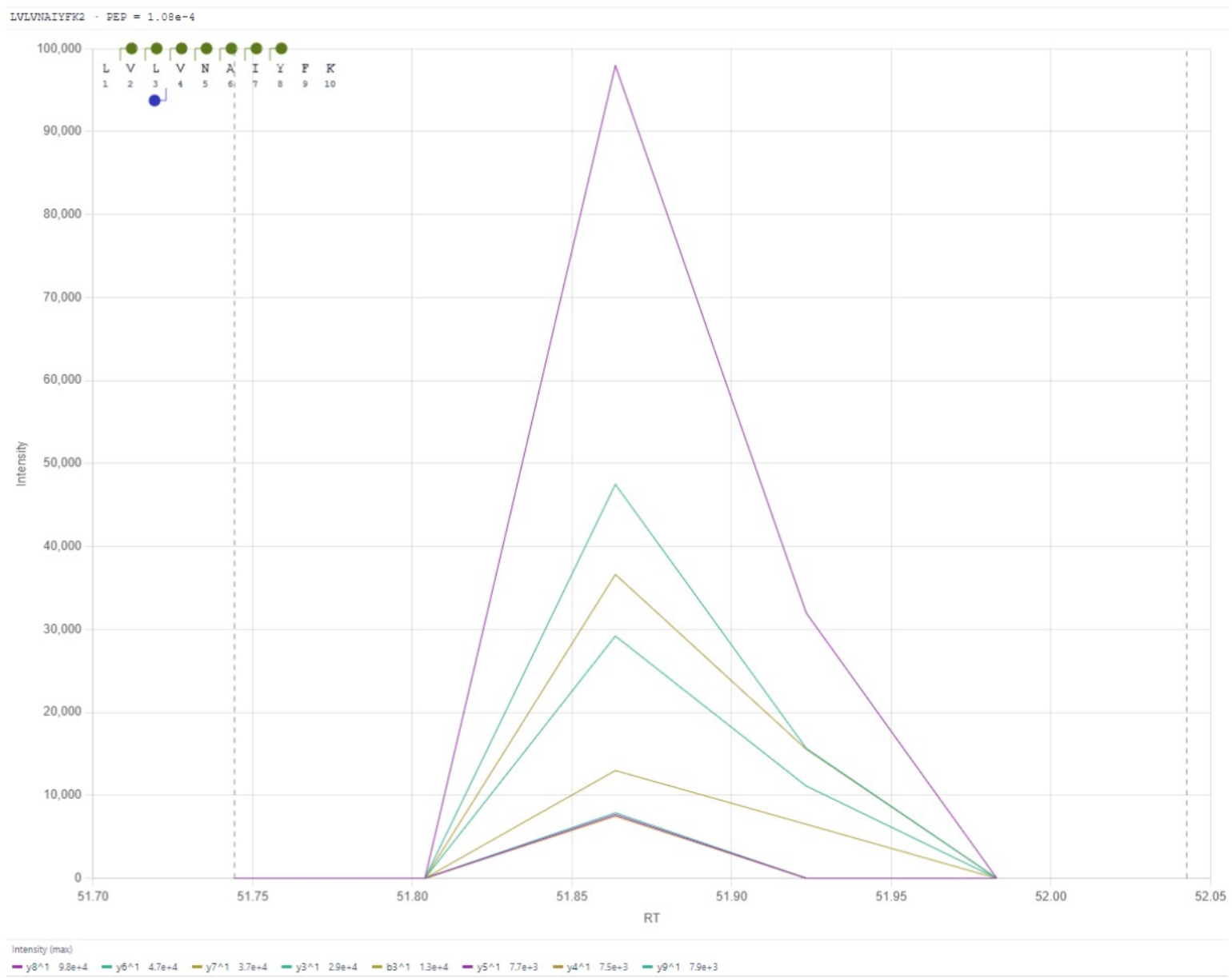

SFT2D2

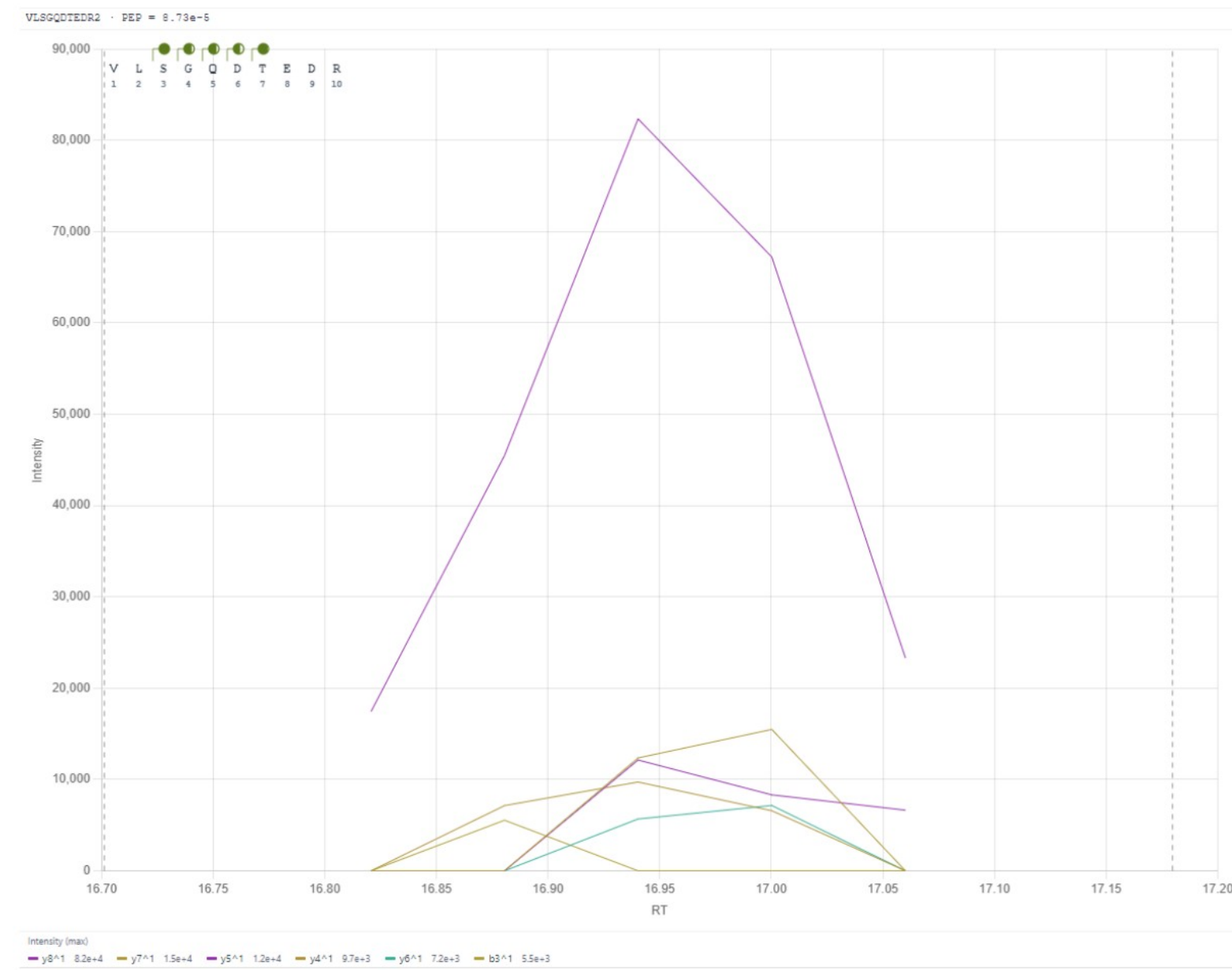

SH3GLB2

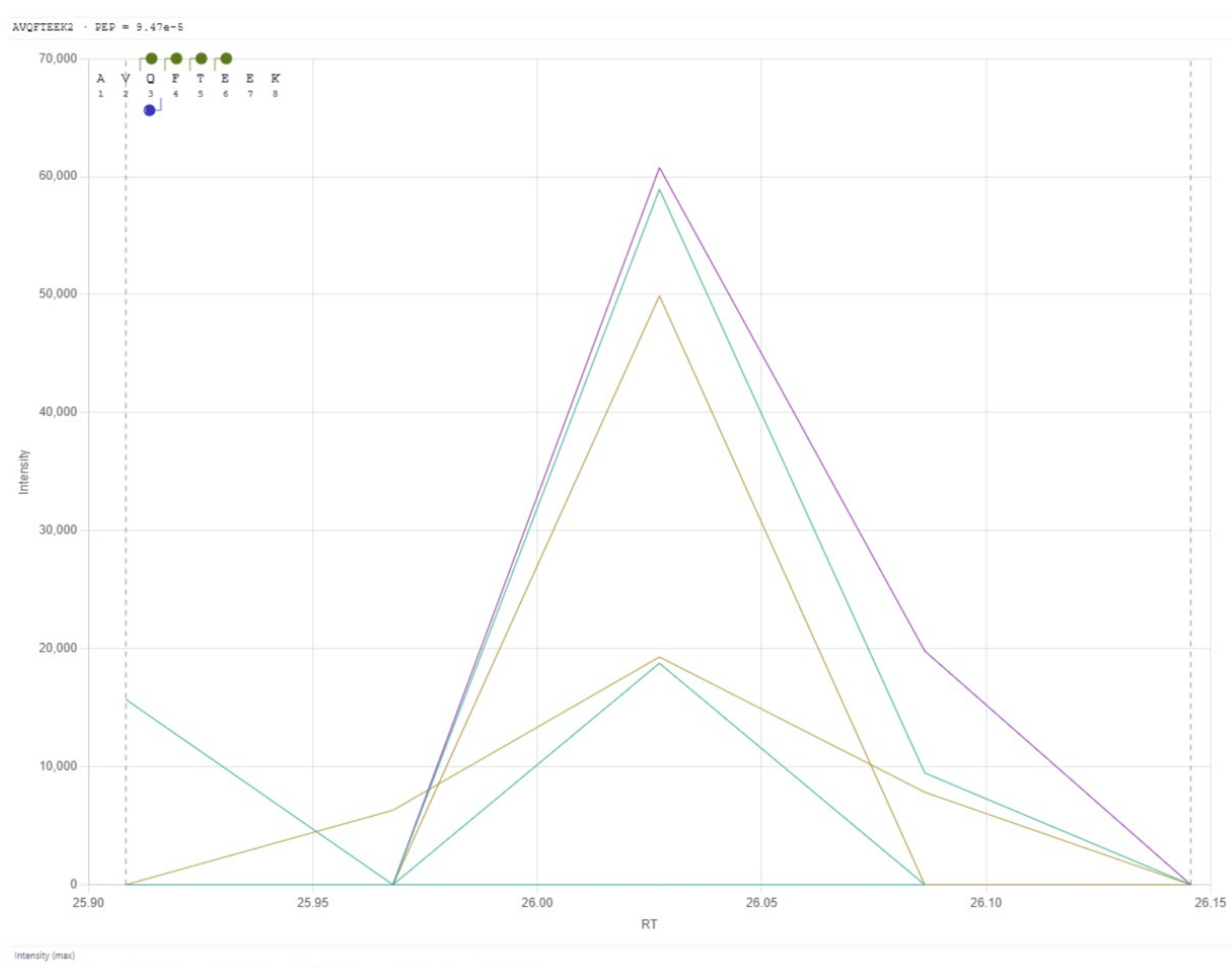

si:dkeyp-13a3.3

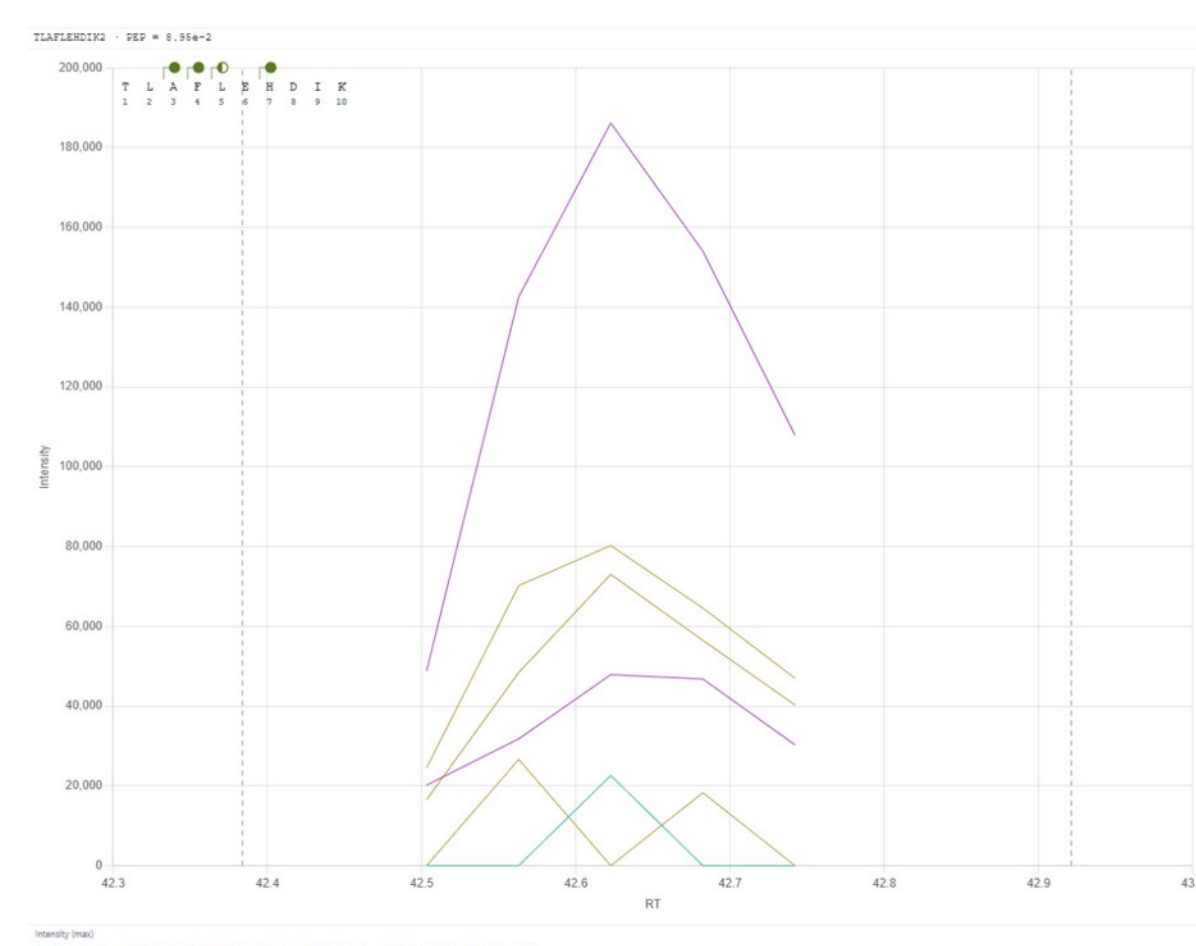

SKIV2L

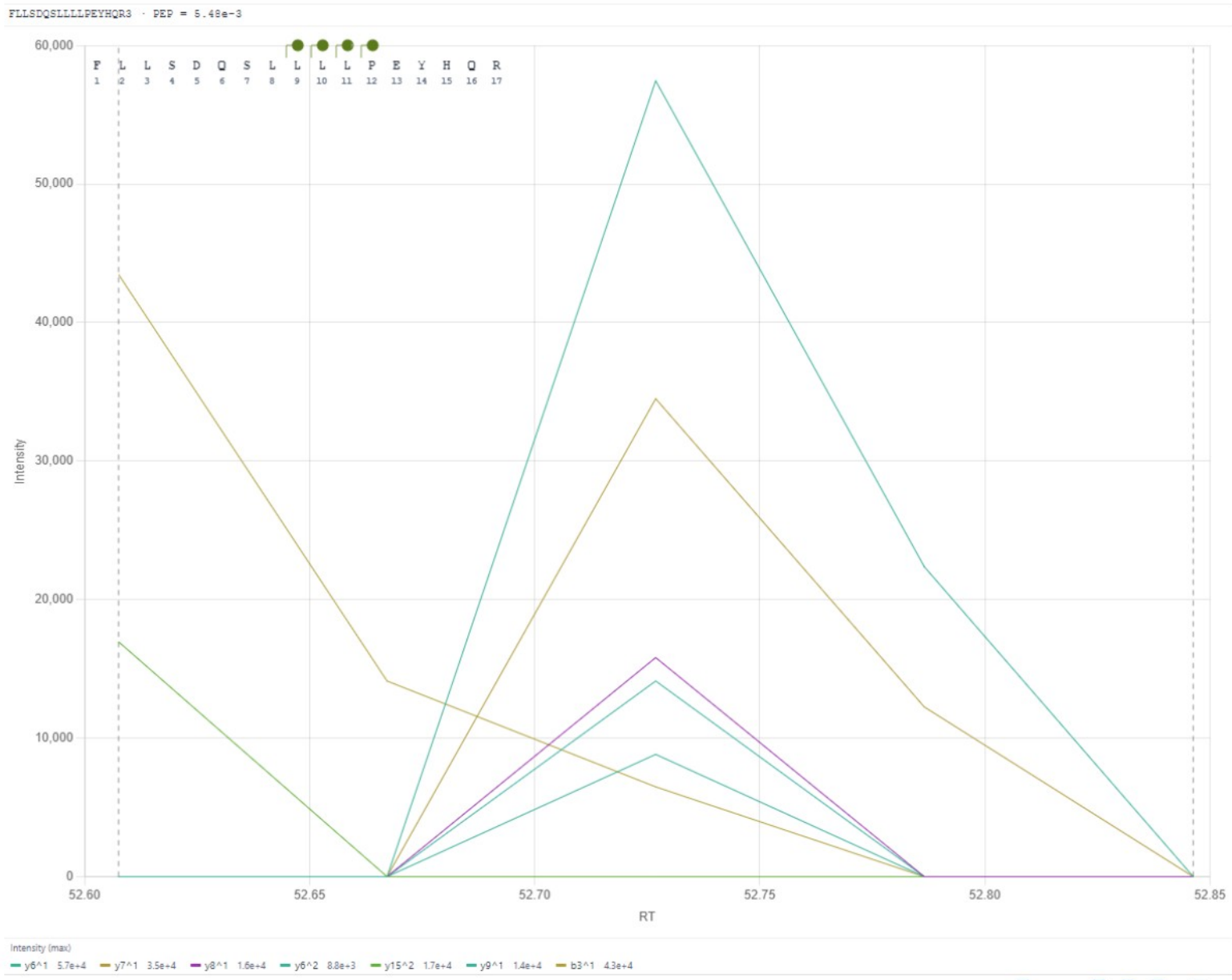

SLC37A4

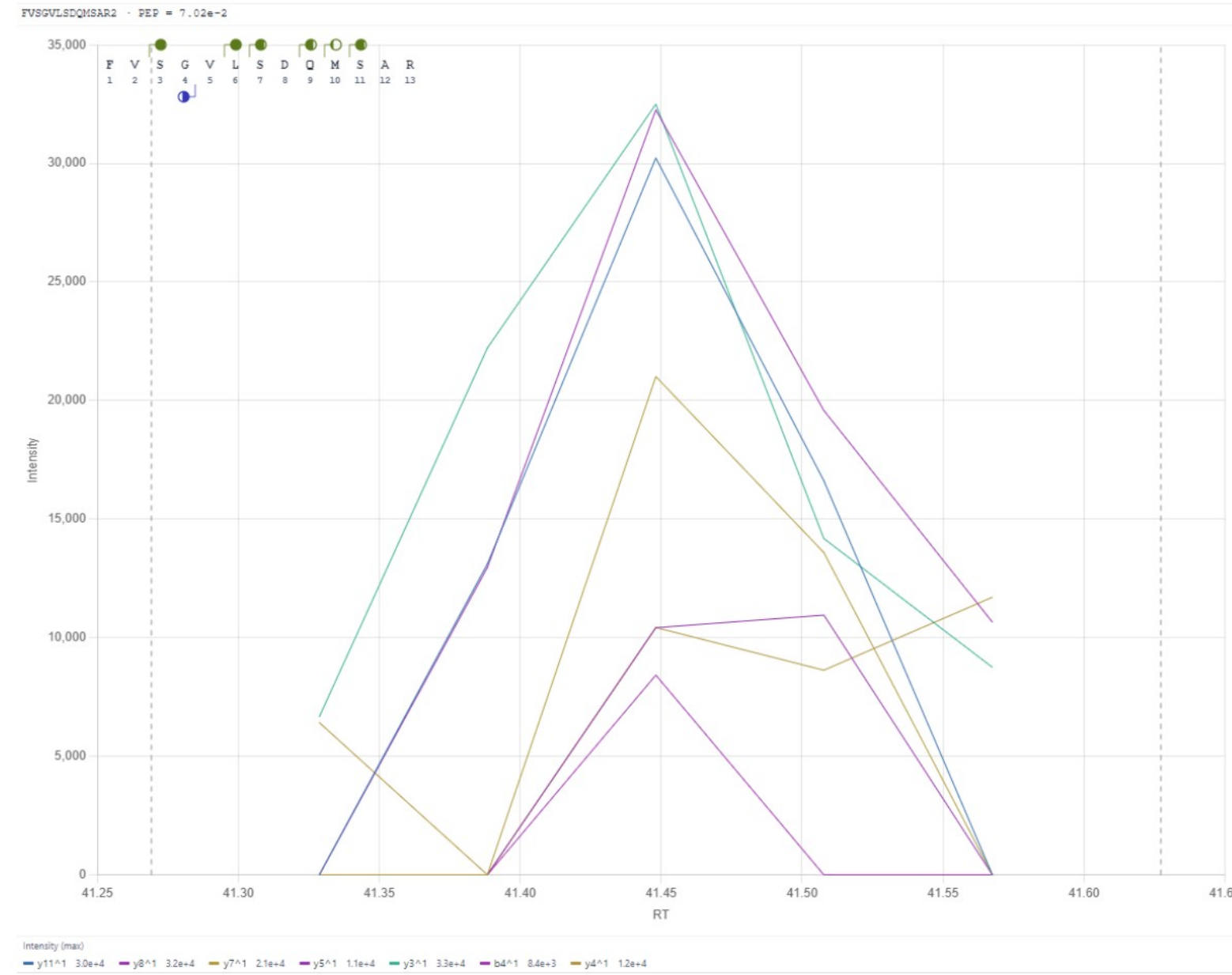

SLC6A4

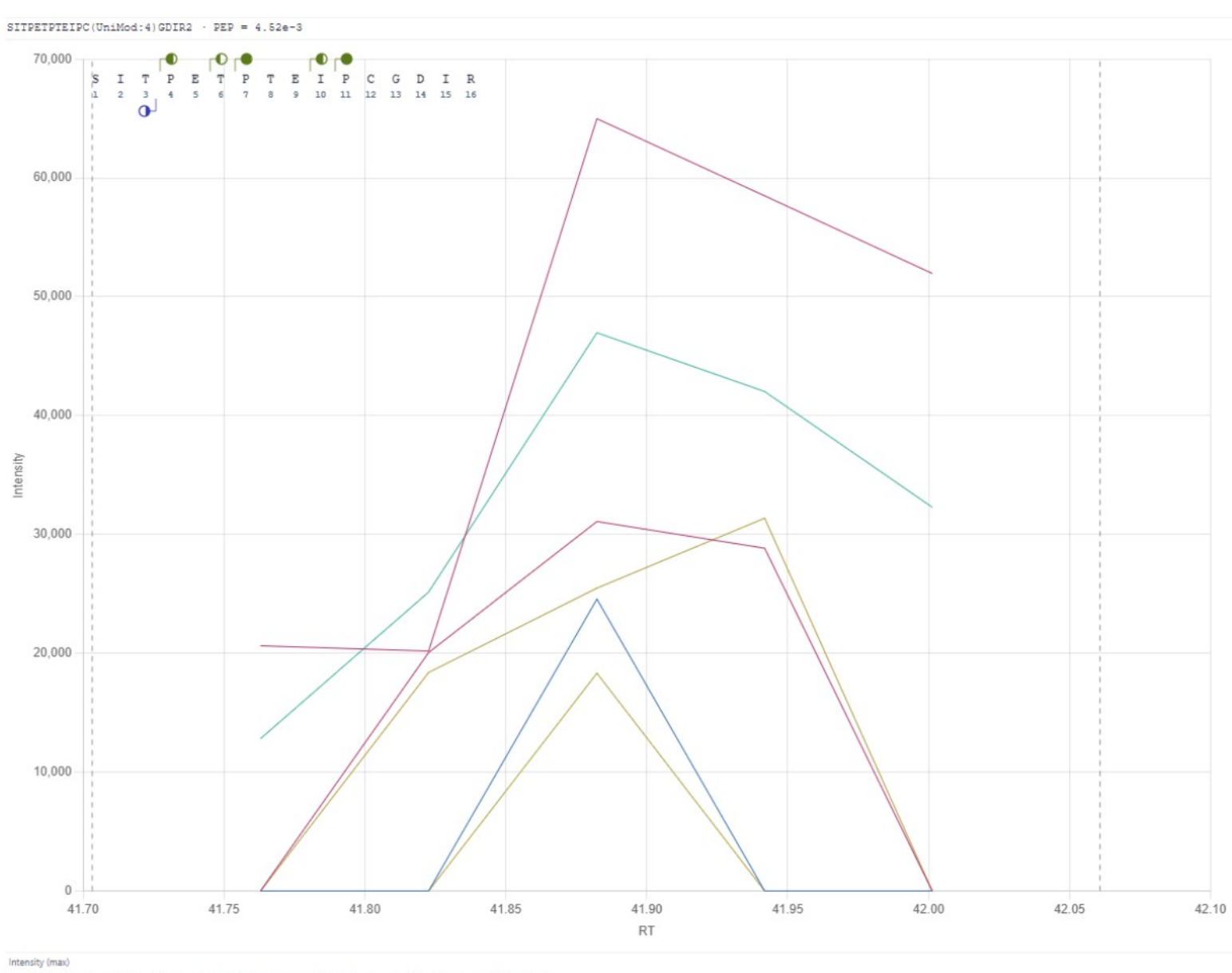

SPCS1

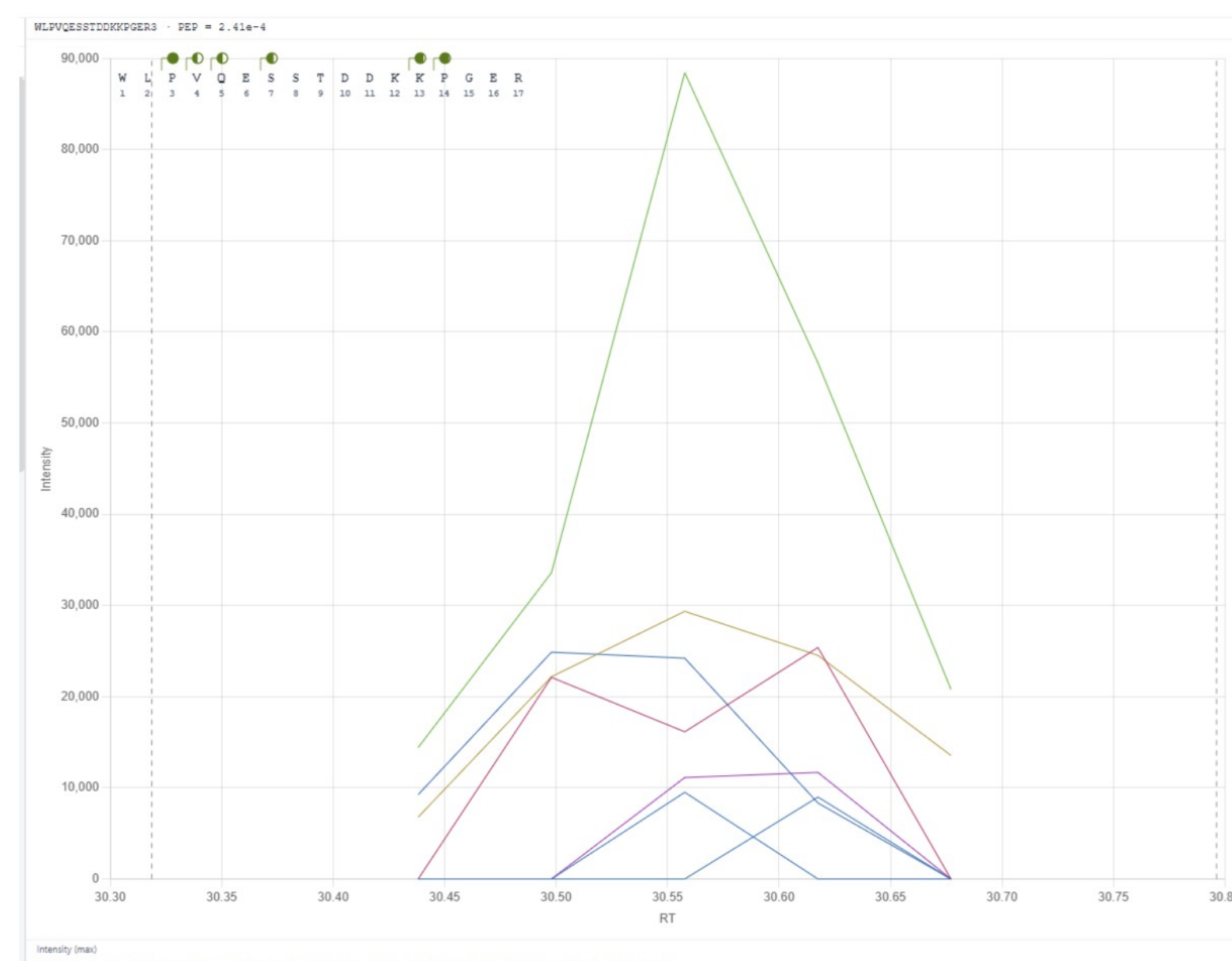

SPI1

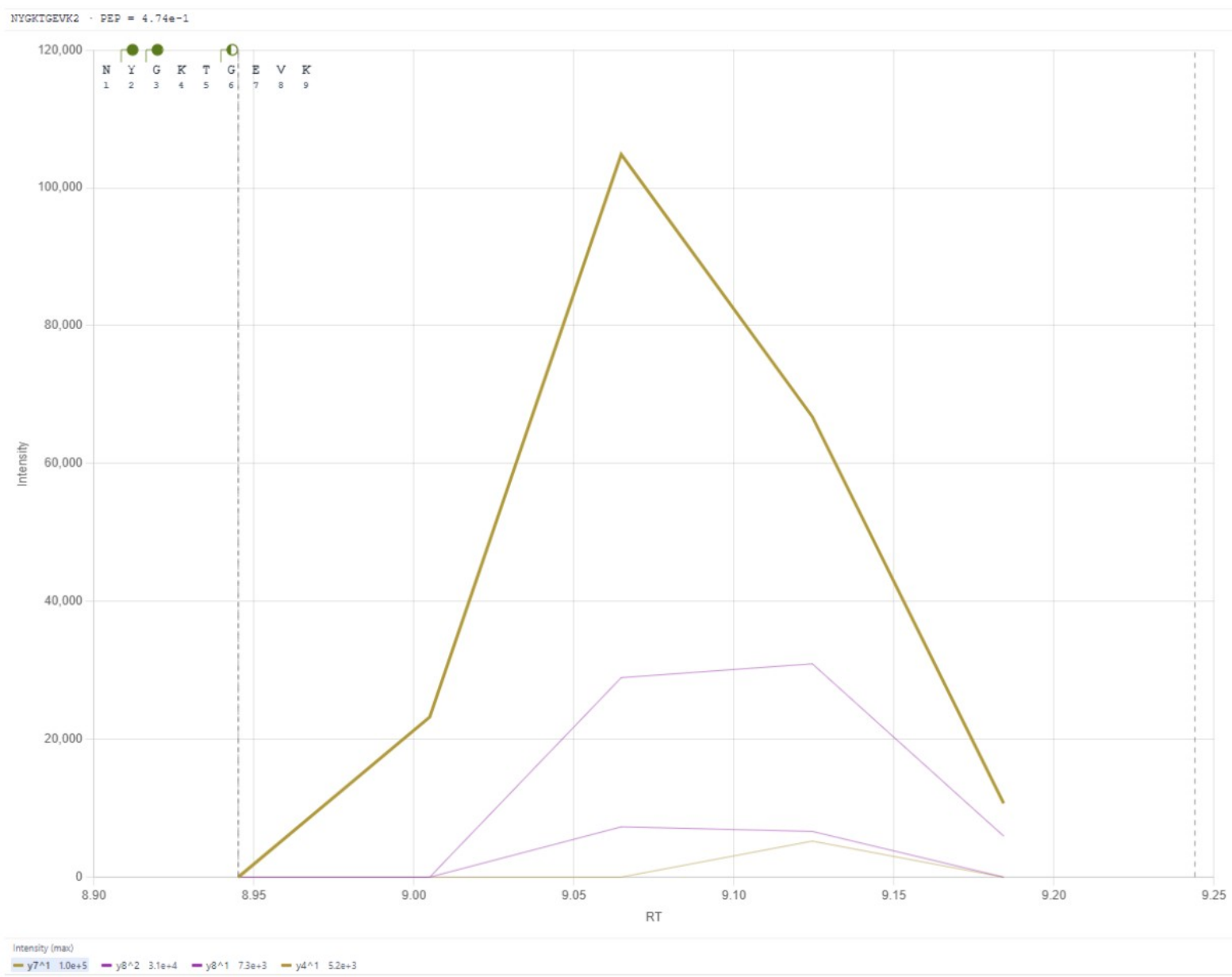

SSR1

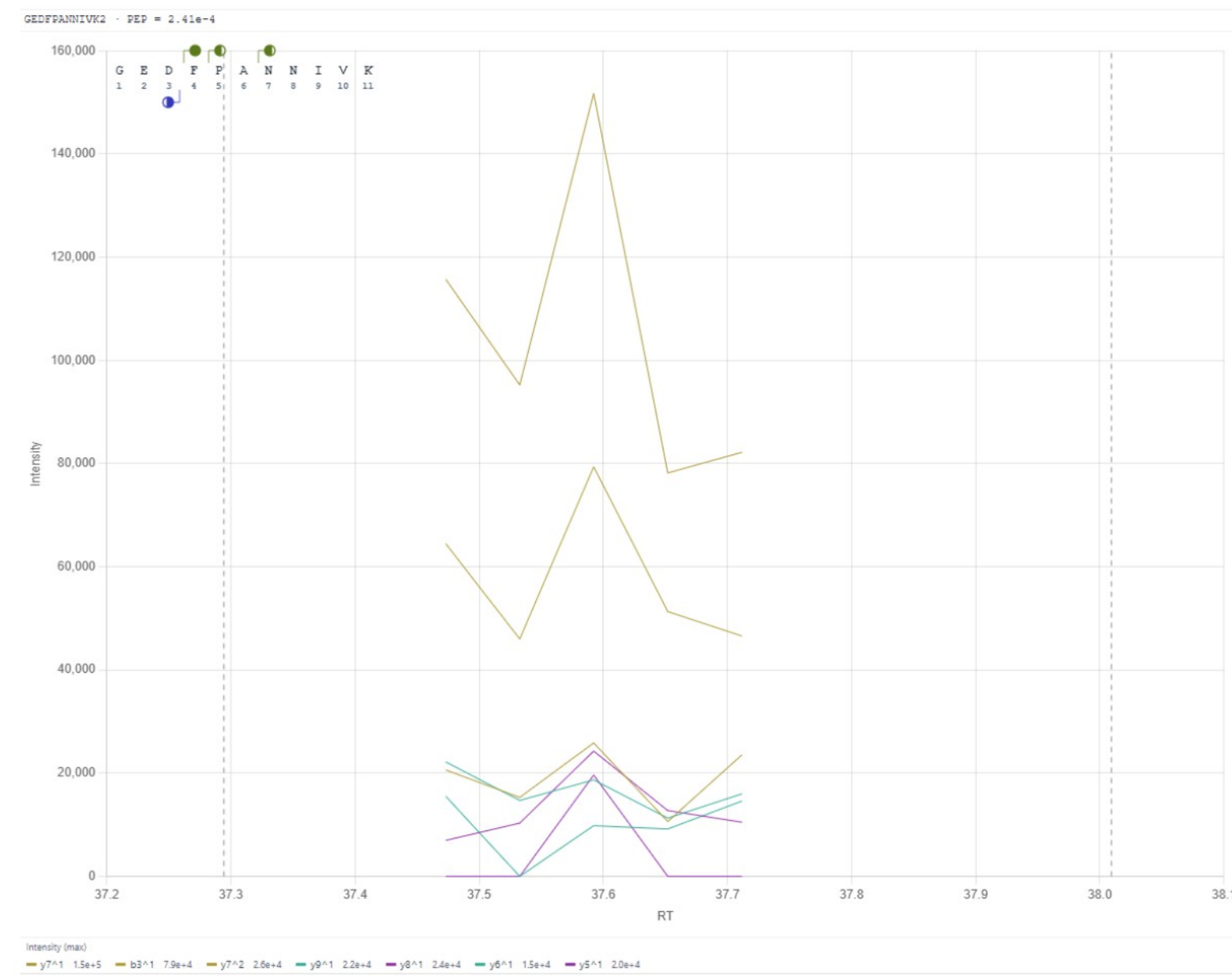

STAM

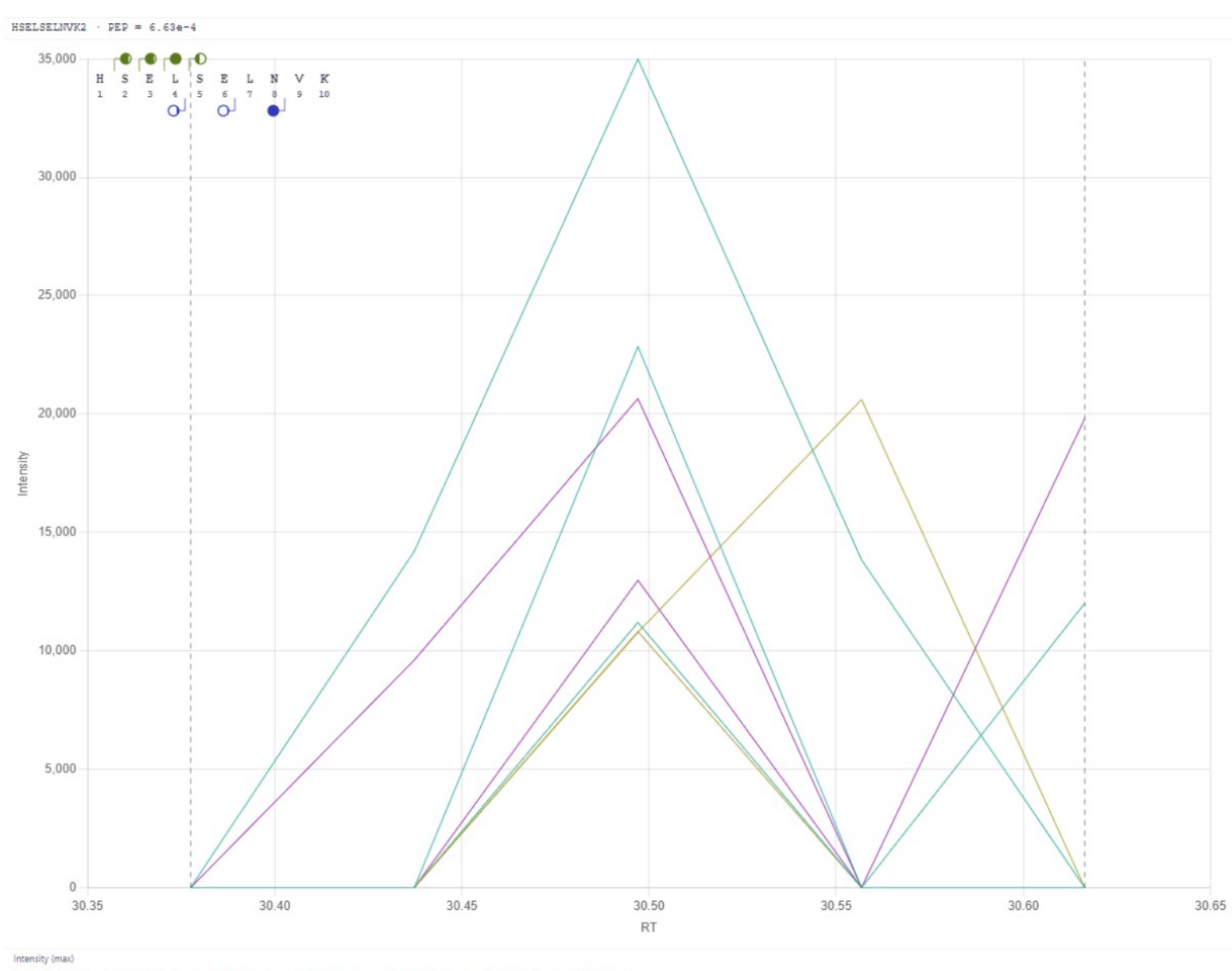

STBD1

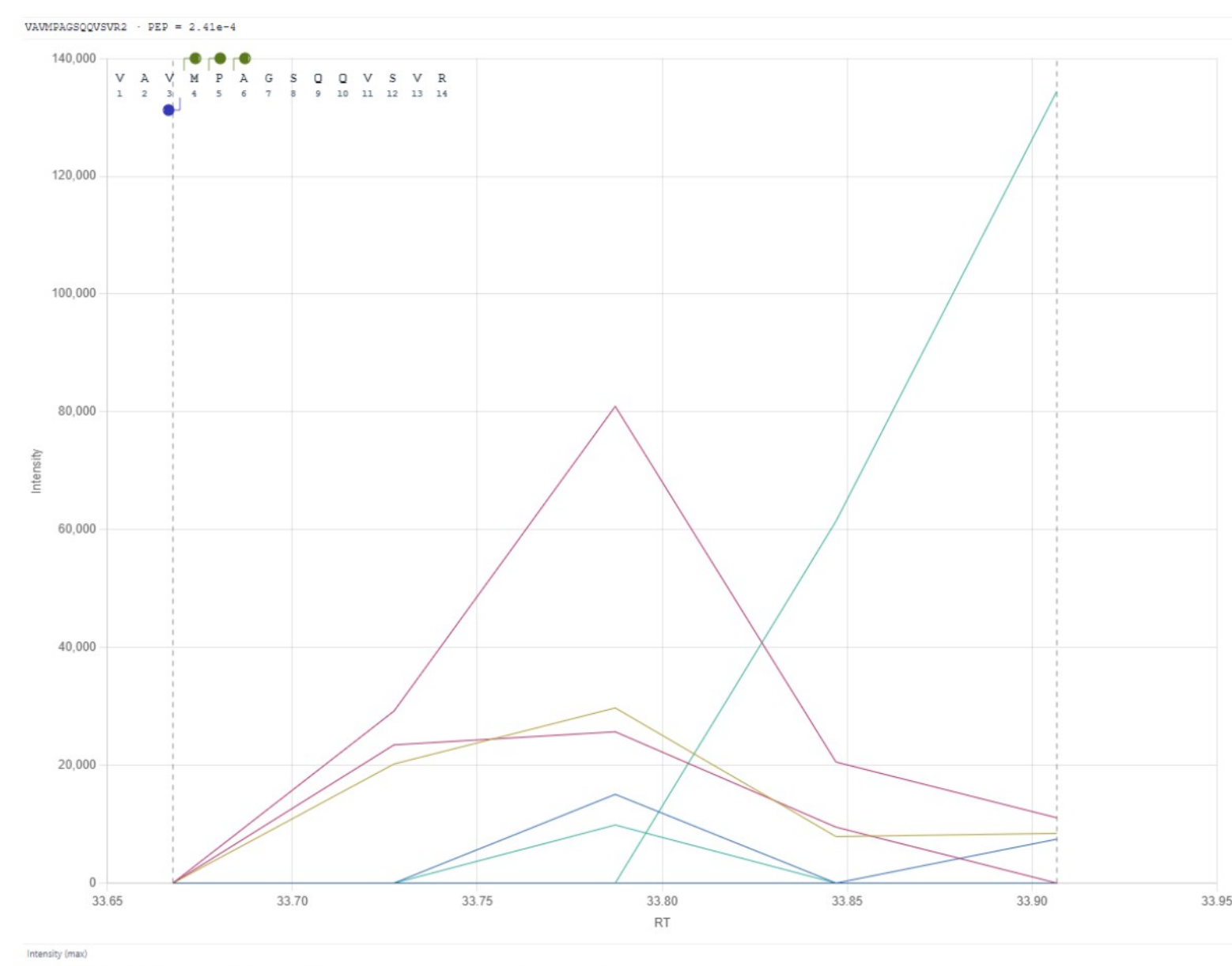

STOM

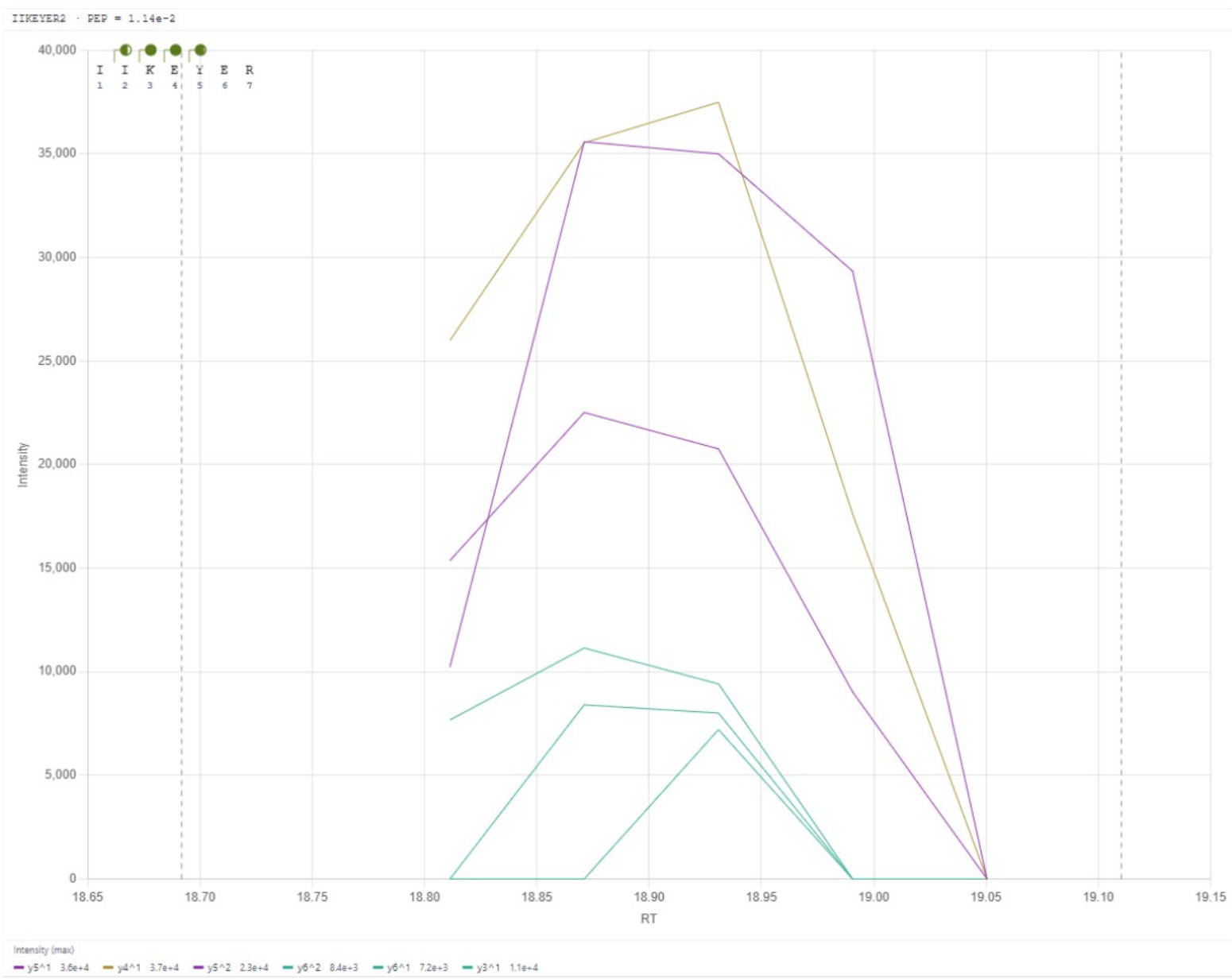

STRN4

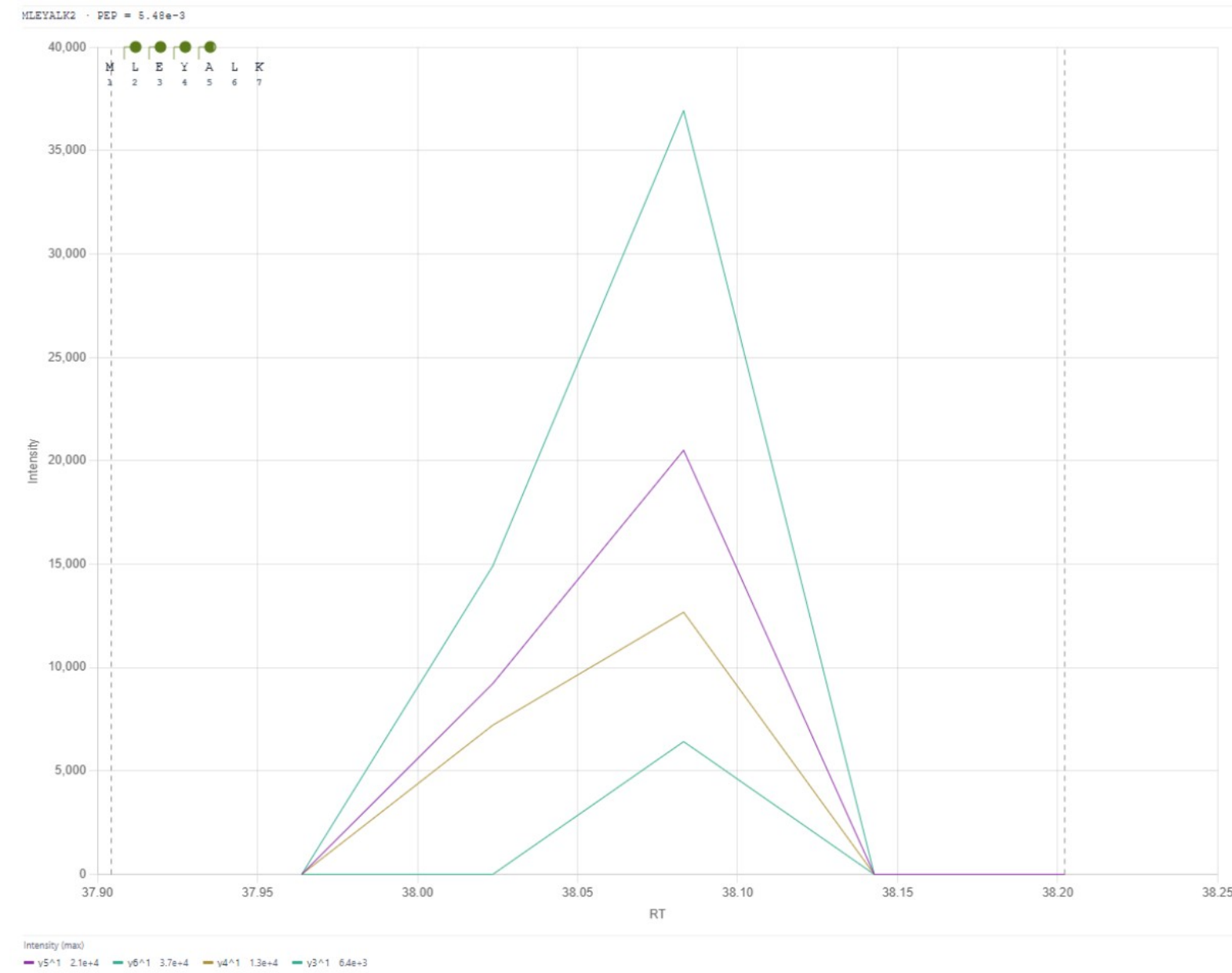

STXBP2

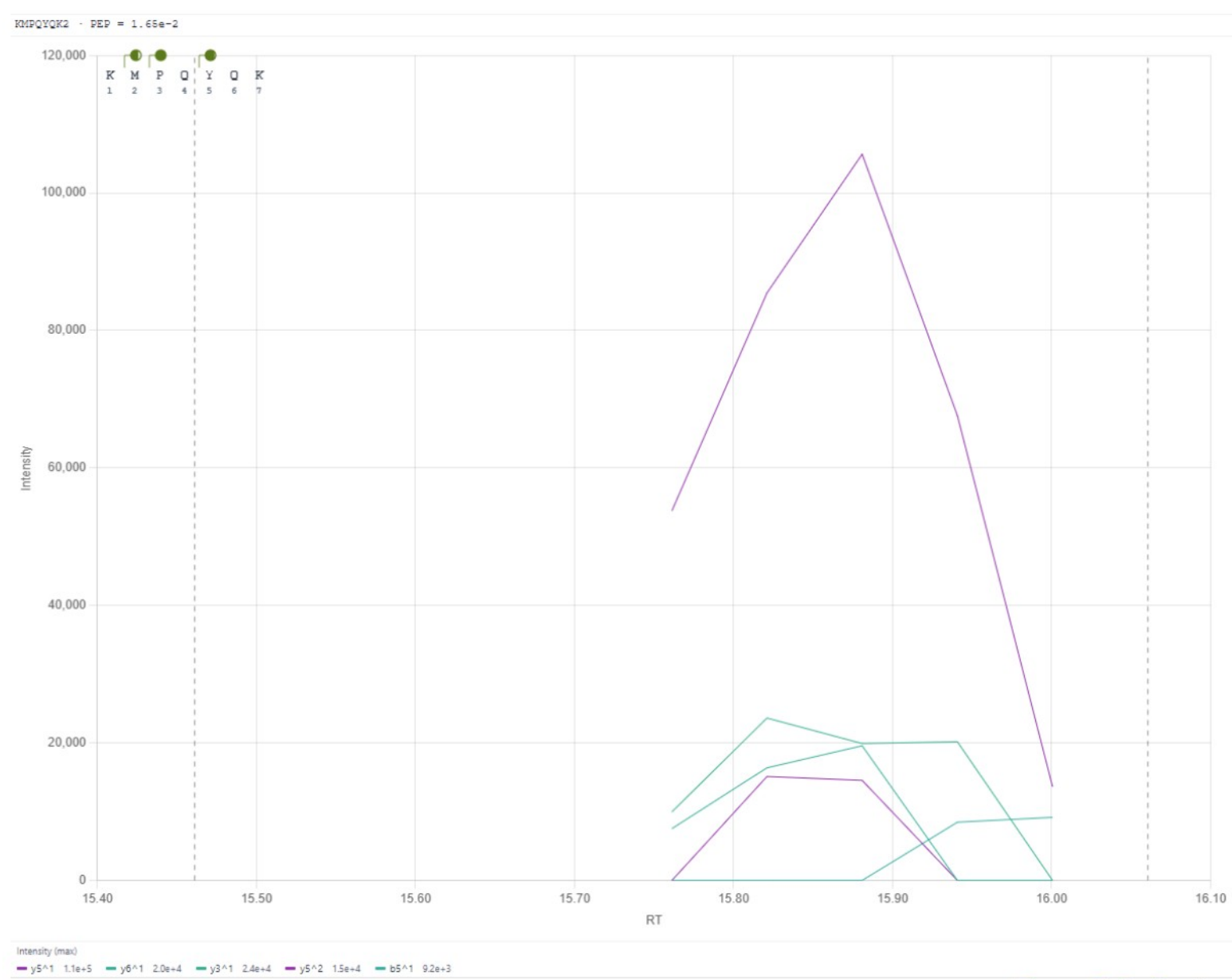

SUMF2

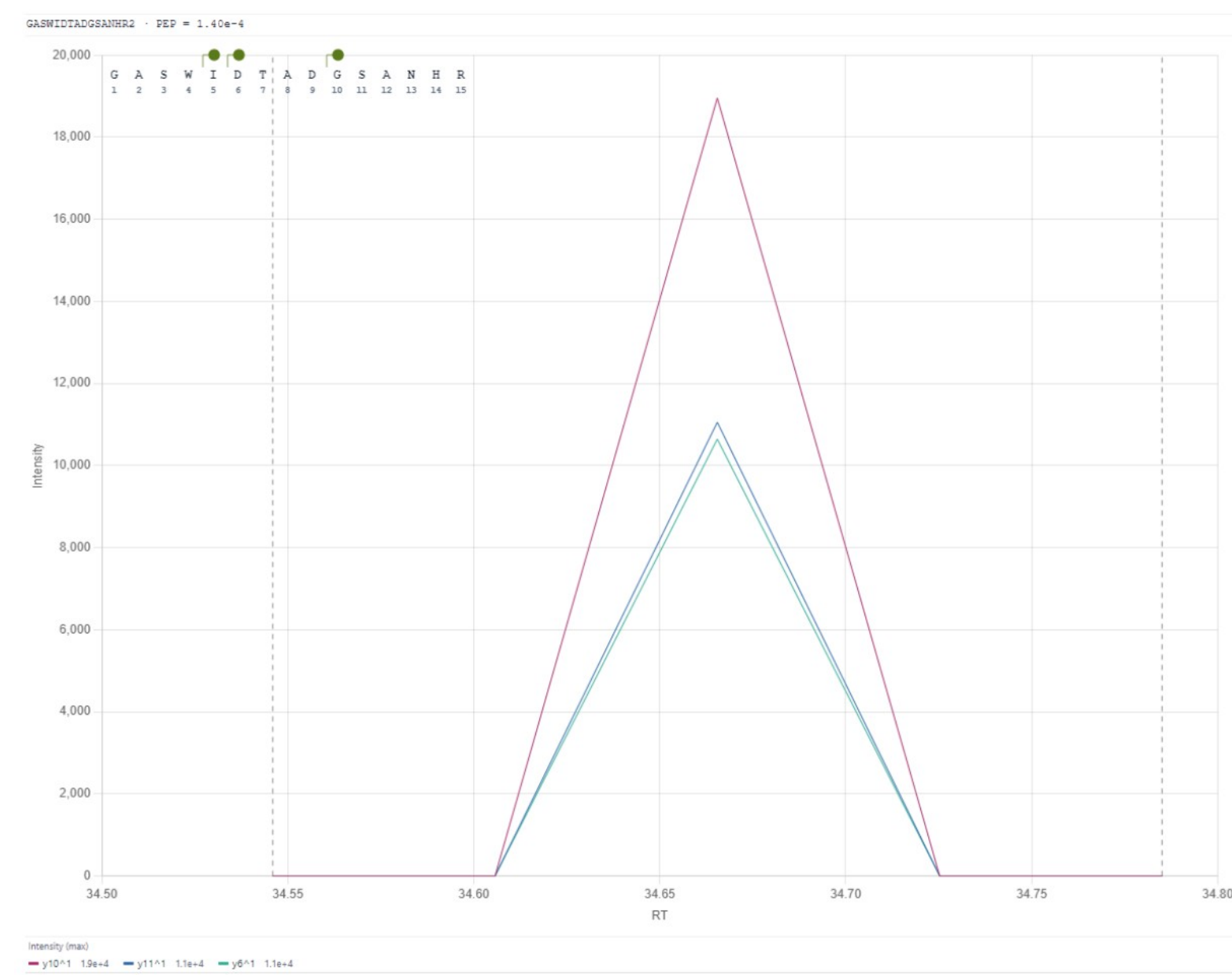

SYAP1

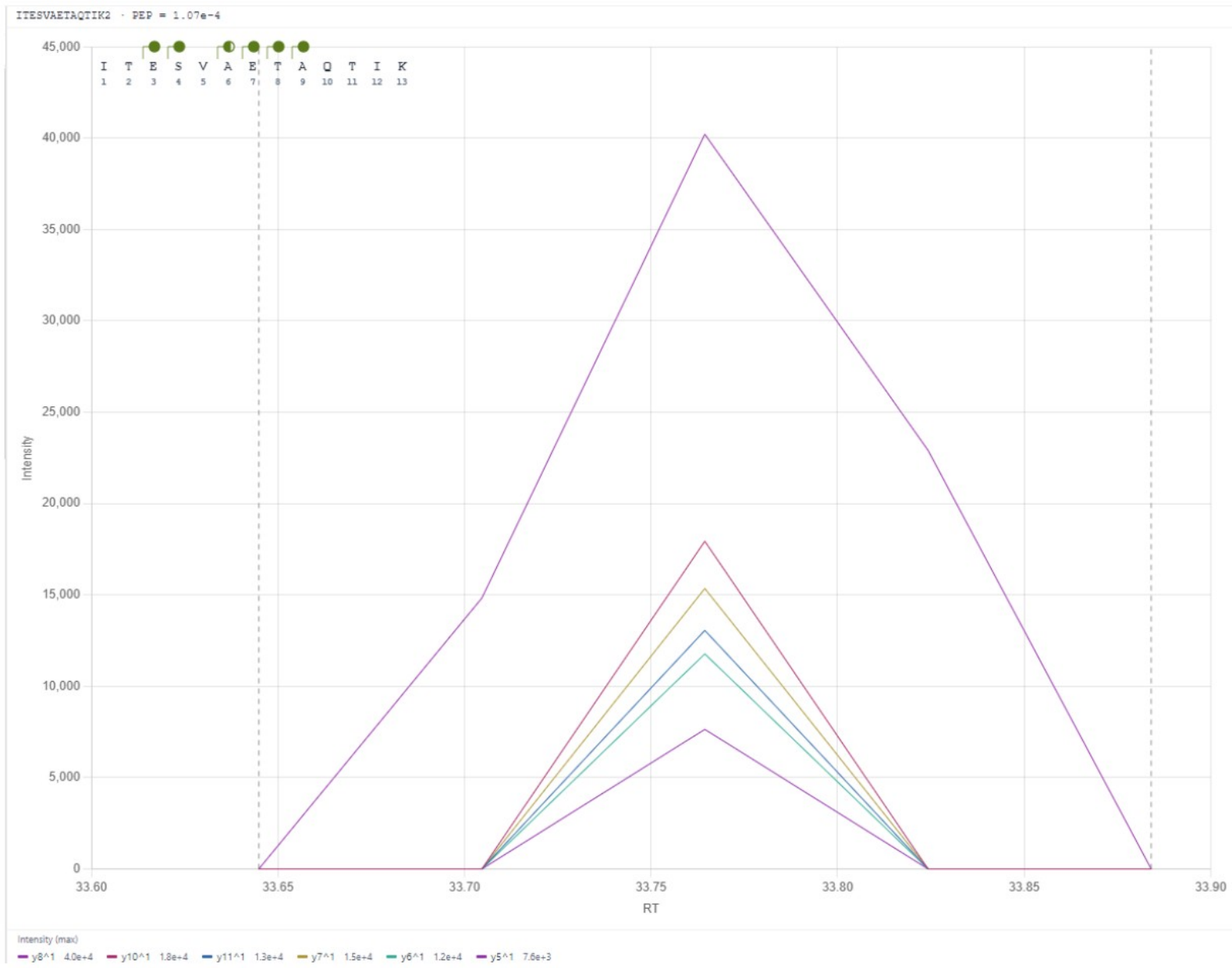

SYNE2

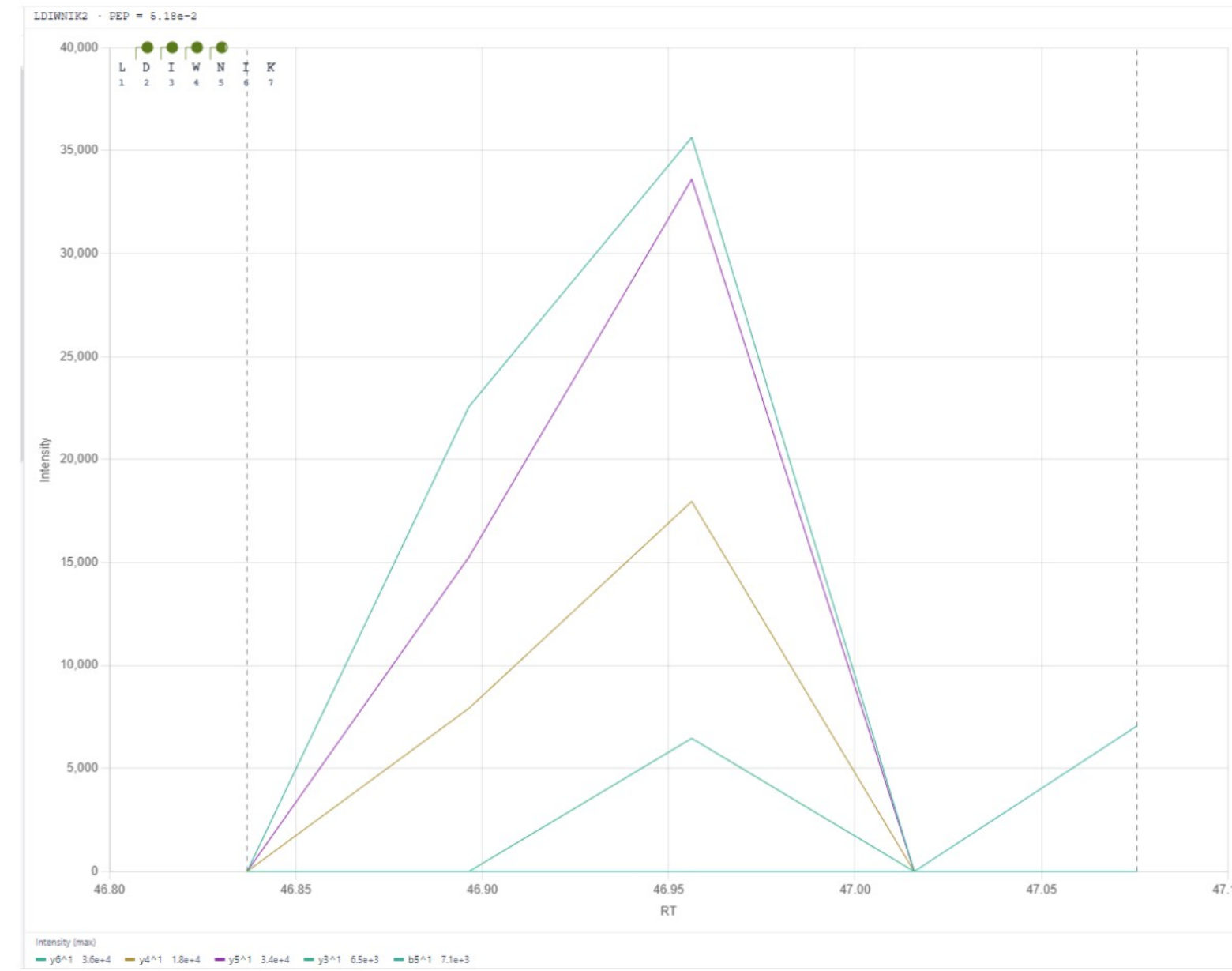

SYNGR2

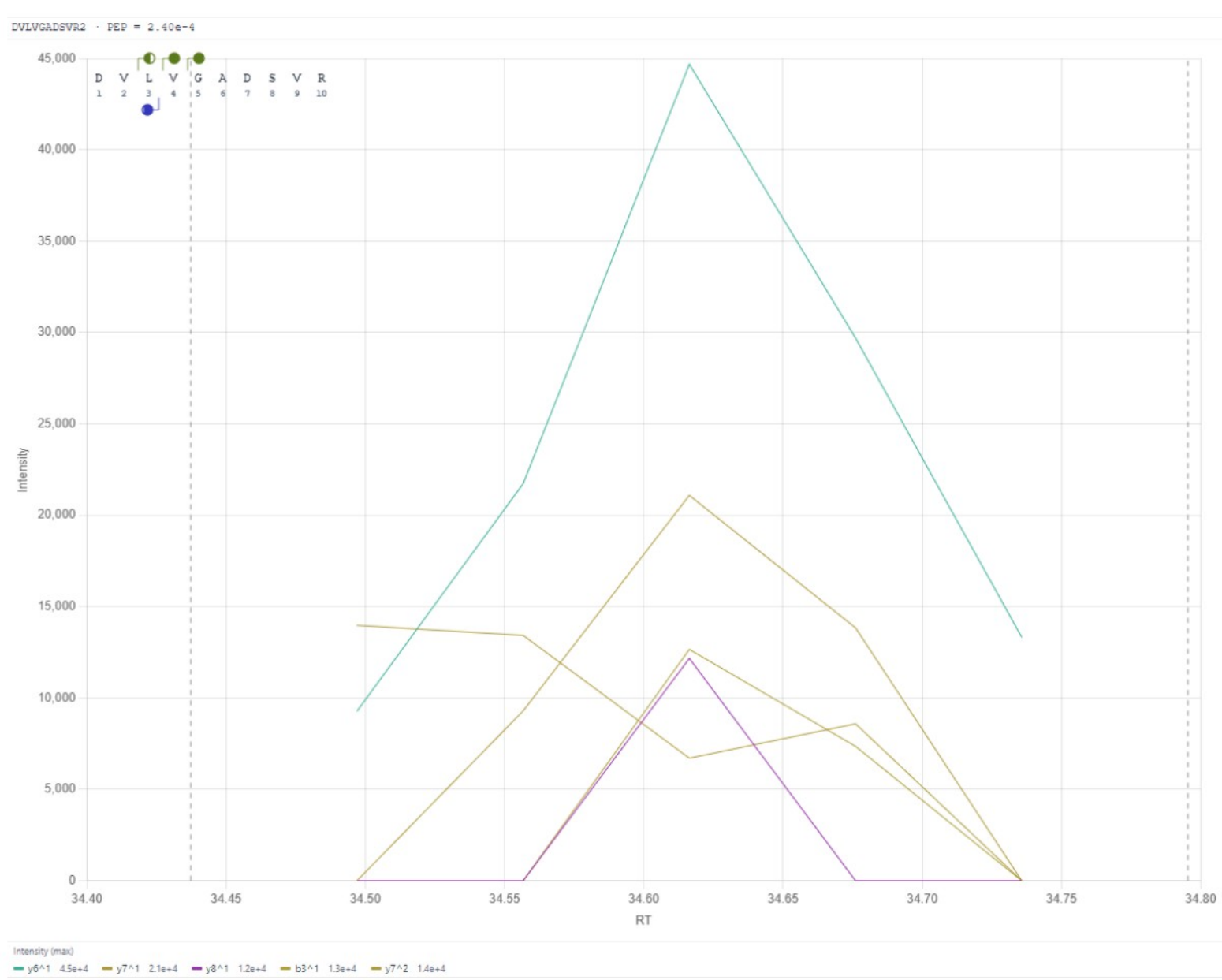

Tbx20

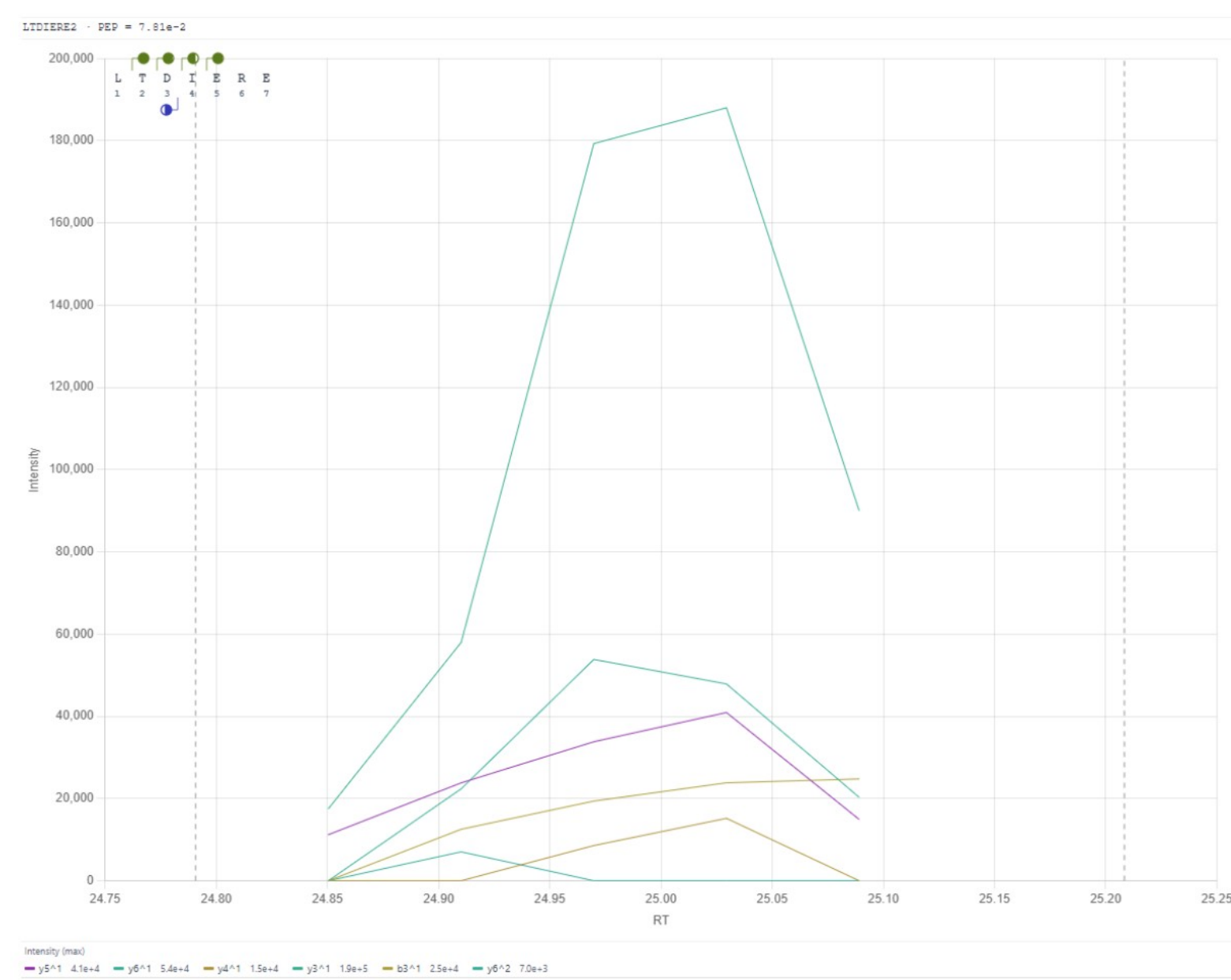

TDO2

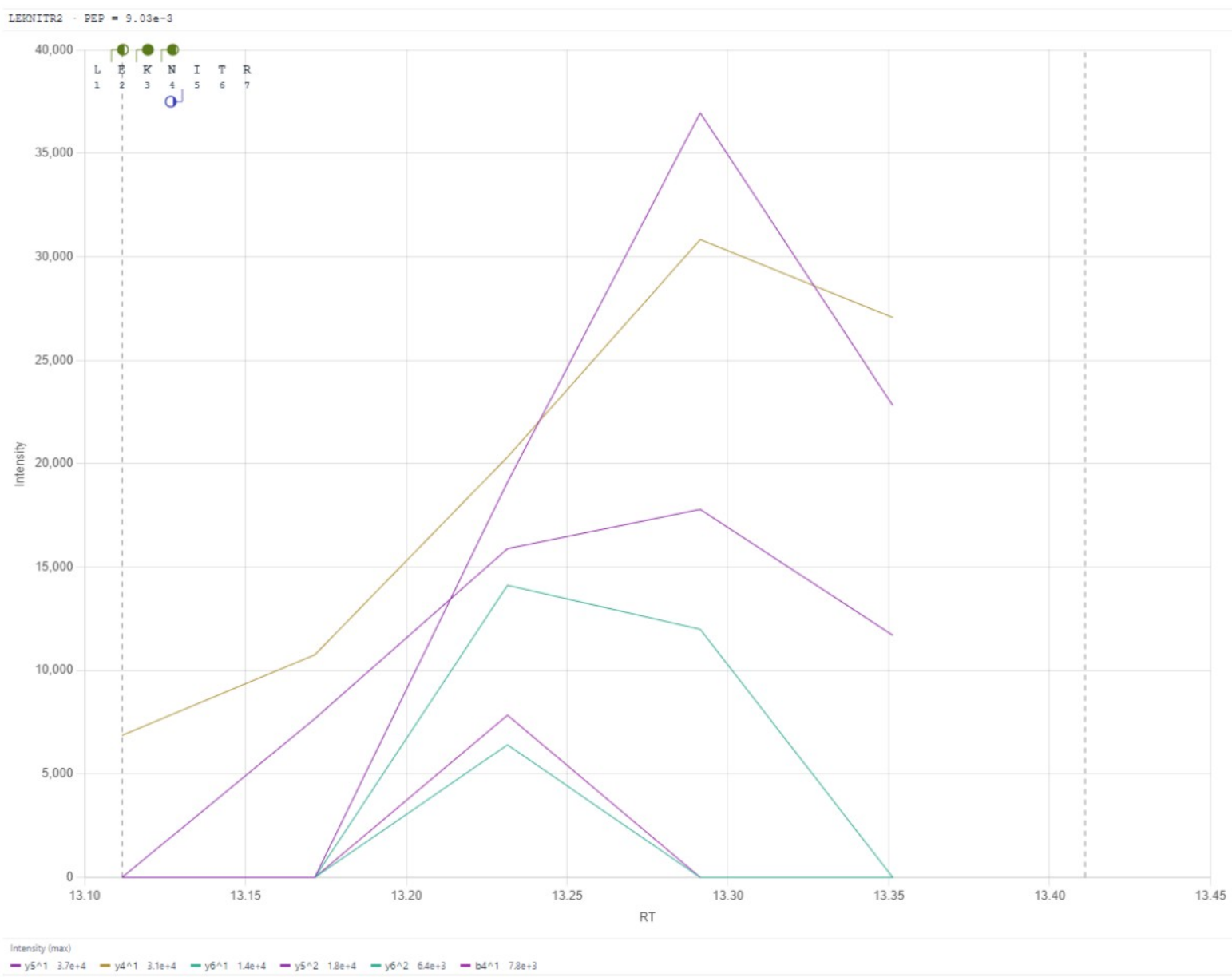

thrC

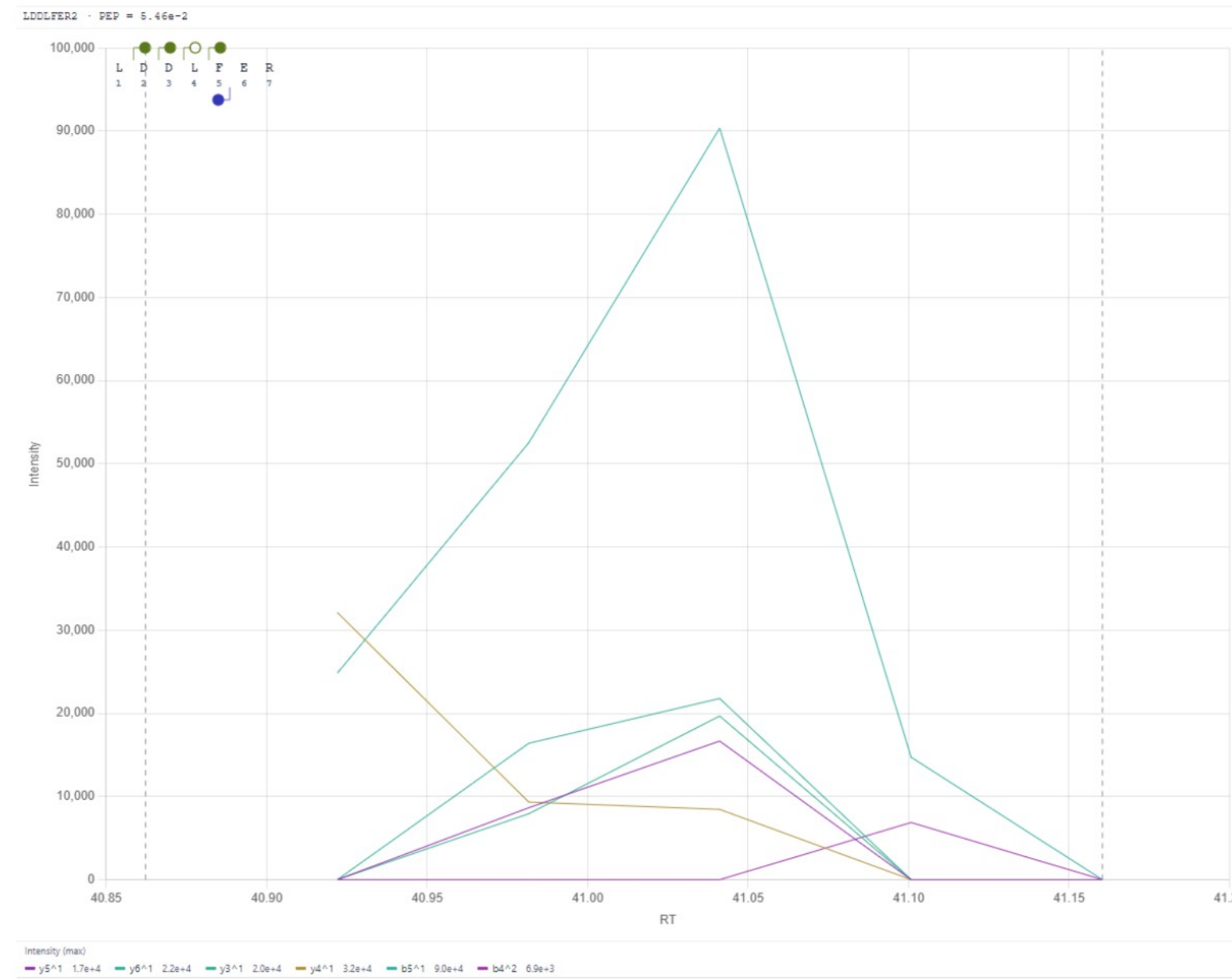

TIM10

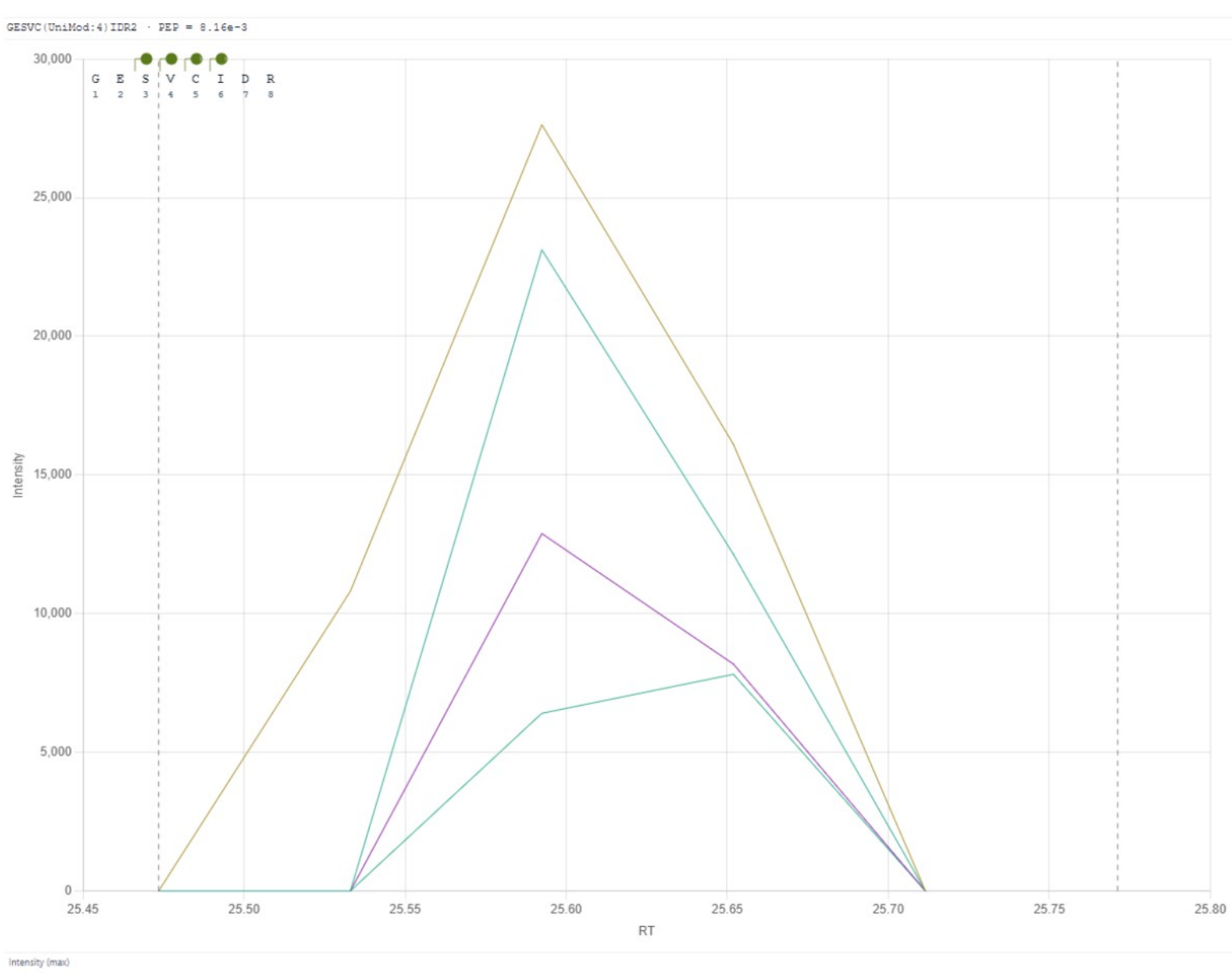

TMEM256

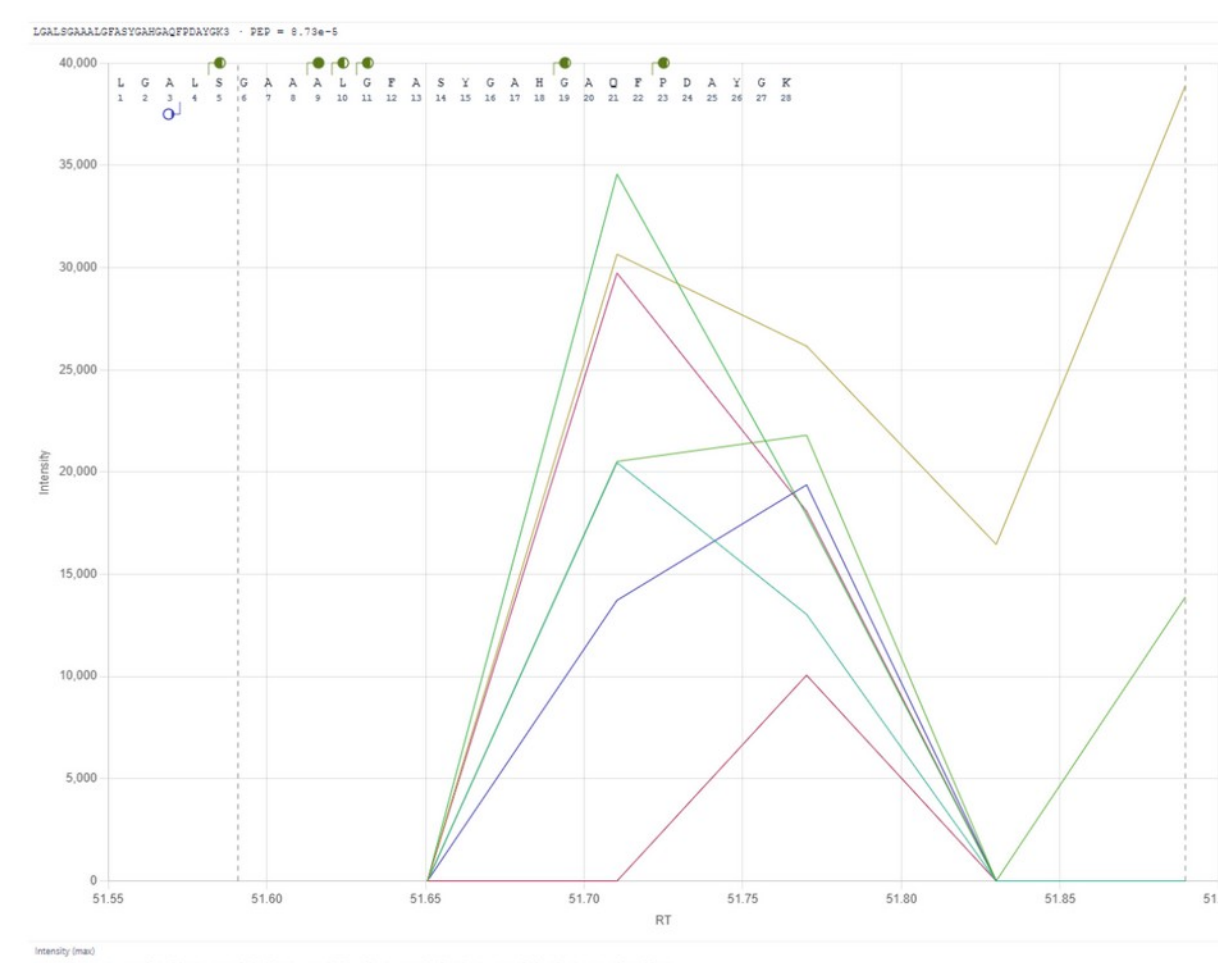

TMX4

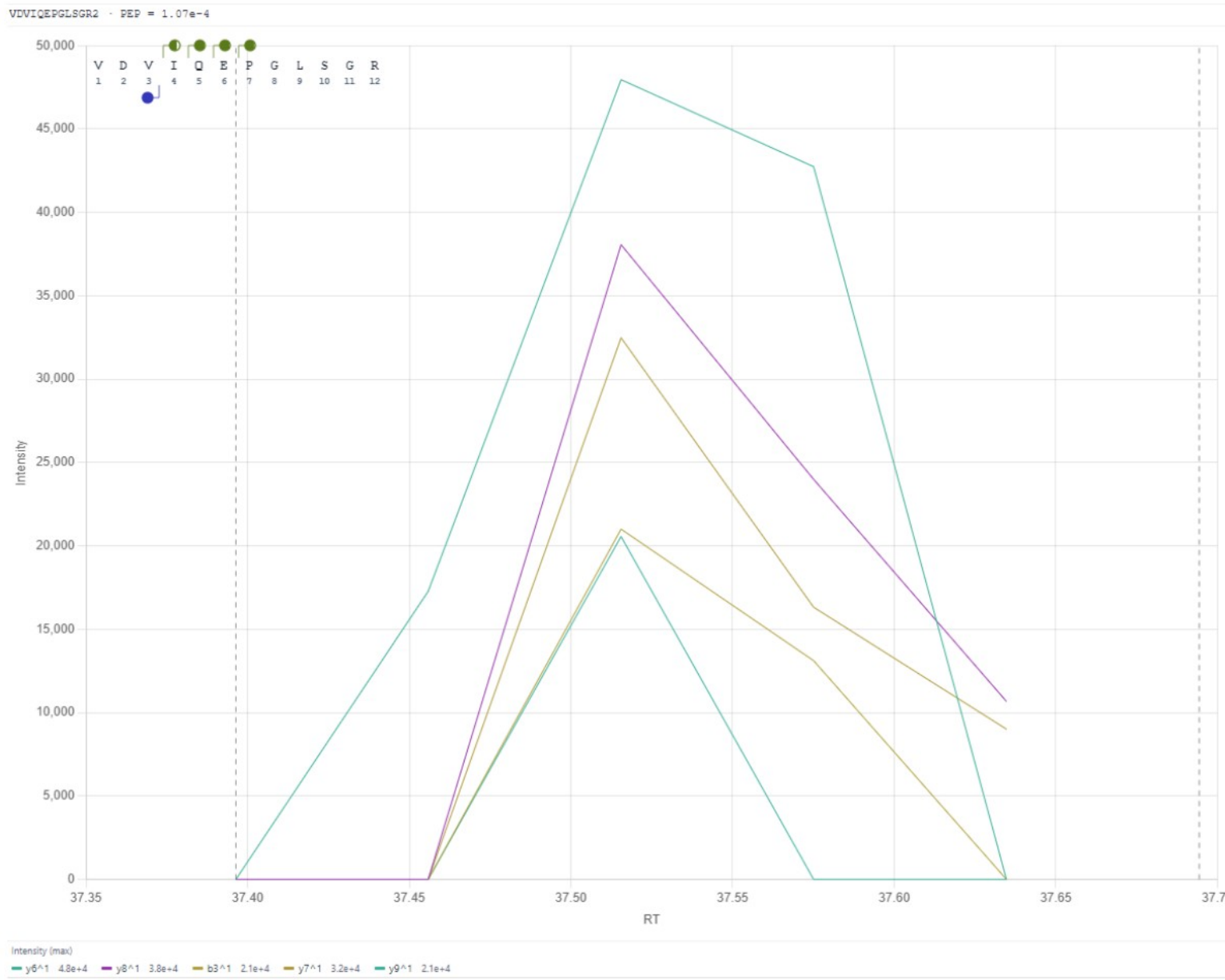

TNPO2

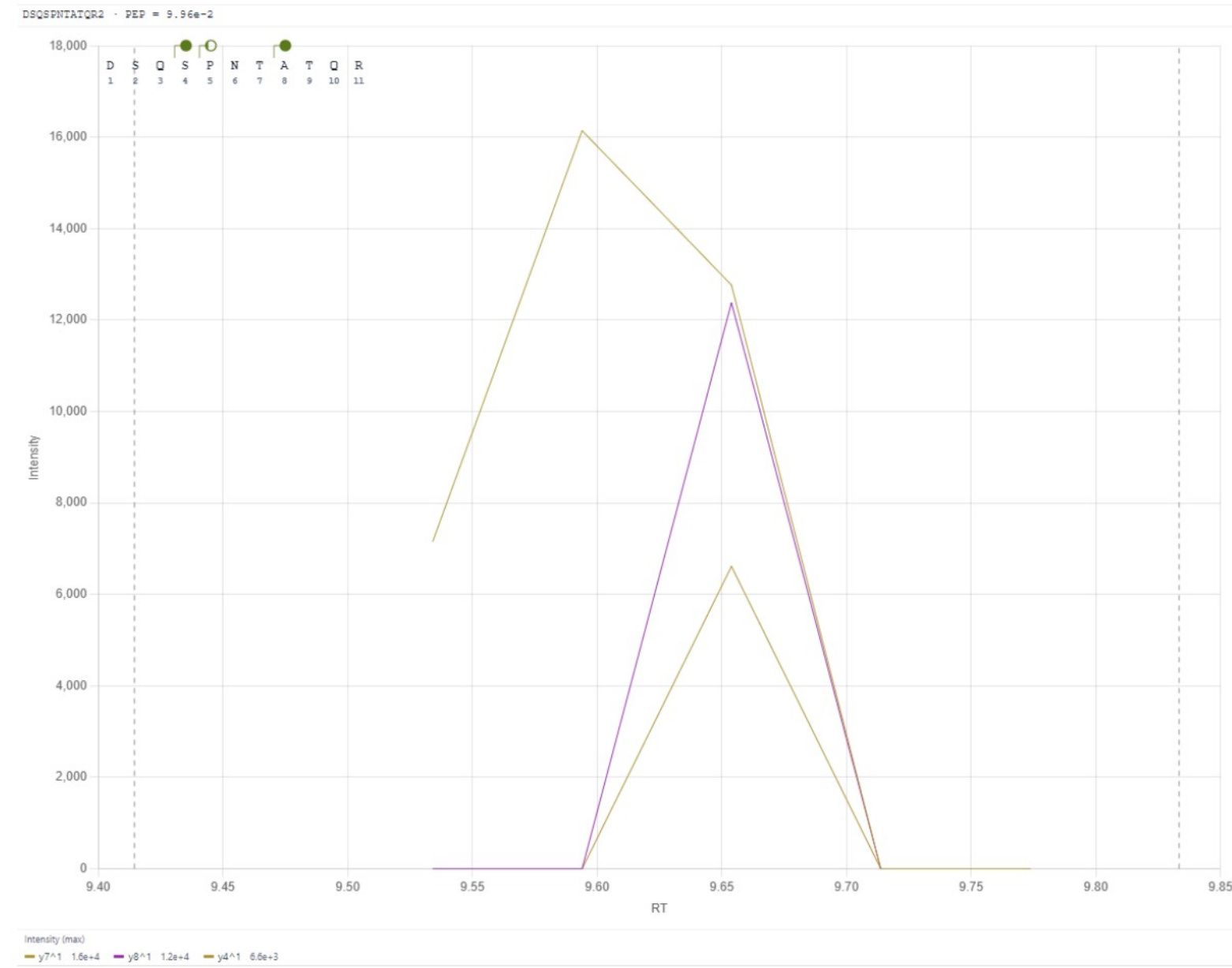

TNS1

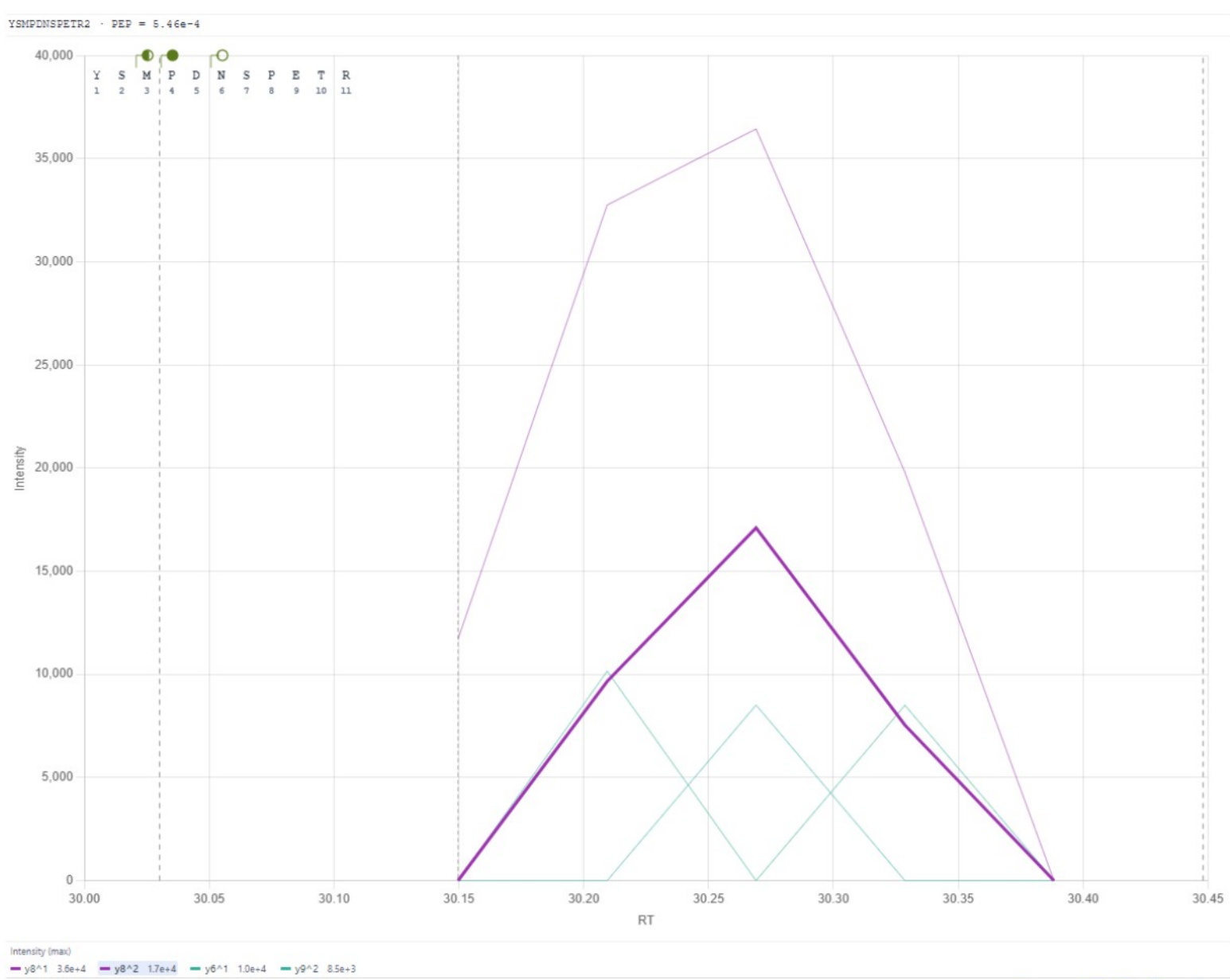

TOM1L1

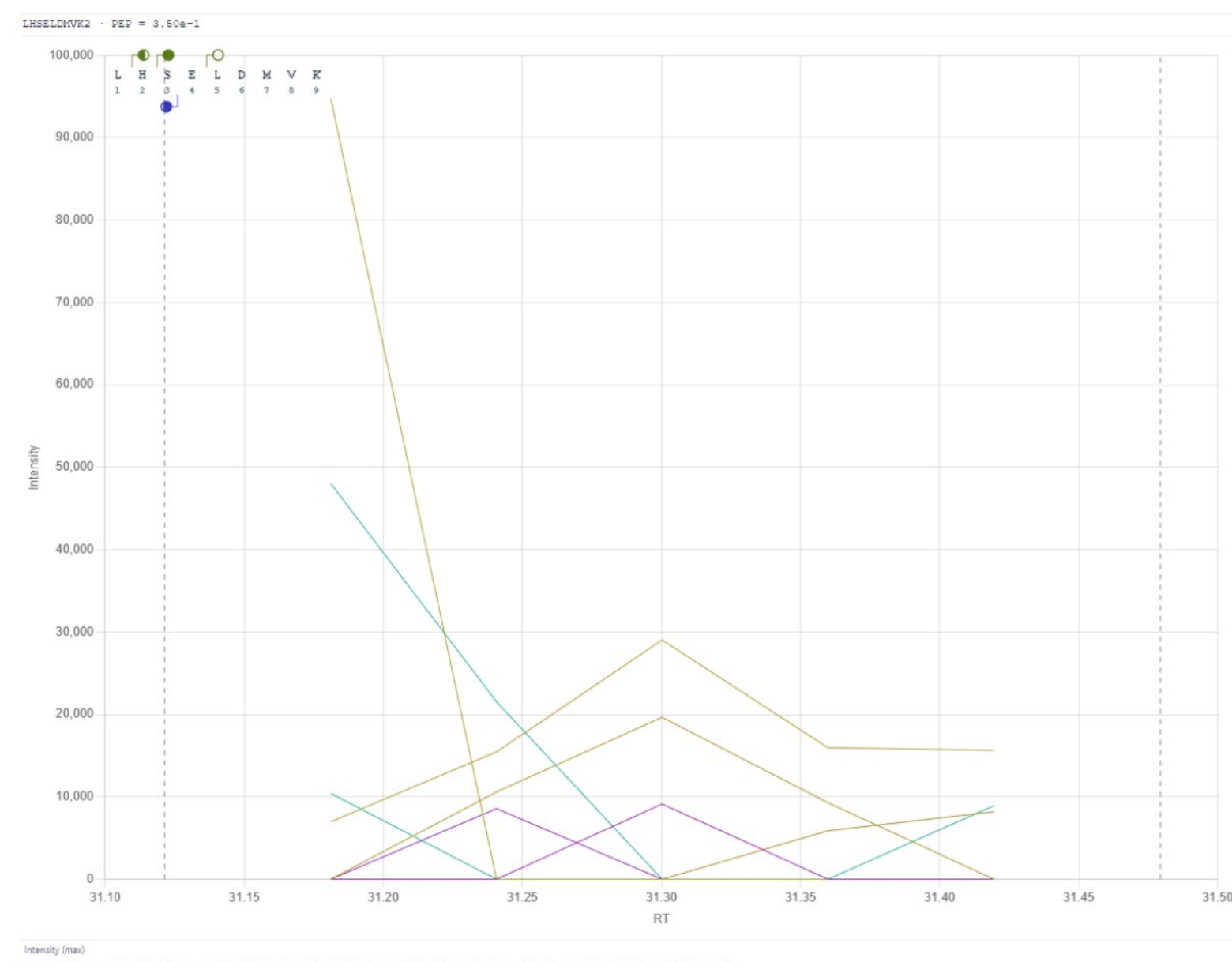

TOMM22

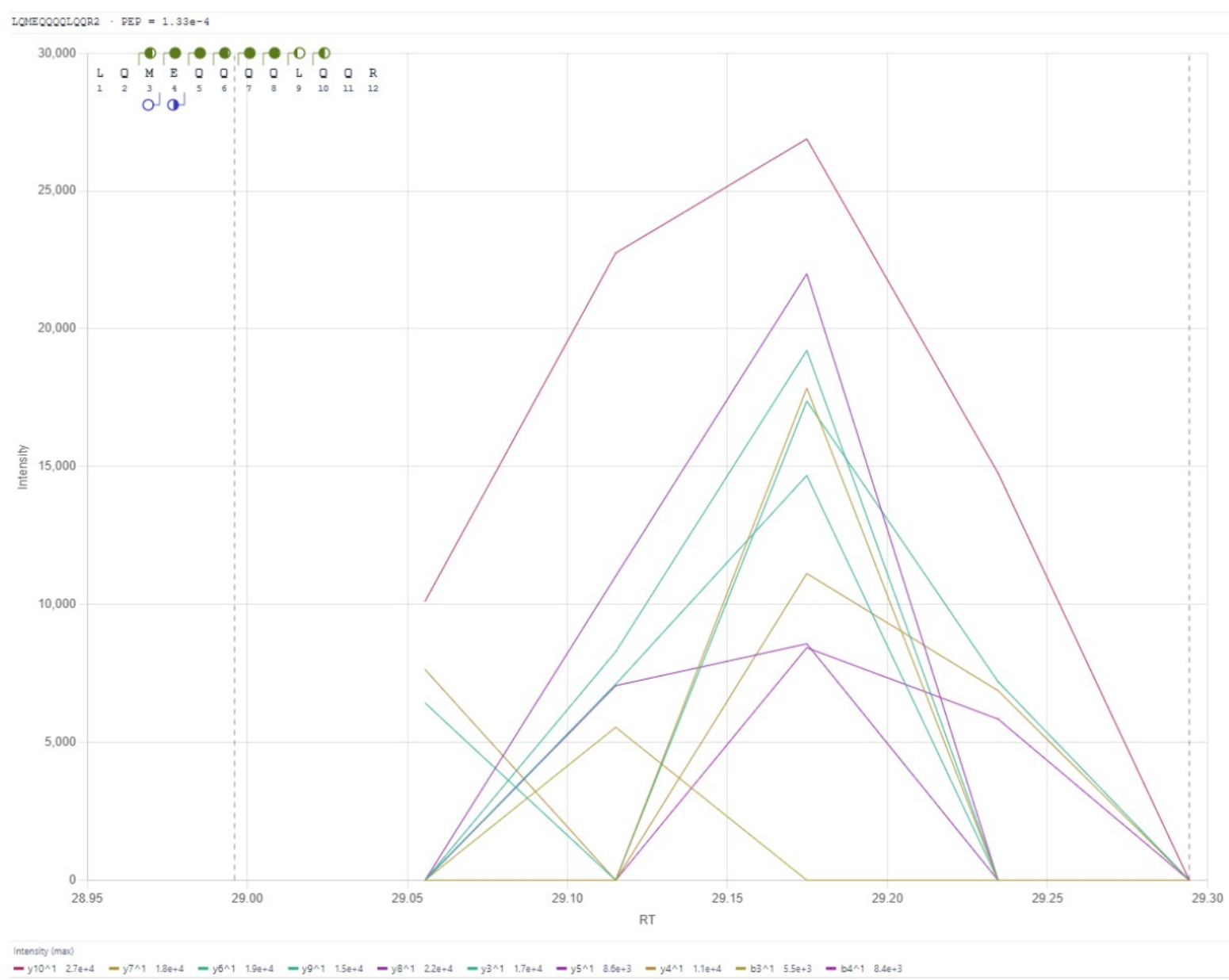

TOR1A

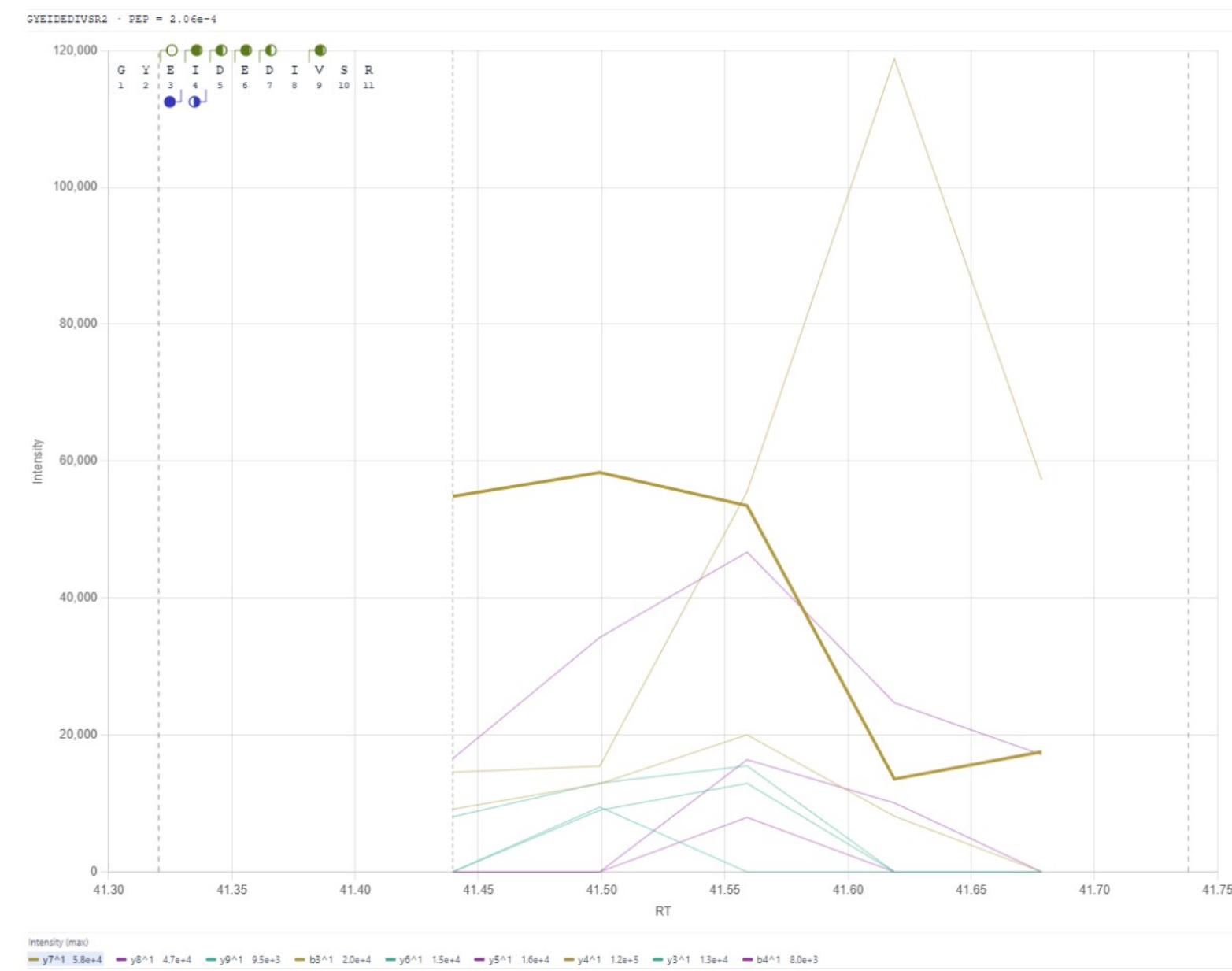

TRAPPC5

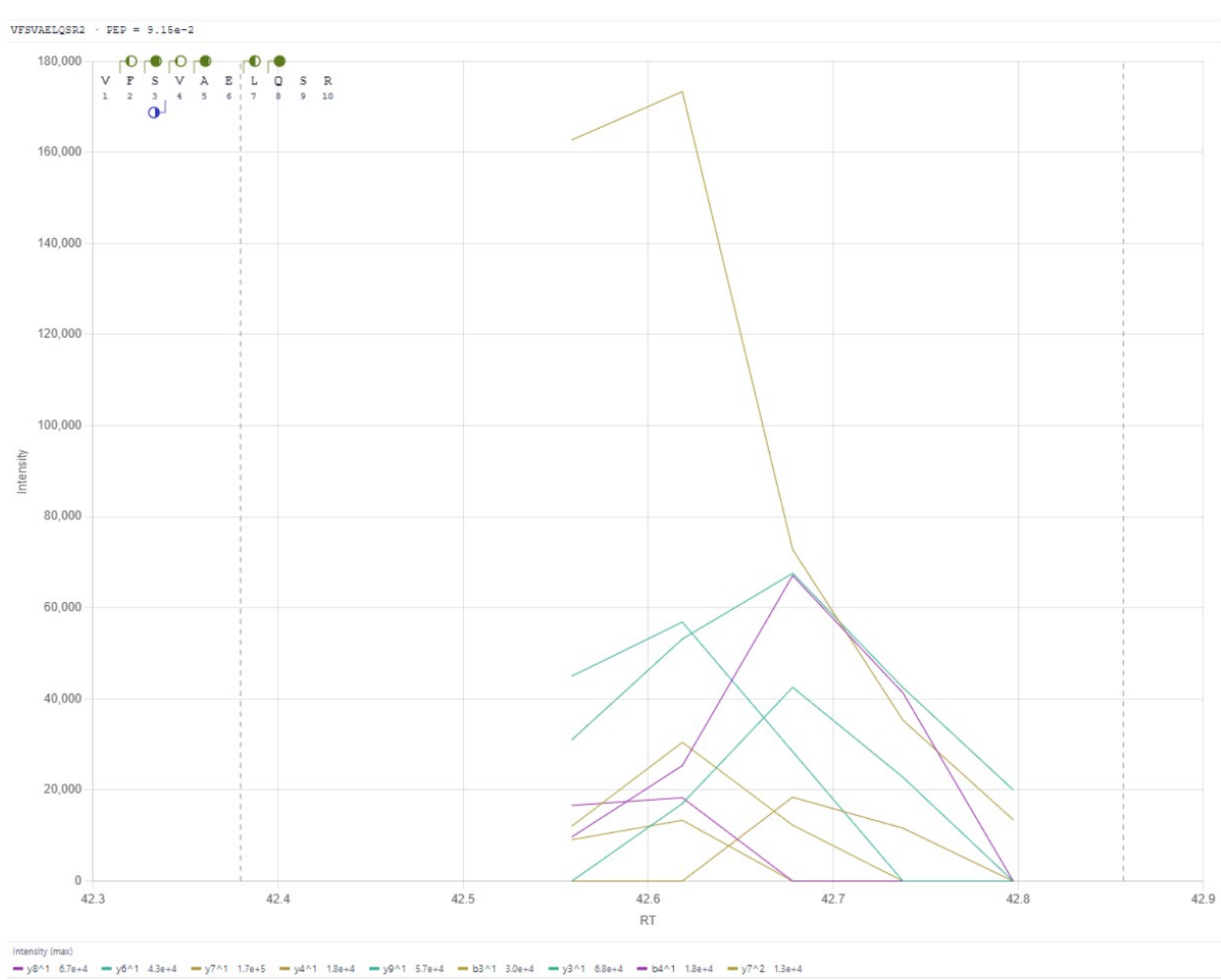

TRPV2

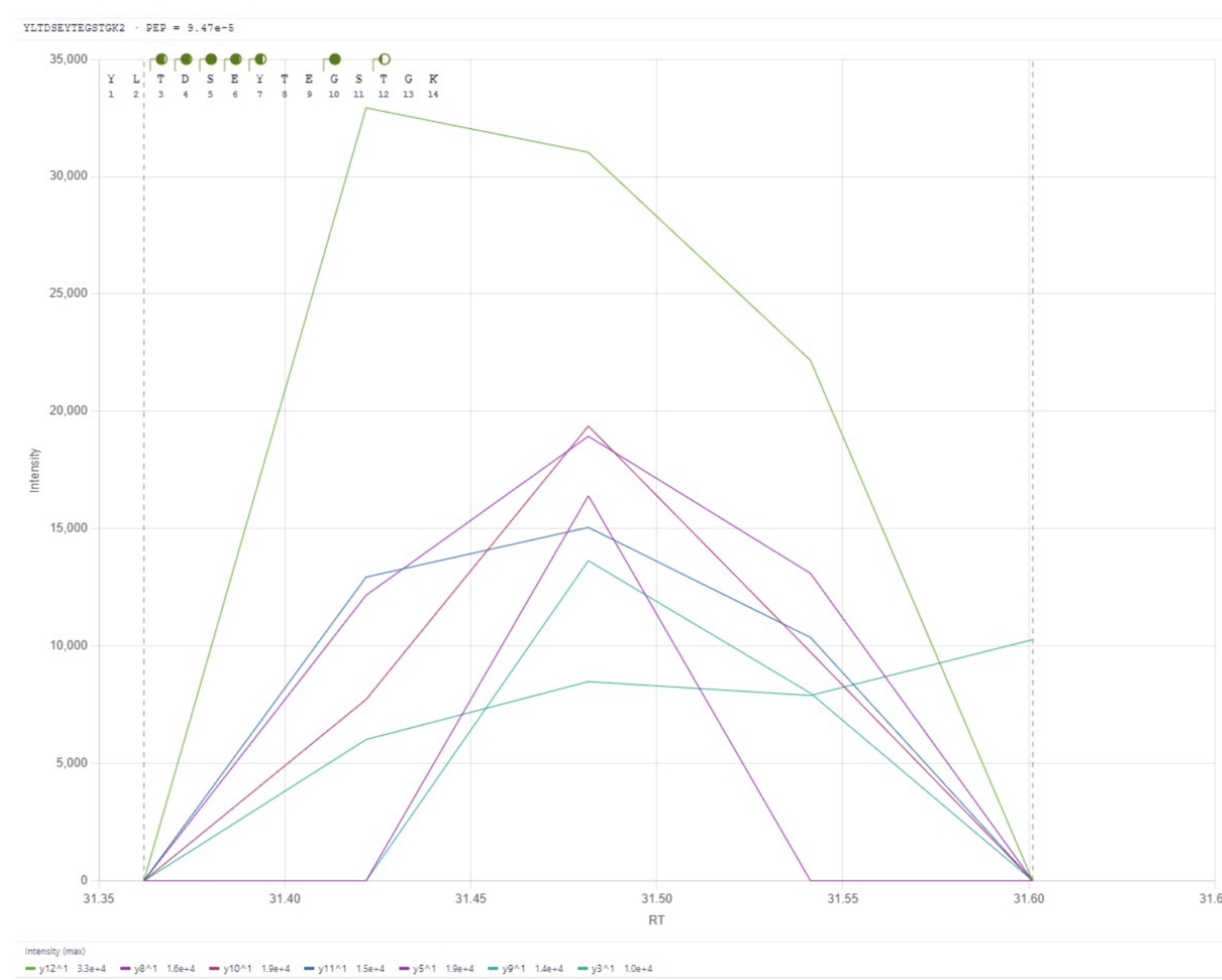

TSC22D3

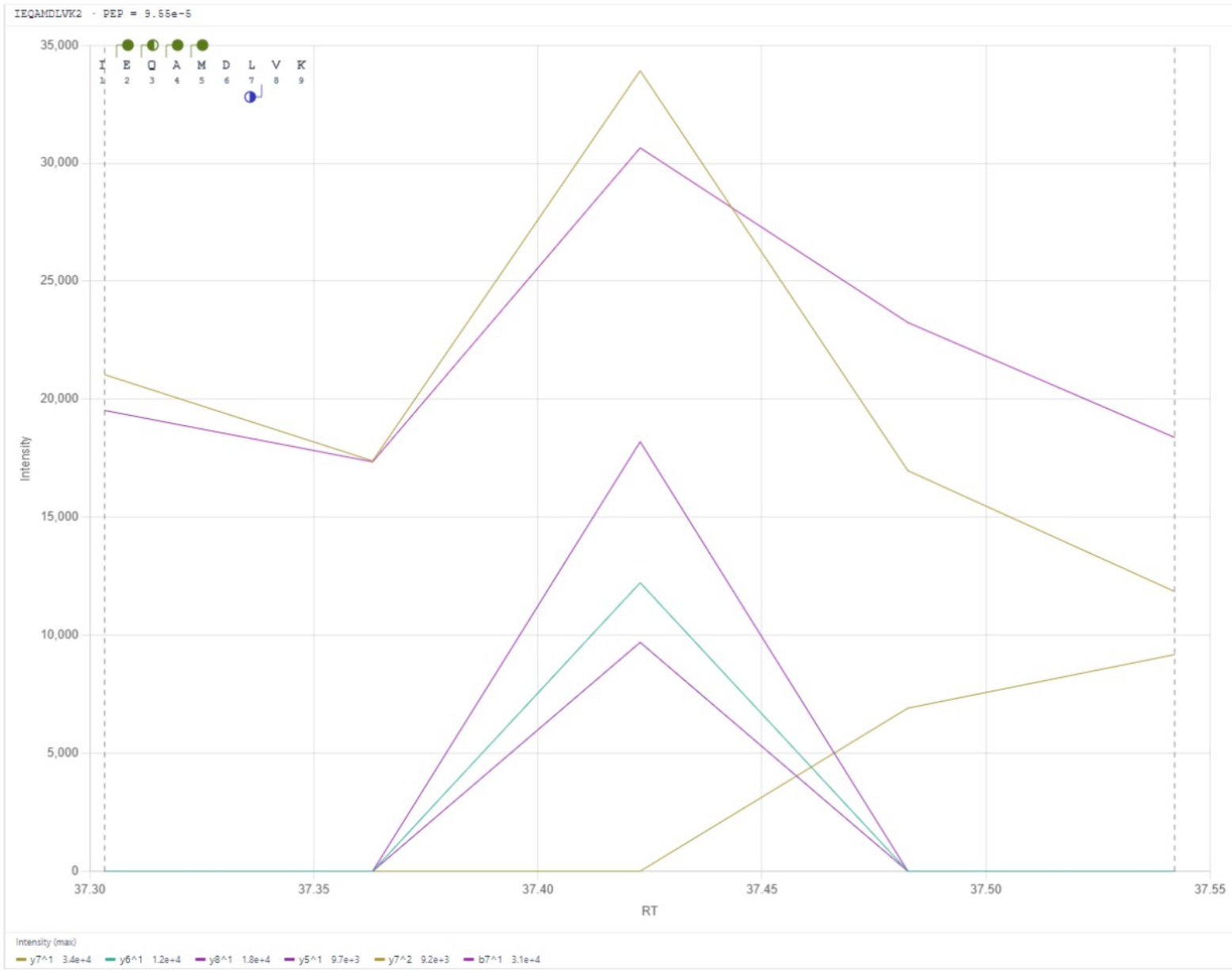

TSFM

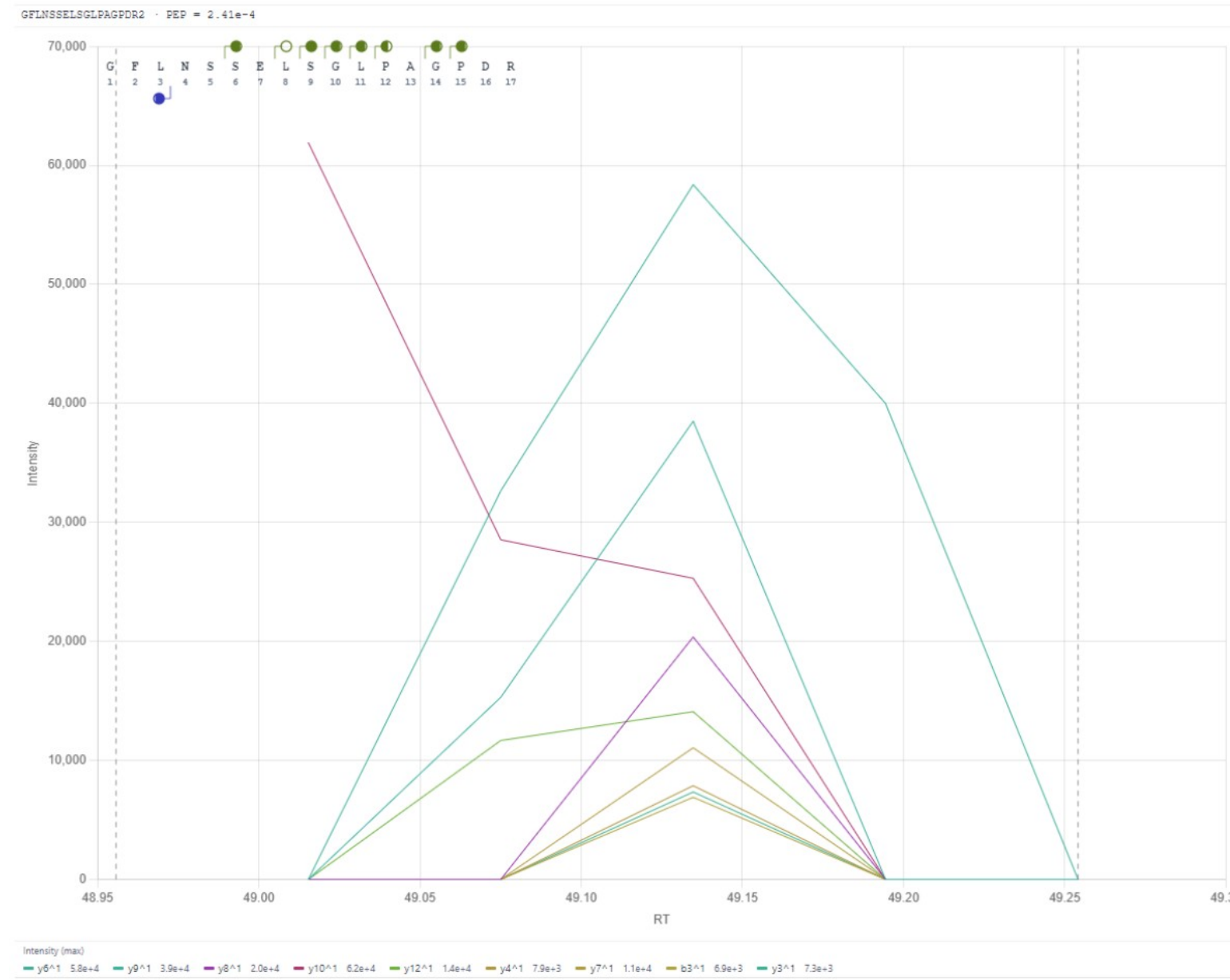

TTLL13

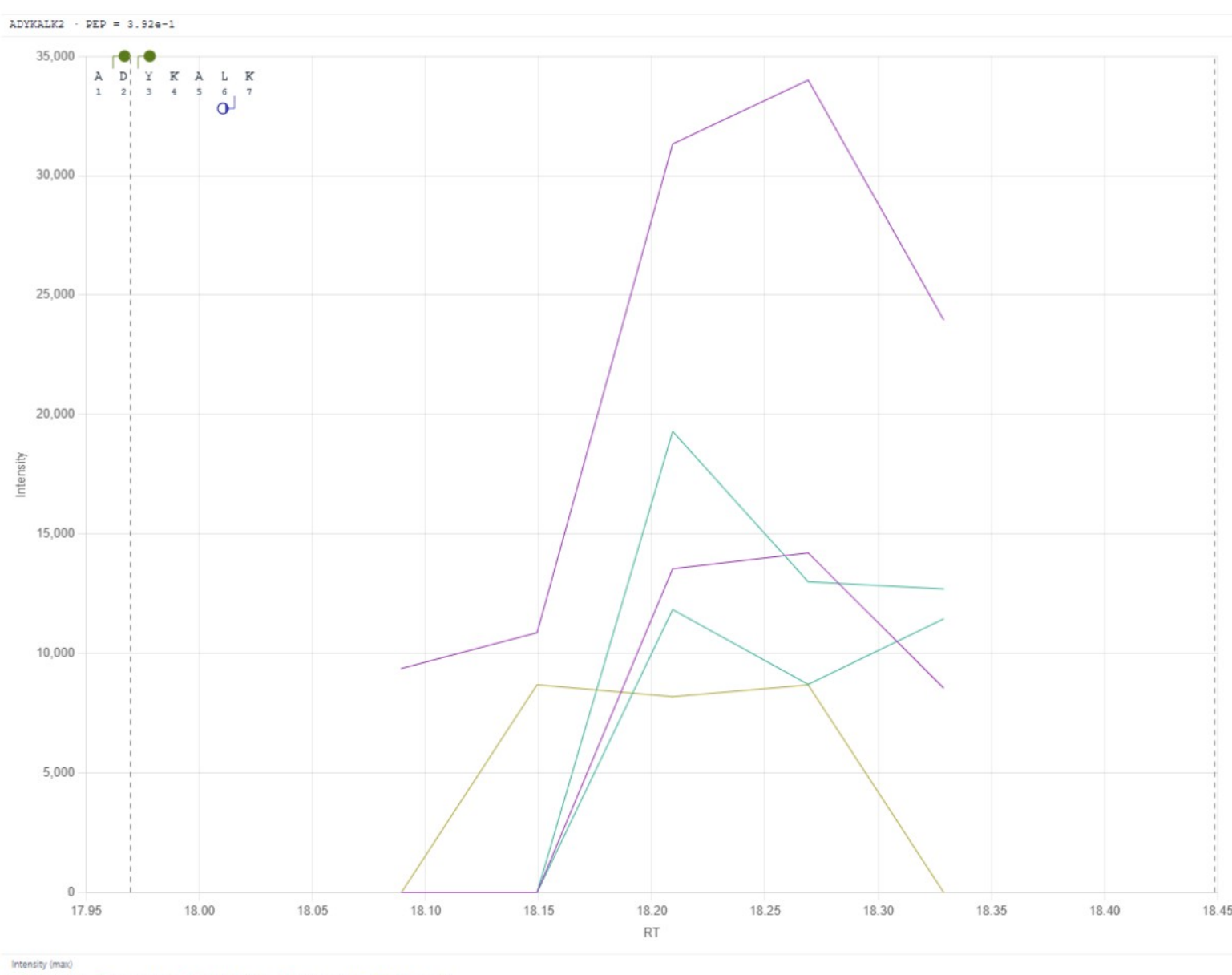

TUBAL3

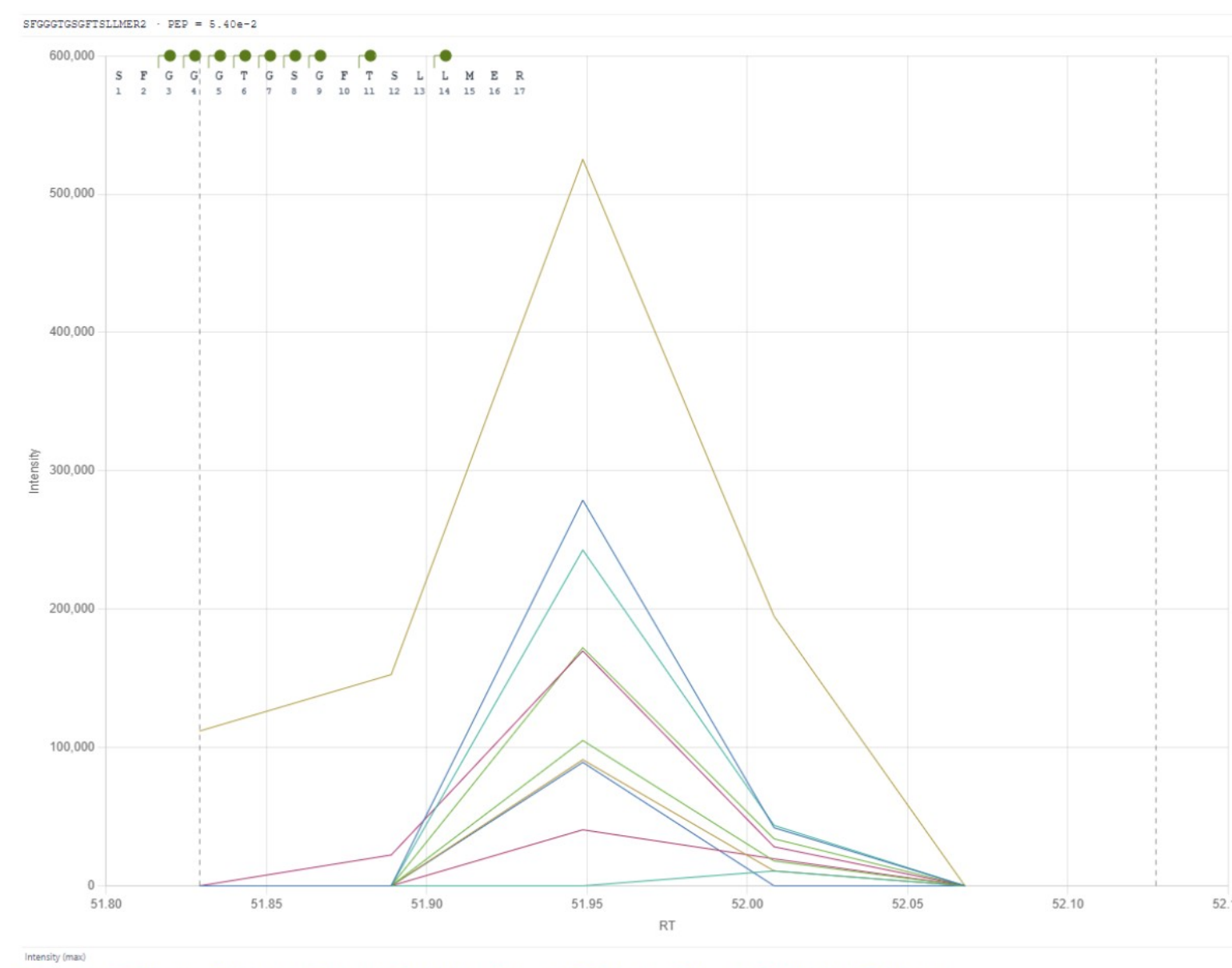

TUBB4B

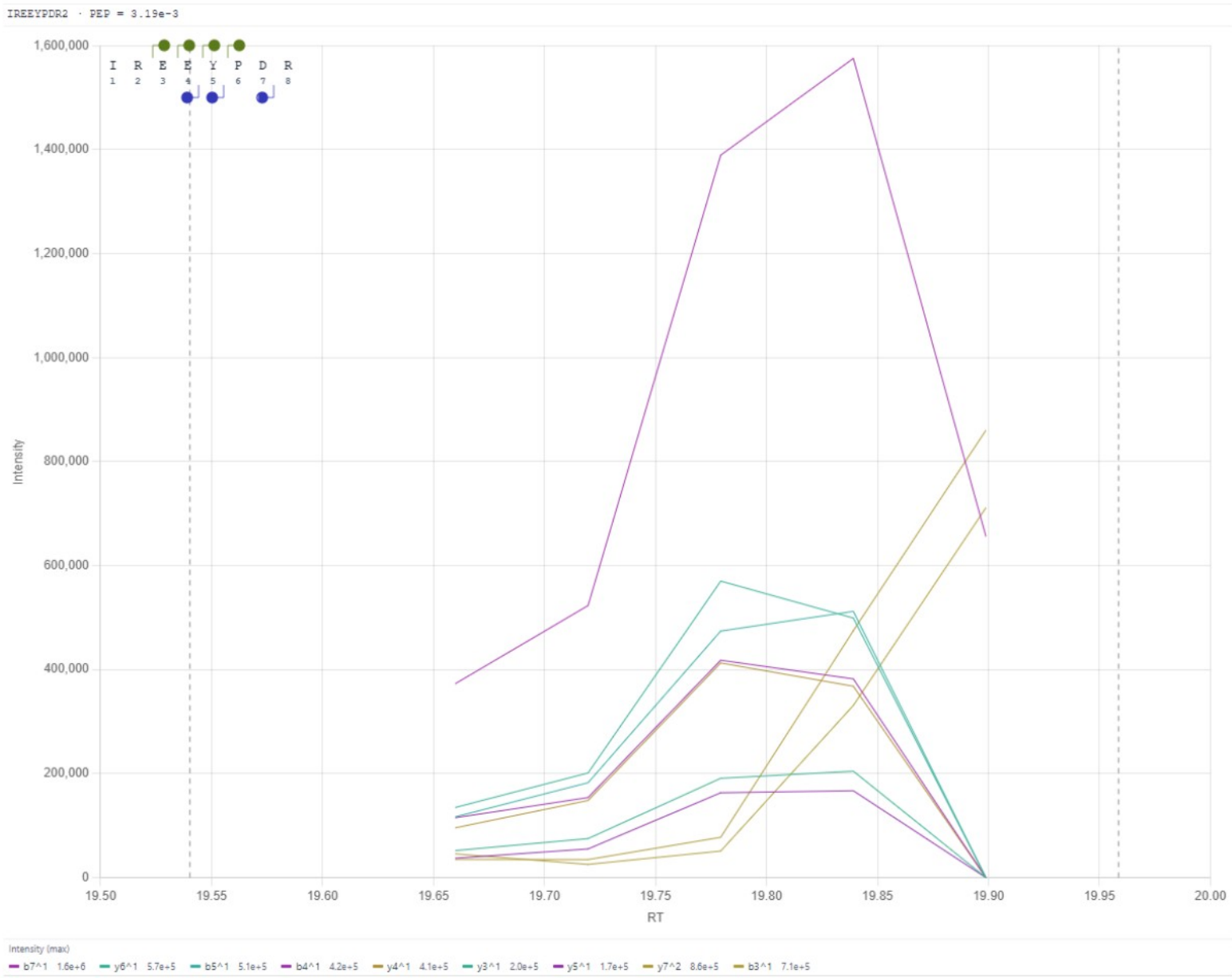

TXN2

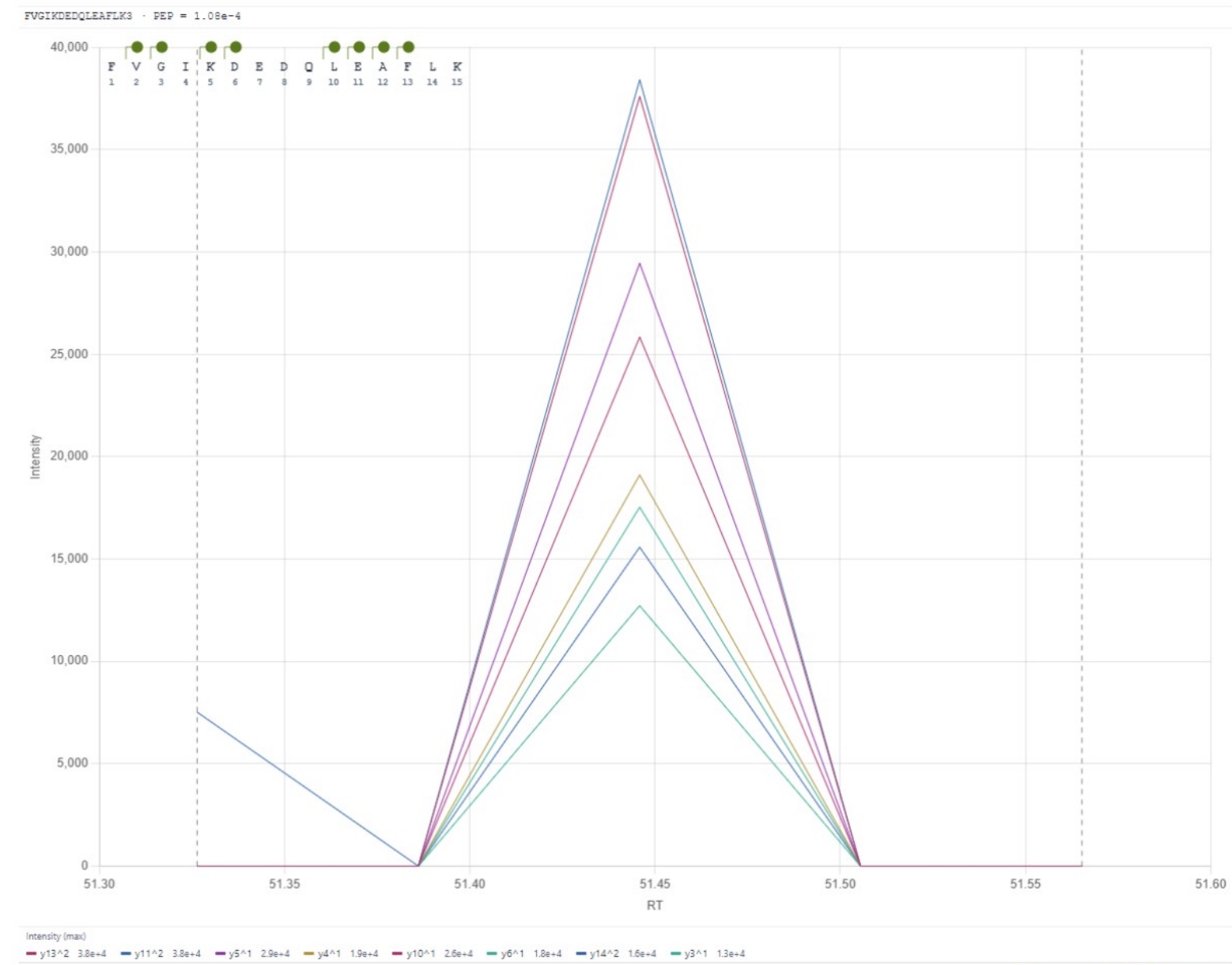

TXNRD1

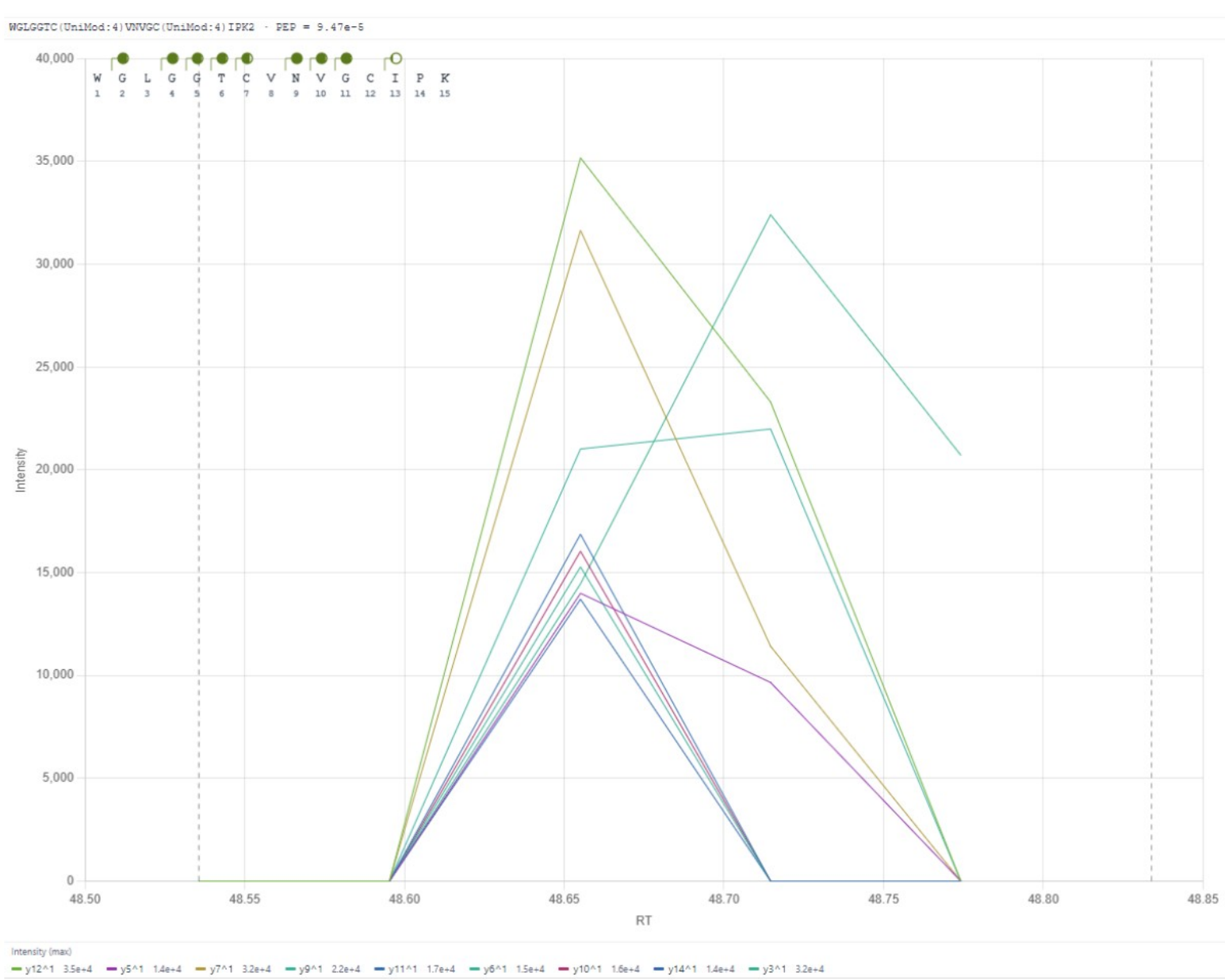

UGGT1

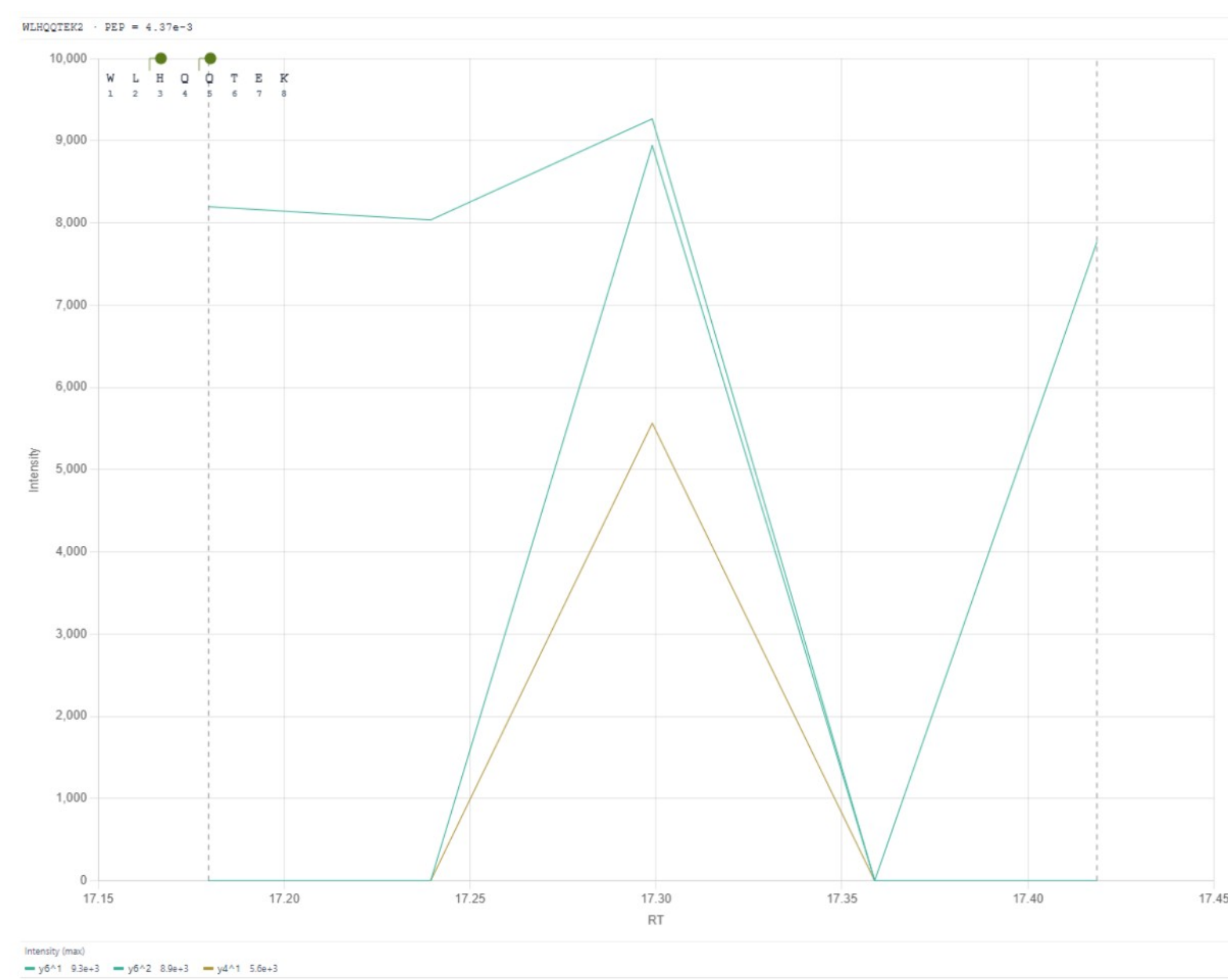

URM1

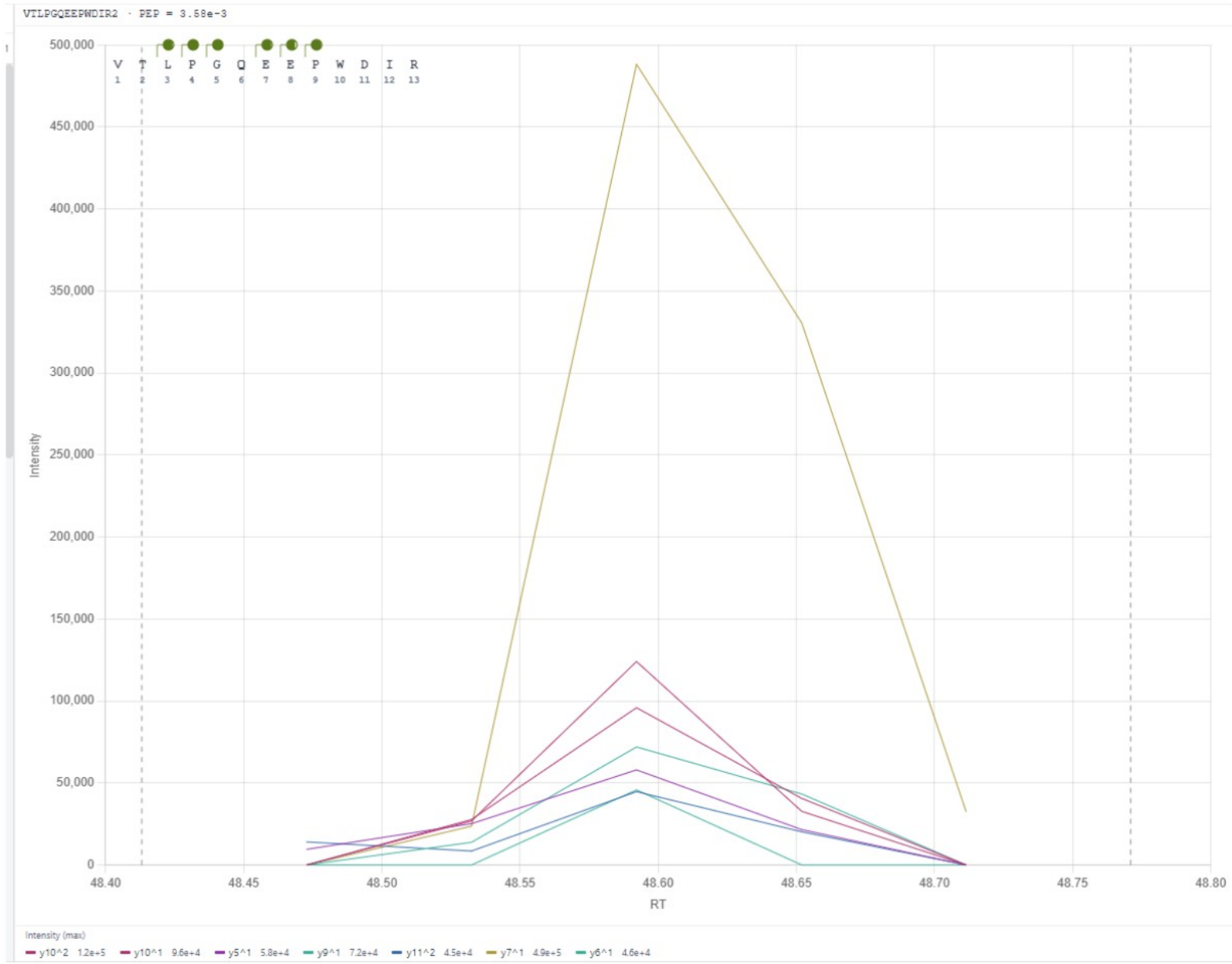

USE1

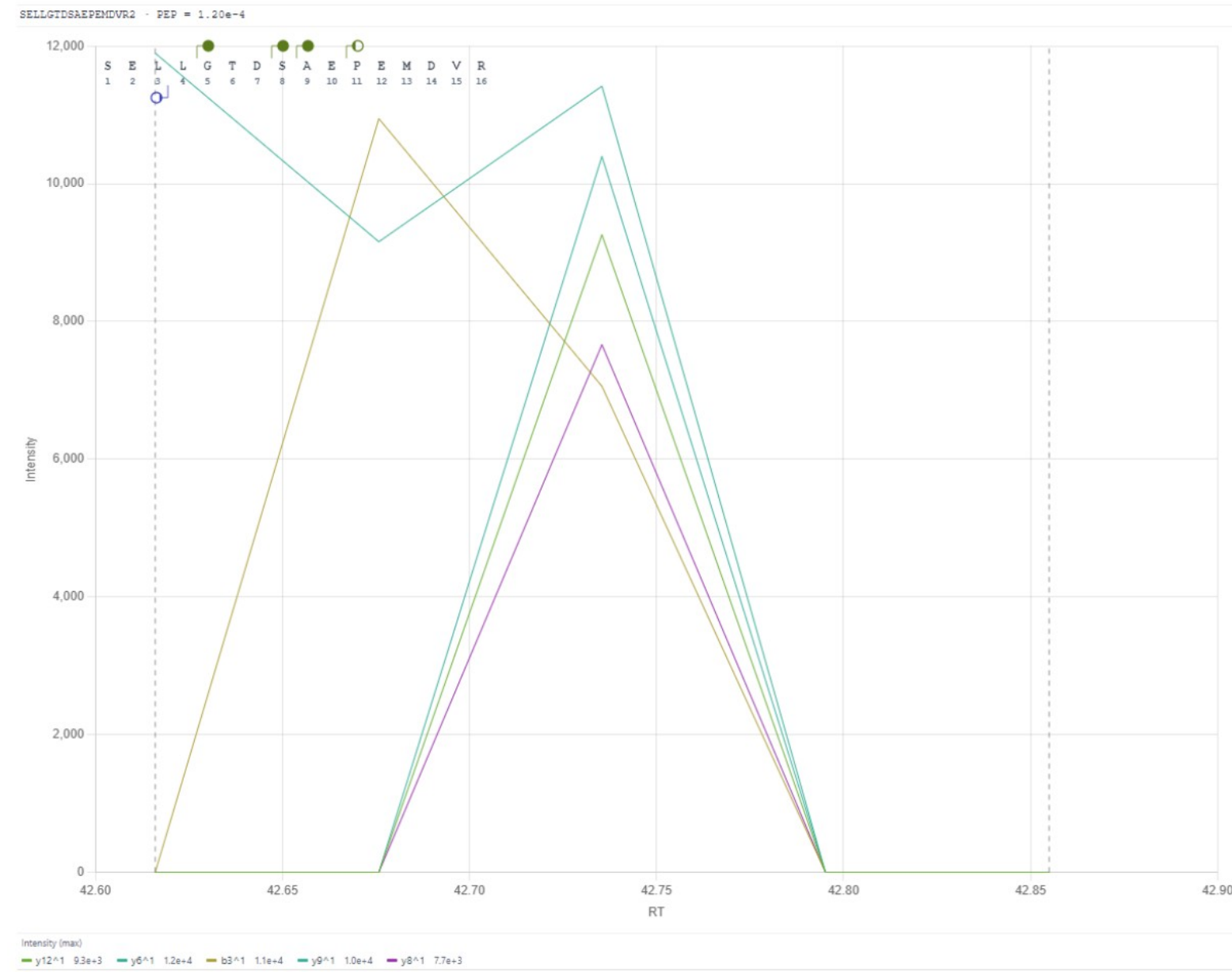

VAPA

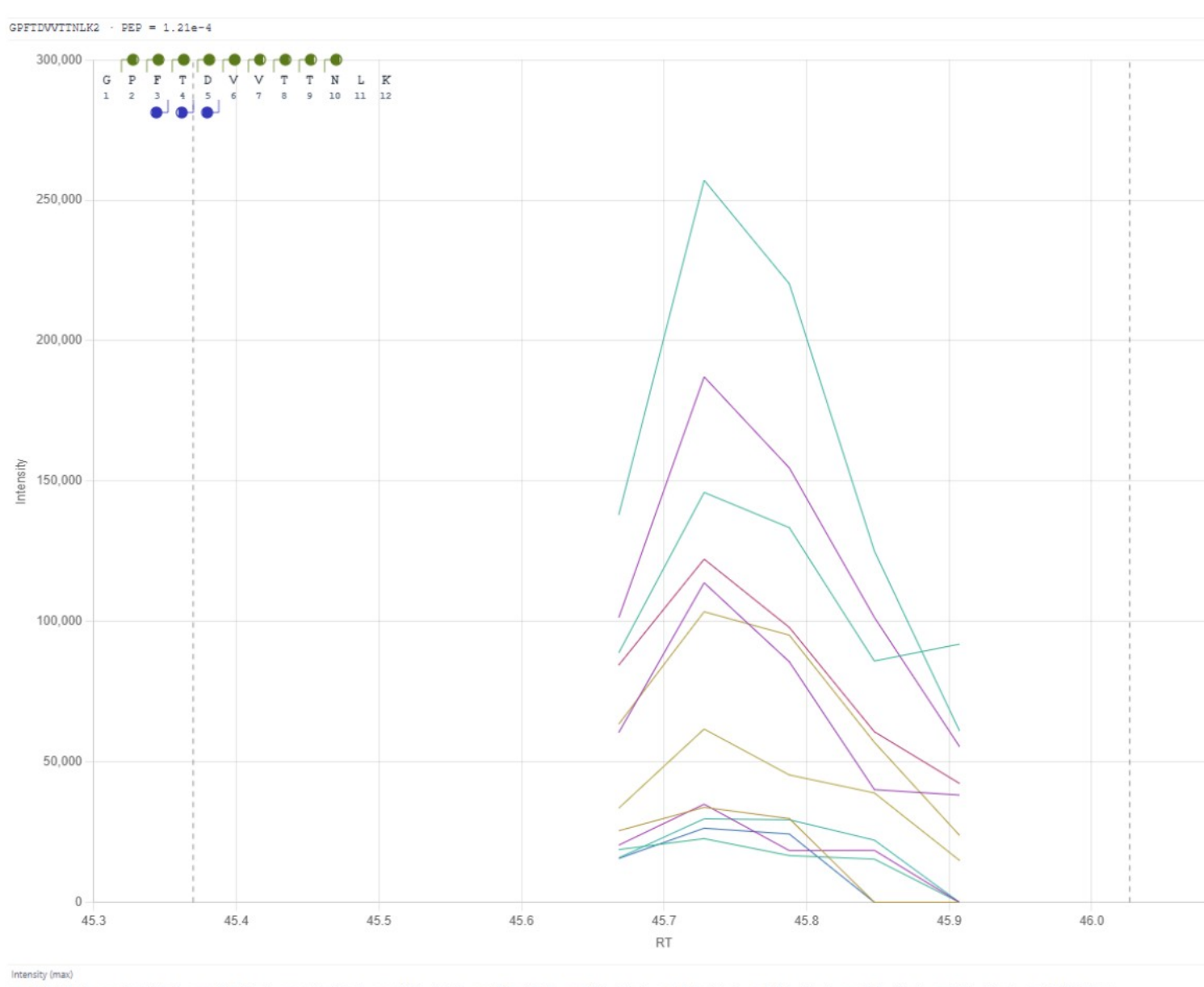

VAV1

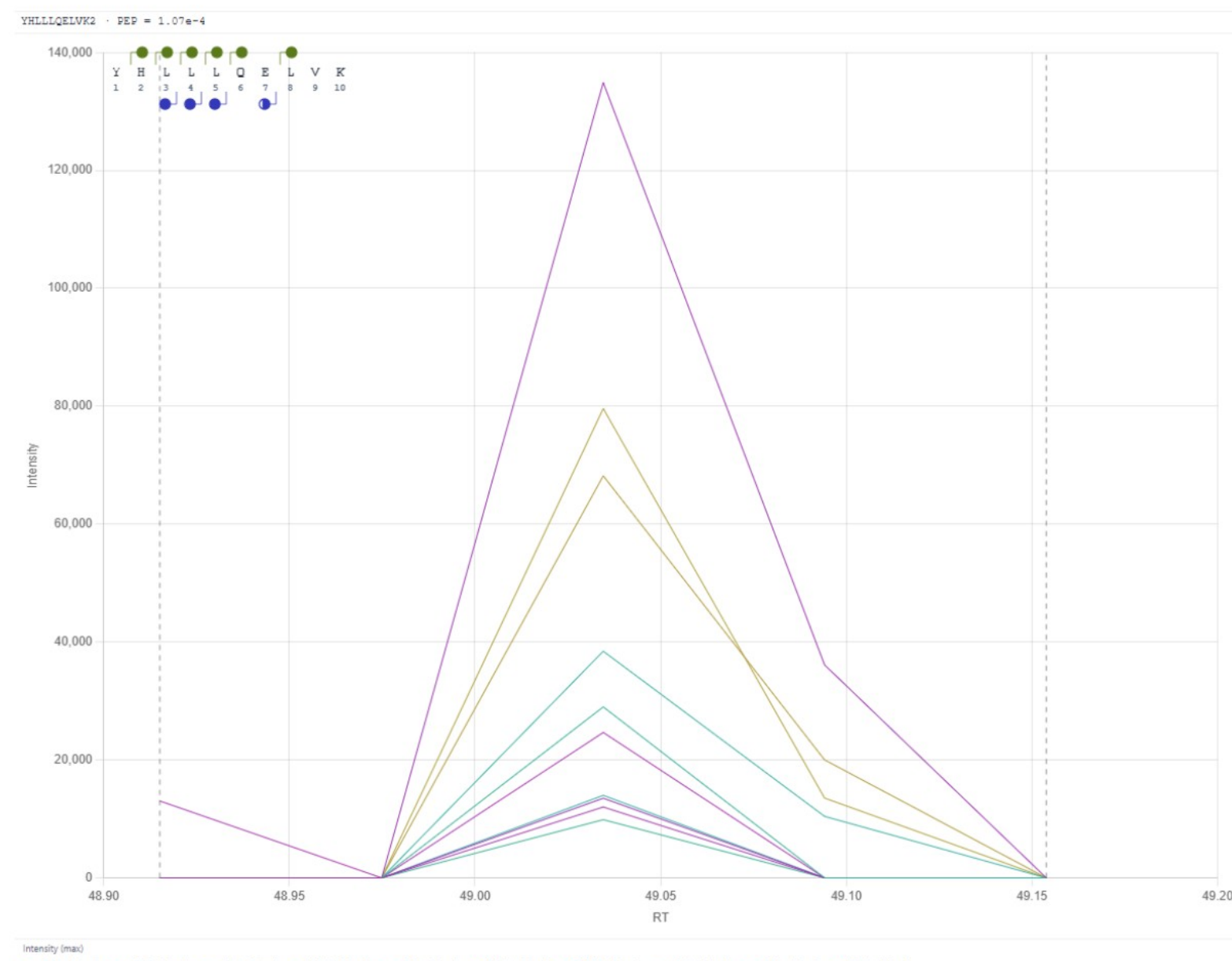

VPS26B

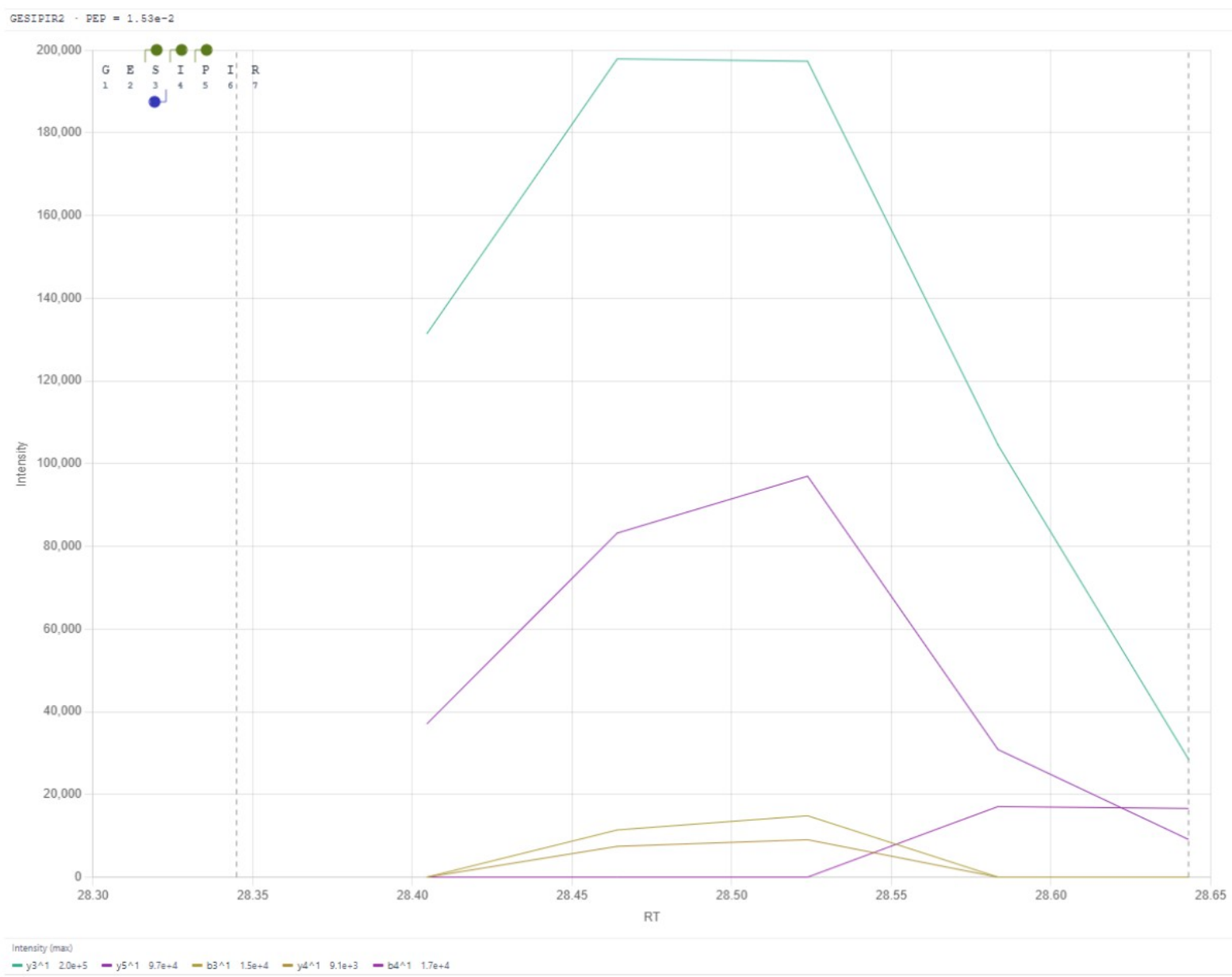

VPS37A

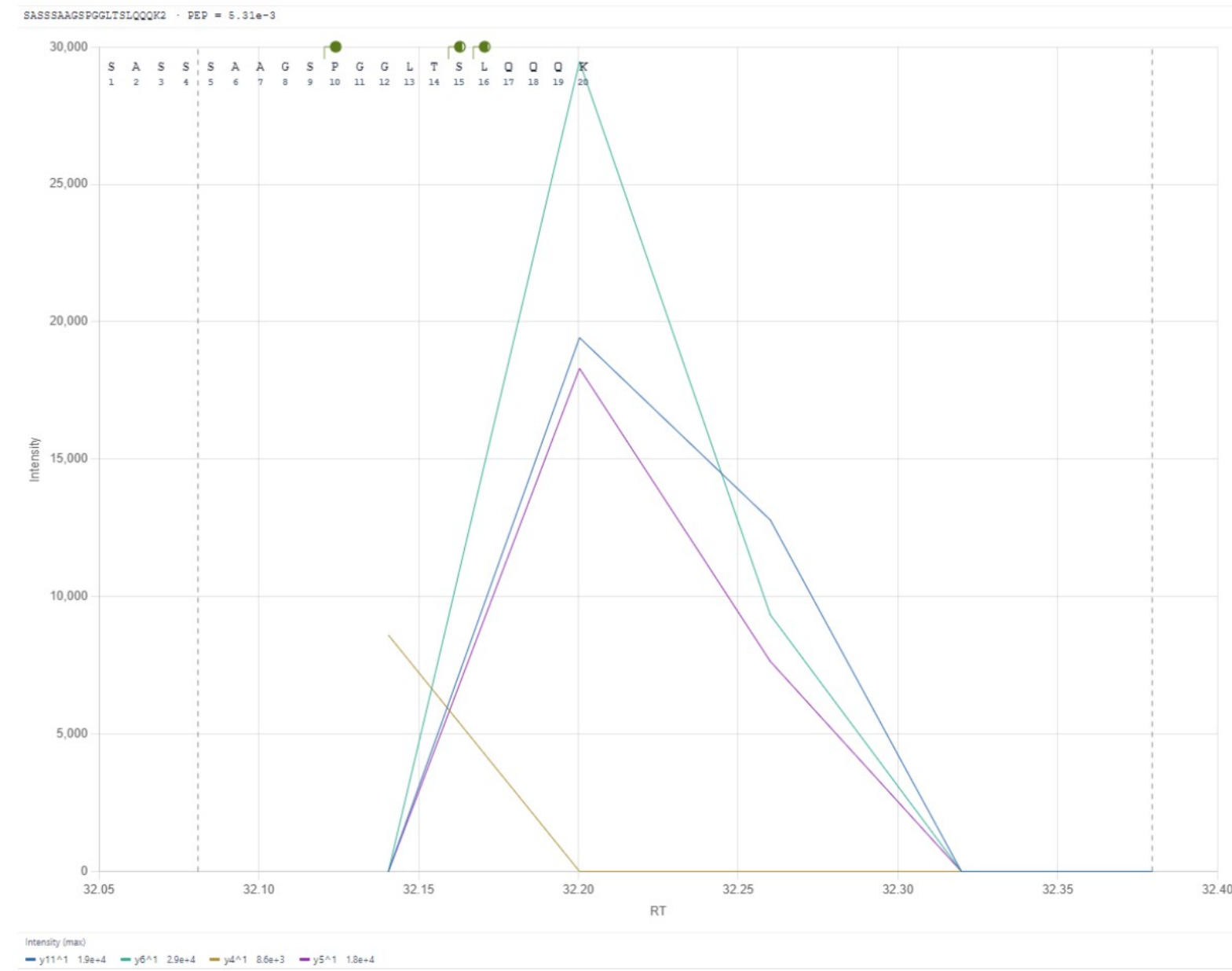

WASF3

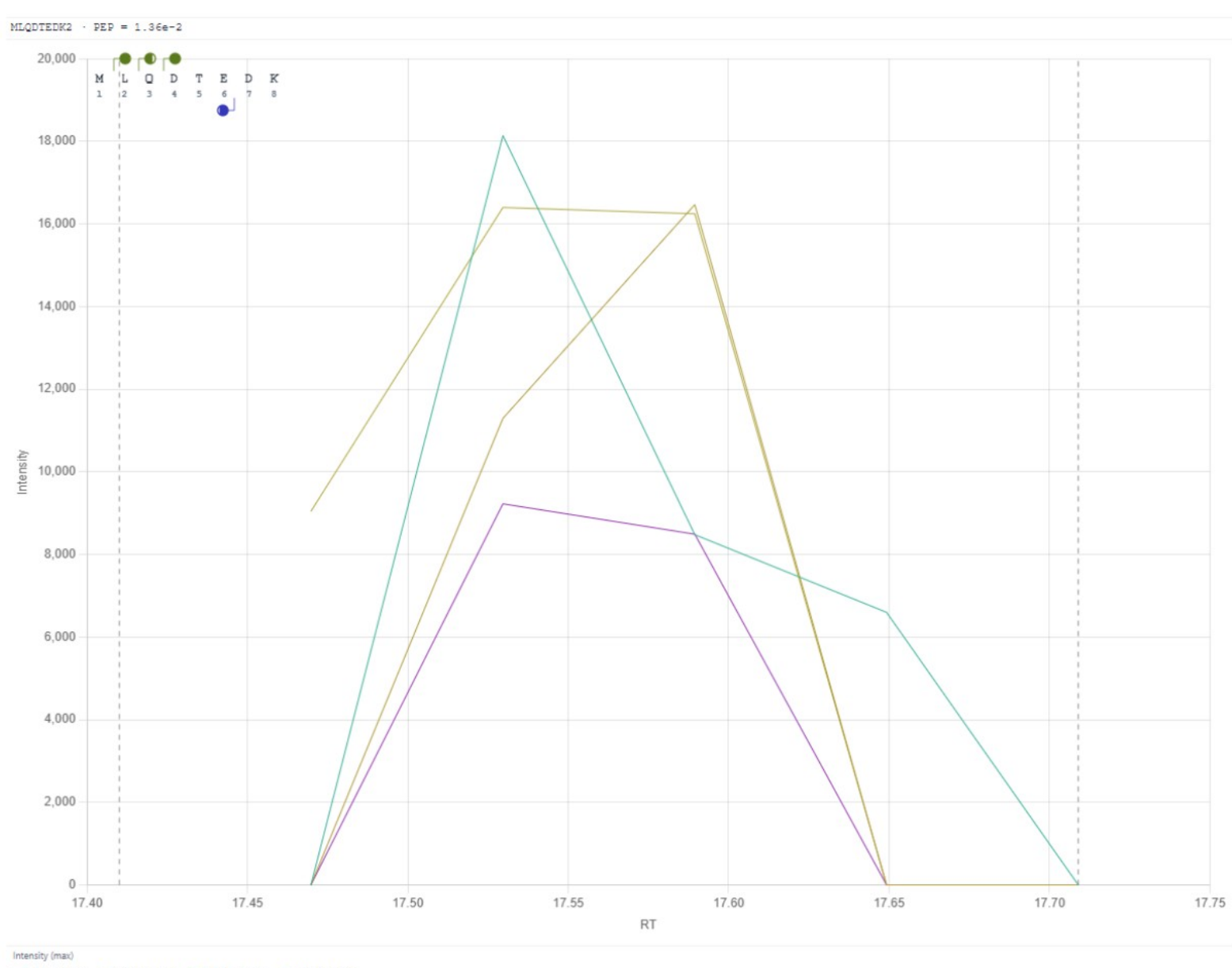

XK

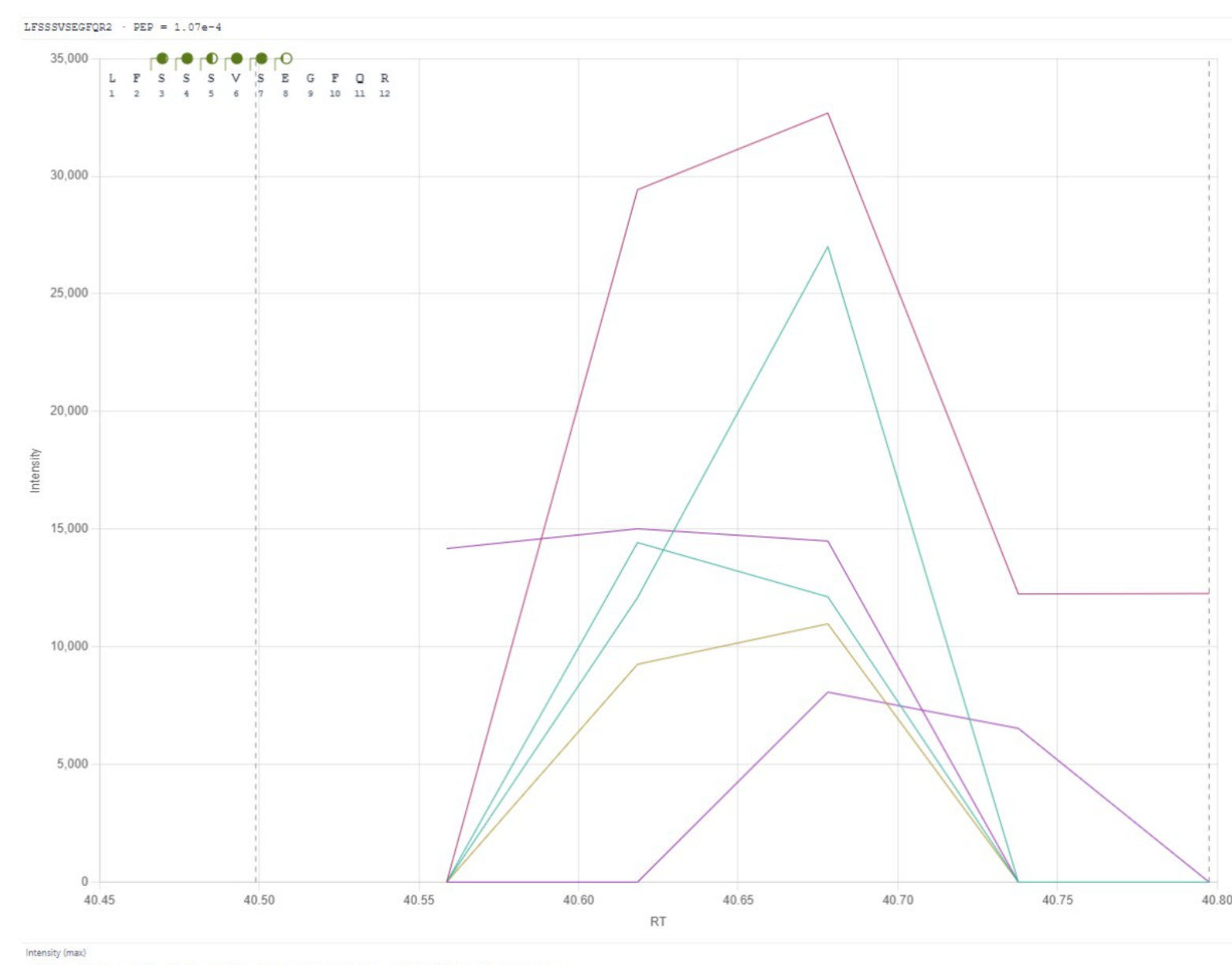

YIF1B

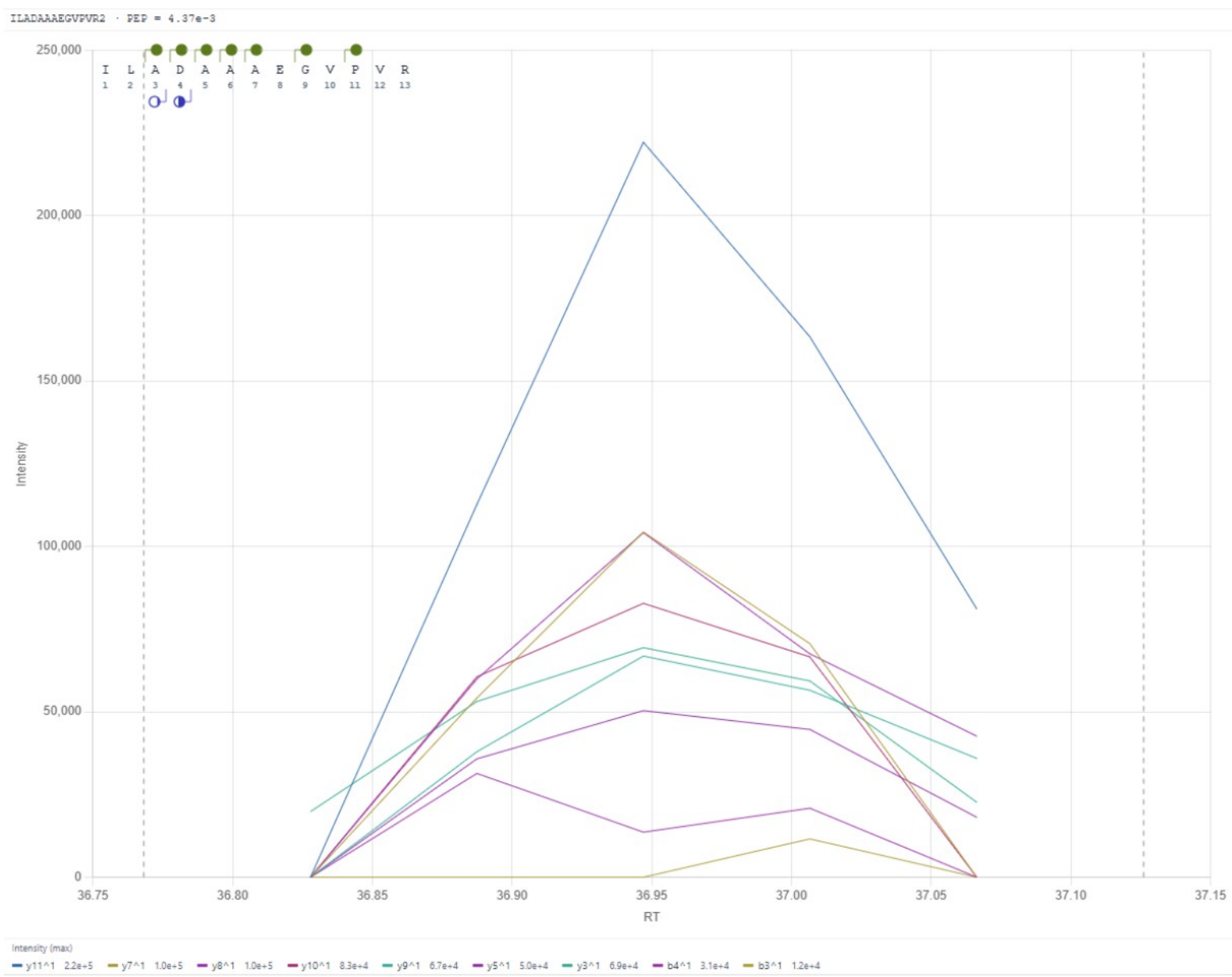
ILADAAAEGVPVR2 · PEP = 4.37e-3
I L A D A A A E G V P V R
1 2 3 4 5 6 7 8 9 10 11 12 13
250,000
200,000
150,000
100,000
50,000
0
Intensity
36.75
36.80
36.85
36.90
36.95
37.00
37.05
37.10
37.15
RT
Intensity (max)
y11^1 2.2e+5
y7^1 1.0e+5
y8^1 1.0e+5
y10^1 8.3e+4
y9^1 6.7e+4
y5^1 5.0e+4
y3^1 6.9e+4
b4^1 3.1e+4
b3^1 1.2e+4

YWHAG

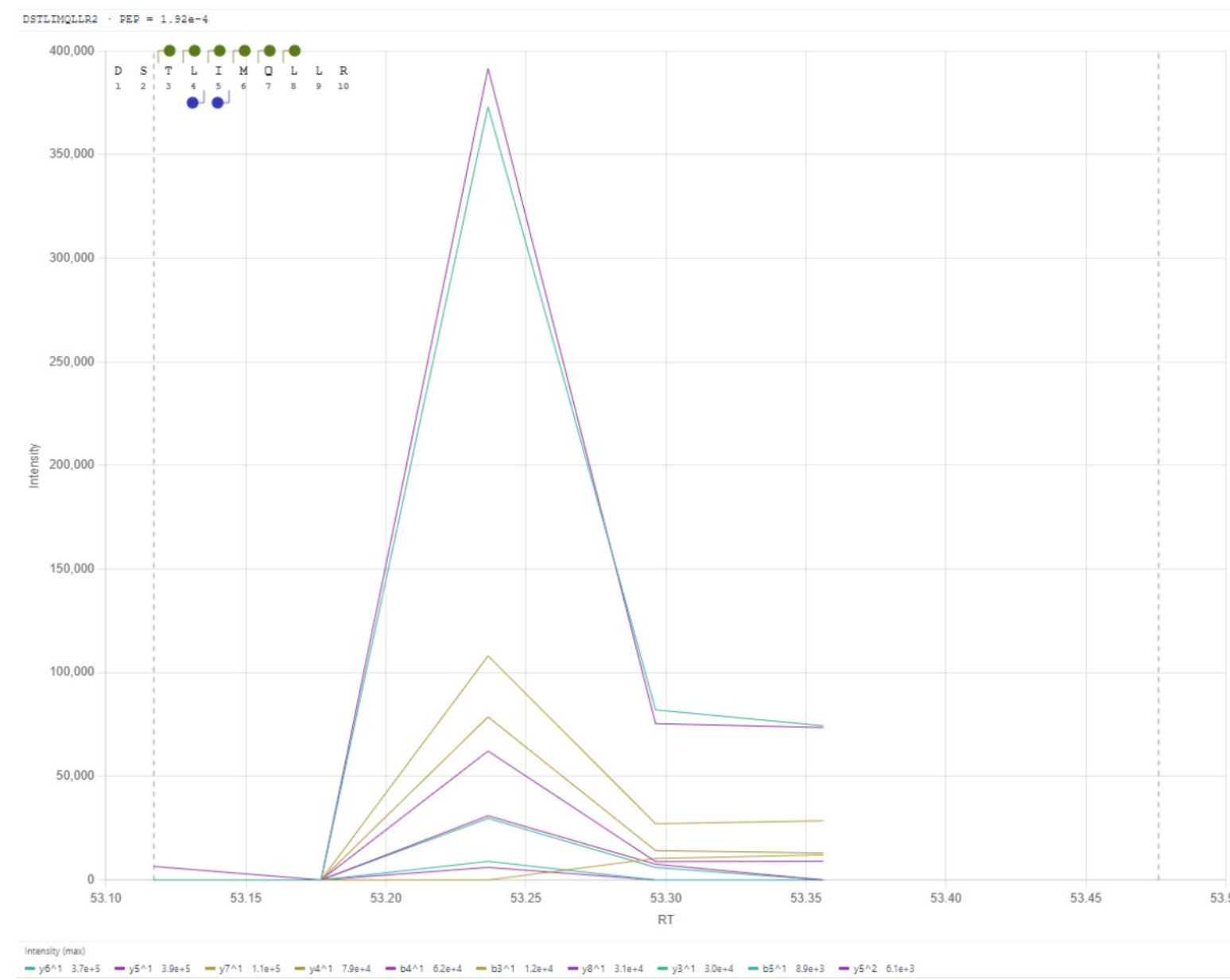
DSTLIMQLLR2 · PEP = 1.92e-4
D S T L I M Q L L R
1 2 3 4 5 6 7 8 9 10
400,000
350,000
300,000
250,000
200,000
150,000
100,000
50,000
0
Intensity
53.10
53.15
53.20
53.25
53.30
53.35
53.40
53.45
53.50
RT
Intensity (max)
y6^1 3.7e+5
y5^1 3.9e+5
y7^1 1.1e+5
y4^1 7.9e+4
b4^1 6.2e+4
b3^1 1.2e+4
y8^1 3.1e+4
y3^1 3.0e+4
b5^1 8.9e+3
y5^2 6.1e+3
